# Alteration in the Immune Microenvironment Based on APC Status in MSS/pMMR Colon Cancer

**DOI:** 10.1155/2022/3592990

**Published:** 2022-07-27

**Authors:** Haishan Lin, Bangwei Cao

**Affiliations:** Cancer Centre, Beijing Friendship Hospital, Capital Medical University, Beijing 100050, China

## Abstract

**Introduction:**

Immunotherapy is currently the most promising antitumor treatment approach. However, the colon cancer immunotherapy indication dMMR/MSI-H do not cover all colon cancer patients suitable for immunotherapy. We performed transcriptome-wide expression profile analyses of pMMR/MSS colon adenocarcinoma (COAD) specimens from TCGA database to identify a genetype signature associated with tumor immune microenvironment types (TIMTs).

**Methods:**

TCGA database was used to identify tumor genotypes suitable for antitumor immunotherapy. We analyzed RNA-sequencing profiles of 338 COAD targeted to the pMMR/MSS group from TCGA public dataset. The ESTIMATE and the CIBERSORT were used to analyze the pMMR/MSS COAD immune microenvironment between APC wild and APC mutation. Furthermore, we further verified the relationship between APC genotype and TIMTs and the efficacy of immunotherapy in 42 colon cancer specimens.

**Results:**

We identified that in APC-wt/MSS colon cancer, the expressions of PD-1, PD-L1, CTLA4, and CYT (GZMA and PRF1) were increased. The TMB, Immunoscore, and the proportion of CT8+ T cell infiltration also were identified increasing in these patients. And pathway enrichment analysis for differentially expressed genes (DEGs) between APC-wt and APC-mt MSS COAD was done to further explore their biological function. Similarly, the significant pathways for DEGs were mainly enriched in the immune response, extracellular matrix, and cell adhesion which involved in immune response. Specimens from 42 colon cancer patients, including 22 APC-mt/MSS and 20 APC-wt/MSS, were immunohistochemically evaluated for expression of CD8 and PD-L1. And APC-wt/MSS tumors showed significantly higher expression of CD8 and PD-L1 than APC-mt/MSS tumor. Moreover, APC-wt was compared with APC-mt MSS/pMMR colon cancer (DOR, 45% and 26.7%, respectively; *P* < 0.05).

**Conclusion:**

Based on the results, we found that more colon cancers of APC-wt/MSS are classified by TMIT I. And APC-wt/MSS colon cancer patients are more likely to benefit from antitumor immunotherapy.

## 1. Introduction

Tumor immune microenvironment, known as the tumor “seventh major marker” [1], is composed of innate and adaptive immune cells, cytokines, and cell surface molecules. These immune components constitute a complex regulatory network and play an important role in tumor genesis and development, wherein the development of immune checkpoint pathways is a major mechanism by which tumors evade immune surveillance [2]. Immune checkpoints refer to inhibitory pathways in the immune system that are essential for maintaining self-tolerance and minimizing chronic autoimmune inflammation [3]. Use of immune checkpoint inhibitors is one of the treatment methods that reactivates antitumor immunity. Currently, the approval of antitumor immunotherapy at clinical brake sites of the immune response include agents directed against CTLA-4 (ipilimumab) and programmed cell death protein 1 (PD-1; nivolumab and pembrolizumab) or PD-1 ligand 1 (PD-L1; atezolizumab, durvalumab, and avelumab) [4–7].

Despite these advances, only a few patients with advanced/metastatic colon cancer respond to immune checkpoint inhibitors, thereby exposing the remaining patients to potentially ineffective, toxic, and expensive treatments. Therefore, biomarkers are needed to predict the response and guide clinical treatment decisions. For example, in colon cancer, NCCN guidelines currently approve immunotherapy for MSI-H/dMMR patients. These patients comprise less than 15% for the sporadic colon cancer patient population [8–10]. At present, the overall clinical response rate of colon cancer to immunotherapy is higher than this rate [11]. For colon cancer patients with MSS/pMMR, other biomarkers are needed to predict the efficacy of immunotherapy.

There are many molecular types associated with colon cancer. About 70% of sporadic colon cancers are caused by inactivation of the tumor suppressor gene for biallelic APC, resulting in abnormal activation of the WNT/*β*-catenin signaling pathway [12]. Most APC-mutation (APC-mt) cancers are assumed to have developed through the classic adenoma-cancer pathway. Other major routes for colorectal cancer development account for another 15% to 20% of the cases [13]. Typical manifestations of this pathway are precursor sessile adenoma, wild-type APC (APC-wt), BRAF mutation, characteristic CpG island methylation phenotype, poor differentiation, and mucosa histology [14]. Here are reports on studies of the effect of APC status on the immune microenvironment of MSS colon cancer, aiming to identify its TIMT and determine whether it might be a potential predictor for immunotherapy efficacy in these patients.

## 2. Methods

### 2.1. Database

The somatic mutation status data (identified by VarScan2), gene expression data, and corresponding clinical information of COAD were downloaded from The Cancer Genome Atlas (TCGA) website (https://portal.gdc.cancer.gov/repository). 390 samples with RNA-sequencing data and somatic mutation status data were subjected to further study.

### 2.2. Immune Scores and Stromal Scores

Immune scores and stromal scores were calculated by applying the ESTIMATE algorithm.

### 2.3. Identification of Differentially Expressed Genes (DEGs)

Package limma was used to perform data analysis. Cutoffs were set as fold change > 1.5 and adj.*P* < 0.05 to screen for differentially expressed genes (DEGs).

### 2.4. Overall Survival Curve

Kaplan-Meier plots were used to illustrate the relationship between gene expression levels of DEGs and patients' overall survival. The relationship was tested by log-rank test.

### 2.5. Enrichment Analysis of DEGs

Functional enrichment analysis of DEGs was performed to identify GO categories by their molecular functions (MF), biological processes (BP), or cellular components (CC). Pathway enrichment was analyzed with reference from KEGG (Kyoto Encyclopedia of Genes and Genomes) pathways. False discovery rate (FDR) < 0.05 was used as the cut-off.

### 2.6. Classification of Immune DEG Status

Two different immune status groups (cluster 1 and cluster 2) among 497 immune DEGs of APC wt/mt colon cancer were selected by using ConsensusClusterPlus package with 50 iterations, resample rate of 0.8. The differential expressions of these genes between tumor samples and normal samples between cluster 1 and cluster 2 were analyzed by limma package with a cut-off *P* < 0.05, then visualized by pheatmap.

### 2.7. Estimation of Immune Cell Type Fractions

The CIBERSORT was used to quantify the proportion of immune cells in COAD samples from microarray data. The normalized gene expression data was analyzed using the CIBERSORT algorithm, and there were 1,000 permutations. The CIBERSORT *P* value reflects the statistical significance of the results; the recommended threshold is <0.05.

### 2.8. Construction and Validation of an Immunoscore Prognostic Model

Using the univariate Cox proportional hazards regression model, we calculated the risk proportion of DEGs in the GEO cohort. We analyzed DEGs with *P* < 0.05 and used LASSO to screen out the most useful prognostic genes in DEGs. Establish an immune score model to predict the patient's survival formula: immune score = gene Xi′s S Cox coefficient × gene Xi scale expression value.

### 2.9. Immunohistochemistry

Immunohistochemical expression of TIMTs markers (CD8 and PD-L1) was investigated in all patients. Each tumor sample was fixed in formalin and embedded in paraffin. The blocks were sliced into 5 *μ*m-thick sections, which were deparaffinized in Histo-Clear (Cosmo Bio), hydrated in a graded series of alcohols, and subjected to heat-activated antigen retrieval. After blocking endogenous peroxidase activity, the tissue was incubated with CD8 (rabbit monoclonal antibody; ab237709; Abcam; ready to use) and PD-L1 (rabbit monoclonal antibody; ab237726; Abcam) antibodies for 4 hours at room temperature. Subsequently, the sections were washed and incubated with biotinylated secondary antibody for 30 minutes at room temperature. The reaction complexes were visualized with diaminobenzidine and counterstained with hematoxylin.

### 2.10. Statistical Analysis

The two normally distributed variables used the unpaired *t* test to estimate the statistical significance of the use of the survminer package to evaluate the best cut-off value based on the association between the overall survival and immune score of each dataset. Logistics regression model was used to calculate the hazard ratio of univariate analysis. In order to select the most useful prognostic genes, we applied the LASSO Cox regression algorithm to the genes related to the prognosis. Receiver-operating characteristic (ROC) was used to describe the sensitivity and specificity of survival prediction based on immune score, and timeROC R package was used to quantify the area under the curve (AUC). Subgroup survival curves were generated by Kaplan-Meier method, and log-rank test showed statistically significant differences. Multivariate Cox regression analysis determined independent prognostic factors; only patients with comprehensive clinical data were included. All statistical analyses were performed using R software. All statistical tests are two-tailed tests, *P* < 0.05 is considered statistically significant.

## 3. Results

### 3.1. Molecular Features and Clinicopathological Assessment of COAD Patients

There are 462 colon cancer patient data in TCGA database, including 390 cases with SNP and transcriptome data. The median age at diagnosis is 68 years. There are 52 patients with MSI-H/dMMR, and 338 with MSS/pMMR, including 261 with APC-mt and 77 with APC-wt.

**Figure 1:**
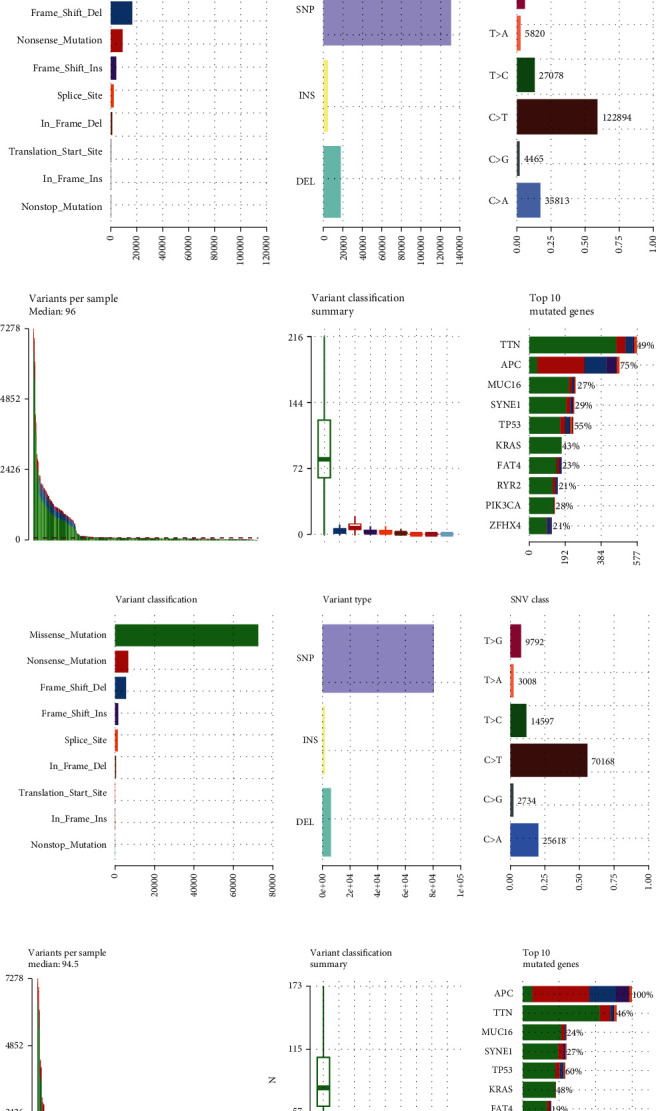
Genomic landscape and clinicopathological findings in COAD samples, based on APC status in the cohort retrieved from TCGA (The Cancer Genome Atlas). (a) Frequency and type of mutations in the top 30 COAD-associated genes. Genes were sorted according to the frequency of mutations. (b) Interactions among mutations in the top 25 genes in COAD. (c) Summary of frequency and classification of mutations in the top 10 COAD-associated genes. (d) Summary of frequency and classification of mutations in the top 10 in APC-mt/MSS COAD-associated genes. (e) Summary of frequency and classification of mutations in the top 10 genes in APC-wt/MSS COAD-associated genes.

Of the 390 colon cancer patients with SNP and transcriptome data, APC mutations were detected in 75% (293 of 390) of the tumors (Figures 1(a) and 1(c)). In the APC-mt cases, the frequencies of KRAS and TP53 mutations were 48.0% and 60%, respectively (Figure 1(d)). In the APC-wt cases, the KRAS and TP53 mutation rates were 33% and 42%, respectively (Figure 1(e)). Moreover, the BRAF mutation rate was significantly increased by 36% in this group compared to the APC-mt group.

### 3.2. Comparison of Gene Expression Profile and Different Gene Subtypes in Colon Cancer Based on Immune Scores, Stromal Scores, and Tumor Mutation Burden (TMB)

**Figure 2:**
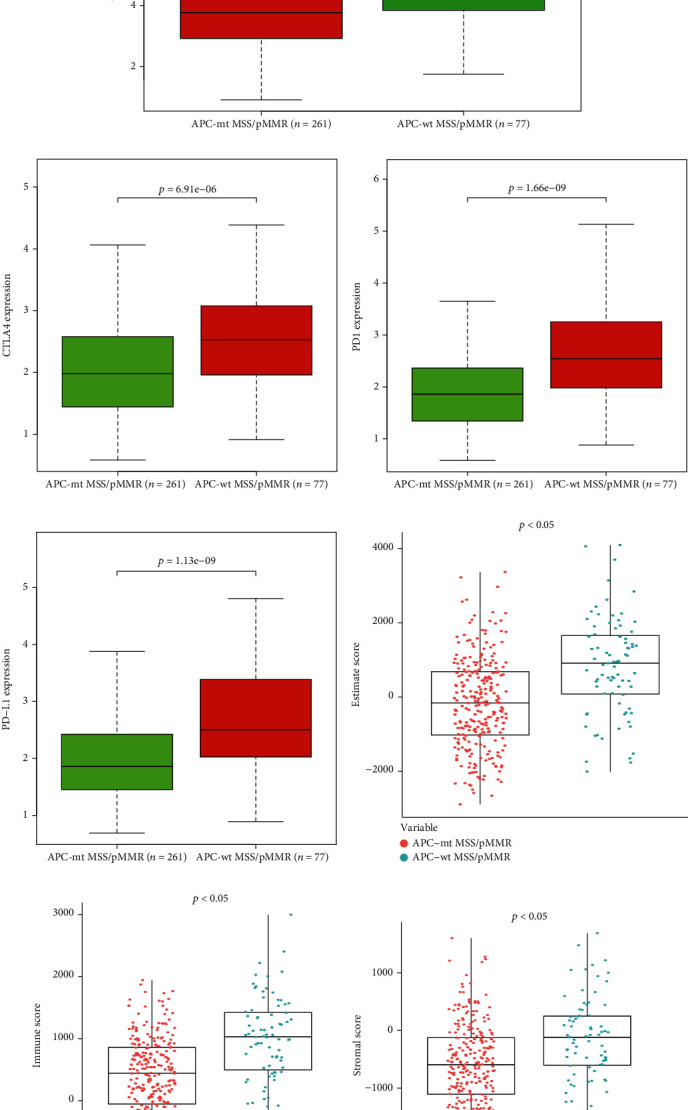
Immune score, stromal score, and TMB are associated with APC mutation status. (a, b) PRF1 and GZMA were significantly higher expression in APC-wt/MSS than APC-mt/MSS colon cancer. (c–e) Expression of CTLA4, PD-1, and PD-L1 in MSS/pMMR colon cancer with different APC gene subtypes. (f–h) Distribution of ESTIMATE score, immune score, and stromal score for APC-wt and APC-mt MSS/pMMR colon cancer. (i) Distribution of TMB for APC-wt and APC-mt MSS/pMMR colon cancer. ^∗^*P* < 0.05, ^∗∗^*P* < 0.01, ^∗∗∗^*P* < 0.001.

We compared the overall gene expression profile of all 338 colon cancer cases obtained from the TCGA database, to reveal the relationship between APC-wt and APC-mt MSS/pMMR colon cancer. And all different expression genes (DEGs) between APC-wt and APC-mt MSS/pMMR colon cancer were showed in Supplement Table S1. In the APC-mt/MSS group, 379 genes were downregulated, and 117 genes were upregulated (fold change > 1.5, *P* < 0.05; Supplementary Table S2). Immune cell cytolytic activity (CYT) might be used to assess TILs including CD8+ CTL and other immune cells (e.g., natural killer T cells). CYT was measured by the mRNA expression levels of granzyme A (GZMA) and perforin 1 (PRF1) [15]. Figures 2(a) and 2(b) showed that PRF1 and GZMA were significantly higher expression in APC-wt/MSS than APC-mt/MSS colon cancer. In MSS/pMMR colon cancer, the expression of immune checkpoint genes such as CTLA4, PD-1, and PD-L1 in the APC-mt/MSS group was significantly lower than in the APC-wt/MSS group (Figures 2(c)–2(e)). In addition, the ESTIMATE score, immune score, and stromal score calculated by the ESTIMATE were significantly lower in the APC-mt/MSS group than in the APC-wt/MSS group (Figures 2(f)–2(h)). Compared with APC-wt/MSS group, the TMB was significantly lower in the APC-mt/MSS group than in the APC-wt/MSS group (Figure 2(i)). These results suggest that the proportion of immune-related components and expression of immune checkpoint are higher in the APC-wt/MSS colon cancer tumor microenvironment. The presence of APC-wt/MSS, combined with TMB, is consistent with this increase. It can, therefore, be presumed that APC-wt/MSS colon cancer could become a beneficiary of immune checkpoint inhibitors. A follow-up research will need to focus on further exploration in this direction.

Furthermore, we find that not only the expression of PD-1, PD-L1, and CTLA4 but also immune score, ESTIMATE score, and stromal score did not differ between TP53-wt/MSS and TP53-mt/MSS groups (Supplement Figure 2). However, the expression of CTLA4, PD-L1 and immune score and stromal score in the KRAS-mt/MSS group was downregulated compared with KRAS-wt/MSS (Supplement Figure 1). The difference for the KRAS genetypes were smaller than that between the APC genetypes. Mutations in KRAS or TP53 did not affect the TMB. In the combination of different genotypes of KRAS, TP53, and APC, the TMB and immune score are significantly higher in wild type than KRAS/TP53/APC mutant type colon cancer (Supplement Figure 3).

### 3.3. Composition of Immune Cells in MSS/pMMR Colon Cancer with Different Genetic Subtypes

**Figure 3:**
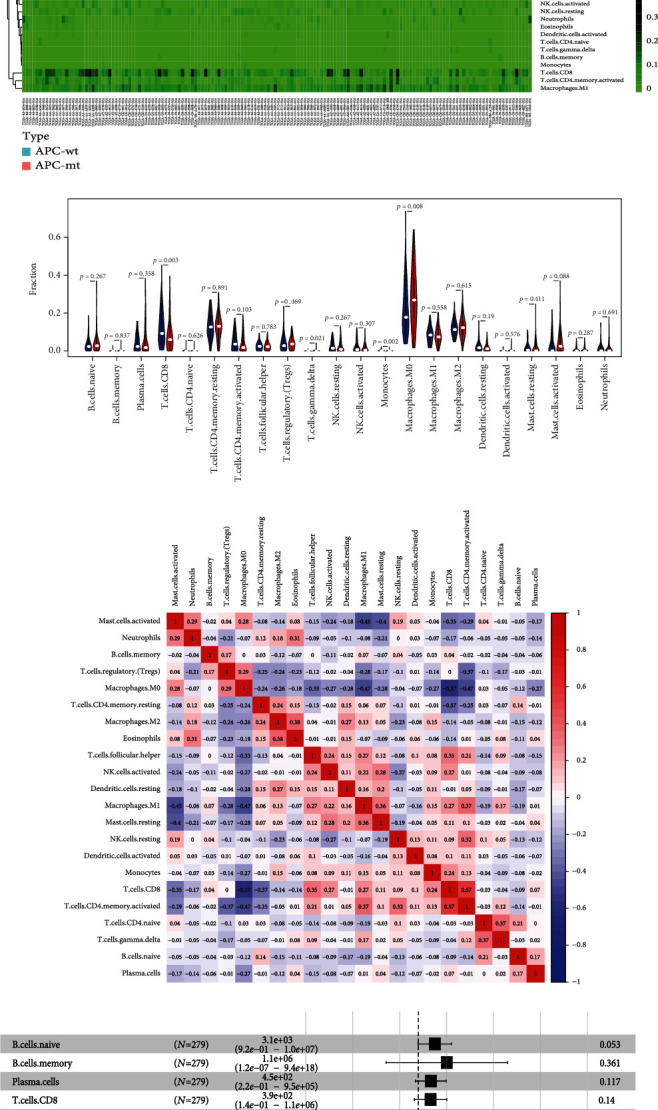
Composition of infiltrated immune cells in association with different genetic subtypes in the cohort retrieved from TCGA. The CIBERSORT tool deemed all samples eligible at *P* < 0.05. Twenty different immune cells were filtered and analyzed in the cohort retrieved from TCGA. (a) Fractions of immune cells in the 338 MSS/pMMR colon cancer samples from TCGA. (b) Comparisons of immune cells between APC-mt/MSS and APC-wt/MSS colon cancer tissues from TCGA. (c) Interaction among the 20 different immune cells in MSS/pMMR colon cancer. (d) Forest plots showing an association between different immune cell subsets in the cohort retrieved from TCGA. (e) Logistics regression analysis the PD-L1 expression the affecting factors.

We studied the proportion of immune cells infiltrating between different genetic subtypes in the colon cancer cohort retrieved from TCGA. All 338 MSS/pMMR colon cancer samples met CIBERSORT requirements at *P* < 0.05. The proportion of CD8+ T cells is significantly lower, and the proportion of M0 macrophages is significantly higher in APC-mt/MSS colon cancer in comparison to APC-wt/MSS (Figures 3(a)–3(c)). However, neither mutations in KRAS nor TP53 could affect the proportion of infiltrating immune cell types in MSS/pMMR colon cancer (Supplement Figure 4). Furthermore, in the application of logistics regression analyses, we tried to take the expression of PD-L1 as the dependent variables, analysis on multiple factors such as the degree of infiltration of various immune cells, and wild type and mutant type of APC. We found the that APC gene type and CD4 memory cells, regulatory (Tregs) cell, NK cells, monocytes, and dendritic cells are associated with the expression level of PD-L1. The immune-promoting lymphocyte infiltration ratio, such as CD8+ T cells, and the expression ratio of PD-1, the immune checkpoints, have significantly increased in APC-wt/MSS colon cancer (Figure 3(e)). Combining the results from Figures 2 and 3, APC-wt/MSS has a higher percentage of immune-related components infiltration in the tumor microenvironment compared to APC-mt/MSS colon cancer. It is speculated that APC-wt colon cancer is more likely to be a “hot tumor,” and is more likely to benefit from antitumor immunotherapy.

### 3.4. Relationship between Immune Status and APC Mutations in MSS/pMMR COAD Patients

**Figure 4:**
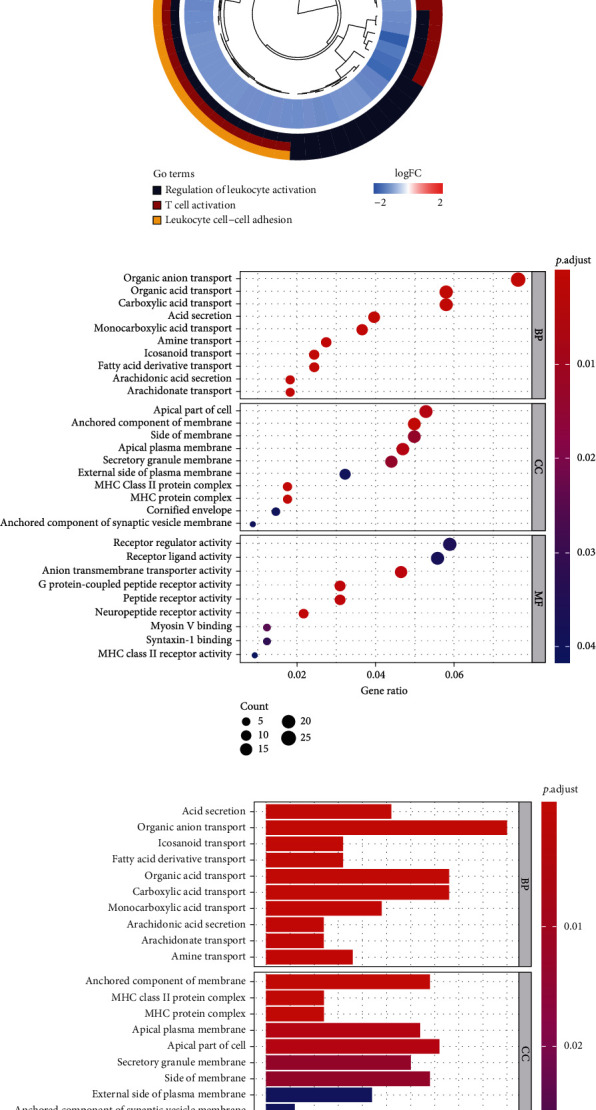
Genomic landscape and gene set enrichment analysis of the COAD samples, based on APC status in the cohort retrieved from TCGA (The Cancer Genome Atlas). (a) The immunity and cancer pathways that are significantly enriched in APC-wt/MSS COAD patients, compared with those in APC-mt/MSS COAD patients. (b, c) Gene Ontology (GO) analysis of the immune-related DEGs. Circular plot of GO pathways enrich in APC-wt/MSS samples. GO pathways cluster distribution. (d, e) GO analysis of the immune-related DEGs. Immune-related DEGs in the significantly enriched immunologic and cancer biological processes. (f, g) KEGG analysis of immune-related DEGs.

We divided the MSS/pMMR COAD samples in the cohort retrieved from TCGA into APC-wt (77 samples) and APC-mt (261 samples) groups and performed gene set enrichment analysis (GSEA). The results show that APC-wt colon cancer was significantly enriched in 115 KEGG pathways (*P* < 0.05; Supplementary Table S3-S4). These include pathways related to immune signaling, such as natural killer cell-mediated cytotoxicity, leukocyte transendothelial migration, NOD-like receptor signaling, TOLL-like receptor signaling, TGF-*β* signaling, and other immune-related signaling pathways (Figure 4(a)). Circular plot of GO pathways was enriched by processes regulating leukocyte and T cell activation and leukocyte cell−cell adhesion (Figures 4(b) and 4(c)). We then performed Gene Ontology (GO) and Kyoto Encyclopedia of Genes and Genomes (KEGG) analysis of the immune-related DEGs in APC-wt/MSS colon cancer (Figures 4(d)–4(g)). These findings to further determine that APC mutations play a role in the immune response of colon cancer.

### 3.5. Calculation and Validation of the Immunoscore, and Evaluation of Its Prognostic Ability in the COAD Cohort Retrieved from TCGA

**Figure 5:**
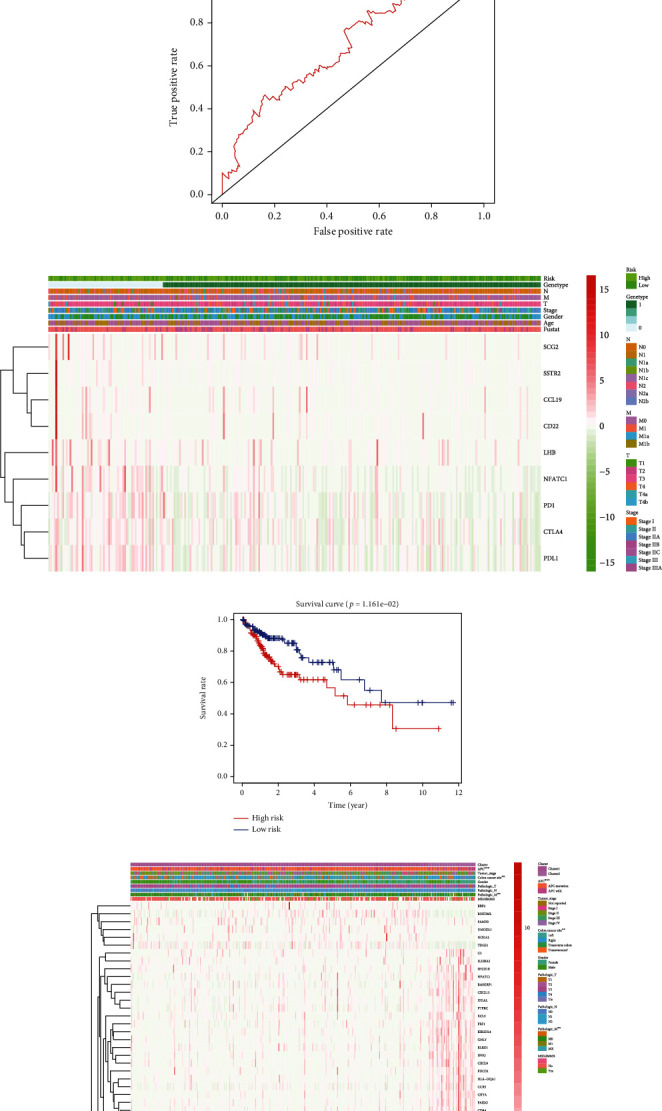
Immune-related DEGs and construction of the Immunoscore model. (a, b) Lasso coefficient profiles of 8 genes were related to prognosis. The optimal values of the penalty parameter *λ* were determined by tenfold crossvalidation. (c–e) Patients were stratified based on low or high Immunoscore (low or high score). Kaplan-Meier curves, heatmap, and time-dependent ROC curve in the cohort retrieved from TCGA. (g) Consensus matrix for *k* = 2. (h) The overall survival curves of cluster 1 and cluster 2 estimated by the Kaplan-Meier plotter. (f) The heatmap shows the expression of the powerful prognostic markers in cluster 1 and cluster 2 (^∗^*P* < 0.05, ^∗∗^*P* < 0.01, ^∗∗∗^*P* < 0.001, ^∗∗∗∗^*P* < 0.0001).

We have identified 65 overlapping genes (shown in Table S5) among the DEGs (496 genes related to APC status, shown in Supplementary Table S1) and the DEGs related to immunophenotypes (1297 genes shown in Supplementary Table S6). Using Lasso and Cox regression analyses, the eight genes with the highest prognostic value were identified (Figures 5(a) and 5(b)). We then selected these genes to establish an immune scoring model, which was assessed for its ability to predict the prognosis of COAD patients. The formula of the immune scoring model is described in the “Methods” section. Next, we divided the COAD patients into a high-score and low-score groups based on an optimal cutoff value of the immune score (shown in Supplementary Table S7) obtained by the survminer R package.  Figure 3(c) shows that the area under the ROC curve (AUC) of the 5-years OS prognostic model is 0.614.  Figure 3(d) shows the immune score distribution and selected gene expression data.

Figure 5(e) shows that patients with a high score had a worse OS than those with a low score.

### 3.6. Consensus Clustering Identified Two Clusters of Immune-Related DEGs from APC-wt/mt

We selected 65 overlapping immune-related DEGs. These genes were defined based on APC gene statue shown in Table S5 for details. These immune-related gene of COAD using ConsensusClusterPlus dividing 462 colon cancer patients into two categories (Figure 5(g)). The results of Kaplan-Meier analysis showed that the cluster 2 group had significantly worse prognosis compared with cluster 1 group (Figure 5(h)). The two clusters grouped by the immune-related DEGs were shown in heatmap and the difference in clinical characteristics between the two groups. Heatmap showed the metastasis rate difference and expression difference of the immune-related DEGs between two clusters (Figure 5(f)).

### 3.7. APC-mt/wt MSS/pMMR Colon Cancer Expression of Markers for TIMTs and Response to ICIs

**Figure 6:**
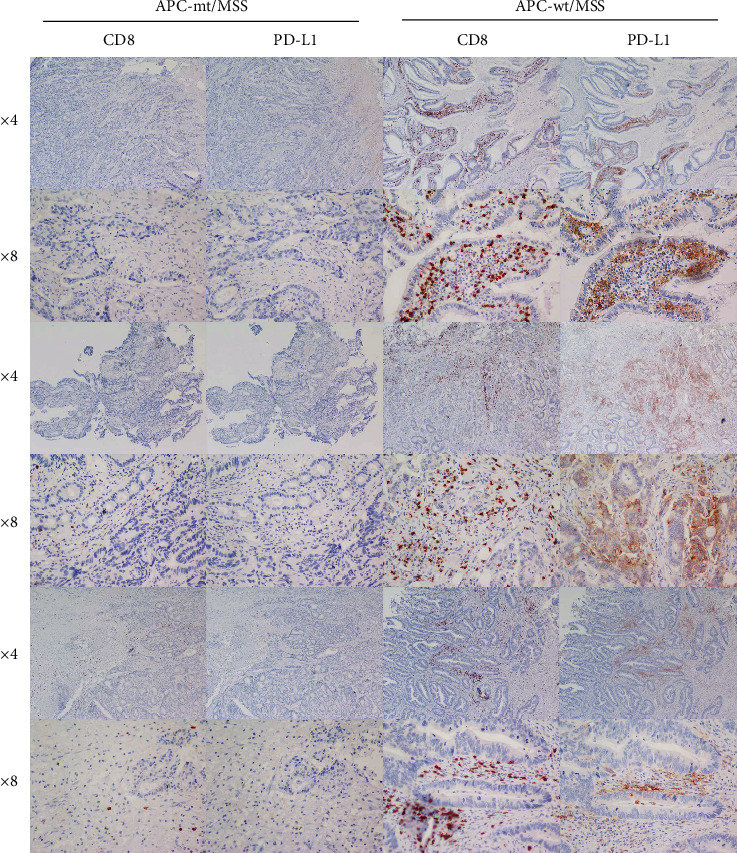
Histology (H&E: hematoxylin and eosin) and immunohistochemistry showing four different expression patterns of CD8 and PD-L1 in the representative case. We identified patients with 22 APC-mt/MSS (left) and 20 APC-wt/MSS (right) colon cancer. APC-wt/MSS is significantly higher than APC-mt/MSS in the positive rate and the degrees of positive expression for CD8 and PD-L1 with immunostaining.

We gathered 42 cases of MSS/pMMR colon cancer tissues, including 22 APC-mt and 20 APC-wt. 8/22 APC-mt MSS/pMMR colon cancer (36.3%) was immunopositive for CD8, and 12/20 APC-wt MSS/pMMR colon cancer (60%) was immunopositive for CD8. 5/22 APC-mt MSS/pMMR colon cancer (22.5%) was immunopositive for PD-L1, and 11/20 APC-wt MSS/pMMR colon cancer (55%) was immunopositive for PD-L1 (Figure 6). In addition to the positive rate, the degrees of positive expression for CD8 and PD-L1, APC-wt/MSS is significantly higher than APC-mt/MSS with immunostaining. TIMTs divide tumors into four categories based on the presence or absence of TILs and PD-L1 expression levels (type I: TILs+ and PD- L1+; type II: TILs- and PD-L1-; type III: TILs+ and PD- L1-; type IV: TILs- and PD-L1+). The 42 patients, containing APC-mt MSS/pMMR and APC-wt MSS/pMMR two groups, were divided into one of the above four types according to expression patterns of markers CD8 and PD-L1. 2/22 APC-m MSS/pMMR colon cancer (9.1%) was immunopositive for both CD8 and PD-L1 (TIMT I). 6/22 APC-mt MSS/pMMR colon cancer (27.2%) was immunopositive for only CD8 (TIMT III). 3/22 APC-mt MSS/pMMR colon cancer (13.6%) was immunopositive for only PD-L1 (TIMT IV). 11/22 APC-mt MSS/pMMR colon cancer (50%) was immunonegative for either CD8 or PD-L1 (TIMT II). 10/20 APC-wt MSS/pMMR colon cancer (50%) was immunopositive for both CD8 and PD-L1 (TIMT I). 2/20 APC-wt MSS/pMMR colon cancer (10%) was immunopositive for only CD8 (TIMT III). 1/20 APC-wt MSS/pMMR colon cancer (5%) was immunopositive for only PD-L1 (TIMT IV). 7/20 APC-wt MSS/pMMR colon cancer (35%) was immunonegative for either CD8 or PD-L1 (TIMT II). Therefore, APC-wt MSS/pMMR mainly includes TIMT I type colon cancer, which was consistent with the statistical result in the TCGA database. There is no difference in clinical characteristics between the two groups (Table 1).

In these colon cancer patients, 20 APC-wt and 15 APC-mt MSS/pMMR received ICIs combined chemotherapy. And patients' first-line and second-line treatments using ICIs were 12/8 and 10/5, respectively. The combined chemotherapy regimen was mainly 5-FU and oxaliplatin. Interestingly, APC-wt MSS/pMMR colon cancer patients have a higher DCR for ICIs (Table 2).

## 4. Discussion

As cancer treatment has entered a new era of immunotherapy, maximizing screening of patients with cancer suitable for immunotherapy can lead to better outcomes for more patients. Transcriptomic analysis was performed on pMMR/MSS colon cancer patients to identify potential beneficiaries of immunotherapy beyond existing colon cancer indications. In the current work, we calculated TMB, immune scores, the expression of immune checkpoints and immune cell infiltrations by comparing the commonly mutated genotypes in colon cancer targeted to pMMR/MSS COAD from TCGA dataset. A four-tiered classification for tumor microenvironment immune type (TMIT) has been proposed to describe the patient's immune status and to determine immunotherapy-responsive subgroups [16–18]. The four TMIT types are defined as follows: type I, PD-L1-positive with TIL (CD8/CYT-positive) (adaptive immune resistance); type II, PD-L1-negative with no TIL (immune ignorance); type III, PD-L1-positive with no TIL (intrinsic induction); and type IV, PD-L1-negative with TIL (possible role of other suppressors in producing immune tolerance) [19–21]. It seems that APC-wt/MSS colon cancer is more likely to be TMIT1, which is more expected to benefit from immunotherapy.

**Figure 7:**
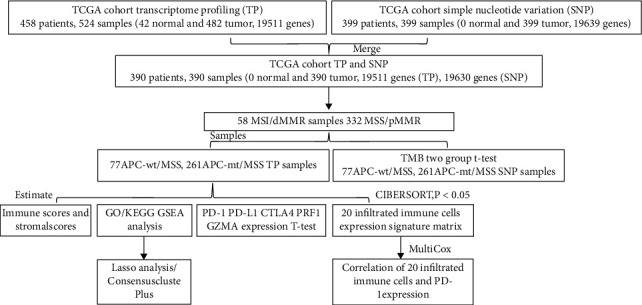
Work flow of the current work.

We divided 338 pMMR/MSS colon cancer from TCGA database into 261 APC-mt and 77 APC-wt. The expression of immune checkpoint genes such as PD-1, PD-L1, and CTLA4 in the APC-mt/MSS group was significantly lower than in the APC-wt/MSS group. In addition, the immune score and stromal score calculated by the ESTIMATE were significantly lower in the APC-mt/MSS group than in the APC-wt/MSS group. The sequencing results of the two groups were used to calculate the TMB for comparison. Moreover, compared with APC-wt/MSS group, the TMB was significantly lower in the APC-mt/MSS group than in the APC-wt/MSS. CIBERSORT was used to calculate the ratio of infiltrating immune cells between the two groups. And the proportion of CD8+ T cells is significantly lower, and the proportion of M0 macrophages is significantly higher in APC-mt/MSS colon cancer in comparison to APC-wt/MSS. We further use GO and KEGG to analyze the 496 DEGs between the APC-wt/MSS and APC-mt/MSS groups and enrich mainly immune-related signaling pathways. Out of these, we performed Cox regression analysis on the 65 immune-related DEGs and established an immune scoring model, which was assessed for its ability to predict the prognosis of COAD patients (Figure 7). In addition, we selected 42 patients' colon cancer tissues, including 22 cases of APC-mt/MSS and 20 APC-wt/MSS, to performed immunohistochemistry to further confirm that APC-wt/MSS contained more TIMT type I tumors, and had a higher DCR for ICIs. A preprint has previously been published [22]. APC is a protein that assists in inactivating *β*-catenin in the Wnt pathway [23]. When it is mutated, it can cause abnormal activation of the Wnt pathway [24]. Mechanistically, this occurs as a result of the *β*-catenin/TCF4 complex binding to the PD-L1 promoter, leading to increased transcription, so that APC mutations can induce tumor immune evasion via an immune checkpoint pathway [25].

## 5. Conclusions

Theoretically, these mechanisms provide support to the conclusions of this study.

In summary, from analysis of the data retrieved from TCGA database, using immune scores based on the ESTIMATE and CIBERSORT algorithms, we found that APC-wt MSS/pMMR colon cancer is more likely to be a TIMT I tumor, and these findings have been partially confirmed in the tissues of colon cancer patients. Therefore, we speculate that patients with APC-wt/mss COAD could potentially benefit from immunotherapy.

## Figures and Tables

**Table 1:** Clinical characteristics of patients with APC-mt and APC-wt MSS/pMMR colon cancer.

Characteristics	APC-mt MSS/pMMR patients	APC-wt MSS/pMMR patients
Patients (*n*)	22	20
Age (years), median (range)	67.5 (42-89)	66.4 (43-89)
>60 years (*n*)	14	12
≤60 years (*n*)	8	8
Sex (male/female) (*n*/*n*)	10/12	9/11
KPS score (%), median (range)	80 (30-100)	80 (20-100)
≥60% (*n* (%))	18	17
<60% (*n* (%))	4	3
Stage I (*n*)	0	0
Stage II (*n*)	4	3
Stage III (*n*)	10	10
Stage IV (*n*)	8	7

**Table 2:** APC-mt and APC-wt MSS/pMMR colon cancer patients' DCR for ICIs+chemotherapy.

	APC-mt MSS/pMMR patients	APC-wt MSS/pMMR patients
Treated with ICIs patients (*n*)	20	15
ICIs in the first-line (*n*)	12	10
CR	0	0
PR	2	0
SD	4	3
PD	6	7
ICIs in the second-line (*n*)	8	5
CR	0	0
PR	1	0
SD	2	1
PD	5	4
Combined chemotherapy	20	15
XELOX	11	8
FORFOX	9	7
DCR in the first-line	6/12	3/10
DCR in the second-line	3/8	1/5
DCR in total	45%	26.7%

## Data Availability

The data generated during this study are available from the corresponding author upon reasonable request. The data that support the findings of this study are available in The Cancer Genome Atlas (TCGA) website. These data were derived from the following resources available in the public domain: https://portal.gdc.cancer.gov/repository.

## Supplementary Materials

Supplementary Materials Supplement Table S1: all different expression genes (DEGs) listed between APC-wt and APC-mt MSS/pMMR colon cancer from the overall gene expression profile of all 338 colon cancer cases obtained from TCGA database. Supplementary Table S2: APC-mt vs. APC-wt MSS/pMMR colon cancer, 379 genes were downregulated, and 117 genes were upregulated. Supplementary Table S3: APC-wt MSS/pMMR (77 samples) COAD samples in the cohort retrieved from TCGA performed gene set enrichment analysis enriched (GSEA) in 115 KEGG pathways. Supplementary Table S4: APC-wt MSS/pMMR COAD samples in the cohort retrieved from TCGA performed gene set enrichment analysis downregulated (GSEA) in 62 KEGG pathways. Supplementary Table S5: 65 overlapping genes among the DEGs (496 genes related to APC status) and immunophenotypes (1297 genes shown in Supplementary Table S6). Supplementary Table S6: 1297 immune-related genes. Supplementary Table S7: Lasso and Cox regression analyze APC-related DEGs constructing immune scoring model. Supplement Figure 1: the expression of CTLA4, PD-L1, and immune score and stromal score in the KRAS-mt/MSS group was downregulated compared with KRAS-wt/MSS. Supplement Figure 2: the expression of not only PD-1, PD-L1, and CTLA4 but also immune score, ESTIMATE score, and stromal score did not differ between TP53-wt/MSS and TP53-mt/MSS groups. Supplement Figure 3: in the combination of different genotypes of KRAS, TP53 and APC, the TMB and immune score are significantly higher in wild type than KRAS/TP53/APC mutant type colon cancer. Supplement Figure 4: neither mutations in KRAS nor TP53 could affect the proportion of infiltrating immune cell types in MSS/pMMR colon cancer.
