# Supplementary material for: Alteration in the Immune Microenvironment Based on APC Status in MSS/pMMR Colon Cancer
Source: Dis Markers. 2022 Jul 27;2022:3592990. doi: 10.1155/2022/3592990 (PMC9348928; doi:10.1155/2022/3592990)
Supplement: Supplementary Materials — Supplement Table S1: all different expression genes (DEGs) listed between APC-wt and APC-mt MSS/pMMR colon cancer from the overall gene expression profile of all 338 colon cancer cases obtained from TCGA database. Supplementary Table S2: APC-mt vs. APC-wt MSS/pMMR colon cancer, 379 genes were downregulated, and 117 genes were upregulated. Supplementary Table S3: APC-wt MSS/pMMR (77 samples) COAD samples in the cohort retrieved from TCGA performed gene set enrichment analysis enriched (GSEA) in 115 KEGG pathways. Supplementary Table S4: APC-wt MSS/pMMR COAD samples in the cohort retrieved from TCGA performed gene set enrichment analysis downregulated (GSEA) in 62 KEGG pathways. Supplementary Table S5: 65 overlapping genes among the DEGs (496 genes related to APC status) and immunophenotypes (1297 genes shown in Supplementary Table S6). Supplementary Table S6: 1297 immune-related genes. Supplementary Table S7: Lasso and Cox regression analyze APC-related DEGs constructing immune scoring model. Supplement Figure 1: the expression of CTLA4, PD-L1, and immune score and stromal score in the KRAS-mt/MSS group was downregulated compared with KRAS-wt/MSS. Supplement Figure 2: the expression of not only PD-1, PD-L1, and CTLA4 but also immune score, ESTIMATE score, and stromal score did not differ between TP53-wt/MSS and TP53-mt/MSS groups. Supplement Figure 3: in the combination of different genotypes of KRAS, TP53 and APC, the TMB and immune score are significantly higher in wild type than KRAS/TP53/APC mutant type colon cancer. Supplement Figure 4: neither mutations in KRAS nor TP53 could affect the proportion of infiltrating immune cell types in MSS/pMMR colon cancer. [file 3592990.f1.zip › supplement table S1.pdf]

496 genes related to APC status

gene	logFC	pValue
WIF1	3.149461469	2.51E-07
CALCA	3.114956255	3.29E-07
PRSS56	3.108382806	2.35E-06
DKK4	3.075787859	0.002340537
MAGEB17	2.47475576	1.37E-11
COL2A1	2.323082113	5.20E-06
MT3	2.02840498	4.10E-05
KRT40	2.028089611	4.02E-05
SPACA3	1.963187667	5.16E-10
LY6G6F	1.932354256	2.24E-13
GRPR	1.923411005	1.64E-05
CPN1	1.816904413	6.29E-07
NOTUM	1.810395752	8.11E-08
NKX2-1	1.800101008	0.000381168
GLYATL2	1.752205175	8.65E-05
RBP2	1.74196789	9.52E-10
CALML3	1.737659618	1.74E-09
DPP10	1.732540043	0.002724876
LDHC	1.725952237	0.006795262
TMEM252	1.649384032	6.07E-06
PAH	1.634216447	7.73E-09
OBP2B	1.634048196	0.00035869
CEL	1.624909172	1.73E-13
STMND1	1.621617857	7.19E-12
MYH7B	1.597473174	1.24E-09
NKD1	1.590127559	1.12E-11
RNF182	1.550889916	7.55E-06
DRD2	1.516689153	2.73E-08
NPTX2	1.504691364	0.008828881
PLA2G12B	1.487885651	2.46E-08
LY6G6F-LY6G6D	1.483884225	3.05E-13
PTPRO	1.463846493	2.29E-13
SLC1A7	1.445199707	1.48E-05
PKLR	1.414424446	8.32E-07
ZNF541	1.405738196	8.88E-08
APCDD1	1.403267156	1.16E-08
NEUROG2	1.397246989	2.37E-06
DPEP1	1.39553697	7.85E-12
LY6G6D	1.394984245	1.57E-12
LY6G6E	1.383502802	1.13E-10
NODAL	1.338491651	9.77E-08

KRT23	1.336279475	2.06E-07
BHLHA9	1.32998965	4.80E-05
SLC30A2	1.321306442	4.69E-10
IZUMO2	1.314520122	9.80E-07
R3HDML	1.311593347	5.46E-11
SLC6A4	1.29234907	8.35E-09
MAP7D2	1.284176033	3.80E-06
KRT75	1.276057459	5.97E-06
KRT83	1.25199605	0.000744616
LRRC36	1.24143571	5.19E-12
TNNC2	1.21670727	1.84E-11
LRRC2	1.215913827	5.71E-15
ENPP3	1.213183493	7.14E-07
FAM3B	1.212663129	3.73E-05
POU5F1B	1.2042216	4.21E-11
TDGF1	1.202894144	1.66E-15
ACSL6	1.19442214	3.20E-10
SULT2A1	1.191507183	0.001066003
FSTL4	1.191489996	4.85E-05
UMODL1	1.189278067	2.65E-09
CBLN1	1.184733552	5.04E-07
SERPINA7	1.184018638	3.33E-08
PRDM13	1.180385188	1.04E-05
SERPINA10	1.172646355	2.43E-07
GRM8	1.172057318	7.26E-10
TMEM249	1.17178299	0.000662403
SLC38A3	1.167993756	3.14E-05
AFP	1.165286204	1.15E-05
PIPOX	1.161081585	0.000154668
IGFL4	1.152410217	1.17E-07
SLC13A3	1.147525158	6.15E-05
SLC39A2	1.135763546	1.10E-06
RPS6KA6	1.135399989	1.57E-10
RIIAD1	1.132473298	3.45E-06
RIPPLY3	1.12920023	5.49E-08
DLX3	1.127472564	1.27E-08
PRKAA2	1.108261874	0.001869235
TBX21	-1.102351689	4.27E-08
NFATC1	-1.104202179	1.04E-08
TRNP1	-1.107023705	1.92E-09
FSCN1	-1.107125545	4.16E-09
AC099489.1	-1.110053866	0.000835765
HLA-DPA1	-1.114991054	4.73E-08
DNAH2	-1.115345056	9.85E-05

MS4A1	-1.118757245	0.008890521
ZBTB16	-1.119886467	0.00320879
ZIC5	-1.132245787	6.60E-05
FPR2	-1.133152748	4.14E-05
IL1RN	-1.136516678	4.06E-09
LCK	-1.142086263	3.33E-08
TCL1A	-1.142302657	0.001047413
CNIH2	-1.143281419	0.005511461
XKR9	-1.144393963	6.75E-06
CD7	-1.145819493	6.60E-11
HOXC8	-1.146391573	0.000141021
FCRL6	-1.148008721	1.06E-07
MSH4	-1.149801842	0.000565222
RAB39B	-1.151450233	4.62E-05
OR2I1P	-1.154128862	0.003418761
SCG2	-1.160271075	0.000270223
DUSP4	-1.160412933	2.54E-10
SECTM1	-1.163013991	8.67E-12
GNLY	-1.164517187	6.02E-06
OSR1	-1.16906157	5.77E-13
PNMA2	-1.169148799	1.25E-05
ACKR4	-1.171485513	9.75E-07
GZMA	-1.175039065	4.57E-09
VNN3	-1.176584694	2.25E-05
SSTR2	-1.177132498	0.000324942
SRRM3	-1.177374139	0.001487221
CHIT1	-1.178416257	0.000454497
SPATC1	-1.179363139	3.15E-05
PDCD1	-1.179925758	3.84E-09
IFNG	-1.181762926	0.000232418
ZNF831	-1.183014926	7.80E-06
ABCA3	-1.183650178	7.28E-06
GABRB3	-1.184237995	0.002291615
CCL19	-1.185020222	0.006395799
AQP9	-1.18923642	4.04E-05
RARRES3	-1.191404939	6.83E-10
GPR18	-1.193640805	0.002637833
HOXD1	-1.198625206	6.07E-06
GPR174	-1.204427523	5.53E-05
KIAA1549L	-1.204488326	1.04E-05
HOXC4	-1.208433651	2.67E-08
GBP4	-1.209766333	7.08E-08
CRTAM	-1.210737943	1.21E-08
FMO2	-1.212374092	0.003371677

GZMK	-1.21249588	1.53E-07
ZNF683	-1.216695283	2.20E-08
HBA2	-1.218067887	5.97E-05
CHI3L2	-1.224274552	2.39E-05
RAB27B	-1.226616116	1.27E-12
FOXD1	-1.234813444	1.75E-06
NXPH4	-1.2372315	0.002871554
VTCN1	-1.23778041	0.000415786
SIX4	-1.23824194	0.001589264
LHB	-1.243280791	6.70E-05
RARRES1	-1.244027679	1.85E-11
B3GALT5	-1.246453902	5.58E-08
SPON1	-1.251976907	9.11E-10
KIR2DL4	-1.252475644	1.88E-09
PTPRU	-1.256448259	5.10E-07
SP140	-1.259150172	7.33E-09
HTR3A	-1.263384277	3.85E-06
PRPH	-1.264637041	0.005763151
HABP2	-1.265376471	0.000342321
AC008687.4	-1.269432505	0.009181016
TFF1	-1.274271027	0.000838827
MUC17	-1.290661123	0.00182548
TRIM7	-1.293969579	8.94E-10
CD109	-1.29809979	8.53E-08
MUCL3	-1.304493968	5.26E-07
EMX1	-1.304705545	2.44E-05
KISS1R	-1.310525561	0.002845466
CIDEC	-1.315285879	7.05E-06
SOX8	-1.319054827	2.44E-06
SNPH	-1.323432218	1.77E-09
ARX	-1.326527854	0.000146254
GRAMD1B	-1.337286223	2.93E-07
UGT1A1	-1.343234841	4.41E-05
CXCL13	-1.347946818	2.35E-08
CHST6	-1.349994537	1.15E-08
HOXC9	-1.351255132	1.24E-06
ABCB11	-1.353166316	1.99E-06
ELOVL3	-1.355005437	9.12E-08
KRT7	-1.356930492	1.46E-08
NXF3	-1.357182946	1.22E-06
ONECUT2	-1.360045236	0.000987885
SYT5	-1.362478575	1.08E-05
FER1L6	-1.363330921	4.75E-06
MYRFL	-1.375006759	0.005975398

SMTNL1	-1.37717199	6.96E-05
ZBED2	-1.381196923	8.22E-09
FCGR3B	-1.383845705	0.000115466
CIITA	-1.389340937	2.88E-09
PI3	-1.392135892	1.11E-05
FFAR2	-1.400315069	0.000184179
DHRS9	-1.403036991	0.000183436
WNT10B	-1.408411154	0.000291212
NTSR1	-1.411737062	4.25E-05
PRDM8	-1.433045805	9.27E-13
TPO	-1.437631166	1.37E-11
IL17REL	-1.440994824	1.09E-05
PLA2G2D	-1.444993628	1.93E-05
HOMER2	-1.449415433	0.000278882
VNN2	-1.454213581	8.95E-12
SAA2	-1.490833109	5.27E-05
GBP5	-1.492995841	5.71E-08
HLA-DOA	-1.493319558	5.27E-07
SNAP25	-1.519173296	0.006864076
VSIG1	-1.521921032	0.000452766
IGLON5	-1.522216748	0.000226849
EPHB6	-1.525656854	0.002349364
RSPO3	-1.535622896	1.11E-05
HOXC6	-1.539220652	2.02E-07
APLP1	-1.548996434	3.65E-06
CTSE	-1.549851431	1.86E-07
RTBDN	-1.553766703	5.43E-05
CLCA4	-1.556697814	0.007249952
COLGALT2	-1.560549888	0.002611456
GABRP	-1.564248927	1.97E-05
IDO1	-1.565476994	5.02E-05
ADRB1	-1.586929596	1.13E-08
WNT7B	-1.587713279	0.000472194
DRGX	-1.594141689	1.20E-05
CCK	-1.599062644	3.94E-06
PSCA	-1.604043897	0.004234871
S100A8	-1.615885886	0.000114037
SAA2-SAA4	-1.615975766	0.001232727
NEUROG3	-1.619526126	0.000156804
TUBB4A	-1.631540249	0.001631051
UNC5A	-1.671709978	3.12E-05
SAA1	-1.699237463	7.21E-05
FAM83A	-1.704533274	0.000353221
NMUR2	-1.723588025	8.01E-05

HMX3	-1.731055483	1.27E-05
HRASLS	-1.739706065	2.75E-05
ADGRF1	-1.776943114	0.001040891
MUC5AC	-1.803249466	0.002581158
SMIM18	-1.80870464	0.007852343
PM20D1	-1.822045971	0.004072436
CAMK2N2	-1.831581382	0.000128003
F5	-1.927891703	6.94E-05
LY6D	-1.940482746	0.000400518
ANXA10	-1.996687062	9.00E-09
LEP	-2.040366607	0.006997419
CRYBA2	-2.049037554	0.001553898
TRPV6	-2.090106792	1.53E-09
CD177	-2.10116449	2.20E-05
PSAPL1	-2.151134651	4.92E-08
UNC13A	-2.192217879	4.25E-06
HOXC12	-2.204734179	0.001609903
INSM1	-2.214543691	0.00917142
VNN1	-2.217818303	9.63E-11
SLC26A9	-2.220189331	0.000254569
PON1	-2.309857806	0.005206827
CYP1A1	-2.317765021	0.008365202
MUC6	-2.343051911	0.00016574
SERPINB7	-2.348059457	0.000192101
SPRR3	-2.354492111	0.000139586
SERPINB2	-2.382168138	0.000138681
TP63	-2.419108822	0.00273933
KRT16	-2.462021221	3.90E-05
TM4SF4	-2.479026997	1.20E-06
SCEL	-2.524552145	1.04E-05
PAX4	-2.672545064	0.00962652
SEMG1	-2.684363145	1.48E-07
AQP5	-2.809972913	8.50E-06
GP2	-2.93765475	0.003522772
MSMB	-2.968053657	0.004525496
HOXC10	-2.996648615	1.81E-05
SPRR1B	-3.144864532	1.69E-06
CLDN18	-3.349002749	1.52E-10
GPR87	-3.477130334	1.26E-05
SEMG2	-4.282387504	8.61E-06
