# Supplementary material for: Alteration in the Immune Microenvironment Based on APC Status in MSS/pMMR Colon Cancer
Source: Dis Markers. 2022 Jul 27;2022:3592990. doi: 10.1155/2022/3592990 (PMC9348928; doi:10.1155/2022/3592990)
Supplement: Supplementary Materials — Supplement Table S1: all different expression genes (DEGs) listed between APC-wt and APC-mt MSS/pMMR colon cancer from the overall gene expression profile of all 338 colon cancer cases obtained from TCGA database. Supplementary Table S2: APC-mt vs. APC-wt MSS/pMMR colon cancer, 379 genes were downregulated, and 117 genes were upregulated. Supplementary Table S3: APC-wt MSS/pMMR (77 samples) COAD samples in the cohort retrieved from TCGA performed gene set enrichment analysis enriched (GSEA) in 115 KEGG pathways. Supplementary Table S4: APC-wt MSS/pMMR COAD samples in the cohort retrieved from TCGA performed gene set enrichment analysis downregulated (GSEA) in 62 KEGG pathways. Supplementary Table S5: 65 overlapping genes among the DEGs (496 genes related to APC status) and immunophenotypes (1297 genes shown in Supplementary Table S6). Supplementary Table S6: 1297 immune-related genes. Supplementary Table S7: Lasso and Cox regression analyze APC-related DEGs constructing immune scoring model. Supplement Figure 1: the expression of CTLA4, PD-L1, and immune score and stromal score in the KRAS-mt/MSS group was downregulated compared with KRAS-wt/MSS. Supplement Figure 2: the expression of not only PD-1, PD-L1, and CTLA4 but also immune score, ESTIMATE score, and stromal score did not differ between TP53-wt/MSS and TP53-mt/MSS groups. Supplement Figure 3: in the combination of different genotypes of KRAS, TP53 and APC, the TMB and immune score are significantly higher in wild type than KRAS/TP53/APC mutant type colon cancer. Supplement Figure 4: neither mutations in KRAS nor TP53 could affect the proportion of infiltrating immune cell types in MSS/pMMR colon cancer. [file 3592990.f1.zip › Supplement table S2.pdf]

APC-mt/MSS vs.APC-wt/MSS DEGs(379 genes were down-regulated and 117 genes were up-regulated (fold change > 1.5, P < 0.05)

gene	conMean	treatMean	logFC	pValue	fdr
CBLN1	0.4390761	0.9559647	1.1224865	2.29E-06	3.54E-05
LYZ	584.30081	288.83029	-1.016489	8.61E-06	9.83E-05
ABCB11	0.0572556	0.0274769	-1.059198	8.57E-05	0.0006097
F5	3.2725695	0.8391437	-1.963434	8.02E-05	0.0005777
CAMK2N2	0.8597211	0.2696911	-1.672561	1.12E-05	0.0001206
SLC6A4	0.3517862	0.7528828	1.0977266	1.13E-06	2.04E-05
JAKMIP1	0.4145564	0.1749528	-1.244602	2.42E-09	2.89E-07
PIWIL1	3.132868	1.1147377	-1.49078	0.0215765	0.0480274
PTPRC	6.7259224	3.1409138	-1.098548	6.63E-08	2.52E-06
MSMB	1.864055	0.3153311	-2.563505	0.0020385	0.0075313
TLR8	1.0051405	0.4805062	-1.06477	4.90E-07	1.07E-05
IQSEC3	0.2433163	0.1172169	-1.053653	5.23E-05	0.0004106
LRRC36	0.4273781	1.0891036	1.3495561	3.85E-12	5.87E-09
CD84	1.8420202	0.8548264	-1.107585	1.15E-07	3.74E-06
CCL21	30.258533	14.973964	-1.014886	0.000263	0.0014807
SCEL	2.93802	0.4601795	-2.674576	0.0002476	0.0014123
RARRES3	56.772029	23.340222	-1.282362	4.65E-09	4.20E-07
ZNF683	1.1727185	0.464674	-1.335566	2.08E-07	5.60E-06
CDHR1	7.010103	15.07607	1.1047528	2.08E-09	2.67E-07
PIK3R5	1.305336	0.647974	-1.010413	1.35E-08	8.33E-07
CD8A	4.0402588	1.8089788	-1.159272	3.41E-09	3.39E-07
ELOVL3	0.3880822	0.1460907	-1.409498	4.49E-07	1.01E-05
MMP25	1.4907544	0.7254612	-1.039072	5.75E-06	7.18E-05
HOXC8	0.3787435	0.1601114	-1.242145	5.47E-05	0.0004255
RAB3C	0.1723381	0.0609937	-1.49851	0.0031122	0.0104693
KCNK3	0.2795439	0.1183206	-1.240374	0.0144312	0.0350315
HCAR3	0.8797708	0.4229228	-1.056733	0.0058507	0.0171509
PRDM8	1.7795142	0.5867037	-1.600779	1.36E-12	3.10E-09
CXCL10	46.730721	23.093668	-1.016874	1.01E-06	1.89E-05
TUBB4A	0.2707053	0.0863887	-1.647809	0.0001677	0.0010381
GSDMC	0.2778741	0.1241856	-1.161933	2.73E-06	4.01E-05
CD177	12.353637	3.1835841	-1.956212	2.75E-05	0.0002433
HTR2B	0.8113287	0.2925948	-1.471382	0.0026677	0.009293
EMX1	0.4474101	0.1815268	-1.301415	0.0007962	0.0036136
XIRP1	0.2054705	0.0959989	-1.097841	1.30E-05	0.0001359
KCND3	1.129915	0.5621655	-1.007147	4.79E-07	1.05E-05
VNN2	2.4933224	0.8380003	-1.573047	3.20E-11	1.83E-08
FCRL1	0.2608293	0.1176941	-1.148063	0.0031896	0.0106773
CPN1	0.1483058	0.4910009	1.7271509	7.67E-06	9.01E-05
DUSP4	14.268759	6.2442635	-1.192257	2.10E-09	2.67E-07
KRT23	13.044047	29.191764	1.1621699	1.58E-05	0.0001571
HRASLS	0.5139923	0.1443001	-1.832675	0.0001066	0.0007186
GLYATL2	0.0612335	0.2655274	2.1164675	0.000442	0.0022547
COL2A1	0.169523	0.7082831	2.0628449	2.52E-05	0.0002287
GPR174	0.6602216	0.2652498	-1.315599	3.26E-05	0.0002807

ROR1	0.7293336	0.3567871	-1.031515	8.62E-07	1.69E-05
ULBP2	3.2236011	1.384457	-1.219353	9.47E-11	3.94E-08
MEOX2	1.0110336	0.3884101	-1.380178	0.0013529	0.0054615
MAGEB17	0.5416015	2.1023239	1.9566813	1.96E-09	2.61E-07
CHST6	0.8793243	0.3161036	-1.475998	2.61E-08	1.33E-06
DRGX	0.4638091	0.154737	-1.583713	0.0013903	0.0055826
NKX3-1	0.7188038	0.3227574	-1.155148	2.37E-05	0.0002179
ZP2	0.0602453	0.1645856	1.4499183	2.10E-06	3.30E-05
SLC38A3	0.1567317	0.3321146	1.0833843	0.0011824	0.0049132
IZUMO2	0.1484569	0.3687753	1.3126974	5.50E-06	6.96E-05
ABCA3	3.1717006	1.341382	-1.241536	3.44E-06	4.85E-05
CADM3	0.6542744	0.2234698	-1.549816	0.0077901	0.021394
IL1RN	11.855689	5.3280391	-1.153903	6.31E-08	2.46E-06
BEX2	2.1464769	4.405267	1.0372588	6.98E-05	0.0005174
THBS4	4.0866056	1.9189334	-1.090598	0.0158507	0.0377038
ACKR4	0.6146553	0.2713714	-1.179509	6.47E-08	2.50E-06
TNFAIP6	5.4755379	2.6870889	-1.026957	8.50E-06	9.75E-05
TRPV6	0.4230044	0.09327	-2.181188	6.74E-08	2.55E-06
PRSS41	0.2735333	0.5535484	1.0169927	0.0078782	0.0215998
KIAA1549L	0.7115795	0.3189061	-1.157893	5.07E-05	0.0004003
SLC39A2	1.544772	3.8796089	1.3285173	1.00E-06	1.88E-05
BEAN1	0.3385535	0.1662076	-1.026397	0.0029721	0.0100823
FCGR3B	2.872245	0.9209939	-1.640915	1.00E-05	0.0001105
S100A9	118.41798	53.268981	-1.152521	0.0159624	0.0378645
IGSF23	0.3411629	0.6896246	1.0153505	9.80E-09	6.71E-07
PTH1R	0.3406145	0.1638319	-1.055923	0.0046211	0.0143142
LY6G6F-LY(	2.9107129	9.5852793	1.7194479	6.49E-13	2.23E-09
PDCD1	1.9806234	0.7999369	-1.307996	5.47E-10	1.24E-07
IKZF1	1.605324	0.7872139	-1.028037	4.31E-08	1.88E-06
KCNJ15	0.3564106	0.169201	-1.074802	6.54E-08	2.51E-06
CCL4L2	5.2037091	2.4424116	-1.091234	6.00E-05	0.0004615
GRIK5	0.3765481	0.1722343	-1.128462	0.0067406	0.0190988
CA8	3.4445751	1.6452791	-1.065994	6.15E-06	7.52E-05
LHB	0.314683	0.1524984	-1.045105	5.30E-05	0.0004146
ABCD2	0.1447223	0.0723238	-1.000746	1.44E-06	2.48E-05
UBASH3B	1.4907115	0.6765234	-1.139789	1.59E-13	1.09E-09
GPR18	0.8000833	0.315312	-1.34337	0.0002948	0.0016233
ADAM11	0.1648228	0.0788117	-1.064434	0.003684	0.012016
CARTPT	0.5124068	0.199155	-1.363398	0.0162071	0.0383454
SAA2	4.0759089	1.3372764	-1.607824	0.0003052	0.0016697
LPO	0.0806888	0.1655075	1.0364561	0.0198668	0.0449656
LRRK2	0.4619879	0.226365	-1.029204	1.26E-05	0.0001324
1-Dec	0.0461214	0.1278958	1.4714593	2.28E-08	1.20E-06
NFATC1	1.5206556	0.638012	-1.253038	1.88E-09	2.53E-07
B3GALT5	3.6133794	1.619328	-1.157953	1.51E-06	2.56E-05
CCR2	0.9920603	0.4896634	-1.018637	3.25E-06	4.63E-05
VNN1	10.933705	2.589117	-2.07825	7.94E-09	5.99E-07
SCRG1	0.1415242	0.0640447	-1.143898	0.0008298	0.0037329
FFAR2	2.2564598	0.847013	-1.413605	9.71E-05	0.0006689
FYB1	3.1329221	1.5632986	-1.002916	1.82E-07	5.07E-06
CCDC175	0.0717447	0.1502035	1.0659738	1.68E-05	0.0001658

CCDC196	0.2737944	0.549412	1.0047954	0.0044011	0.0137967
FCRL3	0.3293594	0.1525372	-1.110502	3.66E-05	0.0003072
ANO1	11.639026	5.7948842	-1.006119	9.82E-09	6.71E-07
PDZRN4	0.3397058	0.1676212	-1.019081	0.0042443	0.013407
HLA-DMB	11.462374	5.4386594	-1.075583	2.52E-09	2.95E-07
CLDN18	30.330794	2.281851	-3.732507	1.98E-09	2.62E-07
HLA-DRB5	126.68054	57.335147	-1.143703	2.99E-05	0.0002611
IL18RAP	0.5559679	0.2737249	-1.022275	3.91E-06	5.31E-05
CCL5	24.904906	11.423502	-1.124425	1.52E-09	2.15E-07
KRT7	8.2214677	3.3542177	-1.29342	2.91E-07	7.21E-06
DPP10	0.0697298	0.2301636	1.7228128	0.0151042	0.0363183
FPR2	1.1804539	0.4125632	-1.516655	6.28E-06	7.65E-05
TNFSF9	10.855007	5.0822493	-1.094822	2.17E-07	5.80E-06
CAPN6	2.0530408	5.1157022	1.31717	0.0108921	0.0278699
SIGLEC5	0.2086436	0.0967685	-1.108432	4.20E-08	1.87E-06
S100A8	19.820361	5.009603	-1.984215	1.55E-05	0.0001551
SCG3	0.7500228	0.1958258	-1.937364	0.0168611	0.0396124
CALML3	0.1049487	0.535959	2.3524378	6.55E-09	5.31E-07
CD163	8.2254806	3.851067	-1.094842	5.26E-07	1.14E-05
NXF3	1.4981768	0.7149327	-1.067329	7.10E-05	0.0005242
RNF148	0.1476592	0.0728101	-1.020062	3.06E-07	7.45E-06
TBX21	0.4911238	0.2139985	-1.198486	1.74E-09	2.36E-07
CCR5	2.6514606	1.3209171	-1.005247	9.04E-09	6.49E-07
PTAFR	5.3740144	2.6798603	-1.003842	7.79E-11	3.54E-08
SLC26A9	1.1623375	0.28587	-2.023598	0.0012024	0.0049799
RBP2	2.0379827	8.4726885	2.0556781	2.33E-09	2.82E-07
LRIT2	0.1585022	0.3452175	1.1230027	5.79E-09	4.95E-07
SLC1A7	0.5755602	1.6361984	1.507309	2.91E-05	0.0002551
RIIAD1	0.2059804	0.527204	1.3558543	1.09E-06	2.00E-05
CD96	1.2941092	0.6426459	-1.009863	1.08E-08	7.06E-07
TMEM211	2.5426555	5.2057585	1.0337724	2.57E-05	0.0002326
KRT83	0.0834163	0.2384055	1.5150168	0.0005982	0.0028716
SERPINB7	0.800253	0.156015	-2.358772	0.0007475	0.0034367
ADCYAP1	0.5236366	0.2015974	-1.377089	0.0116269	0.0293669
SHISA6	0.1529514	0.5262311	1.7826231	0.0151948	0.0364534
ITK	0.7471992	0.3543341	-1.076382	5.93E-06	7.35E-05
TRNP1	9.37372	4.1575493	-1.172888	2.87E-09	3.11E-07
FREM2	0.2081886	0.4323138	1.0541879	1.40E-05	0.0001438
SLA	2.9571743	1.3609272	-1.119629	1.22E-10	4.79E-08
DPYD	2.5842319	1.2571986	-1.039523	3.16E-09	3.28E-07
FCGR3A	21.500845	10.016706	-1.101985	8.15E-08	2.95E-06
HLA-DOA	9.8749773	3.0371417	-1.701063	2.14E-08	1.15E-06
ADGRG6	5.4068498	2.4536042	-1.139886	5.61E-10	1.24E-07
NTRK1	0.1737158	0.078732	-1.141706	7.21E-07	1.46E-05
SAA1	48.959922	14.665883	-1.739137	0.0005617	0.0027355
PRDM13	0.0898709	0.1969959	1.1322398	9.49E-05	0.0006599
VTCN1	0.2710297	0.1261657	-1.103132	0.004168	0.0131994
HLA-DMA	39.803611	17.233033	-1.207723	1.06E-08	7.02E-07
AC008687.‹	0.2025086	0.0811036	-1.320145	0.0076888	0.0212162
RNF182	0.188694	0.489977	1.3766652	0.001131	0.0047443
SAMD9	5.9338181	2.6700232	-1.152108	1.64E-09	2.26E-07

RIPPLY3	0.3169055	0.6929575	1.1287141	9.71E-07	1.84E-05
PIPOX	0.9858853	2.0304528	1.0423098	0.0049065	0.0149847
TNNC2	9.2209806	21.232533	1.2032844	4.04E-10	1.02E-07
LCK	8.9417139	4.0641303	-1.137605	2.37E-07	6.16E-06
FCGR1B	0.1767732	0.0826726	-1.096418	3.65E-07	8.56E-06
SNPH	1.7346825	0.782181	-1.149097	2.01E-08	1.11E-06
FAM3B	3.8244024	9.6679942	1.3379823	5.48E-06	6.94E-05
RERGL	0.2067739	0.0710839	-1.540459	0.0094806	0.0249942
ENPP3	2.707888	6.1431669	1.1818145	4.09E-06	5.48E-05
TRAT1	0.4960565	0.2225227	-1.156552	5.75E-06	7.18E-05
PRF1	6.4125951	3.0155486	-1.088488	5.10E-09	4.46E-07
GALR2	0.4052111	0.1661951	-1.285796	0.0010987	0.0046463
FPR1	5.5416606	2.2807732	-1.280795	2.32E-06	3.56E-05
LAG3	2.5589452	1.2301996	-1.056657	1.02E-09	1.69E-07
MYH7B	0.2459535	0.8349234	1.763258	4.98E-09	4.42E-07
APOBEC3A	0.6593324	0.273856	-1.267588	0.0024913	0.008804
DKK4	1.3674144	9.8448961	2.8479255	0.0179652	0.0416718
GRM8	1.2650089	3.1347842	1.3092186	7.39E-10	1.43E-07
CDH19	0.1669071	0.0601587	-1.472199	2.69E-05	0.0002399
PM20D1	0.1371749	0.0372533	-1.880577	0.0038219	0.0123542
HOXC6	1.3790527	0.4547338	-1.600584	1.44E-06	2.48E-05
NODAL	0.1198331	0.3133234	1.3866253	4.64E-07	1.03E-05
HTR3A	0.2402576	0.0905911	-1.407141	1.50E-06	2.55E-05
NAPSA	0.2383925	0.4877191	1.0327114	0.006238	0.018013
GRPR	0.1837924	0.5522524	1.5872505	0.0005689	0.0027659
NCF1	1.1358725	0.5418748	-1.067769	7.82E-09	5.93E-07
LRRTM2	0.1732621	0.070973	-1.287614	0.0061853	0.0179101
GZMA	13.848644	5.5775037	-1.312053	9.03E-10	1.57E-07
CNN1	28.482724	13.170552	-1.112771	0.0081292	0.0221509
GZMK	1.8740384	0.7242028	-1.371685	2.73E-09	3.07E-07
IGF2BP3	1.0301804	0.5012321	-1.039346	1.22E-06	2.18E-05
TEKT5	0.1312582	0.2668426	1.023582	1.33E-05	0.0001374
CHRNA3	0.8387575	0.3887907	-1.10926	0.0008553	0.0038238
SPERT	0.2529791	0.508616	1.0075585	0.0005361	0.002627
UNC5A	0.6639526	0.1901299	-1.804094	5.12E-06	6.57E-05
FDCSP	8.6514942	2.5536053	-1.760414	0.0220674	0.0488744
ANXA1	35.133018	16.146291	-1.121625	1.37E-07	4.16E-06
SERPINB2	1.6189293	0.2890385	-2.485706	0.0005732	0.0027799
PLA2G12B	1.6008935	5.0753133	1.6646195	1.07E-07	3.57E-06
BCL2A1	9.5366075	4.069449	-1.228643	1.37E-07	4.16E-06
HOXD1	0.5034798	0.2208792	-1.188676	8.30E-06	9.57E-05
OR2I1P	33.868843	13.407809	-1.336885	0.0002077	0.0012327
PYHIN1	0.4962091	0.2373599	-1.063872	3.65E-08	1.69E-06
KRT75	0.4949608	1.1598595	1.228564	5.63E-05	0.0004355
HAPLN3	9.1107373	4.2403792	-1.103375	2.65E-12	4.55E-09
IGFL4	0.4024867	0.8630905	1.1005708	6.41E-07	1.34E-05
WFDC10B	0.2913462	0.6222891	1.0948506	1.75E-07	4.94E-06
SH2D1B	0.2378179	0.1185083	-1.004869	8.85E-07	1.72E-05
APOC1	30.818637	14.511041	-1.086652	0.0001046	0.0007078
NGB	0.0877154	0.0217481	-2.011938	0.0088272	0.023639
GPNMB	25.514272	12.621177	-1.015458	8.74E-07	1.70E-05

APLP1	1.1990823	0.3551588	-1.755395	3.18E-05	0.0002746
BHLHA9	0.082168	0.268167	1.7064828	0.000138	0.0008876
CALCA	0.3218095	2.7554604	3.0980143	1.50E-05	0.0001514
VNN3	0.2374174	0.0988524	-1.264078	3.61E-05	0.0003039
UMODL1	0.1019368	0.2436695	1.2572509	3.95E-08	1.80E-06
AKAP12	6.1151439	2.8424479	-1.105253	0.0063851	0.0183452
HOXD3	0.1953342	0.0962087	-1.021706	1.19E-08	7.55E-07
TACR1	0.1570455	0.0700492	-1.164741	0.0027952	0.0096246
PLEK	10.643036	4.8273356	-1.140611	2.12E-07	5.71E-06
SP140	1.1184746	0.4022083	-1.475518	5.56E-10	1.24E-07
GBP1	18.288091	8.546295	-1.097533	3.16E-09	3.28E-07
FGR	4.0126828	1.9877449	-1.013435	4.28E-08	1.88E-06
CHIT1	1.3315056	0.5273886	-1.33612	0.0024598	0.0087153
GBP4	15.397754	5.5892999	-1.46198	3.44E-09	3.39E-07
NTSR1	2.2346609	0.8026116	-1.477282	2.96E-05	0.0002592
MARCO	5.2983063	2.6188407	-1.016603	0.0015092	0.0059556
ARC	0.5852013	0.2797984	-1.064545	0.0016404	0.006351
AFP	0.1199685	0.2509577	1.0647881	0.0006669	0.0031389
ANXA10	3.1229867	0.8364546	-1.900567	5.64E-08	2.28E-06
CCK	0.7381939	0.2390235	-1.626848	9.18E-06	0.0001033
MT3	0.3446951	1.3352332	1.9536989	0.0007577	0.0034663
ANGPTL1	0.8700971	0.3491697	-1.317248	0.0003096	0.0016886
CIITA	3.7757913	1.2890973	-1.550418	2.34E-09	2.82E-07
SPON1	14.731839	6.2564365	-1.235524	7.22E-08	2.68E-06
SNAP25	0.5547303	0.1513138	-1.874243	0.0013217	0.0053607
FOXD1	2.0374154	0.9067509	-1.167962	6.77E-06	8.14E-05
KRT40	0.33961	1.4964643	2.1396068	0.0001216	0.0008002
HAND2	0.9803884	0.482501	-1.022821	0.0039805	0.012762
FASLG	0.8662114	0.3794299	-1.190886	2.05E-07	5.55E-06
PRSS56	0.4633833	3.7578766	3.0196398	3.28E-06	4.66E-05
CHRDL2	6.0260816	2.5417705	-1.245386	0.0155459	0.0371138
RSPO3	2.2917649	0.7335241	-1.643543	9.79E-07	1.85E-05
MEFV	0.4445423	0.1849894	-1.264878	1.26E-05	0.0001324
PRR9	0.3643972	1.0395154	1.5123272	0.0028328	0.0097297
ZBTB16	0.1910072	0.0804747	-1.24702	9.11E-05	0.0006406
HLA-DPB1	82.141571	36.776822	-1.159316	9.67E-09	6.71E-07
HCAR2	2.2839033	1.0089553	-1.178639	0.0012635	0.0051676
KIR2DL4	0.4584896	0.1817116	-1.335239	1.18E-08	7.51E-07
CCL4	7.2248921	3.0918499	-1.224506	9.97E-09	6.76E-07
TNNT1	4.4894985	2.0456853	-1.13397	0.0001598	0.0009977
PKLR	0.6407491	1.6298219	1.3468829	1.57E-05	0.000157
ZBED2	1.1875039	0.4111688	-1.530129	3.57E-09	3.50E-07
HIST1H3J	3.7544256	1.3468539	-1.478999	0.0032447	0.0108354
CARD17	0.4713924	0.2037093	-1.210417	0.0001922	0.0011607
TEX43	0.5410625	1.1238891	1.0546325	2.63E-08	1.34E-06
STEAP4	0.8688716	0.3654224	-1.249578	6.59E-07	1.36E-05
PLCB4	12.829232	27.953289	1.1235833	7.43E-08	2.74E-06
PSAPL1	0.3123007	0.0582269	-2.423179	8.47E-07	1.66E-05
ARHGEF4	0.7923602	0.3678101	-1.107195	0.0009768	0.0042414
WNT10B	0.5976048	0.2307372	-1.372941	0.0012082	0.0049963
OMG	0.1958889	0.078097	-1.326695	0.0146004	0.0353772

PAH	0.2628538	0.7661492	1.5433646	1.41E-07	4.27E-06
PI3	277.19853	120.04586	-1.207334	0.0006974	0.0032537
HMSD	0.2303666	0.1082856	-1.08909	9.78E-06	0.0001084
PON1	0.2173537	0.0718439	-1.597107	0.016526	0.0389857
CAV2	4.5845352	2.2759139	-1.01033	2.61E-07	6.64E-06
CTXN1	5.6101662	2.6373229	-1.088969	3.91E-06	5.31E-05
CAPN14	0.3264164	0.0992771	-1.717181	0.0165378	0.0390002
AL645941.:	0.2648203	0.1179922	-1.166322	2.46E-06	3.71E-05
PRPH	0.3320884	0.1074978	-1.62726	0.0004635	0.0023372
PRDM16	0.5996013	0.2890022	-1.052923	1.51E-05	0.0001523
CASQ2	1.0573443	0.459629	-1.201904	0.010397	0.0268735
APOE	102.36476	47.895904	-1.095745	2.22E-06	3.44E-05
HLA-DQA1	18.679691	7.9989717	-1.223584	9.11E-09	6.51E-07
SERPINE1	29.161504	13.428491	-1.118768	0.0002263	0.0013191
SCG2	2.5421647	1.0634915	-1.257249	0.0009038	0.0039796
PDCD1LG2	1.5490708	0.7384967	-1.06874	7.22E-09	5.65E-07
R3HDML	0.5993288	1.6647896	1.4739204	1.26E-10	4.81E-08
CD53	23.359914	11.492379	-1.023357	1.04E-09	1.69E-07
TRIM7	2.2514431	0.8539558	-1.398617	6.77E-09	5.37E-07
CCBE1	0.2803389	0.1393504	-1.008455	2.31E-05	0.0002139
SLC4A4	2.6780378	1.1797912	-1.182645	0.0003007	0.0016531
IDO1	18.76692	5.6114277	-1.741752	4.82E-06	6.28E-05
PLA2G2D	1.9831025	0.6881051	-1.527058	7.98E-06	9.30E-05
BACH2	0.3202241	0.1491065	-1.102738	0.0004663	0.002345
HLA-DRB1	359.86515	171.8686	-1.06615	2.97E-07	7.27E-06
KLRG2	0.2655466	0.534996	1.0105628	9.86E-05	0.0006772
LRFN2	0.063059	0.1600382	1.3436411	0.0001459	0.0009269
SLC2A5	1.7271897	0.7528232	-1.198044	5.99E-09	5.01E-07
ASCL5	0.5119242	1.0885869	1.0884545	5.23E-05	0.0004106
STMND1	0.0974627	0.3020952	1.6320809	3.41E-10	9.75E-08
WIF1	0.3098785	2.6984957	3.1223807	1.47E-05	0.000149
MMP8	0.2792786	0.1318314	-1.083011	0.021546	0.0479751
TMOD1	0.7867664	0.3201554	-1.297163	2.45E-06	3.71E-05
NEUROG3	0.9457544	0.449752	-1.072336	0.0043369	0.0136459
HLA-DQB1	33.733796	16.203152	-1.05792	7.73E-06	9.05E-05
SLC13A3	0.8150687	1.7464666	1.0994455	0.0009813	0.0042581
HOMER2	0.2806807	0.1029379	-1.447156	2.16E-05	0.000202
MSR1	3.9616903	1.9758935	-1.003611	3.63E-06	5.03E-05
CD7	5.3079258	2.1967441	-1.272781	1.64E-10	5.72E-08
TCL1A	0.8788448	0.3678868	-1.256346	0.0006319	0.0029975
LY6G6E	0.1299629	0.3441782	1.4050552	6.00E-10	1.27E-07
CD274	2.0706156	0.9899809	-1.064587	4.27E-09	4.04E-07
SEMG1	2.360821	0.4507071	-2.389026	3.58E-06	5.00E-05
SLAMF9	0.9869282	0.4791753	-1.042392	0.0147685	0.0356485
CPNE6	0.068988	0.1409034	1.0302884	0.0012158	0.0050263
TM4SF4	10.52312	2.3144695	-2.184809	6.83E-05	0.0005089
MYOC	0.3163878	0.0641116	-2.303036	0.0221103	0.0489616
ZNF831	0.1910177	0.0806494	-1.243971	8.62E-07	1.69E-05
HOXC9	0.5806082	0.2158382	-1.427615	6.47E-06	7.83E-05
RTBDN	0.2481602	0.0926118	-1.422004	3.37E-05	0.0002878
PRKCB	1.0878667	0.4964007	-1.131925	3.84E-08	1.76E-06

TNMD	0.396789	0.9388482	1.2425201	9.22E-07	1.78E-05
ATP1A3	1.7096097	0.6260704	-1.44927	0.0057242	0.0168703
RSPO1	0.148691	0.0740948	-1.004872	0.0094635	0.0249782
MMP1	107.90289	50.062139	-1.107942	0.0031503	0.0105665
MEIS2	0.8429551	0.3826035	-1.139606	4.98E-08	2.08E-06
FREM1	1.2206606	2.525835	1.0490983	0.0012979	0.0052812
XCL2	0.6564124	0.3247041	-1.015477	1.50E-08	8.91E-07
GIMAP5	0.2472286	0.1125898	-1.134769	2.79E-06	4.07E-05
ARX	0.4807994	0.1776329	-1.436536	0.0010082	0.0043444
MUCL3	0.2074294	0.0805529	-1.364611	1.85E-06	2.97E-05
CXCL13	10.195222	3.6820718	-1.469304	1.70E-08	9.90E-07
ZIC5	1.1643943	0.5646484	-1.044155	0.0013932	0.0055897
SLFN12L	0.2674413	0.1151216	-1.216064	9.63E-06	0.0001074
ADGRG3	1.1162625	0.501803	-1.153483	2.96E-06	4.28E-05
IGLON5	0.4502855	0.1464437	-1.620494	0.0003605	0.0019113
GABRP	5.2339091	1.8398004	-1.50834	0.000282	0.0015676
CD22	1.0222203	0.4550743	-1.167532	0.000641	0.0030359
AIM2	3.0179032	1.3286689	-1.183565	6.34E-06	7.70E-05
CLEC4D	0.2121428	0.0755089	-1.490317	4.06E-06	5.46E-05
SULT2A1	0.2293843	0.5534833	1.2707732	0.0028204	0.0097066
SSTR2	0.2820737	0.1119415	-1.333327	4.68E-06	6.12E-05
RAB27B	3.899938	1.6692229	-1.224275	2.49E-10	7.75E-08
KCNA3	0.4136586	0.2046689	-1.015149	0.0001034	0.0007037
IFNG	0.6916691	0.2595647	-1.413988	1.35E-05	0.0001399
AC099489.	0.1867268	0.0825799	-1.177066	0.000146	0.0009269
KIF25	0.1346102	0.2819743	1.0667761	0.0004447	0.0022647
ZNF541	0.0719014	0.215419	1.583054	8.33E-07	1.65E-05
CEL	6.1567032	16.830334	1.4508339	1.13E-11	9.13E-09
NMUR2	0.9733269	0.2777732	-1.809017	0.0003607	0.0019115
TDGF1	3.2650511	7.2409345	1.1490703	3.39E-13	1.55E-09
PTPRU	5.3124129	2.223163	-1.256754	5.73E-07	1.22E-05
IL17REL	0.1812225	0.0751239	-1.270418	0.0002591	0.0014629
SIX4	0.6063141	0.2276247	-1.413408	0.0004822	0.0024154
RAB39B	0.2598536	0.1097459	-1.243532	1.05E-05	0.0001144
NSG2	0.2408088	0.0695703	-1.791344	0.010795	0.0276989
SOX1	0.3976521	0.9874084	1.3121402	7.30E-05	0.0005349
CLEC4E	1.4467728	0.5976555	-1.275452	1.37E-07	4.16E-06
GNG8	0.8402422	0.301789	-1.477265	0.0057146	0.0168674
CCL19	9.517615	3.9882032	-1.254861	0.002065	0.0076005
TENM3	0.6527118	0.3206083	-1.025634	1.07E-05	0.0001162
TMEM249	0.2022792	0.4370914	1.1115873	0.0041953	0.0132733
MS4A1	1.303945	0.5627337	-1.212359	0.0120801	0.030268
BATF3	1.2830152	0.6078293	-1.0778	6.52E-09	5.31E-07
MUC6	3.97135	1.3058817	-1.604605	0.0078559	0.0215444
FSTL4	0.0893846	0.2029563	1.183071	0.000105	0.0007102
SECTM1	16.047419	6.9066338	-1.216287	2.21E-10	7.23E-08
RXFP4	1.2410431	2.5641167	1.0469087	6.03E-10	1.27E-07
AQP5	10.760398	2.052044	-2.390598	0.0003379	0.0018142
NPFFR1	0.2566369	0.531682	1.0508352	1.71E-08	9.90E-07
SMTNL1	0.4827528	0.1649828	-1.548969	1.46E-05	0.0001479
CEACAM18	0.8346751	0.4100265	-1.025498	0.008611	0.0231965

MUC5AC	7.3239791	2.6122182	-1.487352	0.0195837	0.0444791
COLGALT2	1.1294608	0.3492195	-1.693428	0.0010434	0.004468
ITLN2	0.1755083	0.9129086	2.3789314	0.0128358	0.0318112
SLC19A3	0.9808584	2.2372537	1.1896121	5.55E-08	2.25E-06
TCEAL2	0.6949904	0.1829368	-1.925648	0.0157374	0.0374723
KISS1R	0.4433929	0.1720787	-1.365517	0.0034373	0.0113678
MSH4	0.2406262	0.0915539	-1.3941	0.0001891	0.0011466
SMIM18	0.1590868	0.0392635	-2.018554	0.021648	0.0481325
GPR171	1.0900403	0.5392914	-1.015245	3.00E-08	1.47E-06
APOL3	6.2031876	3.015251	-1.040732	3.63E-09	3.53E-07
FSCN1	35.365227	15.905779	-1.15278	1.81E-08	1.02E-06
XPNPEP2	2.5241506	5.1810303	1.0374411	0.0021144	0.007753
CD109	3.6943533	1.3876964	-1.41263	8.21E-08	2.95E-06
TMEM252	0.6691797	2.791636	2.0606452	2.94E-05	0.0002573
MAP7D2	1.3226915	3.051313	1.2059536	1.07E-05	0.0001162
KYNU	0.5303632	0.2576158	-1.04176	5.12E-08	2.12E-06
TPO	0.2081367	0.0703788	-1.564319	3.16E-11	1.83E-08
IFITM10	1.5225752	0.748187	-1.025043	4.84E-10	1.17E-07
CSF3R	2.4155425	0.9842771	-1.295211	2.70E-06	3.98E-05
NKX2-1	0.2030353	0.9002365	2.1485733	0.0002921	0.0016134
SCGN	0.6836287	0.3082989	-1.148883	0.0113231	0.028769
APCDD1	7.2087115	18.992063	1.3975833	9.94E-08	3.37E-06
CCL18	35.601011	13.082302	-1.444302	2.37E-07	6.16E-06
GPR87	0.4276651	0.0348452	-3.617451	6.26E-05	0.0004763
CEND1	0.298812	0.1457711	-1.035534	0.0001389	0.0008923
SPACA3	0.2721693	1.0689391	1.9736036	8.24E-09	6.18E-07
ACSL6	0.9197702	2.2488972	1.2898723	1.12E-08	7.26E-07
PCDHB8	0.5350099	0.2363955	-1.178363	0.0018638	0.0070219
IL22RA2	0.174068	0.0805766	-1.111218	0.0001913	0.0011572
HLA-DQB2	6.6163357	3.2001325	-1.047901	9.57E-07	1.83E-05
CHI3L2	0.8572749	0.3423683	-1.324209	2.70E-05	0.0002405
FER1L6	1.5709584	0.7006432	-1.164893	9.76E-05	0.0006718
NEUROG2	0.0709213	0.2649702	1.9015401	1.60E-06	2.68E-05
TENT5B	1.0561545	0.4896282	-1.109062	4.19E-06	5.60E-05
SPDYC	0.5171977	1.0781209	1.0597311	0.0007339	0.0033892
LONRF2	0.0866604	0.0390861	-1.148716	0.0063444	0.0182628
SELL	4.1996583	2.00957	-1.063385	1.88E-05	0.0001813
KRT16	6.3749417	1.0780821	-2.563945	0.001016	0.0043684
PRAP1	38.103047	76.985673	1.0146836	6.35E-08	2.46E-06
HLA-DRA	645.00123	299.06134	-1.10886	2.68E-09	3.06E-07
XKR9	1.3506729	0.624111	-1.113804	0.0001652	0.0010264
DOCK2	1.7394746	0.8593035	-1.017412	1.37E-07	4.16E-06
MAP2	0.3050985	0.1198012	-1.348632	0.0029598	0.0100602
EOMES	0.5366722	0.2639276	-1.023899	2.41E-08	1.25E-06
DGKB	0.3184103	0.0818683	-1.959509	0.017635	0.0410447
HLA-DPA1	54.200029	22.60718	-1.261513	4.47E-09	4.12E-07
SEMA3D	0.3790558	0.115009	-1.720663	0.0026798	0.0093233
GPM6A	0.2523512	0.1079092	-1.225615	0.0120708	0.0302596
AHNAK2	3.6403671	1.6332607	-1.156329	0.0002876	0.0015925
TYMP	35.57795	16.622525	-1.097844	8.68E-10	1.57E-07
TP63	0.3118171	0.052707	-2.564633	0.0103218	0.026706

SPHK1	5.9048058	2.9265263	-1.0127	2.28E-08	1.20E-06
OIT3	0.3808922	0.8467012	1.1524701	0.0003352	0.0018025
SERPINA7	0.1477433	0.439346	1.5722653	5.29E-08	2.17E-06
SOX8	1.6188351	0.5961984	-1.441092	3.66E-06	5.05E-05
DPEP1	46.623334	116.36345	1.3195139	6.52E-09	5.31E-07
LDHC	0.0675359	0.2159315	1.6768471	0.0140582	0.0342775
LY6G6D	6.5249197	20.017592	1.6172364	7.79E-12	7.13E-09
ADCYAP1R1	0.0891574	0.0341401	-1.384888	0.0027417	0.0094902
CAMKV	0.1701204	0.4292268	1.3351837	7.16E-06	8.53E-05
WNT7B	1.037742	0.3455207	-1.586604	0.0007376	0.0034027
SIRPB1	0.468675	0.1968308	-1.251632	8.94E-08	3.15E-06
FAM83A	1.0587235	0.3022478	-1.808522	0.0119097	0.0299412
KLRD1	0.3351666	0.1473461	-1.185669	8.70E-09	6.35E-07
SAA2-SAA4	1.7742982	0.5503093	-1.688934	0.0064996	0.0186042
AQP9	4.3074499	1.5057124	-1.516388	1.27E-06	2.25E-05
PNMA2	1.5980324	0.7437205	-1.103464	0.0004476	0.0022744
FGF20	0.2199096	0.5584214	1.3444436	0.0006308	0.0029975
INA	0.3312813	0.1416963	-1.225254	0.0127301	0.0315835
RPS6KA6	0.4280893	1.0000844	1.2241382	2.84E-09	3.11E-07
DNAH2	0.634222	0.3000922	-1.079582	0.0008632	0.0038529
WFDC10A	0.0871795	0.2060789	1.2411364	4.65E-06	6.11E-05
GNLY	4.8656137	1.964786	-1.30825	8.85E-07	1.72E-05
SERPINB5	32.657472	15.864888	-1.041576	1.48E-05	0.0001492
SLC1A3	1.3985788	0.6365772	-1.135554	4.22E-08	1.87E-06
CR1	0.3237253	0.1464245	-1.144613	3.44E-06	4.85E-05
NOTUM	8.9627082	32.702775	1.8674064	1.75E-06	2.85E-05
FMO1	0.4752427	0.2092187	-1.183652	5.12E-06	6.57E-05
C3	47.441504	22.952273	-1.047513	0.0022885	0.0082505
ABCC2	0.6684602	1.3868703	1.0529193	0.0086352	0.0232162
DCSTAMP	0.3111353	0.1526641	-1.027181	0.0138845	0.0339628
ITGAL	3.6387094	1.7358428	-1.067791	9.82E-09	6.71E-07
CTSE	48.718932	19.009835	-1.357737	7.73E-06	9.05E-05
DHRS9	7.6150078	3.5626544	-1.095893	0.0032971	0.0109782
OCA2	0.3007915	0.6256969	1.0567003	1.74E-07	4.93E-06
SPRR3	5.7240273	1.0807734	-2.404966	0.0069357	0.0195427
LY6D	2.8899867	0.7049684	-2.035432	0.0038927	0.0125476
CCR7	2.1433221	1.0618499	-1.013269	0.0040953	0.0130351
DRD2	0.4825354	1.1067286	1.1975949	5.61E-07	1.20E-05
SRRM3	0.7033346	0.2647954	-1.409333	0.0010942	0.0046339
HOXC4	0.7608272	0.3121945	-1.285124	2.53E-08	1.30E-06
GBP5	4.471447	1.3287729	-1.750647	9.26E-10	1.59E-07
TIGIT	1.1316563	0.5534502	-1.03191	5.22E-09	4.53E-07
SEMA3E	0.1789324	0.0395213	-2.178714	0.0067845	0.0191796
VAX2	0.3469257	0.1601741	-1.114986	0.0005323	0.0026113
OSR1	0.4894471	0.193247	-1.340706	5.63E-12	6.44E-09
FCRL6	0.3682621	0.1498343	-1.297365	1.82E-08	1.02E-06
MAOB	5.4014681	2.2723741	-1.249151	1.24E-05	0.0001308
ADRB1	0.8086289	0.2906646	-1.476122	3.72E-06	5.13E-05
CXCR1	1.0552745	0.473518	-1.156127	0.0060219	0.0175548
GLT1D1	0.2514668	0.111193	-1.177302	8.17E-07	1.62E-05
MRC1	5.704344	2.7814169	-1.036241	1.29E-05	0.000135

NKD1	4.5118278	13.511729	1.5824284	4.08E-10	1.02E-07
SPRR1B	12.658694	1.3475244	-3.231745	0.0001275	0.0008339
SAMD9L	5.4305558	2.3352863	-1.2175	1.44E-10	5.36E-08
EPHB6	4.3250916	1.3839789	-1.643909	0.0007445	0.0034242
RARRES1	22.69903	10.68084	-1.087606	5.13E-10	1.21E-07
PTPRO	4.3035383	10.911516	1.3422563	2.98E-10	8.71E-08
LRRC2	0.5603881	1.2021217	1.1010848	1.03E-11	8.79E-09
RASGRP1	0.9783198	0.4804302	-1.025979	2.92E-09	3.11E-07
FCAR	0.4639023	0.199037	-1.220785	0.0006143	0.0029353
SLC30A2	0.5619587	1.2886509	1.1973254	3.41E-08	1.62E-06
SIGLEC14	1.5292036	0.7335016	-1.059909	5.64E-06	7.07E-05
HMX3	0.5883447	0.1585862	-1.891395	7.36E-05	0.0005387
HDAC9	0.4884548	0.239754	-1.02667	0.009238	0.0244818
SORCS1	0.1178132	0.0515559	-1.192293	0.014721	0.0355651
FCGR2B	0.9844121	0.4714226	-1.062242	2.92E-09	3.11E-07
CXCL9	27.297009	12.329792	-1.146594	6.72E-07	1.39E-05
DSCAML1	0.1784429	0.0680947	-1.389848	0.0087562	0.0234953
POU5F1B	0.9597934	1.9628933	1.032186	1.22E-07	3.89E-06
UNC13A	0.5789163	0.1150228	-2.331435	2.07E-05	0.0001956
HTRA4	0.430862	0.1850132	-1.219598	3.03E-07	7.41E-06
CRTAC1	0.315453	0.1462996	-1.1085	0.0092539	0.0245193
C2CD4C	0.6114797	0.2833408	-1.109766	1.64E-06	2.70E-05
IKZF3	1.7419579	0.8320826	-1.065911	2.83E-09	3.11E-07
CTSW	3.3289013	1.4486857	-1.200301	3.89E-11	2.13E-08
ANGPTL7	0.1717003	0.0587239	-1.547872	0.0025687	0.0090259
GZMH	4.0858118	1.6975054	-1.267207	7.92E-11	3.54E-08
TNFAIP2	17.689371	7.9103349	-1.161072	7.99E-11	3.54E-08
GRAMD1B	1.4592692	0.5462814	-1.41753	8.40E-07	1.66E-05
PLP1	0.2335191	0.1106128	-1.078022	0.0050888	0.0153978
SPATC1	0.1907917	0.0844507	-1.175817	7.30E-07	1.47E-05
HOXC10	0.9727591	0.1117639	-3.121629	1.94E-06	3.09E-05
CNR1	0.2253584	0.0766402	-1.556048	0.0134856	0.0331881
CRTAM	0.4875029	0.192085	-1.343666	2.02E-09	2.63E-07
CD74	659.09342	322.96486	-1.029106	1.37E-08	8.42E-07
SYT5	0.2131153	0.0733886	-1.538005	8.01E-06	9.31E-05
NKG7	11.792152	5.0523859	-1.22279	8.34E-11	3.58E-08
RAMP1	28.460735	13.332577	-1.094017	1.41E-05	0.0001444
SMPX	0.2790122	0.8219623	1.5587441	0.0160623	0.0380751
KCNV1	0.1097246	0.2601644	1.2455367	0.0018465	0.0069702
LY6G6F	0.0921588	0.353545	1.9396997	2.68E-11	1.75E-08
GAS1	4.6090224	2.2288043	-1.048191	1.53E-05	0.0001539
ONECUT2	0.6988229	0.2795758	-1.321687	0.0107531	0.027612
MMRN1	1.3018613	0.5069647	-1.360619	0.0004938	0.0024568
