# Supplementary material for: Alteration in the Immune Microenvironment Based on APC Status in MSS/pMMR Colon Cancer
Source: Dis Markers. 2022 Jul 27;2022:3592990. doi: 10.1155/2022/3592990 (PMC9348928; doi:10.1155/2022/3592990)
Supplement: Supplementary Materials — Supplement Table S1: all different expression genes (DEGs) listed between APC-wt and APC-mt MSS/pMMR colon cancer from the overall gene expression profile of all 338 colon cancer cases obtained from TCGA database. Supplementary Table S2: APC-mt vs. APC-wt MSS/pMMR colon cancer, 379 genes were downregulated, and 117 genes were upregulated. Supplementary Table S3: APC-wt MSS/pMMR (77 samples) COAD samples in the cohort retrieved from TCGA performed gene set enrichment analysis enriched (GSEA) in 115 KEGG pathways. Supplementary Table S4: APC-wt MSS/pMMR COAD samples in the cohort retrieved from TCGA performed gene set enrichment analysis downregulated (GSEA) in 62 KEGG pathways. Supplementary Table S5: 65 overlapping genes among the DEGs (496 genes related to APC status) and immunophenotypes (1297 genes shown in Supplementary Table S6). Supplementary Table S6: 1297 immune-related genes. Supplementary Table S7: Lasso and Cox regression analyze APC-related DEGs constructing immune scoring model. Supplement Figure 1: the expression of CTLA4, PD-L1, and immune score and stromal score in the KRAS-mt/MSS group was downregulated compared with KRAS-wt/MSS. Supplement Figure 2: the expression of not only PD-1, PD-L1, and CTLA4 but also immune score, ESTIMATE score, and stromal score did not differ between TP53-wt/MSS and TP53-mt/MSS groups. Supplement Figure 3: in the combination of different genotypes of KRAS, TP53 and APC, the TMB and immune score are significantly higher in wild type than KRAS/TP53/APC mutant type colon cancer. Supplement Figure 4: neither mutations in KRAS nor TP53 could affect the proportion of infiltrating immune cell types in MSS/pMMR colon cancer. [file 3592990.f1.zip › Supplement table S3.pdf]

APC-wt colon cancer was significantly enriched in 115 KEGG pathways

| NAME | GS<br> fo | GS DETAIL | SIZE | ES | NES | NOM p-val | FDR q-val | FWER p-va |
|---|---|---|---|---|---|---|---|---|
| KEGG_FOCAI | KEGG_FOCAI | Details .. | 197 | 0.7570188 | 2.5582616 | 0 | 0 | 0 |
| KEGG_ECM_I | KEGG_ECM_I | Details .. | 84 | 0.8450392 | 2.4824934 | 0 | 0 | 0 |
| KEGG_TGF_I | KEGG_TGF_I | Details .. | 85 | 0.699227 | 2.396502 | 0 | 0 | 0 |
| KEGG_HEDGI | KEGG_HEDGI | Details .. | 56 | 0.7310603 | 2.3887923 | 0 | 0 | 0 |
| KEGG_PATH\ | KEGG_PATH\ | Details .. | 321 | 0.6324012 | 2.3709183 | 0 | 0 | 0 |
| KEGG_CYTOI | KEGG_CYTOI | Details .. | 257 | 0.6428217 | 2.3637373 | 0 | 0 | 0 |
| KEGG_DILA' | KEGG_DILA' | Details .. | 90 | 0.7160319 | 2.360433 | 0 | 0 | 0 |
| KEGG_HYPEI | KEGG_HYPEI | Details .. | 83 | 0.7112744 | 2.3550224 | 0 | 0 | 0 |
| KEGG_BASAI | KEGG_BASAI | Details .. | 55 | 0.7120349 | 2.3016324 | 0 | 1.45E-04 | 0.001 |
| KEGG_REGUI | KEGG_REGUI | Details .. | 211 | 0.6093289 | 2.3015594 | 0 | 1.31E-04 | 0.001 |
| KEGG_AXON | KEGG_AXON | Details .. | 127 | 0.6478015 | 2.280815 | 0 | 2.38E-04 | 0.001 |
| KEGG_CELL | KEGG_CELL | Details .. | 128 | 0.7177891 | 2.2629921 | 0 | 2.18E-04 | 0.001 |
| KEGG_MELAI | KEGG_MELAI | Details .. | 71 | 0.6393313 | 2.2466903 | 0 | 2.83E-04 | 0.002 |
| KEGG_RENAI | KEGG_RENAI | Details .. | 66 | 0.6867642 | 2.2287743 | 0 | 2.63E-04 | 0.002 |
| KEGG_GAP_ | KEGG_GAP_ | Details .. | 88 | 0.6145355 | 2.22563 | 0 | 2.45E-04 | 0.002 |
| KEGG_MAPK | KEGG_MAPK | Details .. | 265 | 0.5767839 | 2.217498 | 0 | 2.74E-04 | 0.003 |
| KEGG_MELAI | KEGG_MELAI | Details .. | 101 | 0.6050672 | 2.2054012 | 0 | 2.58E-04 | 0.003 |
| KEGG_ARRH' | KEGG_ARRH' | Details .. | 74 | 0.6833643 | 2.1969879 | 0 | 3.47E-04 | 0.005 |
| KEGG_HEMA' | KEGG_HEMA' | Details .. | 85 | 0.7132903 | 2.1830263 | 0 | 4.91E-04 | 0.007 |
| KEGG_CALC | KEGG_CALC | Details .. | 176 | 0.5808979 | 2.1566815 | 0 | 9.05E-04 | 0.011 |
| KEGG_GLYC( | KEGG_GLYC( | Details .. | 21 | 0.7722973 | 2.1509366 | 0 | 0.0010698 | 0.013 |
| KEGG_LEUK( | KEGG_LEUK( | Details .. | 115 | 0.6243677 | 2.1440198 | 0 | 0.0010212 | 0.013 |
| KEGG_JAK_ | KEGG_JAK_ | Details .. | 151 | 0.575643 | 2.1409948 | 0.002004 | 9.77E-04 | 0.013 |
| KEGG_WNT_ | KEGG_WNT_ | Details .. | 149 | 0.571755 | 2.1285226 | 0 | 0.0012179 | 0.02 |
| KEGG_GLYC( | KEGG_GLYC( | Details .. | 26 | 0.7095917 | 2.0743122 | 0 | 0.0024357 | 0.038 |
| KEGG_NEUR( | KEGG_NEUR( | Details .. | 270 | 0.5265302 | 2.060153 | 0 | 0.0028584 | 0.047 |
| KEGG_CHEM( | KEGG_CHEM( | Details .. | 184 | 0.6052579 | 2.0568697 | 0 | 0.0029295 | 0.05 |
| KEGG_SMALI | KEGG_SMALI | Details .. | 84 | 0.6172912 | 2.0532057 | 0 | 0.0029061 | 0.051 |
| KEGG_LYSO | KEGG_LYSO | Details .. | 119 | 0.6449431 | 2.04916 | 0 | 0.0029482 | 0.054 |
| KEGG_GLYC( | KEGG_GLYC( | Details .. | 22 | 0.7997208 | 2.047203 | 0 | 0.0029929 | 0.057 |
| KEGG_TOLL | KEGG_TOLL | Details .. | 101 | 0.5795874 | 2.0360425 | 0.00409 | 0.0033098 | 0.063 |
| KEGG_COMPI | KEGG_COMPI | Details .. | 69 | 0.6784996 | 2.0193384 | 0 | 0.0037971 | 0.076 |
| KEGG_PROS' | KEGG_PROS' | Details .. | 89 | 0.588153 | 2.0018654 | 0 | 0.0043582 | 0.087 |
| KEGG_NEUR( | KEGG_NEUR( | Details .. | 125 | 0.5786549 | 1.9879212 | 0 | 0.005005 | 0.104 |
| KEGG_GLIO | KEGG_GLIO | Details .. | 65 | 0.5951785 | 1.9777609 | 0 | 0.0052007 | 0.111 |
| KEGG_ADHEI | KEGG_ADHEI | Details .. | 68 | 0.6035044 | 1.9666892 | 0 | 0.0057177 | 0.122 |
| KEGG_GLYC( | KEGG_GLYC( | Details .. | 15 | 0.7473547 | 1.9592421 | 0.0077821 | 0.0061436 | 0.131 |
| KEGG_PANCI | KEGG_PANCI | Details .. | 69 | 0.5903887 | 1.9589719 | 0 | 0.0060014 | 0.132 |
| KEGG_DORS( | KEGG_DORS( | Details .. | 24 | 0.6370549 | 1.9462773 | 0.0020576 | 0.0066355 | 0.145 |
| KEGG_VASCI | KEGG_VASCI | Details .. | 113 | 0.5658289 | 1.9459258 | 0.0059406 | 0.0064955 | 0.146 |
| KEGG_ADIP( | KEGG_ADIP( | Details .. | 67 | 0.5707006 | 1.9274896 | 0.0040984 | 0.00752 | 0.16 |
| KEGG_NOD_I | KEGG_NOD_I | Details .. | 60 | 0.5972339 | 1.9268221 | 0.005988 | 0.0074036 | 0.16 |
| KEGG_CHROI | KEGG_CHROI | Details .. | 73 | 0.6028569 | 1.9063827 | 0.0020161 | 0.0084033 | 0.175 |
| KEGG_LEISI | KEGG_LEISI | Details .. | 70 | 0.6640824 | 1.9061394 | 0.0176817 | 0.0082123 | 0.175 |
| KEGG_LONG | KEGG_LONG | Details .. | 68 | 0.5212762 | 1.9045513 | 0 | 0.0082288 | 0.179 |
| KEGG_COLOI | KEGG_COLOI | Details .. | 62 | 0.5908852 | 1.8996061 | 0.002004 | 0.0084304 | 0.185 |
| KEGG_MTOR | KEGG_MTOR | Details .. | 51 | 0.5740153 | 1.8988478 | 0.0040733 | 0.0083109 | 0.185 |
| KEGG_T_CEI | KEGG_T_CEI | Details .. | 106 | 0.5907225 | 1.8970954 | 0.0119522 | 0.008289 | 0.186 |
| KEGG_ABC_' | KEGG_ABC_' | Details .. | 44 | 0.5575292 | 1.8886939 | 0.006263 | 0.0089152 | 0.198 |

| | | | | | | | | |
|---|---|---|---|---|---|---|---|---|
| KEGG_NATUI | KEGG_NATUI | Details .. | 131 | 0.5400499 | 1.8813905 | 0.0099206 | 0.009171 | 0.203 |
| KEGG_ACUTI | KEGG_ACUTE_MYELOID_ | | 57 | 0.5844306 | 1.8353963 | 0.004 | 0.0143084 | 0.258 |
| KEGG_ENDO( | KEGG_ENDOCYTOSIS | | 176 | 0.4970297 | 1.8333083 | 0 | 0.0142078 | 0.261 |
| KEGG_VIRAI | KEGG_VIRAL_MYOCARDI | | 68 | 0.5829569 | 1.8299841 | 0.0219124 | 0.0143141 | 0.266 |
| KEGG_ERBB_ | KEGG_ERBB_SIGNALING | | 86 | 0.5428832 | 1.8249168 | 0.0041068 | 0.0147894 | 0.272 |
| KEGG_APOP' | KEGG_APOPTOSIS | | 86 | 0.5223641 | 1.7724222 | 0.0232108 | 0.022413 | 0.344 |
| KEGG_ALDO: | KEGG_ALDOSTERONE_RE | | 42 | 0.5162706 | 1.7364743 | 0.0118812 | 0.0296708 | 0.395 |
| KEGG_FC_G/ | KEGG_FC_GAMMA_R_MED | | 93 | 0.5245306 | 1.733982 | 0.0142566 | 0.0296304 | 0.395 |
| KEGG_PHOSI | KEGG_PHOSPHATIDYLIN | | 76 | 0.5259326 | 1.7297846 | 0.0237624 | 0.0298874 | 0.402 |
| KEGG_BLADI | KEGG_BLADDER_CANCER | | 40 | 0.5387683 | 1.7243181 | 0.0097656 | 0.0306054 | 0.415 |
| KEGG_TYPE_ | KEGG_TYPE_II_DIABET | | 46 | 0.5178133 | 1.6815764 | 0.0221774 | 0.0400472 | 0.472 |
| KEGG_NOTCI | KEGG_NOTCH_SIGNALIN | | 47 | 0.5407462 | 1.6798022 | 0.022541 | 0.0398559 | 0.474 |
| KEGG_INSUI | KEGG_INSULIN_SIGNAL | | 137 | 0.4577613 | 1.677556 | 0.020202 | 0.0399354 | 0.482 |
| KEGG_TIGH' | KEGG_TIGHT_JUNCTION | | 128 | 0.4365727 | 1.6773155 | 0.007984 | 0.0394169 | 0.483 |
| KEGG_VEGF_ | KEGG_VEGF_SIGNALING | | 75 | 0.4790858 | 1.6763093 | 0.0158416 | 0.0390907 | 0.484 |
| KEGG_RENII | KEGG_RENIN_ANGIOTEN | | 17 | 0.6037012 | 1.6708702 | 0.019084 | 0.0401885 | 0.493 |
| KEGG_PROGI | KEGG_PROGESTERONE_M | | 85 | 0.4827653 | 1.6696353 | 0.0220884 | 0.0399486 | 0.495 |
| KEGG_GNRH_ | KEGG_GNRH_SIGNALING | | 101 | 0.4491693 | 1.6642652 | 0.0140281 | 0.0405705 | 0.501 |
| KEGG_LONG_ | KEGG_LONG_TERM_POTE | | 69 | 0.4502982 | 1.6129675 | 0.0372549 | 0.0547724 | 0.574 |
| KEGG_EPITI | KEGG_EPITHELIAL_CEL | | 67 | 0.4566164 | 1.612373 | 0.0315582 | 0.0541504 | 0.574 |
| KEGG_PRIOI | KEGG_PRION_DISEASES | | 35 | 0.5373277 | 1.6041636 | 0.0314342 | 0.0562426 | 0.586 |
| KEGG_ENDOI | KEGG_ENDOMETRIAL_CA | | 52 | 0.5222749 | 1.6035552 | 0.0408602 | 0.0555634 | 0.586 |
| KEGG_B_CEI | KEGG_B_CELL_RECEPTO | | 74 | 0.5327803 | 1.5999873 | 0.0657371 | 0.0562285 | 0.591 |
| KEGG_OTHEI | KEGG_OTHER_GLYCAN_D | | 16 | 0.6376672 | 1.5957426 | 0.0570825 | 0.0567849 | 0.598 |
| KEGG_INOS: | KEGG_INOSITOL_PHOSP | | 54 | 0.508684 | 1.5954226 | 0.0503145 | 0.0561487 | 0.599 |
| KEGG_FC_EI | KEGG_FC_EPSILON_RI_ | | 79 | 0.4389234 | 1.5518049 | 0.0475248 | 0.0705429 | 0.669 |
| KEGG_INTE: | KEGG_INTESTINAL_IMM | | 46 | 0.5691531 | 1.4997884 | 0.1411765 | 0.092031 | 0.743 |
| KEGG_VASOI | KEGG_VASOPRESSIN_RE | | 44 | 0.4354829 | 1.4715904 | 0.0510204 | 0.104516 | 0.77 |
| KEGG_RIG_ | KEGG_RIG_I_LIKE_REC | | 69 | 0.4079681 | 1.461704 | 0.0699589 | 0.1086424 | 0.785 |
| KEGG_NON_: | KEGG_NON_SMALL_CELL | | 54 | 0.442229 | 1.4081341 | 0.1078838 | 0.135588 | 0.84 |
| KEGG_ASTHI | KEGG_ASTHMA | | 28 | 0.5429037 | 1.3804035 | 0.1926229 | 0.1520242 | 0.873 |
| KEGG_GRAF' | KEGG_GRAFT_VERSUS_H | | 37 | 0.595994 | 1.3799728 | 0.212 | 0.1504891 | 0.874 |
| KEGG_PATH( | KEGG_PATHOGENIC_ESC | | 56 | 0.3852621 | 1.3242348 | 0.1340996 | 0.1885971 | 0.923 |
| KEGG_OOCY' | KEGG_OOCYTE_MEIOSIS | | 110 | 0.3570168 | 1.2931105 | 0.1992188 | 0.2098406 | 0.94 |
| KEGG_SNARI | KEGG_SNARE_INTERACT | | 38 | 0.4031101 | 1.2594641 | 0.1921488 | 0.2356426 | 0.958 |
| KEGG_REGUI | KEGG_REGULATION_OF_ | | 34 | 0.390283 | 1.2459625 | 0.1995885 | 0.245062 | 0.963 |
| KEGG_P53_: | KEGG_P53_SIGNALING_ | | 65 | 0.362116 | 1.2102549 | 0.2270916 | 0.2747865 | 0.974 |
| KEGG_SPHII | KEGG_SPHINGOLIPID_M | | 36 | 0.3762355 | 1.204333 | 0.253876 | 0.2770976 | 0.975 |
| KEGG_VIBR: | KEGG_VIBRIO_CHOLERA | | 53 | 0.3428931 | 1.1951721 | 0.2287449 | 0.282511 | 0.975 |
| KEGG_PRIM/ | KEGG_PRIMARY_BILE_A | | 16 | 0.4323643 | 1.1861137 | 0.2309237 | 0.2886117 | 0.978 |
| KEGG_NICO' | KEGG_NICOTINATE_AND | | 22 | 0.3860522 | 1.1856996 | 0.2252066 | 0.2858422 | 0.978 |
| KEGG_PPAR_ | KEGG_PPAR_SIGNALING | | 69 | 0.3515833 | 1.1762629 | 0.244898 | 0.2923736 | 0.982 |
| KEGG_ALLO( | KEGG_ALLOGRAFT_REJE | | 35 | 0.500725 | 1.1714172 | 0.3239437 | 0.2940179 | 0.982 |
| KEGG_TYPE_ | KEGG_TYPE_I_DIABETE | | 41 | 0.4499471 | 1.1703391 | 0.3307543 | 0.2921423 | 0.982 |
| KEGG_PURII | KEGG_PURINE_METABOL | | 152 | 0.2983547 | 1.1453503 | 0.2842943 | 0.3149909 | 0.989 |
| KEGG_UBIQI | KEGG_UBIQUITIN_MEDI | | 130 | 0.3402306 | 1.1387328 | 0.31643 | 0.3184483 | 0.992 |
| KEGG_THYR( | KEGG_THYROID_CANCER | | 29 | 0.3752224 | 1.1337554 | 0.3031496 | 0.320078 | 0.992 |
| KEGG_AMYO' | KEGG_AMYOTROPHIC_LA | | 52 | 0.3041801 | 1.0749251 | 0.3513514 | 0.3817663 | 0.998 |
| KEGG_TYRO: | KEGG_TYROSINE_METAB | | 40 | 0.3071046 | 1.0714182 | 0.3632734 | 0.381781 | 0.998 |
| KEGG_ARACI | KEGG_ARACHIDONIC_AC | | 58 | 0.30103 | 1.0561208 | 0.3616162 | 0.3960246 | 0.999 |
| KEGG_ANTI( | KEGG_ANTIGEN_PROCES | | 80 | 0.331894 | 1.002565 | 0.4407115 | 0.4561516 | 1 |

| | | | | | | | |
|---|---|---|---|---|---|---|---|
| KEGG_LYSII | KEGG_LYSINE_DEGRADA | 39 | 0.3300289 | 0.9602164 | 0.497992 | 0.5059297 | 1 |
| KEGG_AMIN( | KEGG_AMINO_SUGAR_AN | 44 | 0.3206492 | 0.9536998 | 0.4931238 | 0.5094618 | 1 |
| KEGG_TRYP' | KEGG_TRYPTOPHAN_MET | 39 | 0.2787183 | 0.9512767 | 0.541744 | 0.5080811 | 1 |
| KEGG_ETHEI | KEGG_ETHER_LIPID_ME | 30 | 0.2924505 | 0.949746 | 0.5463511 | 0.5052497 | 1 |
| KEGG_SYSTI | KEGG_SYSTEMIC_LUPUS | 136 | 0.2923307 | 0.9496284 | 0.5019231 | 0.5005729 | 1 |
| KEGG_GALA( | KEGG_GALACTOSE_META | 26 | 0.3073849 | 0.9362506 | 0.5374016 | 0.5128763 | 1 |
| KEGG_STER( | KEGG_STEROID_HORMON | 55 | 0.2646864 | 0.8820784 | 0.6294821 | 0.5810363 | 1 |
| KEGG_CYTOS | KEGG_CYTOSOLIC_DNA_ | 55 | 0.2558303 | 0.8738797 | 0.6313726 | 0.5865501 | 1 |
| KEGG_SELEI | KEGG_SELENOAMINO_AC | 25 | 0.300598 | 0.8562903 | 0.613497 | 0.6056925 | 1 |
| KEGG_AUTO | KEGG_AUTOIMMUNE_THY | 50 | 0.3019708 | 0.8547735 | 0.5971944 | 0.6024281 | 1 |
| KEGG_PANT( | KEGG_PANTOTHENATE_A | 16 | 0.3034285 | 0.8233019 | 0.7137097 | 0.6396189 | 1 |
| KEGG_TASTI | KEGG_TASTE_TRANSDUC | 50 | 0.2441287 | 0.7914255 | 0.7514793 | 0.6800845 | 1 |
| KEGG_O_GL' | KEGG_O_GLYCAN_BIOSY | 26 | 0.2696083 | 0.7874782 | 0.7207031 | 0.6798658 | 1 |
| KEGG_PRIM | KEGG_PRIMARY_IMMUNO | 35 | 0.318852 | 0.7718986 | 0.6647287 | 0.6962426 | 1 |
| KEGG_STAR( | KEGG_STARCH_AND_SUC | 52 | 0.2199791 | 0.7409043 | 0.8268839 | 0.7338253 | 1 |

RANK AT MAX LEADING EDGE

| | |
|---|---|
| 6654 | tags=58%, list=12%, signal=66% |
| 4486 | tags=58%, list=8%, signal=63% |
| 6092 | tags=52%, list=11%, signal=58% |
| 4160 | tags=43%, list=8%, signal=46% |
| 7173 | tags=45%, list=13%, signal=52% |
| 5340 | tags=40%, list=10%, signal=44% |
| 6790 | tags=51%, list=12%, signal=58% |
| 6790 | tags=49%, list=12%, signal=56% |
| 5849 | tags=45%, list=11%, signal=51% |
| 4706 | tags=37%, list=9%, signal=40% |
| 7159 | tags=54%, list=13%, signal=61% |
| 5339 | tags=47%, list=10%, signal=52% |
| 5340 | tags=42%, list=10%, signal=47% |
| 5340 | tags=53%, list=10%, signal=59% |
| 4998 | tags=38%, list=9%, signal=41% |
| 8823 | tags=46%, list=16%, signal=55% |
| 6670 | tags=44%, list=12%, signal=49% |
| 8377 | tags=55%, list=15%, signal=65% |
| 7342 | tags=56%, list=13%, signal=65% |
| 6356 | tags=36%, list=12%, signal=40% |
| 3336 | tags=48%, list=6%, signal=51% |
| 8049 | tags=51%, list=15%, signal=60% |
| 7304 | tags=43%, list=13%, signal=49% |
| 6670 | tags=40%, list=12%, signal=46% |
| 6136 | tags=46%, list=11%, signal=52% |
| 8070 | tags=37%, list=15%, signal=43% |
| 6453 | tags=44%, list=12%, signal=50% |
| 6831 | tags=43%, list=12%, signal=49% |
| 7871 | tags=55%, list=14%, signal=64% |
| 4916 | tags=55%, list=9%, signal=60% |
| 5766 | tags=40%, list=10%, signal=44% |
| 4830 | tags=46%, list=9%, signal=51% |
| 6045 | tags=43%, list=11%, signal=48% |
| 7393 | tags=51%, list=13%, signal=59% |
| 6453 | tags=46%, list=12%, signal=52% |
| 8533 | tags=57%, list=15%, signal=68% |
| 5827 | tags=60%, list=11%, signal=67% |
| 5050 | tags=36%, list=9%, signal=40% |
| 2556 | tags=33%, list=5%, signal=35% |
| 7095 | tags=41%, list=13%, signal=47% |
| 7188 | tags=42%, list=13%, signal=48% |
| 3701 | tags=40%, list=7%, signal=43% |
| 5050 | tags=42%, list=9%, signal=47% |
| 7342 | tags=54%, list=13%, signal=63% |
| 4998 | tags=29%, list=9%, signal=32% |
| 6940 | tags=45%, list=13%, signal=52% |
| 6887 | tags=51%, list=12%, signal=58% |
| 7080 | tags=47%, list=13%, signal=54% |
| 7469 | tags=36%, list=14%, signal=42% |

9160 tags=50%, list=17%, signal=60%
6607 tags=51%, list=12%, signal=58%
5998 tags=36%, list=11%, signal=41%
9638 tags=51%, list=17%, signal=62%
7038 tags=45%, list=13%, signal=52%
7823 tags=45%, list=14%, signal=53%
9160 tags=55%, list=17%, signal=66%
6453 tags=41%, list=12%, signal=46%
7212 tags=42%, list=13%, signal=48%
5050 tags=33%, list=9%, signal=36%
7640 tags=43%, list=14%, signal=50%
7275 tags=47%, list=13%, signal=54%
7188 tags=40%, list=13%, signal=46%
8004 tags=34%, list=14%, signal=40%
6733 tags=39%, list=12%, signal=44%
5582 tags=47%, list=10%, signal=52%
4998 tags=35%, list=9%, signal=39%
6733 tags=31%, list=12%, signal=35%
6356 tags=33%, list=12%, signal=38%
6165 tags=33%, list=11%, signal=37%
8255 tags=51%, list=15%, signal=60%
8533 tags=50%, list=15%, signal=59%
6732 tags=41%, list=12%, signal=46%
3697 tags=31%, list=7%, signal=33%
7196 tags=41%, list=13%, signal=47%
6887 tags=33%, list=12%, signal=38%
9851 tags=50%, list=18%, signal=61%
9353 tags=48%, list=17%, signal=57%
6865 tags=36%, list=12%, signal=41%
10266 tags=52%, list=19%, signal=64%
9638 tags=54%, list=17%, signal=65%
12726 tags=73%, list=23%, signal=95%
2758 tags=18%, list=5%, signal=19%
4842 tags=25%, list=9%, signal=28%
7631 tags=37%, list=14%, signal=43%
6115 tags=32%, list=11%, signal=36%
5085 tags=22%, list=9%, signal=24%
2514 tags=19%, list=5%, signal=20%
9727 tags=43%, list=18%, signal=53%
3678 tags=19%, list=7%, signal=20%
7224 tags=41%, list=13%, signal=47%
4767 tags=23%, list=9%, signal=25%
10547 tags=51%, list=19%, signal=64%
10547 tags=49%, list=19%, signal=60%
3704 tags=17%, list=7%, signal=18%
6888 tags=29%, list=12%, signal=33%
6672 tags=31%, list=12%, signal=35%
3980 tags=21%, list=7%, signal=23%
5454 tags=23%, list=10%, signal=25%
5270 tags=17%, list=10%, signal=19%
8460 tags=30%, list=15%, signal=35%

4671 tags=21%, list=8%, signal=22%
6345 tags=27%, list=11%, signal=31%
5454 tags=18%, list=10%, signal=20%
9713 tags=37%, list=18%, signal=44%
2434 tags=14%, list=4%, signal=15%
3520 tags=15%, list=6%, signal=16%
1761 tags=7%, list=3%, signal=8%
3569 tags=16%, list=6%, signal=17%
5209 tags=16%, list=9%, signal=18%
10547 tags=38%, list=19%, signal=47%
4193 tags=25%, list=8%, signal=27%
3472 tags=10%, list=6%, signal=11%
5827 tags=23%, list=11%, signal=26%
7080 tags=26%, list=13%, signal=29%
7174 tags=19%, list=13%, signal=22%
