# Supplementary material for: Alteration in the Immune Microenvironment Based on APC Status in MSS/pMMR Colon Cancer
Source: Dis Markers. 2022 Jul 27;2022:3592990. doi: 10.1155/2022/3592990 (PMC9348928; doi:10.1155/2022/3592990)
Supplement: Supplementary Materials — Supplement Table S1: all different expression genes (DEGs) listed between APC-wt and APC-mt MSS/pMMR colon cancer from the overall gene expression profile of all 338 colon cancer cases obtained from TCGA database. Supplementary Table S2: APC-mt vs. APC-wt MSS/pMMR colon cancer, 379 genes were downregulated, and 117 genes were upregulated. Supplementary Table S3: APC-wt MSS/pMMR (77 samples) COAD samples in the cohort retrieved from TCGA performed gene set enrichment analysis enriched (GSEA) in 115 KEGG pathways. Supplementary Table S4: APC-wt MSS/pMMR COAD samples in the cohort retrieved from TCGA performed gene set enrichment analysis downregulated (GSEA) in 62 KEGG pathways. Supplementary Table S5: 65 overlapping genes among the DEGs (496 genes related to APC status) and immunophenotypes (1297 genes shown in Supplementary Table S6). Supplementary Table S6: 1297 immune-related genes. Supplementary Table S7: Lasso and Cox regression analyze APC-related DEGs constructing immune scoring model. Supplement Figure 1: the expression of CTLA4, PD-L1, and immune score and stromal score in the KRAS-mt/MSS group was downregulated compared with KRAS-wt/MSS. Supplement Figure 2: the expression of not only PD-1, PD-L1, and CTLA4 but also immune score, ESTIMATE score, and stromal score did not differ between TP53-wt/MSS and TP53-mt/MSS groups. Supplement Figure 3: in the combination of different genotypes of KRAS, TP53 and APC, the TMB and immune score are significantly higher in wild type than KRAS/TP53/APC mutant type colon cancer. Supplement Figure 4: neither mutations in KRAS nor TP53 could affect the proportion of infiltrating immune cell types in MSS/pMMR colon cancer. [file 3592990.f1.zip › Supplement table S4.pdf]

APC-wt colon cancer was significantly down-regulated in 62 KEGG pathwa

NAME	GS<br>fo	GS DETAIL	SIZE	ES	NES	NOM p-val	FDR q-val	FWER p-va
KEGG_PARK	KEGG_PARK	Details ..	101	-0.716056	-2.06673	0.0020121	0.0245608	0.036
KEGG_OXID	KEGG_OXID	Details ..	102	-0.702676	-2.009859	0.0060241	0.0271404	0.063
KEGG_RIBO	KEGG_RIBO	Details ..	88	-0.870405	-1.941139	0.0020492	0.0408718	0.115
KEGG_HUNT	KEGG_HUNT	Details ..	161	-0.554882	-1.90868	0.006135	0.0446227	0.149
KEGG_ALZH	KEGG_ALZH	Details ..	145	-0.530323	-1.844796	0.0120724	0.0691133	0.236
KEGG_BASE	KEGG_BASE	Details ..	35	-0.664057	-1.755026	0.0204918	0.1289716	0.37
KEGG_PROT	KEGG_PROT	Details ..	45	-0.694172	-1.753771	0.0349076	0.1113154	0.37
KEGG_CARD	KEGG_CARD	Details ..	74	-0.477145	-1.71678	0.0142857	0.130116	0.424
KEGG_DNA_	KEGG_DNA_	Details ..	36	-0.719833	-1.692602	0.0553279	0.1374188	0.458
KEGG_SPLI	KEGG_SPLI	Details ..	123	-0.591338	-1.663708	0.063786	0.1474051	0.509
KEGG_TERP	KEGG_TERP	Details ..	15	-0.667831	-1.596217	0.0745342	0.2028757	0.622
KEGG_GLYO	KEGG_GLYO	Details ..	16	-0.646236	-1.572221	0.0631365	0.2158829	0.671
KEGG_RNA_	KEGG_RNA_	Details ..	29	-0.569037	-1.559251	0.0738523	0.2143454	0.687
KEGG_MISM	KEGG_MISM	Details ..	23	-0.648977	-1.548417	0.0989474	0.2112742	0.702
KEGG_PYRU	KEGG_PYRU	Details ..	39	-0.481	-1.468151	0.1	0.2969513	0.803
KEGG_PYRI	KEGG_PYRI	Details ..	96	-0.459985	-1.460031	0.1182365	0.2904459	0.817
KEGG_RNA_	KEGG_RNA_	Details ..	56	-0.481067	-1.455655	0.1361789	0.2793357	0.82
KEGG_BUTA	KEGG_BUTA	Details ..	34	-0.474558	-1.436069	0.1187123	0.2898105	0.84
KEGG_PENT	KEGG_PENT	Details ..	26	-0.503913	-1.423198	0.1391129	0.2919565	0.852
KEGG_NUCL	KEGG_NUCL	Details ..	44	-0.504924	-1.41	0.1666667	0.2933846	0.866
KEGG_AMIN	KEGG_AMIN	Details ..	41	-0.564044	-1.409759	0.1759834	0.2795571	0.866
KEGG_PERO	KEGG_PERO	Details ..	78	-0.440175	-1.395769	0.1386139	0.2830099	0.873
KEGG_FRUC	KEGG_FRUC	Details ..	34	-0.476971	-1.388244	0.1569417	0.2794099	0.876
KEGG_GLUT	KEGG_GLUT	Details ..	49	-0.438978	-1.383554	0.122	0.2729605	0.88
KEGG_CITR	KEGG_CITR	Details ..	30	-0.568063	-1.374652	0.1965812	0.2723741	0.889
KEGG_STER	KEGG_STER	Details ..	16	-0.571582	-1.363814	0.1686747	0.2740345	0.897
KEGG_HOMO	KEGG_HOMO	Details ..	26	-0.517383	-1.318483	0.2031873	0.3162612	0.931
KEGG_GLYC	KEGG_GLYC	Details ..	25	-0.469945	-1.306371	0.1936685	0.3200841	0.937
KEGG_ONE_	KEGG_ONE_	Details ..	17	-0.515998	-1.266169	0.2530364	0.3604342	0.961
KEGG_PROT	KEGG_PROT	Details ..	23	-0.490683	-1.257894	0.255489	0.3596298	0.963
KEGG_VALI	KEGG_VALI	Details ..	44	-0.442422	-1.221959	0.2563025	0.3949715	0.971
KEGG_LINO	KEGG_LINO	Details ..	29	-0.379625	-1.207919	0.2144289	0.4018055	0.974
KEGG_CELL	KEGG_CELL	Details ..	124	-0.388234	-1.169462	0.326572	0.4429504	0.986
KEGG_MATU	KEGG_MATU	Details ..	25	-0.434184	-1.150775	0.306	0.4574757	0.99
KEGG_ARGI	KEGG_ARGI	Details ..	51	-0.343238	-1.115959	0.3131313	0.4957393	0.99
KEGG_DRUG	KEGG_DRUG	Details ..	51	-0.32563	-1.099853	0.3143418	0.5072616	0.992
KEGG_PHEN	KEGG_PHEN	Details ..	17	-0.367574	-1.081574	0.3393939	0.5230021	0.993
KEGG_GLYC	KEGG_GLYC	Details ..	25	-0.342284	-1.070383	0.3672199	0.527421	0.994
KEGG_GLYC	KEGG_GLYC	Details ..	61	-0.324414	-1.051483	0.3827161	0.5442873	0.995
KEGG_DRUG	KEGG_DRUG	Details ..	71	-0.316827	-1.047447	0.3817098	0.5379832	0.995
KEGG_META	KEGG_META	Details ..	69	-0.322765	-1.029421	0.4135802	0.5521194	0.995
KEGG_CYST	KEGG_CYST	Details ..	34	-0.334621	-1.022994	0.4263261	0.5490462	0.995
KEGG_PROP	KEGG_PROP	Details ..	32	-0.358874	-1.021089	0.4218107	0.5388455	0.996
KEGG_OLFA	KEGG_OLFA	Details ..	384	-0.350146	-1.016448	0.483366	0.5337167	0.996
KEGG_ALAN	KEGG_ALAN	Details ..	32	-0.327231	-1.014323	0.4265594	0.5253028	0.996
KEGG_RIBO	KEGG_RIBO	Details ..	15	-0.367211	-1.005421	0.4466801	0.5275526	0.997
KEGG_RETI	KEGG_RETI	Details ..	63	-0.292871	-0.974082	0.4736842	0.5631051	0.998
KEGG_PORP	KEGG_PORP	Details ..	41	-0.318379	-0.96892	0.4826176	0.5585555	0.998
KEGG_HIST	KEGG_HIST	Details ..	27	-0.303624	-0.968445	0.4970531	0.5478495	0.998

KEGG_ASCOI	KEGG_ASCOI	Details ..	25	-0.348523	-0.946445	0.5155925	0.5697056	0.999
KEGG_FATT'	KEGG_FATTY_ACID_MET		42	-0.319607	-0.933691	0.5234216	0.577474	0.999
KEGG_PROX	KEGG_PROXIMAL_TUBUL		23	-0.305171	-0.918246	0.5699589	0.5894022	0.999
KEGG_NITRO	KEGG_NITROGEN_METAB		23	-0.293484	-0.877401	0.6274132	0.6394163	0.999
KEGG_BETA	KEGG_BETA_ALANINE_M		22	-0.293683	-0.874249	0.6121212	0.6325101	0.999
KEGG_GLYCI	KEGG_GLYCEROPHOSPHO		71	-0.243909	-0.862458	0.6600791	0.6388145	0.999
KEGG_N_GL'	KEGG_N_GLYCAN_BIOSY		46	-0.301109	-0.856141	0.62	0.6366682	0.999
KEGG_GLYC	KEGG_GLYCINE_SERINE		31	-0.270687	-0.822436	0.6839917	0.675233	0.999
KEGG_GLYCI	KEGG_GLYCEROLIPID_M		43	-0.241441	-0.777295	0.8056112	0.7314289	0.999
KEGG_PENT(	KEGG_PENTOSE_AND_GL		27	-0.260357	-0.722586	0.7884232	0.7980396	1
KEGG_BIOS'	KEGG_BIOSYNTHESIS_O		20	-0.244227	-0.648088	0.8902439	0.8794258	1
KEGG_BASAI	KEGG_BASAL_TRANSCRI		35	-0.209067	-0.639179	0.8897638	0.8747085	1
KEGG_ALPHA	KEGG_ALPHA_LINOLENI		19	-0.201553	-0.586172	0.9566116	0.9119418	1

ays

RANK AT M	LEADING EDGE
5518	tags=58%, list=10%, signal=65%
2542	tags=51%, list=5%, signal=53%
3757	tags=83%, list=7%, signal=89%
6814	tags=46%, list=12%, signal=52%
2568	tags=37%, list=5%, signal=38%
13978	tags=77%, list=25%, signal=103%
8682	tags=69%, list=16%, signal=82%
2483	tags=27%, list=4%, signal=28%
6010	tags=64%, list=11%, signal=72%
7940	tags=45%, list=14%, signal=52%
3774	tags=40%, list=7%, signal=43%
7995	tags=56%, list=14%, signal=66%
7764	tags=41%, list=14%, signal=48%
13014	tags=61%, list=24%, signal=80%
6928	tags=38%, list=13%, signal=44%
12982	tags=51%, list=23%, signal=67%
3802	tags=32%, list=7%, signal=34%
12272	tags=50%, list=22%, signal=64%
9992	tags=42%, list=18%, signal=52%
8276	tags=41%, list=15%, signal=48%
12922	tags=49%, list=23%, signal=64%
7671	tags=35%, list=14%, signal=40%
3571	tags=32%, list=6%, signal=35%
9345	tags=45%, list=17%, signal=54%
4068	tags=37%, list=7%, signal=40%
2636	tags=38%, list=5%, signal=39%
12982	tags=50%, list=23%, signal=65%
10701	tags=40%, list=19%, signal=50%
14984	tags=71%, list=27%, signal=97%
8022	tags=35%, list=15%, signal=41%
11443	tags=43%, list=21%, signal=54%
17582	tags=45%, list=32%, signal=66%
5610	tags=34%, list=10%, signal=38%
3504	tags=20%, list=6%, signal=21%
7153	tags=29%, list=13%, signal=34%
15409	tags=39%, list=28%, signal=54%
7214	tags=41%, list=13%, signal=47%
4117	tags=20%, list=7%, signal=22%
6334	tags=26%, list=11%, signal=30%
9345	tags=31%, list=17%, signal=37%
9345	tags=32%, list=17%, signal=38%
2955	tags=21%, list=5%, signal=22%
13654	tags=41%, list=25%, signal=54%
24086	tags=63%, list=44%, signal=110%
9252	tags=34%, list=17%, signal=41%
3396	tags=27%, list=6%, signal=28%
13548	tags=32%, list=25%, signal=42%
2910	tags=15%, list=5%, signal=15%
10618	tags=41%, list=19%, signal=50%

26066 tags=64%, list=47%, signal=121%
11321 tags=36%, list=20%, signal=45%
10918 tags=26%, list=20%, signal=32%
9491 tags=26%, list=17%, signal=31%
1993 tags=18%, list=4%, signal=19%
5790 tags=18%, list=10%, signal=20%
18187 tags=46%, list=33%, signal=68%
5569 tags=23%, list=10%, signal=25%
3254 tags=16%, list=6%, signal=17%
26066 tags=56%, list=47%, signal=105%
1380 tags=10%, list=2%, signal=10%
13395 tags=23%, list=24%, signal=30%
18202 tags=37%, list=33%, signal=55%
