# Supplementary material for: Alteration in the Immune Microenvironment Based on APC Status in MSS/pMMR Colon Cancer
Source: Dis Markers. 2022 Jul 27;2022:3592990. doi: 10.1155/2022/3592990 (PMC9348928; doi:10.1155/2022/3592990)
Supplement: Supplementary Materials — Supplement Table S1: all different expression genes (DEGs) listed between APC-wt and APC-mt MSS/pMMR colon cancer from the overall gene expression profile of all 338 colon cancer cases obtained from TCGA database. Supplementary Table S2: APC-mt vs. APC-wt MSS/pMMR colon cancer, 379 genes were downregulated, and 117 genes were upregulated. Supplementary Table S3: APC-wt MSS/pMMR (77 samples) COAD samples in the cohort retrieved from TCGA performed gene set enrichment analysis enriched (GSEA) in 115 KEGG pathways. Supplementary Table S4: APC-wt MSS/pMMR COAD samples in the cohort retrieved from TCGA performed gene set enrichment analysis downregulated (GSEA) in 62 KEGG pathways. Supplementary Table S5: 65 overlapping genes among the DEGs (496 genes related to APC status) and immunophenotypes (1297 genes shown in Supplementary Table S6). Supplementary Table S6: 1297 immune-related genes. Supplementary Table S7: Lasso and Cox regression analyze APC-related DEGs constructing immune scoring model. Supplement Figure 1: the expression of CTLA4, PD-L1, and immune score and stromal score in the KRAS-mt/MSS group was downregulated compared with KRAS-wt/MSS. Supplement Figure 2: the expression of not only PD-1, PD-L1, and CTLA4 but also immune score, ESTIMATE score, and stromal score did not differ between TP53-wt/MSS and TP53-mt/MSS groups. Supplement Figure 3: in the combination of different genotypes of KRAS, TP53 and APC, the TMB and immune score are significantly higher in wild type than KRAS/TP53/APC mutant type colon cancer. Supplement Figure 4: neither mutations in KRAS nor TP53 could affect the proportion of infiltrating immune cell types in MSS/pMMR colon cancer. [file 3592990.f1.zip › Supplement table S5.pdf]

| id | CD8A | CTSE | HLA-DMA | HLA-DOA | HLA-DPA1 | HLA-DPB1 | HLA-DQA1 | HLA-DRB5 |
|---|---|---|---|---|---|---|---|---|
| TCGA-A6- | 8.751016 | 11.36869 | 19.6373 | 5.042987 | 29.23252 | 69.78561 | 12.76868 | 64.01305 |
| TCGA-AA- | 3.646071 | 26.08534 | 26.97191 | 10.69193 | 47.63291 | 88.44315 | 25.42544 | 137.7578 |
| TCGA-A6- | 2.636639 | 3.547009 | 34.8185 | 3.226396 | 18.42178 | 47.55057 | 7.667546 | 104.4996 |
| TCGA-AA- | 3.077575 | 8.298292 | 25.41077 | 4.400164 | 45.25592 | 63.16302 | 18.34627 | 40.40879 |
| TCGA-AA- | 5.217013 | 45.43267 | 27.83273 | 7.409244 | 64.40199 | 77.46159 | 33.59417 | 38.33185 |
| TCGA-A6- | 1.790883 | 9.789555 | 17.74994 | 3.441924 | 42.44145 | 59.94634 | 19.07182 | 33.79623 |
| TCGA-A6- | 4.490901 | 10.44536 | 16.91205 | 2.428461 | 21.73097 | 56.22349 | 10.98451 | 22.98934 |
| TCGA-AA- | 2.03607 | 6.603206 | 21.23472 | 6.60332 | 52.62088 | 78.56696 | 11.61374 | 52.58203 |
| TCGA-AA- | 2.589059 | 4.891991 | 24.44303 | 5.409295 | 51.50351 | 70.25189 | 21.08155 | 50.90847 |
| TCGA-AA- | 7.12946 | 16.08297 | 33.45584 | 4.299598 | 60.06354 | 79.6039 | 15.55362 | 89.24484 |
| TCGA-AZ- | 5.376426 | 5.748001 | 58.30518 | 15.21909 | 58.83155 | 119.6459 | 17.12014 | 96.47117 |
| TCGA-AZ- | 1.637148 | 4.159788 | 16.6447 | 2.965801 | 16.6223 | 27.83127 | 3.680062 | 69.30957 |
| TCGA-AA- | 3.59911 | 5.171708 | 25.90211 | 10.0828 | 49.78667 | 82.12983 | 9.979017 | 99.84583 |
| TCGA-A6- | 2.288574 | 9.988662 | 13.94164 | 4.181337 | 26.47259 | 35.37582 | 10.97659 | 139.7584 |
| TCGA-AA- | 4.436302 | 9.299623 | 23.40756 | 6.603295 | 51.84673 | 86.00085 | 25.81293 | 87.37732 |
| TCGA-AA- | 3.12625 | 11.38784 | 26.90852 | 8.794309 | 47.47649 | 105.2342 | 28.33306 | 46.18831 |
| TCGA-AA- | 2.111293 | 19.45581 | 26.21079 | 6.262307 | 66.6046 | 75.39626 | 14.21382 | 178.1329 |
| TCGA-A6- | 3.417988 | 10.82997 | 15.11755 | 4.238438 | 30.10827 | 41.12545 | 10.50846 | 162.7215 |
| TCGA-A6- | 1.308305 | 7.5612 | 17.43135 | 4.669262 | 28.28525 | 41.98978 | 12.84129 | 28.89653 |
| TCGA-AZ- | 2.290232 | 9.540809 | 18.84771 | 3.568438 | 19.50056 | 35.9684 | 2.998206 | 48.61575 |
| TCGA-A6- | 1.449141 | 5.197278 | 21.59498 | 5.77279 | 53.16 | 82.59108 | 15.0005 | 58.05817 |
| TCGA-AZ- | 3.578435 | 22.78405 | 14.10372 | 3.141381 | 19.69491 | 31.44212 | 7.522044 | 62.24206 |
| TCGA-AG- | 3.415995 | 18.99528 | 27.78807 | 4.94009 | 45.21379 | 85.66169 | 24.46798 | 66.42746 |
| TCGA-A6- | 29.01511 | 7.084651 | 68.95831 | 10.78769 | 87.72312 | 131.9665 | 33.51671 | 195.9994 |
| TCGA-A6- | 1.373352 | 7.597975 | 12.82521 | 2.61762 | 16.85084 | 36.33106 | 8.341857 | 25.62398 |
| TCGA-F4-( | 1.252363 | 18.84644 | 8.385929 | 2.078957 | 21.51925 | 33.25121 | 2.717961 | 27.85203 |
| TCGA-AA- | 5.559213 | 5.405224 | 27.03049 | 5.085856 | 41.89422 | 72.62791 | 19.68506 | 160.696 |
| TCGA-AA- | 10.97472 | 16.28374 | 33.29959 | 6.726225 | 52.20576 | 97.96983 | 17.91439 | 196.1482 |
| TCGA-AZ- | 1.744062 | 8.917077 | 12.32711 | 3.422773 | 16.11941 | 40.75803 | 11.36437 | 17.40573 |
| TCGA-A6- | 1.542027 | 9.649535 | 16.81822 | 5.333378 | 43.76093 | 60.49968 | 12.9365 | 27.73538 |
| TCGA-AA- | 1.012443 | 16.80733 | 13.02063 | 3.864196 | 27.87216 | 51.09788 | 4.167748 | 43.47053 |
| TCGA-AA- | 4.200626 | 6.981942 | 17.67019 | 4.538031 | 34.81534 | 49.19698 | 14.56 | 115.1548 |
| TCGA-AA- | 10.32255 | 10.67053 | 69.52953 | 13.87638 | 85.59749 | 123.65 | 22.20427 | 158.8847 |
| TCGA-A6- | 2.14459 | 26.31646 | 15.6404 | 4.612847 | 23.76664 | 50.33149 | 14.84302 | 25.93384 |
| TCGA-AA- | 6.120254 | 2.479302 | 57.00314 | 6.116938 | 49.14619 | 60.13509 | 13.87942 | 58.49021 |
| TCGA-AA- | 5.131377 | 34.64474 | 19.50935 | 6.178578 | 55.13118 | 74.34797 | 16.4596 | 136.957 |
| TCGA-AZ- | 0.559153 | 1.091176 | 8.723473 | 2.061695 | 16.42561 | 26.91545 | 3.940822 | 16.11039 |
| TCGA-AA- | 1.561461 | 5.798327 | 22.22851 | 5.968793 | 51.45146 | 68.29499 | 10.4124 | 59.55713 |
| TCGA-A6- | 1.793578 | 4.238299 | 22.7513 | 5.784149 | 50.87614 | 81.36725 | 14.99671 | 76.32838 |
| TCGA-AA- | 2.775234 | 5.026084 | 21.15173 | 4.369944 | 55.03037 | 67.43552 | 12.44768 | 56.26742 |
| TCGA-AA- | 4.497732 | 5.661064 | 45.17968 | 8.524822 | 80.10393 | 97.74155 | 26.87695 | 108.2336 |
| TCGA-A6- | 3.122876 | 6.549139 | 19.8373 | 4.524967 | 52.16871 | 69.04914 | 11.48572 | 52.42437 |
| TCGA-CM | 1.651833 | 0.178662 | 12.43914 | 5.440165 | 22.57308 | 47.07367 | 13.80355 | 29.24238 |
| TCGA-G4- | 0.39882 | 0.055857 | 11.78376 | 1.136855 | 11.58739 | 17.2126 | 3.393772 | 11.99301 |
| TCGA-AA- | 18.45994 | 33.61735 | 152.6543 | 24.31806 | 212.536 | 261.3116 | 33.39061 | 150.625 |
| TCGA-AD- | 5.204179 | 0.120115 | 45.58606 | 7.525332 | 71.50572 | 62.43051 | 13.11463 | 212.1836 |
| TCGA-A6- | 1.595711 | 0.292917 | 7.88228 | 3.292227 | 33.99632 | 23.38219 | 13.95885 | 8.291327 |
| TCGA-CM | 0.108827 | 0.423038 | 4.459092 | 0.161942 | 1.759379 | 3.815703 | 0.318085 | 3.071448 |
| TCGA-AG- | 6.233187 | 0.05063 | 32.10031 | 8.9829 | 56.60556 | 111.999 | 13.78287 | 123.4263 |
| TCGA-G4- | 0.8542 | 52.12293 | 9.745838 | 1.020214 | 7.039667 | 12.05709 | 2.09786 | 6.954826 |
| TCGA-DM | 0.105844 | 3.3241 | 7.576845 | 0.211716 | 1.405245 | 3.100788 | 0.451157 | 7.504463 |
| TCGA-AA- | 2.664368 | 202.5019 | 26.12606 | 5.230942 | 33.8347 | 72.21758 | 14.84327 | 76.80476 |
| TCGA-DM | 0.20479 | 167.6312 | 5.259707 | 0.210791 | 1.956802 | 6.617664 | 1.298998 | 3.261953 |
| TCGA-A6- | 4.199944 | 2.665021 | 12.04625 | 4.939114 | 17.66101 | 34.3275 | 11.09081 | 19.13678 |
| TCGA-G4- | 7.02935 | 1.455138 | 31.10002 | 6.641121 | 46.75864 | 57.69105 | 18.95896 | 55.86892 |
| TCGA-A6- | 0.919028 | 0.421638 | 14.05329 | 1.548725 | 17.08627 | 52.05267 | 5.482881 | 22.96153 |

| | | | | | | | | |
|---|---|---|---|---|---|---|---|---|
| TCGA-AA- | 1.518654 | 333.9 | 15.85847 | 3.445604 | 26.99531 | 38.83672 | 9.985909 | 22.96306 |
| TCGA-AA- | 2.326371 | 43.32056 | 23.58957 | 2.884078 | 39.25045 | 39.42332 | 13.16603 | 43.81538 |
| TCGA-A6- | 5.199671 | 5.676057 | 20.75466 | 3.902973 | 11.79686 | 49.99831 | 10.65167 | 43.07929 |
| TCGA-AU- | 0.377474 | 0.04505 | 13.28641 | 3.784992 | 21.56608 | 31.39477 | 9.067589 | 29.2798 |
| TCGA-AA- | 1.473626 | 3.367752 | 10.67711 | 0.846236 | 9.109524 | 16.17748 | 2.288937 | 21.50063 |
| TCGA-AA- | 3.547617 | 1.867607 | 30.01443 | 9.333305 | 65.2913 | 150.4356 | 33.78089 | 339.294 |
| TCGA-AA- | 0.765972 | 0.416854 | 17.37425 | 1.491679 | 13.55128 | 19.49776 | 6.48301 | 9.352112 |
| TCGA-DM | 0.935917 | 5.072184 | 5.488086 | 0.913672 | 5.700094 | 12.55161 | 3.843399 | 7.97794 |
| TCGA-AD- | 5.486817 | 49.66904 | 13.95859 | 4.35321 | 27.18422 | 48.83243 | 18.61098 | 97.08654 |
| TCGA-CK- | 0.424618 | 2.345851 | 15.77101 | 1.919173 | 15.0306 | 23.04745 | 3.519166 | 19.8171 |
| TCGA-DM | 0.07299 | 121.1537 | 8.906914 | 0.39067 | 3.691679 | 8.544654 | 0.71362 | 10.90843 |
| TCGA-A6- | 2.317234 | 2.187177 | 7.331669 | 4.932439 | 32.58712 | 20.32795 | 20.33179 | 50.95937 |
| TCGA-AA- | 1.72167 | 53.79645 | 13.94282 | 1.847762 | 19.72974 | 26.1279 | 6.087193 | 21.11387 |
| TCGA-DM | 0.264711 | 9.770733 | 9.954514 | 0.337455 | 5.62501 | 8.954911 | 2.152503 | 70.70494 |
| TCGA-AA- | 0.651714 | 2.916201 | 15.41086 | 2.439954 | 30.64563 | 41.23999 | 9.930246 | 90.84862 |
| TCGA-CM | 5.059399 | 0.114091 | 34.09361 | 11.1403 | 49.36705 | 76.8669 | 31.37418 | 49.661 |
| TCGA-AA- | 1.192699 | 2.839748 | 4.174299 | 0.720217 | 3.743308 | 7.579845 | 1.474879 | 8.190224 |
| TCGA-AM | 6.087892 | 2.479455 | 52.60153 | 17.452 | 73.32505 | 103.8809 | 19.1108 | 118.1659 |
| TCGA-A6- | 4.763907 | 1.297982 | 99.85769 | 20.29595 | 101.2745 | 213.7025 | 38.71015 | 123.0142 |
| TCGA-A6- | 0.04794 | 0.065224 | 6.233359 | 1.128999 | 6.364783 | 17.35266 | 1.827558 | 17.37523 |
| TCGA-AA- | 2.990442 | 0.017613 | 59.8545 | 21.37638 | 118.4296 | 123.4348 | 20.13668 | 162.1913 |
| TCGA-AA- | 4.541968 | 2.540923 | 16.36976 | 3.207324 | 32.72025 | 38.65391 | 12.65852 | 15.5127 |
| TCGA-AA- | 4.041065 | 123.7752 | 75.99882 | 3.104328 | 89.96116 | 81.15577 | 21.4348 | 399.772 |
| TCGA-QG | 0.363116 | 1.859888 | 11.30318 | 0.585255 | 6.725016 | 11.02022 | 3.084345 | 17.38778 |
| TCGA-AA- | 1.744951 | 1.089983 | 11.83538 | 4.860924 | 30.95184 | 33.36553 | 6.926142 | 67.41864 |
| TCGA-AA- | 1.646404 | 1.368887 | 10.00189 | 1.159888 | 9.548715 | 15.75015 | 3.226978 | 5.737697 |
| TCGA-A6- | 0.824875 | 4.693149 | 12.08043 | 1.40375 | 16.37202 | 38.17661 | 5.206004 | 30.20665 |
| TCGA-A6- | 2.760422 | 17.92742 | 12.77303 | 6.693625 | 67.20359 | 44.34702 | 13.60663 | 34.10551 |
| TCGA-AA- | 3.041107 | 1.257814 | 17.02628 | 1.597657 | 14.21222 | 20.74856 | 2.750481 | 33.82074 |
| TCGA-DM | 0.617278 | 0.388579 | 4.683826 | 0.479842 | 4.552012 | 8.013233 | 1.249414 | 25.00404 |
| TCGA-NH | 0.516143 | 0.148549 | 7.330809 | 1.582352 | 7.348377 | 19.52792 | 2.853558 | 15.80228 |
| TCGA-A6- | 0.190381 | 8.278709 | 11.08901 | 3.594792 | 16.5221 | 33.17836 | 6.879959 | 33.89534 |
| TCGA-AD- | 7.721188 | 1.35508 | 81.80862 | 24.34973 | 98.0746 | 255.1281 | 51.5606 | 153.2468 |
| TCGA-CK- | 0.252611 | 24.33449 | 5.205629 | 1.432551 | 7.141607 | 12.15535 | 2.708077 | 29.42751 |
| TCGA-A6- | 0.259424 | 0.120326 | 2.790291 | 0.897203 | 6.489694 | 8.337047 | 2.09095 | 3.877096 |
| TCGA-A6- | 0.410221 | 0.131779 | 7.827612 | 1.832052 | 14.46236 | 20.72702 | 5.571487 | 13.88534 |
| TCGA-AA- | 1.701479 | 1.986981 | 18.17863 | 3.62175 | 33.73244 | 37.18176 | 8.457569 | 17.21638 |
| TCGA-D5- | 2.680585 | 0.013002 | 22.70213 | 5.656742 | 37.76717 | 49.84007 | 15.83999 | 60.88452 |
| TCGA-AA- | 12.00804 | 89.09501 | 20.10352 | 5.426278 | 37.95019 | 69.68137 | 20.25365 | 56.90208 |
| TCGA-AA- | 0.869417 | 0 | 11.08068 | 1.587708 | 14.82317 | 18.1488 | 1.956852 | 27.5826 |
| TCGA-CM | 1.268639 | 58.95068 | 41.4952 | 1.530307 | 16.5656 | 16.61352 | 2.870166 | 52.17475 |
| TCGA-AA- | 0.352709 | 1.286935 | 16.4161 | 0.842259 | 9.548004 | 18.55754 | 0.46259 | 17.10841 |
| TCGA-CK- | 1.302244 | 15.42691 | 25.62536 | 2.835132 | 31.75897 | 50.41312 | 2.588746 | 84.96238 |
| TCGA-AA- | 0.549041 | 8.590411 | 7.217633 | 1.392474 | 14.03501 | 16.56127 | 3.715647 | 38.74309 |
| TCGA-DM | 0.925029 | 0.015442 | 7.878095 | 0.91498 | 8.467354 | 16.00483 | 4.046008 | 56.51692 |
| TCGA-D5- | 0.654288 | 0.483462 | 2.459024 | 0.306539 | 2.756503 | 3.583899 | 0.958728 | 2.50522 |
| TCGA-A6- | 3.142878 | 25.59947 | 46.33289 | 10.20315 | 69.16904 | 152.9648 | 14.52749 | 195.974 |
| TCGA-D5- | 3.300734 | 0.391589 | 14.78912 | 2.939826 | 18.63012 | 35.96829 | 3.572452 | 44.08398 |
| TCGA-NH | 1.287277 | 19.94494 | 8.307355 | 2.153607 | 10.04245 | 23.12753 | 4.870895 | 11.23498 |
| TCGA-DM | 3.334924 | 0.005301 | 7.67116 | 1.03396 | 9.019837 | 13.91943 | 6.131591 | 6.651939 |
| TCGA-A6- | 1.411242 | 7.299994 | 4.759071 | 1.47445 | 10.93847 | 11.53439 | 4.570423 | 9.651618 |
| TCGA-D5- | 3.194154 | 41.32846 | 14.06411 | 5.048592 | 32.44028 | 44.4036 | 17.71259 | 95.08324 |
| TCGA-NH | 4.58936 | 19.71364 | 24.83265 | 7.950315 | 32.95552 | 90.81975 | 25.94618 | 56.41855 |
| TCGA-A6- | 0.704683 | 0.3835 | 7.016337 | 0.930079 | 7.592844 | 10.90547 | 1.526277 | 25.52108 |
| TCGA-DM | 0.754625 | 0.216147 | 8.72725 | 0.839418 | 6.902441 | 16.34141 | 2.29892 | 9.469393 |
| TCGA-AA- | 3.233197 | 0.246338 | 12.50059 | 3.034002 | 32.91805 | 40.83466 | 13.90867 | 25.69956 |
| TCGA-AA- | 1.525442 | 8.259336 | 22.76921 | 1.804694 | 15.86364 | 85.60882 | 1.600536 | 167.5427 |
| TCGA-AA- | 7.515486 | 165.5425 | 105.7853 | 54.9586 | 201.7354 | 234.4458 | 71.86832 | 206.3851 |

| | | | | | | | | |
|---|---|---|---|---|---|---|---|---|
| TCGA-3L-/ | 0.721261 | 0.236867 | 9.032797 | 2.72023 | 15.89695 | 29.03962 | 7.512218 | 18.47082 |
| TCGA-AA- | 4.910503 | 2.964387 | 25.75898 | 7.059609 | 45.12173 | 76.8371 | 19.68768 | 136.3416 |
| TCGA-G4- | 0.562089 | 10.58058 | 3.989636 | 0.406016 | 3.220768 | 5.614046 | 0.989154 | 5.992051 |
| TCGA-G4- | 0.117993 | 0.173912 | 3.680388 | 0.507661 | 4.363953 | 8.744209 | 0.484889 | 11.68386 |
| TCGA-A6- | 0.1146 | 0.048724 | 1.592112 | 0.778518 | 6.285231 | 4.327197 | 4.952435 | 0.57841 |
| TCGA-AA- | 1.412246 | 0.893053 | 19.19753 | 3.423179 | 31.06741 | 51.17573 | 6.772305 | 37.26023 |
| TCGA-A6- | 2.769815 | 21.47806 | 23.94162 | 5.785436 | 54.39993 | 76.28654 | 17.12733 | 49.15327 |
| TCGA-A6- | 0.319501 | 0.026614 | 11.08085 | 1.37612 | 7.62634 | 8.387997 | 1.248558 | 43.178 |
| TCGA-AD- | 0.341358 | 9.86425 | 15.44465 | 0.16984 | 6.015416 | 6.510436 | 0.67849 | 15.54432 |
| TCGA-AA- | 2.716674 | 3.814735 | 42.87653 | 8.408545 | 62.12519 | 78.93497 | 13.74141 | 55.39133 |
| TCGA-A6- | 0.292417 | 0.198922 | 3.960568 | 0.824029 | 5.018929 | 9.144796 | 2.377794 | 5.544363 |
| TCGA-DM | 0.164721 | 88.58371 | 4.670011 | 0.556637 | 3.188289 | 8.166515 | 2.758635 | 5.993282 |
| TCGA-AZ- | 3.45324 | 48.31939 | 32.26484 | 7.661737 | 55.5027 | 93.74786 | 25.77132 | 177.4449 |
| TCGA-WS- | 1.538607 | 0.013124 | 23.31708 | 12.22824 | 35.94252 | 102.0129 | 21.76082 | 100.7697 |
| TCGA-AA- | 3.952449 | 3.197409 | 23.98586 | 4.768805 | 59.49885 | 79.3795 | 28.19652 | 57.56899 |
| TCGA-5M- | 0.75262 | 0.456468 | 8.374562 | 1.002032 | 7.756466 | 12.16075 | 2.140672 | 11.61169 |
| TCGA-AA- | 1.010525 | 30.58341 | 23.32893 | 1.465786 | 16.51691 | 21.558 | 3.44032 | 38.13286 |
| TCGA-A6- | 0.913525 | 1.242886 | 5.801793 | 3.765763 | 12.81215 | 13.0111 | 4.205642 | 12.31329 |
| TCGA-AZ- | 10.06461 | 6.310277 | 33.54226 | 19.61306 | 64.84529 | 95.80386 | 29.70585 | 174.2422 |
| TCGA-D5- | 0.501118 | 0.134564 | 7.725508 | 1.481161 | 12.09324 | 24.71687 | 3.44183 | 15.72702 |
| TCGA-AA- | 2.630964 | 3.337669 | 20.92176 | 5.560524 | 51.12921 | 59.80958 | 13.2988 | 63.71214 |
| TCGA-AA- | 1.843705 | 7.467635 | 12.18695 | 2.418615 | 18.2347 | 89.26304 | 9.194276 | 39.54494 |
| TCGA-AA- | 1.863844 | 5.255422 | 12.74485 | 3.572213 | 24.14783 | 77.83358 | 10.91166 | 69.25907 |
| TCGA-4N- | 0.687223 | 0.631531 | 12.98498 | 0.649777 | 3.872174 | 9.395335 | 1.733059 | 42.49214 |
| TCGA-NH- | 0.015976 | 0.014491 | 3.001403 | 0.332833 | 0.888844 | 3.598702 | 0.583901 | 2.442109 |
| TCGA-G4- | 7.195869 | 31.67034 | 22.47513 | 7.346197 | 39.27371 | 80.26744 | 21.14492 | 60.66075 |
| TCGA-CM | 1.52453 | 1.195705 | 11.15025 | 2.136322 | 14.51994 | 30.47563 | 7.005215 | 28.45078 |
| TCGA-DM | 0.140745 | 4.966376 | 6.706378 | 0.518164 | 3.645393 | 8.902717 | 1.383848 | 5.552042 |
| TCGA-F4-( | 5.581192 | 24.81192 | 62.98285 | 17.59513 | 115.3106 | 188.6232 | 58.20769 | 821.6433 |
| TCGA-AA- | 0.334153 | 7.596071 | 7.068304 | 0.630562 | 8.327083 | 9.807447 | 2.096309 | 36.38113 |
| TCGA-AA- | 1.543303 | 8.915496 | 14.91306 | 2.130129 | 28.86854 | 121.9223 | 11.93252 | 73.19557 |
| TCGA-T9-, | 0.902686 | 9.802236 | 21.83831 | 2.153282 | 7.280809 | 23.81333 | 2.553116 | 32.89094 |
| TCGA-A6- | 1.533547 | 7.4237 | 23.21984 | 6.820441 | 32.15456 | 88.02869 | 17.18117 | 52.1311 |
| TCGA-AA- | 0.564314 | 2.870795 | 6.843695 | 2.400162 | 21.16579 | 75.88795 | 7.253145 | 59.08722 |
| TCGA-AZ- | 0.646284 | 0.212577 | 11.51921 | 3.094219 | 23.96783 | 39.88288 | 4.765322 | 43.91583 |
| TCGA-CM | 0.297988 | 1.743969 | 13.02381 | 0.7926 | 9.24028 | 11.62132 | 2.674958 | 12.42771 |
| TCGA-AA- | 0.223465 | 0.672072 | 2.799894 | 0.362208 | 2.498404 | 6.202051 | 1.122965 | 24.83023 |
| TCGA-AZ- | 2.351379 | 16.77673 | 24.19049 | 0.349973 | 8.905572 | 9.472257 | 1.442056 | 15.31449 |
| TCGA-D5- | 0.883006 | 0.848463 | 9.67148 | 1.259702 | 8.426141 | 20.37143 | 5.605166 | 16.07604 |
| TCGA-G4- | 0.232237 | 0.046466 | 2.752562 | 0.395965 | 1.580526 | 5.291691 | 1.318682 | 18.28162 |
| TCGA-D5- | 4.220094 | 38.25721 | 66.78458 | 4.305328 | 37.11835 | 93.86367 | 10.25783 | 120.4957 |
| TCGA-AD- | 2.803517 | 1.399827 | 28.5298 | 3.033492 | 26.49927 | 73.32961 | 7.621888 | 60.42366 |
| TCGA-A6- | 1.041158 | 4.795741 | 10.27195 | 2.806463 | 13.12516 | 53.31061 | 8.462898 | 32.63317 |
| TCGA-A6- | 3.87722 | 6.072721 | 86.1561 | 9.401323 | 104.4016 | 115.1048 | 21.01146 | 208.8149 |
| TCGA-AA- | 0.706767 | 10.25085 | 17.24984 | 2.391605 | 21.7936 | 32.97238 | 7.380612 | 22.88398 |
| TCGA-AZ- | 12.53728 | 56.0646 | 114.0293 | 5.441239 | 108.9852 | 185.7748 | 24.05307 | 101.3686 |
| TCGA-CK- | 3.898543 | 18.23753 | 12.56601 | 3.152751 | 14.74351 | 40.50517 | 10.08804 | 32.22526 |
| TCGA-AA- | 0.39306 | 1.356032 | 2.919085 | 0.368742 | 2.769002 | 7.585315 | 0.699623 | 11.49829 |
| TCGA-AA- | 0.650262 | 11.98777 | 21.79808 | 3.092228 | 25.46817 | 41.80425 | 13.60281 | 38.49336 |
| TCGA-CM | 1.811623 | 324.7736 | 36.33529 | 0.667367 | 12.4837 | 46.25072 | 1.000424 | 53.44949 |
| TCGA-CM | 1.642538 | 8.402915 | 18.84446 | 6.49397 | 20.17601 | 67.28813 | 19.02723 | 31.44855 |
| TCGA-A6- | 9.929214 | 3.273839 | 18.88481 | 5.101625 | 47.60328 | 80.71984 | 18.06851 | 61.27809 |
| TCGA-AA- | 5.130286 | 3.429597 | 70.74741 | 3.328419 | 78.13505 | 103.3874 | 16.31614 | 316.5609 |
| TCGA-G4- | 1.026676 | 63.23178 | 12.02902 | 1.16699 | 11.30596 | 19.92931 | 4.016681 | 13.95377 |
| TCGA-AA- | 4.015889 | 137.3054 | 17.41558 | 2.852715 | 28.31075 | 43.44249 | 6.305943 | 57.0787 |
| TCGA-AA- | 11.67554 | 6.823965 | 186.8967 | 58.17063 | 233.9722 | 336.8171 | 73.85555 | 389.6192 |
| TCGA-AA- | 0.908046 | 6.995434 | 12.22739 | 2.296569 | 22.59061 | 46.30987 | 10.58539 | 18.19639 |
| TCGA-AA- | 0.575105 | 10.17188 | 6.185667 | 1.82322 | 11.31682 | 16.66104 | 2.602231 | 34.83218 |

| | | | | | | | | |
|---|---|---|---|---|---|---|---|---|
| TCGA-A6- | 0.69 | 0.306906 | 4.586072 | 0.961523 | 3.036842 | 12.64683 | 1.635886 | 6.773812 |
| TCGA-AA- | 1.325929 | 14.40677 | 17.98511 | 4.553804 | 39.98123 | 138.353 | 18.03731 | 157.5336 |
| TCGA-AA- | 1.686365 | 5.838579 | 14.87745 | 1.52748 | 23.1152 | 26.80997 | 5.95875 | 42.39169 |
| TCGA-A6- | 2.260498 | 89.57871 | 15.3608 | 3.274305 | 7.537617 | 14.62939 | 13.12484 | 99.80161 |
| TCGA-AA- | 1.016198 | 2.248993 | 10.43446 | 0.650455 | 7.246958 | 10.44393 | 1.201278 | 15.31855 |
| TCGA-AA- | 1.962084 | 19.266 | 58.49778 | 1.332916 | 42.32283 | 35.21139 | 2.376954 | 73.90076 |
| TCGA-AA- | 15.11032 | 23.83812 | 54.78122 | 5.849003 | 42.26746 | 88.22596 | 20.48395 | 95.02362 |
| TCGA-A6- | 2.166347 | 0.919889 | 28.07948 | 6.886587 | 45.82516 | 77.90095 | 8.657636 | 97.42137 |
| TCGA-AA- | 0.322718 | 0.776817 | 6.354721 | 0.415983 | 6.866354 | 13.59162 | 3.711629 | 39.5215 |
| TCGA-AA- | 1.409463 | 3.908313 | 44.66124 | 6.590036 | 53.88978 | 51.7521 | 15.99973 | 216.7651 |
| TCGA-AA- | 0.25076 | 5.085036 | 7.962875 | 1.946862 | 13.58874 | 23.7412 | 8.870368 | 8.98863 |
| TCGA-AA- | 2.362159 | 4.619852 | 9.598098 | 2.629929 | 16.27188 | 30.37186 | 6.35101 | 20.0125 |
| TCGA-AZ- | 1.225686 | 0.085687 | 18.25838 | 4.27407 | 22.31905 | 37.20391 | 6.013304 | 33.68762 |
| TCGA-D5- | 0.513721 | 0.035539 | 7.991601 | 1.23428 | 12.32256 | 18.37171 | 5.698438 | 21.75998 |
| TCGA-A6- | 1.526451 | 1.51833 | 22.88414 | 4.856642 | 42.99755 | 108.7782 | 11.36594 | 114.8714 |
| TCGA-F4-( | 1.974179 | 0.984999 | 15.66993 | 3.752439 | 40.60294 | 73.69245 | 6.027701 | 48.80798 |
| TCGA-CA- | 1.156498 | 285.1722 | 15.32284 | 1.282059 | 11.99868 | 22.38487 | 9.879494 | 26.45988 |
| TCGA-AA- | 10.27454 | 150.4112 | 95.60449 | 4.059448 | 70.7225 | 174.9834 | 31.58437 | 196.3278 |
| TCGA-A6- | 1.198184 | 232.4729 | 7.289803 | 2.324064 | 8.921849 | 13.5365 | 9.079542 | 59.65469 |
| TCGA-AA- | 0.154832 | 4.198061 | 3.727174 | 0.230402 | 1.748001 | 4.060763 | 0.516373 | 5.358671 |
| TCGA-CM- | 0.886471 | 0.542735 | 18.92625 | 3.98464 | 24.48793 | 50.4179 | 13.26569 | 31.89477 |
| TCGA-D5- | 3.890377 | 4.344484 | 46.04054 | 7.308841 | 62.21034 | 76.20005 | 30.22134 | 69.43206 |
| TCGA-G4- | 3.257304 | 5.829234 | 18.09397 | 3.489073 | 29.03573 | 33.09326 | 4.272066 | 59.50485 |
| TCGA-QL- | 4.507196 | 1.075073 | 42.18211 | 2.633897 | 23.82742 | 36.43775 | 6.606113 | 25.7981 |
| TCGA-G4- | 4.607665 | 5.343705 | 13.8719 | 3.914496 | 18.15739 | 42.88933 | 8.655404 | 32.66367 |
| TCGA-AA- | 3.398387 | 159.3447 | 11.90794 | 4.185139 | 27.06298 | 34.941 | 6.279464 | 77.1074 |
| TCGA-CK- | 5.101364 | 10.50624 | 120.6512 | 8.130702 | 133.4907 | 162.5694 | 18.17127 | 150.5251 |
| TCGA-D5- | 2.480334 | 0.073842 | 20.2759 | 7.462594 | 44.09256 | 67.78417 | 17.90384 | 127.0742 |
| TCGA-CM- | 6.95549 | 66.71507 | 75.24637 | 1.967307 | 56.67975 | 56.03826 | 6.691272 | 434.2556 |
| TCGA-AA- | 2.720721 | 1.575949 | 28.9504 | 10.26744 | 71.49801 | 71.9933 | 19.40318 | 74.32029 |
| TCGA-CM- | 1.178582 | 92.10573 | 16.53808 | 2.186156 | 14.98802 | 34.68554 | 9.321839 | 28.07169 |
| TCGA-AA- | 0.514847 | 16.419 | 12.72898 | 1.622861 | 15.11606 | 21.45128 | 3.532853 | 20.90716 |
| TCGA-A6- | 1.027386 | 0.990977 | 15.78926 | 4.708501 | 39.34044 | 47.18888 | 9.57697 | 58.33352 |
| TCGA-CM- | 1.76627 | 0.558196 | 22.89674 | 6.040404 | 46.07807 | 77.89299 | 20.0279 | 28.05792 |
| TCGA-F4-( | 1.139151 | 16.73363 | 26.91696 | 2.402212 | 25.20115 | 27.98423 | 4.95833 | 51.84858 |
| TCGA-AY- | 1.513445 | 207.5882 | 9.254217 | 2.072469 | 11.53847 | 26.77336 | 5.523434 | 19.22991 |
| TCGA-A6- | 3.036753 | 0.509474 | 17.91287 | 5.138862 | 30.79941 | 47.06707 | 11.72775 | 36.11353 |
| TCGA-AA- | 0.929111 | 25.24465 | 9.914879 | 1.806906 | 17.41475 | 19.07122 | 10.82369 | 6.856812 |
| TCGA-AA- | 3.273365 | 2.332807 | 8.956466 | 1.308346 | 18.17863 | 19.76784 | 7.0053 | 11.86843 |
| TCGA-NH- | 0.109387 | 0.024804 | 3.730056 | 0.308252 | 1.1334 | 3.283722 | 0.902234 | 3.470346 |
| TCGA-CK- | 0.227452 | 0.07986 | 7.449699 | 1.149734 | 10.54374 | 13.68984 | 2.297954 | 14.02992 |
| TCGA-AA- | 0.887641 | 0.40875 | 9.419922 | 0.358712 | 4.947055 | 7.005598 | 1.94148 | 35.17141 |
| TCGA-A6- | 0.434174 | 0.045439 | 4.923708 | 0.707882 | 4.218757 | 7.992663 | 1.934326 | 28.95505 |
| TCGA-D5- | 1.482315 | 0.112488 | 12.87326 | 3.471646 | 19.77347 | 41.78414 | 12.56312 | 27.83128 |
| TCGA-AA- | 2.887912 | 1.562179 | 17.0509 | 0.648327 | 21.47588 | 31.56354 | 4.091387 | 30.865 |
| TCGA-AA- | 0.854428 | 1.056803 | 8.287703 | 1.364925 | 15.48862 | 26.82141 | 1.646281 | 33.64862 |
| TCGA-G4- | 1.787442 | 29.6051 | 19.3447 | 6.400671 | 23.23319 | 28.02651 | 11.61418 | 22.5868 |
| TCGA-AA- | 0.628335 | 8.225788 | 9.050974 | 0.885321 | 8.730579 | 16.0533 | 3.23773 | 30.68654 |
| TCGA-DM | 1.827793 | 0.362087 | 6.214623 | 0.604925 | 8.508023 | 9.205628 | 1.717564 | 12.86036 |
| TCGA-AA- | 0.67215 | 1.544134 | 8.548939 | 1.117837 | 12.74144 | 17.89073 | 2.555045 | 24.85179 |
| TCGA-F4-( | 1.431758 | 14.39667 | 24.48963 | 4.505244 | 38.25354 | 54.57668 | 9.091684 | 49.20836 |
| TCGA-AA- | 0.560066 | 17.54074 | 10.11072 | 1.143906 | 16.09349 | 22.33443 | 3.906053 | 21.15726 |
| TCGA-AA- | 7.724818 | 0.159847 | 59.45546 | 7.329564 | 46.89732 | 127.9819 | 16.22559 | 133.0832 |
| TCGA-AM | 0.529146 | 1.087712 | 7.283278 | 0.984631 | 8.643413 | 14.38601 | 0.971591 | 10.88193 |
| TCGA-5M- | 0.344601 | 3.622883 | 5.633784 | 0.510765 | 5.169316 | 7.741866 | 1.558018 | 7.553444 |
| TCGA-AA- | 0.669852 | 5.802942 | 5.940234 | 1.423982 | 12.04796 | 13.22503 | 3.24161 | 15.96806 |
| TCGA-AA- | 7.305279 | 3.536032 | 117.1048 | 27.10423 | 107.581 | 357.3778 | 23.40006 | 473.8578 |
| TCGA-CK- | 2.841324 | 3.203895 | 34.78584 | 4.195905 | 28.33606 | 83.98063 | 11.14758 | 51.43235 |

| | | | | | | | | |
|---|---|---|---|---|---|---|---|---|
| TCGA-4T-. | 0.433062 | 19.26802 | 8.866518 | 0.509104 | 1.823328 | 7.054312 | 2.682779 | 6.935969 |
| TCGA-AA- | 1.650923 | 8.499617 | 17.86448 | 2.056144 | 21.77634 | 29.50775 | 2.903393 | 55.08156 |
| TCGA-F4-( | 1.389676 | 0.132272 | 12.67109 | 4.361575 | 22.47793 | 31.56427 | 7.42244 | 73.2986 |
| TCGA-AA- | 0.645625 | 113.9494 | 13.5052 | 0.564631 | 8.290214 | 18.8997 | 3.351999 | 25.61943 |
| TCGA-D5- | 2.099101 | 14.9103 | 25.246 | 5.925616 | 42.60541 | 58.60276 | 17.77487 | 195.7732 |
| TCGA-FM- | 3.52175 | 0 | 97.91101 | 8.576068 | 38.38493 | 163.4912 | 32.34091 | 139.4159 |
| TCGA-CM | 4.925866 | 70.41207 | 50.67986 | 3.010784 | 50.92364 | 44.66488 | 12.74381 | 151.6715 |
| TCGA-G4- | 1.876155 | 15.08728 | 14.21022 | 3.651997 | 22.70559 | 32.36349 | 8.41181 | 95.96584 |
| TCGA-G4- | 2.830394 | 9.888021 | 25.76847 | 1.017219 | 14.24618 | 30.50472 | 2.342253 | 129.0842 |
| TCGA-G4- | 4.45094 | 886.4707 | 6.828173 | 1.935927 | 10.27033 | 17.29409 | 4.662649 | 29.72261 |
| TCGA-AA- | 7.586508 | 1.822486 | 22.45975 | 3.866764 | 41.58412 | 39.75632 | 14.49477 | 13.35935 |
| TCGA-AZ- | 0.534878 | 3.693193 | 8.150085 | 0.981079 | 13.27281 | 35.04804 | 1.908111 | 37.02376 |
| TCGA-G4- | 0.531206 | 8.938979 | 11.85802 | 2.94392 | 13.31866 | 22.96103 | 5.756896 | 67.85432 |
| TCGA-AA- | 0.528805 | 1.445838 | 14.0442 | 0.741502 | 13.86468 | 12.86353 | 4.857492 | 14.72348 |
| TCGA-AA- | 2.326785 | 8.705625 | 21.02205 | 6.041676 | 29.42079 | 61.55081 | 11.94216 | 74.60109 |
| TCGA-AY- | 0.423056 | 51.65494 | 1.750103 | 0.240726 | 1.464195 | 3.756985 | 0.625987 | 2.424649 |
| TCGA-AA- | 0.546981 | 5.040193 | 7.523339 | 1.677761 | 6.358235 | 13.90395 | 2.471718 | 16.45686 |
| TCGA-CM | 4.424449 | 4.156763 | 15.92432 | 4.581492 | 19.33955 | 34.48102 | 8.876058 | 40.69788 |
| TCGA-AA- | 0.870598 | 5.732051 | 12.15079 | 2.689596 | 20.61087 | 25.80863 | 5.260526 | 69.05018 |
| TCGA-D5- | 1.722594 | 0.429216 | 13.68037 | 4.96664 | 23.95295 | 43.50817 | 8.582493 | 49.62404 |
| TCGA-AA- | 3.470376 | 1.967326 | 45.38061 | 15.80086 | 47.36569 | 79.24077 | 12.13131 | 224.4019 |
| TCGA-A6- | 0.123668 | 0.056085 | 4.926448 | 0.597422 | 5.71623 | 21.77365 | 3.597128 | 6.6183 |
| TCGA-CM | 0.907103 | 0.860508 | 9.225461 | 0.904556 | 7.486922 | 15.6792 | 4.409521 | 30.21832 |
| TCGA-CA- | 5.775351 | 4.038743 | 84.96423 | 28.20073 | 209.3975 | 206.8914 | 63.08092 | 587.9618 |
| TCGA-AA- | 2.765337 | 1.987656 | 17.04154 | 2.599315 | 25.83692 | 29.24627 | 11.44164 | 24.30296 |
| TCGA-F4-( | 9.209263 | 64.95058 | 88.83648 | 37.39262 | 117.9048 | 151.518 | 46.33091 | 457.2185 |
| TCGA-AA- | 0.872656 | 2.426185 | 8.134568 | 1.563689 | 8.047772 | 29.9126 | 2.388559 | 37.36974 |
| TCGA-AD- | 2.820378 | 0.29716 | 36.30691 | 7.053241 | 58.96834 | 89.2592 | 21.7109 | 99.66606 |
| TCGA-DM | 0.377563 | 0.052536 | 23.44285 | 1.908151 | 24.49962 | 21.12219 | 2.435422 | 43.24827 |
| TCGA-DM | 1.96742 | 1.396566 | 14.32349 | 0.391093 | 6.296468 | 9.058638 | 1.617654 | 7.812427 |
| TCGA-AU- | 2.008085 | 6.878064 | 97.33839 | 5.486083 | 153.1812 | 132.7814 | 17.84651 | 201.0347 |
| TCGA-A6- | 1.949443 | 2.946993 | 31.42562 | 17.18358 | 45.45329 | 73.70348 | 5.255567 | 110.2103 |
| TCGA-AA- | 0.461951 | 2.424225 | 8.88042 | 1.205469 | 8.970253 | 19.36193 | 3.531327 | 15.16314 |
| TCGA-G4- | 9.247399 | 27.5489 | 12.32681 | 1.055508 | 5.649443 | 20.54322 | 3.674045 | 13.67641 |
| TCGA-DM | 3.251747 | 0.540552 | 14.10059 | 1.282269 | 6.682022 | 18.11592 | 3.092091 | 14.03986 |
| TCGA-D5- | 3.982379 | 45.35968 | 55.31715 | 23.31821 | 134.5241 | 277.9515 | 78.97926 | 491.3019 |
| TCGA-DM | 1.402894 | 4.810796 | 3.423733 | 0.535039 | 3.734473 | 7.354577 | 0.746737 | 4.60217 |
| TCGA-A6- | 4.535208 | 77.39354 | 12.08757 | 1.414016 | 13.07621 | 16.99203 | 6.794571 | 84.74491 |
| TCGA-D5- | 0.709852 | 0.097464 | 8.797723 | 1.574985 | 16.51025 | 19.74009 | 6.809075 | 19.79685 |
| TCGA-AA- | 0.595286 | 9.387587 | 12.80545 | 3.651851 | 16.88449 | 32.9412 | 5.20965 | 27.23595 |
| TCGA-A6- | 2.440304 | 340.0752 | 13.17256 | 3.303984 | 25.61008 | 34.42901 | 10.76098 | 20.43769 |
| TCGA-AD- | 1.372107 | 1.003543 | 9.599811 | 1.178175 | 8.263562 | 21.45915 | 3.869587 | 11.46025 |
| TCGA-AA- | 0.721964 | 24.74428 | 6.029481 | 1.23095 | 9.907429 | 19.89264 | 4.578445 | 11.29919 |
| TCGA-A6- | 2.406233 | 9.070856 | 13.71136 | 6.143941 | 10.35574 | 46.74024 | 6.718517 | 60.46458 |
| TCGA-AA- | 3.610325 | 3.560188 | 85.67652 | 23.86244 | 88.41497 | 129.496 | 19.27572 | 378.2596 |
| TCGA-A6- | 0.321805 | 0.187641 | 4.87871 | 3.38956 | 18.22636 | 20.55513 | 4.659527 | 13.94842 |
| TCGA-CK- | 0.187769 | 0.061664 | 2.674212 | 0.404667 | 3.729165 | 4.982389 | 1.314546 | 4.295041 |
| TCGA-G4- | 1.006255 | 0.560066 | 50.47454 | 2.744702 | 17.74059 | 52.02036 | 3.269352 | 26.21844 |
| TCGA-AA- | 0.790773 | 11.23694 | 11.13883 | 1.671292 | 19.57552 | 19.97462 | 4.655542 | 8.159798 |
| TCGA-AA- | 2.047047 | 63.80382 | 37.0961 | 4.262205 | 41.80046 | 44.8262 | 10.09754 | 209.935 |
| TCGA-A6- | 4.960387 | 7.626694 | 15.90455 | 1.059333 | 22.00338 | 83.80472 | 6.114319 | 117.3589 |
| TCGA-F4-( | 2.605656 | 0.737226 | 10.91297 | 1.754114 | 13.80734 | 24.13523 | 5.685381 | 64.02056 |
| TCGA-AA- | 2.683588 | 2.615225 | 61.54058 | 2.803839 | 53.0923 | 73.31204 | 10.44426 | 275.4292 |
| TCGA-AA- | 0.578065 | 0.407805 | 10.42392 | 0.824187 | 6.997963 | 15.66659 | 2.476631 | 5.248634 |
| TCGA-F4-( | 7.378768 | 7.955173 | 28.02093 | 10.49584 | 56.03478 | 70.40852 | 24.00511 | 49.82283 |
| TCGA-AA- | 2.730978 | 18.15273 | 14.22675 | 3.263459 | 27.00119 | 34.42318 | 7.310267 | 31.31615 |
| TCGA-D5- | 1.450789 | 10.53298 | 11.12969 | 3.907868 | 19.16945 | 34.66171 | 3.832109 | 33.45581 |
| TCGA-AA- | 4.386051 | 25.59392 | 25.40514 | 7.390972 | 52.51497 | 81.41632 | 23.02477 | 35.23908 |

| | | | | | | | | |
|---|---|---|---|---|---|---|---|---|
| TCGA-QG- | 1.075237 | 0.661614 | 6.470173 | 1.228426 | 6.480173 | 14.59803 | 3.344821 | 15.24068 |
| TCGA-A6- | 0.177568 | 1.379387 | 12.13279 | 0.959837 | 10.54898 | 14.95345 | 4.756883 | 67.73297 |
| TCGA-D5- | 2.269178 | 42.76201 | 10.83745 | 2.258091 | 15.36293 | 24.69366 | 3.983612 | 22.87607 |
| TCGA-F4-( | 0.90479 | 1.471649 | 13.06337 | 5.656697 | 20.91982 | 38.36273 | 13.56544 | 22.99525 |
| TCGA-AA- | 0.716845 | 18.97587 | 7.486704 | 0.903382 | 8.926145 | 20.12817 | 2.934957 | 24.76473 |
| TCGA-A6- | 1.182951 | 93.36334 | 41.48338 | 3.446984 | 25.28698 | 27.07066 | 5.755116 | 143.5201 |
| TCGA-AA- | 0.664979 | 2.843438 | 27.82597 | 0.946513 | 13.86612 | 15.83234 | 1.389199 | 55.44741 |
| TCGA-AA- | 0.549492 | 39.41372 | 9.834536 | 0.637081 | 5.155408 | 10.93225 | 1.037775 | 26.94166 |
| TCGA-AY- | 1.885047 | 0.568493 | 53.27007 | 3.698742 | 51.7862 | 69.12536 | 14.88364 | 315.3021 |
| TCGA-AA- | 1.181694 | 1.021742 | 14.08788 | 2.235748 | 21.3619 | 23.36683 | 5.446141 | 135.5299 |
| TCGA-AA- | 4.886527 | 6.838682 | 15.13458 | 3.729065 | 19.95495 | 32.0786 | 10.65789 | 100.8886 |
| TCGA-AA- | 1.966019 | 6.153894 | 19.82836 | 3.398624 | 37.4268 | 49.72447 | 10.56343 | 76.97455 |
| TCGA-AA- | 3.772853 | 87.26293 | 86.60785 | 3.965119 | 109.6644 | 130.4614 | 13.06884 | 457.7386 |
| TCGA-AY- | 10.29191 | 14.86507 | 71.99092 | 25.27284 | 139.5068 | 219.9008 | 46.82798 | 456.0828 |
| TCGA-AD- | 0.216586 | 9.345366 | 21.95535 | 0.896822 | 7.476377 | 17.38757 | 2.330915 | 21.5062 |
| TCGA-AZ- | 0.216677 | 95.16793 | 10.7126 | 0.490655 | 4.23291 | 7.440977 | 1.869899 | 8.170873 |
| TCGA-F4-( | 2.489328 | 113.7813 | 13.57143 | 1.449508 | 6.513795 | 19.88457 | 4.679604 | 68.40505 |
| TCGA-AA- | 1.801047 | 414.9969 | 23.97345 | 6.858594 | 53.26834 | 78.44323 | 25.0586 | 78.39228 |
| TCGA-QG- | 5.7764 | 91.87628 | 44.38775 | 5.070256 | 24.94214 | 44.08551 | 14.6855 | 150.6036 |
| TCGA-AA- | 0.477605 | 480.4778 | 7.471977 | 0.865212 | 3.385514 | 13.73749 | 2.47521 | 28.63474 |
| TCGA-AA- | 0.442347 | 15.92114 | 7.247171 | 1.384133 | 13.70336 | 19.98386 | 4.156005 | 15.96663 |
| TCGA-NH- | 0.971104 | 2.5443 | 22.29841 | 2.116087 | 10.80905 | 38.18542 | 10.2182 | 32.63316 |
| TCGA-CM- | 0.532615 | 1.074472 | 10.8252 | 1.197767 | 8.669831 | 13.04873 | 1.865994 | 16.72087 |
| TCGA-AA- | 0.846255 | 1.809284 | 7.031236 | 1.630382 | 12.62815 | 22.07217 | 5.059699 | 7.709531 |
| TCGA-AA- | 1.550179 | 0.283574 | 9.602326 | 1.923298 | 15.12084 | 23.22007 | 4.073062 | 20.21304 |
| TCGA-CA- | 1.858403 | 0.238395 | 18.36654 | 1.923781 | 14.19974 | 29.3339 | 6.100362 | 38.92796 |
| TCGA-AA- | 3.154964 | 2.497015 | 21.92286 | 2.973225 | 29.16929 | 50.11778 | 10.88784 | 89.01018 |
| TCGA-CK- | 3.120062 | 0.137401 | 19.46051 | 1.463601 | 13.12915 | 21.70332 | 7.138465 | 42.72445 |
| TCGA-A6- | 0.513295 | 21.84987 | 43.49516 | 1.359931 | 47.26565 | 27.46008 | 5.617888 | 66.4333 |
| TCGA-A6- | 1.272429 | 0.021373 | 15.85987 | 4.667094 | 26.18949 | 46.08239 | 15.94665 | 156.0818 |
| TCGA-G4- | 0.007025 | 0.611735 | 4.392757 | 0.655441 | 2.45177 | 7.098379 | 1.592194 | 43.28008 |
| TCGA-NH- | 0.191325 | 0.08181 | 5.221035 | 0.539703 | 5.201262 | 7.131763 | 1.167181 | 45.23234 |
| TCGA-DM | 0.062626 | 0.665663 | 4.052743 | 0.244628 | 1.561461 | 3.282045 | 0.907602 | 1.365945 |
| TCGA-NH- | 0.17567 | 4.176904 | 7.707772 | 1.136561 | 4.963407 | 15.4828 | 1.95947 | 6.309043 |
| TCGA-AD- | 0.84356 | 61.42184 | 6.962388 | 1.079333 | 2.700143 | 12.43518 | 3.215403 | 12.573 |
| TCGA-A6- | 1.547518 | 1.555758 | 41.04047 | 12.13899 | 61.25668 | 138.951 | 64.29208 | 64.09035 |
| TCGA-D5- | 1.314228 | 31.33624 | 8.675621 | 1.778659 | 13.15831 | 19.08931 | 4.48821 | 26.13459 |
| TCGA-F4-( | 0.274787 | 1.183885 | 8.220756 | 0.138276 | 1.72278 | 3.239934 | 0.66072 | 3.148067 |
| TCGA-AA- | 0.637665 | 53.4754 | 6.673704 | 0.639031 | 7.453881 | 16.42868 | 1.743815 | 10.87812 |
| TCGA-FF-8 | 4.869524 | 0.026643 | 353.8021 | 30.69774 | 86.68805 | 393.4997 | 100.2329 | 469.7695 |
| TCGA-AG- | 2.90051 | 2.104671 | 17.86652 | 3.540508 | 42.08043 | 47.74685 | 13.25283 | 45.69124 |
| TCGA-AG- | 1.224522 | 3.870003 | 8.926753 | 1.709941 | 15.78172 | 18.05303 | 4.068541 | 77.47617 |
| TCGA-A6- | 0.755755 | 0.092081 | 5.45148 | 2.278427 | 13.00766 | 13.14481 | 5.106892 | 17.04715 |
| TCGA-QG- | 0.867516 | 1.662839 | 17.92606 | 3.234293 | 27.19276 | 34.62891 | 10.86483 | 100.0534 |
| TCGA-AA- | 1.803925 | 41.35283 | 21.72106 | 4.023252 | 33.99914 | 39.39758 | 12.93662 | 81.32978 |
| TCGA-AA- | 1.243488 | 1.804602 | 7.01424 | 1.276507 | 10.56072 | 21.74956 | 2.174503 | 21.75735 |
| TCGA-AA- | 1.513138 | 13.10695 | 28.51722 | 3.186584 | 39.10248 | 38.33621 | 9.466219 | 168.999 |
| TCGA-A6- | 6.214064 | 22.74297 | 17.82951 | 7.1274 | 28.5186 | 50.34126 | 14.96746 | 22.42514 |
| TCGA-AA- | 2.147713 | 2.798363 | 74.27917 | 13.77192 | 77.26681 | 121.3554 | 27.50031 | 479.2207 |
| TCGA-AA- | 5.933415 | 0.067724 | 46.00761 | 15.53702 | 85.98231 | 112.74 | 28.56054 | 118.5688 |
| TCGA-AA- | 0.250321 | 0.885485 | 4.906115 | 0.408125 | 8.018393 | 21.57645 | 3.18674 | 13.47653 |
| TCGA-CM- | 1.687365 | 8.026898 | 28.98663 | 6.774759 | 39.69804 | 65.18605 | 5.47452 | 78.77982 |
| TCGA-AA- | 1.441847 | 1.471267 | 8.036018 | 1.810591 | 12.07823 | 15.95744 | 5.810626 | 11.15226 |
| TCGA-A6- | 7.186009 | 0.247934 | 32.7021 | 7.19122 | 82.1874 | 94.60097 | 19.84365 | 132.9016 |
| TCGA-DM | 0.25601 | 0.348311 | 5.999105 | 0.514794 | 3.376281 | 5.786699 | 0.701078 | 5.582031 |
| TCGA-AD- | 4.646232 | 31.70969 | 23.31198 | 2.047213 | 18.45652 | 37.50254 | 6.321589 | 83.47058 |
| TCGA-AA- | 2.938569 | 2.119765 | 18.21435 | 1.357648 | 20.57319 | 28.14396 | 3.590201 | 28.099 |
| TCGA-CM- | 1.52586 | 8.066417 | 14.83859 | 2.475398 | 19.60554 | 43.49069 | 9.402737 | 43.26333 |

| | | | | | | | | |
|---|---|---|---|---|---|---|---|---|
| TCGA-D5- | 1.42352 | 100.5352 | 55.55568 | 10.72546 | 93.74494 | 79.68292 | 8.985341 | 598.1877 |
| TCGA-D5- | 33.89856 | 61.47265 | 138.6177 | 25.82084 | 246.9642 | 323.3357 | 114.6783 | 806.8858 |
| TCGA-F4-( | 1.574698 | 3.195951 | 9.434335 | 1.667985 | 8.700823 | 22.18924 | 5.77593 | 41.83177 |
| TCGA-G4- | 0.215829 | 0.427587 | 6.619381 | 0.528178 | 2.647638 | 9.248969 | 1.706351 | 4.029732 |
| TCGA-AY- | 3.917424 | 1.663443 | 22.56131 | 4.079455 | 34.27905 | 46.05206 | 11.76794 | 42.63867 |
| TCGA-AA- | 2.4067 | 39.39911 | 13.73113 | 2.179947 | 23.04552 | 33.5552 | 4.968455 | 34.14334 |
| TCGA-DM | 0.021586 | 77.1497 | 3.848833 | 0.293277 | 2.047022 | 4.412532 | 1.075787 | 9.545839 |
| TCGA-A6- | 1.515943 | 20.75758 | 13.02852 | 2.157752 | 17.6758 | 52.67284 | 4.805791 | 43.81541 |
| TCGA-CM | 0.60819 | 0.267942 | 5.972518 | 1.306392 | 11.81361 | 18.30396 | 4.056685 | 7.885089 |
| TCGA-AA- | 0.223449 | 1.097817 | 20.95367 | 0.202396 | 5.226943 | 5.075281 | 1.068044 | 16.46939 |
| TCGA-DX- | 1.076659 | 0.01878 | 15.59869 | 1.169013 | 11.34912 | 20.05732 | 0.930522 | 11.20667 |
| TCGA-A6- | 3.283343 | 0.794154 | 43.31939 | 19.16134 | 90.52925 | 111.3413 | 25.52598 | 49.59253 |
| TCGA-AA- | 1.0837 | 13.65136 | 13.71291 | 2.459765 | 28.96918 | 41.43427 | 11.21664 | 40.96279 |
| TCGA-AZ- | 1.211169 | 7.371544 | 11.72814 | 3.330001 | 14.43358 | 31.1491 | 6.545141 | 18.19228 |
| TCGA-D5- | 1.709303 | 0.052615 | 12.80002 | 2.19278 | 15.00693 | 31.07761 | 8.833338 | 60.11735 |
| TCGA-AA- | 6.483311 | 15.75143 | 19.1896 | 6.225702 | 35.82666 | 56.34573 | 16.25816 | 29.73514 |
| TCGA-A6- | 0.826885 | 0.063381 | 8.568057 | 1.751131 | 9.343648 | 19.36434 | 6.928504 | 10.96691 |
| TCGA-AA- | 2.331735 | 27.12963 | 17.91887 | 3.452101 | 26.08771 | 38.9029 | 6.3019 | 107.2293 |
| TCGA-AA- | 0.806058 | 0.652076 | 11.3097 | 2.052209 | 13.92463 | 35.96737 | 5.508725 | 99.46255 |
| TCGA-CM | 1.313794 | 0.031637 | 35.1978 | 4.623145 | 41.71411 | 80.24757 | 15.32785 | 54.38939 |
| TCGA-NH- | 0.613875 | 0.281637 | 9.141363 | 1.875008 | 12.29747 | 18.57886 | 4.209081 | 57.02632 |
| TCGA-A6- | 2.064594 | 0.243443 | 7.658672 | 3.55314 | 17.49072 | 16.86391 | 7.17744 | 8.614252 |
| TCGA-DM | 0.249408 | 2.827749 | 2.796825 | 0.11923 | 1.511109 | 2.742252 | 0.517921 | 1.788323 |
| TCGA-AZ- | 0.953222 | 74.45242 | 5.012379 | 0.393528 | 1.992422 | 4.280651 | 0.472859 | 8.920919 |
| TCGA-AA- | 0.799432 | 11.96425 | 12.0278 | 2.921855 | 30.27182 | 42.74972 | 7.320987 | 40.20755 |
| TCGA-D5- | 2.668609 | 180.3749 | 39.91313 | 1.003887 | 17.66612 | 18.53411 | 6.158654 | 164.861 |
| TCGA-AZ- | 2.206559 | 107.6675 | 11.4737 | 1.611167 | 11.05111 | 26.97133 | 4.791798 | 24.09227 |
| TCGA-A6- | 0.538273 | 0.811087 | 11.0561 | 3.213688 | 22.74932 | 37.31104 | 10.22356 | 20.94968 |
| TCGA-AY- | 1.102901 | 13.54164 | 46.07768 | 0.847182 | 41.21449 | 28.53746 | 1.525909 | 74.79811 |
| TCGA-AZ- | 0.298671 | 10.97155 | 7.111947 | 1.177937 | 11.58525 | 20.8052 | 6.939926 | 11.82638 |
| TCGA-AA- | 2.339311 | 13.88825 | 13.87666 | 1.692374 | 14.63593 | 31.64552 | 4.438764 | 25.84839 |
| TCGA-CK- | 4.375346 | 5.984037 | 12.98519 | 3.885201 | 34.95249 | 51.3317 | 18.12227 | 26.06504 |
| TCGA-CM | 5.015893 | 51.35417 | 39.9169 | 11.52462 | 92.35875 | 118.3708 | 27.87364 | 102.3908 |
| TCGA-AA- | 0.173248 | 3.457097 | 3.436958 | 0.418468 | 1.916793 | 4.361149 | 0.454442 | 7.370135 |
| TCGA-CA- | 3.647983 | 42.63344 | 27.67084 | 8.428072 | 33.2081 | 98.52012 | 48.26358 | 79.23598 |
| TCGA-AY- | 1.953975 | 3.732407 | 12.04385 | 4.371948 | 12.50068 | 26.80633 | 7.236675 | 11.49495 |
| TCGA-A6- | 3.725658 | 3.724576 | 30.69905 | 6.19122 | 76.56082 | 114.8263 | 35.69688 | 148.5563 |
| TCGA-D5- | 2.114706 | 108.0826 | 14.8255 | 3.847779 | 25.51592 | 38.21705 | 17.55526 | 37.9538 |
| TCGA-AA- | 2.306703 | 123.263 | 35.65732 | 1.95085 | 41.89717 | 50.8757 | 8.424098 | 99.9394 |
| TCGA-AG- | 0.666198 | 1.858096 | 13.9726 | 1.946063 | 13.35152 | 25.11174 | 5.128862 | 74.50224 |
| TCGA-5M- | 0.87511 | 0.146895 | 10.96895 | 0.648539 | 3.535127 | 6.059694 | 2.045058 | 4.621737 |
| TCGA-CK- | 1.414223 | 21.44004 | 14.93937 | 1.641254 | 10.35001 | 23.09122 | 3.653299 | 56.32021 |
| TCGA-CA- | 0.346898 | 0.129962 | 5.739101 | 0.27323 | 1.557459 | 6.14017 | 0.366612 | 7.68498 |
| TCGA-A6- | 1.815338 | 0.151524 | 19.42146 | 6.103083 | 35.72634 | 44.99629 | 8.495208 | 62.92435 |
| TCGA-AY- | 0.441796 | 2.185746 | 6.822157 | 1.68961 | 5.450815 | 15.14789 | 4.516044 | 9.048068 |
| TCGA-AG- | 0.579366 | 0.797009 | 4.348204 | 0.98542 | 6.263506 | 13.53656 | 3.458203 | 9.914399 |
| TCGA-AA- | 1.947497 | 9.241963 | 19.34919 | 3.104655 | 25.88572 | 32.45725 | 10.88706 | 32.75734 |
| TCGA-D5- | 1.785572 | 0.272254 | 14.72392 | 4.02476 | 27.50171 | 38.01997 | 12.76168 | 26.69618 |
| TCGA-A6- | 9.762419 | 21.08785 | 38.90509 | 8.246206 | 81.13369 | 106.4674 | 36.06677 | 69.30848 |
| TCGA-RU- | 0.229461 | 0.180742 | 2.372762 | 0.098451 | 0.912413 | 2.665325 | 0.598724 | 1.689318 |
| TCGA-A6- | 1.276889 | 2.256179 | 4.161778 | 3.499794 | 19.72057 | 16.86315 | 14.81552 | 19.13097 |
| TCGA-AA- | 1.836125 | 3.737188 | 11.41747 | 3.898378 | 26.11414 | 40.79513 | 8.522896 | 27.70668 |
| TCGA-D5- | 1.334253 | 0.057629 | 6.276917 | 0.575497 | 6.025295 | 14.45873 | 2.892458 | 12.33853 |
| TCGA-D5- | 0.483338 | 0.030946 | 5.798027 | 0.607769 | 3.559063 | 6.537596 | 1.709333 | 49.97113 |
| TCGA-AA- | 0.65992 | 0.014029 | 7.718986 | 0.523026 | 3.89509 | 9.189883 | 0.976401 | 7.806478 |
| TCGA-CM | 1.244698 | 35.19283 | 236.2788 | 31.64568 | 187.9163 | 194.6922 | 13.07473 | 181.8247 |
| TCGA-AA- | 1.189532 | 2.589449 | 12.00336 | 1.861064 | 21.63842 | 23.40129 | 3.937158 | 30.15952 |
| TCGA-AA- | 1.411266 | 1.779159 | 19.96039 | 2.064044 | 24.9497 | 32.78891 | 6.058803 | 156.9107 |

| | | | | | | | | |
|---|---|---|---|---|---|---|---|---|
| TCGA-AA- | 3.025165 | 2.00724 | 59.07033 | 6.222687 | 66.31812 | 86.44221 | 17.21512 | 468.1393 |
| TCGA-AA- | 1.858848 | 84.71042 | 11.38774 | 2.533742 | 25.79737 | 34.56563 | 6.106694 | 31.80258 |
| TCGA-G4- | 1.004545 | 0.070252 | 9.565951 | 2.653166 | 15.69037 | 24.88181 | 5.314311 | 17.45111 |
| TCGA-A6- | 0.40916 | 0.01887 | 3.490816 | 1.72114 | 8.176897 | 10.65488 | 3.365294 | 30.60183 |
| TCGA-G4- | 1.352316 | 164.8877 | 10.5325 | 1.055719 | 7.826911 | 13.40522 | 2.23808 | 15.49327 |
| TCGA-G4- | 1.822762 | 84.03735 | 35.04117 | 5.398976 | 39.91715 | 76.561 | 13.5759 | 68.57224 |
| TCGA-CA- | 0.722066 | 456.4613 | 7.948252 | 0.483161 | 6.465088 | 8.088765 | 1.805299 | 15.25312 |
| TCGA-AA- | 1.712979 | 5.44312 | 15.38593 | 2.939849 | 30.12393 | 38.28315 | 10.14532 | 160.2396 |
| TCGA-NH- | 0.844906 | 17.91974 | 27.05322 | 2.581997 | 15.22124 | 38.15404 | 4.780464 | 157.6415 |
| TCGA-AA- | 0.486819 | 5.693274 | 9.046181 | 1.313475 | 12.12116 | 15.54977 | 3.108175 | 16.04199 |
| TCGA-AA- | 0.206103 | 0.420618 | 6.059229 | 0.361702 | 2.59791 | 4.17628 | 0.486561 | 2.712079 |
| TCGA-CM | 0.235625 | 7.243685 | 4.953607 | 0.553998 | 4.129872 | 10.63335 | 1.833144 | 6.304126 |
| TCGA-AD- | 0.475022 | 0.201964 | 6.563908 | 0.654364 | 4.454722 | 9.18084 | 0.693096 | 8.163058 |
| TCGA-F4-( | 0.594107 | 6.881821 | 18.75597 | 4.439584 | 19.9958 | 58.24201 | 10.40653 | 53.7604 |
| TCGA-AZ- | 2.067434 | 3.159019 | 21.2743 | 5.306791 | 34.36597 | 54.35955 | 23.12043 | 37.9047 |
| TCGA-A6- | 1.361363 | 4.376406 | 26.19225 | 5.183379 | 39.21809 | 121.4904 | 14.44812 | 145.0662 |
| TCGA-AA- | 0.948363 | 7.019153 | 8.722418 | 1.477499 | 12.27928 | 18.86788 | 3.78782 | 43.10676 |
| TCGA-A6- | 1.255697 | 0.412378 | 11.29116 | 6.360223 | 28.47461 | 45.78856 | 6.273324 | 67.0202 |
| TCGA-SS-/ | 0.495669 | 0.180357 | 2.421805 | 0.339337 | 3.253965 | 6.108615 | 1.236677 | 3.424324 |
| TCGA-CM | 1.453449 | 31.5714 | 2.102355 | 0.204286 | 1.4338 | 9.919058 | 1.827369 | 4.191931 |
| TCGA-AZ- | 1.007222 | 93.81196 | 9.033957 | 0.154631 | 8.224253 | 18.60475 | 1.209057 | 20.18699 |
| TCGA-AA- | 2.815907 | 3.962612 | 30.94036 | 3.963432 | 48.87022 | 58.30342 | 12.48433 | 58.00455 |
| TCGA-AA- | 1.615805 | 2.913497 | 9.338675 | 1.322501 | 12.64622 | 16.90681 | 3.666444 | 18.66882 |
| TCGA-G4- | 1.52831 | 0.122313 | 3.620675 | 0.095002 | 0.653625 | 1.599743 | 0.224025 | 10.95478 |
| TCGA-G4- | 0.848667 | 10.00692 | 5.480256 | 0.885969 | 5.641295 | 14.93936 | 4.801865 | 32.96741 |
| TCGA-AA- | 8.571174 | 11.58191 | 35.11569 | 7.834199 | 62.90812 | 97.39663 | 16.78033 | 68.93608 |
| TCGA-G4- | 5.702215 | 1.610969 | 45.15961 | 9.663852 | 80.34346 | 104.1706 | 32.98454 | 292.0363 |
| TCGA-A6- | 5.397561 | 3.490473 | 23.55634 | 4.502724 | 43.77198 | 49.74158 | 20.75484 | 52.52505 |
| TCGA-AY- | 0.323695 | 5.979277 | 5.66872 | 1.251728 | 3.84893 | 15.56178 | 2.220658 | 9.317828 |
| TCGA-A6- | 4.044721 | 4.37418 | 26.6043 | 17.07349 | 30.02196 | 65.34046 | 44.20659 | 8.188275 |
| TCGA-CA- | 1.250387 | 317.4092 | 10.76585 | 1.677418 | 9.390415 | 16.00923 | 7.322518 | 34.34158 |
| TCGA-CM | 1.696358 | 26.66978 | 12.43401 | 3.926692 | 11.55886 | 37.79382 | 7.020582 | 38.05287 |
| TCGA-AZ- | 3.36607 | 12.15782 | 14.25823 | 4.493157 | 33.59704 | 47.86902 | 20.24264 | 42.61377 |
| TCGA-D5- | 5.148038 | 8.440314 | 12.40702 | 3.388126 | 18.84626 | 47.3789 | 11.62158 | 29.60695 |
| TCGA-AA- | 0.10597 | 3.542616 | 4.016378 | 0.411367 | 3.292389 | 7.293087 | 0.50034 | 12.88015 |
| TCGA-AA- | 0.950762 | 0.436783 | 14.09524 | 1.890131 | 16.6588 | 32.12194 | 3.776499 | 24.4654 |
| TCGA-DM | 0.432984 | 0.097027 | 7.012969 | 0.719782 | 5.210736 | 9.099794 | 1.643491 | 5.626853 |
| TCGA-AA- | 1.124627 | 0.075251 | 12.16228 | 1.319259 | 11.88905 | 15.02721 | 3.752756 | 42.29932 |
| TCGA-AD- | 1.486764 | 0.614773 | 8.236002 | 1.287273 | 5.381742 | 16.88157 | 2.519632 | 12.77997 |
| TCGA-A6- | 2.051332 | 4.21921 | 9.855054 | 0.994609 | 6.476835 | 11.17859 | 1.899101 | 36.38525 |
| TCGA-AA- | 3.090377 | 60.39305 | 69.22203 | 16.57714 | 88.85129 | 99.84603 | 14.9773 | 282.5164 |
| TCGA-AA- | 2.295448 | 18.09331 | 11.07436 | 2.73457 | 21.49237 | 24.86852 | 4.875798 | 23.24323 |
| TCGA-AA- | 3.445639 | 6.795126 | 16.32152 | 2.348631 | 20.82684 | 32.83205 | 10.07891 | 12.89888 |
| TCGA-AA- | 0.984762 | 1.014425 | 12.48827 | 2.637713 | 22.03065 | 32.14662 | 3.40231 | 48.97279 |
| TCGA-5M- | 5.847237 | 1.4122 | 76.67275 | 9.762163 | 62.69692 | 105.8548 | 19.30373 | 76.1133 |
| TCGA-CM | 1.585074 | 1.48728 | 41.58295 | 4.183428 | 25.433 | 87.39268 | 13.73494 | 135.8461 |
| TCGA-A6- | 0.868964 | 7.044298 | 20.70906 | 3.011723 | 31.10844 | 39.52142 | 8.550741 | 37.12314 |
| TCGA-G4- | 1.155432 | 0.12965 | 20.77231 | 4.234835 | 15.48709 | 17.67308 | 1.668491 | 17.70989 |
| TCGA-CM | 0.298484 | 4.128672 | 8.727556 | 1.111486 | 10.0644 | 29.01835 | 4.880349 | 63.03447 |
| TCGA-A6- | 3.421246 | 0.03521 | 29.01528 | 9.878052 | 72.54326 | 97.54981 | 28.73362 | 186.7175 |
| TCGA-AA- | 3.757498 | 22.50451 | 24.79516 | 3.962789 | 29.36231 | 70.10189 | 9.766575 | 29.51784 |
| TCGA-AY- | 0.367144 | 95.14751 | 3.870257 | 0.207305 | 1.097273 | 3.873957 | 0.590333 | 13.90307 |
| TCGA-AA- | 0.790858 | 10.01502 | 11.7997 | 2.67252 | 21.84599 | 27.32747 | 8.178221 | 97.37839 |
| TCGA-D5- | 2.953449 | 0.354927 | 19.52085 | 4.616167 | 39.78907 | 64.31837 | 18.82766 | 144.9962 |
| TCGA-AA- | 2.353905 | 3.634389 | 12.36502 | 2.227953 | 25.51653 | 49.24477 | 5.203194 | 40.58121 |
| TCGA-AA- | 13.74848 | 175.7712 | 119.8559 | 24.49912 | 171.2248 | 207.3864 | 23.76838 | 239.9259 |
| TCGA-AZ- | 1.817032 | 5.507051 | 27.79519 | 1.565551 | 28.16806 | 72.20659 | 8.253665 | 79.46039 |
| TCGA-CM | 1.202621 | 0.093293 | 12.93506 | 2.556065 | 17.79752 | 33.17159 | 10.95241 | 23.29076 |

| | | | | | | | | |
|---|---|---|---|---|---|---|---|---|
| TCGA-CA- | 1.88119 | 0.336767 | 13.89726 | 3.710573 | 27.94524 | 37.9494 | 15.03721 | 43.5118 |
| TCGA-CA- | 0.144819 | 0.053047 | 5.695346 | 0.580195 | 2.317108 | 8.637522 | 1.75617 | 6.088438 |
| TCGA-5M- | 1.474641 | 0.765658 | 7.831402 | 1.378183 | 5.574019 | 23.88183 | 4.155117 | 23.44439 |
| TCGA-F4-( | 2.737719 | 3.161888 | 18.51738 | 5.038207 | 22.93041 | 56.97416 | 11.9696 | 63.30002 |
| TCGA-AA- | 1.146961 | 4.757578 | 17.89279 | 3.16737 | 24.97283 | 34.9308 | 7.7929 | 79.14951 |
| TCGA-AA- | 2.864796 | 2.39247 | 26.45404 | 6.434464 | 50.97811 | 56.60328 | 9.292391 | 56.82942 |
| TCGA-AA- | 1.37058 | 5.690633 | 13.82658 | 1.551809 | 20.80366 | 23.96839 | 7.748115 | 83.31813 |
| TCGA-CK- | 5.930375 | 26.70899 | 59.44906 | 6.19436 | 76.29383 | 72.11912 | 22.1593 | 76.76498 |
| TCGA-G4- | 0.728811 | 0.096921 | 10.53829 | 2.079703 | 16.9549 | 35.18936 | 8.006231 | 25.19219 |
| TCGA-CK- | 0.491439 | 0.123338 | 13.80803 | 2.860977 | 24.1409 | 40.19405 | 7.223846 | 37.53973 |
| TCGA-AZ- | 3.770448 | 1.866823 | 58.49516 | 6.74686 | 58.63155 | 71.44732 | 12.40956 | 61.20619 |
| TCGA-AA- | 0.557869 | 228.4047 | 6.283936 | 1.359739 | 14.72987 | 18.21206 | 5.113671 | 8.688414 |
| TCGA-AA- | 1.422452 | 15.82314 | 18.27321 | 3.094053 | 15.40214 | 29.20307 | 5.181625 | 113.1374 |
| TCGA-A6- | 2.866193 | 4.127995 | 29.64129 | 8.929012 | 64.76424 | 45.04831 | 14.96724 | 119.587 |
| TCGA-AA- | 8.9958 | 6.43239 | 22.56617 | 5.505919 | 47.74263 | 60.47305 | 14.75353 | 148.6658 |
| TCGA-A6- | 3.254198 | 4.650868 | 46.17596 | 8.288424 | 72.84784 | 69.87333 | 14.40937 | 166.3992 |
| TCGA-CM | 2.098956 | 0.235176 | 28.35873 | 6.540121 | 30.24003 | 42.39578 | 7.560308 | 237.46 |
| TCGA-CM | 0.318971 | 10.53686 | 13.29308 | 1.981982 | 16.74181 | 20.42515 | 6.445812 | 13.50492 |
| TCGA-CM | 0.483414 | 6.214823 | 10.42908 | 1.408793 | 16.10948 | 22.86 | 5.200754 | 73.44952 |
| TCGA-AA- | 2.290283 | 9.612448 | 13.77494 | 3.759243 | 34.61069 | 38.44127 | 7.929993 | 41.35423 |
| TCGA-CM | 1.298633 | 19.98842 | 4.864774 | 1.269722 | 12.16186 | 14.89392 | 6.7173 | 50.84322 |
| TCGA-A6- | 2.407955 | 0.21366 | 31.90493 | 5.83203 | 54.41876 | 68.40773 | 10.81998 | 70.35434 |
| TCGA-AA- | 2.988742 | 128.4461 | 13.37536 | 3.373736 | 36.02474 | 57.76583 | 10.99919 | 34.56628 |
| TCGA-DM | 0.93097 | 0.105005 | 10.0928 | 0.38449 | 4.187134 | 7.040587 | 1.262753 | 8.305521 |
| TCGA-A6- | 2.308603 | 18.27056 | 24.19185 | 11.17409 | 67.81332 | 87.77223 | 11.63703 | 120.4684 |
| TCGA-DM | 0.254321 | 0.278168 | 4.533984 | 0.465236 | 5.264984 | 7.492786 | 0.669718 | 6.069309 |
| TCGA-AA- | 2.318794 | 4.004182 | 54.95394 | 2.598477 | 48.06748 | 66.55717 | 5.669069 | 66.36253 |
| TCGA-AA- | 1.424646 | 36.93743 | 16.85179 | 3.816348 | 42.69652 | 69.30408 | 10.24082 | 139.6138 |
| TCGA-DM | 0.073654 | 0.007708 | 3.989449 | 0.364364 | 1.633376 | 4.949949 | 1.010583 | 17.99098 |
| TCGA-A6- | 1.221746 | 0.233296 | 3.673264 | 1.592003 | 6.728114 | 7.67474 | 3.699487 | 3.029119 |
| TCGA-G4- | 14.431 | 0.973967 | 166.6146 | 147.4746 | 199.9425 | 315.2982 | 126.9642 | 1413.551 |
| TCGA-CM | 0.556364 | 0.073593 | 3.933987 | 0.251972 | 3.536983 | 5.000434 | 0.514837 | 4.368141 |
| TCGA-CK- | 1.582802 | 127.0257 | 81.44367 | 5.620004 | 64.51044 | 116.8448 | 8.455831 | 330.5366 |
| TCGA-AZ- | 0.098999 | 1.049182 | 10.22963 | 0.231259 | 3.847593 | 4.484403 | 1.049661 | 41.46142 |
| TCGA-AA- | 1.237049 | 7.321713 | 14.16537 | 2.633455 | 20.91786 | 25.62388 | 11.58269 | 17.51305 |
| TCGA-D5- | 0.675631 | 63.79089 | 12.21174 | 2.975465 | 20.35211 | 28.84811 | 6.557242 | 76.02925 |
| TCGA-AA- | 1.101418 | 215.9077 | 10.72997 | 2.224634 | 25.14822 | 31.36872 | 9.836207 | 24.68277 |
| TCGA-CM | 2.539268 | 11.55456 | 10.70471 | 3.66872 | 20.88425 | 47.7296 | 11.16371 | 36.33933 |
| TCGA-AA- | 0.959383 | 15.71327 | 8.668593 | 1.695248 | 10.6095 | 19.26979 | 5.06056 | 40.38585 |
| TCGA-AG- | 1.051331 | 5.908081 | 15.27376 | 2.470402 | 24.99747 | 35.0155 | 4.979874 | 35.94663 |
| TCGA-AA- | 1.770098 | 41.18442 | 18.76772 | 2.864366 | 25.28643 | 23.12511 | 6.0308 | 29.7707 |
| TCGA-AA- | 1.315488 | 20.14536 | 17.79048 | 1.662623 | 17.76503 | 37.58249 | 7.125926 | 95.15971 |
| TCGA-CM | 2.510302 | 154.21 | 33.95773 | 2.710303 | 24.40621 | 54.41288 | 8.852547 | 46.40672 |
| TCGA-F4-( | 2.40437 | 4.871147 | 20.27451 | 3.30004 | 17.48104 | 41.28553 | 11.58639 | 98.76374 |
| TCGA-DM | 0.413347 | 1.185997 | 4.795051 | 0.47449 | 3.454159 | 8.914847 | 1.627428 | 3.080688 |
| TCGA-A6- | 3.532145 | 4.252247 | 15.6261 | 2.675825 | 32.27833 | 35.90009 | 4.542687 | 37.32203 |
| TCGA-CM | 0.942686 | 0.045002 | 13.25813 | 2.486705 | 20.00557 | 27.72096 | 6.76458 | 25.12131 |
| TCGA-AA- | 0.5532 | 11.72735 | 12.38906 | 2.161633 | 17.10494 | 21.21506 | 5.719967 | 64.82184 |
| TCGA-A6- | 0.617777 | 4.615162 | 15.67329 | 2.350201 | 26.15405 | 60.26938 | 8.161284 | 50.86078 |
| TCGA-A6- | 0.518049 | 0.307855 | 4.227024 | 0.248104 | 2.187235 | 5.10155 | 1.015253 | 7.109939 |
| TCGA-AA- | 1.784501 | 17.64262 | 28.66243 | 5.890769 | 32.76785 | 72.54468 | 19.99251 | 124.5508 |
| TCGA-F4-( | 0.962212 | 1.751522 | 10.59196 | 2.626687 | 19.00926 | 28.58206 | 5.006123 | 80.8045 |
| TCGA-AA- | 1.288105 | 2.436834 | 8.075194 | 0.477809 | 7.337046 | 10.82971 | 2.400549 | 32.51561 |
| TCGA-AD- | 3.003048 | 3.670748 | 17.70181 | 3.972979 | 25.79884 | 48.05789 | 5.669931 | 57.30632 |
| TCGA-D5- | 1.378694 | 0.211024 | 8.337108 | 0.535942 | 4.517989 | 6.208115 | 0.587667 | 11.69703 |
| TCGA-AA- | 2.086555 | 8.278056 | 10.85614 | 2.245279 | 20.40479 | 22.88618 | 9.177424 | 16.7538 |
| TCGA-AA- | 1.685994 | 1.896258 | 25.16205 | 3.512432 | 32.90137 | 63.6472 | 12.78391 | 135.4078 |
| TCGA-AA- | 2.621736 | 3.248138 | 18.90413 | 3.436051 | 29.17779 | 43.44249 | 12.34017 | 107.2116 |

| | | | | | | | | |
|---|---|---|---|---|---|---|---|---|
| TCGA-A6- | 1.158185 | 6.498768 | 20.02423 | 5.54505 | 33.40958 | 54.32566 | 19.06957 | 45.49932 |
| TCGA-D5- | 0.243984 | 8.854987 | 8.189473 | 0.657017 | 8.47133 | 16.06418 | 3.089235 | 71.87056 |
| TCGA-AA- | 3.90079 | 18.2958 | 27.17139 | 3.732512 | 46.80136 | 46.74202 | 16.60207 | 41.44875 |
| TCGA-CA- | 1.934055 | 0.523101 | 15.58908 | 0.928683 | 12.94934 | 31.14402 | 4.883679 | 65.25657 |

| IFNG | KIR2DL4 | CIITA | ULBP2 | PI3 | CXCL13 | S100A8 | PGLYRP3 | UMODL1 |
|---|---|---|---|---|---|---|---|---|
| 0.070149 | 0.842517 | 1.464671 | 0.088671 | 120.4468 | 1.516565 | 0.64548 | 0 | 0.129136 |
| 0.052722 | 0.707713 | 4.452864 | 0.111072 | 74.4174 | 55.56977 | 0.489383 | 0 | 0.111761 |
| 0.012607 | 1.451782 | 4.092653 | 0.04553 | 5.535266 | 2.285551 | 3.983763 | 0 | 0.168783 |
| 0.142914 | 0.605807 | 1.710715 | 0.064518 | 143.5991 | 6.446986 | 3.186644 | 0.012366 | 0.037869 |
| 0.042297 | 1.389552 | 1.292319 | 0.057285 | 47.58942 | 20.6288 | 1.126636 | 0 | 0.067247 |
| 0.081369 | 0.718588 | 0.73299 | 0.146935 | 23.15947 | 5.517523 | 4.137695 | 0 | 0.072626 |
| 0.086167 | 1.004465 | 0.773737 | 0.012967 | 135.782 | 0.928989 | 1.582269 | 0 | 0.067295 |
| 0.036231 | 1.194539 | 1.176625 | 0.054521 | 13.35873 | 7.956295 | 1.052796 | 0.01254 | 0.1051 |
| 0.030961 | 0.492155 | 1.51618 | 0.181701 | 898.7654 | 20.21213 | 17.16816 | 0 | 0.03886 |
| 0.366431 | 0.595429 | 1.206293 | 0.066169 | 99.82091 | 18.94724 | 2.373532 | 0 | 0.067456 |
| 0.579097 | 0.17729 | 3.997195 | 0.174287 | 155.3705 | 0.805071 | 27.57792 | 0 | 0.012922 |
| 0.064696 | 0.982714 | 1.169304 | 0.175241 | 181.2263 | 0.165199 | 11.67117 | 0.016795 | 0.037895 |
| 0.150753 | 0.966749 | 3.340465 | 0.062822 | 114.1455 | 84.79556 | 2.19026 | 0 | 0.036873 |
| 0 | 0.302363 | 1.18873 | 0.140514 | 9.362716 | 14.9186 | 4.498609 | 0 | 0.048834 |
| 0.104404 | 1.032651 | 1.484614 | 0.031422 | 95.30537 | 21.31736 | 0.477531 | 0 | 0.071831 |
| 0.035198 | 0.621678 | 2.582494 | 0.079449 | 53.31003 | 8.38844 | 0.916227 | 0.036548 | 0.111919 |
| 0.037607 | 0.996352 | 1.776942 | 0 | 72.6777 | 4.353293 | 1.479803 | 0 | 0.132168 |
| 0.038997 | 0.192861 | 1.011936 | 0.088026 | 52.80859 | 15.10273 | 38.24466 | 0 | 0.055474 |
| 0.066725 | 0.636407 | 1.346676 | 0.090369 | 16.97264 | 13.20449 | 6.422548 | 0 | 0.055834 |
| 0.078361 | 0.874717 | 2.150049 | 0.084902 | 164.1237 | 24.95813 | 1.555943 | 0 | 0.057702 |
| 0.029412 | 0.706512 | 1.850452 | 0.013278 | 80.12358 | 14.55755 | 5.53746 | 0 | 0.15013 |
| 0.201099 | 0.529556 | 1.461716 | 0.049519 | 40.48227 | 11.60819 | 4.426874 | 0 | 0.039774 |
| 0.077211 | 0.272748 | 0.814041 | 0.10457 | 27.46327 | 9.496343 | 22.13198 | 0 | 0.006461 |
| 1.347171 | 1.396756 | 6.065283 | 0.063187 | 52.48046 | 7.177725 | 3.240954 | 0.018167 | 0.055632 |
| 0.038839 | 0.164637 | 0.727036 | 0.105202 | 136.5064 | 0.429752 | 1.951482 | 0.020164 | 0.025999 |
| 0.056829 | 0.281048 | 0.59793 | 0.141104 | 58.6914 | 7.352303 | 13.43422 | 0 | 0.061819 |
| 0.041518 | 1.275949 | 0.43415 | 0.131201 | 22.17309 | 2.190955 | 22.31857 | 0 | 0.034741 |
| 0.293731 | 1.136417 | 2.515971 | 0.101031 | 40.39126 | 9.702756 | 2.811171 | 0 | 0.032771 |
| 0 | 0.408477 | 0.87259 | 0.223726 | 20.39936 | 6.46777 | 2.250044 | 0 | 0.120949 |
| 0.132587 | 0.983557 | 0.831873 | 0.071827 | 18.69597 | 1.309079 | 5.730831 | 0 | 0.064348 |
| 0.048721 | 0.937979 | 1.231754 | 0.021995 | 114.6991 | 37.30141 | 2.978909 | 0 | 0.067268 |
| 0.078923 | 1.070563 | 0.868083 | 0.042755 | 21.22472 | 3.909595 | 2.293304 | 0 | 0.095097 |
| 0.903613 | 1.702399 | 5.349927 | 0.050992 | 86.98783 | 69.20415 | 1.777809 | 0 | 0.040956 |
| 0 | 0.498133 | 1.456385 | 0.047745 | 45.20882 | 10.75727 | 0.661586 | 0.036606 | 0.106197 |
| 0.258903 | 3.378528 | 4.886448 | 0.082504 | 44.26835 | 4.666573 | 3.245263 | 0 | 0.028036 |
| 0.161157 | 1.4964 | 1.208674 | 0.06236 | 62.98344 | 5.094844 | 0.83622 | 0 | 0.088616 |
| 0 | 0.059527 | 0.449598 | 0.06086 | 7.078767 | 0.803209 | 1.877026 | 0 | 0.016921 |
| 0.064111 | 0.498237 | 1.259889 | 0.03859 | 16.82646 | 7.675956 | 9.05586 | 0 | 0.057223 |
| 0.082837 | 1.392875 | 0.988139 | 0.074793 | 105.8268 | 6.726379 | 1.48435 | 0 | 0.05268 |
| 0.164506 | 0.79419 | 0.991081 | 0.099021 | 47.45867 | 7.211015 | 7.56858 | 0 | 0.022942 |
| 1.300553 | 0.431575 | 2.784753 | 0.106751 | 456.7283 | 12.24375 | 28.10468 | 0 | 0.009893 |
| 0 | 1.070189 | 0.972861 | 0.025805 | 9.562588 | 5.59513 | 1.868606 | 0 | 0.098054 |
| 0.206208 | 0.072843 | 1.135835 | 0.297894 | 12.79749 | 2.86089 | 3.295559 | 0 | 0.117333 |
| 0.139989 | 0.012363 | 0.337352 | 1.595739 | 16.54835 | 0.327669 | 0.16949 | 0.03634 | 0.064426 |
| 1.853715 | 2.241504 | 10.86413 | 15.32081 | 341.1118 | 11.53004 | 2.546483 | 0.111048 | 0.029829 |
| 1.427937 | 0.166506 | 4.097336 | 0.250344 | 72.25441 | 7.209758 | 14.28403 | 0.028791 | 0.051042 |
| 0.604563 | 0.133476 | 6.682866 | 0.341159 | 2.951173 | 4.148765 | 1.418184 | 0 | 0.050588 |
| 0 | 0.065541 | 0.06587 | 1.382054 | 4.601984 | 0.059221 | 2.864147 | 0 | 0.128084 |
| 0.388598 | 0.078441 | 2.455815 | 0.200493 | 155.7397 | 1.46478 | 2.486875 | 0.057644 | 0.325167 |
| 0.045108 | 0.270881 | 0.320282 | 7.839979 | 62.60601 | 7.409925 | 0.436906 | 0 | 0.052842 |
| 0.13158 | 0 | 0.277451 | 0.475211 | 8.832619 | 0.078396 | 0.908061 | 0.09564 | 0.1079 |
| 0.20176 | 0.342103 | 2.486613 | 2.113144 | 495.6619 | 6.800464 | 19.15142 | 1.634104 | 0.033765 |
| 0 | 0.049054 | 1.288377 | 5.968111 | 1096.321 | 1.004665 | 5.783655 | 0.273967 | 0.013944 |
| 1.579521 | 0.706753 | 20.3823 | 1.901514 | 54.90382 | 7.394278 | 4.525972 | 0.109341 | 0.035245 |
| 0.46186 | 0.533949 | 2.919834 | 0.530739 | 29.15981 | 16.11764 | 2.262177 | 0 | 0.463763 |
| 0.127535 | 0.202731 | 0.850666 | 0.2303 | 46.64151 | 0.244241 | 6.25363 | 0.033107 | 0.112053 |

| | | | | | | | | |
|---|---|---|---|---|---|---|---|---|
| 0.263943 | 0.776979 | 1.404632 | 3.296649 | 168.356 | 1.984462 | 5.113047 | 0.365425 | 0.044172 |
| 0.385606 | 0 | 1.104236 | 1.645849 | 201.6459 | 3.192587 | 22.66428 | 0.1092 | 0.064533 |
| 0.408851 | 0.064189 | 1.581506 | 1.825232 | 5.418046 | 11.87049 | 0.632514 | 0.165097 | 0.095032 |
| 0.335891 | 0.027918 | 0.744823 | 0.624385 | 607.312 | 0.992222 | 5.430307 | 0.615488 | 0.042986 |
| 0.373574 | 0.101511 | 0.398189 | 0.778377 | 18.62273 | 3.081852 | 0.974184 | 0.029839 | 0.211603 |
| 0.4787 | 0.563667 | 2.28429 | 7.44369 | 182.7878 | 1.629789 | 6.82616 | 0.055229 | 0 |
| 0.72369 | 0 | 1.435829 | 1.478778 | 149.8707 | 1.815494 | 2.490246 | 0 | 0.025497 |
| 0.077566 | 0.02192 | 0.311091 | 2.297109 | 26.06675 | 0.290491 | 0.093912 | 0.016108 | 0.451738 |
| 0.281554 | 1.179293 | 4.846422 | 2.651075 | 82.35822 | 24.22132 | 3.968905 | 0.020883 | 0.013463 |
| 0.127968 | 0 | 1.223614 | 1.039869 | 278.8009 | 1.82986 | 11.961 | 0.132877 | 0.05354 |
| 0 | 0.027974 | 0.165277 | 1.233375 | 652.4403 | 0.202209 | 21.71642 | 0.76061 | 0.013253 |
| 1.641992 | 0.488447 | 4.767657 | 1.521554 | 5.655235 | 22.03038 | 5.964055 | 0 | 0 |
| 0 | 0.152483 | 0.349219 | 0.545637 | 64.05712 | 5.768301 | 6.532809 | 0.313753 | 0.845204 |
| 0.014626 | 0.010333 | 0.672182 | 1.505415 | 142.2657 | 0.535294 | 1.859323 | 1.807226 | 0.102802 |
| 0 | 0.061055 | 0.576426 | 0.507179 | 68.59171 | 4.59727 | 1.569469 | 0 | 0.144626 |
| 0.708887 | 0.707049 | 2.40813 | 0.188249 | 11.70715 | 7.91481 | 9.491507 | 0 | 0.041871 |
| 0.042363 | 0.389083 | 0.401062 | 0.650239 | 212.8323 | 0.540864 | 1.179685 | 0 | 0.701875 |
| 1.556327 | 0.62632 | 3.579245 | 5.211667 | 8.73302 | 21.39597 | 22.51619 | 0.020456 | 0 |
| 2.99426 | 1.018543 | 5.405394 | 0.75097 | 3.862613 | 22.30789 | 10.98766 | 0.191922 | 0.015466 |
| 0 | 0 | 0.430855 | 1.03314 | 45.2894 | 0.121742 | 0.692697 | 0 | 0.223412 |
| 0.733863 | 0.382032 | 3.709836 | 0.697471 | 43.37547 | 12.29533 | 11.6442 | 0.401061 | 0.038784 |
| 2.050529 | 0.787333 | 1.361965 | 0.442727 | 101.2493 | 15.06282 | 9.714713 | 0 | 0.02238 |
| 0.381546 | 0.512165 | 5.06361 | 5.339658 | 405.2831 | 6.949729 | 27.57846 | 0 | 0.012771 |
| 0.063728 | 0.05628 | 0.963958 | 0.503474 | 394.3618 | 0.474623 | 0.250765 | 0.231607 | 0.125316 |
| 0.065487 | 0.231331 | 0.918694 | 0.23651 | 47.76097 | 2.647615 | 3.448992 | 2.107971 | 0.186311 |
| 0.068225 | 0 | 0.378731 | 0.277198 | 5.184604 | 1.306567 | 1.61074 | 0.035421 | 0.171265 |
| 0.149156 | 0 | 0.333768 | 0.942704 | 52.89597 | 0.571297 | 16.79477 | 0 | 0.187214 |
| 1.351209 | 0.651569 | 4.518796 | 4.918707 | 27.6627 | 16.4663 | 34.7446 | 0 | 0.017947 |
| 0.362935 | 0.153848 | 1.421249 | 0.75369 | 72.70545 | 3.120027 | 5.1412 | 0.075372 | 0.018222 |
| 0.096208 | 0.067971 | 0.246041 | 0.608058 | 172.0442 | 2.421541 | 0.249606 | 0 | 0.027601 |
| 0 | 0.16738 | 0.407806 | 0.40108 | 7.314108 | 0 | 0.143421 | 0.030751 | 0.009912 |
| 0 | 0 | 0.430579 | 1.873903 | 84.16325 | 0.892559 | 12.19378 | 0 | 0.036561 |
| 2.235522 | 0.134826 | 8.782423 | 0.910756 | 101.6312 | 22.60121 | 10.00131 | 0.254775 | 0.01825 |
| 0 | 0.011576 | 0.41881 | 0.325454 | 11.93223 | 0.41837 | 9.14495 | 0 | 0.01371 |
| 0.052774 | 0.012428 | 0.482964 | 0.349424 | 24.47101 | 0.553993 | 0.851931 | 0.018266 | 0.079487 |
| 0.050997 | 0.024019 | 0.364294 | 0.659974 | 1.901824 | 1.982218 | 1.831734 | 0 | 0.207674 |
| 0.380737 | 0.119551 | 1.321658 | 0.19098 | 13.48439 | 4.212855 | 0.819506 | 0.043927 | 0.056638 |
| 0.427687 | 0.161152 | 1.206111 | 0.386154 | 2.708435 | 3.385449 | 5.109112 | 0 | 0.073961 |
| 1.072425 | 0.392863 | 6.436992 | 2.438638 | 2504.687 | 16.66701 | 4.039539 | 0.082486 | 0 |
| 0 | 0 | 0.316874 | 0.755509 | 38.94792 | 0.747827 | 1.418344 | 0 | 0.07702 |
| 0.090988 | 0.398551 | 1.843395 | 4.830534 | 6.453469 | 1.022266 | 0.55081 | 0 | 0.042636 |
| 0.047833 | 0.067588 | 1.860797 | 0.475072 | 279.8955 | 0 | 0.579136 | 0 | 0.232148 |
| 0.194267 | 0.088232 | 1.703024 | 0.714138 | 1.786789 | 0.377946 | 1.394438 | 0.360215 | 0.078956 |
| 0.054604 | 0 | 0.211477 | 0 | 92.21103 | 0.975997 | 1.718874 | 0 | 0.018276 |
| 0.152387 | 0.095699 | 0.492551 | 1.605202 | 27.91436 | 1.916746 | 1.742502 | 0 | 0.875586 |
| 0 | 0.011889 | 0.130353 | 2.780577 | 0.888096 | 0.028647 | 0.346375 | 0 | 0.763226 |
| 0.460875 | 1.089529 | 2.424169 | 28.07207 | 46.36095 | 4.888349 | 28.3291 | 0 | 0.017799 |
| 0.767377 | 0.206533 | 1.270575 | 0.791837 | 5.052452 | 5.722884 | 1.769696 | 0.018972 | 0.064212 |
| 0.028662 | 0.070873 | 0.781976 | 0.54345 | 74.23707 | 0.158572 | 1.544238 | 2.113063 | 0.062357 |
| 0.232477 | 0.016424 | 0.666275 | 0.209901 | 1.030554 | 2.503099 | 0.971065 | 0 | 0.383223 |
| 0.052111 | 0.220897 | 0.444907 | 0.366993 | 132.046 | 0.239513 | 1.034716 | 0 | 0.70814 |
| 1.263014 | 0.797675 | 8.342054 | 0.933025 | 130.5994 | 7.117921 | 5.16676 | 0.55638 | 0.041633 |
| 0.501743 | 1.463657 | 2.831955 | 0.423792 | 417.6762 | 5.813525 | 3.919212 | 0.016806 | 0.027087 |
| 0.030035 | 0.02122 | 0.204216 | 0.21695 | 4.247954 | 0.230083 | 0.727299 | 0.187126 | 0.316672 |
| 0.052666 | 0.009302 | 0.516734 | 0.844046 | 176.8225 | 0.493098 | 0.685473 | 0 | 0.149836 |
| 0.270102 | 0.027261 | 0.71732 | 1.532914 | 86.58338 | 3.054361 | 4.625027 | 0.120199 | 0.200183 |
| 0.092883 | 0.39373 | 3.653279 | 1.006361 | 593.3044 | 0.316231 | 1.349484 | 0.771572 | 0.031089 |
| 1.573547 | 0.682183 | 10.508 | 4.262226 | 91.11836 | 15.76756 | 6.581427 | 1.782451 | 0 |

| | | | | | | | | |
|---|---|---|---|---|---|---|---|---|
| 0.018551 | 0 | 0.957972 | 0.552741 | 1.997164 | 3.600101 | 1.4824 | 0 | 0.288729 |
| 0.937915 | 0.342742 | 1.631145 | 15.12622 | 18.84282 | 8.671288 | 31.32598 | 0 | 0 |
| 0 | 0.209968 | 0.305114 | 0.364171 | 183.8607 | 2.186296 | 0.873865 | 0 | 0.138553 |
| 0 | 0 | 0.156543 | 0.370831 | 19.63103 | 0.174791 | 0.142076 | 0.060924 | 0.309306 |
| 0.170959 | 0.19627 | 0.595903 | 0.192946 | 0.27066 | 0.982206 | 0.388098 | 0 | 0.10729 |
| 0.089019 | 0 | 0.76359 | 0.321497 | 48.66897 | 1.793187 | 6.03557 | 0.061622 | 1.122286 |
| 0.045158 | 0.255233 | 1.333094 | 1.263963 | 40.41798 | 9.60915 | 17.11319 | 0 | 0.015115 |
| 0 | 0 | 0.27794 | 0.526951 | 27.10375 | 0.198702 | 7.301715 | 0 | 1.240435 |
| 0.043034 | 0.060807 | 0.850625 | 5.1418 | 191.4626 | 0.23198 | 1.806225 | 0.208529 | 1.190711 |
| 0.130033 | 0.183736 | 2.24201 | 1.330601 | 270.281 | 2.840743 | 5.037954 | 1.800292 | 0.007254 |
| 0.080782 | 0.034243 | 0.264198 | 0.466801 | 90.0372 | 0.481307 | 0.684643 | 0.016776 | 0.067596 |
| 0.037805 | 0.053418 | 0.304218 | 0.2048 | 33.19777 | 0.241331 | 1.190054 | 0.039255 | 0.015817 |
| 1.127434 | 0.698494 | 3.763399 | 0.360198 | 2.526379 | 14.27618 | 11.61321 | 0.036021 | 0.005806 |
| 0.172686 | 1.291185 | 2.483239 | 1.806029 | 3.979399 | 6.736666 | 154.6818 | 0 | 0.077065 |
| 0.910614 | 0.70768 | 1.014924 | 0.452202 | 36.52239 | 14.88137 | 168.6844 | 0 | 0 |
| 0 | 0.114682 | 0.47733 | 0.219843 | 25.29943 | 0.644765 | 0.622355 | 0.028092 | 1.208877 |
| 0.16867 | 0.134061 | 0.896407 | 5.235002 | 182.7424 | 1.650989 | 4.518262 | 0 | 0.077626 |
| 2.058086 | 0.078596 | 2.834086 | 1.858227 | 20.54808 | 3.976937 | 31.71991 | 0 | 0.074471 |
| 0.355836 | 0.153631 | 5.401945 | 6.407768 | 119.4313 | 86.70513 | 3.757733 | 0.020527 | 0.109176 |
| 0.098364 | 0 | 0.563817 | 0.479583 | 0.49833 | 0.066978 | 0.333458 | 0 | 1.195105 |
| 0.282872 | 0.074943 | 0.894698 | 1.436638 | 74.71454 | 2.016425 | 4.966002 | 0 | 0.076927 |
| 0.189684 | 0.089341 | 0.604035 | 2.34061 | 202.3395 | 0 | 0.995182 | 0.065654 | 0.22221 |
| 0.080593 | 0.056939 | 0.915591 | 1.673637 | 149.8806 | 1.234748 | 2.829733 | 0 | 0.269751 |
| 0 | 0.012707 | 0.611445 | 3.231611 | 454.9163 | 2.265688 | 0.152433 | 0.298813 | 0.10234 |
| 0 | 0.022451 | 0.206489 | 0.043038 | 2.582577 | 0 | 0.230846 | 0.016498 | 0.130293 |
| 0.244613 | 0.478573 | 1.302029 | 1.579987 | 2.542065 | 15.83942 | 3.234989 | 0.039076 | 0.003149 |
| 0.321075 | 0.264644 | 0.766829 | 0.410685 | 23.32632 | 3.211082 | 3.109891 | 0.027783 | 0.013433 |
| 0.027092 | 0.00957 | 0.258817 | 1.296437 | 187.0076 | 0.092238 | 0.442816 | 0 | 0.061208 |
| 2.286068 | 0.981907 | 5.238517 | 2.52145 | 1041.502 | 25.95866 | 585.2062 | 5.260057 | 0.021738 |
| 0.166162 | 0 | 0.386122 | 1.912832 | 56.03296 | 0.247501 | 5.280923 | 0 | 0.180751 |
| 0.073088 | 0.077455 | 1.267514 | 0.329954 | 19.82544 | 0.622094 | 0.929152 | 0.834815 | 0.103969 |
| 0.217466 | 0.059092 | 2.411531 | 1.525476 | 65.115 | 0.996677 | 0.648109 | 0.01737 | 0.072788 |
| 0.085683 | 0.036321 | 2.010244 | 1.067598 | 231.6213 | 5.615544 | 14.95919 | 0.373673 | 0.13479 |
| 0 | 0.077577 | 0.478821 | 0.495709 | 1.622524 | 0.124614 | 0.17726 | 0 | 0.073505 |
| 0.169516 | 1.586849 | 0.466863 | 5.930846 | 3.444148 | 7.502752 | 5.387511 | 0 | 0.024823 |
| 0.014112 | 0 | 0.502836 | 0.649833 | 44.29743 | 0.468456 | 2.135775 | 0.014654 | 0.151151 |
| 0 | 0.049583 | 0.259729 | 0.253465 | 100.2959 | 0.029868 | 0.127457 | 0 | 3.106589 |
| 0.09881 | 0.418855 | 0.453555 | 2.43854 | 39.28263 | 3.588379 | 4.067524 | 0 | 0.181899 |
| 0.098794 | 0.104697 | 0.47474 | 0.535203 | 48.62619 | 0.798847 | 2.890669 | 0.017097 | 0.126758 |
| 0 | 0.014398 | 0.122777 | 1.288022 | 40.65307 | 0.884643 | 1.579135 | 0 | 0.73668 |
| 0.530147 | 1.04561 | 2.889735 | 5.444812 | 330.5066 | 33.7863 | 5.643086 | 0 | 0.003697 |
| 0.45325 | 0.101888 | 2.540916 | 1.953167 | 22.09276 | 1.753567 | 4.938889 | 0.021393 | 0.075853 |
| 0.037426 | 0.211532 | 0.698981 | 0.270334 | 94.48844 | 0.923808 | 1.631277 | 0.272034 | 0.263064 |
| 1.152824 | 1.123402 | 6.777649 | 6.998985 | 501.053 | 4.466288 | 12.80254 | 0.041278 | 0 |
| 0.23872 | 0.309201 | 1.21219 | 4.167073 | 14.94959 | 1.219124 | 6.64762 | 0 | 0.146486 |
| 2.069266 | 0.202421 | 3.244025 | 0.632355 | 26.94753 | 29.50792 | 3.546012 | 0.132225 | 0 |
| 0.137136 | 0.924821 | 0.724255 | 0.697887 | 79.2656 | 5.167672 | 6.007457 | 0 | 0.0459 |
| 0.167532 | 0.02959 | 0.223491 | 0.340342 | 46.85818 | 0.178244 | 1.36915 | 0 | 0.091121 |
| 0 | 0.102801 | 0.525976 | 0.65689 | 163.2021 | 0.330266 | 2.877473 | 0 | 0.073054 |
| 0.129309 | 0.56641 | 1.117358 | 25.59205 | 460.649 | 3.301855 | 2.035269 | 0 | 0.012984 |
| 0.215231 | 0.011697 | 2.265625 | 4.20052 | 246.1797 | 6.270888 | 6.414469 | 0 | 0.094205 |
| 0.986172 | 0.334429 | 6.457324 | 2.315055 | 88.34855 | 16.51906 | 13.56376 | 0 | 0.019805 |
| 1.377066 | 0.636122 | 2.694004 | 0.191283 | 135.3932 | 17.49124 | 7.887434 | 1.704874 | 0.026591 |
| 0.220228 | 0.424337 | 2.212453 | 13.84662 | 268.8292 | 1.687028 | 7.829452 | 0.103944 | 0.013402 |
| 0.410333 | 0.695757 | 0.766347 | 14.04142 | 1024.431 | 14.45921 | 8.892807 | 0.085215 | 0.020601 |
| 2.823498 | 1.22181 | 13.9607 | 3.696501 | 22.79767 | 28.41819 | 14.74232 | 0 | 0.005907 |
| 0.035185 | 0.174005 | 0.508737 | 0.349448 | 146.539 | 1.736962 | 3.237559 | 0.036535 | 0.047106 |
| 0 | 0 | 0.664548 | 0.387311 | 28.97651 | 0.182559 | 0.77905 | 0 | 1.722944 |

| | | | | | | | | |
|---|---|---|---|---|---|---|---|---|
| 0.07918 | 0.13985 | 0.398656 | 0.357453 | 14.0399 | 0.707636 | 1.91731 | 0.123326 | 0.019876 |
| 0 | 0.232909 | 1.049803 | 1.53788 | 41.8701 | 1.215924 | 2.660937 | 0 | 0.119537 |
| 0.137042 | 0.124483 | 2.053947 | 0.494936 | 9.794354 | 2.049598 | 1.848842 | 0.020329 | 0.062251 |
| 0 | 2.895116 | 3.083534 | 0 | 153.4814 | 4.384093 | 0.999173 | 0.088647 | 0 |
| 0.282978 | 0.085681 | 1.015938 | 1.67899 | 88.66289 | 1.410738 | 2.153559 | 0 | 0.196195 |
| 0.154054 | 0.380934 | 1.640765 | 17.59533 | 272.4316 | 1.802943 | 6.668021 | 0.039991 | 0.012891 |
| 4.880764 | 1.663626 | 15.98829 | 1.778182 | 90.55703 | 32.94269 | 9.797022 | 0 | 0.007165 |
| 0.041169 | 0.174513 | 1.176342 | 2.044392 | 263.7523 | 14.78721 | 171.2151 | 0 | 0.00689 |
| 0.037033 | 0.026164 | 0.133865 | 5.41674 | 109.5991 | 0.094562 | 18.38319 | 0 | 0.33467 |
| 0.2403 | 0.169771 | 2.447316 | 0.361608 | 152.0914 | 8.863049 | 2.909395 | 0.166346 | 0.100538 |
| 0.142507 | 0.100681 | 0.35875 | 0.353838 | 94.00647 | 0.788419 | 2.890017 | 0.073987 | 0.316 |
| 0 | 0.103733 | 0.758204 | 0.530275 | 63.22768 | 1.124749 | 0 | 0 | 0.540584 |
| 0.520354 | 0.051059 | 2.133487 | 0.19576 | 50.61907 | 1.267188 | 10.32518 | 0.030018 | 0.162071 |
| 0.129892 | 0.027531 | 1.169349 | 0.433931 | 23.11438 | 0.486456 | 6.699495 | 0 | 0.236944 |
| 0.267899 | 0.054077 | 1.745737 | 2.07329 | 19.95455 | 0.651495 | 2.502168 | 0 | 0.038429 |
| 0.130003 | 0.169563 | 0.640341 | 1.327277 | 18.85088 | 3.804731 | 7.240345 | 0.051919 | 0.041839 |
| 0.1545 | 0.090962 | 0.589507 | 1.162474 | 1530.237 | 0.175338 | 0.249412 | 0.133689 | 0.017237 |
| 1.173564 | 0.452247 | 7.015149 | 0.3532 | 50.52186 | 8.445097 | 4.21957 | 2.252537 | 0.011903 |
| 1.083296 | 0.433695 | 3.558449 | 4.597061 | 570.0866 | 4.948338 | 5.421217 | 0 | 0.060431 |
| 0 | 0.069936 | 0.326587 | 0.23834 | 61.7826 | 0.168512 | 0.639205 | 0 | 0.844885 |
| 0.066121 | 0.035036 | 1.361519 | 2.671595 | 497.4522 | 0.239188 | 4.923417 | 0.017165 | 0.011066 |
| 0.834592 | 0.181427 | 2.878711 | 1.80851 | 162.0815 | 4.163835 | 7.073292 | 0 | 0.03653 |
| 0.705695 | 0.455836 | 2.261115 | 1.765858 | 1076.899 | 1.287116 | 12.10821 | 0.376852 | 0.030369 |
| 0.264048 | 0.293148 | 3.269215 | 0.17029 | 162.4773 | 6.341023 | 1.621296 | 0.039168 | 0.154663 |
| 0.151695 | 0.348308 | 1.153797 | 0.787542 | 67.32526 | 16.02645 | 6.313389 | 0.019689 | 0.0952 |
| 0.149843 | 0.176439 | 0.87265 | 0.992139 | 50.8198 | 2.763347 | 1.753726 | 0.311182 | 0.117025 |
| 1.881634 | 1.285055 | 5.833578 | 9.136361 | 154.7305 | 6.833314 | 6.429476 | 0 | 0.003499 |
| 0.469602 | 0.171606 | 1.421547 | 0.731033 | 196.1047 | 9.289647 | 11.72417 | 0.201773 | 0.165309 |
| 0.905124 | 0.648734 | 2.254584 | 0.532975 | 32.34247 | 12.21473 | 1.953499 | 0.040863 | 0.006586 |
| 0.498301 | 0.204415 | 3.645359 | 0.304779 | 27.31478 | 1.682841 | 9.57513 | 0.083454 | 1.96645 |
| 0.03365 | 0 | 0.567632 | 2.871099 | 63.67997 | 1.875988 | 12.03893 | 0.052411 | 0.030973 |
| 0.061443 | 0 | 0.57641 | 0.554768 | 169.6504 | 0.47068 | 6.844045 | 0 | 0.380463 |
| 0.411755 | 0 | 2.898037 | 0.764194 | 4.345909 | 1.713393 | 4.098998 | 0 | 0.030626 |
| 0.082989 | 0.102605 | 1.914191 | 0.393383 | 16.42343 | 4.061594 | 2.361229 | 0.086173 | 0.100692 |
| 0.185869 | 0.093797 | 2.161869 | 0.28769 | 230.8164 | 5.356299 | 2.314678 | 0 | 0.022218 |
| 0.035555 | 0.276314 | 0.654853 | 1.187784 | 30.17158 | 2.133523 | 6.0482 | 0.258433 | 0.083304 |
| 0.306094 | 0.108127 | 1.169682 | 0.580377 | 47.26517 | 2.449012 | 9.116746 | 0.031784 | 0.199782 |
| 0.407658 | 0.403213 | 0.343836 | 3.68071 | 59.03259 | 17.21019 | 30.20626 | 0 | 0 |
| 0.186026 | 0.219044 | 0.445623 | 2.911322 | 16.16553 | 3.245894 | 1.651671 | 0.064387 | 0.295755 |
| 0.090657 | 0 | 0.25436 | 0.147336 | 1.798872 | 0.046298 | 1.800089 | 0 | 0.661489 |
| 0.065673 | 0 | 0.39 | 0.237182 | 134.2864 | 1.024791 | 1.696267 | 0.068192 | 0.377344 |
| 0 | 0.230283 | 0.613662 | 0.478234 | 93.14542 | 1.248452 | 1.430569 | 0.042307 | 0.15001 |
| 0.019929 | 0.01408 | 0.255567 | 0.305895 | 19.73026 | 0.339255 | 0.723868 | 0 | 0.106727 |
| 0.405259 | 0.087139 | 1.231374 | 0.699991 | 101.28 | 3.269404 | 18.98645 | 0 | 0.392185 |
| 0.355924 | 0.377188 | 1.488242 | 1.673751 | 180.0677 | 1.691445 | 1.095279 | 0 | 0.332572 |
| 0.04635 | 0.032746 | 0.809797 | 0.083698 | 18.58984 | 0.236706 | 0.561177 | 0 | 1.132501 |
| 0.022597 | 0.03193 | 2.228803 | 0.530477 | 178.8794 | 46.10343 | 0.683985 | 0.258107 | 0.06429 |
| 0.083319 | 0.029432 | 0.466111 | 0.827512 | 22.42476 | 0.88647 | 1.36185 | 0.043258 | 0.020916 |
| 0.50938 | 0.296368 | 0.372641 | 1.09568 | 0.885509 | 1.836257 | 0.52603 | 0 | 0.07271 |
| 0.131503 | 0.023227 | 0.481008 | 0.267148 | 78.28072 | 1.119288 | 4.696838 | 0.034137 | 0.066022 |
| 0.155741 | 0.031437 | 1.619966 | 1.446352 | 174.1945 | 3.692737 | 8.377527 | 0.207921 | 0.052128 |
| 0.131059 | 0 | 0.307371 | 1.064987 | 592.6631 | 0.390429 | 15.31239 | 1.258802 | 0.01645 |
| 1.75267 | 0.77391 | 5.954589 | 0.435179 | 22.84592 | 19.9527 | 12.20161 | 0 | 0.014666 |
| 0 | 0 | 0.212676 | 0.495805 | 17.20642 | 0.175273 | 3.843674 | 0.017819 | 0.037334 |
| 0.074774 | 0 | 0.580803 | 0.337565 | 699.1626 | 0.525066 | 0.294228 | 0.038821 | 0.406697 |
| 0 | 0.028816 | 0.214131 | 5.339783 | 11.19271 | 2.499542 | 0.592585 | 0 | 0.027303 |
| 1.362243 | 0.740323 | 11.87754 | 6.90668 | 166.1202 | 2.586525 | 2.91802 | 0.108808 | 0.035073 |
| 0.593742 | 0.396172 | 4.235684 | 8.845391 | 301.9855 | 6.6259 | 21.9653 | 0 | 0.00552 |

| | | | | | | | | |
|---|---|---|---|---|---|---|---|---|
| 0 | 0.019422 | 0.508695 | 0.645355 | 140.6676 | 0.070197 | 0.299559 | 0.884914 | 0.027604 |
| 0.279867 | 0.47454 | 1.180259 | 0.404303 | 56.71454 | 0.666987 | 5.285981 | 0 | 0.365327 |
| 0.102376 | 0.024109 | 0.54922 | 2.125981 | 6.879387 | 0.726144 | 1.32213 | 0 | 0.371213 |
| 0 | 0.046928 | 2.23525 | 0.059973 | 616.8014 | 0.339218 | 0.241263 | 5.724628 | 0.077813 |
| 0.249745 | 0.040718 | 1.096217 | 2.966094 | 34.87574 | 7.063905 | 9.815563 | 0.239378 | 0.064301 |
| 0.130934 | 0.046252 | 21.5151 | 0.147773 | 0.414585 | 100.8573 | 7.173299 | 0 | 0.076693 |
| 0.460873 | 0.494437 | 4.317637 | 0.95553 | 17.83585 | 11.57932 | 3.5753 | 1.222969 | 0.02571 |
| 0.074141 | 0.117856 | 1.225152 | 2.142124 | 64.44132 | 5.884585 | 4.757563 | 0.038493 | 0.068243 |
| 0.435856 | 0.500387 | 1.976423 | 0.073787 | 18.57363 | 2.991026 | 0.758578 | 0 | 0.159561 |
| 0 | 0.74343 | 0.454635 | 1.387133 | 297.7836 | 9.243095 | 1.605277 | 0 | 0.01761 |
| 1.344651 | 2.111091 | 1.491607 | 0.269794 | 393.7558 | 6.803437 | 4.612713 | 0 | 0.043756 |
| 0.079793 | 0.056373 | 0.782883 | 0.396242 | 238.168 | 1.052693 | 2.705016 | 0 | 0.433991 |
| 0.041708 | 0.058933 | 1.017652 | 1.638103 | 143.9481 | 7.348432 | 3.534793 | 0.281501 | 0.080269 |
| 0.060682 | 0.05359 | 0.708972 | 1.177969 | 57.00218 | 0.942607 | 4.72044 | 0.094515 | 1.77211 |
| 0.92562 | 0.326974 | 1.832272 | 1.253601 | 78.64479 | 5.317947 | 3.782297 | 0.240282 | 0.077453 |
| 0 | 0.091462 | 0.137881 | 0.935095 | 85.19386 | 0.123963 | 0.881662 | 0.016803 | 0.197696 |
| 0 | 0 | 0.686318 | 0.334883 | 142.5735 | 0.757664 | 13.02281 | 0 | 0.341394 |
| 0.176223 | 0.135819 | 1.018928 | 0.969125 | 194.5857 | 7.186011 | 6.478365 | 0.016635 | 0.112604 |
| 0.092768 | 0.09831 | 0.726555 | 0.879472 | 170.5639 | 0.986995 | 3.088726 | 0 | 1.389491 |
| 0.119523 | 0.063332 | 1.242135 | 0.242812 | 8.736017 | 1.525988 | 7.470705 | 0.031027 | 0.127517 |
| 0.50045 | 0.390142 | 2.854604 | 1.028353 | 41.78283 | 13.67478 | 4.136923 | 0.017919 | 0.236817 |
| 0 | 0.057929 | 0.222292 | 3.368449 | 37.99175 | 0 | 0.496367 | 0.08514 | 0.67238 |
| 0.148977 | 0.070168 | 0.21156 | 2.040068 | 13.88946 | 1.437092 | 1.893903 | 0 | 0.103883 |
| 1.462725 | 2.680052 | 13.31808 | 0.667597 | 2.137633 | 14.58432 | 6.714128 | 0.016691 | 0.04304 |
| 0.428097 | 0.302449 | 0.639754 | 1.370408 | 273.2396 | 5.664396 | 5.183127 | 0.161644 | 0.16934 |
| 1.428237 | 0.49251 | 6.718277 | 2.011073 | 1.040856 | 5.745387 | 5.414099 | 0 | 0.022764 |
| 0.169802 | 0.079977 | 0.623537 | 0.970983 | 44.80488 | 0 | 0.548229 | 0.058772 | 1.117738 |
| 0.339993 | 0.080068 | 3.468728 | 1.048836 | 28.70788 | 2.278914 | 3.87629 | 0.01471 | 0.113798 |
| 0.256021 | 0.081395 | 1.405805 | 0.323622 | 9.944069 | 0.501199 | 1.875338 | 0 | 0.26993 |
| 0.109378 | 0.48941 | 0.850369 | 0.164594 | 3.386359 | 0.268948 | 0.191285 | 0.025239 | 0.256267 |
| 0.258384 | 0.422141 | 8.724238 | 2.332928 | 460.4094 | 24.6178 | 6.686846 | 0.788121 | 0.045944 |
| 0.376984 | 0.190241 | 2.933984 | 0.875251 | 767.7021 | 4.904739 | 9.715387 | 0.726971 | 0.081115 |
| 0.032816 | 0 | 0.505553 | 2.370335 | 24.17583 | 1.089323 | 0.834359 | 0.102225 | 2.581175 |
| 1.996557 | 0.1216 | 5.972741 | 3.092517 | 406.2699 | 9.522363 | 1.854655 | 0.017872 | 0 |
| 0.460349 | 0.146356 | 0.954846 | 0.509165 | 29.85727 | 4.329689 | 0.836041 | 0.01195 | 1.348218 |
| 0.322217 | 0.142278 | 5.133845 | 0.569731 | 105.3985 | 14.5127 | 11.96366 | 0 | 0.047184 |
| 0.110149 | 0.164286 | 0.198027 | 0.519361 | 16.04351 | 0.479183 | 1.511422 | 0 | 0.026627 |
| 0.533882 | 0.546269 | 0.777962 | 3.706702 | 1069.972 | 25.60396 | 3.477134 | 0.229392 | 0.015405 |
| 0.233163 | 0 | 0.969921 | 1.368386 | 45.19931 | 3.506083 | 7.551497 | 0 | 0.087797 |
| 0.161462 | 0 | 0.979691 | 0.291565 | 41.75206 | 1.649147 | 2.052622 | 0 | 0.108085 |
| 0.178202 | 0.107914 | 1.854692 | 1.873305 | 43.71641 | 2.719361 | 3.405862 | 0.013217 | 0.136333 |
| 0.118318 | 0.401237 | 0.615058 | 0.448677 | 10.58978 | 2.920487 | 2.03417 | 0.024571 | 0.071283 |
| 0.190062 | 0.111899 | 0.433482 | 4.776347 | 11.70183 | 0.620126 | 23.74018 | 0 | 0.026506 |
| 0.083479 | 0.101104 | 1.963465 | 1.022911 | 68.01985 | 23.31565 | 1.905909 | 0.012383 | 0.023949 |
| 0.410909 | 0.476931 | 5.590218 | 0.159003 | 64.24925 | 8.119118 | 2.949479 | 0.09143 | 0.02456 |
| 0.027432 | 0.019381 | 0.55365 | 0.408679 | 0.492213 | 2.043047 | 2.37475 | 0 | 0.149204 |
| 0 | 0 | 0.074818 | 0.436052 | 4.893453 | 0 | 0.444391 | 0.020059 | 1.115365 |
| 0.238815 | 0.120516 | 2.683729 | 1.170528 | 17.78818 | 2.381138 | 7.063313 | 0.07085 | 0.062805 |
| 0.157289 | 0.037041 | 0.351966 | 0.28403 | 297.2722 | 0.580133 | 2.983486 | 0.163323 | 0.06142 |
| 1.269097 | 0.33623 | 2.402962 | 0.71616 | 171.7883 | 13.70498 | 6.434263 | 0 | 0.026549 |
| 0 | 0.170015 | 1.26339 | 0.977746 | 243.5781 | 0.102413 | 1.456792 | 0 | 0.382593 |
| 0.534212 | 0.148981 | 1.214766 | 1.167758 | 88.67101 | 1.962371 | 1.753138 | 0 | 0.359962 |
| 0.744809 | 0.631446 | 3.455121 | 0.302617 | 109.3487 | 3.645193 | 3.156184 | 0 | 0 |
| 0.191634 | 0 | 0.558015 | 2.076292 | 15.97866 | 0.462144 | 1.04408 | 0.033164 | 0.427608 |
| 0.516601 | 0.126338 | 2.211107 | 1.991315 | 58.04827 | 11.92281 | 5.292413 | 0.309473 | 0.056528 |
| 0.392679 | 0.20807 | 2.420649 | 2.614779 | 108.0696 | 3.425857 | 5.526879 | 0 | 0.073931 |
| 0.056706 | 0.507458 | 1.138765 | 0.870386 | 85.8658 | 0.4183 | 1.281574 | 0 | 0.107553 |
| 0.3399 | 0.120069 | 1.078728 | 0.191808 | 60.53908 | 6.039274 | 1.543232 | 0 | 0.056883 |

| | | | | | | | | |
|---|---|---|---|---|---|---|---|---|
| 0.096725 | 0.125283 | 0.330214 | 1.208098 | 62.88719 | 1.12515 | 7.69014 | 0.050218 | 0.539578 |
| 0.03418 | 0 | 0.244161 | 1.651044 | 378.539 | 0.029092 | 0.744889 | 0.017746 | 0.065781 |
| 0.263979 | 0.087765 | 1.440681 | 1.794589 | 64.77062 | 1.718188 | 7.764599 | 0 | 0.025987 |
| 0.101486 | 0.100379 | 1.821997 | 1.337803 | 3.042031 | 19.03808 | 1.990527 | 0 | 0.016984 |
| 0.046495 | 0.098545 | 0.708285 | 1.091475 | 378.2582 | 1.820416 | 1.913962 | 0.048279 | 0.116716 |
| 0.266372 | 0.352858 | 1.762394 | 1.382903 | 1019.923 | 5.583061 | 2.297855 | 0.172869 | 0.011145 |
| 0.496005 | 0.02503 | 4.226203 | 0.255908 | 121.7541 | 0.753885 | 0.815006 | 0.993278 | 0.029646 |
| 0 | 0.046331 | 1.309411 | 0.651308 | 26.16326 | 0.055817 | 1.429159 | 0.204282 | 0.021949 |
| 0.77917 | 0.070061 | 3.157901 | 3.517526 | 111.6645 | 5.172902 | 1.183498 | 0.058841 | 0.045046 |
| 0.395403 | 0.279351 | 1.182563 | 0.28263 | 133.0941 | 2.524115 | 3.211472 | 0.017107 | 0.08823 |
| 0.433527 | 0.760041 | 3.030541 | 3.044435 | 20.23475 | 5.562297 | 6.026472 | 0 | 0.021497 |
| 0.191692 | 0.162516 | 0.669822 | 0.588462 | 66.11918 | 5.025304 | 3.017148 | 0.238855 | 0.109073 |
| 0.588324 | 0.02771 | 2.491216 | 2.514313 | 2162.405 | 11.61749 | 11.68181 | 0 | 0.013128 |
| 0.840641 | 0.39594 | 7.12248 | 0.801174 | 1.774525 | 52.0934 | 12.01562 | 0 | 0.019539 |
| 0.073852 | 0.156528 | 0.842181 | 9.385203 | 541.7731 | 1.084318 | 6.124912 | 0.038342 | 0.083426 |
| 0.161618 | 0.06851 | 0.581428 | 1.955376 | 1949.608 | 1.471911 | 5.1463 | 0.3692 | 0.05139 |
| 0.471563 | 0.145756 | 1.222632 | 5.481783 | 35.64884 | 4.565605 | 3.568359 | 0 | 0.123309 |
| 0 | 0.160412 | 1.117115 | 4.749244 | 15.09759 | 2.705586 | 1.466134 | 0 | 0.101328 |
| 0.407 | 1.031193 | 5.111774 | 2.863784 | 68.28715 | 17.09401 | 0.849603 | 0.597489 | 0.232524 |
| 0 | 0.335579 | 0.330759 | 0.155951 | 40.28896 | 0.294029 | 1.254738 | 0.089675 | 0.108397 |
| 0 | 0.11302 | 0.35453 | 0.7583 | 177.9625 | 0.544646 | 3.873698 | 0.913605 | 0.046851 |
| 0.298015 | 0.035091 | 2.217196 | 1.270631 | 280.2572 | 1.62058 | 5.271936 | 0 | 0.049874 |
| 0.073062 | 0.051618 | 0.372572 | 0.494753 | 89.56798 | 0.544136 | 1.415342 | 0.075865 | 0.042795 |
| 0.200386 | 0.028314 | 0.496695 | 0.542781 | 9.813583 | 0.818685 | 1.067505 | 0.166459 | 0.060364 |
| 0.31093 | 0.164753 | 0.822881 | 0.70184 | 24.69509 | 1.124758 | 2.117554 | 0 | 0.149601 |
| 0.07921 | 0.279808 | 1.085305 | 0.157339 | 2.67529 | 2.359691 | 0.671316 | 0 | 0.103397 |
| 0.843704 | 0.187004 | 1.255792 | 0.298734 | 277.101 | 8.448481 | 6.870102 | 0 | 0.268551 |
| 1.030804 | 0.571404 | 1.61601 | 0.859111 | 28.01946 | 2.524132 | 2.457661 | 0 | 0.382173 |
| 0.172289 | 0.256968 | 2.896429 | 5.35811 | 460.7519 | 1.156859 | 5.006309 | 0.059633 | 0.032037 |
| 0.187476 | 0.099339 | 1.210144 | 1.26953 | 3.363851 | 8.337585 | 1.532143 | 0 | 0.007844 |
| 0 | 0 | 0.106437 | 0.340657 | 36.06111 | 0.017841 | 0.507562 | 0 | 0.087697 |
| 0.01631 | 0 | 0.61622 | 0.647933 | 215.6233 | 0.152701 | 3.19894 | 0.101611 | 0.693283 |
| 0 | 0 | 0.12664 | 0.253057 | 19.91838 | 0.178917 | 0.282781 | 0.012126 | 0.214981 |
| 0 | 0.044082 | 0.40276 | 1.002791 | 6.085111 | 0.031865 | 0.710121 | 0.129579 | 0.041769 |
| 0.264003 | 0.559552 | 0.640749 | 6.912614 | 1112.497 | 1.408164 | 2.748886 | 0.219305 | 0.011782 |
| 0.159212 | 0.128552 | 2.441031 | 1.355366 | 8.594129 | 3.291046 | 16.57766 | 0.212554 | 0.072321 |
| 0.289887 | 0.226363 | 0.938881 | 0.371941 | 558.3681 | 0.545421 | 7.204255 | 0.158425 | 0.040854 |
| 0.045547 | 0.016089 | 0.125441 | 0.555175 | 43.65042 | 0.290757 | 2.371259 | 0.047294 | 0.365879 |
| 0.080958 | 0.085795 | 0.355355 | 0.950257 | 28.88154 | 0.103362 | 1.764347 | 0.252193 | 0.04742 |
| 0.564785 | 0.082556 | 7.473986 | 8.879981 | 0.328887 | 8.437442 | 5.022434 | 0 | 1.518821 |
| 0.123627 | 0.116456 | 0.585203 | 0.855768 | 312.1164 | 0.210451 | 3.891676 | 0.21395 | 0.144826 |
| 0.190284 | 0.107548 | 0.55354 | 0.171806 | 265.9087 | 1.036551 | 0.737229 | 0 | 0.089165 |
| 0.605782 | 0.047554 | 2.957325 | 0.303864 | 0.781462 | 0.888 | 0.325974 | 0.104837 | 0.270346 |
| 0.2431 | 0.101029 | 1.666421 | 0.296961 | 1457.479 | 1.983956 | 6.83885 | 1.796683 | 0.241709 |
| 0.152325 | 0.071745 | 1.254185 | 0.733509 | 214.7927 | 3.025214 | 8.422097 | 1.528965 | 0.067979 |
| 0.494673 | 0.087371 | 0.617327 | 0.781612 | 235.7313 | 1.89469 | 1.946483 | 0 | 0.082785 |
| 0.112864 | 0.265794 | 1.334017 | 1.256817 | 98.31662 | 1.889272 | 5.056 | 0 | 0.006296 |
| 0.172528 | 0.525067 | 3.945197 | 1.13835 | 105.3359 | 18.18656 | 8.692876 | 0 | 0.055525 |
| 0.203425 | 0.239531 | 5.310714 | 0.36734 | 179.2082 | 17.48769 | 6.075235 | 0.528071 | 0.096458 |
| 0.683162 | 0.146894 | 5.79915 | 0.616821 | 5.8938 | 16.19283 | 27.00756 | 0 | 0.298252 |
| 0 | 0 | 0.132842 | 0.224775 | 83.6619 | 0.169516 | 2.411307 | 0 | 0.03333 |
| 0.321344 | 0.088289 | 3.735183 | 0.419089 | 5.991921 | 1.686662 | 1.642708 | 0 | 0.032864 |
| 0 | 0.069074 | 0.403901 | 0.706203 | 34.05332 | 1.331471 | 6.037033 | 0.609123 | 0.507225 |
| 1.467999 | 0.921899 | 2.640749 | 0.359997 | 15.57064 | 15.10188 | 4.80545 | 0.018819 | 0.027297 |
| 0 | 0.021586 | 0.165662 | 0.234481 | 1.12866 | 0.182037 | 0.203454 | 0.015863 | 1.907207 |
| 0.544729 | 1.008569 | 4.636506 | 2.934024 | 214.9082 | 12.77429 | 3.411322 | 0 | 0.003144 |
| 0.058842 | 0.041572 | 0.825476 | 0.371895 | 36.89055 | 4.006682 | 1.496084 | 0 | 0.009847 |
| 0.090598 | 0.144015 | 0.46784 | 0.961146 | 83.52579 | 3.065215 | 2.303482 | 0 | 0.08339 |

| | | | | | | | | |
|---|---|---|---|---|---|---|---|---|
| 0.463329 | 0.013639 | 6.014736 | 0.313752 | 360.4523 | 2.59623 | 3.319068 | 1.323036 | 0.051693 |
| 4.685577 | 3.543294 | 30.58107 | 1.566876 | 17.87683 | 71.94905 | 20.14964 | 0 | 0.02033 |
| 0.097071 | 0.06858 | 0.556408 | 0.368107 | 22.74489 | 2.610864 | 4.160466 | 0.161272 | 0.103969 |
| 0 | 0 | 0.26155 | 0.102002 | 7.845857 | 1.913523 | 0.20517 | 0 | 0.122891 |
| 0.297782 | 0.736337 | 1.223774 | 0.302473 | 314.7683 | 2.788039 | 2.433614 | 0 | 0.143275 |
| 0.155256 | 0.027422 | 0.477698 | 1.682149 | 185.6604 | 4.129577 | 1.221829 | 0 | 0.058461 |
| 0 | 0 | 0.13118 | 2.054569 | 5.709809 | 0.036544 | 0.935689 | 0 | 0.017963 |
| 0.032307 | 0.022825 | 0.650636 | 1.808504 | 248.6455 | 1.512386 | 3.794141 | 0.16773 | 0.313585 |
| 0 | 0 | 0.452159 | 0.140432 | 7.514958 | 0.132385 | 1.297266 | 0 | 0.211128 |
| 0.111113 | 0 | 1.128433 | 0.70226 | 96.9474 | 0.252197 | 3.766792 | 0 | 0.123968 |
| 0.020592 | 0.189123 | 0.373941 | 0.464801 | 1.086683 | 0.10516 | 4.836625 | 0 | 0 |
| 4.455418 | 0.492154 | 4.732648 | 0.720689 | 1.256046 | 26.18747 | 8.767988 | 0.090417 | 0 |
| 0.190194 | 0.080623 | 0.73334 | 0.755589 | 109.6054 | 1.424584 | 3.868621 | 0.078996 | 0.50291 |
| 0.031979 | 0.022593 | 0.879355 | 3.190521 | 328.5802 | 5.906515 | 3.5427 | 0.083014 | 0.04549 |
| 0.299991 | 0 | 0.438921 | 0.625059 | 16.2698 | 2.081986 | 6.45395 | 0.047923 | 0.216266 |
| 1.090797 | 0 | 1.855697 | 0.525265 | 65.93067 | 9.593834 | 12.32623 | 0.755096 | 0.08519 |
| 0.225859 | 0.073647 | 0.311019 | 0.313733 | 10.04885 | 0.709812 | 3.155258 | 0 | 0.235513 |
| 0.143936 | 0.10169 | 0.796958 | 1.646144 | 76.56708 | 2.654421 | 1.219881 | 0 | 0.096353 |
| 0.129997 | 0 | 1.040505 | 0.176059 | 174.2517 | 0.497911 | 1.967394 | 0.202476 | 0.489497 |
| 0.173443 | 0.024507 | 0.265709 | 0.50112 | 17.28105 | 4.635472 | 32.04501 | 0 | 0.011611 |
| 0.085188 | 0 | 0.725844 | 0.673011 | 72.33464 | 1.305146 | 1.598674 | 0.088456 | 1.660887 |
| 1.478372 | 0.232103 | 3.506083 | 0.630326 | 15.17018 | 7.025622 | 3.380954 | 0.255848 | 0.206176 |
| 0.010335 | 0.014603 | 0.119193 | 0.970476 | 40.46986 | 0.02639 | 0.187697 | 0 | 0.020755 |
| 0.019347 | 0.177692 | 0.203144 | 0.681262 | 40.3091 | 0.955103 | 0.515331 | 0 | 0.025902 |
| 0.041845 | 0.206944 | 1.030006 | 0.188908 | 107.9412 | 1.032881 | 2.178523 | 0 | 0.077032 |
| 0.147787 | 0.639516 | 2.434165 | 7.005353 | 99.36108 | 2.924597 | 14.96307 | 0 | 0.018549 |
| 0.044785 | 0.205664 | 0.549263 | 0.566108 | 333.578 | 0.552728 | 1.897812 | 0.302272 | 0.022485 |
| 0.017269 | 0.0122 | 0.485998 | 0.140325 | 167.6455 | 0.264568 | 2.885263 | 0 | 0.274546 |
| 0.403259 | 0.353277 | 4.096782 | 2.373926 | 38.03372 | 0.398153 | 5.019109 | 0.150743 | 0.048591 |
| 0 | 0.039348 | 0.146996 | 0.435811 | 97.81502 | 0.553051 | 8.38393 | 0 | 0.093206 |
| 0.226608 | 0.160098 | 0.483678 | 0.491045 | 235.3485 | 1.774477 | 6.694435 | 0.04706 | 0.394406 |
| 0.153981 | 0.483499 | 0.951234 | 0.540666 | 17.80508 | 4.994905 | 2.920747 | 0.035531 | 0.071581 |
| 0.359579 | 0.278235 | 2.858944 | 0.6184 | 34.15684 | 11.95077 | 43.45253 | 0.035559 | 0.05158 |
| 0.387675 | 0.030432 | 0.404093 | 0.466705 | 179.2188 | 0.073327 | 0.625829 | 0 | 0.050461 |
| 0.28455 | 0.263857 | 2.295936 | 3.275703 | 48.01545 | 23.09942 | 5.619903 | 0.240067 | 0.011905 |
| 0.308866 | 0.109106 | 0.594788 | 1.150348 | 183.6983 | 3.384735 | 1.963263 | 0.040089 | 0.064612 |
| 0.364168 | 0.171522 | 2.31187 | 2.442544 | 66.36694 | 11.69002 | 18.20333 | 0 | 1.044769 |
| 0.314545 | 0.091504 | 1.329686 | 1.737408 | 64.71831 | 5.79547 | 2.038562 | 0.1537 | 0.030965 |
| 0.076047 | 0.537268 | 0.906484 | 1.510563 | 11.47779 | 0.906184 | 2.854248 | 0 | 0.139994 |
| 0.248457 | 0 | 0.684274 | 0.852452 | 359.2633 | 0.803604 | 3.188643 | 0 | 0.099792 |
| 0.050863 | 0 | 0.309346 | 5.449601 | 50.2479 | 0.158738 | 6.342889 | 0.088024 | 0.224152 |
| 0.226042 | 0.226239 | 1.311552 | 0.306137 | 1.908624 | 1.346774 | 2.759576 | 0.058678 | 0.01261 |
| 0 | 0.031792 | 0.271106 | 0.270867 | 5.034528 | 0.817105 | 1.47104 | 0 | 1.380664 |
| 0.465196 | 0.281708 | 2.488034 | 1.950097 | 32.89666 | 4.044373 | 14.04049 | 0 | 0.111217 |
| 0.066572 | 0.08466 | 0.705885 | 0.769377 | 83.64273 | 1.835889 | 0.967218 | 0.304156 | 0.122552 |
| 0.134446 | 0.108555 | 0.390113 | 2.323745 | 12.60899 | 0.42504 | 3.302068 | 0 | 0.167143 |
| 0.464841 | 0.098523 | 0.948161 | 0.755461 | 174.8572 | 2.927815 | 1.463278 | 0 | 0.248937 |
| 0.199012 | 0.162232 | 1.404502 | 0.359372 | 27.40331 | 0.859978 | 2.909945 | 0.015896 | 0.094792 |
| 1.366661 | 0.386216 | 1.464399 | 12.18521 | 21.5647 | 15.16571 | 10.11414 | 0.372511 | 0.037166 |
| 0 | 0.025457 | 0.187619 | 0.3416 | 12.16983 | 0.276022 | 0.327193 | 0 | 0.138694 |
| 0.74215 | 0.20973 | 2.333513 | 0.402048 | 3.968764 | 1.263366 | 1.138159 | 0.154124 | 0.314644 |
| 0.219129 | 0.092888 | 0.927886 | 1.622367 | 239.6079 | 1.342889 | 10.45311 | 0.409564 | 0.022003 |
| 0.568693 | 0.059523 | 0.119643 | 0.380346 | 11.4711 | 10.5952 | 4.437236 | 0 | 0.853031 |
| 0.118759 | 0.047945 | 0.28181 | 0.291044 | 12.39132 | 0.447651 | 1.725435 | 0.017616 | 0.428728 |
| 0.092293 | 0.04347 | 0.305816 | 0.3611 | 27.79485 | 0.078556 | 5.978241 | 0.047917 | 0.144159 |
| 0.1494 | 0.392045 | 5.066128 | 24.33835 | 5001.831 | 2.47058 | 6.821905 | 0 | 0.003572 |
| 0.258114 | 0 | 0.774738 | 0.116524 | 147.9287 | 0.439389 | 2.812568 | 0.033502 | 0.23758 |
| 0.115889 | 0.054583 | 1.462858 | 0.20927 | 433.2426 | 1.117909 | 1.730497 | 0 | 0.413747 |

| | | | | | | | | |
|---|---|---|---|---|---|---|---|---|
| 0.311235 | 0.785309 | 2.074051 | 0.642312 | 140.5025 | 6.584876 | 6.298337 | 0 | 0.037205 |
| 0.10769 | 0.266289 | 0.644152 | 0.826473 | 64.78742 | 1.099925 | 44.65645 | 0.503195 | 0.009011 |
| 0.070027 | 0.009895 | 0.532777 | 0.619619 | 479.796 | 1.001337 | 2.509599 | 0.101798 | 0.075003 |
| 0.082764 | 0.014618 | 1.15216 | 0.168135 | 3.79988 | 0.651611 | 2.379864 | 0 | 0.232001 |
| 0.267497 | 0.094493 | 0.801942 | 6.561306 | 133.2136 | 2.428594 | 1.052571 | 0.277759 | 0.115647 |
| 0.158784 | 0.11218 | 2.66484 | 5.644987 | 176.3886 | 4.307892 | 5.911551 | 0.041219 | 0.036538 |
| 0.116451 | 0.013712 | 0.268935 | 8.726821 | 91.6902 | 0.759901 | 0.587463 | 0.302296 | 0.029233 |
| 0.235366 | 0.04751 | 0.795808 | 0.182152 | 230.2494 | 1.001664 | 5.088684 | 0 | 0.264472 |
| 0.565423 | 0.216379 | 3.102295 | 0.999759 | 482.9449 | 0.882313 | 20.737 | 0.073389 | 0.023656 |
| 0.061807 | 0.043666 | 0.731425 | 0.251122 | 95.76457 | 0.973233 | 2.544284 | 0.096267 | 0.217216 |
| 0 | 0.081458 | 0.185234 | 0.763415 | 21.74248 | 0.032712 | 2.093942 | 0.039907 | 0.045023 |
| 0 | 0 | 0.130021 | 4.875332 | 18.59724 | 0.114581 | 3.47706 | 0 | 0.360462 |
| 0.014763 | 0.052151 | 0.461229 | 0.519852 | 5.702991 | 0.138223 | 0.554104 | 0 | 0.224831 |
| 0 | 0.069573 | 0.671674 | 0.333423 | 16.78586 | 0.398135 | 3.725879 | 0 | 0.074161 |
| 0.379591 | 0.187726 | 1.078101 | 0.719732 | 139.3837 | 11.04968 | 10.24873 | 0 | 0.053997 |
| 0 | 0.050789 | 1.3024 | 1.200798 | 43.55283 | 2.172196 | 7.702923 | 0 | 0.030077 |
| 0.49045 | 0.13327 | 0.785771 | 0.885645 | 131.8812 | 0.321114 | 3.56283 | 0.117523 | 0.745021 |
| 0.322972 | 0.060848 | 2.286745 | 1.652446 | 57.95018 | 10.99593 | 118.6134 | 0 | 0.03243 |
| 0 | 0 | 0.187959 | 0.139736 | 17.49643 | 0.161002 | 11.76329 | 0 | 0.27915 |
| 0.186917 | 0.264113 | 0.518006 | 0.236272 | 268.3846 | 0.98639 | 1.900982 | 0 | 0.187688 |
| 0.101869 | 0.07197 | 0.333161 | 2.299409 | 1241.621 | 0.476882 | 2.343386 | 0 | 0.025572 |
| 0.370654 | 0.494635 | 1.889219 | 0.66932 | 25.81985 | 4.136332 | 3.141345 | 0 | 0.034461 |
| 0.058083 | 0.123106 | 1.127261 | 0.314655 | 60.4459 | 2.52131 | 0.879038 | 0 | 0.048602 |
| 0.089409 | 0.078959 | 0.192375 | 0.363268 | 12.12617 | 1.331756 | 0.243563 | 0 | 0.123444 |
| 0.243193 | 0.020214 | 0.373056 | 1.020386 | 5.465777 | 1.22979 | 0.866007 | 0 | 0.038305 |
| 2.092318 | 1.396093 | 2.896232 | 1.784184 | 108.5775 | 36.70598 | 29.48415 | 0.181049 | 0 |
| 0.762332 | 0.275861 | 4.887184 | 0.386121 | 89.88084 | 20.28878 | 3.331741 | 0 | 0.444971 |
| 0.795262 | 0.168555 | 1.782984 | 1.84894 | 48.01176 | 10.25488 | 5.873396 | 0 | 0.961575 |
| 0 | 0.104971 | 0.538679 | 0.268303 | 303.5647 | 0.758786 | 0.831997 | 0 | 0.270412 |
| 3.79051 | 0.847117 | 7.393492 | 0.174613 | 3.265901 | 37.1025 | 11.80107 | 0 | 0.012946 |
| 0.098918 | 0.029951 | 0.547293 | 3.406629 | 12.58816 | 0.408945 | 1.112092 | 0.058693 | 0.011825 |
| 0 | 0.794608 | 1.451989 | 0.507748 | 395.3014 | 3.82922 | 4.085199 | 1.167863 | 1.411689 |
| 0.190287 | 0.328624 | 0.802482 | 2.271692 | 35.97275 | 1.871579 | 5.708491 | 0.021954 | 0.02123 |
| 1.300886 | 0.59659 | 9.259412 | 1.44244 | 169.6297 | 9.343661 | 18.76219 | 0.687245 | 0.087847 |
| 0.045167 | 0 | 0.287663 | 0.367029 | 34.99127 | 0.038444 | 0.437483 | 0 | 0.211649 |
| 0.11052 | 0 | 1.299966 | 0.465674 | 10.18736 | 0.43899 | 2.899222 | 0 | 0.086314 |
| 0.075991 | 0 | 0.210594 | 0.439112 | 2.149494 | 0.996067 | 0.588829 | 0 | 0.104282 |
| 0.110014 | 0.019431 | 0.165697 | 0.223493 | 19.1009 | 0.046819 | 0.899083 | 0 | 0.211729 |
| 0.086978 | 0 | 0.387391 | 1.079811 | 4.17698 | 1.887806 | 0.737152 | 0 | 0.229258 |
| 0.39602 | 0.165329 | 1.591089 | 0.178782 | 193.9062 | 0.582218 | 5.187055 | 0.037383 | 0.150626 |
| 1.037792 | 0.422998 | 6.375945 | 3.856171 | 291.194 | 11.61906 | 4.736009 | 0.621695 | 0.0334 |
| 0.260547 | 0.210372 | 0.550992 | 3.59589 | 31.19244 | 3.674963 | 4.371277 | 0.154595 | 0.024916 |
| 0.103842 | 0.256774 | 1.322706 | 0.140637 | 34.08585 | 8.440793 | 20.55603 | 0 | 0.82547 |
| 0.293812 | 0.088962 | 1.221004 | 0.227383 | 195.8099 | 0.464432 | 6.403106 | 0.348666 | 0.224779 |
| 0.929061 | 0.145862 | 5.315129 | 4.380612 | 3677.851 | 22.4931 | 11.56093 | 0 | 0.017276 |
| 0.611534 | 0.172819 | 1.96142 | 1.325156 | 846.0547 | 7.460628 | 3.257786 | 0 | 0.170571 |
| 0.162039 | 0.11448 | 2.276294 | 0.308863 | 60.46704 | 0.291164 | 2.179849 | 0.11217 | 0.316375 |
| 0.124388 | 0.213422 | 1.567595 | 0.224618 | 97.97722 | 1.225105 | 0.968149 | 0.036903 | 0.06245 |
| 0 | 0.034954 | 0.285292 | 0.84874 | 28.92925 | 0.210554 | 0.299506 | 0.154119 | 0.082798 |
| 0.06177 | 0.02182 | 2.406957 | 1.101496 | 17.34217 | 22.02937 | 11.55469 | 0.03207 | 0.3308 |
| 0.666376 | 0.886197 | 1.949969 | 1.097153 | 86.71677 | 1.101012 | 1.09156 | 0 | 0.459202 |
| 0 | 0.020101 | 0.189777 | 3.532245 | 16.00595 | 0.024217 | 0.568389 | 0 | 0.257124 |
| 0.54452 | 0.096175 | 1.023207 | 1.638809 | 115.7101 | 2.433218 | 5.603797 | 0 | 0.045564 |
| 0.389167 | 0.17675 | 2.323039 | 0.276081 | 8.977817 | 15.63929 | 11.37559 | 0.028864 | 1.423523 |
| 0.315643 | 0 | 0.651985 | 0.783727 | 234.7032 | 0.940313 | 3.152825 | 0.163876 | 0.924421 |
| 3.83837 | 0.228706 | 8.88367 | 3.423875 | 1211.761 | 33.61509 | 32.58675 | 0.04802 | 0.007739 |
| 0.264452 | 0.186834 | 0.899027 | 2.825458 | 173.4253 | 6.677637 | 2.508085 | 0 | 0.044257 |
| 0.094424 | 0.022237 | 0.785578 | 0.269973 | 50.09617 | 4.045259 | 4.782497 | 0 | 0.131685 |

| | | | | | | | | |
|---|---|---|---|---|---|---|---|---|
| 0.221553 | 0.091307 | 0.754779 | 3.717379 | 69.64543 | 2.34148 | 3.442442 | 0.019171 | 0.037078 |
| 0.016618 | 0 | 0.1559 | 0.750232 | 0.877004 | 0.183883 | 0.261567 | 0 | 0.845471 |
| 0.264294 | 0.021967 | 0.445565 | 1.487917 | 217.2208 | 0.463141 | 1.93876 | 0.080715 | 0.153506 |
| 0.121914 | 0.183029 | 0.906294 | 0.577892 | 14.05771 | 2.477438 | 5.03702 | 0.047471 | 0.112215 |
| 0.166929 | 0.117935 | 1.537254 | 0.904314 | 26.25187 | 3.196853 | 3.94109 | 0.26 | 0.027936 |
| 0.312708 | 0.073642 | 1.75535 | 0.188227 | 185.3416 | 7.245526 | 18.00484 | 0.036078 | 0.075592 |
| 0.35252 | 0 | 0.509709 | 1.814237 | 154.2605 | 2.070335 | 6.743577 | 0.036604 | 0.507362 |
| 1.608522 | 0.172933 | 3.700217 | 26.80478 | 43.10757 | 3.363221 | 0.867906 | 0 | 0 |
| 0.065397 | 0.023102 | 0.8105 | 0.78237 | 26.26376 | 1.962129 | 1.662759 | 0.016977 | 0.019153 |
| 0.042706 | 0.030172 | 0.474144 | 1.388127 | 34.43706 | 0.375612 | 5.808303 | 0 | 0.011912 |
| 0.36122 | 0.340268 | 2.242822 | 0.900775 | 23.16567 | 17.10028 | 6.643436 | 0 | 0.054694 |
| 0.060525 | 0.149663 | 0.239621 | 2.021968 | 46.2506 | 0.309098 | 1.025923 | 0 | 0.086098 |
| 0.268986 | 0.042231 | 1.481627 | 0.512715 | 599.6675 | 0.788599 | 5.138361 | 0.279305 | 0.275096 |
| 1.699569 | 0.455017 | 2.654495 | 5.588904 | 31.72239 | 25.91686 | 12.7146 | 0 | 0.011976 |
| 0.986123 | 1.24193 | 11.29156 | 2.051702 | 30.31931 | 20.25367 | 4.464274 | 0.356158 | 0.050227 |
| 1.157973 | 0.629311 | 3.461325 | 6.836115 | 84.33135 | 24.71612 | 10.89246 | 0.046246 | 0.007454 |
| 0.250496 | 0.38518 | 2.909209 | 0.372516 | 37.69868 | 3.925582 | 7.153948 | 0 | 0.456204 |
| 0.076134 | 0.040341 | 1.020556 | 0.653038 | 91.40546 | 0.145804 | 3.111017 | 0.197637 | 0.098745 |
| 0.041806 | 0.073839 | 0.768184 | 1.528721 | 10.01625 | 6.831981 | 4.302353 | 0 | 2.259829 |
| 0.496963 | 0.058517 | 0.805528 | 0.299136 | 86.89618 | 4.265165 | 3.961131 | 0 | 0.138614 |
| 0.217266 | 0.306995 | 0.493968 | 6.195619 | 28.62619 | 2.712255 | 8.856066 | 0.0188 | 0.090901 |
| 0.728845 | 0.147122 | 1.268011 | 2.115217 | 108.0269 | 7.931726 | 20.98943 | 0 | 0.052275 |
| 0 | 0.189948 | 0.397228 | 0.24275 | 31.97137 | 0.83908 | 0.108506 | 0 | 0.037495 |
| 0.287837 | 0.061007 | 0.756809 | 0.246891 | 6.744352 | 0.428738 | 0.609864 | 0.104608 | 0.077073 |
| 0.950724 | 0.411235 | 2.985764 | 17.37823 | 10.03451 | 3.996515 | 25.88746 | 0 | 0.045459 |
| 0.029756 | 0.07358 | 0.119087 | 0.241801 | 5.213503 | 0.379909 | 0.144108 | 0 | 0.393407 |
| 0.487829 | 0.501309 | 4.141278 | 0.760789 | 24.90166 | 3.397233 | 6.926506 | 0 | 0.029687 |
| 0.120583 | 0.063894 | 1.08191 | 0.408274 | 52.94444 | 4.952113 | 7.628184 | 0.031302 | 0.075675 |
| 0.050712 | 0.011943 | 0.25751 | 0.473138 | 23.44378 | 0.115104 | 0.204663 | 0.035105 | 0.345133 |
| 1.694687 | 0.112952 | 2.052966 | 0.115481 | 17.95422 | 0.353806 | 1.471114 | 0.033202 | 0.133779 |
| 1.830729 | 1.892201 | 28.09285 | 1.913135 | 1564.2 | 10.56134 | 9.481815 | 0 | 0.002837 |
| 0 | 0.016288 | 0.119054 | 0.228977 | 3.698699 | 0.294349 | 0.446615 | 0 | 0.744657 |
| 0.220379 | 0.81185 | 5.191561 | 0.966467 | 738.1774 | 3.778329 | 8.176164 | 0.800919 | 0.015806 |
| 0.077729 | 0.010983 | 0.430825 | 0.22458 | 105.4054 | 0.251407 | 0.489371 | 0.129138 | 0.595781 |
| 0.093676 | 0.1456 | 1.217404 | 1.725417 | 62.09113 | 5.676964 | 11.18291 | 0.019454 | 0.125416 |
| 0.034755 | 0.098218 | 0.632644 | 0.815883 | 264.5554 | 2.55884 | 5.344074 | 0.018044 | 0.200666 |
| 0.151088 | 0.053372 | 0.416112 | 5.115606 | 70.80353 | 0.385798 | 2.240866 | 0 | 0 |
| 0.169488 | 0.203562 | 0.886894 | 0.275452 | 139.9616 | 1.312767 | 2.380378 | 0.651163 | 0.03971 |
| 0.078208 | 0.176811 | 0.469822 | 0.776742 | 67.65378 | 1.278079 | 3.370916 | 0 | 0.238207 |
| 0.272759 | 0.032117 | 1.134634 | 0.738816 | 96.9217 | 2.669838 | 4.51327 | 0 | 0.045647 |
| 0.534057 | 0.209616 | 3.115331 | 1.821626 | 88.18386 | 4.192091 | 4.454367 | 0.123232 | 0.109238 |
| 0.136914 | 0.096729 | 1.217636 | 1.071359 | 116.4727 | 2.253004 | 29.61695 | 0.047389 | 0.091652 |
| 0.407748 | 0.682277 | 1.414804 | 6.103572 | 105.4159 | 7.653496 | 6.365808 | 0 | 0.017957 |
| 0.062652 | 0.044263 | 1.907477 | 0.353548 | 49.19804 | 8.585527 | 2.958335 | 0 | 0.922681 |
| 0.061052 | 0.05176 | 0.260622 | 0.429962 | 13.73818 | 0.342967 | 0.709612 | 0 | 0.198215 |
| 0.223543 | 0.631728 | 2.46813 | 0.461336 | 56.62562 | 4.26746 | 1.701234 | 0 | 0.368762 |
| 0.164478 | 0.023241 | 0.662497 | 0.267311 | 80.68933 | 4.269883 | 5.814879 | 0.017079 | 0.068815 |
| 0.057576 | 0.14915 | 0.728433 | 0.381224 | 93.62528 | 0.441055 | 3.043984 | 0.039857 | 0.054602 |
| 0.067025 | 0 | 0.334576 | 1.210325 | 78.09931 | 5.020269 | 1.257818 | 0 | 0.190687 |
| 0.035532 | 0.012552 | 0.266053 | 0.4331 | 31.01457 | 0.045365 | 1.204554 | 0.166028 | 0.903853 |
| 1.06484 | 1.253843 | 2.484626 | 17.94678 | 87.41407 | 15.1057 | 8.308433 | 0 | 0 |
| 0.118793 | 0.069939 | 0.816217 | 0.536287 | 74.10044 | 5.965563 | 0.910906 | 0 | 0.086149 |
| 0.183008 | 0.284448 | 0.45992 | 0.462661 | 775.7196 | 2.056133 | 6.647222 | 0 | 0.030627 |
| 0.611968 | 0.576471 | 1.528762 | 1.544279 | 44.15353 | 7.305649 | 8.872174 | 0.032587 | 0.084033 |
| 0.291369 | 0.121089 | 0.777382 | 1.021347 | 34.11752 | 0.743999 | 14.40134 | 0.053391 | 0.243808 |
| 0.14941 | 0.211115 | 0.842266 | 0.382219 | 148.7597 | 2.67058 | 3.648061 | 0 | 0.183365 |
| 0.234747 | 0.189541 | 1.243968 | 0.999198 | 46.0845 | 4.338641 | 8.891927 | 0.069644 | 0.005612 |
| 0.218496 | 0.09262 | 0.597999 | 0.789112 | 151.467 | 0.63231 | 0.105816 | 0 | 0.212083 |

| | | | | | | | | |
|---|---|---|---|---|---|---|---|---|
| 0.150241 | 0.409415 | 1.339236 | 0.232545 | 63.42918 | 2.283533 | 5.015292 | 0 | 0.247841 |
| 0.019674 | 0 | 0.343734 | 2.025058 | 110.5546 | 0.167458 | 27.70297 | 0 | 0.174505 |
| 0.306272 | 0.463671 | 1.943067 | 0.118513 | 105.7513 | 5.474363 | 3.655165 | 0 | 0.153767 |
| 0.278092 | 0.024559 | 0.412864 | 0.219701 | 33.02039 | 2.574099 | 3.114435 | 0 | 0.151254 |

| MUC5AC | R3HDML | OBP2B | RBP2 | IDO1 | SEMG1 | RARRES3 | CHIT1 | LEP |
|---|---|---|---|---|---|---|---|---|
| 0.006856 | 0.310244 | 0 | 1.739157 | 1.938867 | 0.133662 | 54.31662 | 0.028738 | 0.469677 |
| 0.311607 | 0.528524 | 0 | 1.621223 | 1.450644 | 0.15455 | 22.59164 | 0.526131 | 0.012518 |
| 0.00088 | 0.111511 | 0 | 412.8235 | 1.219551 | 0.018478 | 80.47508 | 0.005959 | 0.853058 |
| 0.016627 | 0.200152 | 0 | 0.89266 | 3.002564 | 0.017456 | 59.70907 | 0.118223 | 0 |
| 0.323316 | 0.729558 | 0 | 1.310813 | 0.987389 | 0.108492 | 31.8666 | 5.338412 | 0.451912 |
| 0.009467 | 0.323881 | 0 | 1.251063 | 0.617967 | 0.049693 | 12.70317 | 0.170949 | 0.014489 |
| 0.087219 | 0.177841 | 0 | 1.987245 | 0.606128 | 0.073672 | 59.49129 | 0.020366 | 0.040917 |
| 0.011803 | 0.470033 | 0 | 1.410061 | 0.816465 | 0 | 19.79438 | 0.038059 | 0.055915 |
| 0.561932 | 0.123235 | 0 | 0.55784 | 1.578477 | 1.077752 | 21.20642 | 6.580952 | 0.159882 |
| 0.011511 | 0.145854 | 0 | 2.772949 | 2.025584 | 0.107416 | 82.95783 | 0.049077 | 0.019575 |
| 4.029054 | 0.204892 | 0 | 0.389537 | 27.39025 | 0.311221 | 70.49785 | 0.051707 | 2.46113 |
| 0.029356 | 0.672403 | 0.017357 | 0.676094 | 1.099478 | 0.213357 | 20.07913 | 0.211526 | 1.00228 |
| 0.004048 | 0.400037 | 0 | 0.317589 | 2.260938 | 0.033994 | 28.57493 | 0.012791 | 0.033039 |
| 0.043456 | 0.395764 | 0 | 5.327699 | 1.177083 | 0 | 20.66634 | 0.524155 | 0.228629 |
| 0.011337 | 0.287306 | 0 | 2.842579 | 2.170737 | 0.093515 | 25.99615 | 0.126122 | 0.012394 |
| 0.014742 | 0.669372 | 0.018886 | 0.608818 | 1.275216 | 0.025795 | 18.5807 | 0.077645 | 0.018803 |
| 0.034128 | 0.731825 | 0.060536 | 1.355195 | 0.948138 | 0.124022 | 23.57279 | 0.026666 | 0.006697 |
| 0.006806 | 0.137978 | 0 | 0.590223 | 1.092431 | 0.01429 | 20.89733 | 0.159764 | 0.715262 |
| 0.004658 | 0.619717 | 0 | 2.260213 | 1.881631 | 0.0489 | 15.29706 | 0.031541 | 0.356454 |
| 0.024069 | 0.762442 | 0.033637 | 1.287645 | 1.925852 | 0.12634 | 26.42155 | 0.466724 | 0.039071 |
| 0.014372 | 0.572357 | 0 | 1.377859 | 1.340254 | 0.107775 | 22.7246 | 0.064882 | 0.246161 |
| 0.068915 | 0.80854 | 0 | 2.055432 | 0.969623 | 0.026796 | 26.12408 | 0.538676 | 0.097663 |
| 0.049857 | 0.08537 | 0 | 1.91982 | 1.44915 | 0.070731 | 43.81813 | 6.530114 | 0.048122 |
| 0.021984 | 0.386888 | 0 | 1.033967 | 7.528025 | 0.012822 | 155.2384 | 0.151622 | 0.056078 |
| 0.26028 | 0.188948 | 0 | 0.643807 | 0.725327 | 0.128084 | 15.65935 | 0.027539 | 0.006916 |
| 1.359726 | 0.314171 | 0 | 1.146808 | 0.711072 | 0.458122 | 9.391596 | 0.154465 | 3.450783 |
| 0.007246 | 0.128533 | 0 | 1.466192 | 1.651506 | 0.04564 | 22.81234 | 0.042522 | 0.133075 |
| 0.032221 | 0.185581 | 0 | 2.379044 | 2.382271 | 0.061503 | 111.6348 | 0.180722 | 0.004981 |
| 0.008649 | 0.383559 | 0 | 0.476222 | 2.267477 | 0 | 11.73302 | 0.048804 | 0.051478 |
| 0.015734 | 0.25801 | 0 | 1.471572 | 1.356898 | 0.068017 | 16.87012 | 0.079387 | 0.254985 |
| 0.022957 | 0.183155 | 0 | 1.615232 | 1.178291 | 0.035705 | 59.04528 | 0.134345 | 0.425112 |
| 0.001102 | 0.530552 | 0 | 1.046597 | 1.414943 | 0.011568 | 17.4643 | 0.009949 | 0.044972 |
| 0.449437 | 0.516198 | 0 | 1.248216 | 5.554754 | 0.165554 | 107.9207 | 0.302558 | 0 |
| 0.022149 | 0.779577 | 0 | 1.778541 | 0.862468 | 0.012918 | 21.55218 | 0.111097 | 0.018833 |
| 0.006379 | 0.282892 | 0 | 0.636614 | 24.03317 | 0.011161 | 208.6012 | 0.009599 | 0 |
| 0.131785 | 0.549828 | 0 | 4.114927 | 2.192737 | 0.067488 | 54.13967 | 0.116083 | 0.024598 |
| 0.005882 | 0.029811 | 0 | 0.242898 | 0.427995 | 0 | 27.99851 | 0.015931 | 0.030007 |
| 0.001492 | 0.396958 | 0 | 0.492859 | 1.668227 | 0 | 22.54255 | 0.114488 | 3.942424 |
| 0.009252 | 0.234471 | 0 | 0.883584 | 2.042047 | 0.012141 | 39.53495 | 0.00261 | 0.011801 |
| 0.021054 | 0.242518 | 0 | 1.225131 | 1.259598 | 0.120559 | 28.15027 | 0.017281 | 0.185526 |
| 0.066028 | 0.08715 | 0 | 0.340844 | 83.37461 | 0.086647 | 205.8109 | 0.034154 | 0.203518 |
| 0.008978 | 0.442411 | 0 | 0.782743 | 1.078183 | 0.010473 | 19.65681 | 0.031524 | 0.111967 |
| 0.038866 | 2.097573 | 0.044258 | 0.17834 | 4.035838 | 0.120896 | 15.83536 | 0.422393 | 0.022032 |
| 0.076957 | 5.370911 | 0.037557 | 3.077199 | 0.712407 | 0.025648 | 8.646769 | 0.057902 | 0.006232 |
| 0.114472 | 0.063064 | 0.038255 | 0.154153 | 33.51346 | 6.661867 | 140.2867 | 0.162894 | 0.012696 |
| 0.162586 | 1.520571 | 0.014877 | 2.877563 | 11.96653 | 0 | 41.44081 | 0.126697 | 0.009875 |
| 0 | 2.005331 | 0 | 1.089286 | 9.116968 | 0 | 4.003483 | 0.178612 | 0.134568 |
| 0.053427 | 2.030927 | 4.330584 | 4.81392 | 0.502424 | 0 | 3.370665 | 0.244843 | 0.049559 |
| 0.205391 | 4.272043 | 0.178721 | 2.480592 | 5.494717 | 0 | 29.40963 | 0.122461 | 0 |
| 47.67342 | 1.576011 | 4.090317 | 4.843906 | 0.395925 | 0.3471 | 5.28685 | 1.570757 | 0 |
| 0.226877 | 0.104748 | 0.01412 | 0.550021 | 0.860053 | 0.048214 | 25.27965 | 0 | 0.009372 |
| 0.042253 | 0.142771 | 0.043303 | 0.756136 | 1.974392 | 0.532298 | 75.10422 | 1.347936 | 0.14371 |
| 24.78039 | 0.110548 | 0.178825 | 0.080065 | 0.254148 | 8.243201 | 27.23921 | 0.282262 | 0.014837 |
| 13.48876 | 1.769706 | 0 | 5.691884 | 30.44189 | 2.199355 | 40.13589 | 0.846198 | 0.131257 |
| 0.080603 | 0.519947 | 0.878625 | 0.030261 | 7.951 | 0.061541 | 29.95988 | 0.172012 | 0 |
| 1.139566 | 2.340773 | 1.779191 | 1.516605 | 1.857757 | 0 | 11.3304 | 0.306454 | 0.545042 |

| | | | | | | | | |
|---|---|---|---|---|---|---|---|---|
| 4.885748 | 1.011686 | 0 | 2.029083 | 1.478762 | 3.094901 | 55.31287 | 0.034657 | 0 |
| 0.031812 | 1.302309 | 2.144261 | 1.970637 | 5.053999 | 0 | 16.07305 | 0.011047 | 0 |
| 1.70928 | 0.50228 | 0.024375 | 0.556586 | 4.114632 | 0 | 35.54571 | 0.085896 | 0.016179 |
| 0.048275 | 3.128352 | 0.021203 | 13.84127 | 3.446375 | 0.02896 | 13.61679 | 0.112078 | 0.056294 |
| 0.066199 | 0.660876 | 0.030838 | 2.15391 | 3.177033 | 0.189536 | 26.02848 | 0.013584 | 0 |
| 5.0051 | 0.141142 | 0.228314 | 1.073346 | 9.337177 | 0.03898 | 62.98872 | 0.243045 | 0.056828 |
| 0.02393 | 1.010727 | 0 | 0.109804 | 6.216947 | 0.027914 | 27.28542 | 0.018005 | 0 |
| 0.038986 | 1.879907 | 0.149829 | 1.855971 | 0.863347 | 0.079582 | 6.24019 | 0.024444 | 0 |
| 0.282183 | 0.124522 | 0.302144 | 0.028988 | 24.2999 | 0.08843 | 83.04609 | 0.256677 | 0.014325 |
| 0.14829 | 0.599916 | 0.151059 | 11.62069 | 0.965492 | 0.994084 | 15.02254 | 0.104851 | 0.01823 |
| 0.381436 | 0.035023 | 0.042491 | 2.368533 | 0.022183 | 0.85601 | 27.32478 | 0.006239 | 0.070507 |
| 2.204989 | 0.229325 | 0 | 1.494821 | 4.034818 | 0 | 8.670405 | 0.394897 | 0 |
| 0.02712 | 0.343634 | 0 | 48.78061 | 1.257569 | 0 | 10.92805 | 0.12923 | 0 |
| 0.01021 | 0.491601 | 0.141257 | 6.598589 | 0.344154 | 1.607769 | 18.74661 | 0.006914 | 0.010418 |
| 0.036196 | 0.229323 | 0 | 2.802757 | 0.548729 | 0.063333 | 8.184117 | 0.156595 | 0 |
| 0.049486 | 3.319585 | 0.671229 | 2.284032 | 11.32293 | 0 | 40.50661 | 2.095977 | 0.074254 |
| 0.079846 | 1.16162 | 0.181845 | 2.747844 | 0.474686 | 0.31046 | 9.294864 | 0.0267 | 0 |
| 0.023379 | 0.052277 | 0.063423 | 0 | 52.52266 | 0.028875 | 32.39973 | 0.173832 | 0 |
| 0.063223 | 0.179838 | 0 | 0.372987 | 8.380882 | 0.216726 | 42.25191 | 9.872838 | 0.065826 |
| 0.123145 | 5.566726 | 0.102328 | 2.748926 | 0.267116 | 0.349405 | 2.548153 | 0.338059 | 0 |
| 0.099762 | 0.649125 | 0 | 3.062065 | 9.954207 | 0 | 31.9062 | 2.641305 | 0 |
| 0.186707 | 0.03943 | 0 | 0.192761 | 10.73901 | 0.52269 | 21.14934 | 0.133453 | 0.031751 |
| 2.855242 | 0.134996 | 0 | 0.219987 | 5.244339 | 6.151562 | 75.91541 | 0.03006 | 0 |
| 0.064506 | 1.268321 | 0.017097 | 1.699401 | 0.362994 | 0.945747 | 5.975464 | 0.228446 | 0 |
| 0.148573 | 0.202738 | 0 | 6.843562 | 1.675486 | 0 | 11.72803 | 0.07739 | 0 |
| 0.928705 | 2.323355 | 1.024993 | 8.211427 | 1.987617 | 0.074998 | 14.10549 | 0.091375 | 0.024298 |
| 0.322779 | 0.461767 | 0 | 31.71206 | 1.337056 | 0 | 10.27939 | 0.164516 | 0 |
| 5.443866 | 0.132799 | 0 | 0.958372 | 1.578137 | 2.090498 | 5.208585 | 0.473127 | 0.053469 |
| 0.174816 | 0.417336 | 0 | 0.156943 | 16.23987 | 0.026598 | 13.6462 | 0.068624 | 0 |
| 0.47588 | 2.176107 | 0 | 1.466007 | 2.689934 | 0.43311 | 7.693542 | 0.015159 | 0 |
| 0.274952 | 4.007825 | 0.222461 | 0.128061 | 0.254407 | 0 | 6.69205 | 0.144655 | 0 |
| 0.021351 | 1.855123 | 0.257889 | 14.10772 | 0.82415 | 1.64908 | 8.603509 | 0.399314 | 0.015561 |
| 0.846889 | 0.048229 | 0 | 0.196483 | 17.51419 | 0.399587 | 52.40311 | 4.974408 | 0.145639 |
| 1.500606 | 0.550717 | 0.017583 | 0.023617 | 0.587488 | 0.060037 | 13.62677 | 0.025817 | 0.035011 |
| 0.087188 | 3.874428 | 0.018878 | 9.255042 | 0.643899 | 1.469657 | 3.249371 | 0.019403 | 0.056384 |
| 0 | 1.217928 | 4.888879 | 24.57619 | 1.28254 | 0.012458 | 8.160771 | 0.002678 | 0.024216 |
| 0.380956 | 1.421936 | 0 | 0.792715 | 1.343069 | 0.062005 | 10.43222 | 0.039995 | 0 |
| 0.007962 | 3.26601 | 0.076494 | 6.18531 | 5.68153 | 0 | 18.80774 | 2.598996 | 0.015232 |
| 8.531635 | 0.140532 | 0 | 0.057252 | 37.04403 | 8.76165 | 140.2561 | 0.513198 | 0 |
| 0.388443 | 2.812556 | 0 | 0.36184 | 1.062624 | 0 | 10.51349 | 0.02637 | 0.0149 |
| 0.073679 | 0.579467 | 0.019528 | 2.675489 | 0.958359 | 0.24005 | 43.86849 | 0.387092 | 0.038885 |
| 0.293843 | 1.861646 | 0 | 26.82078 | 2.679902 | 0.140219 | 18.38879 | 0.067833 | 0 |
| 0.58798 | 0.110466 | 0 | 7.740579 | 2.860932 | 0.010169 | 11.03331 | 0.010932 | 0 |
| 0.32781 | 3.5258 | 0.058597 | 3.699218 | 1.427631 | 0.120049 | 13.97727 | 0.008604 | 0 |
| 0.063826 | 3.953871 | 0.799483 | 0.439307 | 1.416608 | 0.012409 | 5.79477 | 0.277464 | 0.01809 |
| 0.031719 | 2.381671 | 0.018059 | 1.673741 | 0.075427 | 0 | 1.230782 | 1.83229 | 0.041953 |
| 61.95429 | 0.109754 | 0 | 0 | 3.224297 | 0.181867 | 56.47692 | 1.600413 | 0.025252 |
| 0.05867 | 0.69493 | 0.137249 | 1.659169 | 7.465747 | 0.053559 | 37.67236 | 0.089246 | 0.006507 |
| 25.01716 | 1.369027 | 0 | 16.60816 | 1.033067 | 0.115527 | 11.5561 | 2.818105 | 0.035727 |
| 0 | 5.212801 | 0 | 18.22927 | 1.250371 | 0 | 18.68094 | 0.309531 | 0.086934 |
| 0.213171 | 2.599683 | 0 | 2.659021 | 1.163926 | 0.160396 | 5.306349 | 0.016422 | 0 |
| 2.058583 | 0.118488 | 0 | 0.220671 | 5.131981 | 0 | 58.36954 | 1.151848 | 0.006815 |
| 8.276157 | 0.357909 | 0.017369 | 0.629903 | 1.759174 | 1.269169 | 13.56735 | 7.301441 | 0.023057 |
| 0.092255 | 0.58448 | 0 | 11.03988 | 0.617011 | 0 | 4.56233 | 0.156176 | 0.363692 |
| 0.092832 | 4.134416 | 0.367365 | 0.056936 | 1.829414 | 0.009649 | 29.55526 | 0.022821 | 0 |
| 0.24781 | 0.785002 | 0 | 0.222475 | 4.900103 | 0.084833 | 10.96252 | 0.072959 | 0.013742 |
| 5.355732 | 2.957692 | 0.099676 | 0.401651 | 0.936693 | 0.06807 | 57.7416 | 0 | 0 |
| 0.953657 | 0.063266 | 0.076755 | 0.257744 | 128.3785 | 1.074552 | 102.274 | 9.844369 | 0.076419 |

| | | | | | | | | |
|---|---|---|---|---|---|---|---|---|
| 0.081586 | 1.526049 | 1.313916 | 3.048369 | 0.582033 | 0 | 8.384117 | 0.038 | 0.006607 |
| 0.952751 | 0.085822 | 0 | 0 | 13.84352 | 0 | 35.11657 | 0.540181 | 0 |
| 1.272948 | 1.50217 | 0.045561 | 4.92643 | 0.340942 | 0.155572 | 4.418827 | 0.00669 | 0 |
| 0.106492 | 1.712654 | 0.346303 | 6.173846 | 0.109574 | 0.0215 | 18.40249 | 0 | 0.010448 |
| 0.005967 | 4.555454 | 0.504517 | 5.636933 | 0.375142 | 0 | 1.082015 | 0.006734 | 0.152213 |
| 0.144997 | 0.866138 | 2.16531 | 7.570482 | 0.919887 | 0.043492 | 17.66736 | 3.427137 | 0 |
| 0.633629 | 0.119832 | 0.436145 | 3.384786 | 2.934831 | 0.297849 | 33.74689 | 1.380406 | 0.016083 |
| 0.044816 | 0.585068 | 0.062631 | 12.05793 | 0.486839 | 0.028514 | 5.600031 | 0.03985 | 0.034642 |
| 0.325443 | 1.776359 | 1.000585 | 50.28558 | 0.578642 | 0.052562 | 19.81209 | 0.00226 | 0.020435 |
| 1.534063 | 0.766791 | 0.511656 | 3.6237 | 4.905381 | 1.270603 | 50.72083 | 0.150253 | 0 |
| 0.109401 | 2.18651 | 0 | 9.175577 | 0.488797 | 1.57476 | 3.196445 | 0.015274 | 0.005754 |
| 2.625843 | 0.835983 | 0.811382 | 0.3542 | 1.044894 | 0.152378 | 8.808635 | 0.01787 | 0.013464 |
| 6.98522 | 0.306847 | 0 | 3.075202 | 11.52525 | 0.203383 | 178.2488 | 3.533818 | 0.055596 |
| 6.130836 | 0.012729 | 0.015443 | 0.020743 | 2.424084 | 0.010546 | 8.122866 | 0.321981 | 0.015375 |
| 0.031784 | 0 | 0 | 0.656288 | 3.89436 | 0.033367 | 17.71701 | 0.071742 | 0.048646 |
| 0.149198 | 1.124726 | 1.30647 | 15.7546 | 0.626504 | 0.099134 | 6.164246 | 0.038366 | 0.009635 |
| 0.025021 | 0.055948 | 0.022626 | 2.735155 | 1.031613 | 3.584702 | 48.13777 | 0.089698 | 0 |
| 0.054362 | 2.263252 | 0 | 3.688164 | 1.537411 | 0.203821 | 1.251731 | 0.499579 | 0.633918 |
| 10.53905 | 0.507094 | 0.021214 | 0.199465 | 3.677078 | 2.462874 | 23.89285 | 0.121481 | 0.049283 |
| 0.00824 | 2.523165 | 0.190002 | 2.977448 | 0.683351 | 0 | 8.337596 | 0.024798 | 0 |
| 0.061708 | 0.750627 | 0.227668 | 4.79091 | 4.529916 | 0.103652 | 17.57169 | 2.139432 | 0.025186 |
| 6.298477 | 1.510035 | 0 | 6.197398 | 0.47232 | 0.046337 | 9.782098 | 1.514333 | 0.022518 |
| 2.301034 | 3.207923 | 0.951354 | 8.015633 | 1.806117 | 0 | 19.11863 | 0.050796 | 0 |
| 0.032644 | 3.834079 | 0.019301 | 1.296257 | 0.382915 | 0.158172 | 9.005546 | 0.025506 | 0.012811 |
| 0.057676 | 3.86489 | 0.255761 | 10.83285 | 0 | 0.058221 | 1.117353 | 0.060086 | 0.028293 |
| 0.045973 | 1.581147 | 0.040385 | 0 | 2.523052 | 0.124106 | 19.44263 | 6.792476 | 0 |
| 0.39597 | 0.236667 | 0 | 1.38841 | 4.127349 | 0.019608 | 11.81636 | 0.177069 | 0.009529 |
| 0.075649 | 0.563146 | 0.04361 | 2.362578 | 0.136606 | 1.369952 | 3.569066 | 0.021344 | 0.024121 |
| 0.112435 | 0.034468 | 0.557555 | 0 | 33.55274 | 0.028557 | 43.34537 | 3.587784 | 3.00222 |
| 0.762656 | 0.992084 | 3.387944 | 3.293249 | 0.543045 | 0.030443 | 5.725675 | 0.359999 | 0 |
| 0.382658 | 0.517192 | 0 | 2.370399 | 0.98276 | 0.026781 | 29.43402 | 1.848379 | 0.013015 |
| 0.317628 | 1.538847 | 0.017951 | 14.20216 | 0.474853 | 0.012259 | 18.27312 | 0.002636 | 0.005958 |
| 0.167476 | 0.287999 | 0.01839 | 6.422252 | 1.900975 | 0.200937 | 13.77027 | 0.361823 | 0.207503 |
| 0.158414 | 0.194252 | 0.039278 | 13.13682 | 0.46481 | 0 | 12.18335 | 1.095776 | 0 |
| 0.031063 | 0.337369 | 0 | 0.030543 | 1.733246 | 0.046586 | 14.00706 | 0.367265 | 0.007546 |
| 0.027584 | 1.198342 | 0.045433 | 4.047993 | 0.558726 | 0.031027 | 10.17561 | 0.796062 | 1.085603 |
| 0.156774 | 1.024283 | 0 | 10.82423 | 0.170386 | 0 | 2.59931 | 0 | 0.012497 |
| 0.314994 | 0.815741 | 0.777597 | 0.142427 | 12.00679 | 2.486189 | 19.88862 | 0.051898 | 0 |
| 0.589658 | 2.315749 | 0.106019 | 5.909776 | 0.707254 | 0 | 6.824038 | 0.630457 | 0.029321 |
| 0.1949 | 1.55025 | 0.043739 | 0.352502 | 0.144624 | 0 | 2.101943 | 0.016056 | 0 |
| 17.41543 | 0.117233 | 0 | 0 | 9.265335 | 4.872677 | 64.33162 | 0.031325 | 0.015734 |
| 0.428581 | 7.015988 | 4.819729 | 0.504841 | 6.856286 | 0 | 34.65575 | 0.727161 | 3.375155 |
| 0.033964 | 1.655234 | 0.281142 | 60.31265 | 1.63553 | 0 | 9.369694 | 0.058972 | 0.013329 |
| 0.355203 | 0.035162 | 0 | 0 | 18.84184 | 8.331965 | 341.9122 | 1.522086 | 0.028315 |
| 20.9222 | 0.14077 | 0.042696 | 0.172048 | 0.757887 | 3.877981 | 13.72852 | 0.012538 | 0.056679 |
| 0.046669 | 0 | 0 | 0.18355 | 45.12439 | 2.053062 | 178.83 | 0.922972 | 0.022675 |
| 0.135764 | 0.132328 | 0 | 0.898502 | 1.443499 | 0.191864 | 26.74561 | 0.068753 | 0 |
| 0.061399 | 1.778249 | 0.134838 | 6.640814 | 0.500593 | 0.030694 | 51.52406 | 0.013199 | 0 |
| 0.348742 | 0.343217 | 0.260248 | 3.915115 | 1.503628 | 0.497634 | 20.37427 | 0.099352 | 0 |
| 47.90489 | 0.022876 | 0 | 0.037278 | 8.452081 | 0.966597 | 25.02517 | 0.004075 | 0 |
| 0.171051 | 0.410046 | 0.035534 | 4.916083 | 3.642279 | 0.606663 | 9.717932 | 0.224351 | 0.076652 |
| 4.697786 | 0.139568 | 0.042332 | 0.05686 | 57.04852 | 1.503253 | 112.8478 | 2.119507 | 0.09834 |
| 0.044367 | 0.468484 | 0 | 0.076343 | 12.28484 | 0.349333 | 43.85107 | 0.258709 | 0 |
| 77.52037 | 0.123963 | 0 | 0.057717 | 1.375922 | 4.929866 | 18.35932 | 0.094639 | 0.00713 |
| 48.40599 | 0.072591 | 0 | 0.709755 | 2.298922 | 4.420487 | 35.55057 | 0.122845 | 0.058454 |
| 32.38618 | 0.031218 | 0 | 0.101746 | 428.3221 | 0.10346 | 398.0422 | 0.077856 | 0.050278 |
| 0.456846 | 0.871417 | 0.075516 | 3.144394 | 1.248461 | 0.54149 | 10.49108 | 0.471239 | 0 |
| 0.329397 | 1.897178 | 1.150843 | 5.874066 | 2.723767 | 0.157185 | 5.429959 | 0.033796 | 0 |

| | | | | | | | | |
|---|---|---|---|---|---|---|---|---|
| 0.118837 | 2.20616 | 3.653701 | 1.369572 | 0.63584 | 0 | 4.23181 | 0.212095 | 0.028199 |
| 0.67122 | 0.194401 | 0 | 1.504768 | 0.88245 | 0.161065 | 21.01956 | 0.259725 | 0 |
| 0.092932 | 0.519506 | 0 | 0.197535 | 3.531829 | 0 | 25.07522 | 0.638549 | 0 |
| 11.3709 | 0.352399 | 0 | 4.060872 | 1.349876 | 3.482782 | 12.86112 | 1.022342 | 0.111483 |
| 5.254558 | 0.858182 | 0.824252 | 5.943546 | 1.796794 | 0.177755 | 17.05711 | 0.038219 | 0.014397 |
| 7.48484 | 0.27253 | 0 | 0.055514 | 6.372529 | 10.58423 | 82.8767 | 0.036411 | 0.068581 |
| 0.188289 | 0.03787 | 0 | 0.061713 | 122.8599 | 2.761099 | 105.6993 | 2.469061 | 0 |
| 0.05173 | 0.327734 | 0 | 0.47473 | 1.783695 | 0.060341 | 7.064075 | 0.10379 | 0.131956 |
| 0 | 1.146487 | 0.079482 | 7.259674 | 2.005636 | 0 | 7.951723 | 0.163386 | 0 |
| 0.195705 | 5.101266 | 0.773617 | 6.696544 | 2.931938 | 0.029351 | 23.47039 | 0.283977 | 0 |
| 0.683928 | 1.229007 | 0.038232 | 17.40871 | 1.011315 | 0.104437 | 6.079751 | 0.673636 | 0.038064 |
| 0.215242 | 0.649365 | 0.157564 | 4.444424 | 2.138783 | 0.107602 | 9.086312 | 0.185082 | 0 |
| 0.213911 | 4.052928 | 0.062045 | 3.979424 | 10.57614 | 0.042371 | 30.03338 | 0.075158 | 0.015443 |
| 0.034456 | 3.573196 | 0.041817 | 1.685063 | 2.566465 | 0 | 7.283204 | 0.032747 | 0.013878 |
| 0.085492 | 0.440079 | 0.08214 | 0.717146 | 7.261622 | 0.028047 | 37.53559 | 0.078394 | 0.395269 |
| 1.88065 | 0.044227 | 0 | 0.072072 | 3.623072 | 0 | 17.51555 | 0.401806 | 0.032053 |
| 24.53468 | 0.045553 | 0 | 0.519633 | 1.558079 | 10.81306 | 6.808714 | 0.113607 | 0.009171 |
| 32.41935 | 0.125825 | 0 | 3.075634 | 18.18414 | 0 | 159.4892 | 6.242316 | 0.063326 |
| 39.53775 | 0.702687 | 0 | 2.394276 | 1.766825 | 7.250912 | 14.96533 | 0.193452 | 0 |
| 0.200397 | 1.13828 | 0.03541 | 31.58128 | 0.246489 | 0.024182 | 4.053037 | 0.025996 | 0 |
| 0.171937 | 0.877297 | 0 | 0.953088 | 3.309356 | 2.689376 | 25.60533 | 12.91127 | 0.158952 |
| 0.001793 | 0.511078 | 0 | 1.017921 | 7.759538 | 3.688614 | 41.81227 | 1.094526 | 0.155475 |
| 0.225201 | 0.481533 | 0 | 19.23966 | 3.870869 | 0.132987 | 15.50247 | 0.012708 | 0 |
| 0.035548 | 3.069628 | 0.101199 | 0.54372 | 3.4025 | 2.944086 | 12.07851 | 0.068352 | 0.006717 |
| 0.123102 | 0.704439 | 0 | 17.98444 | 13.84598 | 0.055585 | 30.84737 | 1.646266 | 0.08779 |
| 2.880024 | 0.265081 | 0.750402 | 8.351471 | 2.742384 | 1.244542 | 13.76892 | 0.141662 | 0 |
| 0.893194 | 0.129451 | 0 | 0.030136 | 6.536037 | 30.32177 | 36.30544 | 2.925319 | 0.044675 |
| 0.012434 | 0.458348 | 0.017377 | 4.598206 | 7.354645 | 1.044313 | 37.51825 | 0.944063 | 0.017301 |
| 0.190469 | 0.185649 | 0.028154 | 14.02986 | 30.26446 | 0.067294 | 120.7468 | 0.049606 | 0 |
| 0.126797 | 2.445525 | 0.224246 | 0.463395 | 4.664958 | 0.0589 | 16.45853 | 1.633645 | 0.400728 |
| 19.13964 | 0.28276 | 0.054166 | 0.315272 | 1.816124 | 0.542525 | 12.06843 | 0.076881 | 0.023968 |
| 1.136641 | 1.576112 | 0.19781 | 109.4674 | 2.340844 | 0.045029 | 14.46064 | 0.019363 | 0 |
| 0.060681 | 1.375906 | 0 | 1.187025 | 9.552233 | 0 | 27.62617 | 0.036044 | 0.464368 |
| 0.15497 | 0.972636 | 0.022264 | 0.687827 | 1.906306 | 0.152047 | 35.63893 | 0.098073 | 0.177335 |
| 0.861913 | 0.892495 | 0.028494 | 0.267915 | 2.191783 | 0.214051 | 18.82213 | 0.117148 | 0 |
| 11.85156 | 0.110073 | 0 | 5.483715 | 1.049117 | 0.469018 | 11.93498 | 0.151263 | 0.037988 |
| 0.03953 | 2.382602 | 0.361326 | 1.698661 | 2.783882 | 0 | 12.48484 | 1.709777 | 2.414629 |
| 0.017075 | 0.072117 | 0.262482 | 0.352564 | 3.989275 | 5.73606 | 30.50263 | 0.449637 | 0.058073 |
| 0.88521 | 0.19197 | 0.133086 | 8.446444 | 1.957063 | 0.090886 | 21.91952 | 0.068394 | 0 |
| 0.050628 | 7.698133 | 0.564261 | 17.53654 | 0.176076 | 0 | 3.077142 | 1.082774 | 0.006457 |
| 0.003056 | 0.851983 | 0 | 1.388379 | 0.67864 | 0.048129 | 3.756246 | 0 | 0.015593 |
| 0.264513 | 2.342547 | 0 | 1.11585 | 1.141351 | 0.029859 | 5.424854 | 0 | 0 |
| 0.147467 | 8.443789 | 0.042773 | 3.849319 | 0.49128 | 0.014605 | 5.194832 | 1.413087 | 0.007098 |
| 0.08241 | 6.545865 | 0.151268 | 0.406364 | 8.463342 | 0.012913 | 10.72228 | 0.677426 | 0.006275 |
| 0.040375 | 0.28859 | 0.047744 | 0.513035 | 22.43904 | 0.391259 | 44.45558 | 0.002337 | 0 |
| 2.261671 | 2.82887 | 0.198958 | 17.23697 | 0.571296 | 0 | 11.42995 | 0.102246 | 0 |
| 0.01893 | 0.879475 | 0 | 0.260578 | 2.599589 | 1.308281 | 11.65355 | 0.249243 | 0 |
| 0.194847 | 0.184247 | 0 | 5.344374 | 1.042527 | 0 | 10.78111 | 0.262569 | 0 |
| 0.006275 | 1.444452 | 63.60204 | 0.799016 | 2.355819 | 0 | 15.45234 | 0.042492 | 0.005336 |
| 0.236381 | 2.559 | 0.07056 | 12.98421 | 0.896383 | 0.048186 | 12.26861 | 0.196846 | 0.011708 |
| 0.065231 | 0.295196 | 0.047752 | 0.513117 | 1.861447 | 0.016305 | 18.48323 | 4.210338 | 0.103008 |
| 0.157818 | 0.521666 | 0 | 10.24841 | 0.881121 | 0.048023 | 6.144329 | 0.263296 | 0.011669 |
| 0.379283 | 0.775146 | 6.159747 | 0.442108 | 23.35396 | 0 | 204.8885 | 0.952768 | 0.06242 |
| 0.017969 | 0.106252 | 0.018415 | 5.367547 | 0.499939 | 0 | 7.9486 | 0.105453 | 0.006111 |
| 0.061333 | 2.447186 | 0.040121 | 15.03546 | 0.802946 | 0.082198 | 4.399505 | 0.002946 | 0.039945 |
| 0.096806 | 0.7937 | 1.225546 | 1.881312 | 0.822641 | 0.149454 | 7.304521 | 0.430589 | 0 |
| 1.528829 | 0.092688 | 0 | 0.755217 | 10.37178 | 0.844734 | 91.96553 | 1.353919 | 0.037319 |
| 0.216449 | 0.116707 | 0 | 0.285277 | 55.99582 | 5.463236 | 327.9383 | 0.14553 | 0.023495 |

| | | | | | | | | |
|---|---|---|---|---|---|---|---|---|
| 0.138173 | 0.656549 | 0.059003 | 6.498667 | 0.2567 | 0.100734 | 2.165246 | 0 | 0 |
| 0.168017 | 1.188246 | 0 | 17.34646 | 1.108037 | 0.205101 | 15.2726 | 0.176392 | 0.059803 |
| 0.005955 | 0.513142 | 0 | 3.910503 | 0.904961 | 0.037513 | 5.727513 | 0.588785 | 0.783891 |
| 0.853177 | 0.528781 | 0 | 2.680825 | 0.645045 | 2.920703 | 20.80197 | 0.146526 | 0 |
| 0.150201 | 0.322865 | 0 | 1.412259 | 1.757989 | 0.056316 | 36.33269 | 2.999818 | 0.21894 |
| 0.020565 | 0.028954 | 0 | 0.094365 | 1.369322 | 0 | 14.02637 | 20.57935 | 0.058288 |
| 2.775161 | 0.075492 | 0.018318 | 1.353226 | 6.362739 | 0 | 80.281 | 0.376542 | 0.024316 |
| 0.062107 | 4.262713 | 0.596722 | 1.816765 | 3.461512 | 0.040751 | 9.471832 | 0.36215 | 0.026405 |
| 0.096983 | 2.770984 | 0.321564 | 96.27952 | 1.098862 | 0.059891 | 52.89823 | 0.030046 | 0 |
| 79.37295 | 0.055846 | 0.022585 | 0.758387 | 0.628849 | 6.369808 | 89.58887 | 0.736175 | 0.029981 |
| 0.023467 | 0.726847 | 0 | 6.029975 | 3.836508 | 1.806621 | 78.13483 | 0.035312 | 0 |
| 0.899574 | 3.846559 | 0 | 2.185278 | 1.102709 | 0.029238 | 24.83927 | 0.427474 | 0.028417 |
| 0.013102 | 1.014525 | 0 | 9.588881 | 0.856793 | 0.015283 | 2.581064 | 2.18841 | 0 |
| 0.011649 | 0.563589 | 0.01628 | 3.126991 | 1.031259 | 0.055589 | 7.297188 | 0.009562 | 0 |
| 2.592682 | 0.102343 | 0 | 0.333552 | 30.25082 | 0.254378 | 51.20622 | 0.455775 | 0.041206 |
| 0.147984 | 1.646082 | 0.503605 | 47.28079 | 0.151104 | 0.201607 | 2.549105 | 0.010199 | 0.011526 |
| 0.126869 | 9.022016 | 0.039802 | 5.45316 | 1.967172 | 0 | 7.546671 | 0.11104 | 0 |
| 0.346683 | 2.30978 | 0 | 1.731895 | 4.930527 | 0.046962 | 12.1973 | 0.174175 | 0.011411 |
| 1.249845 | 1.805234 | 1.144845 | 2.473772 | 1.230048 | 0.067985 | 6.915166 | 3.398507 | 0.016519 |
| 0.009387 | 1.453678 | 0.016033 | 35.21037 | 9.374929 | 0 | 19.90648 | 8.022867 | 0.021284 |
| 0.086736 | 1.007442 | 0.074075 | 11.24325 | 31.28021 | 0.012647 | 63.68779 | 0.008157 | 0.006146 |
| 0.037205 | 1.269213 | 0.043995 | 0.945505 | 0.076563 | 0 | 3.036816 | 0.613684 | 0.058403 |
| 0.149062 | 1.427561 | 0.239807 | 3.221076 | 1.743501 | 0.018196 | 8.194995 | 0.133019 | 0.008843 |
| 0.460052 | 0.028436 | 0.396735 | 0.046338 | 393.3733 | 0 | 186.4357 | 0.346984 | 0.028623 |
| 0.67104 | 1.101573 | 0 | 5.890196 | 5.610915 | 0.057042 | 12.55297 | 0.042926 | 0.01386 |
| 7.150028 | 0.797096 | 4.160135 | 0.26959 | 37.31002 | 0.124606 | 58.62096 | 2.813055 | 0.030277 |
| 2.196843 | 4.806259 | 0.18222 | 5.547817 | 0.486236 | 0.12444 | 8.544499 | 0.222962 | 0.040316 |
| 0.060324 | 1.791876 | 0.212833 | 0.694269 | 18.21763 | 0.145346 | 35.77116 | 1.183047 | 0.060543 |
| 0.000894 | 3.838467 | 0.068686 | 34.33854 | 4.260092 | 0.009381 | 54.49276 | 0.078664 | 0.031913 |
| 0.017816 | 1.612472 | 0.365172 | 1.331349 | 0.54471 | 0 | 16.13574 | 0.00766 | 0.012985 |
| 0.013528 | 0.299971 | 0 | 0.395718 | 10.86316 | 9.349519 | 73.39341 | 0.857519 | 0.063264 |
| 0.285719 | 0.809817 | 0.057793 | 3.648488 | 11.365 | 0.118403 | 135.0281 | 1.6123 | 0.01918 |
| 0.185554 | 0.957881 | 14.12148 | 4.257136 | 0.441249 | 0 | 6.584705 | 0.036195 | 0.023374 |
| 0.084105 | 0.791663 | 0.110822 | 0.099237 | 10.768 | 2.611021 | 182.1386 | 0.642747 | 0 |
| 0.0474 | 2.117403 | 0.024701 | 8.543279 | 2.170774 | 1.771179 | 18.8736 | 0.014507 | 0 |
| 0.008435 | 0.534396 | 0.187297 | 1.296589 | 8.354269 | 2.607349 | 34.41705 | 3.450315 | 1.831289 |
| 0.861189 | 0.822741 | 0.078802 | 0.088206 | 0.443409 | 2.968797 | 36.35462 | 0.385686 | 0.03487 |
| 1.110358 | 0.113988 | 0.019756 | 0 | 2.324131 | 3.791142 | 23.35519 | 0.417713 | 0 |
| 0.062393 | 1.718668 | 0 | 2.212567 | 6.437227 | 0 | 10.05671 | 0.587825 | 0 |
| 0.755174 | 1.356773 | 0.086635 | 4.421957 | 6.724217 | 0 | 12.18721 | 0.572428 | 0.028752 |
| 43.07209 | 0.698056 | 0.259532 | 0.605466 | 3.261415 | 0.447758 | 10.98023 | 0.094265 | 0 |
| 0.099113 | 0.355829 | 0 | 12.10873 | 2.032843 | 0 | 27.3134 | 0.074572 | 0 |
| 0.302947 | 0.084058 | 0.033993 | 0.09132 | 7.903456 | 0.905368 | 11.22727 | 0.009983 | 0 |
| 0.024142 | 0.35865 | 0.025595 | 2.337805 | 2.369665 | 0.017479 | 24.79035 | 0.516749 | 1.061793 |
| 0.626962 | 1.817311 | 0.188982 | 5.372942 | 8.254853 | 0.02151 | 47.25266 | 1.789768 | 0.020906 |
| 0.005745 | 1.868386 | 3.532606 | 19.55327 | 1.583024 | 0.010052 | 7.767972 | 0.015129 | 0.146546 |
| 0.091701 | 0.973982 | 0 | 4.761562 | 0.129877 | 0 | 1.992478 | 0.024351 | 0.00688 |
| 6.02539 | 0.543186 | 0 | 1.229401 | 2.675955 | 0.050005 | 38.83469 | 0.080635 | 0.01215 |
| 0.889375 | 5.518704 | 0.281319 | 2.191621 | 1.899525 | 0.691617 | 10.66779 | 0.016522 | 0 |
| 2.652234 | 0.420959 | 0.085119 | 9.260839 | 22.87118 | 5.289716 | 34.21139 | 0.112483 | 0.028249 |
| 1.973866 | 3.29931 | 0 | 7.110867 | 1.752707 | 0 | 50.76902 | 0.265426 | 0 |
| 0.28754 | 2.499419 | 0.784485 | 5.633327 | 3.628308 | 0.04121 | 22.46143 | 0.18607 | 0.070094 |
| 0.067591 | 0.461165 | 0 | 0 | 19.55676 | 2.565424 | 135.6576 | 0.563319 | 0.013263 |
| 0.276467 | 0.678024 | 0.102824 | 15.79084 | 0.75155 | 0.351098 | 16.95021 | 0.035228 | 0 |
| 0.024966 | 0.579975 | 0.085289 | 4.124153 | 9.558595 | 0 | 40.85625 | 0.488398 | 0.275974 |
| 0.19531 | 0.043417 | 0.052674 | 0.495264 | 8.195052 | 0.683467 | 21.67738 | 0.015468 | 0.017481 |
| 0.186049 | 1.036601 | 0 | 0.108983 | 1.828533 | 0.18008 | 14.17864 | 0.092329 | 0 |
| 0.80377 | 0.977119 | 0 | 5.511803 | 3.396025 | 0.124548 | 16.65233 | 0.709634 | 0.030263 |

| | | | | | | | | |
|---|---|---|---|---|---|---|---|---|
| 0.052892 | 2.908923 | 0.0173 | 0.790057 | 2.414513 | 0.023628 | 5.441635 | 0.00254 | 0 |
| 0.063229 | 5.305911 | 0.055019 | 6.059896 | 0.159579 | 0 | 2.051104 | 0.026928 | 0.006086 |
| 5.061103 | 0.604346 | 0.016664 | 0.08953 | 8.206776 | 3.106695 | 29.178 | 0.058722 | 0.01106 |
| 0.161525 | 0.377022 | 0.108907 | 0.204797 | 1.303944 | 0.104123 | 11.07631 | 0.422159 | 0.086743 |
| 7.039898 | 1.645048 | 0.09979 | 1.474411 | 2.34442 | 0.136296 | 12.46836 | 0.197805 | 0.016559 |
| 0.615951 | 0.206163 | 0 | 0.239971 | 3.270775 | 7.003202 | 32.46908 | 0.540385 | 0 |
| 0.103874 | 0.15669 | 0.03802 | 0.919223 | 1.362986 | 0.597175 | 16.90883 | 0.005582 | 0 |
| 0.068668 | 0.058006 | 0.070374 | 1.890511 | 0.367406 | 0.384472 | 16.0486 | 0 | 0 |
| 0.025713 | 0.939818 | 0.030405 | 0.449244 | 35.48903 | 0.083057 | 51.61907 | 0.256705 | 0.010091 |
| 0.224267 | 1.83617 | 0 | 72.52514 | 2.81217 | 0.024148 | 9.871154 | 0.132394 | 0 |
| 1.146646 | 0.440278 | 0 | 0.393452 | 7.130672 | 0.435382 | 23.36587 | 0.1771 | 0.011437 |
| 0.104376 | 0.576496 | 0.164568 | 2.597301 | 3.809009 | 0.084289 | 25.52292 | 0.356413 | 0.027308 |
| 34.58592 | 0.034693 | 0 | 0.282674 | 18.61948 | 12.93464 | 168.1028 | 0.111241 | 0 |
| 26.45788 | 0.371787 | 0 | 0.100976 | 7.343729 | 0 | 39.84902 | 1.607894 | 37.6395 |
| 49.67863 | 0.097986 | 0 | 0.452418 | 0.565471 | 13.04347 | 14.81876 | 0.008727 | 0.006575 |
| 0.973649 | 0.957807 | 1.12734 | 6.406389 | 0.567433 | 12.95757 | 20.82164 | 0.00764 | 0.011512 |
| 17.84392 | 0.182487 | 0 | 0.297377 | 1.992482 | 1.42554 | 16.13876 | 0.069659 | 0.020993 |
| 3.661333 | 0.066945 | 0.324875 | 2.290943 | 1.201406 | 0.277326 | 65.96171 | 1.347568 | 0.040431 |
| 1.226599 | 0.893804 | 0.015061 | 0.566429 | 3.003639 | 1.203369 | 35.89873 | 0.152586 | 0.004998 |
| 16.55181 | 0.22917 | 0.046339 | 3.361063 | 0.790287 | 0.189872 | 8.664515 | 0.585138 | 0 |
| 3.727086 | 0.636755 | 0.085836 | 13.37412 | 0.941074 | 0.029309 | 12.52481 | 0.050413 | 0.04273 |
| 0.226529 | 1.537686 | 0.195438 | 0.238647 | 3.444425 | 4.258812 | 13.94054 | 0.049566 | 0 |
| 0.121132 | 2.552716 | 0 | 0.473909 | 0.36158 | 0 | 3.493819 | 0.722393 | 0.019515 |
| 0.265781 | 1.169837 | 0.043008 | 7.221023 | 1.137649 | 0.058741 | 4.246339 | 0.094723 | 0.071366 |
| 0.084108 | 1.993949 | 0.041708 | 6.218508 | 6.300266 | 0 | 12.5363 | 0.587911 | 0.055367 |
| 0.032071 | 1.092993 | 0.017 | 12.19386 | 8.254297 | 0.01161 | 25.53427 | 0.461795 | 0.016926 |
| 0.021942 | 1.009676 | 0.071012 | 65.33725 | 18.02406 | 0.012124 | 106.3168 | 0.104267 | 0 |
| 0.052031 | 2.805473 | 0.136146 | 0.18287 | 4.797823 | 0.069732 | 23.24127 | 0.084959 | 0 |
| 0.513154 | 0.084664 | 0.102716 | 0 | 1.043912 | 9.595928 | 46.30975 | 0.063343 | 0.013635 |
| 0 | 4.891946 | 0.502965 | 2.634763 | 1.015338 | 0 | 6.81171 | 1.277616 | 0.033384 |
| 0.073161 | 1.334922 | 0.224937 | 5.13629 | 0.007829 | 0.076806 | 1.120271 | 0.284038 | 0.02986 |
| 0.086528 | 1.817714 | 0 | 23.15627 | 0.201026 | 0.023905 | 1.406914 | 0.010279 | 0.011617 |
| 0.007337 | 1.229225 | 4.260902 | 37.79011 | 0.043618 | 0 | 1.368735 | 0.073604 | 0 |
| 0.002613 | 1.490158 | 0.026783 | 0.179877 | 0.055932 | 0.11889 | 37.27941 | 0.50141 | 0.039999 |
| 25.95593 | 0.264655 | 0.245535 | 0.202954 | 0.466737 | 7.32626 | 10.44623 | 0.138661 | 0.025073 |
| 0.200055 | 0.020118 | 0 | 6.753597 | 1.214814 | 0.500052 | 11.40957 | 0.136185 | 0.032401 |
| 2.606218 | 0.26991 | 0 | 1.60542 | 1.806471 | 0.480795 | 11.65926 | 0.096162 | 0 |
| 0.071539 | 0.805758 | 0.073317 | 46.18658 | 0.289206 | 0.066759 | 17.44582 | 0.007177 | 0.040553 |
| 0.833596 | 0.716103 | 0.043439 | 0.641823 | 1.012986 | 0.029665 | 6.072818 | 0.025513 | 0 |
| 0.008157 | 0 | 0 | 0 | 2.044034 | 0 | 13.8418 | 5.983947 | 0.998778 |
| 0.411368 | 2.259945 | 0.044223 | 2.138385 | 1.554571 | 0.0604 | 11.1668 | 0.694775 | 0 |
| 1.628528 | 2.1544 | 0.08168 | 10.75176 | 1.620445 | 0.11156 | 8.457062 | 0.083952 | 0 |
| 0.115118 | 3.036388 | 0.036116 | 6.209324 | 0.930191 | 0.123319 | 2.52448 | 0.222721 | 0.1678 |
| 0.813572 | 0.543899 | 0 | 4.864512 | 7.098362 | 1.59293 | 17.8599 | 0.274893 | 0.005093 |
| 0.16659 | 0.089824 | 0 | 7.245629 | 0.967202 | 0.111632 | 5.903508 | 0.336022 | 0.054249 |
| 0.362584 | 0.984496 | 0 | 6.417281 | 2.217153 | 0.453152 | 9.624412 | 0.097431 | 0 |
| 0.556766 | 0.332773 | 0.242235 | 4.392486 | 5.733126 | 0.248138 | 16.4031 | 0.005928 | 0 |
| 2.381884 | 0.539993 | 0 | 0.248686 | 3.370717 | 0.175068 | 14.02423 | 0.081555 | 0.004726 |
| 1.001138 | 0.809709 | 0.036383 | 6.597455 | 11.47299 | 0 | 56.33184 | 0.38998 | 0.012075 |
| 0.394996 | 1.077192 | 0.015937 | 5.993972 | 2.113428 | 0 | 21.30569 | 0.650547 | 2.850861 |
| 0.069514 | 1.233146 | 0.320587 | 42.34341 | 0.520712 | 0 | 16.10647 | 0.015691 | 0 |
| 8.195225 | 0.268448 | 0.038316 | 0.283061 | 8.981762 | 0.052333 | 25.51059 | 0.306614 | 0.006358 |
| 0.150151 | 0.691843 | 0 | 46.08318 | 0.43821 | 0 | 11.82584 | 0.092432 | 0 |
| 0.001265 | 0.897722 | 0 | 3.030325 | 18.58145 | 0.013282 | 46.11993 | 2.77857 | 0.05809 |
| 0.103442 | 0.986416 | 0 | 8.873998 | 0.182587 | 0.011195 | 2.220207 | 0.004814 | 0.005441 |
| 4.817514 | 0.199378 | 0.040315 | 0.054151 | 11.25338 | 3.992054 | 155.2716 | 0.156865 | 0.00669 |
| 0.20538 | 0.156143 | 0 | 0.508896 | 1.999978 | 0.34498 | 52.30386 | 2.317902 | 0.041912 |
| 0.011068 | 0.621057 | 0.413196 | 3.656494 | 4.500537 | 0.132789 | 29.4558 | 0.688781 | 0 |

| | | | | | | | | |
|---|---|---|---|---|---|---|---|---|
| 0.04043 | 1.075802 | 0 | 0.834815 | 9.489228 | 7.540879 | 30.8451 | 0.54146 | 0.013751 |
| 8.475215 | 0.798208 | 4.320547 | 0.050029 | 227.6603 | 0.012718 | 161.6285 | 0.634388 | 0.018541 |
| 0.117907 | 0.892969 | 0.10417 | 0.727584 | 2.936777 | 0 | 11.43187 | 0.082594 | 0.041485 |
| 0.085961 | 5.436073 | 0.03637 | 59.01356 | 0.20676 | 0 | 9.395822 | 0.030261 | 0 |
| 0.327402 | 3.325395 | 0 | 8.369967 | 3.475722 | 0.190951 | 23.94257 | 0.287391 | 0.026513 |
| 0.341397 | 0.068664 | 0 | 0.335683 | 1.71067 | 0.768012 | 43.89078 | 0.14678 | 0.027646 |
| 0.014986 | 0.68359 | 0 | 0.247548 | 0.008018 | 0.346113 | 1.529788 | 0.226633 | 0.007645 |
| 0.056381 | 0.22861 | 0.208015 | 1.909268 | 1.496272 | 0.094704 | 16.50924 | 0.402149 | 0.069034 |
| 0.031366 | 3.63813 | 0.222546 | 34.27653 | 0.813308 | 0 | 9.317381 | 0.054461 | 0.012309 |
| 0.062052 | 1.375959 | 0 | 11.21122 | 0.733188 | 0.488576 | 212.2582 | 0.035016 | 0.013191 |
| 0 | 0 | 0 | 0 | 18.22019 | 0 | 14.80897 | 0.022712 | 0 |
| 0.002026 | 0.012837 | 0 | 0 | 11.30193 | 0.191443 | 35.63885 | 7.795516 | 0.206743 |
| 0.353168 | 0.403759 | 0.040821 | 1.80939 | 2.330065 | 2.146518 | 13.12543 | 0.11388 | 0.081283 |
| 0.06139 | 0.820305 | 0.034317 | 0.921902 | 0.4718 | 6.714363 | 8.63564 | 1.496539 | 0.005694 |
| 0.404333 | 2.326926 | 0 | 14.73529 | 1.301481 | 0 | 5.733876 | 0.534502 | 0.008218 |
| 0.637086 | 0.611068 | 0 | 2.096397 | 11.32623 | 0.053293 | 17.93274 | 0.40677 | 0.012949 |
| 0.004851 | 2.627869 | 0.018644 | 6.461072 | 2.829289 | 0.012732 | 10.11635 | 0.643324 | 0 |
| 2.230615 | 0.042439 | 0 | 0.899048 | 4.605517 | 0.562581 | 23.30326 | 0 | 0 |
| 0.340302 | 5.634318 | 0 | 7.682567 | 0.679761 | 0 | 6.886817 | 0.010242 | 0 |
| 0.03148 | 0.368198 | 0 | 1.100018 | 11.32386 | 0 | 28.16713 | 4.509292 | 0.086478 |
| 0.108528 | 5.312284 | 0.045709 | 3.990734 | 0.556818 | 0.546265 | 6.150461 | 0.228189 | 0 |
| 0.0258 | 19.83294 | 0.176276 | 3.847557 | 2.822249 | 0.391238 | 4.120055 | 0.245885 | 0.160877 |
| 1.379449 | 1.380405 | 0.011091 | 0.104281 | 0.193011 | 0.007574 | 1.259789 | 0.008142 | 0.011042 |
| 37.86719 | 0.564731 | 0 | 0.083662 | 0.686491 | 0.212678 | 7.274396 | 0.009145 | 0.00689 |
| 1.069122 | 5.107835 | 0.673577 | 8.142707 | 2.063074 | 0.061333 | 14.71343 | 0.441761 | 0 |
| 6.686453 | 0.245104 | 0.019824 | 0.079884 | 9.922035 | 0.094768 | 44.94909 | 0.090235 | 0.006579 |
| 0.142249 | 0.158456 | 0.02403 | 4.099209 | 0.727648 | 0.443084 | 23.09599 | 0.014113 | 0 |
| 0.07474 | 2.367565 | 0.778319 | 10.5788 | 0.644991 | 1.328809 | 4.695621 | 0.574126 | 0.0123 |
| 0.537111 | 0.185482 | 0 | 6.556678 | 1.036261 | 0.945694 | 9.180598 | 0.012708 | 0 |
| 0.387492 | 0.706108 | 0.079689 | 17.74163 | 0.644865 | 0.013605 | 9.103939 | 0.052654 | 0.019835 |
| 0.066439 | 1.282827 | 0 | 9.733783 | 2.691525 | 0.298926 | 15.89332 | 0.021424 | 0 |
| 0.125406 | 0.469138 | 0 | 4.217083 | 3.137643 | 0.112846 | 12.12789 | 0.010783 | 0.01828 |
| 13.48415 | 0.060583 | 0.055125 | 0.246811 | 6.843129 | 0.476842 | 26.82804 | 0.884945 | 0 |
| 0.018042 | 1.25734 | 1.155625 | 0.74507 | 1.52843 | 0 | 4.402406 | 0.013574 | 0 |
| 1.072641 | 0.125847 | 0.057255 | 1.230469 | 3.507272 | 0.521333 | 18.35965 | 0.114892 | 0.012667 |
| 0.123977 | 0.751304 | 11.35225 | 6.455477 | 2.278418 | 0.056588 | 22.51689 | 0.066918 | 0 |
| 0.171899 | 0.460169 | 0.018609 | 1.149823 | 11.13409 | 0.228755 | 20.52013 | 0.510966 | 0.012352 |
| 3.595871 | 0.245492 | 0 | 0.320041 | 1.437451 | 0.122037 | 28.83383 | 0.137025 | 0.019769 |
| 0.01858 | 0.033633 | 0 | 2.740384 | 3.621491 | 0.027866 | 44.74378 | 0.347493 | 0.108333 |
| 0.360758 | 1.186746 | 0 | 2.650166 | 5.475191 | 3.350309 | 17.09956 | 0.618551 | 0 |
| 0.190549 | 2.414452 | 0.036388 | 1.832868 | 0.487603 | 0.1491 | 4.641949 | 0.010686 | 0.006038 |
| 13.8175 | 0.099971 | 0 | 7.168068 | 0.865385 | 0.082828 | 19.07522 | 0.020777 | 0.006709 |
| 0.030366 | 1.552349 | 0.386325 | 11.26467 | 0.078436 | 0 | 4.219389 | 0.016545 | 0 |
| 0.011598 | 8.670475 | 0.035658 | 0.766334 | 3.872207 | 0 | 18.47568 | 1.062848 | 0.05917 |
| 0.088298 | 2.967821 | 0.014288 | 0.786861 | 0.686277 | 0.146363 | 2.222194 | 0.025175 | 0 |
| 0.044245 | 2.225528 | 0.247332 | 22.28614 | 0.803458 | 0.225208 | 13.3986 | 0.251185 | 0.054722 |
| 0.23039 | 1.685782 | 0 | 3.01514 | 1.927187 | 0.068132 | 15.90872 | 0.344247 | 0 |
| 0.072669 | 8.354766 | 0.197137 | 7.215627 | 8.61679 | 0.022438 | 15.51671 | 0.144729 | 0.010904 |
| 1.049434 | 0.317324 | 0.128328 | 10.21902 | 11.54904 | 0.062598 | 50.55287 | 1.448174 | 0.048672 |
| 0.032699 | 3.505896 | 0.058001 | 1.038756 | 0.100937 | 0 | 1.452779 | 0.051098 | 0 |
| 1.906518 | 5.557983 | 0.265474 | 4.778213 | 0.960948 | 1.305326 | 1.670094 | 0.701634 | 0.510998 |
| 3.974118 | 0.232592 | 0 | 0.568543 | 2.160729 | 0.610239 | 9.903419 | 0.034528 | 0 |
| 0.039699 | 1.732648 | 0.135618 | 12.11372 | 3.319884 | 0 | 6.458027 | 0.136071 | 0.007501 |
| 0.024871 | 0.585259 | 0 | 12.03166 | 0.944175 | 0.136766 | 7.126661 | 0.013366 | 0 |
| 0.007517 | 2.62597 | 0 | 7.228152 | 0.235559 | 0.022546 | 10.16825 | 0.029085 | 0.125999 |
| 89.93569 | 0.018878 | 0 | 0.092292 | 88.59706 | 35.84952 | 455.9535 | 0.010089 | 0 |
| 0.824335 | 1.512553 | 0.069247 | 2.13929 | 1.289441 | 0.070935 | 7.442083 | 0.030503 | 0.011491 |
| 0.399101 | 0.478367 | 0 | 22.38394 | 2.380676 | 0.05662 | 8.837875 | 0.066955 | 0.013758 |

| | | | | | | | | |
|---|---|---|---|---|---|---|---|---|
| 0.38487 | 0.039328 | 19.18086 | 7.498375 | 6.958269 | 0.065168 | 80.62789 | 5.842847 | 0.015835 |
| 2.394338 | 0.285765 | 0 | 8.847891 | 2.855809 | 2.722762 | 79.06318 | 0.025453 | 0.095882 |
| 0.451686 | 4.546454 | 0.030059 | 0.767128 | 2.568452 | 0.061583 | 4.990168 | 1.507238 | 0.004988 |
| 0.114106 | 15.59309 | 0.044408 | 3.877154 | 0.850097 | 0 | 4.074146 | 1.62359 | 0.044213 |
| 0.750043 | 0.236609 | 0.047843 | 0.546232 | 0.482905 | 0.359399 | 18.14242 | 0.049174 | 0.031755 |
| 13.05455 | 0.017556 | 0 | 0.200265 | 1.275093 | 0.494552 | 64.44773 | 0.794362 | 0.028275 |
| 42.30266 | 0.206009 | 0 | 2.741625 | 1.928277 | 2.147754 | 23.60312 | 0.079512 | 0 |
| 2.488019 | 1.130168 | 0 | 36.54333 | 2.725225 | 0.073924 | 16.58327 | 0.180133 | 0 |
| 0.016446 | 1.95886 | 0.101129 | 10.25557 | 1.865497 | 2.676148 | 4.484317 | 0.077956 | 0 |
| 0.191999 | 2.132138 | 0.132654 | 4.855393 | 0.934951 | 0.090591 | 5.940176 | 0.058433 | 0.011006 |
| 0.075121 | 1.971715 | 0.948598 | 1.163356 | 0.488066 | 0.028166 | 3.639673 | 0.006056 | 0 |
| 0.039678 | 0.476297 | 0.096308 | 3.79459 | 0.659235 | 0.032885 | 5.413611 | 0.007071 | 0.005327 |
| 0.039162 | 4.727179 | 89.06814 | 0.255359 | 0.192994 | 0.021638 | 11.14331 | 0.016283 | 0 |
| 0.116864 | 1.633218 | 0 | 0.141945 | 1.039069 | 0.018042 | 10.11071 | 0.418949 | 0.394551 |
| 0.095394 | 1.510921 | 0.101837 | 1.20373 | 2.814317 | 0.111274 | 15.10886 | 0.714746 | 0.020278 |
| 0.110405 | 0.69947 | 0.115719 | 2.590559 | 1.181441 | 0.026342 | 26.22109 | 6.76813 | 1.395342 |
| 1.582804 | 1.701902 | 0.040486 | 1.631409 | 3.621435 | 0 | 10.96607 | 0.053501 | 0.013436 |
| 0.022546 | 1.295076 | 0 | 1.241434 | 1.552126 | 0 | 4.481921 | 0.284984 | 0.237715 |
| 0.042015 | 1.962158 | 0.055361 | 1.313703 | 0.160571 | 0 | 0.935184 | 0.046063 | 0.006124 |
| 0.404495 | 0.099201 | 0 | 7.490051 | 1.884993 | 0.273965 | 44.62168 | 0.011781 | 0.013314 |
| 30.67769 | 0.045053 | 0 | 1.615192 | 1.598037 | 0.671892 | 20.15401 | 0 | 0.01814 |
| 0.468614 | 0.692138 | 0.132586 | 0.474905 | 10.82916 | 0.331997 | 16.87506 | 1.635287 | 0.029334 |
| 1.490073 | 0.847707 | 0.342817 | 14.9025 | 1.724694 | 0.212831 | 20.12339 | 0.306592 | 0.010343 |
| 0.034328 | 2.273684 | 0.095947 | 20.07232 | 0.350643 | 0 | 6.036005 | 0.014088 | 0 |
| 0.957684 | 0.480838 | 0.230274 | 1.546514 | 1.864764 | 0.062903 | 4.949558 | 0.202867 | 0 |
| 33.86143 | 0 | 0 | 0.167551 | 34.29879 | 0.979647 | 83.10617 | 1.959778 | 0 |
| 0.415348 | 2.878138 | 0.538735 | 4.663371 | 10.99354 | 0.10901 | 32.11352 | 3.269581 | 0.019866 |
| 0.134624 | 1.582727 | 0.042671 | 3.324289 | 7.596653 | 0.02914 | 20.71106 | 0.316402 | 0.021242 |
| 0.711778 | 4.6984 | 0.059792 | 11.59175 | 0.4509 | 0.14972 | 5.485539 | 0.058529 | 0.006614 |
| 0.089102 | 0.307913 | 0 | 1.059292 | 8.090141 | 0.113383 | 7.797371 | 0.670397 | 0.13775 |
| 0.271277 | 0.187492 | 0.045494 | 0.244428 | 1.546473 | 2.796136 | 9.83151 | 0.004453 | 0.020131 |
| 6.595133 | 5.471655 | 0.603482 | 1.621187 | 1.050217 | 0 | 13.70958 | 1.240533 | 0 |
| 25.05851 | 0.018702 | 0 | 0.030476 | 6.744061 | 0.015495 | 197.9292 | 0.24986 | 0.03012 |
| 0.03505 | 0.262435 | 0.024492 | 0.032897 | 6.504072 | 0.75265 | 50.90928 | 3.355164 | 0.146305 |
| 0.081978 | 0.439469 | 0.14541 | 0.455733 | 0.168701 | 0 | 7.369017 | 0.64052 | 0 |
| 0.074579 | 1.922579 | 1.265086 | 0.212407 | 1.802547 | 0 | 14.0589 | 0.034829 | 0.02624 |
| 0.036072 | 0.564615 | 0 | 5.301466 | 0.244093 | 0.089104 | 2.679082 | 0.079026 | 0.010825 |
| 0.159355 | 0.827139 | 0 | 10.30743 | 0.61636 | 0 | 12.17213 | 0.528706 | 0.029385 |
| 0.259566 | 2.654251 | 0.023335 | 7.240226 | 0.755317 | 0.254968 | 7.482621 | 0.243263 | 0.023232 |
| 0.535311 | 4.107974 | 0.019317 | 4.903961 | 0.64545 | 0.13192 | 11.39296 | 0.022691 | 0.012822 |
| 2.574606 | 0.423674 | 0.128502 | 0.172603 | 9.123995 | 2.3694 | 81.21477 | 0.169812 | 0 |
| 2.541135 | 0.098769 | 0 | 2.414295 | 1.960211 | 0.027277 | 27.1871 | 0.029324 | 0.013256 |
| 0.13773 | 1.745176 | 0.111436 | 12.94729 | 3.917329 | 0.228302 | 13.53397 | 0.654485 | 0.018491 |
| 0.794039 | 14.59072 | 1.081019 | 4.35606 | 1.896936 | 0.12304 | 7.910519 | 0.059522 | 0.029897 |
| 0.068458 | 0.136964 | 1.938613 | 0 | 20.69523 | 0.643038 | 204.449 | 1.886807 | 0.027573 |
| 0.031306 | 1.081842 | 0 | 3.408379 | 9.913037 | 0.149388 | 68.80815 | 0.077086 | 0 |
| 0.501478 | 1.289958 | 0.038642 | 1.920434 | 4.303814 | 0 | 7.562985 | 0.034043 | 0.012824 |
| 0.630153 | 1.728966 | 0.019069 | 20.61897 | 1.320779 | 0 | 13.54594 | 0.117597 | 0.018986 |
| 0.003454 | 1.444153 | 0 | 5.419885 | 0.720688 | 0.036258 | 8.257414 | 1.161556 | 0 |
| 0.358977 | 1.80305 | 0.049716 | 19.25427 | 2.410978 | 0 | 21.7195 | 3.428461 | 0.060497 |
| 0.407717 | 2.635101 | 0.126195 | 9.492272 | 6.193087 | 0.114907 | 32.16004 | 0.179117 | 0 |
| 2.389351 | 1.145084 | 0.29006 | 0.32809 | 0.132837 | 0.604682 | 2.414346 | 0.403481 | 0.005066 |
| 0.069688 | 0.963291 | 0.925205 | 18.83718 | 5.609929 | 0.199526 | 25.82615 | 0.164448 | 0.032321 |
| 0.087322 | 3.712771 | 0.894912 | 29.77058 | 17.31811 | 0.020372 | 14.80452 | 0.591302 | 0.0099 |
| 0.330514 | 0.418795 | 0 | 275.9421 | 2.475782 | 0.05783 | 17.23974 | 0.323279 | 0.028103 |
| 5.843176 | 0.081811 | 0.049627 | 0.133318 | 79.02351 | 5.456459 | 132.5135 | 0.626664 | 0 |
| 2.892175 | 0.272902 | 0 | 0 | 6.4697 | 6.104825 | 79.01711 | 0.097228 | 0.015697 |
| 0.118647 | 4.844225 | 0.050665 | 1.179575 | 1.622323 | 0 | 12.85251 | 0.190937 | 0.005605 |

| | | | | | | | | |
|---|---|---|---|---|---|---|---|---|
| 0.411139 | 1.665751 | 0.317006 | 1.250792 | 2.392889 | 0.432975 | 11.07936 | 1.675664 | 0.046027 |
| 0.034803 | 1.763943 | 0.071335 | 0.550945 | 0.335182 | 0 | 3.069688 | 0.002619 | 0 |
| 0.017364 | 2.048977 | 0.333671 | 3.652714 | 1.335555 | 0 | 7.551017 | 0.017148 | 0.01661 |
| 0.511692 | 0.01348 | 0 | 3.712265 | 0.813944 | 0 | 12.76002 | 0.084042 | 0.054273 |
| 0.122356 | 1.107407 | 0 | 12.75261 | 3.366842 | 0.734007 | 23.32152 | 0.170968 | 0 |
| 0.116423 | 2.120599 | 0.149145 | 37.46177 | 11.06985 | 0 | 41.50532 | 0.613172 | 0 |
| 0.068904 | 1.714984 | 0.07566 | 6.148368 | 6.63607 | 0.077504 | 11.33747 | 0.016664 | 0 |
| 15.62495 | 0.185581 | 0 | 0.100807 | 29.07307 | 0.563778 | 66.49291 | 0.071628 | 0 |
| 0.015978 | 1.966768 | 0.052635 | 0 | 1.288486 | 0 | 11.74269 | 0.04637 | 0.034936 |
| 0.0159 | 0.856234 | 0.061106 | 0.492461 | 0.430677 | 0 | 5.352449 | 1.758547 | 0.040558 |
| 0.037223 | 0.182578 | 0.055377 | 0.942165 | 21.43262 | 0.189086 | 163.5741 | 0.051496 | 0.018378 |
| 116.345 | 0.026768 | 0.097427 | 0.043621 | 1.00599 | 0.57663 | 24.85705 | 0.524527 | 0.010778 |
| 2.038371 | 1.771231 | 0.352802 | 24.68494 | 7.434601 | 0.109515 | 15.2729 | 0.042384 | 0 |
| 0.111149 | 0.031649 | 0.019198 | 0.28366 | 8.399375 | 0.44577 | 63.39138 | 1.186766 | 0.165657 |
| 1.747904 | 0.151697 | 0 | 28.42834 | 30.92302 | 1.508206 | 84.42295 | 1.351142 | 0.015269 |
| 0.621809 | 0 | 0.095589 | 0 | 6.820331 | 0.424311 | 68.06923 | 1.185986 | 0.015862 |
| 0.106976 | 2.697962 | 0.06325 | 1.762872 | 10.23674 | 0.010799 | 32.41292 | 0.153237 | 0.005248 |
| 3.612685 | 1.077488 | 0.102127 | 0.631012 | 0.909967 | 0.195283 | 6.406957 | 0.143956 | 0.033893 |
| 0.007296 | 2.496059 | 1.323461 | 49.14194 | 1.038379 | 0.015319 | 11.91652 | 0.569799 | 0.007444 |
| 1.121699 | 2.124634 | 0 | 2.566858 | 3.155509 | 0.0607 | 15.8043 | 0 | 0 |
| 19.27823 | 0.192179 | 0 | 0.913416 | 1.541848 | 2.322012 | 6.179448 | 0.199699 | 0.019344 |
| 0.010903 | 1.88801 | 0.167602 | 1.050572 | 4.783421 | 0 | 21.33175 | 1.361705 | 0.074163 |
| 26.76366 | 0.396357 | 0.048087 | 0.387539 | 2.343136 | 0 | 7.562819 | 0.056485 | 0 |
| 0.05827 | 0.878372 | 0.015444 | 0.290425 | 0.747183 | 0 | 4.669066 | 0.024945 | 0 |
| 52.7162 | 0.068649 | 0.020821 | 0.335606 | 2.935013 | 0.284384 | 34.74801 | 1.048625 | 0.00691 |
| 0.019734 | 2.934731 | 0.239493 | 1.608429 | 0.277854 | 0.010904 | 7.686698 | 0.004689 | 0 |
| 0.232187 | 0.196137 | 0 | 0.191773 | 2.004274 | 0.162503 | 43.62032 | 0.034939 | 0 |
| 0.096802 | 0.479967 | 0.452903 | 15.20842 | 2.567183 | 0 | 39.9398 | 0.04275 | 0.966249 |
| 0.0059 | 4.336128 | 1.342375 | 0.170561 | 0.511414 | 0.012388 | 2.837148 | 0.005327 | 0 |
| 0.109373 | 9.616251 | 0.034314 | 8.43442 | 2.79464 | 0.749859 | 2.968157 | 0.136033 | 0.250529 |
| 0.222464 | 0.014994 | 0 | 0.024434 | 28.54175 | 2.223666 | 106.9997 | 0.117522 | 0.078481 |
| 0.037016 | 0.774927 | 0.074223 | 1.927447 | 0.843891 | 0 | 5.685357 | 0.029062 | 0.008211 |
| 22.77729 | 0.027848 | 0.033785 | 1.679056 | 4.056846 | 29.45168 | 70.77334 | 0.104174 | 0.033637 |
| 0.070539 | 1.842612 | 0.033365 | 2.868245 | 0.092904 | 0.102536 | 5.859761 | 0 | 0.005537 |
| 0.676162 | 2.73436 | 1.045474 | 1.836361 | 3.932704 | 0.04119 | 10.01317 | 0.991895 | 0.080068 |
| 0.663557 | 0.660954 | 0.018648 | 2.154162 | 1.914732 | 0.738641 | 7.593001 | 7.973492 | 0.030944 |
| 0.363874 | 0.367516 | 0.162137 | 1.905588 | 1.312049 | 1.273341 | 17.95629 | 0.809426 | 0.053809 |
| 0.428301 | 2.203786 | 0.018188 | 36.69435 | 1.677572 | 0 | 6.849425 | 0.208306 | 0.018108 |
| 1.341937 | 4.012269 | 0.033571 | 7.665635 | 1.542336 | 0.126092 | 10.70631 | 0.483062 | 0.022282 |
| 0.412547 | 0.965057 | 0.048784 | 1.638169 | 3.718509 | 0.133261 | 21.22734 | 0.336662 | 0 |
| 1.789494 | 1.049756 | 0.063679 | 1.454067 | 3.967295 | 3.174564 | 24.98213 | 0.280501 | 0 |
| 10.70452 | 0.242209 | 0 | 0.986752 | 3.681938 | 2.909791 | 37.01523 | 0.086293 | 0.032507 |
| 0.701109 | 0 | 0 | 0.247468 | 4.801329 | 0.346001 | 45.79822 | 0.131878 | 0.007643 |
| 0.031708 | 3.311194 | 0.285742 | 9.34683 | 2.281574 | 0.826461 | 21.85317 | 2.517337 | 17.03027 |
| 0.045176 | 1.026047 | 1.441367 | 2.305645 | 0.602006 | 0 | 21.06621 | 0.088503 | 0.008697 |
| 0.020063 | 0.451955 | 0.06854 | 10.63322 | 5.140862 | 0.023403 | 43.68734 | 0.583698 | 0 |
| 0.05052 | 5.412087 | 0.017651 | 8.037089 | 3.02867 | 0.012054 | 9.422971 | 0.176232 | 0.011715 |
| 0.774375 | 1.629695 | 3.501241 | 4.204902 | 0.910378 | 0.08439 | 14.6482 | 0.136082 | 0 |
| 0.03977 | 0.770714 | 0.071926 | 19.51545 | 1.426947 | 3.094521 | 28.75186 | 0.168976 | 0.011935 |
| 0.473755 | 2.702904 | 0.209717 | 1.997441 | 0.212342 | 0 | 2.448749 | 0.016796 | 0.006327 |
| 0.578151 | 0.104654 | 0 | 0 | 3.933039 | 1.213909 | 65.5643 | 3.131979 | 0.042137 |
| 0.15756 | 4.238081 | 0.276207 | 17.09456 | 2.181519 | 0.01451 | 12.19675 | 1.207311 | 0.028205 |
| 0.112423 | 1.327385 | 0.314225 | 0.527582 | 1.380758 | 0.026824 | 14.48272 | 0.051905 | 0 |
| 0.125969 | 0.499666 | 0 | 2.646307 | 16.81477 | 0.034499 | 54.73245 | 1.038439 | 0.016765 |
| 0.023929 | 0.545771 | 0.055178 | 21.56746 | 2.291784 | 0 | 30.5663 | 0.048611 | 0.006104 |
| 1.235944 | 7.312719 | 0.641343 | 0.825555 | 1.562547 | 0.109495 | 7.191447 | 0.188337 | 0.008868 |
| 0.079594 | 0.444946 | 0 | 0.338369 | 2.279661 | 0.098306 | 33.20508 | 1.114943 | 0.310525 |
| 0.137274 | 0.618453 | 0 | 8.251547 | 1.468969 | 0 | 15.44731 | 0.316737 | 0.015563 |

| | | | | | | | | |
|---|---|---|---|---|---|---|---|---|
| 0.852518 | 2.904654 | 0.023033 | 1.918103 | 8.152804 | 0.298854 | 15.99907 | 0.372006 | 0.542712 |
| 0.344725 | 0.99194 | 0.211129 | 0.368665 | 0.220453 | 0.043255 | 2.176795 | 1.664713 | 0.063061 |
| 1.9578 | 2.747769 | 0 | 2.901062 | 2.729113 | 0.032065 | 21.74316 | 0.67562 | 0.015582 |
| 0.004853 | 0.215233 | 0.074607 | 35.52508 | 2.778488 | 0 | 15.48211 | 0.005477 | 0 |

65 overlapping genes among the APC status DEGs related to immunophenotyp

| GNLY | PLA2G2A | SEMG2 | CCL4 | PDCD1 | AQP9 | FASLG | CCL19 | CCL18 |
|---|---|---|---|---|---|---|---|---|
| 0.39206 | 578.4543 | 0 | 1.53054 | 0.266751 | 0.058105 | 1.131371 | 2.067302 | 2.593203 |
| 0.806817 | 421.0029 | 0 | 1.06394 | 2.733872 | 0.046317 | 0.702925 | 56.86016 | 16.71659 |
| 4.207412 | 155.0449 | 0 | 0.687652 | 0.355912 | 0.170875 | 0.471712 | 1.798338 | 4.869208 |
| 1.079252 | 106.9894 | 0 | 1.78466 | 0.542078 | 0.1121 | 0.806727 | 13.06821 | 1.505604 |
| 2.786126 | 665.5301 | 0 | 2.674778 | 0.548323 | 0.095551 | 0.804969 | 9.348827 | 39.07182 |
| 1.835327 | 43.14271 | 0 | 1.495719 | 0.562579 | 1.429683 | 0.489939 | 15.24242 | 3.535336 |
| 1.960743 | 75.511 | 0 | 1.368706 | 0.36407 | 0.059478 | 0.60221 | 2.003576 | 2.566813 |
| 0.496598 | 18.28916 | 0 | 1.078629 | 0.403583 | 0.027283 | 0.794708 | 10.96143 | 1.621563 |
| 0.95171 | 943.5104 | 0 | 3.702333 | 1.034616 | 1.031633 | 0.519311 | 48.62986 | 81.76344 |
| 1.14833 | 179.77 | 0.01088 | 1.888922 | 0.781153 | 0.062084 | 1.311989 | 28.34689 | 11.83671 |
| 2.789613 | 914.2636 | 0 | 3.933986 | 1.212269 | 0.864869 | 1.207945 | 26.44649 | 33.96271 |
| 1.278465 | 1244.279 | 0 | 0.950098 | 0.167739 | 0.17051 | 0.469544 | 4.79189 | 3.469003 |
| 4.541463 | 6.11078 | 0 | 1.299831 | 1.563435 | 0.082956 | 0.66582 | 107.9051 | 6.126963 |
| 1.289166 | 16.07271 | 0 | 1.212697 | 0.70612 | 0.241703 | 0.301198 | 16.1621 | 5.088241 |
| 1.083662 | 28.26697 | 0 | 2.252899 | 0.688419 | 0.008735 | 0.763341 | 27.96763 | 7.352895 |
| 2.648695 | 23.40727 | 0 | 1.888262 | 1.30803 | 0.039757 | 0.613091 | 20.27679 | 6.306352 |
| 2.784969 | 175.4205 | 0 | 0.958041 | 0.769205 | 0.028319 | 0.230484 | 9.167017 | 11.57436 |
| 0.443697 | 32.29081 | 0 | 0.841516 | 0.629125 | 2.892515 | 0.490587 | 27.11167 | 2.431047 |
| 0.772492 | 44.47021 | 0 | 1.939797 | 0.807333 | 0.414522 | 0.236755 | 11.66251 | 1.948887 |
| 3.184589 | 202.0803 | 0 | 0.986378 | 1.020355 | 0.041305 | 0.303317 | 17.98324 | 3.320379 |
| 1.596889 | 54.92711 | 0 | 1.383962 | 1.194709 | 0.509403 | 0.483856 | 21.92339 | 3.308045 |
| 0.744429 | 139.7877 | 0 | 1.249241 | 1.000654 | 0.075715 | 0.448174 | 17.73923 | 21.69336 |
| 1.980428 | 276.7582 | 0 | 1.95538 | 0.411495 | 0.450594 | 0.510564 | 6.717384 | 72.78769 |
| 2.137266 | 131.2608 | 0 | 4.152894 | 1.340683 | 0.171269 | 3.803709 | 17.03139 | 11.95912 |
| 1.503987 | 82.31491 | 0 | 0.605291 | 0.145453 | 0.065804 | 0.225504 | 5.692527 | 3.166137 |
| 0.635236 | 236.9846 | 0 | 2.42706 | 0.450212 | 1.235655 | 0.100821 | 9.086541 | 4.57077 |
| 4.48339 | 47.9577 | 0 | 2.227311 | 0.382734 | 1.008247 | 1.017801 | 3.35172 | 2.570061 |
| 2.116285 | 34.37607 | 0.024919 | 2.649357 | 0.370706 | 0.12639 | 1.813724 | 5.985753 | 0.621944 |
| 0.865553 | 72.60058 | 0 | 3.032416 | 0.999357 | 0.046647 | 0.146534 | 8.804289 | 2.340418 |
| 3.694542 | 23.50959 | 0 | 1.684843 | 0.275008 | 1.23302 | 0.573087 | 3.782664 | 1.687733 |
| 2.100609 | 287.8538 | 0 | 1.255765 | 0.856167 | 0.683307 | 0.550047 | 34.86451 | 2.484982 |
| 4.618917 | 16.61269 | 0 | 0.965061 | 0.572946 | 0.124803 | 0.5397 | 11.97575 | 4.761657 |
| 1.518091 | 443.5198 | 0 | 3.622179 | 2.559738 | 0.056703 | 1.955296 | 49.16347 | 5.334277 |
| 0.63332 | 130.0914 | 0 | 9.012556 | 0.934341 | 0.09955 | 0.284289 | 16.35774 | 4.349233 |
| 4.043101 | 656.9761 | 0.009044 | 2.309575 | 0.754621 | 0.051606 | 2.456254 | 27.74088 | 2.483021 |
| 1.663541 | 179.8633 | 0 | 1.876776 | 0.835666 | 0.052008 | 1.128779 | 13.17253 | 5.158625 |
| 0.706362 | 14.45876 | 0 | 0.292924 | 0.09709 | 0.031723 | 0.076099 | 1.00379 | 1.46921 |
| 0.546006 | 23.78923 | 0 | 0.794196 | 0.89883 | 0.627596 | 0.427385 | 31.69055 | 2.850721 |
| 1.726239 | 19.81986 | 0 | 1.648492 | 0.630001 | 0.081091 | 0.780231 | 23.86696 | 3.148927 |
| 0.328365 | 186.8121 | 0 | 2.399422 | 0.505502 | 0.402595 | 0.742891 | 13.3235 | 10.15945 |
| 4.247985 | 2961.766 | 0.011702 | 3.425356 | 0.408723 | 0.957077 | 1.360233 | 48.03312 | 9.603882 |
| 1.129575 | 30.48325 | 0 | 1.541845 | 0.419911 | 0.150652 | 0.313451 | 10.97658 | 3.413864 |
| 0.386909 | 19.48179 | 0 | 2.113614 | 0.487116 | 0.737569 | 0.239455 | 5.010302 | 3.595728 |
| 0.523928 | 0.45536 | 0 | 1.143287 | 0.483937 | 0.026353 | 0.135466 | 0.137152 | 0.732314 |
| 14.5017 | 218.49 | 5.419524 | 11.73101 | 4.046207 | 0.483187 | 3.33467 | 0.502931 | 14.67008 |
| 1.001741 | 12.63672 | 0 | 6.606364 | 1.629462 | 2.609846 | 0.885426 | 1.456039 | 3.31187 |
| 1.945881 | 0.901328 | 0 | 6.862584 | 0.152392 | 6.103118 | 0.901921 | 0.473847 | 40.12542 |
| 0.481453 | 6.370548 | 0.013773 | 0.903614 | 0.307338 | 0.506462 | 0.134659 | 0.109067 | 1.718761 |
| 2.925378 | 22.7126 | 0 | 2.761879 | 2.782688 | 0.459831 | 0.895341 | 7.657939 | 2.323245 |
| 1.05344 | 120.6581 | 0.066968 | 0.405568 | 0.402831 | 0.042458 | 0.10185 | 16.47521 | 0.294959 |
| 0.18385 | 12.92581 | 0 | 1.230379 | 0.090973 | 0.079265 | 0.093375 | 0.082504 | 1.342232 |
| 3.189598 | 35.26463 | 0 | 14.02869 | 0.836971 | 17.19828 | 0.780966 | 10.81653 | 167.7111 |
| 0.315991 | 1992.158 | 0.395838 | 3.887184 | 0.128014 | 0.977708 | 0.017917 | 0.130609 | 14.49238 |
| 9.017093 | 307.3604 | 1.688416 | 7.258646 | 2.123462 | 6.363297 | 1.154857 | 1.072936 | 10.42032 |
| 5.011798 | 18.57446 | 0.012467 | 6.505811 | 1.959497 | 0.561201 | 1.02931 | 16.09246 | 19.51171 |
| 1.438306 | 108.2981 | 0.018934 | 3.688491 | 0.495991 | 1.884693 | 0.246828 | 1.849251 | 13.2876 |

| | | | | | | | | |
|---|---|---|---|---|---|---|---|---|
| 6.181687 | 278.2687 | 0.078372 | 2.610466 | 0.582947 | 2.302221 | 0.368933 | 0 | 12.69516 |
| 1.525397 | 8.615444 | 0 | 5.084992 | 0.969471 | 5.08142 | 0.565375 | 2.857465 | 23.19765 |
| 0.788891 | 23.18328 | 0 | 1.946939 | 2.40798 | 0.059864 | 0.82059 | 14.77635 | 0.633715 |
| 0.88343 | 0.31421 | 0.023467 | 3.754327 | 1.024552 | 2.14247 | 0.471624 | 1.672512 | 1.447036 |
| 0.940703 | 3.240452 | 0 | 2.153115 | 0.149011 | 0.21639 | 0.407851 | 0.405417 | 1.578429 |
| 14.1629 | 16.10952 | 0.031587 | 10.26599 | 1.869362 | 2.523276 | 0.960781 | 6.253293 | 24.99545 |
| 3.710173 | 11.86653 | 0 | 3.013677 | 0.636414 | 0.602312 | 0.589734 | 1.671797 | 11.39054 |
| 0.334427 | 1.525066 | 0 | 0.557927 | 0.527347 | 0.011682 | 0.500401 | 0.243181 | 0.865632 |
| 10.0839 | 1125.98 | 0 | 4.954492 | 0.915384 | 1.067643 | 0.518966 | 4.665753 | 20.26947 |
| 0.418909 | 50.20976 | 0.060796 | 2.799738 | 0.575093 | 3.040208 | 0.181622 | 1.384128 | 44.07059 |
| 0.395172 | 487.8057 | 0.058784 | 2.064827 | 0.102659 | 1.587681 | 0.025544 | 0.15517 | 3.642035 |
| 4.899039 | 14.18047 | 0 | 20.85004 | 0.323648 | 8.476142 | 1.310179 | 0.338676 | 80.65941 |
| 1.257965 | 14.78994 | 0 | 6.106273 | 0.373056 | 0.926379 | 0.417712 | 1.48864 | 22.95733 |
| 0.671458 | 23.17416 | 0 | 1.122147 | 0.219095 | 0.457054 | 0.122661 | 0.137561 | 24.24092 |
| 0.413998 | 83.64505 | 0 | 1.42452 | 0.672184 | 1.805836 | 0.055752 | 10.09241 | 41.36524 |
| 0.848991 | 10.18807 | 0.024763 | 4.749033 | 1.369447 | 4.254713 | 0.645631 | 3.202951 | 25.63114 |
| 1.606637 | 17.36279 | 0 | 1.447396 | 0.390527 | 0.223301 | 0.218637 | 0.332036 | 3.250294 |
| 7.683752 | 36.76862 | 0 | 14.54816 | 1.339362 | 1.913667 | 2.363915 | 2.192594 | 80.4185 |
| 12.94223 | 264.1767 | 0 | 20.50711 | 4.11059 | 7.473978 | 0.989687 | 2.665561 | 371.9461 |
| 0.304535 | 5.272922 | 0 | 0.571566 | 0.05494 | 0.951396 | 0.061516 | 0.523163 | 44.02095 |
| 2.343742 | 9.018016 | 0 | 9.885817 | 1.669032 | 9.379805 | 0.722611 | 2.664036 | 163.1233 |
| 10.28587 | 164.72 | 0.079417 | 6.359291 | 13.9717 | 1.94688 | 1.898005 | 3.493851 | 42.09083 |
| 5.559611 | 319.4312 | 0.067975 | 6.906801 | 1.011222 | 7.498768 | 1.033811 | 1.016767 | 7.11887 |
| 0.184449 | 520.3159 | 0.170305 | 1.050485 | 0.220306 | 0.035991 | 0.113061 | 0.524468 | 5.820206 |
| 1.516306 | 10.74551 | 0 | 3.042138 | 0.999866 | 0.986249 | 0.084495 | 1.693807 | 3.197368 |
| 1.048594 | 52.2751 | 0 | 0.961021 | 0.275158 | 0.102748 | 0.088027 | 1.015995 | 2.141386 |
| 1.339768 | 111.3216 | 0 | 3.799744 | 0.171876 | 4.913862 | 0.866024 | 1.636689 | 3.31614 |
| 1.755257 | 8.201343 | 0.254738 | 32.75706 | 0.296576 | 55.99364 | 0.567298 | 1.512936 | 39.4001 |
| 6.375101 | 100.033 | 0 | 3.915844 | 0.669147 | 1.708096 | 0.515107 | 1.365422 | 18.85934 |
| 0.773639 | 2.503582 | 0 | 0.650826 | 0.285075 | 0.020699 | 0.203933 | 3.705686 | 1.617702 |
| 1.572392 | 1.134559 | 0 | 0.74555 | 0.307128 | 0.0892 | 0.324787 | 0.185691 | 0.748778 |
| 0.174431 | 7.785428 | 0.051896 | 3.915461 | 0.251745 | 5.905894 | 0.155034 | 0.924652 | 12.74281 |
| 4.603737 | 174.5811 | 0.14571 | 8.889709 | 5.183479 | 3.171733 | 1.934653 | 14.7011 | 89.94294 |
| 0.385912 | 167.2651 | 0 | 3.830181 | 0.358721 | 10.98055 | 0.04228 | 0.128418 | 8.971016 |
| 0.126407 | 6.078133 | 0.010447 | 1.054432 | 0.415548 | 0.364276 | 0.124834 | 0.165452 | 0.230058 |
| 0.468249 | 7.102288 | 0 | 1.558971 | 0.235059 | 0.172806 | 0.087732 | 4.130295 | 0.741044 |
| 2.279932 | 11.68137 | 0 | 1.496093 | 0.438731 | 0.191134 | 0.218333 | 5.106226 | 1.069626 |
| 1.434201 | 19.75486 | 0 | 4.033388 | 0.821385 | 3.682151 | 0.662189 | 2.055978 | 13.23747 |
| 10.30675 | 430.6959 | 1.934188 | 10.1423 | 4.073479 | 1.67492 | 2.588033 | 4.91877 | 24.31035 |
| 1.87067 | 0.816555 | 0 | 0.72727 | 0.192844 | 0.535582 | 0.296901 | 2.0987 | 0.948415 |
| 0.95894 | 64.44174 | 0.06484 | 1.461641 | 0.65005 | 0.486456 | 0.164356 | 0.057052 | 10.72531 |
| 1.012074 | 35.37156 | 0 | 0.831482 | 0.220477 | 0.01801 | 0.092576 | 1.349677 | 0.542157 |
| 0.576117 | 22.27757 | 0 | 1.15614 | 0.575634 | 0.156735 | 0.402837 | 0.500295 | 0.145179 |
| 0.741149 | 56.9949 | 0 | 2.618394 | 0.125842 | 0.328938 | 0.246584 | 0.513569 | 3.570537 |
| 0.899005 | 11.4314 | 0 | 2.070422 | 0.399974 | 0.835121 | 0.131079 | 0.026542 | 8.089778 |
| 0.120928 | 3.795338 | 0 | 0.161396 | 0.126048 | 0.196419 | 0.054283 | 0.15828 | 0.220085 |
| 10.78449 | 132.847 | 0.084213 | 5.695055 | 1.685139 | 6.954249 | 0.834791 | 0.750239 | 16.52107 |
| 2.363249 | 1.043359 | 0 | 3.833108 | 1.326393 | 1.32079 | 0.919389 | 1.432042 | 3.472697 |
| 0.537784 | 1.988948 | 0 | 1.520448 | 0.421102 | 0.469426 | 0.184906 | 2.740696 | 2.373992 |
| 0.807429 | 0.176448 | 0 | 1.867275 | 0.508986 | 0.083153 | 0.517421 | 0.564856 | 1.641783 |
| 0.345335 | 154.8767 | 0.012379 | 1.874143 | 0.222178 | 0.192277 | 0.31601 | 0.375761 | 0.681507 |
| 4.530268 | 90.86795 | 0 | 5.494437 | 1.609753 | 2.147105 | 1.234552 | 3.989716 | 6.823989 |
| 17.99491 | 128.8932 | 0.192234 | 7.80008 | 2.294018 | 1.535653 | 1.670647 | 0.68503 | 48.62791 |
| 0.131896 | 12.87465 | 0 | 0.612119 | 0.138441 | 0.192245 | 0.155013 | 1.883301 | 0.549925 |
| 1.440218 | 28.69506 | 0 | 0.805001 | 0.348958 | 0.069402 | 0.237835 | 1.011334 | 2.755082 |
| 0.49293 | 1.199349 | 0 | 3.376807 | 2.200935 | 1.685236 | 0.572536 | 2.05653 | 27.01446 |
| 3.374299 | 1147.632 | 0 | 2.059977 | 2.72929 | 0.244798 | 0.539293 | 5.387225 | 2.510439 |
| 10.49348 | 311.4508 | 1.253028 | 13.07617 | 5.130608 | 2.881469 | 2.076419 | 3.251483 | 107.9768 |

| | | | | | | | | |
|---|---|---|---|---|---|---|---|---|
| 0.518412 | 0.46934 | 0 | 1.056378 | 0.502357 | 0.216524 | 0.62233 | 13.90037 | 0.485226 |
| 12.98878 | 2458.905 | 0.019206 | 26.20992 | 2.049748 | 10.08251 | 0.834585 | 3.853056 | 52.9274 |
| 0.644072 | 265.7732 | 0.012607 | 0.343561 | 0.146771 | 0.055948 | 0.05478 | 3.06147 | 0.018508 |
| 0.163963 | 42.00918 | 0 | 0.123093 | 0.118319 | 0.121501 | 0 | 2.851217 | 0.792912 |
| 0.187685 | 0.123577 | 0 | 1.140026 | 0 | 0.128734 | 0 | 0.066997 | 0.354002 |
| 0.698903 | 37.92183 | 0 | 11.73889 | 0.683852 | 1.943936 | 0.172285 | 4.511882 | 2.664697 |
| 1.820806 | 50.30224 | 0 | 4.980566 | 1.144807 | 2.975421 | 0.553524 | 14.15773 | 30.90707 |
| 0.08543 | 1436.326 | 0 | 1.014511 | 0.291427 | 1.530852 | 0.025101 | 0.213471 | 5.325917 |
| 0.286328 | 13.06493 | 0.017037 | 0.361129 | 0.264473 | 0.032405 | 0.101795 | 0.067459 | 0.062533 |
| 4.377833 | 32.67874 | 0 | 3.065767 | 1.248665 | 0.440626 | 0.503328 | 6.930428 | 1.73837 |
| 0.290245 | 22.03768 | 0 | 0.639167 | 0.325804 | 0.182491 | 0.083384 | 0.455875 | 0.323985 |
| 0.988531 | 664.6602 | 0 | 2.062097 | 0.196033 | 0.170804 | 0.048777 | 0.148153 | 10.66275 |
| 9.867289 | 212.0036 | 0 | 9.949779 | 2.898132 | 5.962408 | 1.28683 | 16.74885 | 69.36774 |
| 2.717321 | 2.746161 | 0 | 6.779861 | 0.621839 | 27.75155 | 0.603439 | 1.082785 | 130.2837 |
| 8.252111 | 7.89883 | 0 | 16.75699 | 1.232947 | 19.81687 | 1.204296 | 1.855683 | 41.80068 |
| 0.615624 | 1.505822 | 0.016066 | 0.61623 | 0.202638 | 0.071303 | 0.157081 | 0.593731 | 0.872747 |
| 1.119452 | 8.092649 | 0.951577 | 6.205173 | 0.388724 | 2.000425 | 0.204026 | 0.594905 | 5.900716 |
| 1.621025 | 0.844362 | 0 | 21.53862 | 0.320482 | 63.64518 | 0.645921 | 0.523166 | 8.292945 |
| 0.899678 | 269.1759 | 0.199576 | 5.652218 | 9.419434 | 0.446582 | 0.752444 | 225.7216 | 47.174 |
| 0.204194 | 0.284408 | 0 | 1.202785 | 0.362708 | 0.125918 | 0.203062 | 0.308383 | 2.487042 |
| 2.018555 | 70.08328 | 0 | 4.705179 | 0.814895 | 4.965711 | 0.43341 | 5.099336 | 35.39096 |
| 0.832969 | 373.2672 | 0 | 4.055247 | 0.255006 | 1.642601 | 0.08158 | 3.568114 | 16.20721 |
| 1.99478 | 99.21108 | 0.095722 | 1.98064 | 1.253731 | 1.031692 | 0.36395 | 6.31676 | 2.740398 |
| 0.215406 | 6.227498 | 0.021362 | 0.355769 | 0.310881 | 0.027087 | 0.116032 | 3.214118 | 0.062725 |
| 0.069773 | 0.436438 | 0 | 0.257145 | 0.027463 | 0.047858 | 0.01025 | 0.024907 | 0.013853 |
| 3.492957 | 941.8676 | 0 | 4.861117 | 2.374224 | 2.550414 | 0.279196 | 4.011441 | 48.21539 |
| 2.168332 | 9.496281 | 0 | 2.421727 | 1.002029 | 0.856282 | 0.189873 | 2.264877 | 12.15383 |
| 0.16764 | 29.34569 | 0.064355 | 0.58461 | 0.085851 | 0.448813 | 0.017478 | 0.063703 | 4.452484 |
| 15.74285 | 119.3636 | 0 | 12.69855 | 4.767144 | 10.94485 | 1.466425 | 15.45413 | 160.8223 |
| 0.679924 | 94.61653 | 0 | 2.589573 | 0.167537 | 1.595308 | 0.160793 | 0.390705 | 11.04643 |
| 0.583557 | 9.985217 | 0 | 1.59906 | 1.094874 | 0.31646 | 0.188605 | 2.692418 | 11.75697 |
| 0.534249 | 57.19488 | 0 | 1.01273 | 0.298781 | 0.100772 | 0.086334 | 0.026223 | 0.204187 |
| 1.306658 | 165.938 | 0 | 1.941372 | 1.352653 | 1.961417 | 0.674371 | 30.13995 | 48.01978 |
| 0.350685 | 422.8427 | 0 | 0.833696 | 0.506121 | 0.013781 | 0.070838 | 1.549139 | 1.659413 |
| 26.12173 | 11.61011 | 0.012583 | 3.645231 | 0.903428 | 1.132872 | 0.300738 | 1.29542 | 23.9613 |
| 0.664788 | 26.21662 | 0 | 0.592131 | 0.162618 | 0.913898 | 0.009104 | 1.172461 | 9.006542 |
| 0.252157 | 5.986214 | 0 | 0.420675 | 0.181961 | 0.052848 | 0 | 0 | 0.152973 |
| 1.972318 | 97.68537 | 0 | 1.638635 | 0.797026 | 0.173613 | 0.12749 | 0.051631 | 1.80914 |
| 2.228362 | 2.332838 | 0 | 2.881957 | 0.645108 | 2.3124 | 0.148715 | 2.581115 | 9.575419 |
| 0.17085 | 3.75645 | 0 | 1.148263 | 0.129159 | 0.606164 | 0.026295 | 1.405623 | 10.42987 |
| 11.44629 | 388.9003 | 0.498483 | 4.899031 | 2.036325 | 0.973069 | 1.011786 | 22.16109 | 5.912541 |
| 2.648352 | 34.77355 | 0 | 4.5445 | 0.973354 | 1.869411 | 0.505061 | 11.65865 | 41.0442 |
| 0.642465 | 30.57045 | 0 | 1.72739 | 0.797848 | 0.267734 | 0.144868 | 1.87738 | 5.808272 |
| 17.56782 | 1678.876 | 4.084067 | 15.46441 | 2.611033 | 4.490135 | 0.589846 | 1.620186 | 122.3463 |
| 2.636641 | 142.3608 | 0 | 3.505732 | 0.320928 | 2.771294 | 0.256675 | 0.311842 | 30.97706 |
| 10.29421 | 530.5138 | 0.189054 | 7.880916 | 2.311085 | 1.114703 | 1.930528 | 0.74855 | 24.64716 |
| 1.786725 | 161.0567 | 0.007404 | 4.700375 | 1.709533 | 1.544284 | 0.579077 | 2.97049 | 7.554296 |
| 2.006432 | 12.92746 | 0.024873 | 1.079519 | 0.217182 | 0.141923 | 0.216159 | 0.459581 | 0.511231 |
| 2.246099 | 115.8035 | 0 | 2.1514 | 0.111781 | 0.931342 | 0.187742 | 1.292531 | 0.718895 |
| 1.858389 | 141.8702 | 0.092149 | 1.720702 | 0.655623 | 0.516069 | 0.183526 | 0.04054 | 1.059761 |
| 0.693994 | 143.0134 | 0.029496 | 5.160459 | 0.934824 | 6.495343 | 0.395192 | 18.37473 | 112.563 |
| 15.70048 | 723.945 | 0.374813 | 17.44988 | 3.772793 | 5.242764 | 0.687103 | 3.091782 | 28.75211 |
| 1.776093 | 149.3498 | 0 | 3.904901 | 1.403723 | 1.754838 | 0.820045 | 1.245371 | 39.29722 |
| 3.732515 | 345.2138 | 0.237789 | 5.544279 | 0.54215 | 3.037789 | 0.297066 | 0.251071 | 27.77158 |
| 4.635836 | 101.2029 | 0.146207 | 10.89592 | 2.009545 | 3.398852 | 0.714736 | 4.438251 | 44.61222 |
| 16.75269 | 34.23714 | 0 | 17.6436 | 4.656669 | 2.205864 | 2.550122 | 5.477205 | 47.32654 |
| 0.814681 | 180.6779 | 0 | 2.109009 | 0.445985 | 1.165763 | 0.113493 | 5.515451 | 17.11748 |
| 0.941873 | 43.25599 | 0 | 0.771382 | 0.123577 | 0.242264 | 0.138369 | 0.336218 | 1.870017 |

| | | | | | | | | |
|---|---|---|---|---|---|---|---|---|
| 0.173853 | 32.50939 | 0 | 0.47461 | 0.11405 | 0.149058 | 0.153243 | 1.179134 | 5.004976 |
| 2.193463 | 58.06575 | 0 | 3.655711 | 2.627518 | 0.972291 | 0.035446 | 16.36459 | 9.676706 |
| 0.570537 | 5.986097 | 0 | 2.112283 | 0.710622 | 0.560199 | 0.315749 | 0.767224 | 11.31672 |
| 0.8634 | 256.2238 | 0 | 11.22388 | 0.163959 | 2.303589 | 0.312096 | 0.089217 | 8.981585 |
| 1.00058 | 90.29438 | 0 | 1.284264 | 0.62887 | 0.228307 | 0.130398 | 0.253479 | 2.749162 |
| 1.737381 | 2354.93 | 0.457429 | 2.931831 | 0.554746 | 1.899573 | 0.198768 | 0.120745 | 10.37579 |
| 48.01089 | 1464.73 | 2.008592 | 19.70914 | 7.202912 | 2.014958 | 5.468818 | 8.926082 | 57.52233 |
| 2.268037 | 9.463255 | 0 | 54.38777 | 0.948786 | 58.7925 | 0.451503 | 6.969732 | 112.67 |
| 0.443521 | 0.347999 | 0 | 1.442861 | 0.170695 | 0.780816 | 0.334473 | 0.116103 | 2.873615 |
| 1.05524 | 92.46651 | 0 | 3.144829 | 0.576879 | 1.146011 | 0.516746 | 0.565029 | 9.462858 |
| 0.298676 | 84.10849 | 0 | 1.580271 | 0.24632 | 2.977846 | 0.114919 | 4.41194 | 15.77941 |
| 0.996459 | 224.4726 | 0 | 1.49616 | 0.761361 | 0.276407 | 0.094722 | 6.674671 | 3.328356 |
| 0.819388 | 35.23151 | 0 | 2.157343 | 0.582957 | 0.756457 | 0.354344 | 4.237056 | 2.810296 |
| 0.674111 | 0.441294 | 0 | 1.44817 | 0.404128 | 1.74103 | 0.134075 | 0.753375 | 7.746226 |
| 1.543121 | 20.66529 | 0 | 5.138603 | 0.749712 | 1.786768 | 0.296278 | 8.279018 | 29.56365 |
| 0.962124 | 18.81613 | 0 | 1.966103 | 0.916111 | 1.362983 | 0.141931 | 4.232518 | 11.01192 |
| 0.699024 | 32.5548 | 0.703424 | 1.28109 | 0.356067 | 0.048475 | 0.880439 | 0.766934 | 1.167433 |
| 7.921949 | 863.8357 | 0.021119 | 11.14854 | 5.737109 | 0.696256 | 2.064812 | 5.128695 | 34.4475 |
| 6.472577 | 72.98109 | 0.343105 | 5.692527 | 0.37449 | 10.27828 | 0.302841 | 0.113209 | 5.446566 |
| 0.237109 | 9.469106 | 0.019595 | 0.5538 | 0.209125 | 0.124235 | 0.021287 | 0.155174 | 0.316457 |
| 1.168048 | 5.496515 | 0.009817 | 2.546472 | 1.114321 | 9.951847 | 0.095978 | 2.954019 | 14.74378 |
| 2.250248 | 45.06065 | 0.022875 | 6.387965 | 0.77677 | 4.660268 | 0.969152 | 0.905732 | 12.8403 |
| 1.988158 | 99.28208 | 0.107764 | 2.4534 | 1.312712 | 0.523807 | 1.430826 | 1.801565 | 0.720748 |
| 1.980229 | 8.861664 | 0.044802 | 3.651579 | 1.651735 | 0.156225 | 0.839555 | 2.306086 | 3.272341 |
| 3.368571 | 127.4571 | 0.011261 | 4.887341 | 1.212679 | 1.720563 | 0.599407 | 46.3694 | 27.29471 |
| 1.096686 | 49.82426 | 0.029662 | 2.305307 | 0.661888 | 0.188056 | 0.547783 | 8.142805 | 3.919294 |
| 14.35576 | 88.16706 | 1.82512 | 8.484081 | 3.348726 | 1.755377 | 2.22547 | 5.53867 | 22.22016 |
| 1.137757 | 21.16301 | 0.009616 | 7.648591 | 2.304469 | 5.694447 | 0.679032 | 3.223752 | 65.38187 |
| 2.979725 | 112.5056 | 0 | 6.345356 | 1.617391 | 0.261763 | 0.964737 | 1.850664 | 2.962167 |
| 2.553985 | 30.31459 | 0.009546 | 4.663363 | 1.583685 | 2.420824 | 0.757004 | 3.628432 | 25.33842 |
| 0.235085 | 54.53359 | 0 | 2.339713 | 0.184182 | 1.34295 | 0.108542 | 8.835318 | 7.965294 |
| 0.858517 | 288.9152 | 0.036489 | 2.136128 | 0.212407 | 0.94849 | 0.198194 | 1.059485 | 2.249966 |
| 0.85842 | 2.033851 | 0 | 4.922488 | 0.448113 | 3.238378 | 0.295149 | 4.016159 | 16.83294 |
| 1.929843 | 285.4105 | 0.012321 | 2.051958 | 0.537918 | 0.538993 | 0.441693 | 20.13158 | 7.127022 |
| 0.508809 | 29.51974 | 0.015769 | 1.941745 | 0.566047 | 0.519859 | 0.256948 | 1.956289 | 2.083542 |
| 0.454209 | 297.8988 | 0 | 1.193472 | 0.819413 | 1.941072 | 0.160562 | 2.117924 | 1.534464 |
| 3.555932 | 32.93454 | 0 | 5.430886 | 0.617257 | 2.483566 | 0.503548 | 11.08393 | 4.163235 |
| 1.790169 | 263.6754 | 0 | 14.27029 | 0 | 11.63425 | 0.31559 | 1.022451 | 12.86636 |
| 1.52241 | 17.97394 | 0 | 1.895594 | 0.643082 | 0.31518 | 0.260022 | 0.583216 | 4.865695 |
| 0.065145 | 0.720843 | 0 | 1.021603 | 0.052233 | 0.546127 | 0.245638 | 0.085266 | 2.16572 |
| 0.305871 | 0.775369 | 0 | 0.564231 | 0.18919 | 0.173084 | 0.169469 | 0.92652 | 0.343549 |
| 1.447627 | 5.919772 | 0 | 1.050155 | 0.352124 | 0.122723 | 0.236564 | 2.171535 | 1.065697 |
| 0.421667 | 1.498198 | 0 | 0.537557 | 0.149271 | 0.270125 | 0.089998 | 0.718526 | 2.102446 |
| 1.034015 | 55.88798 | 0 | 5.851103 | 0.964429 | 8.650775 | 0.522887 | 1.24292 | 32.13786 |
| 0.491393 | 49.7513 | 0 | 3.395725 | 1.332946 | 0.011167 | 0.736685 | 0.302214 | 0.762866 |
| 0.666129 | 77.46148 | 0 | 0.83348 | 0.721036 | 0.244316 | 0.029902 | 0.726569 | 0.606168 |
| 0.577355 | 22.80291 | 0.01342 | 0.744979 | 1.718596 | 0.025524 | 0.189516 | 34.0768 | 4.984584 |
| 1.430276 | 37.73242 | 0 | 1.42336 | 0.768084 | 0.721518 | 0.268758 | 1.110175 | 10.24274 |
| 1.54906 | 2.317525 | 0 | 1.975648 | 0.966771 | 0.062048 | 0.889194 | 0.164395 | 1.280089 |
| 1.31244 | 45.9598 | 0 | 2.305599 | 0.492482 | 1.089256 | 0.21209 | 0.721487 | 6.621206 |
| 0.799381 | 130.4291 | 0 | 5.721198 | 0.756311 | 3.576902 | 0.287066 | 8.091338 | 85.19602 |
| 0.575525 | 48.59942 | 0.019458 | 1.630076 | 0.132144 | 2.245178 | 0.105687 | 0.256805 | 8.51282 |
| 7.958966 | 6.112866 | 0 | 8.457064 | 2.221596 | 2.309624 | 2.233123 | 10.44023 | 96.69036 |
| 0.356233 | 1.873052 | 0 | 1.522337 | 0.098871 | 0.872233 | 0.055353 | 0.537999 | 5.266463 |
| 0.641794 | 27.09279 | 0 | 0.403384 | 0.204639 | 0.021115 | 0.084418 | 0.439551 | 0.945303 |
| 0.618741 | 15.00671 | 0 | 1.320194 | 0.516994 | 0.076783 | 0.026313 | 1.918085 | 1.422431 |
| 6.484073 | 102.787 | 0 | 9.421628 | 2.837601 | 1.578135 | 1.892842 | 2.299669 | 29.96649 |
| 6.228615 | 412.6619 | 0.176299 | 12.85175 | 1.425374 | 3.017898 | 0.978878 | 0.982438 | 9.17417 |

| | | | | | | | | |
|---|---|---|---|---|---|---|---|---|
| 0.186571 | 22.95193 | 0.032651 | 0.247175 | 0.126713 | 0.082804 | 0.017735 | 0.21547 | 0.023969 |
| 0.491597 | 94.63664 | 0.03324 | 1.442692 | 0.225747 | 0.737603 | 0.39721 | 2.544521 | 2.342469 |
| 0.470001 | 10.32328 | 0 | 2.31141 | 0.707815 | 1.227019 | 0.231158 | 2.0595 | 2.097562 |
| 1.43192 | 155.133 | 0 | 0.836109 | 0.841947 | 0.050018 | 0.085703 | 1.874212 | 0.521211 |
| 1.288451 | 3.277275 | 0 | 8.095289 | 0.819082 | 8.4266 | 0.495746 | 2.619986 | 100.4641 |
| 1.790263 | 0.18929 | 0 | 4.473521 | 4.18684 | 0.332758 | 0.802454 | 255.4815 | 17.20914 |
| 3.434415 | 539.5529 | 0.010137 | 4.154002 | 2.330815 | 0.661953 | 0.682738 | 8.829931 | 13.40889 |
| 1.457708 | 11.91101 | 0 | 2.622014 | 0.832983 | 2.644914 | 0.155449 | 3.486638 | 28.10282 |
| 1.130997 | 31.44671 | 0 | 1.240967 | 0.753367 | 0.030769 | 0.4042 | 4.48372 | 0.308757 |
| 3.822756 | 33.5593 | 0.76238 | 1.728241 | 0.957921 | 0.103009 | 0.420889 | 8.841402 | 0.972494 |
| 7.813459 | 300.2875 | 0 | 5.821084 | 1.334261 | 1.561 | 1.421688 | 1.053915 | 3.19142 |
| 1.290098 | 105.0892 | 0 | 2.224022 | 0.942451 | 0.225319 | 0.025738 | 2.063824 | 11.20055 |
| 0.166503 | 25.80702 | 0 | 2.962502 | 0.564712 | 5.88872 | 0.04036 | 4.576579 | 89.96368 |
| 0.520839 | 13.66361 | 0 | 3.028083 | 0.454512 | 0.965294 | 0.156591 | 0.570739 | 9.179317 |
| 4.341857 | 388.0681 | 0 | 4.022483 | 1.266597 | 1.176195 | 0.5225 | 1.450971 | 40.25005 |
| 0.620179 | 62.58086 | 0.00961 | 1.163979 | 0.223766 | 0.097484 | 0.062638 | 0.152201 | 0.084653 |
| 1.243775 | 7.748234 | 0 | 2.28991 | 0.619723 | 2.248311 | 0.167495 | 0.813976 | 2.263635 |
| 0.377335 | 82.15012 | 0 | 2.151004 | 0.756878 | 1.48984 | 0.403069 | 4.570538 | 3.938852 |
| 0.425893 | 194.4991 | 0 | 2.363251 | 0.454317 | 1.624139 | 0.209465 | 0.654389 | 22.72765 |
| 0.518903 | 99.74555 | 0 | 2.095568 | 0.550915 | 3.678857 | 0.173492 | 2.271748 | 4.038079 |
| 3.120802 | 34.10428 | 0 | 7.189047 | 1.203069 | 2.020674 | 0.601177 | 1.136157 | 24.20861 |
| 0.130933 | 2.192968 | 0 | 0.344037 | 0.165346 | 0.061743 | 0.052897 | 0.44986 | 1.179555 |
| 0.574912 | 2.763987 | 0 | 1.711549 | 0.844038 | 0.355242 | 0.336382 | 1.245501 | 0.974162 |
| 13.55252 | 18.1024 | 0 | 10.1744 | 3.463659 | 3.328564 | 2.737603 | 7.886636 | 22.49299 |
| 1.98868 | 23.96828 | 0 | 5.15544 | 1.547181 | 0.923131 | 0.376605 | 5.734611 | 8.584624 |
| 6.400854 | 1174.637 | 0 | 11.17005 | 2.987881 | 5.089338 | 2.040231 | 1.812409 | 64.5595 |
| 0.677874 | 43.45047 | 0 | 0.848176 | 0.782665 | 0.170485 | 0.146059 | 0.975982 | 0.937621 |
| 3.449796 | 108.0437 | 0 | 2.819163 | 1.738523 | 1.408106 | 0.621459 | 8.283113 | 10.38736 |
| 4.021839 | 2.720442 | 0 | 0.966805 | 0.420399 | 0.428951 | 0.264265 | 0.541795 | 1.897338 |
| 2.081373 | 853.221 | 0 | 0.866878 | 0.483147 | 0.027454 | 0.344973 | 0.133356 | 0.105959 |
| 2.146818 | 32.31167 | 0.230165 | 3.775149 | 1.609661 | 3.198193 | 0.28129 | 3.442795 | 14.67116 |
| 2.407951 | 35.65885 | 0 | 2.582487 | 1.396302 | 3.507871 | 0.10423 | 3.123579 | 14.22715 |
| 0.47162 | 83.82979 | 0 | 0.668786 | 1.418038 | 0.061777 | 0.105852 | 8.179143 | 0.48639 |
| 13.9759 | 52.65223 | 0.040885 | 7.613838 | 2.102335 | 0.550828 | 1.532315 | 1.834674 | 6.932926 |
| 1.185364 | 332.754 | 0 | 3.200872 | 1.352693 | 0.060664 | 0.749883 | 0.306692 | 2.016851 |
| 1.227376 | 8.986598 | 0.007973 | 6.792083 | 3.078664 | 5.995075 | 0.961403 | 59.20153 | 61.32481 |
| 0.903885 | 442.9478 | 0.007268 | 0.968353 | 0.359624 | 0.327166 | 0.118433 | 0.422071 | 6.67977 |
| 6.393988 | 276.7817 | 0.174924 | 5.749218 | 1.304653 | 0.64462 | 0.819487 | 2.68384 | 21.50815 |
| 0.65933 | 1.095521 | 0 | 2.434157 | 0.559746 | 1.353392 | 0.22563 | 1.431538 | 5.437943 |
| 0.966867 | 34.02172 | 0.047943 | 1.403337 | 0.558167 | 0.334354 | 0.156245 | 0.253103 | 14.49968 |
| 1.0976 | 205.1537 | 0.07559 | 1.510686 | 0.586702 | 0.632605 | 0.303831 | 10.57518 | 2.596882 |
| 2.919061 | 92.6415 | 0 | 1.219836 | 1.049815 | 0.240556 | 0.15266 | 2.262745 | 3.074091 |
| 1.239297 | 64.59435 | 0 | 3.778512 | 0.310267 | 7.120187 | 0.081743 | 0.148968 | 9.22447 |
| 0.528454 | 52.83428 | 0 | 1.343879 | 1.731497 | 0.408595 | 0.176951 | 48.24953 | 9.825655 |
| 1.628687 | 208.0237 | 0 | 2.726925 | 1.31903 | 0.596739 | 0.681657 | 8.879764 | 10.31273 |
| 0.36687 | 0.773347 | 0.008145 | 1.397671 | 0.276594 | 0.557737 | 0.035395 | 1.311562 | 0.562058 |
| 0.061696 | 0.670272 | 0 | 0.254747 | 0.055651 | 0.08728 | 0.074775 | 0.030282 | 0.101057 |
| 2.383451 | 118.1261 | 0.02026 | 1.94272 | 0.746946 | 1.207435 | 1.320564 | 1.283514 | 16.35975 |
| 3.369828 | 62.79813 | 0 | 1.791328 | 0.392701 | 0.927786 | 0.169119 | 1.972488 | 2.37701 |
| 6.586325 | 730.8531 | 0.188415 | 9.603945 | 2.102204 | 0.985511 | 0.614047 | 0.994697 | 23.85859 |
| 4.611311 | 178.4731 | 0 | 2.884906 | 1.941102 | 0.226512 | 0.077624 | 4.715355 | 3.566793 |
| 1.666829 | 59.64048 | 0 | 2.511132 | 1.255462 | 0.502836 | 0.580437 | 4.032803 | 6.226503 |
| 8.251123 | 928.8744 | 0 | 7.567254 | 1.180103 | 1.205831 | 0.720745 | 1.692931 | 35.74812 |
| 0.752277 | 91.30918 | 0 | 1.167815 | 0.717675 | 0.012025 | 0.123628 | 0.750997 | 1.253095 |
| 2.490671 | 66.68494 | 0 | 5.752444 | 2.163644 | 1.660763 | 0.974183 | 25.69579 | 27.16306 |
| 1.312888 | 93.17294 | 0.058299 | 6.06092 | 0.989824 | 1.644791 | 0.569988 | 2.539147 | 12.71028 |
| 1.411085 | 196.2042 | 0 | 1.06502 | 0.337606 | 0.241968 | 0.402407 | 1.214833 | 0.60976 |
| 1.356924 | 178.1603 | 0.025231 | 3.208889 | 1.713566 | 1.679666 | 0.520786 | 4.329126 | 13.81721 |

| | | | | | | | | |
|---|---|---|---|---|---|---|---|---|
| 0.418319 | 42.94114 | 0 | 0.724726 | 0.603732 | 0.267063 | 0.1664 | 1.086634 | 1.574192 |
| 0.08187 | 0.889446 | 0 | 0.358535 | 1.457282 | 0.656316 | 0.099226 | 1.125162 | 7.003066 |
| 2.033286 | 507.1005 | 0.073772 | 4.374628 | 0.894668 | 2.373661 | 0.59104 | 0.657218 | 15.59635 |
| 0.380835 | 21.40605 | 0 | 1.119297 | 0.853688 | 0.634286 | 0.104754 | 29.43078 | 11.16642 |
| 2.02319 | 269.4415 | 0.055223 | 1.114779 | 0.375038 | 1.347935 | 0.089985 | 2.988239 | 10.13435 |
| 0.544989 | 406.8028 | 0.454787 | 2.414946 | 0.441234 | 0.513991 | 0.236285 | 1.409252 | 5.428639 |
| 1.753818 | 97.21454 | 0 | 1.529022 | 4.633678 | 0.133392 | 0.845677 | 1.666116 | 4.417175 |
| 0.471234 | 268.3011 | 0 | 0.746854 | 0.2267 | 0.148144 | 0 | 0.411192 | 0.971982 |
| 0.814398 | 1.054761 | 0.016826 | 4.271308 | 0.530548 | 0.202687 | 0.539221 | 3.530959 | 4.792387 |
| 2.045476 | 55.35316 | 0 | 3.634129 | 0.920753 | 0.911841 | 0.191314 | 0.335737 | 15.1829 |
| 10.06368 | 135.575 | 0.123959 | 4.128887 | 1.147139 | 2.448387 | 1.04621 | 2.114256 | 11.99731 |
| 0.994838 | 28.76595 | 0 | 3.837726 | 0.839382 | 0.577387 | 0.519396 | 6.0699 | 4.779922 |
| 1.550122 | 503.3206 | 7.290403 | 7.382081 | 0.881318 | 2.126479 | 0.809694 | 1.414099 | 16.6193 |
| 5.155114 | 192.7114 | 0 | 16.29238 | 6.834616 | 5.609211 | 1.79267 | 97.22152 | 29.90754 |
| 1.50361 | 81.71685 | 0.098679 | 2.312969 | 0.574429 | 0.715995 | 0.238218 | 0.31836 | 2.752624 |
| 0.567777 | 87.29197 | 0.134369 | 0.697503 | 0.167612 | 0.066935 | 0.302366 | 0.532029 | 5.382751 |
| 2.564961 | 157.0551 | 0 | 1.377963 | 0.91697 | 0.210837 | 0.47534 | 1.617013 | 1.284814 |
| 0.981958 | 1124.525 | 0 | 4.309745 | 1.548015 | 2.293894 | 0.927691 | 7.830231 | 57.34229 |
| 0.87403 | 28.32431 | 0.016669 | 1.817079 | 1.803199 | 0.052841 | 0.814863 | 5.280002 | 3.560741 |
| 0.75849 | 5.587181 | 0.025643 | 0.440012 | 0.895648 | 0.048774 | 0.167143 | 0.473824 | 0.715314 |
| 0.734428 | 402.3562 | 0 | 1.198616 | 0.414764 | 0.722771 | 0.025801 | 1.065766 | 17.71337 |
| 0.680781 | 13.55946 | 0.029496 | 2.540555 | 0.915754 | 0.710629 | 0.405877 | 0.570971 | 7.174182 |
| 0.875021 | 35.54304 | 0 | 0.974419 | 0.27362 | 0.701464 | 0.129619 | 2.46239 | 26.91347 |
| 0.879966 | 10.60295 | 0 | 2.089974 | 0.392545 | 0.754469 | 0.491244 | 2.13601 | 7.023362 |
| 1.427465 | 1.011399 | 0 | 1.001762 | 0.671794 | 0.395102 | 0.225663 | 2.55887 | 2.677014 |
| 0.695676 | 9.206887 | 0 | 1.557316 | 0.556777 | 0.936445 | 0.214622 | 0.844335 | 2.251375 |
| 4.339005 | 179.6725 | 0 | 4.898581 | 0.934089 | 0.815951 | 0.469589 | 12.00679 | 9.923367 |
| 4.988777 | 8.127489 | 0 | 6.853645 | 0.694414 | 0.459755 | 0.634307 | 0.248593 | 8.309753 |
| 3.362578 | 409.4557 | 0.454733 | 4.165286 | 0.419122 | 1.549623 | 0.185247 | 0.630174 | 2.052913 |
| 0.954249 | 0.203276 | 0 | 1.601345 | 0.594089 | 1.623481 | 0.211656 | 15.35532 | 9.602959 |
| 0.058575 | 4.318195 | 0 | 0.577952 | 0.012077 | 0.520867 | 0.013522 | 0.032858 | 2.686447 |
| 0.390659 | 2.369655 | 0 | 1.065594 | 0.084572 | 0.718457 | 0.073652 | 0.460194 | 1.052263 |
| 0.060607 | 2.667508 | 0 | 0.223998 | 0.040371 | 0.092334 | 0.007534 | 0.054919 | 0.906175 |
| 0.747277 | 4.582433 | 0 | 0.658251 | 0.747757 | 0.592009 | 0.064405 | 0.117372 | 1.555865 |
| 8.023963 | 48.35741 | 1.316952 | 2.574134 | 0.517166 | 1.663277 | 0.397403 | 0.634559 | 20.65445 |
| 1.30751 | 61.96563 | 0 | 5.221553 | 0.851791 | 5.669025 | 0.352155 | 1.28353 | 80.29269 |
| 2.259717 | 1985.654 | 0.018121 | 3.941631 | 0.90543 | 1.740567 | 0.413399 | 1.60242 | 7.94146 |
| 0.427301 | 78.06207 | 0 | 1.078405 | 0.288666 | 0.471592 | 0.073459 | 0.321292 | 0.516244 |
| 0.743353 | 21.7697 | 0 | 0.994809 | 3.428402 | 0.060963 | 0.574513 | 0.126908 | 0.388218 |
| 5.403501 | 0.014078 | 0 | 5.97694 | 2.648132 | 0.659935 | 0.879486 | 40.02343 | 11.18978 |
| 1.217389 | 21.14942 | 0.024472 | 2.050191 | 1.210891 | 0.899899 | 0.265851 | 2.58392 | 10.06009 |
| 1.975065 | 73.83511 | 0 | 1.779308 | 0.789365 | 0.444189 | 0.31917 | 4.53391 | 7.664713 |
| 0.161224 | 2.262425 | 0.09993 | 0.726223 | 0.310246 | 0.468833 | 0.238826 | 2.057476 | 1.907252 |
| 0.610837 | 112.805 | 0.008492 | 3.394341 | 1.021647 | 0.436109 | 0.341337 | 1.7933 | 12.8667 |
| 1.114854 | 111.5394 | 0 | 3.286985 | 1.843033 | 2.198473 | 0.687884 | 2.546986 | 8.632485 |
| 1.23425 | 50.06029 | 0 | 0.593023 | 0.28501 | 0.139686 | 0 | 1.550866 | 5.391118 |
| 1.937446 | 341.8086 | 0 | 3.405143 | 0.36849 | 2.25219 | 0.533953 | 2.948703 | 3.706501 |
| 1.049028 | 696.1606 | 0.007881 | 2.736522 | 1.643984 | 1.873791 | 0.308223 | 30.54001 | 19.19615 |
| 3.559696 | 128.3214 | 0 | 5.629313 | 1.758073 | 2.795562 | 0.743663 | 10.68254 | 77.1809 |
| 4.05534 | 66.38699 | 0 | 6.783357 | 2.130629 | 5.887995 | 1.130559 | 61.48381 | 40.50268 |
| 0.377661 | 80.87116 | 0 | 1.163942 | 0.200809 | 0.412419 | 0.064242 | 1.404889 | 0.520925 |
| 1.917144 | 44.43057 | 0 | 2.739435 | 1.131442 | 1.142666 | 0.35703 | 2.994384 | 16.60782 |
| 3.903095 | 45.79589 | 0 | 3.867868 | 0.563309 | 1.214762 | 0.441517 | 0.459782 | 47.73567 |
| 7.821165 | 1.100442 | 0 | 6.930825 | 2.245026 | 2.947796 | 1.391339 | 6.363777 | 23.40166 |
| 0.04269 | 0.684641 | 0 | 0.183138 | 0.096819 | 0.023007 | 0.029566 | 0.55078 | 0.013319 |
| 13.07777 | 371.4073 | 0.133858 | 5.607059 | 2.4134 | 0.487983 | 0.860371 | 5.623955 | 11.75865 |
| 1.432921 | 138.0649 | 0 | 3.103798 | 0.915363 | 2.791456 | 0.303684 | 0.276716 | 21.70094 |
| 0.88611 | 101.0812 | 0.01345 | 3.203995 | 0.587234 | 1.833444 | 0.175341 | 0.532566 | 37.02613 |

| | | | | | | | | |
|---|---|---|---|---|---|---|---|---|
| 0.485539 | 39.9528 | 0.217828 | 3.066535 | 1.090051 | 1.540946 | 0.33627 | 2.1789 | 16.17532 |
| 20.52057 | 166.4755 | 0 | 22.84311 | 16.89773 | 5.815169 | 7.075574 | 27.6116 | 46.61674 |
| 2.549884 | 34.70491 | 0 | 1.571002 | 2.740488 | 0.650553 | 1.039544 | 2.130315 | 15.33552 |
| 0.12177 | 30.22313 | 0 | 0.331813 | 0.175743 | 0.008507 | 0.014576 | 4.197084 | 0.078798 |
| 4.874022 | 58.87125 | 0.04421 | 3.770673 | 1.329666 | 0.546571 | 0.576322 | 3.150856 | 4.024214 |
| 0.232426 | 30.72199 | 0.0461 | 1.372662 | 0.536712 | 0.263047 | 0.651038 | 4.441575 | 2.199643 |
| 0.179972 | 4.42254 | 0 | 0.733463 | 0.111318 | 0.630445 | 0 | 0 | 0.767387 |
| 0.477199 | 33.60474 | 0 | 5.267262 | 0.595641 | 1.982682 | 0.166735 | 9.520868 | 9.407837 |
| 0.455341 | 5.533998 | 0 | 1.025526 | 0.308668 | 0.162668 | 0.10034 | 0.677258 | 1.491674 |
| 0.680152 | 54.32159 | 0 | 1.554049 | 0.213396 | 1.115594 | 0.023894 | 0.58059 | 2.680229 |
| 0.854929 | 8.201444 | 0 | 13.62649 | 0.296602 | 0.093035 | 0.225832 | 0.387346 | 0.017953 |
| 4.02082 | 24.18067 | 0 | 14.81455 | 1.045207 | 11.34359 | 1.366936 | 0.341246 | 183.5349 |
| 0.516311 | 153.6496 | 0.02259 | 3.898947 | 0.416412 | 1.160074 | 0.196319 | 2.444769 | 20.56223 |
| 0.69577 | 41.28505 | 0.085459 | 1.792252 | 0.85676 | 3.371281 | 0.082522 | 10.65244 | 23.96408 |
| 0.700137 | 1.334448 | 0 | 1.839668 | 0.771142 | 1.198992 | 0.35729 | 1.049032 | 15.51206 |
| 1.248321 | 68.57161 | 0 | 5.601181 | 2.199655 | 2.669503 | 1.196295 | 25.02152 | 14.61419 |
| 0.215011 | 7.472375 | 0 | 1.916177 | 0.440443 | 1.295187 | 0.156916 | 1.171087 | 5.528891 |
| 2.221835 | 71.82511 | 0 | 2.64582 | 0.884588 | 0.216772 | 0.588095 | 3.986125 | 1.213103 |
| 1.686632 | 10.94726 | 0.0386 | 2.259714 | 0.56174 | 1.101254 | 0.041932 | 0.611335 | 3.570207 |
| 1.066307 | 3.084176 | 0 | 12.33005 | 0.199861 | 9.011741 | 0.246164 | 1.305041 | 51.53557 |
| 0.765185 | 8.759483 | 0 | 2.093564 | 0.490819 | 1.748028 | 0.219829 | 5.441676 | 4.32639 |
| 0.803308 | 2.790335 | 0.26826 | 3.716906 | 0.02366 | 6.030019 | 0.768289 | 0.579362 | 12.17337 |
| 0.127904 | 2.883706 | 0 | 0.16107 | 0.029773 | 0.027239 | 0.033337 | 0.032402 | 0.09912 |
| 1.598784 | 3.5382 | 0 | 0.452275 | 0.200645 | 0.007284 | 0.124813 | 1.516389 | 0.590382 |
| 0.818551 | 183.6638 | 0 | 1.655434 | 0.482188 | 0.535669 | 0.134977 | 2.88618 | 4.961743 |
| 6.526701 | 1421.539 | 0 | 9.423203 | 1.117576 | 3.213367 | 0.56013 | 0.115833 | 10.17919 |
| 0.590004 | 148.0904 | 0.079788 | 0.778498 | 0.580578 | 0.08431 | 0.476722 | 1.263673 | 2.420908 |
| 0.39295 | 8.263467 | 0 | 1.500889 | 0.18904 | 1.4889 | 0.100264 | 0.406045 | 3.477922 |
| 3.129576 | 42.40674 | 0.076633 | 5.018051 | 0.557619 | 1.099248 | 0.291372 | 0.530994 | 3.417443 |
| 0.17046 | 131.8333 | 0 | 1.991894 | 0.192533 | 2.390499 | 0.011977 | 0.698437 | 21.43031 |
| 2.768225 | 144.9002 | 0.053829 | 3.341439 | 0.261124 | 0.784939 | 0.614002 | 4.404765 | 4.781246 |
| 1.77584 | 36.55757 | 0.01016 | 2.389489 | 1.005467 | 0.998459 | 0.430463 | 7.321742 | 3.580038 |
| 1.551691 | 123.7595 | 0.040674 | 5.624431 | 1.726453 | 10.24404 | 0.39767 | 11.81009 | 28.88726 |
| 0.30953 | 8.406914 | 0 | 0.800407 | 0.148908 | 0.16218 | 0.222311 | 0.270092 | 0.938894 |
| 2.172529 | 13.40821 | 0.084491 | 6.993046 | 2.10056 | 1.714162 | 0.504821 | 45.77606 | 10.45081 |
| 1.448814 | 33.04295 | 0 | 1.781944 | 1.267935 | 0.276189 | 0.149443 | 4.418033 | 1.817709 |
| 2.644545 | 24.55638 | 0 | 14.82264 | 1.258912 | 8.585811 | 0.447495 | 23.43236 | 232.4904 |
| 0.524441 | 60.72065 | 0.010988 | 4.247717 | 1.19397 | 1.233047 | 0.513271 | 9.368326 | 21.82639 |
| 4.690448 | 40.65054 | 0 | 8.706382 | 1.336358 | 1.216866 | 0.662308 | 0.536438 | 9.282387 |
| 0.793498 | 532.0011 | 0 | 5.003958 | 0.429452 | 1.721241 | 0.320572 | 0.545262 | 10.39783 |
| 0.419639 | 2.402054 | 0.010068 | 0.802842 | 0.166062 | 0.989428 | 0.043751 | 0.132885 | 0.93126 |
| 1.609261 | 26.53792 | 0 | 1.964629 | 0.75971 | 0.241135 | 0.267347 | 0.767728 | 4.22077 |
| 0.173657 | 0.444551 | 0 | 0.575432 | 0.077781 | 0.355798 | 0.135476 | 0.094054 | 0.745446 |
| 0.888766 | 3.266584 | 0 | 5.158588 | 0.938093 | 9.708279 | 0.471602 | 0.937576 | 8.517374 |
| 0.776036 | 18.3825 | 0 | 1.556259 | 0.30685 | 0.235611 | 0.154611 | 2.441945 | 0.859026 |
| 1.732854 | 1.041255 | 0 | 0.99008 | 0.22132 | 0.802684 | 0.594751 | 0.24086 | 4.437569 |
| 1.410337 | 147.9114 | 0 | 3.455012 | 0.830249 | 1.365123 | 0.119953 | 2.550343 | 8.510881 |
| 2.132879 | 34.51426 | 0 | 2.275679 | 0.776177 | 1.786777 | 0.355536 | 1.607818 | 20.38101 |
| 27.47724 | 18.26359 | 0 | 15.45196 | 2.647679 | 4.772518 | 1.818444 | 12.854 | 72.13334 |
| 0.208577 | 0.169299 | 0 | 0.291575 | 0.134943 | 0.074615 | 0.069736 | 0.056483 | 0.251324 |
| 0.533297 | 3.361827 | 0.675787 | 2.520825 | 0.085519 | 4.712959 | 0.542618 | 1.551159 | 37.78811 |
| 1.242205 | 86.73828 | 0.026026 | 2.364264 | 0.833271 | 1.699574 | 0.424098 | 0.755699 | 33.12836 |
| 2.749583 | 0.304503 | 0 | 3.976932 | 0.291251 | 0.793025 | 0.394055 | 1.023533 | 8.961582 |
| 0.379283 | 0.208481 | 0 | 0.599992 | 0.195498 | 0.287446 | 0.164175 | 0.55849 | 2.056059 |
| 0.521967 | 0.077833 | 0 | 0.488673 | 0.141802 | 0.700773 | 0.099235 | 0.192901 | 1.542296 |
| 11.87737 | 112.4984 | 10.00025 | 2.021311 | 0.491875 | 0.208929 | 0.220302 | 1.137516 | 2.456276 |
| 0.83722 | 37.64195 | 0.038321 | 1.450461 | 0.483322 | 0.996112 | 0.249774 | 1.264409 | 3.009927 |
| 1.125765 | 71.45581 | 0 | 2.894366 | 0.778987 | 0.349063 | 0.224289 | 1.877188 | 10.17133 |

| | | | | | | | | |
|---|---|---|---|---|---|---|---|---|
| 9.371944 | 279.0988 | 0 | 10.50051 | 2.075 | 2.343645 | 0.717094 | 14.21828 | 26.28278 |
| 3.396304 | 467.7609 | 0.031976 | 5.293121 | 0.186139 | 4.277596 | 0.416842 | 3.629435 | 20.04579 |
| 0.670934 | 13.37345 | 0.008317 | 3.52587 | 0.314703 | 1.956322 | 0.099387 | 1.119669 | 3.565555 |
| 0.264323 | 0.194433 | 0 | 0.868162 | 0.19074 | 1.48015 | 0.160179 | 0.356778 | 2.417324 |
| 2.135766 | 52.69052 | 0.105903 | 2.405102 | 0.783447 | 0.109107 | 0.287616 | 0.698866 | 8.706954 |
| 1.101379 | 113.1126 | 0.082508 | 2.415108 | 0.674701 | 2.122308 | 0.409744 | 11.79186 | 18.65463 |
| 0.410651 | 405.2389 | 0 | 0.779453 | 0.134188 | 0.073074 | 0.087647 | 0.334665 | 1.099906 |
| 2.335611 | 461.9872 | 0 | 1.934822 | 0.794278 | 0.683617 | 0.802589 | 1.159565 | 1.524405 |
| 2.821555 | 7.169545 | 0.251835 | 3.262103 | 0.651547 | 1.330535 | 0.592751 | 0.443169 | 8.27786 |
| 0.468809 | 194.1889 | 0.018352 | 0.944716 | 0.249274 | 0.733029 | 0.099683 | 2.761267 | 0.781369 |
| 0.276173 | 5.445105 | 0 | 0.783259 | 0.110717 | 0.217054 | 0.024794 | 0.180738 | 0.971744 |
| 0.208994 | 6.530493 | 0 | 0.394492 | 0.180977 | 0.208371 | 0.135094 | 0.562729 | 0.339067 |
| 0.937092 | 6.520317 | 0 | 0.504404 | 0.374261 | 0.194546 | 0.152386 | 0.092569 | 0.399018 |
| 0.560208 | 2.117697 | 0 | 2.789041 | 0.312057 | 1.028882 | 0.111177 | 0.656062 | 9.272655 |
| 1.863915 | 41.89888 | 0.011271 | 3.083038 | 1.071653 | 3.122773 | 0.514257 | 22.49231 | 51.56254 |
| 0.444836 | 109.672 | 0 | 3.425734 | 0.414195 | 4.452479 | 0.18551 | 15.04425 | 17.89452 |
| 0.69281 | 41.23351 | 0.022404 | 0.972396 | 0.173894 | 0.340907 | 0.170371 | 1.596766 | 3.355075 |
| 0.988499 | 1.603089 | 0 | 35.99525 | 0.632682 | 55.16635 | 0.569511 | 2.160127 | 32.77693 |
| 0.356976 | 0.745812 | 0 | 1.391516 | 0.237785 | 0.284877 | 0.410467 | 0.134781 | 0.179913 |
| 1.074526 | 739.635 | 0.133203 | 0.963544 | 0.409237 | 0.140751 | 0.120585 | 0.293005 | 2.966004 |
| 0.813347 | 199.0373 | 0.181486 | 1.251752 | 0.293463 | 0.13424 | 0.131437 | 1.197646 | 0.621713 |
| 1.611228 | 693.9296 | 0.024457 | 3.925061 | 1.02032 | 1.519584 | 0.318825 | 1.807632 | 8.833071 |
| 0.313031 | 64.76212 | 0 | 1.183725 | 0.786427 | 0.317095 | 0.262296 | 5.144263 | 4.684261 |
| 0.365854 | 1.211791 | 0 | 0.535923 | 0.528014 | 0.008416 | 0.08652 | 0.035038 | 0.097441 |
| 3.175278 | 18.4788 | 0 | 2.460976 | 0.585198 | 0.107722 | 0.203035 | 0.650319 | 15.55318 |
| 18.469 | 268.2042 | 0.310634 | 22.15675 | 3.01377 | 5.492524 | 2.212191 | 9.839551 | 63.64522 |
| 6.153466 | 123.4219 | 0 | 6.653621 | 2.399664 | 1.827148 | 0.995597 | 18.3623 | 34.23773 |
| 4.190725 | 253.0507 | 0 | 8.818718 | 3.184471 | 2.14835 | 1.013264 | 2.773744 | 34.33883 |
| 1.04543 | 349.7779 | 0.044118 | 0.434169 | 0.353124 | 0.2867 | 0.095853 | 1.281001 | 0.437205 |
| 2.980121 | 77.19432 | 0 | 19.05755 | 0.690837 | 24.91693 | 1.696781 | 1.940206 | 274.5703 |
| 0.795431 | 1.36877 | 0 | 1.202791 | 0.22797 | 0.191537 | 0.255259 | 0.310122 | 1.848078 |
| 0.673502 | 71.95041 | 0 | 6.74165 | 0.972022 | 1.693853 | 0.362792 | 8.815337 | 8.825433 |
| 4.034597 | 53.3384 | 0 | 7.578651 | 0.901449 | 2.72249 | 0.231879 | 5.336043 | 20.60914 |
| 4.364203 | 32.33462 | 0.013553 | 9.097238 | 2.550988 | 4.717476 | 1.295664 | 0.822846 | 28.05655 |
| 0.450783 | 6.431848 | 0 | 1.516121 | 0.260234 | 0.425143 | 0.145693 | 0.354012 | 0.511937 |
| 0.911834 | 21.59668 | 0 | 1.369096 | 0.849026 | 0.346762 | 0.261431 | 0.981734 | 1.027826 |
| 0.133481 | 9.07439 | 0 | 1.257166 | 0.192644 | 0.137333 | 0.088243 | 1.57239 | 2.477893 |
| 1.21875 | 4.453299 | 0 | 0.873747 | 0.079232 | 0.559182 | 0.177432 | 1.681424 | 1.942327 |
| 0.729179 | 7.858993 | 0 | 0.860235 | 1.002263 | 0.253796 | 0.252504 | 0.886236 | 4.398336 |
| 2.888855 | 159.7866 | 0 | 1.974555 | 0.342256 | 1.036953 | 0.325161 | 0.902965 | 1.616526 |
| 8.477228 | 66.90911 | 0.189631 | 6.364179 | 1.494833 | 1.743284 | 1.055763 | 1.188823 | 45.55412 |
| 2.674637 | 143.3235 | 0.022104 | 3.547382 | 0.343123 | 2.116105 | 0.528269 | 1.050234 | 11.68263 |
| 1.430199 | 56.2589 | 0 | 4.917257 | 0.837611 | 6.861551 | 0.60292 | 6.429772 | 18.96737 |
| 2.982606 | 38.74441 | 0 | 2.339792 | 0.556213 | 0.963993 | 0.27078 | 1.05273 | 9.734269 |
| 4.275578 | 450.7543 | 0.199235 | 7.796429 | 2.393921 | 1.768422 | 0.965639 | 17.59775 | 102.4449 |
| 2.441325 | 126.8673 | 0.024211 | 2.981348 | 0.657703 | 0.828888 | 0.341915 | 4.154023 | 11.05454 |
| 0.309066 | 23.82938 | 0.010692 | 0.863357 | 0.529041 | 0.494849 | 0.220687 | 1.749827 | 0.345343 |
| 1.085368 | 4.970943 | 0 | 1.288812 | 0.389051 | 0.127118 | 0.114637 | 0.557105 | 2.804206 |
| 0.059253 | 3.504769 | 0 | 0.474493 | 0.342066 | 0.149022 | 0.095753 | 0.620443 | 5.090012 |
| 0.684303 | 10.15802 | 0 | 2.962061 | 1.183353 | 5.314221 | 0.338723 | 48.6083 | 33.53861 |
| 3.990392 | 117.9571 | 0.232783 | 4.158792 | 1.490584 | 0.368964 | 0.733354 | 1.966286 | 10.69709 |
| 0.261244 | 29.66716 | 0.016896 | 0.324034 | 0.188518 | 0.048206 | 0.082598 | 0.066901 | 0.396902 |
| 3.351272 | 13.809 | 0.026947 | 7.588583 | 0.49674 | 1.708464 | 0.673296 | 0.569048 | 30.14663 |
| 1.032038 | 5.284586 | 0 | 4.065568 | 1.361348 | 3.778227 | 0.502124 | 1.481539 | 55.57287 |
| 1.890136 | 160.7974 | 0.093723 | 2.790694 | 0.818373 | 0.564499 | 0.763613 | 2.845055 | 9.150381 |
| 8.233959 | 364.9383 | 0.109853 | 18.905 | 5.435522 | 7.713393 | 4.14695 | 2.102292 | 82.45423 |
| 7.161345 | 766.7689 | 0 | 3.751518 | 0.761831 | 0.680381 | 0.255908 | 2.003639 | 12.1432 |
| 0.452345 | 4.220394 | 0 | 2.471477 | 0.788848 | 1.576109 | 0.23351 | 5.797301 | 13.61113 |

| | | | | | | | | |
|---|---|---|---|---|---|---|---|---|
| 0.611758 | 31.65619 | 0 | 3.563494 | 0.659524 | 2.599808 | 0.333503 | 2.286388 | 13.89179 |
| 0.053074 | 1.201261 | 0 | 0.517985 | 0.105323 | 0.012514 | 0.053605 | 0.364707 | 0.028978 |
| 1.173017 | 3.775932 | 0 | 2.721097 | 0.689716 | 0.181456 | 0.180533 | 0.584891 | 0.420194 |
| 3.327776 | 101.0133 | 0 | 4.000916 | 0.597053 | 4.871272 | 0.452236 | 14.93028 | 69.19641 |
| 3.831823 | 3.861267 | 0 | 2.90171 | 1.2984 | 0.785625 | 0.269227 | 2.878402 | 15.71836 |
| 2.524484 | 49.62842 | 0 | 4.102853 | 1.321242 | 2.210833 | 0.78453 | 3.703662 | 9.724159 |
| 0.703654 | 183.1382 | 0 | 2.303213 | 0.446837 | 1.526352 | 0.181936 | 0.442079 | 7.099811 |
| 5.276745 | 329.2894 | 0.508765 | 7.681868 | 1.561397 | 0.217233 | 1.522714 | 1.617028 | 63.429 |
| 0.600473 | 31.838 | 0 | 1.313195 | 0.348534 | 0.572475 | 0.105474 | 11.63544 | 9.878362 |
| 0.142074 | 1.759598 | 0 | 1.971086 | 0.246056 | 2.974651 | 0.055102 | 0.17852 | 8.414932 |
| 5.987898 | 644.6373 | 0.010215 | 3.227162 | 1.962277 | 1.075063 | 0.355098 | 0.404455 | 14.3971 |
| 1.558483 | 551.1491 | 0.035943 | 2.938637 | 0.383594 | 0.398794 | 0.097616 | 1.138531 | 2.717662 |
| 1.26473 | 156.9747 | 0.035498 | 2.723041 | 0.757673 | 1.541631 | 0.578434 | 1.686614 | 2.058567 |
| 7.192001 | 10.17741 | 0.084994 | 17.82255 | 0.401996 | 9.483985 | 1.765842 | 2.075263 | 73.54409 |
| 7.565375 | 95.88944 | 0.076385 | 10.17705 | 4.718226 | 2.130836 | 1.217029 | 12.76977 | 75.24848 |
| 15.13073 | 43.7514 | 0.026449 | 17.56607 | 3.515507 | 6.120558 | 0.804504 | 3.071879 | 135.7912 |
| 1.982381 | 18.26685 | 0 | 3.055989 | 1.001789 | 1.237172 | 0.114072 | 4.38866 | 3.057595 |
| 0.281142 | 186.9073 | 0.011303 | 0.958346 | 0.252226 | 0.802621 | 0.110511 | 0.238691 | 2.306667 |
| 0.233653 | 4.20049 | 0 | 1.97965 | 0.060217 | 4.249859 | 0.02697 | 9.469606 | 85.71042 |
| 1.355694 | 103.458 | 0.024594 | 3.773201 | 0.88285 | 0.99792 | 0.534341 | 3.960034 | 8.449083 |
| 1.105877 | 65.07118 | 0.05376 | 7.944103 | 0.792805 | 5.392122 | 0.221927 | 0.312197 | 7.671801 |
| 1.849703 | 15.27849 | 0 | 8.924761 | 0.629896 | 7.664022 | 0.436612 | 2.285027 | 23.60268 |
| 0.626104 | 333.5271 | 0 | 2.578504 | 0.335628 | 0.016871 | 0.375804 | 0.070242 | 6.836943 |
| 1.275481 | 0.249675 | 0 | 1.509654 | 0.265343 | 0.021674 | 0.417805 | 0.225602 | 0.301147 |
| 4.252417 | 27.57334 | 0.046089 | 6.001112 | 1.173789 | 10.51947 | 1.189125 | 0.486637 | 8.289063 |
| 0.154429 | 0.268867 | 0 | 0.365643 | 0.120011 | 0.016805 | 0.028795 | 0.09329 | 0.14269 |
| 7.825328 | 45.90559 | 0 | 2.897512 | 0.33217 | 0.651198 | 1.144407 | 1.04278 | 4.369226 |
| 1.420079 | 156.6322 | 0 | 6.812249 | 0.67738 | 3.29152 | 0.272269 | 25.61245 | 7.096435 |
| 0.276681 | 0.183286 | 0 | 0.405298 | 0.097394 | 0.019093 | 0.087242 | 0.344477 | 0.162119 |
| 0.472303 | 3.4901 | 0.113932 | 2.280784 | 0.294765 | 1.324278 | 0.247537 | 0.15037 | 0.641198 |
| 16.33588 | 651.5365 | 0.090599 | 13.8084 | 3.008107 | 2.757128 | 4.352398 | 9.778546 | 357.2577 |
| 0.230096 | 2.049823 | 0 | 0.373122 | 0.132833 | 0.086803 | 0.029747 | 0.180701 | 0.643228 |
| 6.805758 | 855.7534 | 3.767295 | 3.349615 | 0.825328 | 2.068433 | 0.375742 | 0.34546 | 7.534704 |
| 0.27307 | 4.618542 | 0 | 0.326143 | 0.161224 | 0.040972 | 0.11032 | 0.243692 | 0.935224 |
| 2.385911 | 92.21237 | 0 | 9.197419 | 0.647667 | 5.586717 | 0.471377 | 4.610886 | 46.5047 |
| 0.561927 | 50.20777 | 0.01032 | 3.822778 | 0.180221 | 2.669455 | 0.123318 | 4.630891 | 8.984522 |
| 1.719018 | 63.31675 | 0 | 2.332015 | 0.544068 | 1.251486 | 0.073103 | 0.473682 | 7.212162 |
| 0.987863 | 19.67576 | 0.010065 | 2.255357 | 1.113232 | 0.867862 | 0.907532 | 1.381555 | 13.97915 |
| 0.861713 | 156.0063 | 0 | 1.481356 | 0.252336 | 1.183717 | 0.272451 | 0.760093 | 3.286598 |
| 1.433707 | 511.2124 | 0 | 1.989182 | 0.916724 | 1.865639 | 0.205292 | 1.49649 | 31.4702 |
| 1.634553 | 196.0666 | 0.281914 | 3.236756 | 1.059861 | 0.938355 | 0.689069 | 1.116227 | 5.173636 |
| 1.566858 | 331.8728 | 0.027102 | 2.872362 | 0.44701 | 2.594634 | 0.176654 | 0.429244 | 15.63758 |
| 4.652036 | 85.97102 | 0.012744 | 3.177303 | 0.729512 | 2.658319 | 0.318428 | 2.422127 | 30.96614 |
| 0.650297 | 38.99276 | 0 | 3.154542 | 1.389749 | 0.678179 | 0.323347 | 79.45262 | 3.482286 |
| 1.009034 | 0.388357 | 0 | 1.74925 | 0.154773 | 0.165503 | 0.102405 | 0.114844 | 7.611801 |
| 3.607893 | 8.564135 | 0 | 5.091754 | 0.66238 | 2.140204 | 0.844678 | 0.951135 | 14.17199 |
| 0.74198 | 56.2839 | 0 | 2.494323 | 0.530688 | 3.70944 | 0.328939 | 5.723844 | 6.094644 |
| 0.44438 | 99.33038 | 0.011397 | 1.115879 | 0.751922 | 0.708143 | 0.309533 | 1.895346 | 10.90989 |
| 0.816097 | 4.554198 | 0 | 2.591313 | 0.366862 | 0.16403 | 0.216198 | 4.465312 | 1.811545 |
| 0.432637 | 0.603579 | 0 | 0.521805 | 0.163776 | 0.180603 | 0.034384 | 0 | 0.092937 |
| 11.33596 | 1082.224 | 0 | 8.368496 | 1.363371 | 4.09829 | 1.144928 | 12.98273 | 109.8607 |
| 1.319964 | 65.00325 | 0 | 0.913808 | 0.798511 | 0.149088 | 0.447048 | 14.2766 | 3.193472 |
| 3.068485 | 54.34348 | 0 | 4.980226 | 0.695914 | 2.521876 | 0.472253 | 2.75402 | 3.47838 |
| 3.877569 | 126.4356 | 0 | 7.73142 | 1.464607 | 3.456151 | 0.394797 | 5.682011 | 116.4929 |
| 0.862124 | 220.2668 | 0 | 3.205328 | 0.483874 | 2.019815 | 0.298541 | 0.134335 | 4.393316 |
| 1.570687 | 117.2526 | 0 | 3.716639 | 0.932574 | 1.190705 | 0.2731 | 1.795608 | 18.36744 |
| 3.146121 | 888.3827 | 0 | 4.040379 | 1.062692 | 1.906568 | 0.346153 | 8.516158 | 19.53124 |
| 1.918984 | 6.254406 | 0 | 2.75034 | 1.08264 | 0.21389 | 0.253724 | 8.425678 | 6.896091 |

| | | | | | | | | |
|---|---|---|---|---|---|---|---|---|
| 3.37591 | 327.1847 | 0 | 5.12031 | 0.853259 | 4.420281 | 0.318466 | 6.493415 | 61.7899 |
| 0.29846 | 74.6201 | 0 | 8.71496 | 0.022671 | 7.451923 | 0.114231 | 0.061681 | 24.73509 |
| 1.432279 | 522.95 | 0 | 4.694455 | 0.831888 | 1.219027 | 0.282263 | 3.566467 | 12.09257 |
| 0.888144 | 1.382001 | 0 | 1.791927 | 0.380535 | 0.117792 | 0.40366 | 1.525747 | 0.909223 |

es

| NFATC1 | CD22 | SAA1 | SAA2 | FPR1 | FPR2 | ANGPTL7 | CALCA | CCK |
|---|---|---|---|---|---|---|---|---|
| 0.697248 | 0.615942 | 5.316338 | 0.130445 | 0.533595 | 0.06004 | 0.435223 | 0.024091 | 0.057371 |
| 2.827904 | 21.73885 | 0.027981 | 0.028835 | 0.792174 | 0.040496 | 0.946879 | 0.010059 | 0.010266 |
| 0.995673 | 0.276735 | 6.38288 | 0.158584 | 0.834642 | 0.14525 | 1.873068 | 0.014431 | 0.662808 |
| 0.740643 | 0.6783 | 1.744479 | 0.540626 | 1.616096 | 0.258751 | 1.212051 | 0 | 0.160015 |
| 1.076606 | 1.601834 | 4.006956 | 0.011567 | 0.575953 | 0.062657 | 0.437374 | 0.02421 | 0.086481 |
| 0.708588 | 0.99231 | 0.388657 | 0 | 1.146195 | 0.450895 | 0.405937 | 0 | 0.007922 |
| 1.004993 | 0.332286 | 1.143261 | 0.031418 | 0.445052 | 0.042548 | 1.469382 | 0.057539 | 0.067115 |
| 0.906797 | 1.409131 | 1.499832 | 0.039632 | 0.277866 | 0.015903 | 0.815025 | 0 | 0.007055 |
| 1.22886 | 2.697069 | 9.267289 | 1.075251 | 6.316494 | 0.881598 | 1.103666 | 0.177209 | 0.063302 |
| 0.96222 | 1.456549 | 0.291708 | 0 | 0.67962 | 0.054281 | 3.469992 | 0.010487 | 0.792018 |
| 1.830504 | 0.679497 | 42.3819 | 1.678628 | 4.749401 | 0.629087 | 3.487832 | 0.066291 | 0.462334 |
| 0.951543 | 0.227098 | 4.506534 | 0.521913 | 2.437817 | 0.127784 | 0.49294 | 0.009258 | 0.018897 |
| 1.967256 | 10.75323 | 0.793919 | 0.031712 | 0.892982 | 0.095435 | 1.457877 | 0.013275 | 0.013549 |
| 0.765595 | 2.604482 | 0.743349 | 0.031919 | 1.023036 | 0.211327 | 0.42349 | 0.011135 | 0.045457 |
| 0.877238 | 1.878858 | 0.147757 | 0.006345 | 0.452094 | 0.057281 | 0.707096 | 0 | 0 |
| 1.56251 | 5.234443 | 0.868627 | 0.009625 | 0.644551 | 0.05214 | 0.459742 | 0.020146 | 0 |
| 0.846101 | 0.840402 | 0.029938 | 0 | 1.271397 | 0.167129 | 0.429811 | 0.021525 | 0 |
| 1.817614 | 2.363763 | 12.38697 | 0.437238 | 5.337677 | 0.898628 | 3.289693 | 0.078123 | 1.355485 |
| 0.627371 | 2.388397 | 4.62132 | 0.310197 | 2.067817 | 0.197687 | 0.526558 | 0.019096 | 0.136427 |
| 1.103056 | 4.100412 | 2.195832 | 0.11143 | 0.345864 | 0.05675 | 1.714407 | 0 | 0.091553 |
| 1.325437 | 4.92083 | 1.77951 | 0.008043 | 2.237295 | 0.338879 | 0.288131 | 0.016835 | 0.025773 |
| 1.296545 | 3.362311 | 41.15789 | 1.809771 | 0.643808 | 0.030091 | 0.815866 | 0.010464 | 0 |
| 1.120058 | 1.099387 | 1.536656 | 0.042229 | 2.891272 | 0.343131 | 5.746395 | 0.099435 | 0.315733 |
| 1.21025 | 1.799171 | 2.869163 | 0.918608 | 1.667652 | 0.161263 | 1.647272 | 0 | 0.010221 |
| 2.214525 | 0.30339 | 0.463782 | 0.042484 | 0.446797 | 0.063927 | 6.552637 | 0.011115 | 0.011344 |
| 1.618243 | 1.3711 | 12.89356 | 0.23311 | 2.508288 | 0.40689 | 1.623748 | 0.024395 | 0.041498 |
| 0.797455 | 0.22579 | 1.751725 | 0.045414 | 3.957378 | 0.656026 | 0.564887 | 0 | 0.41231 |
| 0.659436 | 0.716418 | 1.113488 | 0.122399 | 0.413759 | 0.096693 | 1.14946 | 0.016012 | 0.130735 |
| 2.454585 | 1.927428 | 0.46027 | 0.022587 | 1.076216 | 0.163138 | 0.899034 | 0.023638 | 0.120626 |
| 0.669613 | 0.443123 | 8.021758 | 0.058012 | 1.332267 | 0.410274 | 1.067945 | 0.007589 | 0.030981 |
| 0.729671 | 3.716717 | 11.69391 | 0.299776 | 0.514726 | 0.140336 | 0.80873 | 0.006972 | 0.014231 |
| 0.83534 | 0.674252 | 1.533016 | 0.017266 | 1.104319 | 0.114314 | 1.159717 | 0.090346 | 0.027663 |
| 1.701736 | 8.480708 | 3.956408 | 0.988416 | 1.785542 | 0.154927 | 0.481533 | 0 | 0 |
| 1.089103 | 3.66366 | 0.05613 | 0.019281 | 0.703512 | 0.034815 | 0.642743 | 0.030267 | 0.030891 |
| 0.568681 | 0.92462 | 1.13965 | 0.174918 | 3.117825 | 0.506355 | 0.132616 | 0 | 0.035587 |
| 0.67722 | 1.406842 | 0.109966 | 0 | 0.562122 | 0.083366 | 0.476125 | 0 | 0 |
| 2.229488 | 0.136633 | 0.268299 | 0 | 0.632995 | 0.027736 | 4.952381 | 0.212193 | 1.132067 |
| 1.429478 | 1.897561 | 13.13365 | 0.385703 | 2.629002 | 0.34471 | 1.442186 | 0.048927 | 0.237196 |
| 0.7121 | 1.238466 | 0.052756 | 0.072489 | 0.972395 | 0.081807 | 0.577062 | 0 | 0.009678 |
| 1.414156 | 0.607275 | 4.583586 | 0.119963 | 1.815209 | 0.288819 | 6.69983 | 0.125544 | 0.512533 |
| 0.894616 | 0.639094 | 15.34189 | 1.29328 | 5.847611 | 1.686565 | 4.50423 | 0.214296 | 0.564055 |
| 1.0058 | 1.24912 | 1.751948 | 0.062526 | 1.013209 | 0.122311 | 1.197713 | 0 | 0.066785 |
| 0.709058 | 0.37925 | 2.429537 | 0.236839 | 2.57552 | 0.237585 | 0.067336 | 0.094422 | 0.012046 |
| 0.183331 | 0.179947 | 2.786063 | 0.401959 | 0.221843 | 0.011521 | 0 | 0.040063 | 0.051111 |
| 7.986773 | 0.246743 | 55.33883 | 8.16922 | 2.125791 | 0.117351 | 0 | 0 | 0.437321 |
| 0.4916 | 0.265938 | 57.45553 | 3.44993 | 4.153075 | 1.090725 | 0.007545 | 0.007935 | 0 |
| 0.954147 | 0.239116 | 0.3008 | 0.33065 | 1.54354 | 0.559731 | 3.022915 | 0.194643 | 0.066219 |
| 0.168219 | 0.036692 | 0.664667 | 0.088791 | 0.816741 | 0.160329 | 0 | 1.725698 | 0.013548 |
| 1.021549 | 1.328389 | 13.34645 | 1.062669 | 2.841233 | 0.164472 | 0.030213 | 0.222422 | 0.06486 |
| 0.451379 | 1.048151 | 0.323182 | 0.012335 | 0.20121 | 0.022273 | 0 | 22.68121 | 0.013175 |
| 0.075997 | 0.013011 | 1.131281 | 0.899556 | 0.092674 | 0.012994 | 0 | 0.451875 | 0.007687 |
| 2.130071 | 0.98952 | 23.00036 | 8.077442 | 19.61026 | 3.387283 | 0.065883 | 0 | 2.451547 |
| 0.719976 | 0.090625 | 91.88741 | 9.227796 | 0.3521 | 0.118854 | 0 | 0.015896 | 1.476411 |
| 1.810422 | 0.968332 | 28.2501 | 5.327249 | 5.488257 | 3.1371 | 3.725073 | 0.120542 | 0.246057 |
| 0.713417 | 1.594247 | 1.905233 | 0.355939 | 2.89809 | 0.331722 | 0.022851 | 0.096129 | 0 |
| 0.817969 | 0.138716 | 21.47307 | 0.558014 | 5.703877 | 0.671728 | 0.121466 | 1.78842 | 0.055877 |

| | | | | | | | | |
|---|---|---|---|---|---|---|---|---|
| 1.943976 | 0.165291 | 0.350199 | 0.048119 | 4.21373 | 0.810948 | 0.119706 | 0.050358 | 0.102792 |
| 0.541483 | 0.201042 | 60.22248 | 3.604427 | 6.847342 | 1.338613 | 0 | 5.678236 | 0.225261 |
| 0.67425 | 0.89838 | 8.100776 | 0.645986 | 0.405279 | 0.014954 | 0.012362 | 0.039002 | 0.013269 |
| 0.721862 | 0.425905 | 39.07119 | 6.462156 | 3.525414 | 0.53985 | 0.010753 | 0 | 0 |
| 0.162113 | 0.051146 | 3.156956 | 0.298617 | 0.404792 | 0.094597 | 0.015639 | 0.46054 | 0 |
| 2.279018 | 0.30504 | 4.657682 | 0.174542 | 15.18456 | 4.394814 | 0.144737 | 0 | 0.155358 |
| 0.312079 | 0.067792 | 24.07551 | 2.916449 | 1.359221 | 0.326 | 0.041459 | 0.370616 | 0 |
| 0.166697 | 0.039883 | 3.754325 | 0.48362 | 0.145683 | 0.005107 | 0.008443 | 0.266379 | 0 |
| 1.96383 | 1.364159 | 1.024636 | 0.175987 | 1.879162 | 0.20523 | 0.087561 | 0.011511 | 0.152728 |
| 0.682381 | 0.346704 | 55.47974 | 4.150331 | 3.52108 | 0.395981 | 0.020893 | 3.1422 | 0.209315 |
| 0.571722 | 0.054812 | 15.76038 | 2.338792 | 0.78085 | 0.293271 | 0.010775 | 10.49294 | 0.277564 |
| 0.574591 | 0.307629 | 3.233488 | 2.883197 | 3.205693 | 2.731079 | 2.045945 | 0.197858 | 0 |
| 0.243515 | 0.179266 | 1.580718 | 0.188868 | 2.634883 | 0.426292 | 0.023492 | 0.395309 | 0.025216 |
| 0.390935 | 0.153295 | 7.358496 | 1.127879 | 0.274697 | 0.019258 | 0 | 0.008371 | 0.059807 |
| 0.522349 | 0.469983 | 0.825562 | 0.047265 | 2.292652 | 0.113794 | 0.070549 | 0.469907 | 0 |
| 1.305028 | 0.503031 | 8.431747 | 0.729802 | 5.32567 | 1.249151 | 0.022694 | 0.190939 | 0.02436 |
| 0.278818 | 0.092155 | 4.249281 | 0.532898 | 0.377938 | 0.013946 | 0.046111 | 0.4607 | 0.09899 |
| 1.121955 | 1.059692 | 6.743696 | 1.788582 | 4.902605 | 1.776928 | 0 | 0 | 0.207151 |
| 0.600794 | 0.376486 | 3.678497 | 0.919905 | 4.035569 | 0.827483 | 0 | 0 | 0 |
| 1.293599 | 0.084858 | 0.22773 | 0.026076 | 0.111933 | 0.015695 | 0 | 0.027289 | 0.027852 |
| 0.81451 | 0.50413 | 15.31251 | 1.858963 | 10.86339 | 1.792775 | 0.021021 | 0.486362 | 0 |
| 0.742447 | 0.625898 | 15.75604 | 3.120651 | 3.955918 | 1.247305 | 0.02426 | 0 | 4.557088 |
| 1.850075 | 0.196181 | 13.36457 | 2.211971 | 16.69705 | 2.135212 | 0.04153 | 0 | 0.044578 |
| 0.147659 | 0.091371 | 4.464492 | 0.792943 | 0.119694 | 0.010489 | 0 | 0.182381 | 0 |
| 0.369436 | 0.129506 | 23.30328 | 3.277191 | 2.721286 | 0.582054 | 0 | 0.412308 | 0 |
| 0.224514 | 0.161905 | 2.878547 | 0.279853 | 0.35238 | 0.089835 | 0.018565 | 0.683369 | 0 |
| 0.641103 | 0.073743 | 21.72945 | 2.406528 | 9.804991 | 1.767624 | 0.040588 | 3.414896 | 0.043567 |
| 2.054032 | 0.076347 | 1.365928 | 0.574785 | 18.63078 | 31.63046 | 1.202282 | 0 | 0.012529 |
| 0.648361 | 0.322982 | 22.47837 | 2.620175 | 2.215389 | 0.191159 | 0.039504 | 0.020773 | 0 |
| 0.280604 | 0.250057 | 7.768337 | 0.56377 | 0.154883 | 0.004524 | 0.01496 | 0.157333 | 0.008029 |
| 0.477333 | 0.029283 | 0.51866 | 0.032394 | 0.472781 | 0.009749 | 0 | 0.305107 | 0.380599 |
| 1.035854 | 0.125292 | 2.365282 | 0.203125 | 5.088001 | 2.164713 | 0 | 0.200071 | 0 |
| 1.841912 | 0.663143 | 32.55459 | 2.877726 | 4.941169 | 0.538471 | 0 | 0.015604 | 0 |
| 0.871489 | 0.081004 | 1.591281 | 0.179221 | 8.047113 | 3.392558 | 0.008917 | 0 | 0.086142 |
| 0.141769 | 0.048703 | 2.436689 | 0.481052 | 0.768166 | 0.185306 | 0.201049 | 1.077344 | 0 |
| 0.351625 | 0.215149 | 4.113889 | 0.325401 | 1.388832 | 0.139897 | 0.009251 | 0.768645 | 0.00993 |
| 0.352298 | 1.146148 | 4.782194 | 0.231372 | 0.397274 | 0.013926 | 0.138141 | 0.266349 | 0.024713 |
| 0.928714 | 0.20299 | 4.743911 | 0.350868 | 1.077724 | 0.276887 | 0.007759 | 0.01632 | 0.008328 |
| 4.588148 | 0.267066 | 162.2728 | 8.233215 | 4.513317 | 0.496858 | 0.021617 | 0 | 0.139218 |
| 0.286603 | 0.173752 | 1.532103 | 0.09153 | 0.766154 | 0.055091 | 0.06831 | 0 | 0 |
| 0.574402 | 0.143949 | 0.840226 | 0.258769 | 0.649387 | 0.047923 | 0.049519 | 0 | 0.042522 |
| 0.218447 | 0.00946 | 5.026445 | 0.549386 | 0.291979 | 0.031492 | 0 | 1.697456 | 0 |
| 0.262801 | 0.13172 | 2.960483 | 0.371873 | 0.820956 | 0.091358 | 0.022656 | 0.198558 | 0.008106 |
| 0.124683 | 0.118782 | 0.6955 | 0.179184 | 1.12811 | 0.233674 | 0 | 1.93771 | 0 |
| 0.475321 | 0.080363 | 0.727872 | 0.111125 | 0.739376 | 0.061312 | 0.009215 | 0.765611 | 0.019782 |
| 0.146926 | 0.06656 | 0.321526 | 0.027612 | 0.237054 | 0.016619 | 0.009159 | 0.028897 | 0.039323 |
| 2.780918 | 0.206823 | 5.446941 | 0.562296 | 7.074663 | 2.526638 | 0 | 0.050729 | 0.031065 |
| 0.390205 | 0.173435 | 2.996281 | 0.259812 | 1.441272 | 0.324787 | 0.029831 | 0 | 0 |
| 0.550525 | 0.323085 | 0.410708 | 0.337031 | 1.076644 | 0.084917 | 0.046796 | 0.902284 | 0.351613 |
| 0.224827 | 0.048273 | 1.480559 | 0.114433 | 0.272896 | 0.007653 | 0 | 0.039919 | 0 |
| 0.38077 | 0.100993 | 2.00784 | 0.393309 | 0.856393 | 0.130373 | 0.022688 | 2.82756 | 0.006088 |
| 1.056432 | 0.359521 | 22.69897 | 2.752624 | 2.201443 | 0.485066 | 0.010415 | 0.010953 | 0.089432 |
| 0.59567 | 0.252862 | 4.432347 | 0.061965 | 3.647859 | 0.367633 | 0 | 0.009264 | 0.07564 |
| 0.189613 | 0.083157 | 2.391054 | 0.131417 | 0.465398 | 0.029662 | 0.032693 | 0.017191 | 0 |
| 0.268814 | 0.085926 | 9.098044 | 0.943346 | 0.24111 | 0.017337 | 0 | 0.075361 | 0.046149 |
| 0.456088 | 0.190769 | 5.897757 | 0.738627 | 4.16711 | 0.673211 | 0.042 | 0.331281 | 0 |
| 1.609395 | 2.149117 | 65.21712 | 5.486406 | 0.91587 | 0 | 0 | 0.212654 | 0.10852 |
| 5.471251 | 0.212171 | 4.782911 | 0.958408 | 1.863921 | 0.553313 | 0 | 0 | 0.167132 |

| | | | | | | | | |
|---|---|---|---|---|---|---|---|---|
| 0.47842 | 1.408771 | 1.181457 | 0.101461 | 0.461663 | 0.036641 | 0.323079 | 3.270382 | 0 |
| 1.033899 | 0.179086 | 4.376936 | 0.442214 | 17.72203 | 4.769677 | 0.035203 | 0.259161 | 20.74479 |
| 0.25092 | 0.705281 | 2.771505 | 0.208985 | 0.049838 | 0 | 0 | 0.048602 | 0 |
| 0.58319 | 0.03481 | 0.420379 | 0.016045 | 0.1102 | 0 | 0.143695 | 0.35262 | 0.15424 |
| 0.198055 | 0.029583 | 0.034024 | 0.140252 | 0.421432 | 0.035174 | 2.163223 | 0.318017 | 0 |
| 0.422475 | 0.240592 | 5.385802 | 0.535551 | 4.472414 | 0.732599 | 0.032298 | 2.683443 | 0.034668 |
| 1.564922 | 0.517967 | 9.274978 | 0.913832 | 6.721619 | 1.025727 | 0.614419 | 8.839732 | 0 |
| 0.083618 | 0.138501 | 0.154871 | 0.01064 | 2.557698 | 0.480309 | 0 | 0.04454 | 0.272751 |
| 0.055876 | 0.045389 | 119.4474 | 8.174931 | 0.175122 | 0.009444 | 0 | 2.348178 | 0 |
| 0.669526 | 0.608596 | 7.59123 | 1.967609 | 1.506061 | 0.142685 | 0.02359 | 0.074427 | 0.025321 |
| 0.260414 | 0.047926 | 3.061113 | 0.441818 | 0.751033 | 0.111689 | 0 | 1.646047 | 0 |
| 0.210731 | 0.037381 | 2.67849 | 0.134395 | 0.186385 | 0.018667 | 0.030862 | 0 | 0.09938 |
| 1.628512 | 1.711652 | 16.23832 | 2.390592 | 3.550927 | 0.393979 | 0.066079 | 0.009928 | 0.060795 |
| 2.321419 | 0.147984 | 19.15432 | 3.038009 | 51.04891 | 14.7279 | 0.046991 | 0.008237 | 1.437514 |
| 0.770567 | 0.090041 | 6.886738 | 0.647448 | 21.16492 | 4.826203 | 0 | 0 | 0.159587 |
| 0.334318 | 0.23541 | 1.809134 | 0.236746 | 0.228658 | 0.04453 | 0.029448 | 0.046455 | 0.126435 |
| 0.716482 | 0.125086 | 1.712004 | 0.426656 | 2.643245 | 0.49972 | 0 | 0 | 0.714362 |
| 1.74829 | 0.484007 | 2.833986 | 0.88224 | 16.84608 | 12.46966 | 4.268429 | 0.318374 | 0.097482 |
| 7.163986 | 23.12822 | 2.23471 | 0.118932 | 1.169566 | 0.234275 | 0.247453 | 0.577063 | 0.080838 |
| 0.274811 | 0.070028 | 0.532475 | 0.021519 | 0.33254 | 0.03238 | 0 | 0.551742 | 0 |
| 0.622167 | 0.293688 | 2.589663 | 0.193387 | 4.366492 | 0.640188 | 0.076974 | 0.12143 | 0.020656 |
| 1.155007 | 0.125039 | 1.510035 | 0.034581 | 4.423576 | 0.353838 | 0.172055 | 0 | 0.073872 |
| 0.963438 | 0.844717 | 11.16357 | 1.190115 | 5.71416 | 0.848973 | 0 | 0.507419 | 0 |
| 0.53873 | 0.508632 | 20.30312 | 1.426349 | 0.050671 | 0 | 0.009789 | 0.030884 | 0 |
| 0.089635 | 0.141397 | 0.025297 | 0 | 0.268578 | 0.020922 | 0.017295 | 0.081848 | 0.037127 |
| 3.654595 | 0.245593 | 2.696271 | 0.113202 | 2.217616 | 0.17963 | 0.040962 | 0 | 0.065952 |
| 0.53189 | 0.380973 | 1.874404 | 0.175603 | 1.495027 | 0.264236 | 0.043685 | 1.408926 | 0 |
| 0.69322 | 0.064292 | 1.639116 | 0.259302 | 0.400708 | 0.031214 | 0 | 0.085286 | 0.17409 |
| 1.603348 | 0.819416 | 79.22725 | 9.640143 | 9.868074 | 2.343164 | 0.537251 | 0.996224 | 3.224829 |
| 0.48543 | 0.156085 | 0.198417 | 0.068158 | 2.184573 | 0.656384 | 0 | 0.142659 | 0 |
| 0.554667 | 0.158993 | 5.411119 | 0.89941 | 2.831259 | 0.13233 | 0.039777 | 0.167334 | 0.021348 |
| 0.148296 | 0.095936 | 4.474492 | 0.530645 | 0.290622 | 0.044054 | 0.054624 | 0.229792 | 0.019544 |
| 2.216591 | 0.931951 | 44.20021 | 7.076157 | 7.016447 | 0.541552 | 0.45699 | 2.893484 | 0.020021 |
| 0.816102 | 0.065145 | 1.282062 | 0.040037 | 1.529564 | 0.048195 | 0 | 0.335194 | 0 |
| 2.840442 | 0.117332 | 1.619372 | 0.11589 | 1.432714 | 0.223211 | 0 | 0 | 0 |
| 0.51369 | 0.156286 | 29.97352 | 1.157749 | 0.377701 | 0.060393 | 0.00768 | 0.355406 | 0 |
| 0.164967 | 0.013879 | 0.78218 | 0.172728 | 0.04943 | 0.034655 | 0 | 0.100424 | 0 |
| 1.349324 | 0.026054 | 3.618392 | 0.108083 | 0.29384 | 0.054212 | 0 | 0.018852 | 0.057722 |
| 0.621474 | 0.143275 | 0.760265 | 0.108066 | 1.785954 | 0.184292 | 0.017922 | 0.047122 | 0 |
| 0.23541 | 0.100755 | 2.01172 | 0.167189 | 0.354054 | 0.053669 | 0.022182 | 0.828179 | 0 |
| 2.162959 | 3.424822 | 4.114875 | 0.3866 | 2.219606 | 0.18179 | 0.132241 | 0 | 0.051616 |
| 1.079233 | 0.350389 | 228.3349 | 9.217117 | 2.128211 | 0.189897 | 0.381225 | 0.978743 | 0.012035 |
| 0.81438 | 0.155429 | 6.971845 | 0.962057 | 2.882021 | 0.246406 | 0 | 1.542354 | 0 |
| 2.755184 | 0.141506 | 6.519108 | 0.456574 | 7.260927 | 1.845142 | 0.021635 | 0 | 0 |
| 1.859743 | 0.180968 | 3.040639 | 0.239363 | 2.223116 | 0.19646 | 0 | 0.022773 | 0.092969 |
| 1.937033 | 0.075548 | 4.865866 | 0.644217 | 1.509738 | 0.073358 | 0 | 0.036443 | 0.130179 |
| 0.559295 | 0.63855 | 0.218342 | 0.129551 | 5.104487 | 0.915185 | 0.020355 | 0.064221 | 0.014566 |
| 0.219401 | 0.082828 | 3.267533 | 1.672199 | 0.530981 | 0.05515 | 0 | 0.119863 | 0.024467 |
| 0.286652 | 0.738573 | 4.633434 | 0.344855 | 5.397486 | 0.367231 | 0 | 0.333137 | 0 |
| 1.705597 | 0.04603 | 4.611721 | 1.018401 | 0.376442 | 0.085134 | 0 | 0 | 0.045323 |
| 1.194185 | 1.424255 | 1.871568 | 0.135825 | 6.988732 | 1.793093 | 0.666777 | 0.094763 | 0.009672 |
| 1.345805 | 0.491462 | 47.22982 | 2.049572 | 11.35404 | 3.830718 | 0.021468 | 0.022578 | 0.460876 |
| 0.597581 | 0.324698 | 48.40376 | 5.214119 | 3.03402 | 0.348704 | 0 | 0 | 0.03094 |
| 2.699908 | 0.063349 | 7.172126 | 0.744587 | 2.932996 | 1.061087 | 0.010896 | 0 | 0.31578 |
| 4.574571 | 0.494998 | 2.025275 | 0.201979 | 9.460091 | 1.472342 | 0.178654 | 0.023486 | 1.366321 |
| 2.564665 | 0.523475 | 827.388 | 57.67734 | 5.700767 | 0.546061 | 0.019208 | 0.020201 | 4.35032 |
| 0.453693 | 0.292241 | 10.41966 | 0.750493 | 1.96598 | 0.312726 | 0.019149 | 0.644437 | 0 |
| 0.460946 | 0 | 3.41492 | 0 | 0.805679 | 0 | 0.11673 | 3.314622 | 0 |

| | | | | | | | | |
|---|---|---|---|---|---|---|---|---|
| 0.467952 | 0.360146 | 1.260663 | 0.4114 | 0.669211 | 0.221553 | 0.021546 | 0 | 0.208145 |
| 2.295205 | 0.749741 | 2.886856 | 0.210354 | 11.17096 | 1.356538 | 0.089709 | 0.062897 | 0.032097 |
| 0.34185 | 0.12002 | 4.582043 | 0.471125 | 1.195024 | 0.244898 | 0.010655 | 1.299839 | 0.06862 |
| 0.848559 | 1.485712 | 0.770244 | 0.638123 | 3.460753 | 2.182705 | 3.469195 | 0 | 0 |
| 0.309503 | 0.103929 | 16.73455 | 0.972825 | 0.702318 | 0.013308 | 0.022001 | 1.295742 | 0 |
| 1.712282 | 0.137095 | 0.613193 | 0.273831 | 4.340105 | 0.735335 | 0 | 0 | 0.067495 |
| 2.90409 | 0.508009 | 429.6513 | 5.47931 | 2.734004 | 0.408721 | 0 | 0 | 0 |
| 1.144649 | 1.359626 | 60.10647 | 11.16798 | 63.57806 | 13.86396 | 0.044811 | 0.047127 | 0 |
| 0.169123 | 0.043942 | 4.304239 | 0.891184 | 1.582364 | 0.243817 | 0.020155 | 2.925111 | 0 |
| 0.312008 | 0.182166 | 13.00823 | 2.562802 | 1.899332 | 0.276864 | 0.021797 | 0.06877 | 0 |
| 0.425895 | 0.26773 | 3.233233 | 0.194851 | 5.035239 | 0.774044 | 0.038779 | 0.183525 | 0.020812 |
| 0.532482 | 0.464582 | 2.571486 | 0.642425 | 0.896244 | 0 | 0.159817 | 1.932899 | 0.085772 |
| 0.465954 | 0.28013 | 1.58793 | 0.260878 | 2.674052 | 0.428237 | 0.157331 | 0.355746 | 0 |
| 0.957836 | 0.154124 | 4.963411 | 0.660683 | 2.781156 | 0.718353 | 0.007069 | 0.899589 | 0 |
| 0.822487 | 0.50709 | 7.738611 | 0.167452 | 4.366737 | 0.289766 | 0.270772 | 0.153337 | 0.089428 |
| 0.865029 | 0.266981 | 1.464814 | 0.08751 | 2.36186 | 0.263357 | 0.076195 | 0.028619 | 0 |
| 0.529182 | 0.127308 | 5.042766 | 0.999915 | 0.241816 | 0.016953 | 0 | 0.014739 | 0.030085 |
| 0.926681 | 0.393838 | 10.02194 | 0.3501 | 1.352556 | 0.140482 | 0.019354 | 0 | 11.55071 |
| 0.819687 | 0.656978 | 0.344955 | 0.671478 | 2.933251 | 2.638909 | 2.515492 | 0.041336 | 0.970342 |
| 0.163987 | 0.006525 | 2.784408 | 0.559448 | 0.278882 | 0.021724 | 0 | 0.169977 | 0 |
| 1.860639 | 0.418436 | 62.0337 | 5.677654 | 2.460474 | 0.663877 | 0.2519 | 0.009461 | 0.086909 |
| 0.888186 | 0.330097 | 20.3 | 3.497174 | 1.712208 | 0.367727 | 0.006988 | 0.198427 | 0 |
| 0.538937 | 0.450572 | 36.43599 | 8.270603 | 1.344357 | 0.305318 | 0 | 0.969402 | 0 |
| 0.80053 | 0.663914 | 12.6422 | 4.218955 | 0.681903 | 0.080713 | 0.020529 | 0.010795 | 0 |
| 1.502686 | 2.197431 | 6.219187 | 0.6015 | 2.804584 | 0.268408 | 0.299272 | 0.054266 | 0.033231 |
| 1.32165 | 0.553146 | 3.658121 | 0.21854 | 0.492505 | 0.082211 | 0.135916 | 0.028588 | 0.029178 |
| 2.642518 | 0.285285 | 1.364779 | 0.045738 | 2.768337 | 0.37853 | 0.079649 | 0 | 0 |
| 1.08317 | 0.742945 | 26.11722 | 1.948415 | 4.295932 | 0.922196 | 0.017626 | 0.055611 | 0.00946 |
| 0.177957 | 0.121925 | 5.388457 | 0.581127 | 0.381881 | 0.030228 | 0.007139 | 0 | 0.682009 |
| 0.60238 | 0.267022 | 7.165956 | 0.747268 | 4.347405 | 1.026544 | 0 | 0.110405 | 0 |
| 0.847458 | 0.116455 | 5.22362 | 0.754557 | 3.760411 | 0.681243 | 0.064097 | 0 | 0.245715 |
| 0.338373 | 0.194416 | 1.760898 | 0.571284 | 2.885048 | 0.262945 | 0 | 1.055049 | 0.035894 |
| 0.436306 | 0.285 | 3.46 | 0.425376 | 2.137455 | 0.135545 | 0.883918 | 0.091652 | 0.013363 |
| 2.248901 | 2.182779 | 24.93985 | 0.828344 | 2.250351 | 0.150255 | 1.264638 | 0.534379 | 0.02424 |
| 0.403017 | 1.218243 | 17.03726 | 2.13478 | 0.735594 | 0.157336 | 0 | 0.51673 | 0 |
| 0.300869 | 0.147658 | 7.161053 | 0.612545 | 2.312212 | 0.819302 | 0 | 0.071227 | 0.051926 |
| 0.920957 | 0.490318 | 89.23385 | 4.202002 | 3.945255 | 0.523968 | 0.091623 | 0.140159 | 0.008941 |
| 0.503756 | 0.064495 | 0.649056 | 0.222958 | 8.881606 | 2.683934 | 0 | 0 | 0 |
| 0.208222 | 0.085839 | 0.098727 | 0.050871 | 1.543138 | 0.1633 | 0 | 0.124221 | 0 |
| 0.121769 | 0.032271 | 0.43302 | 0.039666 | 0.715134 | 0.161154 | 0.029603 | 0.487758 | 0.010592 |
| 0.276394 | 0.073595 | 5.402346 | 0.227481 | 0.647564 | 0.072063 | 0 | 0.025059 | 0 |
| 0.18607 | 0.201437 | 30.74863 | 3.297998 | 0.306097 | 0.093887 | 0 | 0.48973 | 0 |
| 0.2891 | 0.043353 | 2.157661 | 0.098098 | 0.140364 | 0.00656 | 0.043385 | 0.228136 | 0 |
| 0.930107 | 0.174225 | 6.817051 | 0.780578 | 6.213291 | 1.479075 | 0.019179 | 0.110936 | 0 |
| 0.119518 | 0.049858 | 14.663 | 2.749617 | 0.299427 | 0.048819 | 0.008071 | 0 | 0 |
| 0.267704 | 0.091662 | 0.590373 | 0.12675 | 0.217635 | 0.07629 | 0 | 0.371411 | 0 |
| 1.933449 | 11.68154 | 3.489917 | 0.630311 | 0.180378 | 0.007439 | 0.07379 | 0.025868 | 0 |
| 0.514802 | 0.181249 | 29.31732 | 1.982269 | 0.606395 | 0.06857 | 0 | 0.238447 | 0.048673 |
| 1.074584 | 0.245911 | 0.28624 | 0.081939 | 0.175866 | 0.024659 | 0.016307 | 0 | 29.18773 |
| 0.198711 | 0.058513 | 20.3615 | 2.337463 | 1.574533 | 0.357136 | 0.017892 | 0.696228 | 0 |
| 0.992156 | 0.800783 | 23.55665 | 3.066435 | 3.604147 | 0.527332 | 0.448018 | 1.222512 | 0.051989 |
| 1.016609 | 0.123111 | 0.678165 | 0.03584 | 3.938434 | 0.61479 | 0 | 0.037507 | 0.114842 |
| 0.865154 | 0.866517 | 417.1836 | 25.13869 | 3.641587 | 0.43272 | 0 | 0.225714 | 0.947072 |
| 0.384927 | 0.162894 | 0.355185 | 0.075083 | 2.111077 | 0.16947 | 0.242821 | 0.255373 | 0.040098 |
| 0.215937 | 0.085027 | 19.94126 | 3.128531 | 0.087775 | 0.018461 | 0 | 1.35885 | 0.01092 |
| 0.816286 | 0.217782 | 0.649391 | 0.022307 | 0.957564 | 0.067133 | 0.022198 | 0 | 0.929236 |
| 1.36527 | 0.124337 | 30.53149 | 3.954461 | 11.36581 | 1.793742 | 0.114059 | 0.059977 | 0.244857 |
| 1.657039 | 0.136988 | 43.80031 | 4.798814 | 3.99597 | 0.694946 | 0 | 0 | 0 |

| | | | | | | | | |
|---|---|---|---|---|---|---|---|---|
| 0.214168 | 0.043493 | 0.26262 | 0.16539 | 0.116174 | 0.036199 | 0.014962 | 1.148652 | 0 |
| 0.526278 | 0.343147 | 5.436225 | 1.469434 | 1.208974 | 0.44222 | 0.060925 | 0.544636 | 0 |
| 1.003818 | 0.398166 | 27.95414 | 1.045177 | 1.946832 | 0.12357 | 0.157863 | 1.220761 | 0 |
| 0.749437 | 0.210173 | 13.64255 | 3.959802 | 0.218321 | 0 | 0 | 0.342167 | 0 |
| 0.660586 | 0.357124 | 25.23442 | 2.511183 | 5.493487 | 1.106718 | 0.020911 | 0.428839 | 0.246898 |
| 16.87893 | 148.7119 | 3.283352 | 0.071611 | 4.856859 | 0.280163 | 0.017815 | 0.037471 | 0 |
| 1.618667 | 1.009312 | 1.087084 | 0.140035 | 1.394581 | 0.264095 | 0.046449 | 0 | 0.259258 |
| 1.003322 | 0.86873 | 0.590222 | 0.152061 | 3.202761 | 0.811515 | 0.342977 | 0.244007 | 0.010828 |
| 0.160995 | 0.156228 | 3.209523 | 0.417165 | 0.102327 | 0.03587 | 0 | 4.038298 | 0.015913 |
| 1.557569 | 1.594028 | 11.52668 | 3.522156 | 0.563264 | 0.096991 | 0.057269 | 0 | 0.036883 |
| 1.394722 | 0.228984 | 13.08325 | 1.5117 | 3.525172 | 1.057428 | 0.020328 | 0.17103 | 0 |
| 0.616263 | 0.047339 | 6.479158 | 0.087281 | 3.053497 | 0.144468 | 0 | 0.159848 | 0.209757 |
| 0.778695 | 4.297258 | 2.456995 | 0.193893 | 1.889824 | 0.398162 | 0.045398 | 0.023872 | 0.012182 |
| 0.264898 | 0.048002 | 13.81599 | 1.593046 | 1.745193 | 0.349578 | 0.008256 | 0.286543 | 0 |
| 1.10341 | 0.343219 | 96.71364 | 6.074925 | 1.303861 | 0.114264 | 0 | 0 | 0.202771 |
| 0.082596 | 0.009601 | 0.489531 | 0.212412 | 0.189958 | 0.015981 | 0 | 2.213675 | 0.066173 |
| 0.572916 | 0.110024 | 12.93265 | 1.37941 | 1.933116 | 0.122097 | 0 | 0.063688 | 0 |
| 0.768199 | 0.716004 | 3.494428 | 0.367999 | 3.648284 | 0.87016 | 0.061032 | 0.036678 | 0 |
| 0.423655 | 0.155939 | 1.62471 | 0.152211 | 1.916582 | 0.244305 | 0.025244 | 0.026549 | 0 |
| 0.872946 | 0.452054 | 2.354959 | 0.114398 | 3.633869 | 0.924625 | 0.07318 | 0.145374 | 0.008728 |
| 0.292058 | 0.139921 | 5.742422 | 1.283598 | 3.492348 | 0.761226 | 0 | 0.355583 | 0 |
| 0.29736 | 0.097291 | 2.023489 | 0.156956 | 0.539 | 0.026992 | 0 | 0.187724 | 0 |
| 0.180093 | 0.17677 | 34.07697 | 3.856677 | 0.443026 | 0.057215 | 0 | 0.255809 | 0.014505 |
| 2.048912 | 0.568999 | 23.5192 | 3.305492 | 5.622828 | 1.100602 | 0.017496 | 0.791217 | 5.023614 |
| 0.731836 | 0.723461 | 16.1105 | 1.426106 | 3.398914 | 0.384341 | 0 | 0.089102 | 0 |
| 5.280132 | 0.363147 | 21.54865 | 1.897048 | 2.13162 | 0.375009 | 0 | 0 | 0.158921 |
| 0.456152 | 0.179093 | 2.072699 | 0.092869 | 1.541445 | 0.130427 | 0.030804 | 0.874706 | 0 |
| 0.675496 | 0.717192 | 3.496037 | 0.18595 | 1.563161 | 0.447688 | 0.077098 | 0.145951 | 0.008276 |
| 0.232121 | 0.068351 | 12.77904 | 0.917156 | 0.601066 | 0.054781 | 0.006967 | 0.652094 | 0 |
| 0.499511 | 0.036051 | 0.212846 | 0.01994 | 0.039945 | 0 | 0.013228 | 0.006956 | 0 |
| 1.093232 | 1.485033 | 12.54734 | 5.308202 | 4.147731 | 1.185486 | 0.026367 | 0.009243 | 0.188676 |
| 0.520826 | 0.426012 | 24.26592 | 2.591996 | 7.914913 | 1.382817 | 0 | 0.462372 | 0.031461 |
| 0.401108 | 0.75929 | 28.63194 | 1.866572 | 0.600933 | 0.043211 | 0.196456 | 0.187828 | 0.01917 |
| 0.642694 | 0.292725 | 82.56742 | 9.64883 | 0.428328 | 0.039661 | 0.009367 | 0 | 0.140764 |
| 0.173133 | 0.050071 | 1.337628 | 0.062944 | 0.518771 | 0.022731 | 0.006263 | 0.052698 | 0.047062 |
| 3.348765 | 1.311599 | 34.24407 | 0.675541 | 8.327547 | 0.649681 | 0.496859 | 0 | 0 |
| 0.197267 | 0.07503 | 0.09743 | 0.020081 | 0.59765 | 0.028202 | 0 | 0 | 0.214486 |
| 0.388225 | 0.516979 | 8.44162 | 1.449897 | 0.847132 | 0.309075 | 0 | 0 | 2.570317 |
| 0.33928 | 0.138331 | 48.75518 | 7.353801 | 1.423248 | 0.313415 | 0 | 0.055606 | 0 |
| 0.693995 | 0.079827 | 1.928046 | 0.35323 | 0.682323 | 0.132879 | 0 | 0.508287 | 0 |
| 1.106179 | 0.883547 | 0.911977 | 0.181003 | 2.31299 | 0.51539 | 0.076202 | 0.014571 | 1.003831 |
| 0.416377 | 0.336937 | 16.38908 | 1.578943 | 0.922221 | 0.070108 | 0 | 0.582403 | 0.013824 |
| 1.080721 | 0.018793 | 1.563477 | 0.155924 | 12.64271 | 4.327507 | 0.01724 | 0.163178 | 1.18431 |
| 1.816468 | 5.926638 | 18.68357 | 0.300029 | 1.607082 | 0.211991 | 0.356968 | 13.07826 | 0.062699 |
| 0.756929 | 0.73135 | 10.37425 | 0.914997 | 1.529742 | 0.222224 | 0 | 0.067198 | 0 |
| 0.313197 | 0.192588 | 1.856254 | 0.255058 | 2.5375 | 0.505704 | 0.156761 | 0.463191 | 0.016025 |
| 0.11417 | 0.019102 | 1.014992 | 0.11622 | 0.199555 | 0.050874 | 0 | 0.044228 | 0 |
| 0.151221 | 0.047228 | 7.387335 | 0.223909 | 1.201439 | 0.247076 | 0 | 2.714273 | 0 |
| 0.492959 | 0.072579 | 2.086905 | 0.143375 | 1.15705 | 0.258889 | 0 | 0.180054 | 0 |
| 0.564659 | 0.047058 | 6.566953 | 1.258055 | 1.489745 | 0.469995 | 0 | 0 | 0 |
| 0.468686 | 0.09518 | 12.64381 | 0.592267 | 1.69491 | 0.039609 | 0 | 0 | 0.281157 |
| 0.551377 | 0.177929 | 7.878765 | 0.768877 | 0.501673 | 0.166601 | 0.091812 | 0.080465 | 0.008212 |
| 0.555231 | 0.20621 | 7.708045 | 0.71287 | 1.171567 | 0.110332 | 0 | 0 | 0 |
| 0.377519 | 0.063162 | 6.610736 | 0.57645 | 0.734842 | 0.042056 | 0 | 0.895761 | 0.037316 |
| 1.910867 | 1.202376 | 16.22875 | 0.999762 | 3.797112 | 0.34666 | 0.551492 | 0.159216 | 0.011607 |
| 0.534034 | 0.786267 | 27.58724 | 2.711416 | 3.641515 | 0.484746 | 0 | 0 | 0 |
| 0.462076 | 0.340161 | 2.076545 | 0.496221 | 0.798778 | 0.105779 | 0.041148 | 0.054094 | 0.011042 |
| 0.918803 | 0.630172 | 0.20294 | 0.023237 | 1.635881 | 0.321687 | 0.092493 | 0.972739 | 0 |

| | | | | | | | | |
|---|---|---|---|---|---|---|---|---|
| 0.1624 | 0.076513 | 4.106715 | 1.075662 | 0.666114 | 0.068989 | 0.026321 | 0.424446 | 0.009417 |
| 0.381051 | 0.172364 | 0.925133 | 0.168244 | 0.625909 | 0.050632 | 0.046505 | 2.200887 | 0.009983 |
| 0.473458 | 0.239525 | 0.395572 | 0.067942 | 4.090344 | 0.914994 | 0.025353 | 0.088878 | 0.009071 |
| 1.453104 | 7.136786 | 1.034117 | 0.033303 | 0.733841 | 0.053453 | 0.176743 | 0 | 0 |
| 0.593287 | 0.303428 | 11.03005 | 0.457725 | 0.982415 | 0.030611 | 0.025304 | 3.193465 | 0 |
| 0.286229 | 0.368743 | 73.31731 | 10.14333 | 0.78171 | 0.241139 | 0 | 0 | 0.486273 |
| 0.34263 | 0.07707 | 55.22379 | 11.66491 | 0.282803 | 0.011663 | 0 | 0.081114 | 0 |
| 0.2114 | 0.103749 | 2.297033 | 0.251064 | 0.092376 | 0.043175 | 0 | 0 | 0.229854 |
| 0.218828 | 0.42024 | 56.70488 | 9.057553 | 0.472286 | 0.004664 | 0.00771 | 0.040543 | 0 |
| 1.451672 | 0.133583 | 1.862401 | 0.14417 | 1.655465 | 0.352523 | 0.008966 | 0.075439 | 0.009624 |
| 2.9978 | 0.924022 | 0.357904 | 0.061472 | 2.397489 | 1.046557 | 0.017477 | 0.00919 | 0 |
| 0.365618 | 0.454907 | 14.22249 | 0.587109 | 1.476132 | 0.252412 | 0 | 1.272734 | 0.067189 |
| 2.602485 | 0.209424 | 15.67419 | 1.136916 | 3.885844 | 1.252399 | 0.021346 | 0.022449 | 0.183298 |
| 4.149576 | 4.604053 | 265.4929 | 11.96671 | 14.36338 | 0.799442 | 0.08896 | 0 | 0.327386 |
| 0.26535 | 0.02921 | 3.968438 | 0.181761 | 1.499766 | 0.401134 | 0.010048 | 0.010568 | 0.064713 |
| 0.145446 | 0.233319 | 33.11726 | 5.68366 | 0.129008 | 0.00532 | 0 | 2.525389 | 0.018883 |
| 0.467129 | 0.163198 | 2.862431 | 0.193432 | 0.539711 | 0.097021 | 0.01604 | 0 | 0.086086 |
| 1.982325 | 0.561271 | 0.723005 | 0.020697 | 4.637588 | 0.336344 | 0.12357 | 0.043319 | 1.989565 |
| 1.008524 | 8.301375 | 1.05022 | 0.537306 | 0.461288 | 0.04158 | 0.007638 | 0.024099 | 0.032794 |
| 0.1798 | 0.017079 | 0.550007 | 0.425101 | 0.202754 | 0.042644 | 0 | 0.148293 | 0.227026 |
| 0.193386 | 0.16609 | 5.157715 | 0.546832 | 3.436489 | 0.447622 | 0 | 0.663835 | 0 |
| 0.513704 | 0.268483 | 20.5347 | 2.453926 | 1.920169 | 0.354262 | 0.045053 | 0 | 0.029015 |
| 0.704125 | 0.209506 | 0.727039 | 0.049949 | 0.394518 | 0.04209 | 0.039763 | 0.721368 | 0 |
| 0.339136 | 0.126811 | 10.78377 | 0.767173 | 0.827996 | 0.118737 | 0.021811 | 0.734047 | 0 |
| 0.438518 | 0.153723 | 1.05198 | 0.106284 | 0.948972 | 0.08956 | 0.063457 | 1.290257 | 0 |
| 0.408551 | 0.169177 | 0.832356 | 0.043322 | 0.877746 | 0.119945 | 0.043109 | 0.018135 | 0 |
| 0.311088 | 0.170122 | 15.2505 | 1.158129 | 4.606295 | 0.637164 | 0.018007 | 0.132563 | 0 |
| 0.308864 | 0.028226 | 0.959472 | 0.130101 | 0.796753 | 0.10963 | 0 | 0.018154 | 0 |
| 1.931036 | 0.045429 | 3.718436 | 0.575843 | 1.896594 | 0.535648 | 0.010418 | 0.219139 | 0.257208 |
| 0.742855 | 1.084449 | 0.149246 | 0.076901 | 2.816914 | 0.154288 | 0.025508 | 0.134132 | 0 |
| 0.199896 | 0.024871 | 0.600715 | 0.05732 | 0.196842 | 0 | 0 | 0.047989 | 0 |
| 0.118296 | 0.032254 | 4.959763 | 0.615486 | 0.850045 | 0.214757 | 0.008876 | 0.606779 | 0 |
| 0.622727 | 0.009238 | 1.152786 | 0.044709 | 0.04935 | 0.011533 | 0 | 0.661732 | 0 |
| 0.506207 | 0.18509 | 1.967008 | 0.354908 | 0.287117 | 0.024648 | 0.013583 | 8.299785 | 0.16767 |
| 0.43971 | 0.194914 | 14.43148 | 2.493128 | 1.223085 | 0.260721 | 0.009579 | 0 | 0.52436 |
| 1.729138 | 0.265379 | 1.665793 | 0.360747 | 7.849501 | 0.576519 | 0.024757 | 0 | 0.03986 |
| 0.438556 | 0.280605 | 12.5589 | 2.061101 | 2.765284 | 0.713196 | 0.008304 | 0.09606 | 0 |
| 0.327304 | 0.094577 | 5.040015 | 0.647682 | 0.224558 | 0.074968 | 0.012394 | 0.130349 | 0 |
| 0.391472 | 0.01601 | 65.15821 | 2.745244 | 0.646232 | 0.013325 | 0 | 2.038874 | 0.82764 |
| 27.21582 | 17.40848 | 6.945751 | 0.330198 | 5.212397 | 0.134633 | 0 | 0 | 0 |
| 0.575654 | 0.260783 | 5.642571 | 0.495841 | 2.302592 | 0.393402 | 0 | 0.141521 | 0 |
| 0.316928 | 0.1731 | 0.72711 | 0.333028 | 0.750515 | 0.162863 | 0 | 0.130695 | 0 |
| 0.800116 | 0.232943 | 0.428667 | 0.276097 | 1.216779 | 0.210496 | 2.747402 | 0.096314 | 0.03932 |
| 0.372627 | 0.367635 | 24.08841 | 4.559659 | 0.651307 | 0.164759 | 0.007783 | 0.990371 | 0 |
| 0.634263 | 0.230949 | 18.83615 | 4.859767 | 4.601347 | 0.551582 | 0.082901 | 0.552179 | 0 |
| 0.282386 | 0.122283 | 15.94883 | 0.811645 | 0.696813 | 0 | 0 | 0.707838 | 0 |
| 0.333514 | 0.23064 | 6.828503 | 1.502051 | 2.596746 | 0.532536 | 0.04095 | 0.279933 | 0.571412 |
| 1.796856 | 8.154461 | 0.380343 | 0.079843 | 4.41815 | 1.031038 | 0.057782 | 0.022788 | 0.093034 |
| 0.765063 | 0.67719 | 15.16856 | 1.409265 | 5.667341 | 0.736617 | 0.018452 | 0.485141 | 0 |
| 2.677037 | 9.222164 | 155.896 | 16.33448 | 7.79624 | 1.989785 | 0.30714 | 0.280515 | 0 |
| 0.133755 | 0.059079 | 2.061116 | 0.081694 | 1.800161 | 0.114733 | 0 | 0.512969 | 0 |
| 0.774526 | 0.268317 | 7.134393 | 1.347422 | 1.751947 | 0.099906 | 0.029148 | 0.1124 | 0.062573 |
| 0.196984 | 0.03867 | 6.226585 | 0.855561 | 2.249456 | 0.836803 | 0 | 0.167881 | 0 |
| 0.9932 | 0.419337 | 7.935224 | 0.792972 | 3.565595 | 0.912804 | 0.02959 | 0.03112 | 0 |
| 0.272905 | 0.030211 | 0.121613 | 0 | 0.136287 | 0.010058 | 0.008314 | 0.139901 | 0 |
| 2.495251 | 0.43833 | 16.23932 | 2.72242 | 2.37253 | 0.197869 | 0.020446 | 0 | 0.065838 |
| 0.537442 | 0.058183 | 7.682224 | 0.289639 | 1.298561 | 0.174331 | 0.192144 | 0 | 0 |
| 0.444166 | 0.138853 | 0.613043 | 0.074325 | 1.573966 | 0.223678 | 0 | 0.012964 | 0.013231 |

| | | | | | | | | |
|---|---|---|---|---|---|---|---|---|
| 0.510835 | 0.335968 | 8.698629 | 1.182561 | 1.541007 | 0.343177 | 0 | 0.01105 | 0 |
| 4.256336 | 4.361963 | 0.718387 | 0.313213 | 16.38659 | 3.227701 | 0.094447 | 0 | 1.885617 |
| 0.565868 | 0.299469 | 3.771075 | 0.329161 | 1.385608 | 0.115037 | 0.190186 | 0.53338 | 0.011341 |
| 0.186644 | 1.056748 | 1.43896 | 0.179184 | 0.042437 | 0.003719 | 0.012297 | 0.536694 | 0 |
| 0.919946 | 0.515281 | 6.874679 | 1.404704 | 3.12852 | 0.196053 | 0.020258 | 0.213051 | 0 |
| 0.33366 | 0.51428 | 30.03378 | 2.48371 | 0.601422 | 0.038331 | 0.042248 | 0.533181 | 0.181392 |
| 0.164358 | 0.216514 | 0.273436 | 0.023482 | 0.191518 | 0.035334 | 0 | 0.061436 | 0.025081 |
| 1.050131 | 0.511116 | 2.623303 | 0.282709 | 4.990748 | 0.329685 | 0.316484 | 0.480775 | 0.094363 |
| 0.510677 | 0.14354 | 3.714561 | 0.368621 | 0.641051 | 0.011378 | 0.018811 | 0.079132 | 0 |
| 0.114421 | 0.058596 | 24.29551 | 3.241082 | 2.486889 | 0.402349 | 0 | 0.042398 | 0.021636 |
| 0.921025 | 0.024433 | 0 | 0 | 2.455853 | 0.162685 | 0.145687 | 0.023572 | 0.012029 |
| 0.302147 | 0.212383 | 0.207959 | 0.650865 | 5.860381 | 1.586112 | 0.505496 | 0 | 0 |
| 0.480276 | 0.233199 | 1.392969 | 0.228848 | 4.108022 | 0.37566 | 0.062106 | 10.14587 | 0 |
| 1.290756 | 1.400796 | 2.062077 | 0.349801 | 3.54367 | 0.347395 | 0.052212 | 0.045759 | 0.046703 |
| 0.666406 | 0.200796 | 5.878549 | 0.757256 | 1.376085 | 0.174718 | 0 | 0.158497 | 0 |
| 1.059786 | 1.315864 | 142.9321 | 13.97994 | 5.651037 | 1.436315 | 0 | 0 | 0 |
| 0.441054 | 0.099639 | 0.47025 | 0.047511 | 1.509188 | 0.245928 | 0.122921 | 0.029833 | 0 |
| 0.309333 | 0.351065 | 11.15289 | 1.521962 | 0.563205 | 0.07897 | 0 | 0.357 | 0.028028 |
| 0.288106 | 0.205664 | 7.554586 | 1.066474 | 4.08963 | 0.406538 | 0 | 0.669654 | 0 |
| 0.400701 | 0.08918 | 6.68279 | 0.398413 | 10.04961 | 1.575837 | 0 | 2.620814 | 0.020264 |
| 0.663655 | 0.336935 | 1.525869 | 0.256253 | 1.17999 | 0.133204 | 0.069544 | 0.060949 | 0.012441 |
| 0.722584 | 0.341089 | 0.19615 | 0.269519 | 2.487414 | 1.47351 | 1.944409 | 0.352572 | 0 |
| 0.244323 | 0.057228 | 1.56324 | 0.118703 | 0.072792 | 0.006804 | 0.01125 | 0.017747 | 0.030188 |
| 0.384602 | 0.286954 | 0.800891 | 0.084651 | 0.045422 | 0 | 0.010529 | 0.033221 | 0.090416 |
| 0.500231 | 0.31446 | 1.998718 | 0.251747 | 2.829334 | 0.275499 | 0 | 0.766426 | 0 |
| 1.593002 | 0.062106 | 0.2353 | 0.222277 | 4.666647 | 1.410844 | 0 | 0 | 2.676322 |
| 0.712837 | 0.287843 | 4.848759 | 1.041003 | 0.494175 | 0.1032 | 0.036561 | 0.025634 | 0.039244 |
| 0.477815 | 0.109281 | 1.759636 | 0.236115 | 2.748742 | 0.329709 | 0.009398 | 0.434896 | 0 |
| 0.392156 | 0.248815 | 29.38029 | 7.295864 | 1.514787 | 0.642503 | 0.017557 | 0.009233 | 0.018846 |
| 0.705686 | 0.047728 | 0.738951 | 0.020307 | 2.083362 | 0.372791 | 0.010104 | 0.510043 | 0 |
| 0.346989 | 0.241995 | 29.72952 | 5.378877 | 2.277015 | 0.208871 | 0 | 0.492872 | 0 |
| 0.682524 | 0.619175 | 2.151981 | 0.196504 | 1.534391 | 0.371719 | 0.018623 | 0 | 0 |
| 3.247468 | 0.734804 | 3.189673 | 0.38396 | 18.55614 | 4.148564 | 0.167739 | 0.117607 | 0.050013 |
| 0.214074 | 0.008518 | 15.0195 | 2.2852 | 0.182031 | 0 | 0 | 0 | 0 |
| 2.541654 | 2.606119 | 0.622942 | 0.068087 | 3.565695 | 0.321994 | 0.503303 | 0.773621 | 0.020778 |
| 0.601553 | 0.565 | 14.62994 | 1.034673 | 1.123955 | 0.025419 | 0.084048 | 0.994416 | 0.022554 |
| 2.028787 | 0.696171 | 0.358932 | 0.028453 | 10.12118 | 1.495648 | 0.16988 | 0.555835 | 0 |
| 0.566636 | 0.794017 | 0.972149 | 0.394662 | 2.076387 | 0.261907 | 0.02014 | 0.730732 | 0.129705 |
| 1.394277 | 0.112792 | 2.784803 | 0.374326 | 4.177764 | 0.325439 | 0 | 0.021763 | 4.99775 |
| 0.380445 | 0.147404 | 68.43571 | 4.022255 | 2.356565 | 0.474378 | 0 | 0.48351 | 0 |
| 0.177627 | 0.043587 | 2.15951 | 0.213272 | 1.146355 | 0.05023 | 0.018454 | 0.077633 | 0.019808 |
| 0.432653 | 0.227235 | 0.929726 | 0.082419 | 1.132126 | 0.080611 | 0.010252 | 0 | 0.011004 |
| 0.137005 | 0.020765 | 3.271874 | 0.278931 | 0.478935 | 0.034565 | 0.008164 | 0.103026 | 0 |
| 0.821223 | 0.236563 | 2.433611 | 0.16356 | 5.055101 | 2.307999 | 0.470184 | 0.114113 | 0 |
| 0.24322 | 0.908406 | 0.127192 | 0.03641 | 0.812727 | 0.083277 | 0.014492 | 0.007621 | 0 |
| 0.350851 | 0.075965 | 1.437249 | 0.273117 | 1.316675 | 0.271871 | 0 | 0.098939 | 0.01122 |
| 0.430813 | 0.441248 | 9.547285 | 1.220317 | 1.942547 | 0.397854 | 0 | 0.079818 | 0 |
| 1.568903 | 0.236139 | 1.608665 | 0.133962 | 2.745848 | 0.428352 | 0.074983 | 0.227816 | 0 |
| 1.514434 | 0.456081 | 0.625582 | 0.056059 | 5.438482 | 1.017876 | 0.018595 | 0 | 0.019959 |
| 0.079857 | 0.046317 | 10.72804 | 0.788279 | 0.076135 | 0.011861 | 0.049025 | 0.360917 | 0 |
| 1.076592 | 0.381594 | 0.630197 | 0.29766 | 1.719132 | 0.814359 | 2.988893 | 0.283189 | 0.173417 |
| 0.582775 | 0.208007 | 5.023985 | 0.599235 | 4.650672 | 0.62036 | 0 | 0 | 0 |
| 0.053753 | 0.029157 | 1.073124 | 0.046079 | 1.295575 | 0.166407 | 0.011463 | 3.749299 | 0 |
| 0.139007 | 0.117429 | 1.647728 | 0.157742 | 0.557629 | 0.055849 | 0.009233 | 0.563214 | 0 |
| 0.25 | 0.212939 | 1.224546 | 0.100955 | 1.343412 | 0.106337 | 0.025115 | 1.549557 | 0.026958 |
| 1.146696 | 0.067532 | 1748.603 | 108.4181 | 1.403 | 0.224827 | 0 | 0.024432 | 3.191785 |
| 0.260021 | 0.153133 | 1.489722 | 0.370568 | 1.817941 | 0.371736 | 0 | 0.277005 | 0.018848 |
| 0.653772 | 0.145148 | 1.783626 | 0.359167 | 2.575642 | 0.216179 | 0.084094 | 0 | 0 |

| | | | | | | | | |
|---|---|---|---|---|---|---|---|---|
| 1.343721 | 0.404469 | 12.03443 | 0.899745 | 6.137837 | 0.380548 | 0 | 0.356282 | 0 |
| 0.433942 | 0.25556 | 4.286474 | 0.147245 | 5.84028 | 1.75479 | 0.058609 | 0.246553 | 0 |
| 0.477818 | 0.246502 | 1.337921 | 0.245115 | 1.861046 | 0.470261 | 0.083844 | 0.264535 | 0.016363 |
| 0.519705 | 0.106387 | 1.581271 | 0.181062 | 0.893808 | 0.272449 | 0.822033 | 0.094742 | 0.012087 |
| 0.745543 | 0.167517 | 8.304989 | 1.414238 | 0.502407 | 0.014676 | 0.012132 | 0.11483 | 0.612035 |
| 2.178085 | 0.447465 | 4.992981 | 0.542768 | 4.902076 | 0.294019 | 1.166616 | 0.465775 | 3.930592 |
| 0.114704 | 0.14969 | 6.921899 | 0.636898 | 0.109358 | 0.012778 | 0.021126 | 0.111088 | 0.170069 |
| 0.469694 | 0.232729 | 8.083678 | 1.342444 | 2.162938 | 0.243509 | 0.018299 | 0.115471 | 0.019642 |
| 0.322774 | 0.218976 | 198.3158 | 19.83023 | 1.781006 | 0.736769 | 0 | 0.202269 | 0 |
| 0.253205 | 0.342241 | 2.558565 | 0.405645 | 1.842843 | 0.152596 | 0 | 0.159194 | 0 |
| 0.180679 | 0.022801 | 1.346205 | 0.105099 | 0.27069 | 0.012652 | 0 | 7.193268 | 0 |
| 0.257162 | 0.04437 | 2.786335 | 0.188155 | 0.512698 | 0.044315 | 0.065124 | 0.710584 | 0.017476 |
| 0.269684 | 0.023356 | 0.376085 | 0.064595 | 0.436715 | 0.082618 | 0.024104 | 0.321099 | 0.051746 |
| 0.720878 | 0.228826 | 7.565065 | 0.242365 | 2.635613 | 0.048626 | 0 | 0.12682 | 0 |
| 1.478597 | 0.848265 | 4.955822 | 0.705866 | 2.905233 | 0.29365 | 0.082635 | 0.119496 | 0 |
| 1.390469 | 0.973847 | 62.83783 | 2.162483 | 6.582258 | 0.5088 | 0.919429 | 0.267456 | 0.587939 |
| 0.354719 | 0.126834 | 47.09264 | 3.590273 | 0.744008 | 0.074516 | 0 | 0.561436 | 0.022039 |
| 0.760615 | 1.941668 | 24.51123 | 7.878188 | 46.55647 | 18.45694 | 0.761682 | 0.123239 | 0 |
| 0.136276 | 0.068014 | 2.327211 | 0.26334 | 0.742841 | 0.084912 | 0.009359 | 1.998033 | 0.010046 |
| 0.075319 | 0 | 6.725796 | 0.736053 | 0.54415 | 0 | 0 | 0.14978 | 0.611475 |
| 0.205243 | 0.030218 | 3.568202 | 0.668574 | 0.693564 | 0.050301 | 0.02772 | 0.029153 | 0.53558 |
| 0.602959 | 0.415368 | 0.917994 | 0.045049 | 3.616135 | 0.447389 | 0.022414 | 0.023572 | 0.072175 |
| 0.487601 | 0.648984 | 1.202203 | 0.04765 | 0.981812 | 0.066921 | 0.015805 | 1.180192 | 0.016965 |
| 0.096074 | 0.013261 | 0.960879 | 0.07335 | 0.041981 | 0 | 0.012165 | 0.28146 | 0 |
| 0.574525 | 0.073555 | 28.17458 | 2.456737 | 0.261965 | 0.042383 | 0.007786 | 0 | 1.880293 |
| 1.60035 | 0.333319 | 43.39942 | 6.548164 | 15.25508 | 1.970643 | 0.094893 | 0 | 0.475329 |
| 1.356113 | 2.283436 | 5.387874 | 0.467784 | 2.400878 | 0.459057 | 0.182146 | 0.063854 | 0 |
| 0.726365 | 0.141543 | 5.15969 | 0.978633 | 6.562703 | 0.981719 | 0.064922 | 0.102416 | 0 |
| 0.279399 | 0.213028 | 3.814575 | 0.832936 | 0.697651 | 0.134505 | 0.010108 | 0.924838 | 0.01085 |
| 0.368864 | 0.489541 | 1.662728 | 1.163486 | 4.104472 | 4.634667 | 1.789278 | 0.044277 | 0 |
| 0.349246 | 0.142523 | 9.967076 | 1.545736 | 0.643617 | 0.018607 | 0.023072 | 0.008088 | 0.03302 |
| 0.981955 | 1.556961 | 4.476808 | 0 | 1.848366 | 1.110732 | 0.306055 | 0.643752 | 0.657028 |
| 2.25204 | 1.434162 | 12.62363 | 2.358981 | 5.777866 | 1.545132 | 0.046027 | 0.012102 | 0.012351 |
| 0.668277 | 0.230182 | 17.55082 | 2.746086 | 7.2013 | 1.427459 | 0.037263 | 0.052252 | 0.039997 |
| 0.345806 | 0.142916 | 0.503392 | 0.024703 | 0.5302 | 0.029737 | 0.024582 | 0 | 0.052771 |
| 1.088622 | 0.072854 | 2.228888 | 0.382824 | 0.743814 | 0.024255 | 0 | 0.084344 | 0.043042 |
| 0.138815 | 0.168312 | 0.508154 | 0.058186 | 0.199814 | 0.020012 | 0 | 6.219733 | 0 |
| 0.177322 | 0.070708 | 1.357482 | 0.150423 | 0.374509 | 0.063377 | 0.119747 | 0.015742 | 0.032133 |
| 0.449786 | 0.580524 | 1.177103 | 0.083248 | 0.296091 | 0.03579 | 0 | 0.883657 | 0 |
| 0.239368 | 0.149514 | 5.388133 | 1.132191 | 1.749614 | 0.402948 | 0 | 0.236973 | 0 |
| 1.372502 | 0.157872 | 10.99434 | 0.873221 | 4.310645 | 0.656981 | 0 | 0 | 0.34976 |
| 0.294968 | 0.103051 | 0.711139 | 0.122142 | 2.901167 | 0.428846 | 0 | 0.10652 | 0.173948 |
| 0.383567 | 0.43125 | 3.05865 | 0.340762 | 9.385994 | 1.965557 | 0.028257 | 0.089154 | 0 |
| 0.682169 | 0.157711 | 30.40661 | 2.892467 | 2.857697 | 0.359244 | 0.045687 | 0.024024 | 0 |
| 2.572061 | 0.729818 | 68.12586 | 5.420008 | 4.956125 | 0.1869 | 0.042136 | 0.073856 | 0.814098 |
| 0.525698 | 0.403122 | 22.1994 | 1.560826 | 3.235141 | 0.174469 | 0.044376 | 0.163344 | 0 |
| 0.532029 | 0.413021 | 2.350589 | 0.384033 | 1.420246 | 0.35561 | 0.009799 | 0.360679 | 0 |
| 0.195718 | 0.087853 | 9.223249 | 0.680308 | 0.300373 | 0.035098 | 0.009671 | 2.247752 | 0 |
| 0.372143 | 0.136978 | 0.866492 | 0.027059 | 1.463537 | 0.065147 | 0 | 0.056636 | 0.028902 |
| 3.117563 | 2.385111 | 3.663469 | 0.143581 | 7.758567 | 0.864201 | 0.756398 | 0.088389 | 0 |
| 0.810824 | 0.317827 | 3.869438 | 0.900423 | 1.067519 | 0.129038 | 0 | 0 | 0.045797 |
| 0.131846 | 0.01688 | 1.494901 | 0.093366 | 0.060118 | 0.009366 | 0.023227 | 0.154708 | 0.099725 |
| 0.938619 | 0.134605 | 115.8839 | 7.916818 | 3.132042 | 0.567626 | 0 | 0.025972 | 0.053016 |
| 0.716877 | 0.63768 | 2.257168 | 0.152032 | 5.273106 | 1.07978 | 0.105899 | 0.095463 | 0 |
| 0.445166 | 0.312107 | 31.91216 | 4.186349 | 4.038689 | 0.389649 | 0.128838 | 0.045166 | 0.138293 |
| 1.546692 | 0.310946 | 75.83887 | 7.790163 | 16.24231 | 4.277802 | 0.050337 | 0 | 0.216123 |
| 0.651214 | 0.183042 | 43.50836 | 1.566879 | 1.303806 | 0.087055 | 0 | 0.605455 | 0.077243 |
| 0.995608 | 0.5353 | 0.451014 | 0.077464 | 2.172483 | 0.191681 | 0.068519 | 0.081068 | 0 |

| | | | | | | | | |
|---|---|---|---|---|---|---|---|---|
| 0.798525 | 0.266536 | 3.027748 | 0.141368 | 2.782781 | 0.24311 | 0.060289 | 0.063405 | 0 |
| 0.223216 | 0.065729 | 0.158755 | 0.009089 | 0.358943 | 0.005471 | 0.06331 | 0.380475 | 0.009708 |
| 0.626462 | 0.067639 | 10.86646 | 0.790765 | 0.883284 | 0.322421 | 0 | 0.035594 | 0 |
| 1.930231 | 0.605752 | 1.383001 | 0.058343 | 4.765553 | 0.316044 | 0.116112 | 0.104669 | 0.124632 |
| 1.19956 | 0.429154 | 4.119552 | 0.045649 | 1.136522 | 0.109903 | 0 | 0 | 0.195031 |
| 0.466694 | 0.206136 | 66.21802 | 6.8601 | 4.290711 | 0.766331 | 0 | 0.178984 | 0.101486 |
| 0.36933 | 0.111543 | 18.18506 | 2.660664 | 2.81391 | 0.591834 | 0 | 0.645669 | 0 |
| 2.273267 | 0.048407 | 0.361883 | 0.019125 | 2.200145 | 0.328065 | 0.009515 | 0 | 0.010214 |
| 0.770804 | 0.274825 | 1.353599 | 0.080477 | 1.727272 | 0.048438 | 0.418202 | 0.121652 | 0 |
| 0.529646 | 0.216769 | 0.22665 | 0.031143 | 4.404867 | 0.445184 | 0.178191 | 0.602945 | 0 |
| 0.67926 | 0.125861 | 18.15734 | 3.452597 | 0.508828 | 0.141559 | 0 | 0.895921 | 0 |
| 0.878005 | 0.047878 | 0.337281 | 0.231719 | 1.449388 | 0.199243 | 0.03294 | 0 | 0.265179 |
| 0.457644 | 0.200957 | 289.7469 | 26.49702 | 3.676762 | 0.757571 | 0.016266 | 0.085533 | 0 |
| 0.271537 | 0.297188 | 44.97624 | 11.58495 | 7.039415 | 2.779731 | 0.292095 | 0 | 0.167216 |
| 0.817763 | 0.712229 | 156.4601 | 5.111951 | 5.45569 | 0.846837 | 0.023334 | 0 | 0.025046 |
| 0.95117 | 0.299462 | 67.57777 | 8.890886 | 6.022751 | 2.096557 | 0 | 0 | 0.130088 |
| 0.433443 | 0.166098 | 2.228751 | 0.282064 | 0.989385 | 0.184324 | 0.032077 | 0.30362 | 0 |
| 0.414162 | 0.143034 | 0.818218 | 0.437216 | 1.501433 | 0.24436 | 0.020717 | 0.370402 | 0 |
| 0.859142 | 1.368273 | 4.359784 | 0.617346 | 1.158156 | 0.289003 | 0.113761 | 0.167499 | 0 |
| 0.717577 | 0.384927 | 1.780297 | 0.385052 | 2.333466 | 0.299924 | 0.022539 | 0.165927 | 0 |
| 0.252918 | 0.379536 | 12.42434 | 1.356619 | 5.712902 | 2.670138 | 0 | 0 | 0.624027 |
| 0.83912 | 0.175022 | 5.802176 | 0.085419 | 5.108938 | 1.696632 | 0 | 0.208584 | 0 |
| 0.740311 | 0.354462 | 2.497048 | 0.392121 | 0.694327 | 0 | 0 | 0.051295 | 0.654417 |
| 0.463941 | 0.014231 | 81.1157 | 3.967102 | 0.047303 | 0.014213 | 0.015665 | 0 | 0 |
| 1.928528 | 0.157318 | 1.760837 | 0.286516 | 7.98068 | 4.8542 | 0.063357 | 0.011105 | 0.068007 |
| 0.115908 | 0.014712 | 0.165819 | 0.008137 | 0.10479 | 0.014693 | 0.008097 | 0.153285 | 0 |
| 0.696942 | 0.070162 | 3.601067 | 0.169785 | 1.603405 | 0.262781 | 0 | 0.025383 | 0 |
| 1.490079 | 0.590198 | 32.20579 | 1.071682 | 7.643577 | 0.476335 | 0.738287 | 0 | 0.070441 |
| 0.090821 | 0.033429 | 1.883973 | 0.268112 | 0.095247 | 0.005565 | 0.027599 | 0.116104 | 0 |
| 0.596986 | 0.082204 | 0.203638 | 0.717009 | 2.116947 | 0.621028 | 2.488495 | 0.402635 | 0.037358 |
| 1.473127 | 0.536361 | 20.13376 | 1.233045 | 5.651137 | 0.686358 | 0.212185 | 0.009702 | 0 |
| 0.374701 | 0.027356 | 0.697434 | 0.075656 | 0.129904 | 0.007589 | 0.025095 | 1.636294 | 0 |
| 1.091009 | 0.239702 | 33.9851 | 4.700702 | 4.915208 | 0.932741 | 0 | 0 | 0.009196 |
| 0.091876 | 0.012297 | 6.90567 | 0.756716 | 0.109493 | 0.035823 | 0.008461 | 0.605063 | 0.009081 |
| 0.374956 | 1.004072 | 14.07947 | 0.952946 | 5.955585 | 1.116304 | 0.050982 | 0.386044 | 0.010945 |
| 0.550855 | 0.611711 | 2.600777 | 0.161572 | 3.084314 | 0.812313 | 0.066203 | 0 | 0.030455 |
| 0.750876 | 0.082168 | 2.104866 | 0.082634 | 4.43392 | 0.310854 | 0 | 548.7684 | 0.110327 |
| 0.373352 | 0.28155 | 3.508067 | 0.852813 | 1.344939 | 0.150643 | 0.009224 | 0 | 0 |
| 0.420189 | 0.368097 | 0.149422 | 0.076992 | 1.784688 | 0.231706 | 0.034051 | 0.017905 | 0 |
| 0.72052 | 0.530417 | 0.434276 | 0 | 3.564692 | 0.658456 | 0.123705 | 0.936713 | 0.053113 |
| 0.454313 | 0.21123 | 33.35074 | 2.823523 | 1.030918 | 0.390679 | 0 | 0 | 0.103994 |
| 1.293435 | 0.099279 | 8.792198 | 0.399368 | 5.207267 | 0.691082 | 0 | 0.052243 | 0.079981 |
| 2.182076 | 0.152784 | 1.298397 | 0.657284 | 2.760998 | 0.247259 | 0.023359 | 0.012283 | 0.250732 |
| 1.386419 | 2.64526 | 177.7571 | 5.73095 | 0.823699 | 0.061873 | 0.613752 | 0.14344 | 0 |
| 0.167289 | 0.060368 | 1.535831 | 0.14692 | 0.303867 | 0.036176 | 0 | 6.220083 | 0 |
| 0.321706 | 0.094731 | 0.966055 | 0.20959 | 2.189235 | 0.357426 | 0 | 2.504139 | 0.018655 |
| 0.457314 | 0.536698 | 2.933002 | 0.197906 | 4.672413 | 0.812167 | 0.080563 | 0.338912 | 0 |
| 0.27583 | 0.261883 | 2.933451 | 0.398871 | 1.577019 | 0.227442 | 0.010445 | 4.350033 | 0.056057 |
| 0.423127 | 0.364508 | 3.895071 | 0.293261 | 0.692367 | 0.044128 | 0.091193 | 0.076726 | 0 |
| 0.403284 | 0.014054 | 3.903496 | 0.485832 | 0.492173 | 0.07018 | 0.009669 | 0.010169 | 0.010378 |
| 1.223681 | 1.988831 | 0.188377 | 0.194129 | 4.055477 | 0.42843 | 0 | 0 | 0.552934 |
| 0.733982 | 1.311664 | 4.633869 | 0.422311 | 0.734421 | 0.045623 | 0.420235 | 0.022665 | 0.05783 |
| 1.519126 | 0.224387 | 6.643399 | 0.900822 | 2.801332 | 0.771126 | 0.03984 | 0.02095 | 0.021382 |
| 0.552206 | 0.729237 | 12.96633 | 1.355964 | 3.455525 | 1.131237 | 0.02562 | 0.008981 | 0 |
| 0.151941 | 0.044063 | 26.49716 | 2.774681 | 2.945457 | 0.835029 | 0 | 0.03924 | 0.010012 |
| 0.357888 | 0.325019 | 7.969101 | 1.470883 | 1.753864 | 0.459051 | 0.013552 | 0.171035 | 0 |
| 0.495486 | 0.338228 | 0.640722 | 0.073365 | 4.345991 | 0.574052 | 0.073005 | 0.691004 | 0 |
| 0.522395 | 0.587651 | 1.669824 | 0.0478 | 1.087494 | 0.043156 | 0 | 2.601252 | 0 |

| | | | | | | | | |
|---|---|---|---|---|---|---|---|---|
| 0.87351 | 0.573008 | 14.52327 | 1.091691 | 3.628018 | 0.480445 | 0.268661 | 0.073708 | 0 |
| 0.710862 | 0.178975 | 17.98022 | 1.495682 | 6.558931 | 1.917055 | 0.010707 | 1.520221 | 0.034479 |
| 0.499532 | 0.925826 | 2.159515 | 0.287155 | 2.177668 | 0.259255 | 0.071436 | 2.078559 | 0.051119 |
| 0.093382 | 0.041246 | 21.36345 | 2.319451 | 0.750816 | 0.057216 | 0.018918 | 0.019896 | 0 |

| EREG | FAM3B | FGF20 | IL1RN | LHB | NODAL | RETNLB | SCG2 | SECTM1 |
|---|---|---|---|---|---|---|---|---|
| 1.407829 | 0.090213 | 0 | 0.82922 | 0.065347 | 0.151402 | 35.14984 | 5.235195 | 100.2325 |
| 1.42277 | 0.202216 | 0 | 1.222779 | 0.040928 | 0.03793 | 77.82128 | 9.029706 | 26.28481 |
| 1.254295 | 77.43225 | 0.009097 | 2.688076 | 0.293596 | 0.408138 | 12.25904 | 8.431437 | 109.3418 |
| 0.880442 | 19.75341 | 0 | 0.975661 | 0 | 0.017136 | 29.92952 | 3.590419 | 128.9348 |
| 0.995756 | 0.071573 | 0.01526 | 2.025278 | 0.049252 | 0.015215 | 161.4712 | 12.89371 | 28.18614 |
| 0.366937 | 4.406008 | 0 | 4.190362 | 0.031583 | 0.039026 | 12.42579 | 4.380079 | 39.99785 |
| 1.569589 | 0.121505 | 0 | 0.558709 | 0 | 0.072323 | 15.95779 | 3.834192 | 80.54777 |
| 0.560916 | 0.22071 | 0.017429 | 0.320762 | 0 | 0.052132 | 25.31006 | 4.133269 | 54.53242 |
| 1.319223 | 3.578204 | 0 | 4.279434 | 0.036052 | 0.011137 | 26.91865 | 5.395065 | 35.77417 |
| 0.733836 | 0.136411 | 0 | 0.356392 | 0.042669 | 0.079087 | 73.58511 | 5.589169 | 41.23984 |
| 0.806056 | 0.953781 | 0 | 3.126663 | 0 | 0.069437 | 20.68403 | 9.312881 | 24.31332 |
| 0.337694 | 0.842956 | 0 | 0.66554 | 0.075335 | 0.046545 | 31.77648 | 4.405147 | 28.50211 |
| 0.363177 | 0.380679 | 0.016735 | 0.494526 | 0.054013 | 0.025028 | 19.61576 | 4.150559 | 72.82564 |
| 2.17173 | 13.33171 | 0.014037 | 0.647662 | 0 | 0.083973 | 5.870002 | 5.228593 | 124.8649 |
| 0.93668 | 0.4397 | 0 | 0.932985 | 0 | 0.008346 | 12.44297 | 3.37253 | 75.90974 |
| 0.247462 | 2.001199 | 0.012699 | 0.895339 | 0.081971 | 0.012661 | 27.36607 | 2.514863 | 53.58503 |
| 1.402131 | 0.038182 | 0.013568 | 0.274327 | 0 | 0.06764 | 28.65957 | 3.563033 | 40.73156 |
| 1.615977 | 0.046192 | 0 | 0.889875 | 0 | 0.042084 | 31.78047 | 9.084015 | 26.0835 |
| 0.895593 | 13.45868 | 0.024073 | 1.684836 | 0.077697 | 0.072007 | 13.26357 | 3.497155 | 46.11148 |
| 0.470792 | 0.233372 | 0.011309 | 1.629815 | 0.036499 | 0.124026 | 11.58098 | 15.79615 | 32.55598 |
| 1.14673 | 0.184148 | 0.021223 | 1.435834 | 0.034249 | 0.03174 | 53.50977 | 3.708919 | 47.23344 |
| 0.84909 | 0.030935 | 0 | 1.484023 | 0.042576 | 0.039457 | 31.28679 | 3.668884 | 56.6418 |
| 1.941074 | 0.032663 | 0.027856 | 2.151791 | 0 | 0.111097 | 45.76749 | 8.344231 | 20.61268 |
| 0.793946 | 2.587492 | 0.012624 | 1.636195 | 0.122236 | 0.088109 | 28.11121 | 5.47832 | 109.0607 |
| 0.711614 | 0.026288 | 0.042037 | 2.222908 | 0 | 0.125739 | 12.16863 | 7.750915 | 29.97952 |
| 1.531587 | 0.072122 | 0.010251 | 5.133956 | 0.033087 | 0.020442 | 17.08547 | 6.052444 | 8.602582 |
| 1.021634 | 44.62519 | 0 | 0.737719 | 0.09669 | 0.029869 | 11.06475 | 2.826935 | 24.4475 |
| 1.260519 | 9.580889 | 0 | 0.994137 | 0 | 0.020126 | 4.112759 | 5.162917 | 143.8972 |
| 0.998629 | 2.865157 | 0 | 2.525863 | 0.048089 | 0.044567 | 14.53089 | 4.023708 | 10.37957 |
| 0.511279 | 8.947273 | 0 | 3.397454 | 0 | 0.028616 | 8.424964 | 2.619249 | 63.11825 |
| 2.000737 | 0.325649 | 0 | 1.189214 | 0.028366 | 0.035052 | 10.7712 | 6.596611 | 119.0301 |
| 0.282481 | 0.229702 | 0.01139 | 0.531418 | 0.07352 | 0.034068 | 22.40661 | 3.429343 | 71.47405 |
| 0.421124 | 16.39255 | 0 | 1.436595 | 0.087683 | 0.013544 | 22.01699 | 2.639512 | 62.69323 |
| 2.474799 | 0.035793 | 0.012719 | 1.971558 | 0.082101 | 0.076088 | 72.4063 | 3.624317 | 31.4176 |
| 0.535366 | 11.01432 | 0 | 2.854227 | 0 | 0.021913 | 7.513773 | 2.674312 | 207.1469 |
| 0.828926 | 0.163619 | 0.033224 | 0.947339 | 0.053616 | 0 | 122.5093 | 2.433885 | 54.74718 |
| 0.366195 | 0.085544 | 0.012159 | 0.151289 | 0 | 0.157604 | 1.577408 | 10.2813 | 11.41657 |
| 0.72847 | 0.614747 | 0 | 0.839392 | 0 | 0.122997 | 25.01715 | 5.772551 | 54.1856 |
| 0.960071 | 1.205478 | 0.011954 | 0.77469 | 0 | 0.059596 | 21.69348 | 1.781167 | 147.4351 |
| 0.484828 | 0.436108 | 0.019784 | 0.5641 | 0.063852 | 0.059175 | 16.15386 | 10.91868 | 38.15409 |
| 1.204884 | 3.254388 | 0 | 5.358971 | 0.045891 | 0.014177 | 34.56942 | 8.24459 | 108.5927 |
| 0.907277 | 0.193451 | 0 | 0.347473 | 0.033281 | 0.020562 | 52.39027 | 4.225 | 58.9067 |
| 15.7322 | 0.146552 | 0.267826 | 1.319061 | 0 | 0.296706 | 12.03577 | 1.745845 | 5.123323 |
| 2.154825 | 0.627735 | 0 | 0.255289 | 0.040752 | 0.088124 | 1.811445 | 0.203806 | 2.598491 |
| 0.121517 | 0.627348 | 0 | 6.667644 | 1.82645 | 0.025647 | 0.196292 | 0.367287 | 52.92188 |
| 28.0562 | 3.9364 | 0.010003 | 7.540482 | 0.032286 | 0.019948 | 1.312993 | 0.360195 | 5.976432 |
| 61.32556 | 0.959065 | 0.817933 | 5.936543 | 0 | 0.407759 | 0.582561 | 2.352742 | 2.642283 |
| 3.295723 | 69.89485 | 5.438812 | 1.709129 | 0.054012 | 1.451628 | 0.153246 | 0.976589 | 1.039875 |
| 4.305102 | 2.066622 | 0 | 4.340224 | 1.034281 | 0.239632 | 1.650665 | 0.049736 | 17.67473 |
| 0.225837 | 14.99083 | 0 | 0.995552 | 0.577773 | 0.178485 | 4.893023 | 0.505158 | 10.33506 |
| 1.23906 | 9.378096 | 0.028483 | 1.319133 | 0 | 0.009466 | 11.65035 | 0.772148 | 7.377269 |
| 0.59319 | 1.884561 | 0 | 74.70437 | 0.469874 | 0.029031 | 2.932931 | 0.68689 | 13.54455 |
| 0.248515 | 1.658956 | 0 | 19.01247 | 0.03234 | 0.049952 | 0.948153 | 3.862996 | 7.26592 |
| 0.302864 | 0.766193 | 0.037991 | 16.17001 | 1.471401 | 1.780297 | 0.173947 | 0.117927 | 13.49277 |
| 19.69762 | 24.61818 | 0.060593 | 1.193696 | 0.048891 | 0.453105 | 13.96421 | 0.122256 | 8.454324 |
| 21.31572 | 16.88681 | 0.023006 | 5.724954 | 0.297012 | 0.275258 | 1.158708 | 0.971214 | 6.701896 |

| | | | | | | | | |
|---|---|---|---|---|---|---|---|---|
| 0.599816 | 0.446628 | 0 | 9.972287 | 1.741621 | 0.031648 | 9.7375 | 0.335001 | 7.491495 |
| 7.408702 | 7.640164 | 1.163547 | 7.133672 | 0.163278 | 0.02522 | 0.656285 | 0.157033 | 7.008039 |
| 2.42443 | 5.919008 | 0.032779 | 0.739222 | 0.211592 | 0.179753 | 240.4856 | 0.406997 | 7.222393 |
| 7.888563 | 6.218671 | 0.085541 | 10.01501 | 0.184058 | 0.59702 | 1.544898 | 0.274378 | 5.185358 |
| 12.19557 | 0.457085 | 0.124411 | 2.977668 | 0 | 0.20674 | 0.221526 | 0.399056 | 1.430302 |
| 3.716843 | 0.756025 | 0 | 18.2653 | 1.858053 | 0.038266 | 0.292876 | 0.285917 | 38.79171 |
| 4.528064 | 0.270697 | 0.714574 | 2.30822 | 0.088704 | 0.027402 | 4.614069 | 0.102374 | 3.775941 |
| 3.384398 | 1.632782 | 3.179037 | 0.858869 | 0.072257 | 0.75893 | 104.9311 | 0.201529 | 1.257122 |
| 0.0557 | 0.156541 | 0 | 21.21513 | 0.140508 | 0.028937 | 0.044295 | 0.630623 | 46.26872 |
| 4.392134 | 11.50256 | 0.350881 | 15.72531 | 0.14901 | 0.027619 | 4.481467 | 0.653497 | 4.053223 |
| 1.737749 | 14.11694 | 1.057103 | 22.50631 | 0.184424 | 0 | 2.398256 | 0.212843 | 8.070367 |
| 16.50619 | 0.073118 | 0 | 22.64584 | 0 | 0.062174 | 1.094479 | 0.096782 | 6.949813 |
| 18.48484 | 5.653495 | 3.395017 | 8.800351 | 0.201056 | 0.962704 | 7.130567 | 1.392231 | 2.006448 |
| 1.096826 | 0.722661 | 0.052767 | 27.85912 | 0.238429 | 0.294622 | 0.483203 | 3.551023 | 0.619673 |
| 1.546574 | 0.116987 | 0 | 1.535567 | 0 | 0.279779 | 8.565387 | 1.064592 | 10.89561 |
| 5.294846 | 1.284206 | 3.129221 | 5.974336 | 0 | 0.149999 | 4.684037 | 0.952656 | 8.130462 |
| 13.15908 | 2.781354 | 0.855898 | 2.281994 | 0.295976 | 0.396208 | 4.99189 | 0.170793 | 2.979847 |
| 51.19663 | 0.733385 | 0.184796 | 4.252187 | 0.321157 | 0.014173 | 0.086781 | 2.497458 | 24.22191 |
| 4.658417 | 1.119678 | 0 | 17.6656 | 0.086089 | 0.013297 | 4.091313 | 0.198712 | 22.32233 |
| 33.6217 | 0.129083 | 1.685712 | 2.015366 | 0.666207 | 0.617413 | 3.202835 | 1.409596 | 3.059469 |
| 10.91971 | 3.660029 | 0 | 15.27211 | 0 | 0 | 5.827387 | 0.32874 | 5.322489 |
| 35.58533 | 1.086191 | 1.061444 | 3.101587 | 0.103814 | 0.03207 | 7.609078 | 7.588044 | 14.17413 |
| 2.723142 | 0.413202 | 0 | 10.79024 | 0.355428 | 0.082349 | 0.294128 | 0.837492 | 11.55136 |
| 1.055633 | 39.5655 | 0.022992 | 6.108861 | 0.148415 | 0.022924 | 44.49538 | 0.114191 | 9.817348 |
| 19.91631 | 25.69744 | 0 | 2.180251 | 0 | 0.28268 | 32.99415 | 0.073338 | 3.5962 |
| 18.13265 | 0.427148 | 0.393828 | 1.046377 | 0.158887 | 0.31904 | 4.883679 | 1.146069 | 1.501427 |
| 7.404178 | 26.40031 | 0.161439 | 14.20014 | 0.173683 | 0.321924 | 13.30513 | 0.835199 | 3.619951 |
| 9.010894 | 0.021776 | 0 | 152.6725 | 0.149847 | 0.632639 | 0.283436 | 1.950366 | 4.075987 |
| 15.57284 | 0.233372 | 0 | 6.530362 | 0.084523 | 0.104443 | 0.159875 | 0.975481 | 7.270548 |
| 20.24814 | 0.460484 | 0.009917 | 0.42159 | 0.096024 | 0.039552 | 1.634669 | 1.243665 | 0.751925 |
| 0.214517 | 0.962145 | 2.606986 | 0.420969 | 0.068968 | 0.085223 | 326.2319 | 5.717674 | 3.386552 |
| 22.66711 | 0.317923 | 2.679859 | 16.2141 | 0.101757 | 1.05306 | 2.574329 | 0.851423 | 6.012058 |
| 0.127781 | 0.572035 | 0.019672 | 2.671956 | 0 | 0.019614 | 217.9385 | 1.465495 | 17.70745 |
| 0.51313 | 0.299427 | 0.011822 | 15.74556 | 0.076314 | 0.035362 | 7.452008 | 0.32294 | 12.33363 |
| 49.90543 | 28.70103 | 0 | 0.322444 | 0.16387 | 0.189835 | 0.174353 | 0.291565 | 1.324204 |
| 32.21788 | 0.667339 | 0.049063 | 1.271792 | 0.158354 | 1.161815 | 0.786257 | 1.698111 | 1.538348 |
| 11.52743 | 4.45255 | 0.244201 | 0.99698 | 0.295563 | 0 | 32.98439 | 0.587466 | 1.757573 |
| 24.2435 | 24.37436 | 0.010287 | 9.071344 | 0.066402 | 0.338461 | 0.329698 | 0.798275 | 5.870125 |
| 0.177706 | 0.524244 | 0.08598 | 14.41245 | 0.185003 | 0.142877 | 3.018175 | 0.177927 | 25.6351 |
| 1.149855 | 12.5734 | 0 | 1.34597 | 0.097436 | 0.361198 | 8.93857 | 0.674706 | 2.288941 |
| 3.260525 | 1.447257 | 3.939205 | 24.20679 | 0.169519 | 0.039276 | 1.322661 | 0.880388 | 6.736958 |
| 5.166759 | 0.566585 | 0 | 0.966246 | 0 | 0.378542 | 11.53632 | 0.107137 | 4.092639 |
| 11.91396 | 37.53593 | 0 | 1.505334 | 0 | 0.119796 | 51.74293 | 3.543112 | 3.253536 |
| 42.27518 | 0.739174 | 2.6004 | 1.777066 | 0 | 0.982091 | 0.96213 | 0.317982 | 3.59377 |
| 4.303529 | 10.21124 | 0.061087 | 1.406116 | 0.315457 | 0.231446 | 4.47516 | 1.3956 | 4.443598 |
| 1.154472 | 0.449926 | 5.585745 | 0.667294 | 0.039192 | 0.278462 | 37.65861 | 0.851854 | 0.422267 |
| 0.200154 | 0.365936 | 0 | 22.35276 | 0.165126 | 0.025505 | 1.346948 | 1.619861 | 22.53811 |
| 9.610802 | 1.279957 | 4.943853 | 8.611779 | 0.042551 | 0.433774 | 0.321938 | 1.088552 | 5.043022 |
| 3.28218 | 1.551995 | 0.630782 | 8.202177 | 0.166875 | 0.288684 | 3.55099 | 0.943692 | 6.944308 |
| 18.56592 | 12.12406 | 0 | 0.569618 | 0.162423 | 1.170761 | 1.113682 | 0.05207 | 1.190579 |
| 36.66817 | 10.10174 | 0 | 1.017563 | 0.194175 | 0.494871 | 11.6153 | 0.116718 | 6.936578 |
| 1.569654 | 0.239625 | 0.069041 | 6.335345 | 0.044567 | 0.041302 | 84.67714 | 0.137158 | 38.31196 |
| 0.131033 | 3.100283 | 0 | 4.631736 | 0 | 0.058221 | 19.60669 | 0.514773 | 7.689716 |
| 19.17143 | 15.78594 | 0 | 1.76399 | 0.069949 | 0.237693 | 4.862314 | 0.161456 | 4.682541 |
| 11.48416 | 3.6093 | 0 | 0.81268 | 0.061326 | 0.11367 | 6.510449 | 0.271312 | 15.51152 |
| 6.371044 | 1.08386 | 0.139211 | 2.323906 | 0 | 0.305361 | 6.798951 | 0.639543 | 4.333126 |
| 0.257253 | 0.502947 | 0 | 2.953378 | 1.514191 | 0 | 0.818313 | 0 | 79.05085 |
| 0.030477 | 0.326781 | 0 | 5.605367 | 0.583003 | 0.025729 | 0.118152 | 1.233548 | 37.6155 |

| | | | | | | | | |
|---|---|---|---|---|---|---|---|---|
| 12.95963 | 10.39048 | 15.31345 | 1.471197 | 0.302424 | 3.523436 | 8.151494 | 2.019373 | 1.945903 |
| 3.672584 | 3.841829 | 0 | 48.98646 | 0.15064 | 0 | 2.172638 | 1.535711 | 61.46336 |
| 23.48623 | 0.33766 | 0.045953 | 1.429392 | 0.098876 | 0.122179 | 8.743386 | 0.152151 | 3.999358 |
| 5.743902 | 32.90261 | 0 | 0.208511 | 0 | 0.084423 | 2.035378 | 3.508825 | 5.032193 |
| 60.20192 | 0.079554 | 0 | 0.143892 | 0 | 0.092245 | 0.541279 | 0.794543 | 0.184599 |
| 9.45737 | 54.90023 | 0.021411 | 10.26749 | 0.069104 | 0.042695 | 67.80628 | 0.398767 | 3.991318 |
| 17.98149 | 32.41481 | 2.085413 | 10.03429 | 0.420671 | 0.064977 | 11.73658 | 1.375575 | 6.445783 |
| 27.48764 | 33.57759 | 0 | 2.306939 | 0 | 0.181948 | 0.342788 | 0.122006 | 0.760232 |
| 10.58329 | 29.37042 | 0.062103 | 3.241075 | 0.066814 | 0.29928 | 0.315945 | 0.051406 | 6.449386 |
| 3.379822 | 3.183162 | 0.093827 | 2.610492 | 0.201887 | 0.09355 | 6.682728 | 0.291248 | 10.57297 |
| 32.91353 | 26.84686 | 0.011658 | 0.688992 | 0.037626 | 0.058118 | 0.249096 | 0.340159 | 2.020361 |
| 0.044298 | 0.211103 | 0 | 2.269771 | 0.220105 | 0.067995 | 335.6649 | 0.812874 | 1.64061 |
| 0.203245 | 0.129142 | 0 | 16.56501 | 0.040395 | 0 | 0.210118 | 1.429663 | 30.97966 |
| 1.627977 | 0.048701 | 0 | 14.57761 | 0.100541 | 0.196707 | 0.618061 | 1.211911 | 13.06429 |
| 1.406537 | 0.107862 | 0.591359 | 62.35435 | 0.318105 | 0.065513 | 31.88992 | 0.265146 | 10.9377 |
| 12.88803 | 9.824279 | 0.117128 | 0.880476 | 0 | 0.253028 | 1.847223 | 0.387814 | 0.779009 |
| 0.161708 | 0.049947 | 0 | 10.87617 | 0.049101 | 0.030337 | 50.57069 | 0.236117 | 2.012082 |
| 8.532443 | 0.056474 | 0.401362 | 41.01203 | 0.129541 | 1.400619 | 2.205243 | 1.594703 | 0.960992 |
| 0.088445 | 2.301446 | 0.841596 | 2.64742 | 1.841546 | 0.071111 | 0.587805 | 23.16609 | 7.830908 |
| 1.911209 | 42.2503 | 1.845366 | 0.897815 | 0 | 1.585158 | 0.151654 | 2.02689 | 0.606924 |
| 14.62191 | 3.446335 | 0.153083 | 11.22975 | 0 | 0.457891 | 2.647894 | 0.538538 | 5.861564 |
| 2.303484 | 0.363768 | 0 | 12.72492 | 0.44175 | 1.182697 | 10.5839 | 0.424854 | 6.892308 |
| 1.184749 | 28.7205 | 0 | 3.949411 | 0.375382 | 0.289907 | 0.621281 | 0.252717 | 7.624912 |
| 0.134115 | 1.229554 | 0 | 1.796407 | 0.12566 | 0.051758 | 0.752673 | 1.07157 | 8.907709 |
| 0.961366 | 15.16909 | 0 | 3.797999 | 0.037003 | 0.102878 | 7.104104 | 0.270466 | 0.28104 |
| 1.138499 | 0.057312 | 0 | 3.061874 | 0.219104 | 0.09476 | 0 | 10.61204 | 15.52655 |
| 11.57747 | 6.284194 | 0.038613 | 1.561393 | 0 | 0.019249 | 10.84338 | 0.743112 | 2.872593 |
| 0.300139 | 16.48066 | 0.009774 | 5.715867 | 0.063094 | 0.194908 | 43.76857 | 2.075269 | 4.964742 |
| 0.769308 | 0.874772 | 0.018745 | 76.44534 | 0.151249 | 0.065413 | 0.085826 | 3.398032 | 16.32288 |
| 18.11625 | 1.419901 | 0.65943 | 9.152704 | 0 | 0.298856 | 1.463907 | 0.148867 | 2.204296 |
| 1.378068 | 20.86406 | 0.50101 | 3.677337 | 0.170214 | 0.657277 | 66.2028 | 0.163703 | 2.405178 |
| 44.50621 | 0.186822 | 0.108634 | 1.795947 | 0.038958 | 0.168487 | 0.1658 | 1.086563 | 3.240996 |
| 3.187255 | 8.368646 | 11.38827 | 6.44247 | 0.079818 | 0.0863 | 14.64459 | 6.010675 | 6.400563 |
| 4.928293 | 14.64142 | 0.871544 | 0.588751 | 0.170481 | 1.895934 | 5.038515 | 0.262336 | 3.432765 |
| 0.018058 | 0.193619 | 0 | 1.379214 | 0.246738 | 0.030489 | 2.963572 | 1.034628 | 30.21044 |
| 8.767237 | 14.38522 | 0 | 1.800169 | 0.197194 | 0.040611 | 2.548779 | 1.662608 | 3.349506 |
| 7.535854 | 16.47156 | 0 | 0.95825 | 0.326888 | 0.025245 | 18.97433 | 0.015719 | 1.212498 |
| 1.017484 | 22.25995 | 0 | 1.860461 | 0.383527 | 0.023696 | 0.362721 | 0.368858 | 2.072912 |
| 16.68056 | 0.434651 | 0.035643 | 9.614946 | 0.11504 | 0.035538 | 0.725326 | 0.560574 | 9.285973 |
| 15.16154 | 3.538122 | 0.5882 | 0.869076 | 0.189844 | 1.730065 | 1.436356 | 1.588464 | 0.905363 |
| 0.108241 | 0.142038 | 0 | 4.280333 | 0 | 0.015892 | 27.36715 | 1.959242 | 21.83472 |
| 5.77191 | 0.704209 | 0.029732 | 4.647238 | 0.14394 | 0.622522 | 0.226886 | 4.53142 | 6.949242 |
| 10.82817 | 2.875195 | 0.729146 | 4.970125 | 0.087161 | 0.376959 | 4.533785 | 0.553258 | 6.034913 |
| 0.372646 | 0.12108 | 0 | 12.11953 | 0.925786 | 0 | 0 | 0.516418 | 40.32432 |
| 0.161054 | 3.433551 | 0.287086 | 3.408287 | 0.463291 | 0.228991 | 0.832499 | 16.25439 | 13.02746 |
| 0.094953 | 0.420178 | 0 | 18.26801 | 0.074139 | 0 | 1.227053 | 1.155117 | 44.72009 |
| 5.452885 | 9.138653 | 0.944545 | 7.583012 | 0.058068 | 0.080722 | 8.663229 | 0.80419 | 13.318 |
| 10.08314 | 0.411057 | 0.030221 | 0.595367 | 0 | 0.120528 | 0.368995 | 0.225143 | 7.580531 |
| 2.573027 | 1.362415 | 0 | 2.648998 | 0 | 0 | 128.6218 | 0.999452 | 0.798059 |
| 0.446296 | 0.332589 | 0.055983 | 10.00328 | 0.602289 | 0 | 0.882903 | 0.463401 | 18.05302 |
| 33.29404 | 0.481865 | 0.788461 | 11.92216 | 0.077115 | 0.119111 | 5.870964 | 2.862772 | 4.985186 |
| 0.747966 | 0.533996 | 0 | 24.68718 | 0.367467 | 0.141897 | 0.086883 | 0.31807 | 32.51765 |
| 4.479721 | 2.814145 | 0.114651 | 3.724792 | 0 | 0 | 4.549546 | 0.972755 | 10.02185 |
| 0.230331 | 0.826618 | 0 | 20.98508 | 0.093251 | 0 | 0.793733 | 0.295959 | 26.66645 |
| 8.83826 | 2.124679 | 0.029608 | 35.21217 | 0.382245 | 0.059041 | 6.552344 | 1.617548 | 14.4315 |
| 0.172944 | 0.047777 | 0 | 7.261446 | 0.410973 | 0 | 0.388678 | 1.707495 | 44.77348 |
| 0.937009 | 20.31418 | 0 | 2.395461 | 0.163882 | 0.075939 | 81.60284 | 0.330988 | 6.730024 |
| 2.696036 | 21.12288 | 1.238111 | 2.567483 | 0.999011 | 0.154307 | 12.5188 | 1.441199 | 3.705551 |

| | | | | | | | | |
|---|---|---|---|---|---|---|---|---|
| 7.135635 | 7.95857 | 0.542765 | 1.303253 | 0.276599 | 0.085447 | 5.231865 | 1.418767 | 9.119689 |
| 0.924765 | 9.483331 | 0.079292 | 7.666498 | 0.255918 | 0.118587 | 18.39463 | 0.689162 | 15.1655 |
| 19.48668 | 1.596756 | 0.070632 | 2.944048 | 0 | 0.084508 | 4.096394 | 0.561273 | 3.511166 |
| 1.988595 | 0.154092 | 0.184804 | 36.44938 | 0 | 0.184258 | 0.689459 | 0.701123 | 4.050219 |
| 24.53728 | 1.108167 | 0 | 1.240028 | 0.094146 | 0.058167 | 31.02986 | 0.036218 | 2.095242 |
| 0.295388 | 9.814587 | 0.02779 | 7.131481 | 0.179386 | 0 | 6.446847 | 0.241534 | 27.40732 |
| 0.136821 | 0 | 0 | 8.680508 | 1.096784 | 0 | 0 | 0.038358 | 15.2514 |
| 1.482285 | 0.05573 | 0.029706 | 72.58157 | 0.095876 | 0 | 3.17363 | 2.120811 | 8.501169 |
| 14.52515 | 11.31725 | 1.17575 | 1.606973 | 0.17249 | 0.266427 | 10.03263 | 0.165892 | 1.614739 |
| 67.80002 | 25.06135 | 0 | 3.161167 | 0.093271 | 0.144066 | 4.983939 | 0.448518 | 4.99176 |
| 27.09715 | 0.49434 | 0.025707 | 7.783089 | 0 | 0.538252 | 10.16174 | 0.494739 | 4.894762 |
| 3.691192 | 1.838539 | 0 | 0.659098 | 0.341941 | 0.422528 | 6.467817 | 2.762441 | 5.073337 |
| 18.29819 | 19.22936 | 0.01043 | 1.292281 | 0.067324 | 0.083191 | 4.982334 | 1.243185 | 5.37494 |
| 33.70341 | 0.211004 | 0 | 3.479037 | 0.0605 | 0.046724 | 0.257483 | 0.791335 | 2.767718 |
| 3.856681 | 8.574265 | 0.524689 | 7.172558 | 0.445645 | 0.082601 | 0.505763 | 1.405806 | 4.486702 |
| 0.046872 | 0.399352 | 0 | 2.644789 | 0.093157 | 0.035972 | 8.270673 | 3.529987 | 9.078623 |
| 0.373049 | 0.958597 | 0 | 4.835534 | 0.059969 | 0 | 10.12368 | 0.06921 | 5.26433 |
| 1.060718 | 0.770262 | 0 | 13.71559 | 0.911027 | 0.10234 | 0.352474 | 0.382333 | 27.51633 |
| 2.307949 | 0.122206 | 0 | 43.22536 | 0 | 0.311744 | 0 | 1.43964 | 5.451308 |
| 31.1355 | 35.90191 | 0 | 0.357958 | 0 | 0.949558 | 3.997201 | 0.650372 | 1.805226 |
| 3.701383 | 0.033566 | 0.011928 | 35.859 | 0.615953 | 0.344882 | 0.983033 | 1.829015 | 8.214063 |
| 3.876232 | 0.05649 | 0.111178 | 2.82904 | 0.388732 | 0.240173 | 2.177578 | 2.133894 | 5.867915 |
| 6.293113 | 10.20812 | 0.276425 | 3.386554 | 0.093913 | 0.348137 | 9.614548 | 0.61418 | 6.079413 |
| 11.53229 | 1.78722 | 0.653237 | 3.012627 | 0.087848 | 0.135689 | 1.848574 | 0.050693 | 5.671276 |
| 12.35774 | 4.068509 | 0.31469 | 6.093076 | 0.04416 | 0.300119 | 17.33209 | 5.04551 | 19.33597 |
| 0.574629 | 2.451026 | 0.864969 | 2.20475 | 0 | 0.143736 | 204.3456 | 2.729684 | 2.999688 |
| 0.28507 | 2.391524 | 0.045257 | 3.824409 | 0.194759 | 0 | 0.092097 | 1.395458 | 25.47337 |
| 0.282895 | 0.328813 | 0.385585 | 6.65721 | 0.188559 | 0.198048 | 0.748985 | 0.67461 | 5.721814 |
| 1.204523 | 0.670349 | 0.009465 | 5.167139 | 0.091649 | 0.056624 | 0.462276 | 0.088143 | 12.53479 |
| 0.188353 | 6.85435 | 0 | 4.972773 | 0.224609 | 0.034693 | 9.966228 | 0.432036 | 6.598872 |
| 0.422973 | 0.854098 | 0.291365 | 8.056084 | 0.039183 | 0.060522 | 51.76899 | 1.45461 | 6.567895 |
| 12.6192 | 1.642738 | 0.753702 | 2.091607 | 0 | 0.265227 | 2.571292 | 0.825724 | 3.99339 |
| 17.65201 | 8.887384 | 2.59144 | 3.157591 | 0.799104 | 0.263316 | 0.604603 | 2.940943 | 2.681763 |
| 0.897297 | 4.205845 | 0.179646 | 0.954617 | 0.096635 | 0.059705 | 5.049466 | 5.037307 | 13.21969 |
| 0.526103 | 0.46728 | 0 | 5.46304 | 0.061838 | 0 | 23.45175 | 1.10619 | 9.436945 |
| 5.972846 | 29.15543 | 0.076966 | 10.55384 | 0 | 0.089528 | 17.97237 | 0.143345 | 4.226757 |
| 13.31651 | 0.839085 | 0.022087 | 3.125942 | 0.035643 | 0.066064 | 0.455073 | 1.288906 | 3.934724 |
| 2.223388 | 1.186479 | 0.411812 | 15.24962 | 0 | 0.351939 | 4.579189 | 0.51132 | 0.905522 |
| 4.313341 | 0.398722 | 0.044743 | 3.978126 | 0 | 0.111528 | 10.96021 | 1.083314 | 1.594191 |
| 16.69918 | 1.589264 | 2.655839 | 2.346766 | 0 | 0.326108 | 4.073364 | 0.243663 | 0.999412 |
| 0.130588 | 41.77653 | 0 | 1.7033 | 0 | 0.503971 | 97.70878 | 0.147094 | 4.1332 |
| 25.77216 | 0.717019 | 0 | 1.356483 | 0.094887 | 0.175875 | 3.230623 | 0.054755 | 1.960904 |
| 14.36381 | 18.7971 | 0.02876 | 1.252451 | 0.232062 | 0.55917 | 2.282517 | 0.107129 | 1.352637 |
| 5.540767 | 0.244486 | 0.012714 | 22.89139 | 0.246208 | 0.012676 | 8.266205 | 3.717606 | 4.145809 |
| 0.609792 | 3.141849 | 0.010701 | 3.361871 | 0 | 0.096024 | 22.1951 | 0.053146 | 6.685978 |
| 4.591796 | 7.937185 | 0 | 1.109669 | 0 | 0.533533 | 6.992996 | 0.103815 | 2.306985 |
| 0.986939 | 0.183542 | 1.516405 | 2.924802 | 0 | 0.065029 | 6.992879 | 1.538648 | 1.245579 |
| 3.754358 | 31.38389 | 0.961925 | 3.241473 | 0.19404 | 0.089914 | 54.32004 | 0.354575 | 6.94033 |
| 2.489643 | 0.045632 | 0.172965 | 0.431536 | 0.628032 | 0.075449 | 0.049497 | 3.322053 | 10.35953 |
| 29.78851 | 1.045846 | 0.284663 | 3.222096 | 0.076563 | 0.26017 | 1.484398 | 0.3829 | 1.649844 |
| 18.70434 | 2.379352 | 0 | 17.90232 | 0.103629 | 0.016007 | 6.688996 | 2.890295 | 6.772464 |
| 1.01915 | 0.088708 | 0 | 6.630808 | 0.228914 | 0.070716 | 6.963952 | 0.909989 | 9.946529 |
| 0.700133 | 1.21596 | 0.695565 | 2.802846 | 0.612262 | 0.283709 | 1.013332 | 0.942149 | 22.99851 |
| 10.56215 | 0.029038 | 0 | 1.392991 | 0 | 0.049383 | 0.434656 | 4.712204 | 3.910607 |
| 23.57723 | 0.873045 | 0.215818 | 3.440488 | 0.130605 | 0.080693 | 4.858432 | 0.460567 | 1.776286 |
| 2.007296 | 1.58739 | 2.589869 | 4.577248 | 0.094987 | 0.146717 | 1.347515 | 0.749104 | 5.637265 |
| 0.0893 | 1.063893 | 0 | 6.585417 | 0.488075 | 0.075388 | 1.961789 | 0.751051 | 22.4142 |
| 1.637421 | 3.940627 | 0.023801 | 18.76797 | 0.230458 | 0 | 0.435912 | 0.635377 | 23.54147 |

| | | | | | | | | |
|---|---|---|---|---|---|---|---|---|
| 2.946029 | 13.63921 | 0 | 2.581229 | 0.256092 | 0.079112 | 63.69829 | 0.529537 | 1.696251 |
| 23.80243 | 25.70556 | 0 | 4.020183 | 0 | 0.241615 | 12.32832 | 0.852509 | 12.27207 |
| 9.629549 | 0.427309 | 0.196988 | 4.276442 | 0.079473 | 1.166162 | 0.037581 | 4.089185 | 9.236547 |
| 0.396239 | 0.764301 | 0.047929 | 10.38626 | 0.154691 | 0 | 42.79253 | 0.178529 | 11.85819 |
| 12.10275 | 3.686396 | 0.06931 | 23.22671 | 0.089481 | 0.069106 | 3.575463 | 0.662647 | 8.819146 |
| 0.020921 | 0.033234 | 0.070858 | 0.318367 | 0.686087 | 0 | 0 | 0.058653 | 1.696565 |
| 9.498814 | 6.429507 | 0.246333 | 7.29199 | 0.357771 | 0.036841 | 10.95917 | 2.003348 | 7.681452 |
| 18.09791 | 2.521685 | 0.160493 | 7.627021 | 0 | 0.693415 | 1.041026 | 2.715096 | 9.660172 |
| 10.95731 | 2.13883 | 0.766589 | 9.558475 | 0.190322 | 0.254775 | 1.919973 | 0.073217 | 5.759634 |
| 1.892133 | 3.10536 | 0 | 36.26947 | 0.098025 | 0.015141 | 5.284295 | 0.461949 | 47.05962 |
| 20.67403 | 0.16433 | 0 | 3.54898 | 0.26096 | 0.080616 | 2.385762 | 0.217515 | 18.25292 |
| 2.677455 | 21.76522 | 0 | 3.626617 | 1.300787 | 0.028703 | 7.952493 | 0.214463 | 4.775671 |
| 32.4109 | 12.41418 | 0.015047 | 13.76874 | 0.048566 | 0.060012 | 1.332008 | 1.074293 | 5.730229 |
| 4.738197 | 4.117559 | 0 | 3.331202 | 0.105991 | 0.174627 | 0.735097 | 0.251443 | 3.269943 |
| 0.271153 | 4.581354 | 0 | 2.813318 | 0.538912 | 0 | 15.67256 | 0.984769 | 27.74726 |
| 36.24828 | 2.141325 | 0.910772 | 2.542451 | 0.075373 | 0.197915 | 43.59021 | 0.521928 | 1.031765 |
| 4.464626 | 4.631795 | 0 | 11.83503 | 0.086378 | 0.266838 | 1.674687 | 0.814126 | 5.355173 |
| 10.31097 | 1.53434 | 0 | 2.493021 | 0.037309 | 0.288138 | 0.511635 | 1.528577 | 7.334554 |
| 3.893523 | 19.22954 | 0.066938 | 2.82825 | 0.216044 | 0.433812 | 1.02162 | 0.602564 | 6.677967 |
| 60.04794 | 0.384274 | 0 | 9.428448 | 0.104383 | 0.31171 | 0.115174 | 2.201893 | 4.308133 |
| 21.20274 | 3.609249 | 0.074712 | 5.345104 | 0.080378 | 0.31038 | 8.304941 | 0.216451 | 5.338136 |
| 0.524063 | 23.78097 | 0.029582 | 1.257564 | 0.286431 | 0 | 8.939446 | 0.055095 | 1.399717 |
| 3.48604 | 16.37741 | 1.684111 | 1.746179 | 0 | 0.178632 | 5.304712 | 0.355923 | 1.634168 |
| 0.239717 | 18.10381 | 0.533524 | 5.868592 | 0.561511 | 0.138769 | 0.10621 | 1.706505 | 50.91208 |
| 1.882153 | 2.449787 | 0.056164 | 3.741501 | 0.090635 | 0.139994 | 3.257284 | 0.645045 | 7.412074 |
| 0.010867 | 7.889038 | 0.184029 | 8.268512 | 0.079195 | 0.024465 | 0.037449 | 2.460151 | 49.50403 |
| 8.971698 | 29.44163 | 0 | 1.122177 | 0.922712 | 0 | 16.64279 | 0.304258 | 2.630934 |
| 4.0292 | 10.38922 | 0.26577 | 3.66184 | 0.098975 | 0.163068 | 21.21727 | 2.544725 | 14.47501 |
| 82.2373 | 2.703304 | 0 | 2.763017 | 0.178872 | 0.230237 | 0.225557 | 0.223639 | 3.817611 |
| 18.61122 | 40.77164 | 0.078923 | 1.127463 | 0.028303 | 0.02623 | 6.504532 | 0.250428 | 16.25225 |
| 2.332678 | 0.513735 | 0 | 12.83712 | 0.488917 | 0.116181 | 0.160059 | 0.361705 | 8.648998 |
| 20.69856 | 1.25759 | 0.03886 | 3.324079 | 0.125421 | 0 | 1.067552 | 2.436598 | 10.1904 |
| 2.349107 | 4.409003 | 1.515442 | 1.239849 | 0.229272 | 1.038789 | 4.11985 | 0.352804 | 6.911356 |
| 0.231017 | 0.145623 | 0 | 42.41059 | 0.841761 | 0.049531 | 0.151637 | 0.316115 | 74.53105 |
| 1.032257 | 0.482963 | 2.258762 | 1.5843 | 0.160814 | 0.513344 | 16.64116 | 0.211373 | 1.410756 |
| 0.317497 | 0.817852 | 0.106563 | 12.13882 | 0.625335 | 0.038636 | 1.034969 | 3.548355 | 11.87365 |
| 0.083438 | 0.318928 | 0.927258 | 4.981132 | 0.028502 | 0.008805 | 2.143017 | 0.142544 | 4.878305 |
| 0.266708 | 2.249159 | 0.013284 | 14.72374 | 0.042874 | 0.052978 | 0.20274 | 2.416313 | 18.11413 |
| 29.5981 | 0.335362 | 0.01402 | 3.372559 | 0.045251 | 0.11183 | 0.898711 | 0.339454 | 24.90491 |
| 5.727482 | 0.546432 | 0 | 0.754994 | 0 | 0.058081 | 24.71595 | 0.940268 | 4.151063 |
| 1.738291 | 0.835704 | 0.018369 | 11.98006 | 0.296436 | 0.082417 | 4.667903 | 1.727691 | 7.2413 |
| 22.39933 | 20.45342 | 0.478093 | 0.840942 | 0.275547 | 1.327897 | 28.84824 | 0.519414 | 1.683971 |
| 0.668126 | 0.364492 | 0.251427 | 22.12337 | 0.590174 | 0.022789 | 7.988619 | 2.625149 | 6.893006 |
| 19.03766 | 0.076683 | 3.089215 | 8.172731 | 0.083319 | 0.197332 | 0.315197 | 2.233023 | 4.434617 |
| 6.371932 | 1.827684 | 0.148249 | 3.733014 | 0 | 0.042232 | 2.715122 | 0.420732 | 12.26649 |
| 58.16078 | 0.612734 | 0.098971 | 0.661889 | 0.063886 | 1.24335 | 0.362523 | 2.218083 | 1.060716 |
| 43.43279 | 12.63093 | 0.013939 | 0.426361 | 0 | 0.305757 | 12.55183 | 0.069229 | 0.53241 |
| 0.603282 | 37.81288 | 0.615429 | 5.181458 | 0.079453 | 0.466345 | 5.25997 | 0.275089 | 2.834806 |
| 21.99387 | 41.87494 | 0.113494 | 4.354042 | 0.122102 | 0.754392 | 0.692868 | 0.234863 | 5.348566 |
| 3.937402 | 0.348966 | 0.228934 | 6.49803 | 0.184723 | 0.114129 | 6.55131 | 0.461909 | 4.502593 |
| 1.10229 | 4.357098 | 0 | 2.565582 | 0.280217 | 0 | 46.37761 | 0.107799 | 13.90796 |
| 12.91479 | 0.333038 | 0.18259 | 1.703874 | 0 | 0.121367 | 2.337754 | 1.416936 | 5.476316 |
| 0.142813 | 0.214254 | 0 | 6.603227 | 0.260185 | 0 | 3.23992 | 0.483784 | 44.5623 |
| 3.803746 | 5.25318 | 0 | 1.923576 | 0.297527 | 0.206801 | 64.6133 | 0.929977 | 1.510871 |
| 8.136061 | 0.221902 | 0.028674 | 4.950155 | 0.138819 | 0.071473 | 9.343331 | 5.117851 | 7.257776 |
| 0.847055 | 7.126404 | 0.850029 | 9.070632 | 0.228625 | 0.176566 | 3.675774 | 0.395784 | 10.7012 |
| 2.263194 | 0.972334 | 0.218223 | 1.753562 | 0.08804 | 0.013599 | 6.140739 | 1.329365 | 24.37546 |
| 32.70451 | 0.158168 | 0.030657 | 3.703219 | 0 | 0.183401 | 1.450489 | 0.456783 | 11.74461 |

| | | | | | | | | |
|---|---|---|---|---|---|---|---|---|
| 10.28986 | 0.687424 | 1.070167 | 0.657322 | 0.037543 | 0.695874 | 2.698513 | 0.252752 | 2.181692 |
| 19.79966 | 20.49739 | 0.147978 | 0.69044 | 0.0796 | 1.53688 | 0.282308 | 1.232547 | 4.653786 |
| 0.638493 | 0.073572 | 0.089636 | 14.22562 | 0.144652 | 0.022343 | 1.350949 | 0.57039 | 6.291653 |
| 0.116755 | 0.261026 | 0.014646 | 1.085757 | 0.189077 | 0.043807 | 2.168192 | 2.318527 | 2.513097 |
| 10.31181 | 0.865433 | 0.100647 | 6.904919 | 0.541403 | 0.10035 | 4.966713 | 0.708145 | 14.84143 |
| 3.348263 | 2.287494 | 0.360383 | 18.03557 | 0.155087 | 0 | 0.330015 | 1.267814 | 11.23107 |
| 0.430241 | 2.386031 | 9.816659 | 1.139775 | 0.082509 | 0.229399 | 47.48324 | 0.095224 | 3.759986 |
| 2.403069 | 0.84335 | 0.520507 | 7.97269 | 0 | 0 | 22.60451 | 0.117505 | 14.94436 |
| 22.89307 | 47.66118 | 0.010222 | 3.08429 | 0.164962 | 0.030576 | 2.262192 | 0.11423 | 5.29132 |
| 7.371016 | 0.323387 | 0.641945 | 1.46063 | 0.076737 | 0.071117 | 2.267938 | 0.479712 | 3.171639 |
| 2.616933 | 0.82053 | 0.023172 | 3.417648 | 0.112181 | 0.046206 | 11.56437 | 0.525065 | 14.41832 |
| 0.351222 | 7.252891 | 0.055327 | 3.657101 | 0 | 0.165491 | 28.07681 | 0.841527 | 3.847978 |
| 52.10041 | 3.159133 | 0.028301 | 15.53762 | 0.091342 | 0.028217 | 0.215967 | 0.808205 | 44.99694 |
| 0.208947 | 0.727045 | 0.25274 | 8.385755 | 0.054382 | 0.050399 | 1.10578 | 5.596286 | 17.12649 |
| 0.220276 | 20.56332 | 0 | 14.22755 | 0.300984 | 0.026566 | 0.853968 | 0.347366 | 6.037762 |
| 5.963717 | 2.969992 | 1.341105 | 9.963459 | 0.075278 | 0.104646 | 0.676341 | 0.115837 | 6.628177 |
| 0.163257 | 0.089769 | 0 | 7.596287 | 0.205914 | 0.021204 | 28.43253 | 2.284039 | 3.734039 |
| 2.757272 | 2.663809 | 0.682636 | 12.98095 | 0.264388 | 0.136124 | 1.20855 | 1.644312 | 12.04826 |
| 0.373754 | 1.562636 | 0.040507 | 8.468253 | 0.294161 | 0.060581 | 1.76195 | 0.169746 | 6.252215 |
| 2.281514 | 1.768248 | 1.994101 | 6.493536 | 0 | 0.062132 | 97.34246 | 0.328836 | 1.56309 |
| 18.39578 | 11.6264 | 0.317434 | 2.19921 | 0 | 0.172635 | 6.166045 | 1.630294 | 5.034042 |
| 0.687826 | 1.081404 | 0.023893 | 2.979018 | 0.038558 | 0.023822 | 0.784017 | 0.459827 | 7.069217 |
| 18.35596 | 0.673789 | 0 | 1.49637 | 0.042538 | 0.144549 | 0.885069 | 1.767355 | 2.051179 |
| 4.474129 | 31.19546 | 19.54878 | 2.188839 | 0 | 1.297481 | 1.721293 | 0.897648 | 2.307996 |
| 13.97733 | 1.959857 | 0.084133 | 1.773762 | 0 | 0.447387 | 0.21401 | 1.253555 | 2.382143 |
| 39.8061 | 0.016084 | 0.171465 | 2.79714 | 0.036894 | 0.740821 | 0.331478 | 0.879973 | 5.271895 |
| 19.76193 | 1.046952 | 1.169824 | 2.085511 | 0.038527 | 0.190428 | 5.228701 | 0.414998 | 6.709737 |
| 31.16795 | 1.051924 | 0 | 1.755971 | 0 | 0.068455 | 18.18049 | 0.198911 | 1.813495 |
| 0.240628 | 12.49071 | 0 | 6.760044 | 0.267492 | 0.068861 | 4.110942 | 1.449237 | 21.08006 |
| 6.839998 | 36.68829 | 3.990646 | 0.806505 | 0 | 0.606945 | 1.806536 | 0.692849 | 1.754432 |
| 9.302044 | 0.134781 | 0.075623 | 1.293769 | 0 | 0.18096 | 4.801374 | 0.187792 | 4.518041 |
| 6.928608 | 16.68573 | 0.94147 | 3.459296 | 0.075966 | 0.539747 | 3.017472 | 0.3653 | 1.308233 |
| 9.864919 | 0.533544 | 0.185383 | 0.991654 | 0.217574 | 0.184835 | 5.658692 | 0.130782 | 0.187346 |
| 0.194083 | 30.13737 | 0.054027 | 2.394778 | 0.087187 | 0.062845 | 3.655602 | 2.543516 | 3.906417 |
| 0.13124 | 1.79883 | 0 | 22.74065 | 0.245932 | 0.050649 | 15.38971 | 0.630733 | 5.393652 |
| 10.14689 | 17.80404 | 0.29541 | 17.19517 | 0.105938 | 0.179995 | 0.976864 | 1.385653 | 5.239312 |
| 17.83891 | 34.87399 | 0.022018 | 7.419615 | 0.106596 | 0.043906 | 4.452595 | 0.881664 | 23.35306 |
| 28.55818 | 24.23914 | 0.032865 | 4.57476 | 0.053037 | 0.114688 | 71.87858 | 0.265242 | 1.826731 |
| 1.181495 | 28.4123 | 3.154502 | 2.771061 | 0.188542 | 0 | 16.98436 | 0.41706 | 2.481003 |
| 0.058089 | 5.51666 | 0.014053 | 1.879611 | 0.181422 | 0.014011 | 0 | 0.095966 | 3.580979 |
| 8.920014 | 0.167355 | 0 | 3.006009 | 0.479853 | 0.326119 | 16.92754 | 1.052218 | 4.525983 |
| 11.71602 | 1.081877 | 0 | 2.363219 | 0.177259 | 0.191656 | 3.269037 | 0.32391 | 9.564959 |
| 98.90383 | 1.366754 | 0.024284 | 0.629472 | 0.470263 | 1.525368 | 0.926565 | 0.919627 | 1.412059 |
| 3.077069 | 1.263114 | 0.030955 | 4.541583 | 0.466241 | 0.113167 | 40.07898 | 0.384349 | 4.199947 |
| 6.977328 | 40.02075 | 0.073275 | 3.532858 | 0.236497 | 0 | 24.2679 | 0.523138 | 6.725353 |
| 6.507797 | 7.114958 | 0.178469 | 1.017756 | 0.576015 | 0.266913 | 2.179065 | 0.94177 | 2.238306 |
| 0.440836 | 0.165517 | 0.760095 | 10.17504 | 0 | 0 | 7.291882 | 0.134823 | 10.29118 |
| 0.590939 | 0.682694 | 0.009576 | 4.244689 | 0.432704 | 0.066835 | 1.417688 | 2.021315 | 19.35818 |
| 11.35491 | 17.95692 | 0 | 5.415553 | 0 | 0.121959 | 1.045448 | 0.759382 | 6.471112 |
| 17.04481 | 24.64802 | 0.010716 | 8.138917 | 0.069174 | 0.245745 | 0.883186 | 6.978782 | 8.699313 |
| 3.850605 | 0.48866 | 0 | 1.247903 | 0 | 0.179103 | 5.921873 | 0 | 10.77303 |
| 2.293474 | 18.25817 | 0.785782 | 3.566166 | 0.083152 | 0.102749 | 142.0062 | 1.479474 | 8.393683 |
| 51.36606 | 31.56588 | 0 | 4.75456 | 0 | 0.140677 | 2.045737 | 0.394171 | 2.252169 |
| 34.15984 | 0.576546 | 0.078463 | 7.782971 | 0.042207 | 0.18254 | 0.299381 | 1.40451 | 19.52473 |
| 22.14782 | 4.611625 | 0.011023 | 0.142865 | 0 | 0.087923 | 2.456228 | 0.109492 | 1.036851 |
| 1.012469 | 0.972611 | 0.121983 | 10.39234 | 0.218725 | 0.067568 | 1.26184 | 1.691283 | 32.591 |
| 2.457098 | 4.420849 | 0.084917 | 6.141215 | 0.137035 | 0.084666 | 3.95284 | 0.711687 | 3.146585 |
| 21.05814 | 0.260616 | 0.065372 | 4.973444 | 0.052747 | 0.097768 | 3.067987 | 0.933431 | 3.605588 |

| | | | | | | | | |
|---|---|---|---|---|---|---|---|---|
| 6.950952 | 0.672948 | 0.02786 | 10.59428 | 0.08992 | 0.125 | 0.425207 | 1.072354 | 10.42683 |
| 0.033275 | 0.223176 | 0 | 6.647558 | 0.282907 | 0.09988 | 1.605354 | 1.554774 | 39.61165 |
| 2.423797 | 1.511168 | 17.83293 | 1.532374 | 0 | 0.712328 | 26.59696 | 7.018286 | 2.699262 |
| 20.17917 | 0.015293 | 0.008152 | 0.211303 | 0.289409 | 0.235701 | 1.418306 | 0.177125 | 0.96195 |
| 12.27605 | 3.917729 | 0 | 4.079802 | 0 | 0 | 2.0906 | 0.533576 | 6.568629 |
| 1.720007 | 1.300435 | 0.252061 | 4.312283 | 0.090393 | 0.027924 | 81.3 | 0.347741 | 5.7797 |
| 0.695188 | 0.762841 | 0.01549 | 6.504702 | 0.249974 | 0 | 0.496468 | 0.471209 | 2.878764 |
| 6.352917 | 1.257349 | 0.046623 | 7.649957 | 0.225714 | 0.418365 | 2.952995 | 1.374846 | 9.608146 |
| 57.40016 | 1.023508 | 0.473856 | 1.577396 | 0.040247 | 0.696252 | 2.359944 | 1.223159 | 4.506984 |
| 58.98388 | 2.04313 | 0 | 8.603943 | 0.345023 | 0.026646 | 0.530247 | 0.066365 | 29.90695 |
| 0.017548 | 0.069688 | 0 | 2.934805 | 0.239777 | 0.1037 | 0 | 0.387417 | 6.674619 |
| 18.0754 | 1.276994 | 0 | 5.727505 | 0.270387 | 0.052205 | 0.990909 | 0.260045 | 5.533994 |
| 13.41235 | 44.15574 | 0.274475 | 4.567679 | 0.177175 | 0.082099 | 7.582255 | 1.039431 | 5.335694 |
| 0.926578 | 5.265168 | 0.230748 | 12.7103 | 0.037237 | 0.011503 | 10.21302 | 2.313527 | 6.18917 |
| 14.3066 | 28.55977 | 0.016651 | 1.648772 | 0.053742 | 0.315434 | 5.565454 | 0.506522 | 4.10069 |
| 2.23872 | 27.94509 | 0.13118 | 10.26911 | 0.169355 | 0.444695 | 19.58051 | 0.586359 | 9.183329 |
| 36.28914 | 1.175952 | 0 | 3.470551 | 0 | 0.17499 | 1.320187 | 3.953631 | 2.136852 |
| 0.500869 | 0.811866 | 0.346197 | 3.661355 | 0.111736 | 0 | 205.96 | 0.17194 | 3.670884 |
| 4.777494 | 24.06494 | 0.0469 | 7.975147 | 0 | 0.420857 | 2.576894 | 0.029117 | 2.646952 |
| 15.06895 | 4.61365 | 0.02503 | 7.42242 | 0.080785 | 0.573991 | 0.229208 | 0.357398 | 3.824109 |
| 5.381243 | 15.24385 | 0 | 2.796333 | 0 | 0.16854 | 1.759026 | 1.54551 | 3.12749 |
| 33.97251 | 1.056234 | 0.059263 | 2.288918 | 0.095637 | 0.974957 | 0.633142 | 1.324499 | 1.36828 |
| 0.295053 | 0.202866 | 0.02983 | 0.60312 | 0.120346 | 0.007435 | 60.65326 | 3.736181 | 1.751549 |
| 0.016487 | 0.648209 | 0 | 0.40529 | 0 | 0 | 854.252 | 0.112667 | 1.504129 |
| 7.016141 | 1.30286 | 0.181163 | 1.768829 | 0.097452 | 0.391362 | 7.050632 | 0.881009 | 6.816297 |
| 0.673009 | 3.28849 | 0 | 23.81352 | 0.387198 | 0.026581 | 0 | 0.488242 | 22.11298 |
| 0.844419 | 1.24284 | 0 | 2.295193 | 0.156449 | 0.01611 | 99.67684 | 0.060186 | 4.126603 |
| 15.15034 | 36.87083 | 0.087223 | 5.180769 | 0.040216 | 0.732992 | 0.931848 | 0.464137 | 0.731649 |
| 5.209817 | 2.576627 | 0.023278 | 6.649495 | 0.187828 | 0.011605 | 1.438869 | 0.411867 | 5.633272 |
| 29.73125 | 0.24503 | 0 | 7.708598 | 0.129705 | 0.574313 | 1.451577 | 1.155959 | 2.153513 |
| 11.45166 | 43.72879 | 0 | 2.424397 | 0.105548 | 0.326058 | 5.889507 | 0.243626 | 3.728582 |
| 23.4195 | 0.046321 | 0 | 2.617651 | 0.039845 | 0.295411 | 12.37893 | 1.111301 | 3.969296 |
| 8.135016 | 0.764916 | 0 | 18.9724 | 0 | 0.086231 | 21.72296 | 1.288611 | 15.58004 |
| 2.064843 | 12.28905 | 1.11893 | 2.755399 | 0 | 0.154948 | 0.521808 | 0.038592 | 2.143975 |
| 2.883386 | 0.373161 | 0.192489 | 6.353386 | 0.041418 | 0.038384 | 1.684339 | 3.146836 | 8.720153 |
| 3.232596 | 7.382334 | 0.083575 | 2.079721 | 0.449568 | 0.305536 | 1.105466 | 0.345898 | 5.653777 |
| 3.62066 | 0.944874 | 0 | 20.26546 | 0.161543 | 0.199615 | 2.043424 | 2.72664 | 10.97104 |
| 0.512455 | 0.137759 | 0.213613 | 3.121567 | 0.129271 | 0.09318 | 9.678743 | 1.591375 | 4.954765 |
| 0.502252 | 49.56812 | 0.109745 | 5.547257 | 0.708413 | 0 | 1.214342 | 2.16318 | 7.366828 |
| 7.294224 | 0.65586 | 0 | 3.792048 | 0 | 0.250247 | 1.805872 | 0.912647 | 4.292487 |
| 12.51228 | 0.23525 | 0.036701 | 3.088668 | 0.197422 | 0.182962 | 1.960473 | 5.346753 | 2.141049 |
| 3.158359 | 0.605615 | 0.095144 | 4.615426 | 0.043869 | 0.027104 | 14.08541 | 0.658173 | 4.904788 |
| 39.71645 | 43.789 | 1.742573 | 1.402791 | 0.069866 | 8.072017 | 0.165189 | 0.503952 | 1.295741 |
| 21.20232 | 4.66682 | 0.047953 | 8.651266 | 0.619075 | 0.334677 | 0.475711 | 1.741532 | 6.273777 |
| 5.806584 | 2.554891 | 0.778187 | 1.59381 | 0.186046 | 0.59389 | 39.12026 | 0.506968 | 1.161097 |
| 46.19372 | 1.059501 | 11.12855 | 2.155423 | 0 | 1.160695 | 0.063454 | 0.421583 | 1.927734 |
| 110.8881 | 2.548501 | 0 | 4.190678 | 0.216511 | 0.267538 | 2.969098 | 0.91621 | 35.98594 |
| 15.02238 | 0.06217 | 0.055231 | 3.126737 | 0.035652 | 0.308379 | 2.242227 | 3.421956 | 4.94327 |
| 0.225653 | 0.306416 | 0.012327 | 12.03328 | 0.517201 | 0.061451 | 0.9971 | 0.635165 | 7.61041 |
| 29.96967 | 57.82558 | 0 | 0.451545 | 0.041957 | 0.103692 | 5.952185 | 0.056494 | 10.70136 |
| 67.43038 | 5.056762 | 0.178503 | 4.349416 | 0 | 0.854283 | 0.926278 | 1.573608 | 0.630915 |
| 0.205415 | 0.548779 | 0.031623 | 3.803477 | 0.102065 | 0.063059 | 214.919 | 0.569333 | 4.524937 |
| 50.91384 | 2.516252 | 0.030396 | 1.252778 | 0.098105 | 5.076322 | 0.788651 | 0.311362 | 0.753776 |
| 25.46058 | 2.032538 | 0 | 1.56758 | 0.197554 | 0.134262 | 0.822081 | 0.311596 | 0.872346 |
| 7.399837 | 0.026029 | 1.065529 | 1.582394 | 0.250763 | 0.18813 | 0.406558 | 0.068906 | 3.701552 |
| 23.36739 | 1.516831 | 0 | 7.520837 | 1.391733 | 0 | 0.164529 | 0.162532 | 30.86812 |
| 6.454549 | 26.21675 | 0 | 1.412115 | 0 | 0.23212 | 7.959053 | 0.260155 | 3.092331 |
| 11.5549 | 0.352979 | 0.027874 | 3.309164 | 0 | 0.083374 | 0.808287 | 0.46722 | 3.098585 |

| | | | | | | | | |
|---|---|---|---|---|---|---|---|---|
| 1.553509 | 2.75364 | 0.994551 | 5.472043 | 0.207093 | 0 | 0.783435 | 0.079669 | 25.38569 |
| 5.081912 | 17.91279 | 0 | 15.73107 | 0.125398 | 0.077476 | 0.94876 | 0.916571 | 8.505199 |
| 19.9349 | 0.383923 | 0.010106 | 8.120597 | 0.032617 | 0.39296 | 0.709485 | 1.072822 | 1.90691 |
| 30.64118 | 16.74265 | 0.02986 | 1.648624 | 0.337305 | 0.744285 | 0.843088 | 0.213179 | 1.310717 |
| 2.64528 | 0.11316 | 0.273439 | 19.75447 | 0.622966 | 0.064149 | 4.246935 | 1.248204 | 8.601002 |
| 1.222064 | 14.60971 | 0 | 5.687333 | 0.600905 | 0.057117 | 0.196721 | 10.10917 | 17.37391 |
| 3.514709 | 4.755491 | 1.302414 | 20.46677 | 0.1356 | 0.069815 | 0.213739 | 0.965056 | 4.630504 |
| 7.536002 | 1.285849 | 2.01372 | 2.540741 | 0.234916 | 0.580561 | 6.035676 | 0.94891 | 6.555916 |
| 7.865208 | 0.223247 | 0.237994 | 9.341807 | 0.1646 | 0.152544 | 0.415121 | 0.031661 | 6.182893 |
| 13.58927 | 3.252619 | 1.382528 | 3.294694 | 0.07197 | 0.111165 | 2.926833 | 0.775235 | 4.601769 |
| 5.657961 | 0.442229 | 1.885761 | 1.567084 | 0 | 0.193549 | 0.888821 | 1.256793 | 0.885319 |
| 1.816417 | 2.51078 | 3.194686 | 1.661808 | 0.104503 | 0.839357 | 1.087171 | 1.782302 | 1.119804 |
| 64.93743 | 13.375 | 0.447409 | 0.585395 | 0.068763 | 0.94528 | 0.373938 | 0.198399 | 1.724675 |
| 2.292081 | 0.374928 | 0.053292 | 2.643104 | 0.057334 | 0.017712 | 46.7411 | 3.286426 | 1.58993 |
| 13.67536 | 17.23345 | 0 | 7.518736 | 0.132603 | 0.109236 | 6.54218 | 1.810938 | 4.817038 |
| 15.69112 | 0.425762 | 0.155618 | 11.56363 | 0 | 0.077579 | 13.33998 | 2.721183 | 24.20419 |
| 26.9262 | 15.76825 | 0 | 1.368944 | 0.087861 | 0.13571 | 5.31806 | 0.219701 | 8.799198 |
| 3.6331 | 0.080155 | 0 | 25.38763 | 0.200576 | 0.061962 | 1.327863 | 3.144335 | 3.250484 |
| 2.564531 | 8.915693 | 2.866274 | 0.45029 | 0.120143 | 0.692802 | 7.044757 | 0.562331 | 1.37935 |
| 0.302651 | 22.73489 | 0.080924 | 3.76179 | 0 | 0 | 226.2243 | 0.100478 | 5.351402 |
| 0.727051 | 0.155138 | 0 | 8.821752 | 0.237239 | 0.036644 | 41.3961 | 0.182532 | 10.24539 |
| 0.421159 | 1.449521 | 0.029717 | 3.558776 | 0 | 0.029629 | 4.444716 | 0.903982 | 11.62141 |
| 15.57949 | 3.253209 | 2.137438 | 3.020134 | 0.067634 | 0.626802 | 33.90131 | 1.014732 | 4.945699 |
| 61.16435 | 0.476568 | 0 | 0.183952 | 0 | 0.289456 | 90.8071 | 0.180231 | 0.799921 |
| 0.106672 | 2.570762 | 0.154835 | 0.759894 | 1.232677 | 0.041167 | 26.15183 | 0.153798 | 15.43509 |
| 1.844976 | 0.393388 | 0 | 15.61035 | 0 | 0.041813 | 4.928425 | 2.577499 | 18.69566 |
| 22.9677 | 17.98406 | 0.040249 | 5.36958 | 0.173207 | 0.267535 | 3.726694 | 1.424277 | 9.430444 |
| 26.40136 | 17.13738 | 0.631216 | 4.42517 | 0.231508 | 0.944027 | 2.080012 | 0.543273 | 9.126898 |
| 18.81483 | 23.24358 | 0.013401 | 3.008302 | 0.086506 | 0.187064 | 18.1216 | 0.557421 | 4.522735 |
| 15.89582 | 0.497417 | 0 | 30.34121 | 0 | 0.111307 | 1.192674 | 0.502467 | 5.441349 |
| 0.015053 | 0.267813 | 0 | 17.47079 | 0.131639 | 0.030499 | 0.108935 | 0.721641 | 0.621972 |
| 8.506484 | 2.283807 | 0 | 5.469516 | 7.857954 | 0.404579 | 11.1475 | 1.259565 | 8.32768 |
| 21.21167 | 0.071554 | 0 | 28.65487 | 0.738591 | 0.091266 | 0.209557 | 12.84295 | 10.85402 |
| 41.75273 | 0.34757 | 0.049404 | 12.3622 | 0.265754 | 0.032839 | 1.809627 | 1.298393 | 14.32609 |
| 6.351045 | 0.794861 | 0 | 1.740293 | 0.105188 | 0.129979 | 1.691193 | 0.424893 | 3.065597 |
| 3.547614 | 2.680542 | 0 | 1.860439 | 0.171591 | 0.715604 | 0.040571 | 1.600769 | 28.71685 |
| 18.18752 | 46.21914 | 0.010966 | 0.216041 | 0.070789 | 0.349889 | 11.36454 | 0.29275 | 1.16907 |
| 28.38374 | 51.30517 | 0.853355 | 1.286054 | 0 | 0.949769 | 0.817791 | 0.406573 | 3.235649 |
| 7.555837 | 1.486505 | 1.161064 | 1.15505 | 0 | 0.281587 | 2.250969 | 2.405939 | 0.867621 |
| 21.03154 | 11.02043 | 0.948183 | 3.865177 | 0.041922 | 0.621622 | 0.317181 | 0.145146 | 6.501267 |
| 0.408185 | 0.715943 | 0 | 14.05043 | 0.371829 | 0 | 1.450585 | 1.180101 | 16.36972 |
| 1.25292 | 1.713135 | 0.026857 | 5.778276 | 0.086683 | 0.053556 | 17.0519 | 0.333469 | 4.440115 |
| 30.03248 | 20.69917 | 0 | 9.400489 | 0.241834 | 0.149414 | 4.574297 | 0.88382 | 8.799304 |
| 5.034523 | 11.53433 | 2.725763 | 1.946953 | 0.09775 | 2.264761 | 2.49607 | 0.225627 | 3.539432 |
| 5.102332 | 0.026202 | 0.037243 | 1.283974 | 1.442442 | 0.185666 | 0.255786 | 1.005771 | 14.46926 |
| 3.978083 | 24.47644 | 0 | 0.838793 | 0 | 0.087992 | 12.07741 | 0.730512 | 12.4938 |
| 9.869518 | 4.66736 | 0.038974 | 2.79504 | 0.125789 | 0.129529 | 11.85688 | 0.129043 | 11.85707 |
| 26.73165 | 0.859967 | 0.012822 | 0.857498 | 0.165534 | 0.063921 | 9.393207 | 0.159202 | 0.913691 |
| 1.823522 | 2.293887 | 0.428393 | 1.573141 | 0 | 0.533909 | 1.634554 | 0.687046 | 1.70952 |
| 3.62232 | 0.334477 | 0.568284 | 6.123351 | 0.035964 | 0.255528 | 1.649622 | 10.50791 | 10.78616 |
| 6.68932 | 0.464306 | 0.113137 | 2.8007 | 0.547731 | 0.310208 | 0.604353 | 0.386306 | 20.89701 |
| 0.112141 | 2.335016 | 0.472189 | 3.315383 | 0.099392 | 0.133051 | 307.3475 | 0.248534 | 1.714586 |
| 1.053752 | 10.96469 | 0.163711 | 3.972022 | 0 | 0 | 1.149352 | 0.548826 | 5.263697 |
| 20.58575 | 22.47447 | 0.882542 | 4.51295 | 0 | 0.319977 | 5.632716 | 2.017256 | 6.494789 |
| 1.042335 | 17.83933 | 0.74021 | 3.483225 | 0 | 0.51094 | 10.51512 | 0.919069 | 3.862713 |
| 4.847446 | 0.422569 | 0.100107 | 30.42969 | 0.646198 | 0 | 2.648282 | 0.870074 | 38.5595 |
| 2.666813 | 0.656316 | 0.063606 | 4.633022 | 0.102646 | 0.285383 | 1.650313 | 0.45411 | 1.885547 |
| 8.053484 | 1.224969 | 0.022711 | 3.755911 | 0.03665 | 0.16983 | 1.19584 | 2.678881 | 2.395203 |

| | | | | | | | | |
|---|---|---|---|---|---|---|---|---|
| 11.32052 | 0.418636 | 0.799324 | 3.135562 | 0.085995 | 0.132827 | 0.711634 | 7.087866 | 24.59135 |
| 10.18612 | 29.91475 | 0.431685 | 0.242447 | 0 | 1.315143 | 0.201314 | 2.382199 | 0.765626 |
| 72.55044 | 10.02301 | 0.089743 | 1.733072 | 0.108619 | 0.771751 | 5.18767 | 0.564106 | 4.866661 |
| 9.688123 | 27.05549 | 1.495462 | 6.122502 | 0.03549 | 0.30698 | 0.453125 | 2.682825 | 3.153106 |
| 0.462333 | 1.327596 | 0 | 8.898366 | 0.388757 | 0 | 0.643417 | 1.15905 | 6.729262 |
| 49.019 | 0.176381 | 0 | 5.432945 | 0 | 0.074991 | 1.721872 | 0.964997 | 10.59067 |
| 0.247843 | 12.32394 | 0.127183 | 4.668207 | 0 | 1.03982 | 0.465862 | 1.010653 | 3.958726 |
| 0.480516 | 0.183428 | 0 | 1.138024 | 0.447896 | 0.012579 | 0.077018 | 0.156642 | 9.996844 |
| 11.68269 | 3.159384 | 0.106174 | 1.369972 | 0 | 0.25877 | 12.83756 | 2.717145 | 3.907382 |
| 13.60228 | 0.192706 | 0.862828 | 10.31484 | 0.033152 | 0.93197 | 0.485986 | 2.404083 | 1.949949 |
| 4.28397 | 33.62369 | 0.07447 | 6.884929 | 0.440647 | 0.012375 | 3.068739 | 4.969934 | 12.32565 |
| 0.186975 | 0.95248 | 0 | 6.316906 | 0 | 0 | 75.65286 | 0.271129 | 4.917148 |
| 31.9901 | 35.45197 | 0.021566 | 5.377679 | 0.069604 | 0.494544 | 1.316555 | 0.240989 | 7.056798 |
| 9.585887 | 0.054491 | 0 | 10.16567 | 0.124992 | 0.025742 | 2.994684 | 0.448788 | 8.382003 |
| 0.228361 | 20.34307 | 0.216559 | 7.345671 | 0.09985 | 0 | 1.180416 | 0.268887 | 9.453155 |
| 6.006313 | 0.180872 | 0.032137 | 20.29249 | 0.207444 | 0.032042 | 3.825709 | 0.359117 | 29.95379 |
| 28.29754 | 5.111418 | 0 | 6.234155 | 0.034316 | 0.243821 | 0.665316 | 2.039617 | 4.897476 |
| 0.300075 | 4.457494 | 0.027468 | 1.858329 | 0.177307 | 0.013693 | 27.92001 | 0.451891 | 7.563194 |
| 3.736361 | 35.116 | 0.150828 | 6.114732 | 0.146041 | 3.067809 | 1.081927 | 1.825915 | 5.270807 |
| 15.92572 | 2.396649 | 0 | 2.246331 | 0 | 0.119177 | 0.456074 | 0.500893 | 5.587603 |
| 0.196725 | 2.273262 | 0.326607 | 18.66604 | 0.084331 | 0.013026 | 6.639671 | 26.02663 | 4.55796 |
| 13.85315 | 7.52316 | 0.07513 | 18.15038 | 0 | 0.374539 | 0.802652 | 2.658581 | 12.09941 |
| 0.17184 | 0.363957 | 0.032333 | 0.653733 | 0.104356 | 0.064475 | 101.113 | 0.120438 | 2.064429 |
| 6.162983 | 0.657529 | 0.259616 | 0.68911 | 0.033517 | 0.186371 | 39.00494 | 0.051576 | 1.101128 |
| 0.553914 | 0.374282 | 0 | 12.15727 | 0.090372 | 0.083753 | 0.833327 | 1.234198 | 11.72436 |
| 10.14647 | 17.69868 | 1.685484 | 0.166968 | 0.069299 | 1.49854 | 24.16764 | 0.37323 | 1.1261 |
| 0.075587 | 0.10506 | 0 | 2.60458 | 0.103281 | 0.063811 | 65.73739 | 0.317858 | 21.21601 |
| 0.725743 | 1.305873 | 0.348033 | 16.14847 | 0.210617 | 0.238566 | 0.597572 | 2.849352 | 13.28079 |
| 6.107953 | 30.51471 | 0.012197 | 0.246614 | 0 | 0.170259 | 0.260622 | 0.053006 | 2.663167 |
| 83.92732 | 16.61068 | 0 | 1.004745 | 0.297865 | 0.460081 | 0.492985 | 0.644561 | 1.512587 |
| 0.520046 | 0.791671 | 0 | 7.862937 | 0 | 0 | 0.33602 | 0.48598 | 69.07983 |
| 31.80407 | 24.54641 | 0.316077 | 0.53471 | 0.107384 | 2.338694 | 1.218701 | 1.776358 | 0.758687 |
| 0.275002 | 2.732913 | 0 | 31.72732 | 0.03666 | 0.056624 | 0.624076 | 8.17268 | 19.91118 |
| 12.47312 | 0.399848 | 0.044869 | 1.791139 | 0.181022 | 0.100658 | 0.428004 | 0.041784 | 1.330111 |
| 11.17225 | 1.528062 | 4.163744 | 7.218693 | 0.130896 | 0.242617 | 4.827991 | 0.511949 | 6.774172 |
| 1.743771 | 1.12328 | 0 | 6.923121 | 0.202351 | 0.075012 | 15.1185 | 2.747908 | 3.767115 |
| 2.422234 | 0.524107 | 0.027255 | 5.242124 | 0 | 0.081523 | 71.83812 | 0.693733 | 4.490319 |
| 8.25095 | 3.166235 | 1.516475 | 2.681896 | 0.157886 | 0.45116 | 4.143657 | 1.374217 | 1.812679 |
| 26.87584 | 0.598167 | 0.214441 | 3.551651 | 0.145708 | 0.540145 | 0.585666 | 1.807742 | 8.866184 |
| 8.532635 | 1.215404 | 0 | 5.832909 | 0.317611 | 0.130821 | 11.66477 | 1.344034 | 10.69629 |
| 2.010116 | 3.594706 | 0.042817 | 6.326343 | 0 | 0.170764 | 13.20044 | 5.050526 | 6.468442 |
| 0.515325 | 0.725921 | 0.098792 | 12.51391 | 0.42514 | 0.032833 | 20.05355 | 0.47021 | 9.668127 |
| 1.16589 | 0.522921 | 0 | 15.02017 | 0.249893 | 0.015439 | 3.332341 | 0.394149 | 37.29699 |
| 10.92525 | 0.333948 | 0.689411 | 5.495904 | 0.109431 | 0.540885 | 0.500223 | 6.455046 | 6.217412 |
| 50.52728 | 1.880214 | 1.066082 | 3.695234 | 0.028437 | 0.043923 | 0.497536 | 0.300837 | 0.9945 |
| 20.07754 | 1.599519 | 0.023043 | 7.036236 | 0.223115 | 0.091899 | 20.15157 | 0.443468 | 18.90305 |
| 10.83495 | 15.00143 | 0 | 5.279085 | 0 | 0.035499 | 2.59022 | 2.01145 | 3.037832 |
| 4.056136 | 19.85558 | 0.73396 | 2.369183 | 0.044696 | 0.207111 | 12.15295 | 0.395474 | 9.655106 |
| 5.519061 | 13.20155 | 4.014113 | 2.269071 | 0.156093 | 1.084949 | 18.19473 | 0.630513 | 2.688129 |
| 25.29906 | 32.42535 | 0 | 1.083276 | 0.082749 | 0.613508 | 0.195651 | 0.30242 | 5.130206 |
| 0.100828 | 1.121154 | 0 | 6.019267 | 1.102167 | 0 | 0.521188 | 1.06001 | 50.32354 |
| 2.471822 | 18.72114 | 5.757321 | 1.0517 | 0.046109 | 0.797662 | 22.28351 | 2.669593 | 3.257688 |
| 1.863703 | 37.23523 | 0 | 12.04884 | 0.08524 | 0.052665 | 0.161232 | 0.459088 | 9.816487 |
| 20.63665 | 26.53088 | 0.079257 | 10.36615 | 0.073087 | 0.079023 | 1.520683 | 1.209011 | 8.31359 |
| 12.00254 | 0.893265 | 0.321546 | 7.764281 | 0.039915 | 0.382249 | 0.41525 | 0.161232 | 3.912876 |
| 43.59861 | 7.129568 | 0 | 4.107986 | 0 | 0.537451 | 2.879446 | 0.379266 | 7.651682 |
| 0.157183 | 1.611596 | 0 | 4.340517 | 0.078099 | 0.024126 | 3.656199 | 0.525786 | 16.29151 |
| 3.696095 | 4.90995 | 0 | 2.23953 | 0.30531 | 0.062877 | 9.769261 | 0.587263 | 5.248863 |

| | | | | | | | | |
|---|---|---|---|---|---|---|---|---|
| 5.212829 | 9.079565 | 0.015487 | 6.431096 | 0.049984 | 0.185294 | 10.51821 | 1.990206 | 9.614492 |
| 0.788015 | 25.82751 | 0.255532 | 21.62281 | 0.091637 | 0.268931 | 7.994953 | 2.09755 | 3.96554 |
| 16.99317 | 22.87718 | 0 | 4.648254 | 0 | 0.157387 | 1.108227 | 0.803583 | 13.89424 |
| 10.95331 | 40.9866 | 2.28252 | 0.806219 | 0.080955 | 0.425145 | 0.153127 | 0.420436 | 0.886541 |

| TDGF1 | TNFSF9 | ADRB1 | CSF3R | GALR2 | HTR3A | OPRL1 | SSTR2 | TACR1 |
|---|---|---|---|---|---|---|---|---|
| 0.664713 | 0.338686 | 0.401301 | 0.49061 | 0.413425 | 0.308229 | 0.303341 | 0.779112 | 0.057948 |
| 1.084565 | 1.028478 | 0.870313 | 0.467263 | 0.047079 | 2.023147 | 0.729792 | 1.084981 | 0.217762 |
| 0.208287 | 0.276667 | 1.345517 | 0.480927 | 1.069449 | 0.476383 | 0.207676 | 0.432178 | 0.237211 |
| 0.486128 | 0.383334 | 0.701644 | 0.667721 | 0.106347 | 0.4448 | 0.318807 | 0.225382 | 0.20496 |
| 0.431629 | 0.402243 | 1.029277 | 0.617312 | 0 | 0.185851 | 0.428228 | 0.787336 | 0.514404 |
| 0.80728 | 0.208334 | 0.694761 | 0.754468 | 0.07266 | 0.065547 | 0.40492 | 0.366037 | 0.099581 |
| 0.882795 | 0.777418 | 0.447567 | 0.369386 | 0.282127 | 0.681502 | 0.285864 | 0.300739 | 0.270221 |
| 0.853896 | 0.37106 | 1.072435 | 0.408366 | 0.075491 | 0.244104 | 0.460073 | 1.350717 | 0.102537 |
| 0.549138 | 0.690783 | 0.442792 | 1.319782 | 0.12441 | 0.482938 | 0.40377 | 1.056571 | 0.735302 |
| 1.558044 | 0.643334 | 0.625745 | 0.582446 | 0.261767 | 0.515224 | 0.578484 | 0.562722 | 0.201799 |
| 0.787933 | 0.437749 | 0.873538 | 2.605244 | 0.430927 | 0.746392 | 0.867846 | 0.269488 | 0.695419 |
| 0.620905 | 0.816396 | 0.490256 | 0.605327 | 0.231085 | 0.518795 | 0.466272 | 0.441569 | 1.2396 |
| 0.493071 | 0.220561 | 1.153515 | 0.434003 | 0.082841 | 0.708257 | 0.589013 | 0.568428 | 0.399144 |
| 0.463203 | 0.270385 | 0.340506 | 0.449766 | 0.034742 | 0.384647 | 0.240347 | 0.576363 | 0.285688 |
| 0.673629 | 0.475219 | 0.876581 | 0.380514 | 0.072511 | 0.326217 | 0.394139 | 1.009492 | 0.23957 |
| 0.70126 | 0.231735 | 1.089429 | 0.676454 | 0.03143 | 0.502635 | 0.742903 | 0.335782 | 0.205955 |
| 0.982267 | 0.330131 | 0.513768 | 0.70374 | 0.050373 | 0.082622 | 0.554985 | 0.980049 | 0.094925 |
| 0.492708 | 0.627613 | 0.924006 | 2.175179 | 0.626819 | 1.482199 | 0.468431 | 0.610977 | 0.612976 |
| 1.061896 | 0.4149 | 0.740645 | 0.554409 | 0.387291 | 0.498418 | 0.343497 | 0.382966 | 0.122489 |
| 0.491213 | 0.492983 | 0.528571 | 0.32611 | 0.069973 | 1.060487 | 0.306582 | 0.459472 | 0.467509 |
| 0.686044 | 0.268952 | 0.464599 | 0.860651 | 0.078793 | 0.368324 | 0.519852 | 0.818959 | 0.070866 |
| 0.786214 | 0.708805 | 1.139505 | 0.385693 | 0.326498 | 0.449842 | 0.23842 | 0.260899 | 0.381747 |
| 0.408028 | 0.564825 | 0.502685 | 0.845152 | 0.327497 | 0.424077 | 0.337857 | 0.440155 | 0.341058 |
| 0.395334 | 0.575941 | 0.866434 | 0.983452 | 0.140607 | 0.392063 | 0.450334 | 0.531931 | 0.128469 |
| 0.528451 | 0.511414 | 0.605209 | 0.322705 | 0.052023 | 0.494902 | 0.333233 | 0.581382 | 0.539186 |
| 0.59028 | 0.478083 | 0.436707 | 1.36728 | 0.152239 | 0.243462 | 0.234043 | 0.960872 | 0.39121 |
| 1.437469 | 0.28853 | 0.717852 | 1.193874 | 0.203906 | 0.228033 | 0.299223 | 0.412175 | 0.038108 |
| 0.237891 | 0.34789 | 0.42994 | 0.495185 | 0.599521 | 0.639171 | 0.350424 | 0.318944 | 0.22467 |
| 0.968291 | 0.513584 | 1.789537 | 0.21185 | 0.129071 | 0.308484 | 0.212599 | 0.416399 | 0.028429 |
| 0.731266 | 0.320072 | 0.962265 | 0.743367 | 0.734048 | 0.238857 | 0.318522 | 1.336843 | 0.222095 |
| 0.35513 | 0.36532 | 0.717598 | 0.429455 | 0.054383 | 0.396043 | 0.221551 | 1.148754 | 0.176081 |
| 0.755522 | 0.242484 | 1.0445 | 0.483547 | 0.14095 | 0.513236 | 0.390036 | 0.367273 | 0.351333 |
| 0.448247 | 0.605936 | 0.827797 | 1.161557 | 0.067241 | 0.504574 | 0.820515 | 0.259895 | 0.008639 |
| 1.396184 | 1.147621 | 0.398838 | 0.583287 | 0.04722 | 0.294315 | 0.308523 | 1.435495 | 0.279088 |
| 0.12211 | 0.356509 | 0.656682 | 0.358713 | 0.149594 | 0.220829 | 0.214296 | 0.852829 | 0.132797 |
| 1.622174 | 1.010495 | 1.061498 | 0.405864 | 0.082232 | 0.131507 | 0.331849 | 2.731985 | 0.116222 |
| 0.065509 | 0.419126 | 0.762578 | 0.314119 | 2.347419 | 1.59934 | 0.468446 | 0.122856 | 1.778719 |
| 1.059234 | 0.437727 | 1.094811 | 1.145665 | 1.049562 | 1.154972 | 0.586737 | 0.914931 | 0.691429 |
| 0.603804 | 0.509023 | 1.011432 | 0.734962 | 0.133147 | 0.131033 | 0.341152 | 0.452309 | 0.053223 |
| 0.439667 | 0.340967 | 0.643781 | 0.828033 | 1.93415 | 2.252817 | 0.602215 | 0.216904 | 1.119867 |
| 0.201087 | 0.302717 | 0.68142 | 1.287045 | 0.809422 | 0.848524 | 0.331376 | 0.50741 | 0.501907 |
| 0.520818 | 0.397248 | 0.750404 | 0.885877 | 0.025522 | 0.326514 | 0.495345 | 0.727086 | 0.265611 |
| 15.35127 | 5.49086 | 0.167265 | 0.843264 | 0.036827 | 0.072485 | 0.226463 | 0.108756 | 0.132489 |
| 16.18593 | 1.548901 | 0.336173 | 0.187115 | 0.046877 | 0 | 0.13212 | 0.040716 | 0.012046 |
| 0.008661 | 22.21837 | 0.563102 | 0.700567 | 0.827648 | 0.125309 | 0.56278 | 0.132714 | 0.03272 |
| 3.142701 | 2.778788 | 0.20715 | 2.257704 | 0.111416 | 0.006092 | 0.180799 | 0.070966 | 0.066805 |
| 6.701837 | 1.022719 | 0.209706 | 1.92738 | 0 | 0.016603 | 0.116709 | 0.644735 | 1.335238 |
| 5.308188 | 0.984027 | 0.217828 | 0.130702 | 0 | 0.417815 | 0.063676 | 0.07555 | 0.074506 |
| 2.953906 | 0.649765 | 0.3081 | 3.144563 | 0.24786 | 0.19514 | 0.647774 | 0.068891 | 0.012739 |
| 2.679645 | 4.850642 | 2.705644 | 0.120585 | 0.060419 | 0.08919 | 0.23221 | 0.027988 | 0.015526 |
| 4.472581 | 13.81261 | 1.1291 | 0.043731 | 0.046998 | 0 | 0.018063 | 0.022452 | 0 |
| 0.411779 | 22.34573 | 0.361768 | 8.239087 | 0.792723 | 0.124113 | 0.387762 | 0.181523 | 0.14815 |
| 1.79159 | 28.64684 | 0.438704 | 0.180596 | 0.111602 | 0.012203 | 0.042892 | 0.040928 | 0.044611 |
| 0.217472 | 23.03236 | 1.416088 | 5.949523 | 0.047015 | 0 | 0.180695 | 0.579869 | 2.875395 |
| 9.90062 | 0.30715 | 0.044813 | 0.82514 | 0.018747 | 0.092245 | 0.230557 | 0.081413 | 0.038538 |
| 7.095989 | 3.591879 | 0.245011 | 1.488133 | 0.31318 | 0 | 0.2845 | 0.143428 | 0.073162 |

| | | | | | | | | |
|---|---|---|---|---|---|---|---|---|
| 0.352714 | 5.438498 | 0.46951 | 1.048853 | 0.039282 | 0.019329 | 0.150973 | 0.184243 | 0.010094 |
| 7.180067 | 0.666742 | 0.149657 | 2.254149 | 0.031303 | 0 | 0.144369 | 0.092442 | 0.024132 |
| 8.465794 | 4.669089 | 0.610912 | 0.34463 | 0.101414 | 0.019961 | 0.202678 | 0.059898 | 0.020848 |
| 4.190955 | 1.416473 | 0.118093 | 1.81013 | 0.264651 | 0.121543 | 0.467886 | 0.098077 | 0.009068 |
| 14.8229 | 5.318475 | 0.098145 | 0.149488 | 0 | 0.025253 | 0.059173 | 0.031203 | 0.019782 |
| 0.155079 | 35.05735 | 0.522269 | 5.84894 | 0.33247 | 0 | 1.168268 | 0.189765 | 0.04882 |
| 14.17776 | 3.622227 | 0.17887 | 0.583413 | 0.204072 | 0.050208 | 0.078432 | 0.064992 | 0.02622 |
| 7.406526 | 4.267006 | 0.311276 | 0.136743 | 0.055411 | 0 | 0.133102 | 0.036096 | 0.032038 |
| 0.913745 | 26.45188 | 0.22323 | 0.540528 | 0.287334 | 0.114877 | 0.227766 | 0.065512 | 0.110755 |
| 2.68636 | 3.519831 | 0.245844 | 2.329929 | 0.217114 | 0.061851 | 0.241549 | 0.093297 | 0.008809 |
| 0.889881 | 19.73966 | 0.067616 | 0.497773 | 0.017678 | 0.008699 | 0.054355 | 0.064491 | 0.036343 |
| 0.209975 | 23.29636 | 0.590314 | 2.603723 | 0.038585 | 0.075945 | 0.118637 | 0.321733 | 0.694068 |
| 5.327874 | 26.39854 | 0.092142 | 0.605039 | 0.308364 | 0 | 0.162958 | 0.127222 | 0.009905 |
| 2.050414 | 9.746924 | 0.024976 | 0.137373 | 0.039181 | 0.713334 | 0.055214 | 0.063524 | 0.003356 |
| 16.58781 | 1.454029 | 0.110683 | 0.680581 | 0.154339 | 0.056958 | 0.430053 | 0.241297 | 0.089236 |
| 3.647372 | 1.403198 | 0.356044 | 1.982418 | 0 | 0 | 1.159189 | 0.27814 | 0.25835 |
| 6.793328 | 0.27891 | 0.072343 | 0.471358 | 0.340459 | 0 | 0.058155 | 0.072285 | 0.019442 |
| 0.052652 | 29.31282 | 0.883097 | 1.19847 | 0.087958 | 0.259686 | 0.480036 | 0.055006 | 0.013562 |
| 1.984935 | 12.41224 | 0.078908 | 2.470515 | 0.231066 | 0.008121 | 0.291792 | 0.183494 | 0.042412 |
| 9.255692 | 2.755324 | 0 | 0.434035 | 0.170296 | 0 | 0.654506 | 0.066561 | 0.021881 |
| 7.188519 | 0.452079 | 0.049468 | 2.556218 | 0.20694 | 0.118799 | 0.331392 | 0.155776 | 0.053178 |
| 0.476552 | 0.91306 | 0.228368 | 1.584581 | 0.119416 | 0.528842 | 1.682837 | 0.255845 | 0.040915 |
| 1.093901 | 8.122177 | 1.449713 | 6.963395 | 0.61327 | 0.050295 | 0.301173 | 0.118371 | 0.043776 |
| 2.883894 | 0.198132 | 0.965845 | 0.089786 | 0.085361 | 0 | 0.04921 | 0.022242 | 0.014623 |
| 4.288071 | 32.43212 | 0.083873 | 1.599232 | 0.292386 | 0.014387 | 0.224748 | 0.06095 | 0.060108 |
| 8.512 | 1.073033 | 0.101943 | 0.226747 | 0.274149 | 0.014989 | 0.409752 | 0.111121 | 0.007828 |
| 8.516464 | 6.164862 | 0.159194 | 2.230763 | 0.066595 | 0.032769 | 0.230354 | 0.057843 | 0.034226 |
| 1.266303 | 5.036397 | 0.915646 | 18.04989 | 0.019152 | 0.28272 | 0.426925 | 0.585547 | 0.275605 |
| 2.698363 | 5.840971 | 0.046483 | 1.342581 | 0.486131 | 0 | 0.124558 | 0.219564 | 0.049969 |
| 8.117988 | 2.664359 | 0.058676 | 0.089371 | 0.294549 | 0.018117 | 0.07547 | 0.044771 | 0.006308 |
| 8.28188 | 0.563262 | 1.618302 | 0.333787 | 0.026445 | 0 | 0.203271 | 0.055125 | 0.013591 |
| 6.014059 | 3.531941 | 0.223844 | 1.859408 | 0.078034 | 0.191987 | 0.352393 | 0.152499 | 0.005013 |
| 0.808118 | 12.02584 | 0.290972 | 3.872486 | 0.097377 | 0.191663 | 0.505243 | 0.207218 | 0.068814 |
| 4.116245 | 3.991245 | 2.119431 | 2.279967 | 0.087784 | 0 | 0.12933 | 0.08133 | 0.022558 |
| 10.64678 | 0.77211 | 0.09012 | 0.172852 | 0.172791 | 0.015459 | 0.138856 | 0.060032 | 0.14128 |
| 11.43657 | 2.412447 | 0.065315 | 0.218617 | 0.136615 | 0.067223 | 0.390876 | 0.081743 | 0.11312 |
| 8.592882 | 23.58148 | 0.505693 | 0.482927 | 0.037776 | 0 | 0.116149 | 0.072184 | 0.029122 |
| 3.221343 | 0.657019 | 0.182588 | 1.02178 | 0.127303 | 0.068905 | 0.518623 | 0.159222 | 0.029442 |
| 1.167718 | 23.88405 | 0.881763 | 1.773507 | 0.141872 | 0 | 0.340788 | 0.104742 | 0.009114 |
| 5.895922 | 12.42605 | 0.446539 | 0.399015 | 0.07472 | 0 | 0.186663 | 0.064899 | 0.019201 |
| 0.353714 | 4.805656 | 0.403981 | 0.3024 | 0.081249 | 0.095951 | 1.267797 | 0.093152 | 0.041757 |
| 7.79836 | 2.379431 | 0.081684 | 0.10368 | 0.640701 | 0 | 0.032832 | 0.356155 | 0.010976 |
| 4.204244 | 4.527306 | 0.550935 | 0.328842 | 0.037173 | 0.012194 | 0.19049 | 0.071032 | 0.025473 |
| 13.25368 | 2.117046 | 0.046623 | 0.339282 | 0.048759 | 0 | 0.093699 | 0.093171 | 0.01253 |
| 18.76766 | 2.018951 | 0.491541 | 0.256901 | 0.120956 | 0.126476 | 0.139463 | 0.073541 | 0.011656 |
| 14.88331 | 4.788849 | 1.0633 | 0.12402 | 0.075137 | 0.007394 | 0.11551 | 0.036546 | 0.108124 |
| 0.908744 | 5.355409 | 0.476755 | 4.579819 | 0.047486 | 2.126308 | 0.882102 | 0.112735 | 0.061012 |
| 3.507003 | 4.277032 | 0.522613 | 0.675882 | 0.097891 | 0.144506 | 0.232008 | 0.087859 | 0.025155 |
| 0.282039 | 0.53466 | 0.281435 | 0.552914 | 0.038391 | 0 | 0.191814 | 0.077805 | 0.138115 |
| 8.543283 | 0.705766 | 0.148873 | 0.156208 | 0.083037 | 0.005107 | 0.067817 | 0.045077 | 0.016004 |
| 10.83046 | 0.228724 | 0.088988 | 0.341865 | 0.195439 | 0.004579 | 0.089421 | 0.347587 | 0.023915 |
| 2.445679 | 6.943506 | 1.241804 | 2.519148 | 0.051265 | 0.058859 | 0.210163 | 0.092022 | 0.008782 |
| 2.292649 | 6.097611 | 0.366219 | 1.454725 | 0.115623 | 0 | 0.416603 | 0.326385 | 0.025998 |
| 12.15059 | 0.59324 | 0.115404 | 0.143225 | 0.268205 | 0 | 0.113388 | 0.060568 | 0.048245 |
| 7.300249 | 5.779054 | 0.163011 | 0.119863 | 0.188116 | 0 | 0.090374 | 0.055145 | 0.02417 |
| 9.759557 | 7.621257 | 0.082365 | 1.366052 | 0.344558 | 0.016954 | 0.278093 | 0.047884 | 0.026562 |
| 0.112838 | 5.6396 | 1.665451 | 0.550291 | 3.151764 | 0.081625 | 0.286894 | 0.043224 | 0 |
| 0.060824 | 33.51269 | 0.229015 | 1.317767 | 0.35128 | 0.062855 | 0.331385 | 0.038832 | 0.114887 |

| | | | | | | | | |
|---|---|---|---|---|---|---|---|---|
| 11.97604 | 0.977097 | 0.253436 | 0.522731 | 0.115959 | 0.048908 | 0.286501 | 0.094963 | 0.076623 |
| 1.13156 | 3.785458 | 6.240931 | 6.468041 | 0.288801 | 0.284217 | 0.432884 | 0.190641 | 0.022264 |
| 14.66882 | 0.403759 | 0.108753 | 0.067485 | 0.151649 | 0.055966 | 0.050998 | 0.062566 | 0.004871 |
| 6.53629 | 0.55798 | 0.500977 | 0.029674 | 2.960218 | 0.232027 | 0.17116 | 0.072811 | 0.390442 |
| 9.776899 | 1.860293 | 0.109478 | 0.142046 | 0.15266 | 0 | 0 | 0.235357 | 0.657086 |
| 11.03064 | 2.300905 | 0.190019 | 1.560746 | 0.07949 | 0.06519 | 0.203672 | 0.115071 | 0.020427 |
| 10.82939 | 16.28611 | 0.481973 | 2.101186 | 0.483895 | 0.158738 | 0.24797 | 0.196138 | 0.32123 |
| 10.54067 | 1.693534 | 0 | 0.604399 | 0.121603 | 0 | 0.046736 | 0.084497 | 0.008928 |
| 7.482917 | 0.902445 | 0.018372 | 0.041456 | 0.038428 | 0.441206 | 0.063999 | 0.026702 | 0.003292 |
| 6.224016 | 0.919527 | 0.314578 | 1.321561 | 0.30964 | 0 | 0.416517 | 0.134472 | 0.009946 |
| 14.4184 | 0.980975 | 0.034488 | 0.245136 | 0.129844 | 0.007099 | 0.14971 | 0.057642 | 0.007415 |
| 4.027757 | 4.784353 | 0.177534 | 0.17481 | 0.050637 | 0.008306 | 0.04541 | 0.082099 | 0.004337 |
| 0.080072 | 4.948523 | 0.896002 | 1.759488 | 0.21684 | 0.015243 | 0.309544 | 0.150672 | 0.083582 |
| 0.1958 | 0.621097 | 0.116728 | 20.36809 | 0.064251 | 0.158077 | 0.953176 | 0.285727 | 0.141991 |
| 4.524567 | 16.15396 | 0.019438 | 2.460634 | 0.203286 | 1.960579 | 0.203137 | 0.056502 | 0.020895 |
| 9.669362 | 0.455194 | 0.1155 | 0.175922 | 0.048317 | 0 | 0.157843 | 0.029377 | 0.043456 |
| 1.434354 | 14.54432 | 0.630075 | 0.533161 | 0.433022 | 0.018528 | 0.180897 | 0.114468 | 0.019352 |
| 0.513565 | 2.034534 | 1.353575 | 18.82435 | 0 | 0.024441 | 0.229079 | 0.759297 | 1.480595 |
| 0.758898 | 2.212593 | 0.725807 | 0.565605 | 0.794369 | 1.798048 | 4.158903 | 7.727598 | 0.798373 |
| 39.55322 | 2.115508 | 0.075588 | 0.238789 | 0.070268 | 0.008644 | 0.12828 | 0.079342 | 0.022571 |
| 7.070481 | 5.043909 | 0.075477 | 1.921138 | 0.284168 | 0.015537 | 0.582481 | 0.170031 | 0.016227 |
| 3.917416 | 2.960213 | 0.458886 | 0.977608 | 0.677523 | 0.027782 | 0.49909 | 0.088271 | 0.10156 |
| 4.053387 | 3.596342 | 0.653728 | 1.548892 | 0.791633 | 0.141649 | 0.69148 | 0.112514 | 0.018493 |
| 2.683173 | 3.184044 | 0.015357 | 0.080568 | 0.080303 | 0.015806 | 0.067899 | 0.159028 | 0.012381 |
| 3.783265 | 0.337072 | 0.040699 | 0.10791 | 0.113505 | 0.006981 | 0.043624 | 0.25879 | 0.025521 |
| 0.032002 | 18.52735 | 4.964428 | 0.940768 | 0.151221 | 0.115749 | 2.027711 | 0.178046 | 0.077718 |
| 4.908188 | 1.252673 | 0.022845 | 0.668871 | 0.143354 | 0.058783 | 0.183652 | 0.153565 | 0.049117 |
| 3.857335 | 2.754787 | 0.780709 | 0.185954 | 0.024192 | 0 | 0.209201 | 0.121872 | 0.021758 |
| 2.000854 | 4.684428 | 0.21072 | 5.345493 | 0.023197 | 0.125561 | 0.566137 | 0.487595 | 0.262286 |
| 10.22422 | 1.337079 | 0.017734 | 0.708312 | 0.111282 | 0 | 0.028513 | 0.070881 | 0.076257 |
| 3.844641 | 7.298171 | 0.171616 | 0.538629 | 0.358958 | 0.032115 | 0.413879 | 0.113375 | 0.016771 |
| 7.376916 | 25.06176 | 0.078557 | 0.244141 | 0.149376 | 0.00735 | 0.074633 | 0.088226 | 0.007677 |
| 2.743838 | 13.52627 | 0.329216 | 1.552609 | 0.061209 | 0.331307 | 0.382277 | 0.167468 | 0.664548 |
| 1.99204 | 8.246775 | 0.734419 | 0.370228 | 0.882464 | 0.016083 | 0.540145 | 0.147619 | 0.041994 |
| 1.184124 | 11.87357 | 0.588002 | 0.474594 | 0.018921 | 0.651736 | 0.203619 | 0.062451 | 0.009724 |
| 14.5588 | 2.611859 | 0.186768 | 0.344631 | 0.088212 | 0.086812 | 0.125925 | 0.083185 | 0.016191 |
| 4.43349 | 0.20536 | 0.029962 | 0.07606 | 0.407351 | 0 | 0.060215 | 0.070763 | 0.008052 |
| 0.024008 | 38.55082 | 3.93717 | 0.204654 | 0.23529 | 0.014472 | 0.101734 | 0.081746 | 0.015116 |
| 6.545065 | 7.443935 | 0.021089 | 0.820864 | 0.04411 | 0.021705 | 0.096066 | 0.117491 | 0.007557 |
| 13.0968 | 1.460996 | 0.087003 | 0.126628 | 0.072792 | 0.116409 | 0.538545 | 0.047419 | 0.084174 |
| 1.271987 | 5.348672 | 0.433799 | 0.53944 | 0.2367 | 0.727948 | 0.288078 | 0.109648 | 0.0811 |
| 15.52266 | 3.511589 | 0.149523 | 1.152118 | 0.073588 | 0.009053 | 0.509083 | 0.319582 | 0.373472 |
| 4.637627 | 1.560572 | 0.07989 | 0.827444 | 0.434463 | 0.016445 | 0.231201 | 0.191583 | 0.060116 |
| 0 | 5.72881 | 0.339423 | 2.406858 | 0.070995 | 0.017467 | 0.191001 | 0.129495 | 0.018244 |
| 0.628349 | 9.92488 | 0.883256 | 0.891129 | 0.17764 | 1.870577 | 0.068273 | 0.055545 | 0.136945 |
| 0.363539 | 86.46912 | 0.299 | 0.418616 | 0.198991 | 0.055952 | 0.120181 | 0.148147 | 0.021915 |
| 4.28612 | 2.362061 | 0.095802 | 1.711406 | 0.166987 | 0.032867 | 0.295222 | 0.075421 | 0.028607 |
| 5.769932 | 1.348105 | 0.089403 | 0.387337 | 0.374 | 0 | 0.07187 | 0.045478 | 0.009611 |
| 11.61963 | 0.319331 | 0.041413 | 0.553687 | 0 | 0 | 0.116521 | 0.052666 | 0.022259 |
| 0.307897 | 29.28621 | 0.055205 | 0.108375 | 0.069281 | 0.102272 | 0.142011 | 0.116339 | 0.023738 |
| 3.210065 | 4.626556 | 0.169636 | 4.236929 | 0.458307 | 0.152769 | 0.431833 | 0.200319 | 0.231744 |
| 0.450464 | 50.52796 | 0.488378 | 3.88748 | 0.281796 | 0 | 0.433215 | 0.110142 | 0.009052 |
| 3.088461 | 0.813643 | 0.49745 | 1.033205 | 0.141885 | 0 | 0.218124 | 0.098589 | 0.012153 |
| 0.423203 | 10.2374 | 0.965836 | 1.40022 | 0.107266 | 0.202331 | 0.151162 | 0.080746 | 0.004594 |
| 0.209366 | 34.57977 | 0.595609 | 2.371744 | 0.659541 | 0.216357 | 0.225319 | 0.197317 | 0.028247 |
| 0.060027 | 23.0559 | 5.75581 | 3.03959 | 0.220612 | 0.046524 | 0.46028 | 0.164243 | 0.097184 |
| 16.19995 | 0.875118 | 0.195274 | 0.696541 | 0.094256 | 0 | 0.301881 | 0.092783 | 0.024221 |
| 7.869029 | 4.393248 | 0.091567 | 0.247945 | 0 | 0 | 0 | 0 | 0.049217 |

| | | | | | | | | |
|---|---|---|---|---|---|---|---|---|
| 13.50516 | 0.724029 | 0.135213 | 0.268876 | 0.070704 | 0.086978 | 0.149457 | 0.030706 | 0.063591 |
| 0.720889 | 9.485721 | 0.703707 | 3.271099 | 0.245317 | 0.168996 | 0.829697 | 0.238644 | 0.075647 |
| 5.798415 | 3.838167 | 0.426257 | 0.738359 | 0.052446 | 0 | 0.167972 | 0.085032 | 0.022462 |
| 0.221255 | 10.42952 | 0.93547 | 1.805216 | 0 | 0 | 0.068365 | 0.384043 | 0.01306 |
| 4.753897 | 4.228866 | 0.069034 | 0.578311 | 0.252689 | 0 | 0.083243 | 0.062708 | 0 |
| 0.159078 | 4.648669 | 0.263074 | 0.845915 | 0.171955 | 0.016923 | 0.211482 | 0.083639 | 0.044187 |
| 0.04161 | 18.13404 | 0.621452 | 2.165321 | 0.344079 | 0.018812 | 0.279176 | 0.139466 | 0.098242 |
| 0.190051 | 9.034838 | 0.298786 | 26.78199 | 0.294096 | 0.036178 | 0.536897 | 0.485341 | 0.037787 |
| 16.502 | 0.677268 | 0.01581 | 0.57794 | 0.264552 | 0 | 0.025419 | 0.034467 | 0.008498 |
| 10.46069 | 12.0414 | 0.205177 | 0.584514 | 0.035763 | 0.087989 | 0.10996 | 0.118038 | 0 |
| 5.929477 | 0.599428 | 0.106468 | 2.167353 | 0.127253 | 0.062617 | 0.379035 | 0.116055 | 0.057226 |
| 11.98656 | 5.155608 | 0.062683 | 0.3819 | 0 | 0.12903 | 0.201561 | 0.182206 | 0 |
| 11.78604 | 0.687294 | 0.160441 | 1.077748 | 0.064536 | 0.025404 | 0.223229 | 0.116591 | 0.053068 |
| 2.66995 | 0.931199 | 0.055453 | 2.734727 | 0.266774 | 0 | 0.307591 | 0.141042 | 0.014903 |
| 6.564894 | 1.847781 | 0.326775 | 1.211125 | 3.759231 | 0.050449 | 0.525382 | 0.130606 | 0.061473 |
| 0.165222 | 0.248726 | 4.354662 | 0.816448 | 0.098228 | 0.030758 | 0.429003 | 0.131854 | 0.227175 |
| 0.356617 | 5.048306 | 2.495452 | 0.167441 | 0.068981 | 0.045258 | 0.123722 | 0.035949 | 0.04727 |
| 0.328342 | 39.36088 | 0.136641 | 1.217902 | 0.127024 | 0.015626 | 0.183073 | 0.09378 | 0.032641 |
| 0.017547 | 11.7549 | 0.693719 | 4.309998 | 0 | 0 | 0.074356 | 0.156838 | 0.555159 |
| 4.249096 | 0.458625 | 0.056348 | 0.104898 | 0.206254 | 0.014499 | 0.101919 | 0.102369 | 0.007572 |
| 0.168687 | 0.749734 | 4.403645 | 2.469869 | 0.428068 | 0.56654 | 0.720489 | 0.212827 | 0.189657 |
| 11.55219 | 0.704458 | 0.202819 | 2.135539 | 0.068793 | 0 | 0.237956 | 0.12747 | 0.005893 |
| 5.447575 | 6.593096 | 0.077471 | 0.984774 | 0.054014 | 0.168328 | 0.214512 | 0.037532 | 0.04164 |
| 3.482713 | 5.256693 | 0.024156 | 0.490567 | 0.101051 | 0.024862 | 0.116511 | 0.032182 | 0.043278 |
| 2.050171 | 14.32894 | 0.331902 | 1.106963 | 0.287848 | 0.324937 | 0.318873 | 0.373552 | 0.6048 |
| 5.521733 | 3.178826 | 0.575736 | 0.274264 | 0.133804 | 0.087787 | 0.154276 | 0.061983 | 0.137535 |
| 0.152392 | 3.211773 | 0.116033 | 2.07851 | 0.149354 | 0.055119 | 0.337234 | 0.05189 | 0.076759 |
| 1.219672 | 7.699776 | 0.076045 | 4.389709 | 0.130139 | 0.092497 | 0.400134 | 0.138153 | 0.0706 |
| 0.175296 | 35.81241 | 0.140005 | 0.248314 | 0.105423 | 0.005764 | 0.036016 | 0.044766 | 0.01505 |
| 2.80417 | 0.29397 | 0.089211 | 3.502699 | 0.043061 | 0.148321 | 0.29238 | 0.139632 | 0.040573 |
| 4.07564 | 5.144704 | 0.567445 | 0.790143 | 0.030048 | 0.022178 | 0.207872 | 0.057417 | 0.115821 |
| 6.105885 | 6.067958 | 0.262314 | 1.269647 | 0.109733 | 0.107992 | 0.084348 | 0.123904 | 0.056396 |
| 6.852977 | 1.92441 | 0.244147 | 0.690851 | 0.367682 | 0.16082 | 0.290476 | 0.124194 | 0.498662 |
| 0.917449 | 1.335601 | 0.699735 | 0.713525 | 0.05558 | 0.173208 | 0.719157 | 0.479528 | 1.390145 |
| 6.328888 | 12.80051 | 0.215381 | 0.529492 | 0.047421 | 0.140005 | 0.109353 | 0.065902 | 0.018279 |
| 5.006146 | 6.866533 | 0.068306 | 0.649924 | 0.031749 | 0.078113 | 0.183035 | 0.041365 | 0.004079 |
| 8.675405 | 1.265135 | 0.751396 | 1.629912 | 0.041 | 0.01345 | 0.430706 | 0.265895 | 0.217738 |
| 12.53948 | 5.606439 | 0.13923 | 0.636196 | 0 | 0.107474 | 0.083944 | 0.164413 | 0.018709 |
| 10.96813 | 26.26414 | 0.092654 | 0.389773 | 0.138428 | 0.040869 | 0.138327 | 0.072141 | 0.042686 |
| 9.026563 | 0.915194 | 0.023222 | 0.487321 | 0.113334 | 0.039834 | 0.074671 | 0.053438 | 0.033284 |
| 26.46639 | 0.096084 | 0.785036 | 0.132857 | 0.058643 | 0.019238 | 0.127719 | 0.057727 | 0.050232 |
| 9.166892 | 1.788323 | 0.104366 | 0.306151 | 0.472975 | 0.143221 | 0.055932 | 0.056882 | 0 |
| 7.311644 | 4.767265 | 0.025524 | 0.155508 | 0.071184 | 0.061297 | 0.068396 | 0.068011 | 0.013719 |
| 10.83115 | 1.636972 | 0.105312 | 3.964279 | 0.157339 | 0 | 0.235836 | 0.142126 | 0.024259 |
| 11.26017 | 11.93359 | 0.056981 | 0.143579 | 0.132428 | 0 | 0.066165 | 0.018404 | 0 |
| 9.369652 | 0.847664 | 0.098939 | 0.314789 | 0.248333 | 0.020366 | 0.206793 | 0.086278 | 0.021271 |
| 9.059225 | 4.760834 | 0.231534 | 0.169798 | 0.080714 | 1.012772 | 0.11633 | 0.056085 | 0.051853 |
| 2.439399 | 14.01868 | 0.142283 | 0.252835 | 0.595209 | 0.07322 | 0.114379 | 0.051698 | 0.009559 |
| 0.054601 | 0.60278 | 0.409344 | 0.134223 | 0.066891 | 0.092161 | 1.93327 | 0.058099 | 0.003438 |
| 10.67162 | 1.539172 | 0.070176 | 0.91686 | 0.146784 | 0.534478 | 0.191807 | 0.045897 | 0.007544 |
| 3.611039 | 1.54619 | 0.579404 | 2.147602 | 0.317877 | 0.14664 | 0.328334 | 0.144951 | 0.260371 |
| 8.295138 | 11.28912 | 0.027976 | 1.273591 | 0.731441 | 0.071983 | 0.303607 | 0.055907 | 0.09022 |
| 4.407477 | 11.50715 | 1.328141 | 2.50097 | 0.195633 | 0.077011 | 0.330829 | 0.197108 | 0.030163 |
| 0.921441 | 3.991956 | 0.080587 | 0.453783 | 0.153235 | 0.052781 | 0.159012 | 0.090505 | 0.019688 |
| 5.77735 | 7.343436 | 0.11971 | 0.102647 | 0.150234 | 0.024642 | 0.064155 | 0.034797 | 0.012869 |
| 9.860364 | 4.952921 | 0.922872 | 0.218069 | 0.036421 | 0.017922 | 0.139979 | 0.037961 | 0.037437 |
| 0.891105 | 9.428645 | 1.610494 | 3.346375 | 0.187143 | 0.276259 | 0.539441 | 0.195056 | 0 |
| 1.803256 | 15.6604 | 0.464713 | 1.644431 | 1.001468 | 0.072469 | 0.350936 | 0.127918 | 0 |

| | | | | | | | | |
|---|---|---|---|---|---|---|---|---|
| 1.382642 | 1.910498 | 0.035209 | 0.091367 | 0.098194 | 0.012079 | 0.037739 | 0.03838 | 0.006308 |
| 6.351089 | 0.655141 | 0.645195 | 1.059557 | 0.299893 | 0.221349 | 0.211308 | 0.347303 | 0.102752 |
| 5.302309 | 0.399418 | 0.429776 | 0.643512 | 0.457087 | 0.172436 | 0.374771 | 0.264674 | 0.152695 |
| 1.178128 | 10.39841 | 0.737291 | 0.547099 | 0 | 0 | 0.182369 | 0.010304 | 0 |
| 14.65658 | 7.912157 | 0.082016 | 2.048713 | 0.223013 | 0.033765 | 0.309879 | 0.086421 | 0.052899 |
| 0 | 10.00922 | 2.808876 | 0.950733 | 0.263067 | 2.258108 | 4.426175 | 7.20003 | 0.052578 |
| 0.717485 | 6.518094 | 0.109309 | 0.77696 | 0.030485 | 0.060001 | 0.146453 | 0.121799 | 0.08617 |
| 4.647599 | 4.189777 | 0.126609 | 1.612379 | 0.148962 | 0.146597 | 0.267171 | 0.247266 | 0.182888 |
| 4.527189 | 3.068857 | 0.162816 | 0.082663 | 0.194601 | 0 | 0.009349 | 0.042256 | 0.006251 |
| 0.265897 | 2.92514 | 1.266848 | 0.270658 | 0.075171 | 0.416128 | 0.115564 | 0.075085 | 0.072438 |
| 7.142188 | 1.092949 | 0.350813 | 3.66478 | 0.10006 | 0.032824 | 0.269195 | 0.168024 | 0.008571 |
| 3.169786 | 5.924635 | 0.20439 | 0.703337 | 0.498762 | 0.087651 | 0.301229 | 0.129963 | 0.027464 |
| 3.1313 | 2.379821 | 0.06232 | 1.120988 | 0.148974 | 0.32987 | 0.32922 | 0.271727 | 0.062208 |
| 14.61848 | 1.720144 | 0.038859 | 0.879054 | 0.067733 | 0.026663 | 0.078097 | 0.016473 | 0.010443 |
| 4.244916 | 18.6208 | 0.44456 | 0.936272 | 0 | 0.254195 | 0.198543 | 0.107686 | 0 |
| 12.37333 | 1.491571 | 0.117445 | 0.121595 | 0.0289 | 0 | 0.033322 | 0.010041 | 0.014853 |
| 1.045358 | 2.686122 | 0.14251 | 2.379643 | 0.13248 | 0.179269 | 0.114562 | 0.092054 | 0.042554 |
| 5.608971 | 0.890669 | 0.198341 | 2.893683 | 0.071527 | 0.035196 | 0.280402 | 0.131708 | 0.01838 |
| 6.86332 | 3.630648 | 0.059407 | 0.958461 | 0.082838 | 0.081523 | 0.238781 | 0.158291 | 0.085148 |
| 3.542922 | 1.726841 | 0.127567 | 1.608384 | 0.200118 | 0.118165 | 0.287139 | 0.09502 | 0.051424 |
| 5.496867 | 1.186653 | 0.184183 | 1.513641 | 0.092459 | 0.007583 | 0.195442 | 0.141875 | 0.01188 |
| 7.142035 | 0.41987 | 0.07001 | 0.361372 | 0.439306 | 0.018014 | 0.23919 | 0.006359 | 0.03763 |
| 15.81192 | 3.632712 | 0.190803 | 0.290619 | 0.044344 | 0.01091 | 0.042607 | 0.034664 | 0.051277 |
| 0.542857 | 1.458062 | 0.199005 | 2.62697 | 0.157887 | 0.035314 | 0.397187 | 0.142123 | 0.162289 |
| 2.259942 | 1.081849 | 0.049844 | 1.017887 | 0.069504 | 0 | 0.213703 | 0.193182 | 0.035721 |
| 0 | 7.811129 | 0.587965 | 2.053992 | 0.030366 | 0.022413 | 0.717741 | 0.174073 | 0.039016 |
| 12.66578 | 1.49059 | 0.338296 | 0.621595 | 0.303254 | 0.02487 | 0.233102 | 0.087799 | 0 |
| 4.302472 | 1.782461 | 0.139102 | 1.234375 | 0.1518 | 0.012449 | 0.457012 | 0.129652 | 0.117025 |
| 3.016936 | 16.24716 | 0.20767 | 0.405098 | 0.160032 | 0.016874 | 0.114225 | 0.228355 | 0 |
| 7.624185 | 0.995723 | 0.601854 | 0.107125 | 0.108523 | 0.03738 | 0.033367 | 0.033933 | 0.02231 |
| 2.452311 | 4.347372 | 0.55844 | 1.974184 | 0.187466 | 0.078054 | 0.266029 | 0.075151 | 0.092641 |
| 1.714136 | 4.845769 | 0.13795 | 3.509719 | 0.04809 | 0 | 0.295724 | 0.175433 | 0.061789 |
| 5.653016 | 3.744916 | 0.084058 | 0.203903 | 0.117213 | 0.014419 | 0.292819 | 0.055994 | 0.097891 |
| 1.066384 | 33.99543 | 0.925848 | 0.184046 | 0.061478 | 0.08319 | 0.082698 | 0.032038 | 0.003949 |
| 6.736172 | 3.889572 | 0.039306 | 0.212867 | 0.051384 | 0 | 0.082944 | 0.023208 | 0.015845 |
| 1.425502 | 3.231215 | 0.44134 | 3.301929 | 0.035966 | 0.188774 | 1.290138 | 0.274902 | 0.834877 |
| 3.003359 | 0.304403 | 0.423221 | 0.199842 | 0.076501 | 0 | 0.067204 | 0.064548 | 0.008425 |
| 0.201284 | 22.75974 | 0.950995 | 0.300606 | 0.065757 | 0.072802 | 0.056863 | 0.074249 | 0.004224 |
| 8.096409 | 0.397988 | 0.091247 | 0.788962 | 0.052051 | 0.691541 | 0.186715 | 0.150701 | 0.06242 |
| 7.84603 | 3.779666 | 0.034466 | 0.16332 | 0.144179 | 0.035473 | 0.471011 | 0.112707 | 0.03705 |
| 6.086395 | 1.63882 | 0.510809 | 1.397881 | 0.068198 | 0.016779 | 0.327633 | 0.104647 | 0.25411 |
| 5.180288 | 7.374324 | 0.070717 | 0.468459 | 0.063392 | 0.010398 | 0.170545 | 0.080755 | 0.02172 |
| 4.502457 | 2.247754 | 1.96091 | 5.630156 | 0.056573 | 0.278374 | 0.358756 | 0.068792 | 0.029075 |
| 2.071736 | 1.448171 | 0.341114 | 0.616926 | 1.778395 | 0.461122 | 0.421558 | 0.153541 | 0.191555 |
| 8.543273 | 0.966192 | 0.213016 | 0.95003 | 0.078627 | 0.025793 | 0.292117 | 0.186668 | 0.020205 |
| 8.328148 | 1.866288 | 0.146392 | 0.2973 | 0.036744 | 0.078348 | 0.45661 | 0.123404 | 0.195138 |
| 14.74281 | 1.582755 | 0.008247 | 0.097702 | 0.034501 | 0.016976 | 0.046409 | 0.014983 | 0.039895 |
| 5.006677 | 9.75827 | 0.043695 | 0.433827 | 0.335111 | 0 | 0.128794 | 0.079382 | 0.007829 |
| 6.815224 | 7.248892 | 0.134299 | 0.719733 | 0.187271 | 0.046075 | 0.431846 | 0.05693 | 0.012031 |
| 4.952876 | 9.283842 | 0.101588 | 0.939853 | 0.283314 | 0 | 0.244996 | 0.147646 | 0 |
| 5.846927 | 19.54043 | 0 | 0.591151 | 0.429775 | 0 | 0.247765 | 0.186645 | 0 |
| 12.884 | 1.120958 | 0.198057 | 0.385971 | 0.288729 | 0.04324 | 0.221937 | 0.085047 | 0.058066 |
| 7.012394 | 4.440539 | 0.127189 | 0.645755 | 0.199526 | 0.147269 | 0.153369 | 0.057767 | 0.042727 |
| 4.314644 | 16.30831 | 0.095447 | 0.2123 | 0.370763 | 0.098236 | 0.142497 | 0.014863 | 0 |
| 18.83247 | 2.427344 | 0.144204 | 1.780101 | 0.390335 | 0.165878 | 0.334133 | 0.206502 | 0.373863 |
| 3.864046 | 3.339364 | 0.922032 | 1.418564 | 0.219155 | 0.08627 | 0.202148 | 0.091368 | 0.011263 |
| 5.561606 | 5.931941 | 0.314714 | 0.715614 | 0.016879 | 0.016611 | 0.285431 | 0.580549 | 0.091084 |
| 11.17992 | 0.559456 | 0.072555 | 1.387523 | 0.113819 | 0.018669 | 0.306211 | 0.131813 | 0.097494 |

| | | | | | | | | |
|---|---|---|---|---|---|---|---|---|
| 10.58728 | 0.424546 | 0.055058 | 0.435614 | 0.043186 | 0.007083 | 0.094054 | 0.07502 | 0.007398 |
| 12.90121 | 2.762898 | 0.087552 | 0.244482 | 0.305213 | 0.052564 | 0.246337 | 0.076878 | 0.031372 |
| 3.089962 | 5.58879 | 0.099439 | 2.158569 | 0.13866 | 0.020469 | 0.287776 | 0.072262 | 0.07839 |
| 2.658105 | 9.398761 | 1.265121 | 0.492736 | 0.054374 | 0.517268 | 0.557269 | 0.217245 | 0.153697 |
| 11.66958 | 2.925073 | 0.059549 | 0.524048 | 0.290627 | 0.02043 | 0.207439 | 0.072123 | 0 |
| 0.768549 | 22.06783 | 0.312728 | 0.27906 | 0.11893 | 0.029261 | 0.114272 | 0.046484 | 0.00764 |
| 3.821996 | 8.060298 | 0.226879 | 0.240617 | 0.316367 | 0.015567 | 0.109431 | 0.016487 | 0.016259 |
| 1.274671 | 20.43625 | 0.08399 | 0.208474 | 0.351352 | 0.028815 | 0.090024 | 0.010172 | 0.015048 |
| 6.801517 | 10.53958 | 0.163297 | 0.128968 | 0.177105 | 0.043573 | 0.087515 | 0.039556 | 0.003251 |
| 0.696511 | 1.639083 | 0.021101 | 1.073685 | 0.029423 | 0 | 0.169626 | 0.08178 | 0.018902 |
| 1.115752 | 1.362521 | 0.260484 | 2.044086 | 0.186393 | 0.042331 | 0.374716 | 0.231634 | 0.029475 |
| 11.89527 | 0.448732 | 0.114572 | 0.609394 | 0.205408 | 0.050537 | 0.315781 | 0.065417 | 0.017595 |
| 0.066707 | 91.64178 | 0.200934 | 1.02583 | 0.280187 | 0.155104 | 0.107685 | 0.073009 | 0.036 |
| 0.374457 | 1.759456 | 3.309723 | 2.645424 | 0.104258 | 0.092343 | 2.131724 | 0.789644 | 1.366358 |
| 1.054186 | 12.34466 | 0.867038 | 0.517575 | 0.181353 | 0.089237 | 0.088709 | 0.071599 | 0.004237 |
| 3.078617 | 2.778376 | 0.200094 | 0.102758 | 0.101023 | 0.312462 | 0.088747 | 0.072703 | 0.044503 |
| 4.532863 | 5.066642 | 1.056925 | 0.187389 | 0.052636 | 0.0259 | 0.151723 | 0.137154 | 0.040578 |
| 0.248249 | 13.73059 | 0.355421 | 0.973342 | 0.304123 | 0.199531 | 0.753258 | 0.0587 | 0.02605 |
| 1.554931 | 3.901335 | 0.281605 | 0.342732 | 0.087726 | 0.604334 | 0.32271 | 0.130622 | 0.028984 |
| 4.77368 | 3.253588 | 1.474782 | 0.224629 | 0.038559 | 0 | 0.177834 | 0.207645 | 0 |
| 0.388684 | 2.311249 | 0.102443 | 0.918872 | 0.249987 | 0 | 0.192157 | 0.080649 | 0.055062 |
| 4.3646 | 2.773535 | 0.035341 | 0.844522 | 0.029568 | 0.123671 | 0.181826 | 0.089888 | 0.015196 |
| 3.417216 | 1.162477 | 0.304118 | 0.282415 | 0.40776 | 0.016052 | 0.156716 | 0.181334 | 0.012574 |
| 8.140555 | 0.674309 | 0.085549 | 0.492253 | 0.178937 | 0.158487 | 0.178806 | 0.149203 | 0.202319 |
| 9.849327 | 9.979593 | 0 | 0.477378 | 0.17353 | 0 | 0.080032 | 0.060289 | 0.053511 |
| 6.01614 | 1.019824 | 0.02029 | 0.345667 | 0.11317 | 0.006961 | 0.135922 | 0.019659 | 0.014541 |
| 6.720562 | 0.592992 | 0.063563 | 0.953814 | 0.103407 | 0.029076 | 0.181682 | 0.105213 | 0.026573 |
| 8.997031 | 1.078894 | 0.182798 | 0.256656 | 0.042483 | 0 | 0.038098 | 0.083639 | 0.018195 |
| 0.865122 | 10.881 | 0.5966 | 0.661125 | 0.051282 | 0.269165 | 0.151107 | 0.065329 | 0.004393 |
| 3.587125 | 4.525779 | 0.140065 | 0.833034 | 0 | 0.123564 | 0.514728 | 0.196299 | 0 |
| 18.18142 | 1.165351 | 0.026846 | 0.154473 | 0.018717 | 0.00921 | 0.086324 | 0.055275 | 0.00962 |
| 8.515845 | 7.671597 | 0.027851 | 0.58683 | 0.058255 | 0 | 0.044779 | 0.068308 | 0.007485 |
| 6.299032 | 0.905545 | 0.029914 | 0.079312 | 0.072997 | 0.005131 | 0.064126 | 0.04891 | 0.00268 |
| 6.318767 | 0.629894 | 0.095897 | 0.414748 | 0.111434 | 0.01645 | 0.44541 | 0.073559 | 0.002864 |
| 1.231572 | 4.441911 | 0.503429 | 0.658702 | 0 | 0.525873 | 0.096645 | 0.19384 | 0.016155 |
| 5.189106 | 2.495753 | 0 | 2.234897 | 0.04062 | 0.069957 | 0.764971 | 0.373982 | 0.062629 |
| 2.361371 | 2.678671 | 0.117245 | 2.191456 | 0.081745 | 0 | 0.172795 | 0.144368 | 0.031509 |
| 12.1953 | 0.699703 | 0.03889 | 0.309336 | 0.10168 | 0.100066 | 0.039079 | 0.042392 | 0 |
| 7.661595 | 1.391756 | 0.63941 | 0.239821 | 0 | 0 | 0.097246 | 0.025117 | 0.018577 |
| 0.004732 | 19.83159 | 0.041572 | 1.134127 | 0.260861 | 39.05576 | 18.50758 | 1.706871 | 0.017876 |
| 7.519384 | 3.918945 | 0.035186 | 1.381503 | 0.404779 | 0 | 0.353569 | 0.051139 | 0.028368 |
| 19.30692 | 0.473277 | 0.11373 | 0.566424 | 0.373817 | 0 | 0.052244 | 0.03542 | 0 |
| 6.492529 | 0.443149 | 0.402298 | 0.661384 | 0.090157 | 0.029575 | 0.334952 | 1.383428 | 3.135351 |
| 1.188262 | 4.519116 | 0.305248 | 0.528991 | 0.166002 | 0.09425 | 0.201216 | 0.070983 | 0.04922 |
| 8.006535 | 6.017255 | 0.238445 | 1.672848 | 0.181361 | 0 | 0.45307 | 0.181153 | 0.058255 |
| 1.983131 | 0.723737 | 0.105593 | 0.339534 | 0.110431 | 0 | 0 | 0.230201 | 0 |
| 6.928729 | 1.018286 | 0.658513 | 1.130757 | 0.067189 | 0.330612 | 0.426079 | 0.087537 | 0.474804 |
| 0.554619 | 1.94169 | 0.226633 | 1.887049 | 0.023702 | 0.42569 | 0.469132 | 0.417907 | 0.066997 |
| 12.52942 | 1.264897 | 0.086846 | 2.253622 | 0.18165 | 0.074486 | 0.279257 | 0.19459 | 0.00778 |
| 8.335423 | 2.357539 | 0.259953 | 7.865228 | 0.066308 | 0.05873 | 0.69318 | 0.377813 | 0.419168 |
| 4.246195 | 4.771419 | 0.021256 | 0.366931 | 0.355686 | 0.021877 | 0.085439 | 0.038617 | 0.011425 |
| 7.824986 | 5.82459 | 3.216292 | 0.601069 | 0.047825 | 0.007844 | 0.251202 | 0.116309 | 0.061448 |
| 9.73952 | 1.716518 | 0.292178 | 0.52273 | 0 | 0 | 0.100662 | 0.015166 | 0.022435 |
| 10.77958 | 3.805008 | 0.100584 | 1.579171 | 0.113285 | 0.09556 | 0.323434 | 0.134943 | 0.045746 |
| 12.97983 | 0.435834 | 0.019565 | 0.059602 | 0.068207 | 0.013425 | 0.052428 | 0.004739 | 0.007011 |
| 0.073022 | 23.09846 | 0.601435 | 0.892996 | 0.117412 | 0.61901 | 0.161162 | 0.17191 | 0.112066 |
| 7.448598 | 3.443581 | 0.150725 | 0.391126 | 0.210175 | 0 | 0.121166 | 0.03651 | 0 |
| 7.742833 | 5.451113 | 0.038678 | 0.536749 | 0.060675 | 0 | 0.17101 | 0.105401 | 0.025986 |

| | | | | | | | | |
|---|---|---|---|---|---|---|---|---|
| 2.035719 | 3.064582 | 0.09066 | 1.464566 | 0.137912 | 0.008483 | 0.139136 | 0.065882 | 0.031009 |
| 0.185525 | 4.659061 | 2.111493 | 15.25918 | 0.077482 | 0.282134 | 1.244764 | 0.360721 | 0.135393 |
| 4.066083 | 0.852126 | 0.803964 | 0.406779 | 0.156025 | 0.059713 | 0.13992 | 0.234899 | 0.623683 |
| 8.951038 | 0.487594 | 0.028938 | 0.081623 | 0.211848 | 0.129063 | 0.104684 | 0.075354 | 0.015554 |
| 8.600796 | 2.668503 | 0.111238 | 1.075746 | 0.299147 | 0.065422 | 0.242719 | 0.05774 | 0.051248 |
| 17.22963 | 3.038118 | 0.115993 | 0.280433 | 0.207956 | 0.102328 | 0.066604 | 0.126437 | 0.017813 |
| 3.859765 | 2.622582 | 0.073319 | 0.130287 | 0.038339 | 0 | 0.05894 | 0.03996 | 0.004926 |
| 8.79144 | 5.648373 | 0.262054 | 0.994359 | 1.70207 | 0.241321 | 0.476763 | 0.040091 | 0.326183 |
| 2.334599 | 0.429833 | 0.147558 | 0.267204 | 0.138888 | 0.197431 | 0.166069 | 0.134037 | 0.075346 |
| 1.331847 | 17.31318 | 0.094872 | 0.770685 | 0.165366 | 0.406853 | 0.025422 | 0.068943 | 0 |
| 0.005003 | 5.422841 | 0.281311 | 0.821243 | 0.036775 | 0.009048 | 0.65016 | 0.281087 | 0.047251 |
| 1.530345 | 7.845626 | 0.037175 | 1.02339 | 0 | 0.063768 | 0.189267 | 0.285904 | 0.39296 |
| 12.28294 | 1.196545 | 0.276072 | 0.956419 | 0.033967 | 0 | 0.287205 | 0.10621 | 0.017457 |
| 3.045787 | 2.304269 | 0.14335 | 1.051276 | 0.128502 | 0.196719 | 0.252425 | 0.280272 | 0.022014 |
| 6.610399 | 3.49436 | 0.039407 | 0.736933 | 0.185456 | 0.050698 | 0.300946 | 0.136024 | 0.042361 |
| 1.590175 | 6.117638 | 0.357023 | 2.532452 | 0.259744 | 0.111834 | 0.411792 | 0.112803 | 0.091777 |
| 5.71561 | 0.660892 | 0.089006 | 0.61257 | 0.26374 | 0.061071 | 0.077514 | 0.148226 | 0.07176 |
| 5.653796 | 11.93331 | 0.348212 | 0.339716 | 0.214216 | 0.063245 | 0.164661 | 0.052097 | 0 |
| 7.991017 | 2.615157 | 0.194243 | 2.733167 | 0.348246 | 0.05712 | 0.133843 | 0.070578 | 0.014915 |
| 17.69085 | 1.116535 | 0.059237 | 0.736843 | 0.185854 | 0.060968 | 0.214289 | 0.096856 | 0.00796 |
| 4.248265 | 1.137299 | 0.054553 | 0.753974 | 0.380349 | 0.065504 | 0.138872 | 0.201519 | 0.361632 |
| 4.709477 | 0.781066 | 0.19285 | 1.596264 | 0.03667 | 0.018044 | 0.211404 | 0.681604 | 1.394631 |
| 8.572932 | 0.30998 | 1.561943 | 0.065711 | 0.027687 | 0 | 0.031923 | 0.020841 | 0.002372 |
| 4.20244 | 0.594425 | 1.214164 | 0.058709 | 0.086381 | 0 | 0.039839 | 0.045017 | 0.026637 |
| 5.652868 | 4.499817 | 0.232238 | 1.173052 | 0.298929 | 0.018386 | 0.430831 | 0.38946 | 0.009602 |
| 0.538612 | 24.9195 | 0.646703 | 4.329796 | 0.131968 | 0.040585 | 0.272618 | 0.071639 | 0.021195 |
| 3.269865 | 2.670093 | 0.152958 | 0.317105 | 0.179962 | 0.039357 | 0.161386 | 0.069471 | 0.071937 |
| 10.2921 | 0.328446 | 0.199052 | 0.898319 | 0.092521 | 0.015175 | 0.177795 | 0.077682 | 0.035663 |
| 0.897489 | 8.484106 | 0.392523 | 1.265655 | 0.11523 | 0.014175 | 0.049823 | 0.060051 | 0.022208 |
| 17.91179 | 1.453136 | 0.05548 | 0.633101 | 0.132621 | 0.008157 | 0.165655 | 0.12383 | 0.080939 |
| 2.929112 | 0.431004 | 0.058046 | 0.59596 | 0.242823 | 0.039828 | 0.062217 | 0.133575 | 0.031199 |
| 5.649299 | 12.07774 | 0.094954 | 0.731793 | 0.091666 | 0.022553 | 0.23487 | 0.066349 | 0.066741 |
| 0.956868 | 1.991611 | 0.592114 | 4.654087 | 0.03058 | 0.549226 | 0.458364 | 0.268264 | 0.267177 |
| 1.904787 | 11.24933 | 0.662024 | 0.248976 | 0.038464 | 0 | 0.044349 | 0.060136 | 0.009884 |
| 2.601268 | 6.192685 | 0.250552 | 1.053644 | 0.301735 | 0.164101 | 0.402832 | 0.204145 | 0.563163 |
| 0.131328 | 1.89229 | 0.708751 | 0.46863 | 0.896367 | 0.033928 | 2.968036 | 0.335378 | 0.088592 |
| 5.650162 | 2.625947 | 0.051823 | 2.626123 | 0.170337 | 0.114295 | 0.809399 | 0.231339 | 0.202941 |
| 1.200312 | 10.27325 | 0.173781 | 1.2379 | 0.016522 | 0.03252 | 0.19685 | 0.281272 | 0.012737 |
| 3.603006 | 5.284908 | 0.064932 | 0.653836 | 0.169767 | 0 | 0.130495 | 0.171047 | 0.01745 |
| 5.107057 | 0.981471 | 0.106071 | 1.041166 | 0.17749 | 0 | 0.136431 | 0.146454 | 0.022805 |
| 3.765088 | 1.364285 | 0.021714 | 0.825621 | 0.090837 | 0.014899 | 0.302569 | 0.078898 | 0.00389 |
| 3.446293 | 10.15566 | 0.152794 | 0.337521 | 0.033641 | 0 | 0.187477 | 0.070127 | 0.004322 |
| 14.42499 | 4.389178 | 0.121672 | 0.370644 | 0.254494 | 0.164772 | 0.030887 | 0.211738 | 0.013768 |
| 4.658359 | 3.597512 | 0.297901 | 2.285524 | 0.029672 | 0.102202 | 0.456151 | 0.262872 | 0.343112 |
| 3.888462 | 2.766147 | 0.028421 | 0.529088 | 0.118893 | 0.087754 | 0.123376 | 0.059895 | 0.036662 |
| 13.10373 | 4.116698 | 0.131194 | 0.696616 | 0.017151 | 0.075953 | 0.224114 | 0.119172 | 0.074923 |
| 5.421196 | 2.414325 | 0.297674 | 1.000833 | 0.456594 | 0.061274 | 0.271203 | 0.165844 | 0.031999 |
| 12.83974 | 0.403154 | 0.222208 | 1.499812 | 0.35542 | 0.087445 | 0.294215 | 0.211345 | 0.108897 |
| 0.053959 | 4.486402 | 0.291727 | 2.16245 | 0.335603 | 0.127607 | 0.275556 | 0.254395 | 0.08624 |
| 11.70945 | 2.280038 | 0.169212 | 0.135375 | 0.032176 | 0.055413 | 0.037098 | 0.108992 | 0.012402 |
| 5.734138 | 1.55633 | 0.33796 | 1.201104 | 0.265085 | 0.434795 | 0.475444 | 1.051448 | 1.657561 |
| 3.939864 | 8.36765 | 0.168391 | 2.88146 | 0.195674 | 0 | 0.466265 | 0.061184 | 0 |
| 37.16889 | 1.987636 | 0.008992 | 0.167397 | 0.056425 | 0 | 0.072286 | 0.055543 | 0.004833 |
| 18.59896 | 2.507 | 0.043458 | 0.22064 | 0.302993 | 0.037273 | 0.075693 | 0.073688 | 0.003893 |
| 2.769408 | 14.48207 | 0.039402 | 2.587279 | 0.082414 | 0 | 0.195327 | 0.047722 | 0.021178 |
| 0.025928 | 29.68025 | 2.551267 | 0.749428 | 0.171525 | 0.768989 | 0.065923 | 0.430391 | 0.063667 |
| 11.82149 | 3.894379 | 0.234162 | 1.100283 | 0.316918 | 0 | 0.055365 | 0.150145 | 0.029614 |
| 10.02399 | 1.045574 | 0 | 0.876377 | 0.310453 | 0.016974 | 0.26515 | 0.065914 | 0.008864 |

| | | | | | | | | |
|---|---|---|---|---|---|---|---|---|
| 9.701003 | 3.838007 | 0.360653 | 0.918751 | 0.238218 | 0.019536 | 0.228888 | 0.213806 | 0.010203 |
| 0.457891 | 0.984731 | 0.459748 | 3.1356 | 0.240407 | 0 | 0.110876 | 0.167048 | 0.012356 |
| 3.896265 | 1.36263 | 0.113604 | 1.032131 | 0.137569 | 0.030769 | 0.129777 | 0.063003 | 0.170328 |
| 4.650188 | 2.906119 | 0.044167 | 0.451468 | 0 | 0.136372 | 0.106515 | 0.173317 | 0.807132 |
| 4.636184 | 20.93801 | 0.08565 | 0.154614 | 0.119432 | 0.039179 | 0.114755 | 0.038036 | 0.015345 |
| 0.670319 | 14.66464 | 1.491335 | 1.075523 | 5.334779 | 3.078547 | 0.74248 | 0.357143 | 1.22514 |
| 2.900121 | 64.69988 | 0.157431 | 0.084136 | 0.242634 | 0.04264 | 0.053287 | 0.024085 | 0.008907 |
| 1.086544 | 1.377423 | 0.071773 | 1.102918 | 0.090074 | 0 | 0.173093 | 0.135608 | 0.030862 |
| 0.435036 | 1.240874 | 0.080463 | 2.001754 | 0.063113 | 0.51759 | 0.161709 | 0.054818 | 0.043248 |
| 4.107185 | 3.594492 | 0.07916 | 0.625182 | 0.248361 | 0.040736 | 0.137877 | 0.143812 | 0.021274 |
| 19.15217 | 1.715006 | 0.2133 | 0.161054 | 0.068638 | 0.151985 | 0.03957 | 0.155004 | 0.044095 |
| 9.132795 | 8.392475 | 0.229885 | 0.153459 | 0.253775 | 0.052578 | 0.128334 | 0.030163 | 0.041187 |
| 10.92237 | 4.006702 | 0.245805 | 0.145064 | 0.118647 | 0.304882 | 0.126666 | 0.084732 | 0.030489 |
| 7.411231 | 5.727033 | 0.493984 | 0.697263 | 0.021984 | 0 | 0.38021 | 0.095472 | 0.045193 |
| 2.960538 | 5.567541 | 0.275493 | 1.736045 | 0.067792 | 0.016679 | 0.416876 | 0.203143 | 0.169851 |
| 2.34928 | 5.127441 | 0.36829 | 3.324177 | 2.696157 | 0.126351 | 0.74016 | 0.551995 | 0.255688 |
| 5.289036 | 0.745157 | 0.370446 | 0.496094 | 0.437953 | 0.099462 | 0.090634 | 0.193122 | 0.008657 |
| 0.104629 | 2.992677 | 0.29415 | 17.48872 | 0.05768 | 0.047304 | 0.406422 | 0.561112 | 0.335968 |
| 8.285204 | 0.452863 | 0.022024 | 0.670913 | 0.076777 | 0 | 0.135738 | 0.117368 | 0.063135 |
| 3.778525 | 0.246125 | 0.159597 | 0.064823 | 0.100146 | 0 | 0.038489 | 0.028995 | 0 |
| 2.388459 | 10.43283 | 0.108724 | 0.264963 | 0.272895 | 0.02238 | 0.052441 | 0.023703 | 0 |
| 0.140089 | 2.741575 | 0.140657 | 0.904567 | 0.110327 | 0.018096 | 0.367486 | 0.108603 | 0.028351 |
| 15.19896 | 2.719327 | 0.185975 | 0.411259 | 0.103732 | 0.076564 | 0.15947 | 0.058564 | 0.019992 |
| 10.37294 | 1.324455 | 0.07634 | 0.022609 | 0.119758 | 0 | 0.099725 | 0.07628 | 0 |
| 5.397876 | 0.376736 | 0.054965 | 0.088888 | 0.038323 | 0.597145 | 0.147287 | 0.079886 | 0.016413 |
| 0.621337 | 7.567951 | 0.49625 | 3.048619 | 0.674686 | 0.280913 | 0.518609 | 0.234404 | 0.066682 |
| 15.15662 | 2.434704 | 0.19051 | 1.080084 | 0.149429 | 0.130718 | 0.382871 | 0.193242 | 0.204794 |
| 11.45815 | 2.690636 | 0.101854 | 1.752472 | 0.142028 | 0.401848 | 0.614093 | 0.231302 | 0.022811 |
| 5.324786 | 0.991806 | 0.555027 | 0.413297 | 0.132676 | 0.040803 | 0.089236 | 0.048976 | 0.034094 |
| 1.597607 | 3.508539 | 0.132101 | 3.414947 | 0.069077 | 0.016995 | 0.066372 | 0.479986 | 0.825412 |
| 1.840317 | 12.82871 | 0.102559 | 0.316505 | 0.08833 | 0.65817 | 0.19399 | 0.039456 | 0.029183 |
| 8.471375 | 1.64553 | 2.160731 | 1.543965 | 1.004328 | 0.988386 | 2.894978 | 0.697862 | 0.645207 |
| 0.071919 | 3.650163 | 0.036105 | 0.79435 | 0.03776 | 0.092901 | 0.885255 | 0.150866 | 0.160102 |
| 5.08491 | 7.012077 | 0.253328 | 3.713419 | 0.305695 | 0.030084 | 0.39163 | 0.084965 | 0.031422 |
| 5.36637 | 6.542149 | 0.038565 | 0.319808 | 0.201663 | 0 | 0.077506 | 0.014013 | 0.010364 |
| 5.961322 | 0.754588 | 0.094366 | 0.415226 | 0.065793 | 0.016187 | 2.553944 | 0.245728 | 0.076081 |
| 9.471679 | 1.300795 | 0.51907 | 0.14275 | 0.108571 | 0 | 0.036511 | 0.094301 | 0.0279 |
| 25.09262 | 0.744426 | 0.27006 | 0.210637 | 0.024559 | 0.02417 | 0.047195 | 0.068261 | 0.044177 |
| 10.84646 | 0.524924 | 0.157813 | 0.169674 | 0.038834 | 0.009554 | 0.223878 | 0.070833 | 0.034927 |
| 11.2884 | 0.329206 | 0.122959 | 0.957224 | 0.096445 | 0.007909 | 0.049423 | 0.094938 | 0.037175 |
| 1.803847 | 19.82622 | 0.66458 | 1.251607 | 0.071285 | 0.210461 | 0.178083 | 0.074299 | 0.009159 |
| 6.321412 | 10.53735 | 0.47671 | 0.962746 | 0.166184 | 0.016355 | 0.089418 | 0.075058 | 0.017082 |
| 9.234276 | 1.405326 | 0.199494 | 6.887413 | 0.27818 | 0.18251 | 0.267285 | 0.225511 | 0 |
| 13.71648 | 2.45637 | 0.179191 | 1.522354 | 0.224882 | 0.092214 | 0.17286 | 0.104174 | 0.028894 |
| 0.012541 | 6.003462 | 2.192503 | 2.002567 | 0.138269 | 0.170093 | 0.531415 | 0.33627 | 0.124359 |
| 6.032483 | 0.626299 | 0 | 0.565551 | 0.436861 | 0.017914 | 0.209876 | 0.139129 | 0.009355 |
| 7.471615 | 0.500488 | 0.407379 | 0.775291 | 0.048232 | 0.023733 | 0.271876 | 0.050271 | 0.082627 |
| 15.01619 | 0.519964 | 0.037931 | 0.100142 | 0.095206 | 0.007808 | 0.067083 | 0.088206 | 0.004078 |
| 6.02245 | 2.135359 | 0.105609 | 0.157282 | 0.441793 | 0.043478 | 0.084898 | 0.046047 | 0.034058 |
| 2.907848 | 0.881146 | 0.586754 | 1.729392 | 2.564875 | 0.956739 | 0.980468 | 0.3689 | 0.815013 |
| 5.828695 | 3.5557 | 0.234285 | 1.013899 | 0.315026 | 0 | 0.255603 | 0.072966 | 0.026984 |
| 4.610926 | 2.216641 | 0.376548 | 0.104839 | 0.063516 | 0.006251 | 0.073234 | 0.039721 | 0.022851 |
| 12.65681 | 1.128612 | 0.348699 | 1.376967 | 0.04052 | 0 | 0.155731 | 0.084466 | 0.010412 |
| 6.213628 | 0.610045 | 0.071204 | 3.534775 | 0.099289 | 0.024428 | 0.391141 | 0.310461 | 0.191358 |
| 1.725555 | 2.424482 | 0.370574 | 1.208687 | 0.281858 | 0.277384 | 0.189573 | 0.061203 | 0 |
| 0.123598 | 33.22105 | 1.776875 | 14.46765 | 0.247772 | 0.14224 | 0.412652 | 0.121951 | 0.021223 |
| 10.58039 | 12.8325 | 0.131716 | 0.133748 | 0.354218 | 0 | 0.226896 | 0.041022 | 0 |
| 16.4877 | 2.095251 | 0.073904 | 1.282573 | 0.070264 | 0.034574 | 0.253846 | 0.153794 | 0.075835 |

| | | | | | | | | |
|---|---|---|---|---|---|---|---|---|
| 6.307126 | 3.254968 | 0.078821 | 1.043145 | 0.329731 | 0.170361 | 0.361171 | 0.117422 | 0.279613 |
| 16.04602 | 4.425113 | 0.042568 | 0.163294 | 0.178076 | 0.03651 | 0.108364 | 0.020623 | 0.041947 |
| 0.400399 | 1.501221 | 0.033186 | 0.82447 | 0.041648 | 0.034156 | 0.229429 | 0.069936 | 0.057079 |
| 4.824538 | 19.75416 | 0.14313 | 1.431352 | 0.13608 | 0.060264 | 0.355641 | 0.229298 | 0.353183 |
| 0.648936 | 8.853267 | 0.285061 | 1.013102 | 0.074531 | 0.183369 | 0.544249 | 0.116523 | 0 |
| 2.346878 | 1.957131 | 0.207668 | 1.420868 | 0.558472 | 0.061068 | 0.178866 | 0.215587 | 0.015946 |
| 14.81766 | 3.352435 | 0.225746 | 0.764093 | 0.25183 | 0.01549 | 0.133082 | 0.071088 | 0.024267 |
| 0.012744 | 25.73358 | 0.694173 | 0.970156 | 0.078062 | 0.007682 | 0.480032 | 0.073227 | 0.036108 |
| 5.346819 | 0.777405 | 0.418791 | 0.48195 | 0.437981 | 0.229882 | 0.359106 | 0.180064 | 0.589002 |
| 5.527088 | 4.509112 | 0.224863 | 0.8043 | 0.050847 | 0.018765 | 0.244276 | 0.04858 | 0.062064 |
| 0.969595 | 6.857761 | 0.440605 | 0.596534 | 0.230397 | 0.234298 | 0.135776 | 0.160093 | 0.043417 |
| 6.830816 | 1.018353 | 1.937957 | 0.485402 | 0.054047 | 0 | 0.145404 | 0.070415 | 0.034721 |
| 3.841424 | 2.776666 | 0.114835 | 2.319169 | 0.4537 | 0.026265 | 0.174372 | 0.069542 | 0.027432 |
| 0.278192 | 28.25536 | 0.061101 | 1.220193 | 0 | 0.015722 | 0.01842 | 0.238661 | 0.168312 |
| 1.166731 | 2.164139 | 0.530819 | 1.716145 | 0.153143 | 0.169551 | 0.26486 | 0.119713 | 0.019677 |
| 0.194781 | 72.85007 | 0.209153 | 1.982198 | 0.198851 | 0.156556 | 0.244561 | 0.089812 | 0 |
| 13.55088 | 0.603633 | 0.289371 | 0.824013 | 0.210526 | 0.025898 | 0.171938 | 0.185142 | 0.121723 |
| 8.453665 | 2.353093 | 0.097509 | 0.981881 | 0.169962 | 0.476703 | 0.150241 | 0.135814 | 0.078615 |
| 11.86905 | 0.626938 | 0.071391 | 0.516506 | 0.466639 | 0.440862 | 0.351516 | 0.181578 | 0.071947 |
| 9.448394 | 1.423881 | 0.070721 | 0.939537 | 0.147923 | 0.018197 | 0.312685 | 0.167026 | 0.019006 |
| 0.681854 | 0.582768 | 0.780687 | 3.4587 | 0.242513 | 0.270485 | 0.360396 | 0.337022 | 0.083091 |
| 7.690599 | 5.331729 | 0.355609 | 2.542699 | 0.185952 | 0.04575 | 0.500272 | 0.258419 | 0 |
| 1.665766 | 0.458917 | 1.377371 | 0.284903 | 0.080027 | 0.039378 | 0.153785 | 0.027804 | 0.010282 |
| 7.724335 | 0.168449 | 0.172036 | 0.05823 | 0.115662 | 0 | 0.014818 | 0.040184 | 0 |
| 0.881556 | 2.994842 | 0.828331 | 3.813016 | 0 | 1.892629 | 0.40619 | 0.126408 | 0.133566 |
| 10.0242 | 3.439301 | 0.127036 | 0.070947 | 0.119571 | 0.006537 | 0.071486 | 0.016155 | 0.01707 |
| 0.678837 | 7.591439 | 0.586925 | 0.769002 | 0.039601 | 0.038973 | 0.0761 | 0.048155 | 0.020353 |
| 2.168038 | 1.47752 | 0.347484 | 1.393949 | 0.619136 | 0.052983 | 0.486256 | 0.434886 | 0.276695 |
| 14.4818 | 0.717219 | 0.007217 | 0.092821 | 0.045284 | 0 | 0.058014 | 0.047199 | 0.023273 |
| 7.364978 | 0.982417 | 0.027302 | 0.900989 | 0 | 0.028099 | 0.197527 | 0.238079 | 1.210637 |
| 0.024712 | 16.54204 | 2.648664 | 1.452533 | 0.030274 | 0.074483 | 0.511946 | 0.157768 | 0.26061 |
| 13.90321 | 0.826405 | 0.029528 | 0.059966 | 0.267633 | 0.111432 | 0.142422 | 0.032186 | 0 |
| 0.845249 | 33.15261 | 0.423379 | 1.953923 | 0.028113 | 0.152167 | 0.189083 | 0.065929 | 0.003612 |
| 15.08975 | 1.285071 | 0.106189 | 0.103334 | 0.069409 | 0.013662 | 0.026676 | 0.057875 | 0.010702 |
| 2.881454 | 3.727867 | 0.5039 | 2.777647 | 0 | 0.024696 | 0.353641 | 0.066843 | 0.055888 |
| 7.844851 | 7.996006 | 0.467386 | 1.795423 | 0.06207 | 0.045814 | 0.27434 | 0.099737 | 0.119626 |
| 4.460221 | 34.59456 | 0.064502 | 0.916961 | 0.101187 | 0.016597 | 0.220374 | 0.041014 | 0.043337 |
| 1.025387 | 0.309964 | 1.693168 | 0.649017 | 0.090808 | 0 | 0.139602 | 0.060469 | 0.18279 |
| 8.908115 | 6.59074 | 0.060099 | 0.858882 | 0.069836 | 0.041237 | 0.171779 | 0.11161 | 0.050248 |
| 6.837045 | 4.589235 | 0.485192 | 1.596268 | 0.243564 | 0 | 0.358837 | 0.260914 | 0.104315 |
| 2.768189 | 21.40055 | 1.165328 | 1.054681 | 0 | 0.07822 | 0.224016 | 0.294552 | 0.040849 |
| 1.674375 | 6.376651 | 0.194837 | 2.321347 | 0.163012 | 0.100265 | 0.187953 | 0.084952 | 0.010472 |
| 0.672632 | 7.268624 | 1.246013 | 0.387632 | 0.095817 | 0.047148 | 0.117842 | 0.09321 | 0.083715 |
| 10.63284 | 5.981044 | 0.254099 | 0.681257 | 0.321688 | 0.605634 | 0.564422 | 0.26483 | 0.575056 |
| 22.54725 | 0.69672 | 0.078193 | 0.146447 | 0.054517 | 0.005365 | 0.096383 | 0.04167 | 0.014009 |
| 11.53003 | 0.911087 | 0.013634 | 0.470689 | 0.171099 | 0 | 0.197277 | 0.13375 | 0 |
| 10.66213 | 0.397059 | 0.056175 | 1.868109 | 0.014687 | 0.021681 | 0.197569 | 0.043374 | 0.022645 |
| 22.20084 | 1.263564 | 0.180256 | 0.826436 | 0.017138 | 0.008433 | 0.164666 | 0.130991 | 0.030827 |
| 9.371984 | 16.25374 | 0.143071 | 0.28087 | 0.149627 | 0.014725 | 0.230027 | 0.031191 | 0.03076 |
| 7.709387 | 1.663535 | 0.015169 | 0.354274 | 0.015864 | 0 | 0.134138 | 0.038582 | 0.004077 |
| 1.178624 | 12.29032 | 0.050511 | 1.230965 | 0.211303 | 0.36391 | 0.203027 | 0.183531 | 0.081448 |
| 7.667936 | 1.549735 | 0.566319 | 0.400534 | 0.371273 | 0.052197 | 0.326154 | 0.285621 | 0.140838 |
| 4.739979 | 5.033737 | 0.015626 | 1.343398 | 0.130735 | 0.096495 | 0.138176 | 0.056776 | 0.025196 |
| 3.416028 | 5.039198 | 0.107184 | 1.811682 | 0.084071 | 0.041368 | 0.210024 | 0.148478 | 0.108019 |
| 6.296448 | 4.200221 | 0.080488 | 1.315104 | 0.091829 | 0.08284 | 0.099996 | 0.069125 | 0.003933 |
| 4.083953 | 0.674004 | 0.063786 | 1.191041 | 0.022236 | 0 | 0.094007 | 0.173823 | 0.034284 |
| 12.98794 | 5.200814 | 0.157486 | 1.434384 | 0.089837 | 0.014735 | 0.230183 | 0.15606 | 0.053866 |
| 14.907 | 0.60738 | 0.074624 | 0.587256 | 0.351196 | 0.076805 | 0.269953 | 0.04745 | 0.010027 |

| | | | | | | | | |
|---|---|---|---|---|---|---|---|---|
| 6.153485 | 2.920354 | 0.256563 | 2.72615 | 0.095828 | 0.103738 | 0.235712 | 0.129844 | 0.132975 |
| 18.49463 | 4.519185 | 0.050396 | 3.044795 | 0.158115 | 0 | 0.283588 | 0.094608 | 1.295674 |
| 14.26628 | 3.872817 | 0.037358 | 0.973645 | 0.11721 | 0.057675 | 0.240254 | 0.434366 | 0 |
| 4.890198 | 2.415766 | 0.074202 | 0.1306 | 0.124163 | 0 | 0.09544 | 0.010784 | 0.02393 |

| LCK | FCGR3B |
|---|---|
| 5.29689 | 0.088972 |
| 10.5199 | 0.076621 |
| 2.106202 | 0.414731 |
| 4.079326 | 0.424834 |
| 3.224697 | 0.293383 |
| 2.893979 | 0.827778 |
| 3.331789 | 0.176456 |
| 3.163134 | 0.057442 |
| 4.856331 | 0.791505 |
| 5.867964 | 0.319521 |
| 3.802374 | 1.032877 |
| 1.81129 | 0.250016 |
| 8.764807 | 0.340125 |
| 1.923073 | 0.562864 |
| 3.685718 | 0.06437 |
| 6.214293 | 0.285992 |
| 2.942588 | 0.201229 |
| 5.064275 | 5.958618 |
| 1.935035 | 1.123995 |
| 3.498749 | 0.043482 |
| 3.910743 | 1.124975 |
| 3.854576 | 0.086953 |
| 2.386995 | 2.195774 |
| 12.87906 | 0.429941 |
| 1.494688 | 0.238607 |
| 1.290454 | 2.663533 |
| 3.846793 | 2.731662 |
| 5.94921 | 0.52667 |
| 2.83216 | 0.024553 |
| 2.698766 | 1.06154 |
| 3.319005 | 0.33794 |
| 3.454723 | 0.256507 |
| 11.39249 | 0.380536 |
| 3.555751 | 0.16069 |
| 6.127655 | 0.156945 |
| 5.441966 | 0.05475 |
| 0.436438 | 0.053433 |
| 3.866561 | 0.94867 |
| 3.916167 | 0.407129 |
| 2.690379 | 0.575958 |
| 4.348809 | 1.390237 |
| 2.83058 | 0.436134 |
| 1.669897 | 0.531256 |
| 1.531959 | 0.034678 |
| 7.594221 | 0.2402 |
| 3.226438 | 2.956234 |
| 0.813213 | 4.103522 |
| 1.116736 | 0.073539 |
| 9.760698 | 1.584237 |
| 5.133745 | 0.044697 |
| 1.132749 | 0.031292 |
| 3.253036 | 7.213183 |
| 5.612615 | 0.093568 |
| 3.956419 | 4.194546 |
| 4.816913 | 0.424368 |
| 2.035339 | 2.224156 |

| | |
|---|---|
| 6.049667 | 1.359999 |
| 3.030614 | 6.599784 |
| 6.239052 | 0.126039 |
| 4.742485 | 2.388547 |
| 2.872202 | 0.102509 |
| 5.820675 | 1.581129 |
| 3.97371 | 0.694451 |
| 4.459226 | 0.006149 |
| 8.5172 | 0.223191 |
| 4.896743 | 1.663641 |
| 0.888274 | 0.902388 |
| 1.560463 | 4.74408 |
| 4.660829 | 1.043651 |
| 3.643911 | 0.052173 |
| 1.150631 | 0.23977 |
| 4.928376 | 2.446101 |
| 2.287081 | 0.251863 |
| 37.11717 | 2.38935 |
| 5.832054 | 1.948682 |
| 0.59132 | 0.113383 |
| 4.621318 | 8.312768 |
| 6.910794 | 3.091939 |
| 2.811439 | 8.740957 |
| 1.319259 | 0.018944 |
| 3.356301 | 1.596296 |
| 3.80771 | 0.135206 |
| 5.685766 | 3.133303 |
| 1.361326 | 21.15032 |
| 4.183572 | 0.920649 |
| 1.524117 | 0.02179 |
| 3.057182 | 0.011738 |
| 3.163804 | 0.857251 |
| 9.318275 | 4.830113 |
| 48.10652 | 2.136544 |
| 1.193545 | 0.38348 |
| 0.706901 | 0.11454 |
| 4.938145 | 0.184442 |
| 2.17376 | 0.892784 |
| 9.504688 | 2.078078 |
| 1.434607 | 0.11608 |
| 2.177295 | 0.100978 |
| 0.854984 | 0.037918 |
| 3.720404 | 0.186997 |
| 2.151177 | 0.541061 |
| 2.08145 | 0.033555 |
| 2.946596 | 0.040021 |
| 6.1363 | 1.896959 |
| 13.64961 | 0.267945 |
| 2.864747 | 0.352169 |
| 1.789341 | 0.023036 |
| 1.110124 | 0.202413 |
| 3.965001 | 2.56369 |
| 3.814019 | 0.59019 |
| 1.1285 | 0.071428 |
| 2.194924 | 0.005219 |
| 4.447825 | 1.498789 |
| 3.998067 | 0.110444 |
| 5.89653 | 0.411064 |

| | |
|---|---|
| 4.337715 | 0.198528 |
| 49.36146 | 6.140244 |
| 1.657145 | 0.04207 |
| 0.342447 | 0.046511 |
| 0.202678 | 0.127051 |
| 3.788424 | 2.657987 |
| 4.958321 | 5.817085 |
| 1.774127 | 0.971559 |
| 0.460472 | 0 |
| 4.806601 | 1.889779 |
| 0.97833 | 0.307378 |
| 8.150091 | 0.022476 |
| 7.219289 | 0.350617 |
| 8.577438 | 9.285682 |
| 4.899559 | 6.442539 |
| 0.799377 | 0.117954 |
| 1.553571 | 0.610037 |
| 0.83191 | 12.19191 |
| 20.2707 | 0.062683 |
| 1.550061 | 0.007797 |
| 3.688896 | 6.138446 |
| 2.467883 | 0.225547 |
| 3.939434 | 0.511096 |
| 1.246566 | 0.071288 |
| 0.173877 | 0.037786 |
| 52.43961 | 0.044748 |
| 2.576674 | 0.296939 |
| 2.801716 | 0.005369 |
| 7.017178 | 3.500823 |
| 0.833422 | 1.399506 |
| 3.359307 | 0.188298 |
| 0.994643 | 0.119346 |
| 31.64291 | 1.344848 |
| 2.964041 | 0.101551 |
| 59.81847 | 0.218362 |
| 1.369332 | 0.061528 |
| 1.90726 | 0.013908 |
| 91.91215 | 0.208875 |
| 5.766113 | 0.515576 |
| 2.482947 | 0.03231 |
| 5.189215 | 0.245148 |
| 5.193064 | 0.204145 |
| 1.884022 | 0.741704 |
| 4.321279 | 2.142835 |
| 8.374223 | 0.236545 |
| 6.189579 | 0.290212 |
| 3.310705 | 2.495367 |
| 1.054188 | 0.083003 |
| 3.361633 | 0.653626 |
| 4.509391 | 0.153757 |
| 3.702139 | 4.829742 |
| 6.547189 | 2.704851 |
| 4.057248 | 2.057274 |
| 21.778 | 1.380745 |
| 6.211772 | 2.992534 |
| 8.870491 | 8.015616 |
| 2.720956 | 0.669392 |
| 2.268943 | 0.170024 |

| | |
|---|---|
| 1.646337 | 0.062766 |
| 4.870375 | 1.785763 |
| 2.563783 | 0.310385 |
| 5.096897 | 2.300956 |
| 4.527142 | 0.352503 |
| 2.318069 | 2.106568 |
| 13.69662 | 1.612097 |
| 5.556466 | 18.40602 |
| 1.094217 | 0.924726 |
| 2.863447 | 0.984189 |
| 1.208621 | 2.259337 |
| 2.624415 | 0.058196 |
| 1.9456 | 0.635922 |
| 1.336174 | 2.018152 |
| 4.439473 | 0.530917 |
| 1.809345 | 0.527163 |
| 3.118508 | 0.071443 |
| 10.83204 | 0.493339 |
| 1.053772 | 4.279388 |
| 0.409243 | 0.039235 |
| 1.398946 | 0.989336 |
| 2.065524 | 2.834659 |
| 4.140837 | 1.454471 |
| 3.302377 | 0.14951 |
| 4.108638 | 1.796235 |
| 5.6846 | 0.316752 |
| 6.978217 | 6.02437 |
| 5.806522 | 4.550537 |
| 4.359213 | 0.29116 |
| 3.359818 | 2.81603 |
| 2.203314 | 0.700205 |
| 2.106854 | 1.315086 |
| 3.780034 | 0.680006 |
| 3.912753 | 0.189136 |
| 2.760479 | 0.399924 |
| 3.949287 | 0.288894 |
| 2.233142 | 0.485289 |
| 2.617217 | 5.429005 |
| 2.239327 | 0.344084 |
| 0.211647 | 0.431188 |
| 1.149895 | 0.208238 |
| 1.738915 | 0.177639 |
| 0.836021 | 0.07899 |
| 1.600422 | 1.117401 |
| 1.828492 | 0.082292 |
| 1.284976 | 0.110227 |
| 6.49553 | 0.017913 |
| 3.221684 | 0.148608 |
| 15.79946 | 0.059381 |
| 1.726902 | 2.358509 |
| 8.034746 | 1.375674 |
| 2.450141 | 1.909011 |
| 7.304445 | 1.788801 |
| 0.225351 | 0.714167 |
| 0.818284 | 0.037046 |
| 4.329564 | 0.307156 |
| 6.803964 | 2.367395 |
| 18.24128 | 5.857172 |

| | |
|---|---|
| 1.664673 | 0.043585 |
| 2.736008 | 0.843044 |
| 1.680504 | 0.250225 |
| 2.495675 | 0 |
| 3.321717 | 1.91884 |
| 85.54163 | 0.116767 |
| 4.078395 | 0.324745 |
| 1.690325 | 1.079944 |
| 1.66941 | 0.129565 |
| 2.802101 | 0.27527 |
| 5.381891 | 5.951381 |
| 1.906491 | 0.332075 |
| 1.749627 | 0.520729 |
| 1.333669 | 1.076311 |
| 5.191329 | 1.100623 |
| 1.652834 | 0.076967 |
| 1.772392 | 1.440683 |
| 2.834277 | 2.984367 |
| 1.472033 | 1.415607 |
| 2.359831 | 1.296854 |
| 4.116912 | 1.593689 |
| 0.747746 | 0.113746 |
| 2.536044 | 0.108254 |
| 17.55512 | 3.542262 |
| 4.031801 | 1.280303 |
| 4.874999 | 1.098483 |
| 3.035085 | 0.089736 |
| 4.645677 | 0.701864 |
| 1.741745 | 0.096401 |
| 1.245732 | 0.009634 |
| 2.438805 | 0.748887 |
| 2.671741 | 10.8009 |
| 2.070911 | 0.104054 |
| 5.073013 | 0.061398 |
| 2.271198 | 0.095792 |
| 7.762419 | 2.772422 |
| 5.817168 | 0.019404 |
| 5.379115 | 0.36484 |
| 1.254535 | 0.523688 |
| 0.883471 | 0.159991 |
| 22.21766 | 0.418744 |
| 2.045779 | 0.168825 |
| 1.155513 | 7.219326 |
| 5.154993 | 0.307239 |
| 5.117721 | 0.767797 |
| 1.200811 | 0.342498 |
| 0.591933 | 0.091882 |
| 1.667626 | 0.175789 |
| 1.510897 | 0.436396 |
| 5.208085 | 1.13178 |
| 6.715377 | 0.190762 |
| 2.235872 | 0.222882 |
| 3.450481 | 0.30997 |
| 4.497164 | 0.063296 |
| 5.776582 | 1.456928 |
| 11.81742 | 1.945505 |
| 2.15815 | 0.284692 |
| 5.3935 | 1.195649 |

| | |
|---|---|
| 1.864141 | 0.134182 |
| 0.785491 | 0.108379 |
| 3.16071 | 2.843455 |
| 18.68762 | 0.056314 |
| 1.780842 | 0.239571 |
| 48.40137 | 0.14517 |
| 4.122676 | 0 |
| 0.621961 | 0.207938 |
| 1.896558 | 0.078611 |
| 1.322155 | 0.894611 |
| 4.498256 | 1.635573 |
| 5.370269 | 0.59263 |
| 7.840378 | 1.710027 |
| 17.32566 | 0.768194 |
| 4.626873 | 1.273309 |
| 3.755436 | 0.025623 |
| 3.601596 | 0.093453 |
| 11.7196 | 0.764943 |
| 5.994933 | 0.066752 |
| 1.685415 | 0.03423 |
| 3.442939 | 1.14131 |
| 3.478712 | 0.702156 |
| 2.871708 | 0.00724 |
| 9.517217 | 0.698933 |
| 1.871447 | 0.123239 |
| 1.38692 | 0.119302 |
| 4.574249 | 0.970434 |
| 1.185898 | 0.106855 |
| 1.66895 | 0.500776 |
| 1.846464 | 0.130038 |
| 0.481705 | 0 |
| 0.398609 | 0.277968 |
| 0.438772 | 0.027772 |
| 2.562857 | 0.084085 |
| 2.452517 | 0.585979 |
| 3.75843 | 0.027045 |
| 3.289224 | 1.826278 |
| 0.714431 | 0.243714 |
| 7.693089 | 0.064177 |
| 47.74744 | 0.09263 |
| 4.088768 | 0.865672 |
| 1.665885 | 0.452521 |
| 0.896185 | 0.280122 |
| 4.37131 | 0.18704 |
| 6.204775 | 1.931996 |
| 1.218015 | 0.14705 |
| 2.511405 | 1.699904 |
| 13.37607 | 1.6254 |
| 4.984114 | 2.297909 |
| 6.197592 | 6.392646 |
| 1.870731 | 0.236815 |
| 7.964431 | 0.148594 |
| 2.99581 | 1.162546 |
| 4.8988 | 1.530048 |
| 0.462522 | 0.006055 |
| 17.77391 | 0.282911 |
| 1.481041 | 0.139934 |
| 1.652407 | 0.430906 |

| | |
|---|---|
| 3.323926 | 1.117162 |
| 21.58028 | 19.52075 |
| 9.57474 | 0.115424 |
| 1.500048 | 0.004478 |
| 4.616558 | 0.693412 |
| 3.001612 | 0.369219 |
| 0.947536 | 0 |
| 3.276182 | 1.050004 |
| 1.115815 | 0.068497 |
| 0.770103 | 1.012924 |
| 1.299476 | 0.220364 |
| 5.272788 | 3.882743 |
| 4.412509 | 1.311691 |
| 2.198903 | 0.380251 |
| 2.062345 | 0.265245 |
| 9.81489 | 1.469968 |
| 0.89994 | 0.764367 |
| 3.307817 | 0.513449 |
| 2.608108 | 1.082022 |
| 6.023161 | 1.594885 |
| 1.561514 | 0.278559 |
| 0.8239 | 3.792466 |
| 2.118768 | 0.012289 |
| 14.07247 | 0.030673 |
| 2.686507 | 0.762934 |
| 48.57256 | 2.291788 |
| 3.716619 | 0.257388 |
| 1.190555 | 0.841873 |
| 1.85347 | 0.907857 |
| 2.133197 | 0.640167 |
| 3.521399 | 1.419112 |
| 4.874245 | 1.390158 |
| 4.497154 | 6.976736 |
| 0.70708 | 0.051219 |
| 6.701471 | 0.874068 |
| 4.14055 | 0.275446 |
| 4.415388 | 2.343806 |
| 17.0017 | 0.865366 |
| 3.204008 | 0.723397 |
| 2.175168 | 0.709036 |
| 3.803517 | 0.389755 |
| 1.855249 | 0.141855 |
| 0.640187 | 0.130797 |
| 3.018129 | 3.529613 |
| 2.103016 | 0.126654 |
| 1.450272 | 0.814548 |
| 3.611726 | 0.47903 |
| 2.295136 | 1.067906 |
| 10.38189 | 1.347437 |
| 3.055954 | 0.114253 |
| 0.812164 | 1.843371 |
| 5.707278 | 2.848778 |
| 2.36644 | 0.233753 |
| 0.594117 | 0.134488 |
| 0.656502 | 2.389956 |
| 2.833901 | 0.862854 |
| 2.083173 | 0.997472 |
| 1.972781 | 0.459331 |

| | |
|---|---|
| 4.962985 | 0.634422 |
| 11.3528 | 5.719577 |
| 1.691034 | 0.976992 |
| 1.184976 | 0.426449 |
| 3.293239 | 0.061847 |
| 1.527673 | 0.306808 |
| 0.743381 | 0.076927 |
| 3.2135 | 1.252734 |
| 2.810223 | 1.755516 |
| 2.389869 | 0.820667 |
| 0.462645 | 0.213263 |
| 4.124908 | 0.260855 |
| 1.249393 | 0.093623 |
| 1.08664 | 0.224431 |
| 4.396333 | 1.128397 |
| 4.090896 | 1.866332 |
| 1.7753 | 0.284112 |
| 6.149867 | 18.57874 |
| 2.810217 | 0.143131 |
| 2.986444 | 0.10372 |
| 16.35025 | 0.32301 |
| 19.99561 | 0.701906 |
| 11.25055 | 0.14964 |
| 0.505524 | 0.017719 |
| 2.379565 | 0.0567 |
| 7.250739 | 19.81109 |
| 6.267048 | 0.515873 |
| 4.714052 | 1.686354 |
| 0.995907 | 0.11042 |
| 1.636667 | 9.443588 |
| 1.360859 | 0.151226 |
| 5.948966 | 2.006041 |
| 18.04737 | 0.427388 |
| 4.970151 | 6.775337 |
| 1.713509 | 0.089511 |
| 1.437916 | 0.116814 |
| 0.388078 | 0.036143 |
| 1.053432 | 0.239825 |
| 5.155773 | 0.025856 |
| 2.311363 | 0.927519 |
| 10.55616 | 1.455492 |
| 1.670026 | 1.947359 |
| 2.727317 | 17.40994 |
| 2.051665 | 1.364173 |
| 6.363839 | 0.26595 |
| 4.134328 | 0.904903 |
| 4.045653 | 0.627978 |
| 1.361227 | 0.028172 |
| 2.273984 | 0.058829 |
| 3.77421 | 0.685525 |
| 2.488 | 0.093219 |
| 2.272939 | 0.073301 |
| 2.631848 | 2.428015 |
| 3.143406 | 2.412885 |
| 5.555521 | 0.813195 |
| 8.721479 | 24.69 |
| 2.138344 | 0.209634 |
| 2.65793 | 0.505244 |

| | |
|---|---|
| 4.680716 | 0.695192 |
| 3.509937 | 0.006587 |
| 2.319486 | 0.363559 |
| 3.452107 | 0.736896 |
| 3.988455 | 1.124776 |
| 4.790919 | 1.693893 |
| 4.526447 | 1.872291 |
| 14.49671 | 0.48509 |
| 2.475026 | 0.090722 |
| 3.136446 | 0.671432 |
| 1.920017 | 0.374973 |
| 4.128666 | 0.52777 |
| 4.088359 | 2.001974 |
| 3.6415 | 4.141072 |
| 11.65173 | 1.988262 |
| 3.899126 | 2.347802 |
| 1.225513 | 0.706679 |
| 2.298149 | 1.093886 |
| 1.227622 | 0.057995 |
| 2.432202 | 0.837139 |
| 5.435517 | 4.865468 |
| 3.342339 | 3.487213 |
| 12.12852 | 0.088803 |
| 1.648452 | 0.028521 |
| 5.923997 | 2.814649 |
| 0.75439 | 0.005897 |
| 2.653077 | 0.527328 |
| 3.749823 | 0.418195 |
| 0.561129 | 0.0201 |
| 1.119736 | 1.432111 |
| 17.01068 | 0.806243 |
| 1.093297 | 0.018276 |
| 17.54798 | 2.264823 |
| 1.451732 | 0.067779 |
| 7.456141 | 2.361404 |
| 1.673494 | 2.018096 |
| 7.178589 | 0.628788 |
| 4.964609 | 0.859871 |
| 1.16967 | 0.601361 |
| 2.089439 | 1.35137 |
| 3.073719 | 0.799666 |
| 1.631485 | 2.387728 |
| 2.418953 | 0.263685 |
| 4.022323 | 0.105538 |
| 0.873006 | 0.043557 |
| 2.539508 | 0.518956 |
| 2.513931 | 1.623277 |
| 1.547194 | 0.631371 |
| 3.42291 | 0.172677 |
| 1.224846 | 0.190124 |
| 3.323253 | 0.234475 |
| 3.033337 | 0.039237 |
| 3.498096 | 1.479737 |
| 4.21854 | 1.816065 |
| 2.419558 | 2.499924 |
| 2.071062 | 0.829071 |
| 2.605631 | 1.408942 |
| 5.085048 | 0.259806 |

| | |
|---|---|
| 2.802873 | 3.326221 |
| 0.631555 | 1.083919 |
| 4.484828 | 0.346835 |
| 1.787921 | 0.137779 |
