# Supplementary material for: Alteration in the Immune Microenvironment Based on APC Status in MSS/pMMR Colon Cancer
Source: Dis Markers. 2022 Jul 27;2022:3592990. doi: 10.1155/2022/3592990 (PMC9348928; doi:10.1155/2022/3592990)
Supplement: Supplementary Materials — Supplement Table S1: all different expression genes (DEGs) listed between APC-wt and APC-mt MSS/pMMR colon cancer from the overall gene expression profile of all 338 colon cancer cases obtained from TCGA database. Supplementary Table S2: APC-mt vs. APC-wt MSS/pMMR colon cancer, 379 genes were downregulated, and 117 genes were upregulated. Supplementary Table S3: APC-wt MSS/pMMR (77 samples) COAD samples in the cohort retrieved from TCGA performed gene set enrichment analysis enriched (GSEA) in 115 KEGG pathways. Supplementary Table S4: APC-wt MSS/pMMR COAD samples in the cohort retrieved from TCGA performed gene set enrichment analysis downregulated (GSEA) in 62 KEGG pathways. Supplementary Table S5: 65 overlapping genes among the DEGs (496 genes related to APC status) and immunophenotypes (1297 genes shown in Supplementary Table S6). Supplementary Table S6: 1297 immune-related genes. Supplementary Table S7: Lasso and Cox regression analyze APC-related DEGs constructing immune scoring model. Supplement Figure 1: the expression of CTLA4, PD-L1, and immune score and stromal score in the KRAS-mt/MSS group was downregulated compared with KRAS-wt/MSS. Supplement Figure 2: the expression of not only PD-1, PD-L1, and CTLA4 but also immune score, ESTIMATE score, and stromal score did not differ between TP53-wt/MSS and TP53-mt/MSS groups. Supplement Figure 3: in the combination of different genotypes of KRAS, TP53 and APC, the TMB and immune score are significantly higher in wild type than KRAS/TP53/APC mutant type colon cancer. Supplement Figure 4: neither mutations in KRAS nor TP53 could affect the proportion of infiltrating immune cell types in MSS/pMMR colon cancer. [file 3592990.f1.zip › supplement table S7.pdf]

Lasso and Cox regression analyses APC related DEGs Constructing immune scoring model

id	futime	fustat	SSTR2	SCG2	CCL19	CD22	NFATC1	LHB	riskScore	risk
TCGA-AM-5820	0.038356164	0	0.09050476	4.712204	0.5379994	0.1628935	0.3849265	0	1.395827127	high
TCGA-F4-6854	0.043835616	0	0.08504743	1.416936	4.032803	0.177929	0.5513765	0	1.151058504	low
TCGA-AM-5821	0.076712329	0	0.05500639	2.497458	2.192594	1.059692	1.121955	0.3211569	1.781764814	high
TCGA-AA-A020	0.076712329	0	0.01486308	0.9299766	0.7509969	0.06316226	0.3775192	0.2975269	1.42261907	high
TCGA-AA-3818	0.082191781	1	0.0721406	1.083314	0.5832155	0.08583939	0.2082223	0	1.09351712	low
TCGA-AA-3856	0.082191781	0	0.1866675	0.4207324	8.879764	0.7313504	0.7569285	0	1.134490727	low
TCGA-AA-3821	0.084931507	0	0.1842426	0.3350011	0	0.1652913	1.943976	1.741621	5.563687347	high
TCGA-AA-3494	0.084931507	0	0.04589694	0.3829003	0.7214871	0.0585132	0.1987113	0.07656316	1.116887874	low
TCGA-AA-A01V	0.084931507	0	0.01648716	0.09522412	1.666116	0.07707037	0.3426304	0.08250937	1.114377303	low
TCGA-AA-3496	0.084931507	0	0.1396321	0.4320357	3.628432	0.2670218	0.6023796	0.224609	1.333700564	high
TCGA-AA-A01S	0.084931507	0	0.0707625	0.01571922	0	0.01387906	0.1649668	0.3268876	1.35700971	high
TCGA-AZ-4323	0.117808219	1	7.727598	23.16609	225.7216	23.12822	7.163986	1.841546	163.1840196	high
TCGA-5M-AAT4	0.134246575	1	0.07889829	5.346753	0.1328849	0.04358728	0.1776273	0.1974217	1.69347759	high
TCGA-A	0.161	1	0.057	0.041	0.243	0.012	0.091	0.181	1.187	lo

Z-6608	643836		8754	78352	6924	29739	87645	022	749482	w
TCGA-AA-A02H	0.167123288	1	0.2302005	0.9417702	1.550866	0.1222827	0.2823857	0.5760147	1.828277909	high
TCGA-AA-3666	0.167123288	1	0.08446584	0.5488261	0.5690483	0.134605	0.9386191	0	1.116911907	low
TCGA-CK-6748	0.167123288	0	0.04858025	2.404083	0.1785198	0.2167693	0.5296459	0.03315245	1.258718326	high
TCGA-AA-3952	0.167123288	1	0.05590697	0.9099889	0.2568045	0.123111	1.016609	0.2289144	1.404875469	high
TCGA-AA-3930	0.167123288	1	0.167048	0.9165708	3.629435	0.2555599	0.4339417	0.1253978	1.244462464	low
TCGA-AA-3966	0.167123288	0	0.1906409	1.535711	3.853056	0.1790855	1.033899	0.1506402	1.375878192	high
TCGA-CA-5256	0.178082192	0	0.01078438	0.4204362	1.525747	0.04124645	0.09338219	0.08095504	1.113541748	low
TCGA-CA-5254	0.18630137	0	0.03945642	0.7216409	0.3101219	0.1425232	0.3492463	0.1316388	1.215607121	low
TCGA-AA-A02E	0.246575342	1	0.0251165	0.4170595	0.1269078	0.01601029	0.3914717	0.1885418	1.249230404	low
TCGA-CM-6680	0.249315068	0	0.1163091	1.479474	2.994384	0.268317	0.7745256	0.08315207	1.270576304	high
TCGA-CA-5255	0.254794521	0	0.02408509	0.9650558	0.334665	0.1496901	0.1147043	0.1355995	1.218081772	low
TCGA-AZ-6607	0.265753425	1	0.1508662	12.84295	5.336043	1.434162	2.25204	0.738591	5.463024122	high
TCGA-CA-5796	0.271232877	0	0.03594905	0.06920973	0.7669335	0.1273078	0.529182	0.05996854	1.108352634	low
TCGA-CA-5797	0.273972603	0	0.01965922	0.8799726	0.8443346	0.1691766	0.4085511	0.03689394	1.134613172	low

TCGA-AA-A00N	0.334246575	1	0.08495217	0.4702103	0.4292435	0.09927868	1.293435	0.4251399	1.658042472	high
TCGA-CM-6678	0.334246575	0	0.03016282	1.782302	0.5627288	0.04437006	0.2571623	0.1045029	1.253211155	high
TCGA-CM-4752	0.336986301	0	0.1391294	0.730512	4.154023	0.4031219	0.5256979	0	1.11556736	low
TCGA-CM-6677	0.419178082	0	0.1535654	0.7431123	2.264877	0.380973	0.5318904	0	1.115587461	low
TCGA-AA-3696	0.419178082	1	0	1.441199	0.3362179	0	0.4609464	0.9990109	2.740500283	high
TCGA-CM-5862	0.419178082	1	0.07554959	0.9765888	0.109067	0.03669213	0.1682191	0.05401214	1.133352886	low
TCGA-CM-6676	0.419178082	0	0.1340371	1.223159	0.677258	0.1435404	0.5106771	0.040247	1.173736931	low
TCGA-AZ-4616	0.42739726	1	0.1600925	4.969934	0.4044551	0.1258611	0.6792598	0.4406473	2.140746575	high
TCGA-AZ-6605	0.435616438	1	0.2031431	1.810938	22.49231	0.8482649	1.478597	0.1326028	1.479967059	high
TCGA-AZ-4614	0.471232877	1	0.08174621	0.3688576	0.05163057	0.0260542	1.349324	0.3835273	1.587831786	high
TCGA-DM-A285	0.490410959	1	0.1218723	2.075269	0.06370267	0.06429223	0.6932197	0.06309366	1.273785923	high
TCGA-CM-6168	0.495890411	0	0.2003194	2.862772	18.37473	1.424255	1.194185	0.07711467	1.517761412	high
TCGA-AA-3681	0.498630137	0	0.09136839	0.3957841	2.539147	0.7862666	0.5340335	0.2286248	1.36283404	high
TCGA-CM-6171	0.498630137	0	0.3370221	26.02663	0.3121973	0.3795361	0.2529177	0.08433071	5.761640687	high
TCGA-CM-4743	0.50410958	0	0.4303906	0.1625322	1.137516	0.06753198	1.146696	1.391733	3.82669663	high

	9								2	
TCGA-AA-3662	0.504109589	0	0.06684259	0.5119486	4.610886	1.004072	0.3749562	0.1308957	1.25869754	high
TCGA-F4-6570	0.515068493	1	0.1740733	2.460151	1.812409	0.3631466	5.280132	0.07919481	1.863723256	high
TCGA-AA-3663	0.580821918	0	0.1144679	0.2361168	0.594905	0.1250856	0.7164817	0.04910146	1.126659474	low
TCGA-CM-6165	0.580821918	0	0.1087556	1.745845	5.010302	0.3792503	0.7090578	0	1.201881843	low
TCGA-AA-3511	0.580821918	0	0.1116101	1.807742	0.7600928	0.3680974	0.4201887	0.1457083	1.342849349	high
TCGA-CM-6172	0.580821918	0	0.1358139	0.4518905	0.2386907	0.1430342	0.4141618	0.1773066	1.254177897	high
TCGA-AA-3489	0.58630137	1	0.377813	6.978782	61.48381	9.222164	2.677037	0.06917381	3.303685059	high
TCGA-CM-4744	0.58630137	0	0.04476603	0.08814308	1.850664	0.1219253	0.1779574	0.09164857	1.11357074	low
TCGA-DM-A1DA	0.624657534	1	0.06454791	0.1425436	0.4220707	0.07503002	0.1972665	0.02850243	1.055679178	low
TCGA-D5-6898	0.62739726	0	0.09501957	2.201893	2.271748	0.4520544	0.8729457	0.1043828	1.375913351	high
TCGA-DM-A280	0.646575342	1	0.06449069	0.2128433	0.1551697	0.05481203	0.5717221	0.1844235	1.248727257	low
TCGA-CM-6170	0.663013699	0	0.0433735	2.01145	5.723844	0.5366975	0.4573141	0	1.208039345	low
TCGA-AA-3989	0.663013699	1	0.0478836	0.6395428	2.05653	0.1907686	0.4560876	0	1.087915853	low
TCGA-AA-3980	0.663013699	0	0.1680242	0.2175146	1.053915	0.2289843	1.394722	0.2609599	1.434820608	high
TCGA-CM-6169	0.66575342	0	0.27814	0.9526563	3.202951	0.5030312	1.305028	0	1.20650640	low

	5								9	
TCGA-A6-3810	0.676712329	0	0.2584187	2.658581	2.285027	0.1750221	0.8391199	0	1.275789456	high
TCGA-G4-6295	0.695890411	0	0.0814131	0.1222556	16.09246	1.594247	0.7134172	0.04889145	1.210729189	low
TCGA-F4-6459	0.717808219	1	0.2646744	4.089185	2.0595	0.3981658	1.003818	0.07947316	1.532450319	high
TCGA-CM-5341	0.747945205	0	0.09685591	0.3573983	1.305041	0.08917992	0.4007014	0.08078528	1.139223054	low
TCGA-D5-6926	0.753424658	0	0.1421264	3.717606	1.24292	0.1742254	0.9301066	0.2462075	1.699669615	high
TCGA-A6-6650	0.761643836	0	0.05764232	0.3401594	0.4558746	0.04792641	0.2604136	0.03762636	1.080564394	low
TCGA-A6-3808	0.764383562	0	0.1961383	1.375575	14.15773	0.5179674	1.564922	0.4206713	1.834037027	high
TCGA-A6-6138	0.767123288	0	0.1349425	1.40451	6.363777	0.4193374	0.9931997	0.04220717	1.250222709	low
TCGA-A6-5659	0.783561644	0	0.1234035	2.218083	1.311562	0.1925875	0.3131968	0.06388636	1.260855506	high
TCGA-D5-6927	0.78630137	0	0.08496549	1.298393	0.8228458	0.2301821	0.6682772	0.2657541	1.460641653	high
TCGA-5M-AAT6	0.794520548	1	0.3362698	1.005771	17.59775	0.7298183	2.572061	1.442442	4.837807806	high
TCGA-CM-5863	0.832876712	0	0.05741704	1.45461	8.835318	0.1164545	0.8474579	0.03918291	1.216468859	low
TCGA-CM-5860	0.832876712	0	0.1054009	0.9334308	0.5325664	0.1388529	0.4441655	0.05274749	1.158645629	low
TCGA-CA-6718	0.838356164	1	0.1421226	1.706505	7.886636	0.5689988	2.048912	0.5615108	2.193539589	high
TCGA-A	0.838	1	0.087	0.304	0.975	0.179	0.456	0.922	2.414	hi

A-3848	356164		79901	258	9815	0934	1521	7122	072076	gh
TCGA-AA-3811	0.838356164	1	0.108603	0.9039822	1.807632	0.415368	0.6029593	0	1.132827587	low
TCGA-D5-6922	0.843835616	0	0.2113454	3.421956	1.607818	0.2361389	1.568903	0.03565179	1.456534162	high
TCGA-D5-7000	0.854794521	0	0.07226165	0.5703896	0.6572183	0.2395253	0.4734578	0.1446523	1.23648501	low
TCGA-A6-6649	0.898630137	0	0.1046474	1.727691	10.57518	0.883547	1.106179	0.2964361	1.650573205	high
TCGA-A6-3807	0.912328767	0	0.1754328	2.436598	3.123579	0.4260118	0.5208262	0.125421	1.38949904	high
TCGA-NH-A50U	0.915068493	1	0.07355898	2.543516	0.1173715	0.1850902	0.5062066	0.08718704	1.328654667	high
TCGA-AA-3680	0.917808219	1	0.06198283	2.729684	8.142805	0.5531457	1.32165	0	1.346075002	high
TCGA-CM-5861	0.917808219	0	0.1163387	0.4634013	0.04054015	0.04602976	1.705597	0.6022887	1.992673816	high
TCGA-F4-6461	0.926027397	1	0.2172451	2.318527	29.43078	7.136786	1.453104	0.1890773	2.227835698	high
TCGA-A6-5660	0.936986301	0	0.07768227	0.4641371	0.4060453	0.1092808	0.4778152	0.04021634	1.112628244	low
TCGA-D5-6932	0.947945205	0	0.1174913	0.5605737	2.581115	0.1432751	0.6214743	0.1150397	1.212691829	low
TCGA-A6-6137	0.953424658	0	0.05027089	0.1290432	1.749827	0.413021	0.5320293	0.1257894	1.198065823	low
TCGA-D5-5541	0.95890411	0	0.1592223	0.798275	2.055978	0.2029897	0.9287139	0.06640182	1.210012218	low
TCGA-D5-6928	0.969863014	0	0.3607207	1.554774	27.6116	4.361963	4.256336	0.2829065	2.446484316	high

TCGA-AZ-6606	0.978082192	1	0.07270345	0.1158372	0.5320294	0.2333189	0.1454456	0.07527758	1.103852983	low
TCGA-AY-6386	0.98630137	0	0.0413646	0.1433445	2.117924	0.147658	0.3008686	0	1.040613449	low
TCGA-A6-5666	0.991780822	0	0.08449654	0.1220057	0.2134707	0.1385012	0.08361798	0	1.023747867	low
TCGA-T9-A92H	0.991780822	0	0.08822559	1.086563	0.02622255	0.09593647	0.1482956	0.03895778	1.128359917	low
TCGA-D5-6931	1	0	0.2812718	1.591375	9.368326	0.7940174	0.5666355	0.1292707	1.359175692	high
TCGA-A6-5657	1.005479452	0	0.1535408	2.233023	48.24953	5.926638	1.816468	0.08331931	1.950168194	high
TCGA-AZ-6600	1.008219178	1	0.2802718	2.313527	10.65244	1.400796	1.290756	0.03723742	1.424156937	high
TCGA-CA-6716	1.016438356	0	0.02062277	2.382199	0.3647071	0.065729	0.2232162	0	1.184101471	low
TCGA-D5-5538	1.021917808	0	0.08642084	0.6626471	2.619986	0.3571235	0.6605858	0.08948064	1.20862751	low
TCGA-D5-6920	1.032876712	0	0.1443683	0.8816637	1.60242	0.2806049	0.4385556	0.1065963	1.221670922	low
TCGA-CA-6715	1.049315068	0	0.2117379	0.5039524	0.09405381	0.02076468	0.1370045	0.06986598	1.117307496	low
TCGA-A6-2684	1.060273973	0	0.1241943	2.940943	4.016159	0.2849996	0.4363056	0.7991043	2.567150193	high
TCGA-CA-6717	1.063013699	0	0.2041445	3.146836	45.77606	2.606119	2.541654	0.04141757	1.764192907	high
TCGA-NH-A6GC	1.065753425	0	0.05512546	5.717674	0.1856908	0.0292827	0.4773325	0.06896831	1.577573758	high
TCGA-CM-5348	1.07671232	0	0.2128273	1.829015	2.954019	0.4184362	1.860639	0.6159533	2.27316078	high

	9									
TCGA-AA-3939	1.082191781	0	0.1272217	1.392231	1.48864	0.1792656	0.243515	0.2010556	1.344773511	high
TCGA-AA-3973	1.087671233	0	0.06489947	0.6747063	2.0987	0.1737523	0.2866026	0.09743607	1.174071617	low
TCGA-F4-6809	1.104109589	1	0.2648298	6.455046	79.45262	2.64526	1.386419	0.109431	2.151727475	high
TCGA-DM-A1D7	1.109589041	1	0.05514463	0.2713116	1.011334	0.08592578	0.268814	0.06132648	1.101661021	low
TCGA-D5-6930	1.112328767	0	0.1096483	1.959242	22.16109	3.424822	2.162959	0	1.588878587	high
TCGA-F4-6460	1.115068493	0	0.2856212	2.669593	14.2766	1.311664	0.733982	0.04610905	1.40612861	high
TCGA-D5-6929	1.117808219	0	0.1360238	0.5065222	1.049032	0.2007955	0.6664055	0.05374152	1.15197531	low
TCGA-F4-6569	1.120547945	0	0.2292981	2.682825	14.93028	0.605752	1.930231	0.03549011	1.460293319	high
TCGA-D5-5540	1.126027397	0	0.05554307	0.3113623	1.023533	0.02915742	0.05375308	0.09810465	1.120487325	low
TCGA-F4-6463	1.136986301	0	0.2348989	7.018286	2.130315	0.2994685	0.5658678	0	1.653079628	high
TCGA-A6-2682	1.161643836	1	0.05784274	0.835199	1.636689	0.07374269	0.6411027	0.1736831	1.29385487	high
TCGA-AA-A004	1.161643836	0	0.04322404	0	5.387225	2.149117	1.609395	1.514191	4.807426653	high
TCGA-AA-3667	1.167123288	0	0.07057762	0.02911656	0.6113353	0.2056643	0.2881056	0	1.03591757	low
TCGA-AA-A02K	1.167123288	1	0.04772172	0.06890602	0.1929014	0.2129385	0.2500001	0.2507626	1.292833008	high
TCGA-C	1.167	0	0.083	1.662	1.172	0.156	0.513	0.197	1.385	hi

M-5868	123288		18532	608	461	2864	6901	1937	64551	gh
TCGA-DM-A288	1.169863014	1	0.03996021	0.4712091	0	0.2165144	0.1643577	0.2499742	1.316704639	high
TCGA-CM-6163	1.169863014	0	0.1317081	1.528577	4.570538	0.7160035	0.7681988	0.03730914	1.253127752	low
TCGA-D5-6924	1.191780822	0	0.3104612	2.017256	1.481539	0.6376802	0.7168774	0	1.2463942	low
TCGA-CA-6719	1.191780822	0	0.1174215	7.087866	2.286388	0.2665364	0.7985245	0.0859948	1.811732202	high
TCGA-AU-3779	1.208219178	0	0.09807677	0.2743778	1.672512	0.425905	0.7218615	0.184058	1.293078472	high
TCGA-A6-4105	1.210958904	1	0.2313015	0.5432729	2.773744	0.1415434	0.7263649	0.2315079	1.357909773	high
TCGA-AA-3844	1.243835616	0	0.06499185	0.1023737	1.671797	0.06779206	0.3120792	0.08870433	1.11976818	low
TCGA-AA-A01C	1.252054795	0	0.1127067	0.9402683	0.2531026	0.07982657	0.6939947	0	1.122664147	low
TCGA-AA-A017	1.252054795	0	0.09205439	0.8141261	0.8139764	0.1100236	0.5729163	0.086378	1.193462162	low
TCGA-AA-3679	1.252054795	0	0.05693008	0.2348631	1.972488	0.07257916	0.4929588	0.1221019	1.178073867	low
TCGA-A6-5656	1.265753425	0	0.1733165	0.2131793	0.356778	0.1063873	0.5197046	0.337305	1.433488105	high
TCGA-A6-2685	1.271232877	0	0.551995	2.721183	15.04425	0.9738468	1.390469	0	1.405977687	high
TCGA-AD-6888	1.293150685	1	0.02670162	0.05140642	0.06745852	0.04538856	0.05587571	0.0668136	1.072074925	low
TCGA-D5-6541	1.298630137	0	0.12747	2.133894	0.9057319	0.3300968	0.8881859	0.3887316	1.754590575	high

TCGA-CK-4952	1.301369863	0	0.0813295	0.32294	0.1284184	0.08100433	0.8714893	0.07631444	1.16918761	low
TCGA-AY-5543	1.312328767	0	0.03955567	0.1142299	3.530959	0.4202402	0.218828	0.1649624	1.212703833	low
TCGA-F4-6806	1.317808219	0	0.06590157	1.10619	1.956289	1.218243	0.4030167	0.06183785	1.244104926	low
TCGA-AA-3833	1.328767123	0	0.1973173	1.617548	4.438251	0.494998	4.574571	0.3822453	2.218391452	high
TCGA-A6-2686	1.336986301	0	0.2543952	0.6351654	12.854	0.4560805	1.514434	0.5172013	1.897313151	high
TCGA-AA-3971	1.339726027	0	0.4343657	0.8035826	3.566467	0.9258262	0.499532	0	1.159974654	low
TCGA-D5-6540	1.345205479	0	0.07163947	0.4882424	0.1158328	0.06210569	1.593002	0.3871983	1.635429962	high
TCGA-F4-6807	1.353424658	0	0.2065016	5.117851	25.69579	1.202376	1.910867	0.1388189	1.923913604	high
TCGA-F4-6805	1.35890411	0	0.1449514	2.890295	8.091338	0.8007825	0.992156	0.1036291	1.478791095	high
TCGA-AY-4070	1.35890411	1	0.3353782	0.3458979	4.418033	0.5650002	0.6015525	0.449568	1.654886181	high
TCGA-A6-2672	1.378082192	0	0.5798685	0.1179267	1.072936	0.9683318	1.810422	1.471401	4.518929063	high
TCGA-NH-A8F8	1.4	1	0.2015188	1.54551	5.441676	0.3369348	0.6636553	0	1.184109431	low
TCGA-AA-3866	1.419178082	0	0.1557763	0.3287402	2.664036	0.5041295	0.8145102	0	1.116316851	low
TCGA-AA-3846	1.419178082	0	0.1180382	0.4485184	0.5650293	0.1821659	0.3120081	0.09327124	1.157350102	low
TCGA-D5-6538	1.42739726	0	0.03654631	0.8518536	0.15828	0.06656034	0.1469256	0.0391917	1.10857009	low

									5	
TCGA-A6-2680	1.430136986	0	0.191583	0.5532584	1.87738	0.1554291	0.8143795	0.08716088	1.202148231	low
TCGA-A6-2674	1.432876712	0	0.5611118	3.144335	2.160127	1.941668	0.7606146	0.2005759	1.72881517	high
TCGA-CK-4947	1.463013699	0	0.06634871	1.111301	7.321742	0.6191753	0.6825237	0.03984475	1.208497038	low
TCGA-D5-6531	1.479452055	0	0.08785938	1.088552	1.432042	0.1734354	0.3902046	0.04255054	1.156720035	low
TCGA-A6-2677	1.482191781	0	0.06056809	0.1614555	1.883301	0.08315696	0.1896126	0.06994861	1.096635754	low
TCGA-D5-6536	1.487671233	0	0.09460761	2.09755	0.06168132	0.178975	0.7108622	0.09163744	1.316565203	high
TCGA-AA-3831	1.498630137	0	0.06094975	0.07333845	1.693807	0.1295063	0.3694356	0	1.040647547	low
TCGA-AA-3875	1.504109589	0	0.2255107	0.8838201	6.429772	0.4312495	0.3835665	0.2418342	1.388194855	high
TCGA-A6-2681	1.512328767	0	0.04009123	1.374846	9.520868	0.511116	1.050131	0.2257144	1.478075769	high
TCGA-D5-6532	1.520547945	0	0.07368769	0.311596	0.5584901	0.1174289	0.1390066	0.1975539	1.237347805	low
TCGA-DM-A1D6	1.561643836	1	0.02084139	3.736181	0.03240205	0.05722837	0.2443232	0.1203461	1.435192245	high
TCGA-AA-3688	1.583561644	0	0.1501445	0.2601548	1.264409	0.1531333	0.2600209	0	1.048870568	low
TCGA-AA-3713	1.58630137	0	0.2316339	0.525065	2.114256	0.9240217	2.9978	0.1121809	1.492246284	high
TCGA-AA-3715	1.58630137	1	0.189765	0.2859174	6.253293	0.3050397	2.279018	1.858053	6.354796375	high
TCGA-A	1.589	0	0.072	0.587	5.106	1.146	0.352	0.295	1.472	hi

A-3530	041096		18429	4661	226	148	2982	5629	265169	gh
TCGA-AA-3986	1.589041096	0	0.1128028	0.5863588	25.02152	1.315864	1.059786	0.1693552	1.405610611	high
TCGA-NH-A50V	1.610958904	0	0.08988767	0.4598274	0.570971	0.2684825	0.5137039	0.03855766	1.123249486	low
TCGA-F4-6856	1.619178082	0	0.1371537	2.284039	1.617013	0.1631978	0.4671292	0.2059141	1.450136365	high
TCGA-D5-5539	1.632876712	0	0.09973742	2.747908	4.630891	0.6117113	0.5508549	0.2023508	1.531704493	high
TCGA-CM-4751	1.663013699	0	0.1217985	2.003348	8.829931	1.009312	1.618667	0.3577711	1.850739267	high
TCGA-D5-6529	1.682191781	0	0.1381533	0.6746099	3.223752	0.7429451	1.08317	0.188559	1.390657239	high
TCGA-D5-6530	1.701369863	0	0.09202224	0.1371583	3.989716	0.3595214	1.056432	0.04456662	1.156614561	low
TCGA-AA-3955	1.747945205	0	0.09317108	0.3179822	0.5135692	0.1187822	0.1246827	0	1.03869694	low
TCGA-CK-5916	1.761643836	1	0.0518895	1.395458	5.53867	0.2852848	2.642518	0.1947593	1.591762077	high
TCGA-A6-2671	1.775342466	0	0.1434276	0.9712138	1.849251	0.1387163	0.8179693	0.2970116	1.48300917	high
TCGA-AD-6548	1.780821918	0	0.1484775	1.209011	5.682011	0.7292372	0.5522057	0.07308688	1.250558718	low
TCGA-AA-3968	1.832876712	0	0.1670256	0.5008927	3.960034	0.3849265	0.7175767	0	1.114559947	low
TCGA-AA-A02R	1.835616438	1	0.03883222	1.233548	3.251483	0.2121708	5.471251	0.583003	2.702279214	high
TCGA-CM-4746	1.915068493	0	0.02899455	0.1004775	0.2930054	0	0.07531933	0	1.012690406	low

TCGA-AA-3941	2	0	0.03796108	0.7491041	1.918085	0.2177816	0.8162859	0.09498737	1.223642666	low
TCGA-AA-3979	2	0	0.06894317	0.0663653	0.5805896	0.05859628	0.1144212	0.3450234	1.381775772	high
TCGA-AA-3950	2	0	0.05650165	0.2651463	1.855683	0.09004118	0.7705671	0.3181053	1.433286628	high
TCGA-AA-3867	2.002739726	0	0.2412969	1.064592	10.09241	0.4699829	0.5223488	0	1.148364316	low
TCGA-DM-A0XD	2.035616438	1	0.06352449	3.551023	0.1375605	0.1532954	0.3909354	0.2384294	1.601750348	high
TCGA-A6-A566	2.076712329	1	0.3739816	1.385653	1.28353	0.2653789	1.729138	0.1059383	1.389387662	high
TCGA-AA-3819	2.084931507	0	0.1438121	0.7752351	2.761267	0.3422413	0.2532054	0.07197023	1.165629264	low
TCGA-AD-6895	2.090410959	0	0.17191	1.691283	5.623955	0.4383301	2.495251	0.2187247	1.658521214	high
TCGA-AA-A01X	2.167123288	0	0.05599434	0.3528042	8.179143	0.7592895	0.4011083	0.2292721	1.345895983	high
TCGA-AA-3949	2.167123288	0	0.2344043	2.577499	9.839551	0.3333192	1.60035	0	1.352398493	high
TCGA-AD-6965	2.205479452	0	0.07083303	2.405939	0.8862355	0.5805243	0.4497861	0	1.239828025	low
TCGA-DM-A28A	2.205479452	1	0.07354111	1.3956	0.02654187	0.08036251	0.4753213	0.3154574	1.502284323	high
TCGA-AA-3982	2.252054795	0	0.1318128	0.456783	4.329126	0.6301717	0.9188026	0	1.140730463	low
TCGA-AA-3994	2.252054795	0	0.04101432	0.6937331	0.4736819	0.08216753	0.7508756	0	1.107632958	low
TCGA-AA-3869	2.25205479	1	0.09244156	0.1570325	2.857465	0.2010418	0.5414832	0.1632777	1.22994436	low

	5								2	
TCGA-AU-6004	2.257534247	0	0.0751509	0.3617046	3.442795	1.485033	1.093232	0.4889167	1.847248821	high
TCGA-G4-6294	2.350684932	1	0.04071584	0.2038058	0.1371518	0.1799474	0.1833308	0.04075217	1.075199475	low
TCGA-AA-3870	2.498630137	0	0.05870044	1.644312	7.830231	0.5612712	1.982325	0.2643877	1.666237002	high
TCGA-AA-3862	2.504109589	0	0.09858925	0.9727546	1.245371	0.3246984	0.5975805	0	1.131403777	low
TCGA-AA-3861	2.504109589	0	0.05209714	0.17194	3.986125	0.3510651	0.3093327	0.1117363	1.164890277	low
TCGA-AA-3877	2.583561644	0	0.1183705	0.8374919	1.016767	0.196181	1.850075	0.3554283	1.669422595	high
TCGA-AA-A01I	2.583561644	0	0.03120316	0.3990555	0.4054174	0.05114618	0.1621133	0	1.041006428	low
TCGA-AA-3858	2.589041096	0	0.06591429	0.4672203	1.877188	0.1451478	0.6537719	0	1.089395847	low
TCGA-AA-3860	2.589041096	0	0.1931821	0.6450445	5.734611	0.7234605	0.7318358	0.0906349	1.24321081	low
TCGA-QG-A5Z2	2.608219178	0	0.1306215	0.1697456	5.280002	8.301375	1.008524	0.2941607	2.180310968	high
TCGA-AA-3855	2.671232877	0	0.1356084	0.9489095	1.159565	0.2327294	0.4696943	0.2349162	1.373629251	high
TCGA-AZ-4615	2.745205479	0	0.1481465	1.155117	0.7485496	0.07554772	1.937033	0.07413933	1.328216741	high
TCGA-AA-3947	2.750684932	0	0.1644132	0.5113195	1.022451	0.06449459	0.5037555	0	1.079452253	low
TCGA-AA-A01T	2.753424658	0	0.3561553	0.1071369	1.349677	0.009459502	0.2184469	0	1.034856867	low
TCGA-A	2.753	0	0.119	0.268	12.76	0.712	0.817	0.099	1.230	lo

A-3815	424658		7134	8866	977	2287	7626	85014	501921	w
TCGA-AA-3851	2.756164384	0	0.06541745	0.8415271	6.0699	0.4549066	0.3656184	0	1.111670509	low
TCGA-F4-6808	2.805479452	0	0.04239156	0.2652422	0.321292	0.09457743	0.3273035	0.05303673	1.097562262	low
TCGA-AA-3956	2.835616438	0	0.3473031	0.8525085	2.544521	0.3431472	0.5262784	0	1.127559024	low
TCGA-AA-3975	2.838356164	0	0.3894599	0.8810091	2.88618	0.3144598	0.5002306	0.09745184	1.22910379	low
TCGA-AA-A010	2.915068493	0	0.1076864	0.9847688	1.450971	0.3432187	1.10341	0.5389124	1.894334512	high
TCGA-AA-3502	2.917808219	0	0.02780354	0.1204377	0.0702424	0.3544624	0.7403106	0.1043563	1.187331073	low
TCGA-AA-A01R	2.917808219	0	0.09377967	0.3823333	5.128695	0.3938383	0.9266811	0.9110266	2.513344045	high
TCGA-AA-3812	2.920547945	0	0.08827128	0.4248536	3.568114	0.1250394	1.155007	0.4417502	1.665324705	high
TCGA-F4-6855	2.950684932	0	0.0954721	3.286426	0.6560617	0.2288255	0.720878	0.05733437	1.380559253	high
TCGA-G4-6586	2.983561644	0	0.03203848	0.3161154	1.834674	0.2927246	0.6426941	0.8417606	2.288503361	high
TCGA-F4-6703	2.989041096	0	0.4875946	3.398032	15.45413	0.8194158	1.603348	0.1512489	1.685772089	high
TCGA-G4-6314	2.994520548	0	0.1800637	2.717145	11.63544	0.2748254	0.7708036	0	1.281008048	high
TCGA-DM-A28F	2.997260274	1	0.04477135	1.243665	3.705686	0.2500569	0.2806036	0.09602421	1.219157351	low
TCGA-AA-3692	3	1	0.1150712	0.3987668	4.511882	0.2405917	0.4224753	0.06910433	1.14274901	low
TCGA-A	3.002	0	0.070	0.271	1.138	0.047	0.878	0	1.087	lo

A-3854	739726		41506	1286	531	87782	0047		750857	w
TCGA-G4-6315	3.060273973	0	0.06256573	0.1521507	3.06147	0.7052811	0.2509195	0.09887614	1.166887336	low
TCGA-AA-3841	3.079452055	0	0.1125143	0.2527169	6.31676	0.8447167	0.9634383	0.3753822	1.593261226	high
TCGA-AA-3842	3.084931507	0	0.0365102	0.7116871	0.2767163	0.05818276	0.5374424	0.1370354	1.231505184	low
TCGA-AA-A01Z	3.084931507	0	0.01401261	0.424893	0.3540124	0.1429155	0.3458062	0.1051884	1.164423924	low
TCGA-AA-3495	3.087671233	0	0.1335751	0.2436263	4.404765	0.2419946	0.3469888	0.1055481	1.163360123	low
TCGA-AA-3685	3.087671233	0	0.1052133	0.4149978	12.00679	0.1701219	0.3110882	0.03852701	1.102454738	low
TCGA-AA-A01P	3.17260274	1	0.1642426	1.707495	5.477205	0.523475	2.564665	0.4109729	1.986460715	high
TCGA-DM-A0XF	3.183561644	1	0.2283545	0.223639	0.5417948	0.06835113	0.232121	0.1788717	1.221196106	low
TCGA-AA-3837	3.249315068	0	0.182206	2.762441	6.674671	0.4645823	0.5324822	0.341941	1.724960255	high
TCGA-AA-A024	3.254794521	1	0.2076448	0.328836	0.4738236	0.01707888	0.1798	0	1.041712699	low
TCGA-5M-AATE	3.287671233	0	0.02937654	0.3878141	0.5937312	0.2354102	0.3343175	0	1.063150368	low
TCGA-AA-A02Y	3.331506849	0	0.01840359	0.05314637	0.3022144	0.04985762	0.1195178	0	1.015149126	low
TCGA-AA-A02F	3.331506849	0	0.1550041	1.256793	0.1807384	0.02280138	0.1806786	0	1.102449371	low
TCGA-CK-4950	3.361643836	0	0.07542059	0.8041904	2.97049	0.6385503	0.5592945	0.0580676	1.194471089	low

TCGA-AA-A03J	3.41369863	0	0.07296567	0.3863057	1.966286	0.3178274	0.8108244	0.5477308	1.797261497	high
TCGA-AA-A02W	3.416438356	0	0.1111214	1.146069	1.015995	0.1619049	0.224514	0.1588866	1.27198043	high
TCGA-D5-6534	3.605479452	0	0.274902	3.548355	59.20153	1.311599	3.348765	0.6253347	3.022238721	high
TCGA-DM-A1DB	3.693150685	1	0.04507719	0.05207004	0.5648561	0.04827321	0.2248268	0.1624227	1.181969769	low
TCGA-AA-3678	3.917808219	0	0.06954168	0.2409892	1.686614	0.2009569	0.4576438	0.06960373	1.130017759	low
TCGA-AA-3675	3.920547945	0	0.01647271	0.2514432	0.570739	0.04800175	0.2648983	0.1059905	1.14061458	low
TCGA-G4-6627	3.928767123	0	0.3735522	5.04551	46.3694	2.197431	1.502686	0.0441597	1.821492123	high
TCGA-AA-3673	4.169863014	0	0.05856374	1.014732	5.144263	0.648984	0.4876006	0.06763385	1.215163857	low
TCGA-AZ-4313	4.197260274	0	0.02370278	0.182532	1.197646	0.03021825	0.2052428	0.2372393	1.269744628	high
TCGA-AA-3972	4.249315068	0	0.07088118	0.1488673	0.3907048	0.1560853	0.4854296	0	1.055607371	low
TCGA-G4-6628	4.4	0	0.1577678	0.4859798	9.778546	0.5363611	1.473127	0	1.185481526	low
TCGA-AA-3864	4.416438356	0	0.07505812	0.3334689	1.050234	0.1030511	0.2949681	0.0866828	1.134913734	low
TCGA-G4-6304	4.468493151	0	0.07938186	0.2750893	1.283514	0.04722785	0.1512212	0.0794527	1.109184926	low
TCGA-AA-3664	4.501369863	0	0.294552	5.050526	1.116227	0.2112295	0.4543134	0	1.445813401	high
TCGA-G4-6307	4.58630137	0	0.07535438	0.1771247	4.197084	1.056748	0.1866439	0.2894085	1.40377479	high

									1	
TCGA-A6-A56B	4.687671233	1	0.06656134	1.409596	0.5231628	0.08485787	1.293599	0.6662073	2.174355253	high
TCGA-AA-3506	4.835616438	0	0.06120313	0.9190689	2.845055	0.3121071	0.4451657	0	1.113953775	low
TCGA-AZ-4315	4.865753425	0	0.04102167	0.45411	2.003639	0.1830423	0.6512138	0.1026457	1.193390769	low
TCGA-SS-A7HO	5.010958904	0	0.1173682	0.5623308	0.1347805	0.06801395	0.1362758	0.1201428	1.171823664	low
TCGA-DM-A28G	5.065753425	1	0.03609613	0.2015294	0.2431805	0.03988254	0.1666971	0.07225668	1.096048155	low
TCGA-AA-3655	5.084931507	0	0.08503173	0.5612729	0.7672242	0.1200203	0.3418496	0	1.070826198	low
TCGA-A6-A567	5.153424658	1	0.03070578	1.418767	1.179134	0.3601456	0.4679523	0.2765986	1.472520333	high
TCGA-AA-3509	5.246575342	0	0.07108767	1.010653	0.4420793	0.1115427	0.3693298	0	1.102431327	low
TCGA-AA-3510	5.331506849	0	0.1560597	0.5257855	8.516158	0.3382281	0.4954855	0.07809946	1.175830904	low
TCGA-G4-6303	5.487671233	1	0.06300259	1.072822	1.119669	0.246502	0.4778184	0.03261662	1.155955725	low
TCGA-AD-6889	5.632876712	0	0.071599	0.347366	0.3183603	0.02920969	0.2653503	0.3009841	1.364910661	high
TCGA-CK-4951	5.846575342	1	0.1279182	0.6353774	0.9824379	0.1369877	1.657039	0.2304582	1.451574604	high
TCGA-G4-6299	6.21369863	0	0.1780459	10.61204	4.011441	0.2455931	3.654595	0.2191043	3.114607402	high
TCGA-AA-3660	6.506849315	0	0.130991	0.3954737	1.895346	0.2618833	0.2758298	0.04469586	1.107517861	low
TCGA-D	6.780	1	0.004	0.109	0.550	0.030	0.272	0	1.028	lo

M-A28C	821918		739371	4918	7796	2107	9048		426698	w
TCGA-G4-6297	6.865753425	0	0.2472662	2.715096	3.486638	0.86873	1.003322	0	1.342767108	high
TCGA-AA-3697	7.087671233	0	0.1738229	0.3792664	1.795608	0.3250188	0.3578879	0	1.074560803	low
TCGA-DM-A1HA	7.123287671	0	0.05809922	3.322053	0.1643946	0.2459113	1.074584	0.6280315	2.35147834	high
TCGA-G4-6625	7.649315068	0	0.1932422	1.424277	18.3623	2.283436	1.356113	0.1732071	1.597222811	high
TCGA-DM-A1D4	7.728767123	1	0.0401842	0.05157555	0.2256015	0.01423059	0.4639411	0.03351671	1.070180054	low
TCGA-DM-A28M	7.931506849	0	0.02245177	0.7721475	0.08250441	0.01301062	0.07599677	0	1.056634013	low
TCGA-DM-A28K	8.18630137	0	0.08209924	0.8128743	0.148153	0.03738102	0.2107308	0.2201049	1.303930842	high
TCGA-AZ-6601	8.334246575	1	0.1506721	1.429663	16.74885	1.711652	1.628512	0.04039462	1.403123974	high
TCGA-CK-4948	8.536986301	0	0.09329659	0.6534974	1.384128	0.3467038	0.6823814	0.1490104	1.274491763	high
TCGA-DM-A28H	9.756164384	0	0.09430115	0.2927498	1.57239	0.168312	0.1388145	0.07078897	1.108507744	low
TCGA-DM-A0X9	9.975342466	0	0.02320797	0.2113726	0.3066917	0.05007103	0.1731333	0.1608139	1.186961818	low
TCGA-DM-A28E	9.994520548	0	0.04719873	0.05300623	0.3444771	0.03342934	0.09082105	0	1.013148913	low
TCGA-DM-A1D0	10.887671233	0	0.04891049	0.1307824	0.05491851	0.009237795	0.6227267	0.2175739	1.280294525	high
TCGA-DM-A282	11.597260274	0	0.0161553	0.3732297	0.09329027	0.01471151	0.1159081	0.0692988	1.098844441	low

TCGA-DM-A1D9	11.6986301 4	0	0.04166968	0.300837	0.1148439	0.06036811	0.1672886	0.0284365	1.06219703 5	low
