# Supplementary figures and images for: Alteration in the Immune Microenvironment Based on APC Status in MSS/pMMR Colon Cancer
Source: Dis Markers. 2022 Jul 27;2022:3592990. doi: 10.1155/2022/3592990 (PMC9348928; doi:10.1155/2022/3592990)

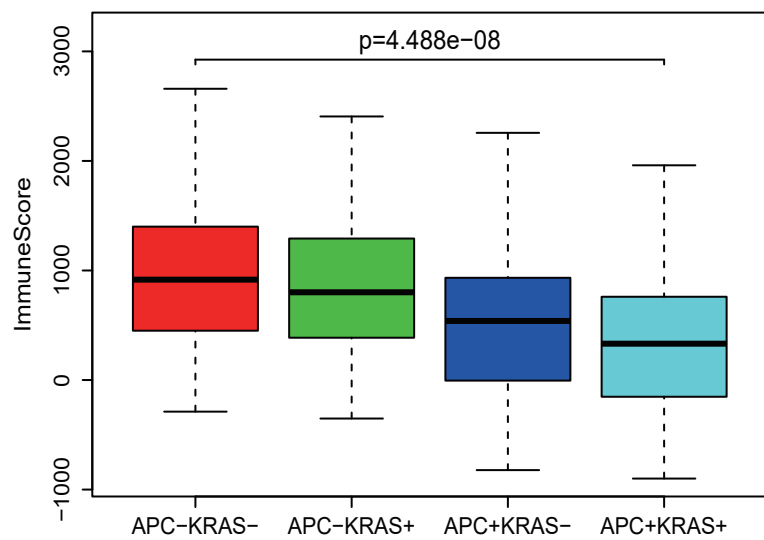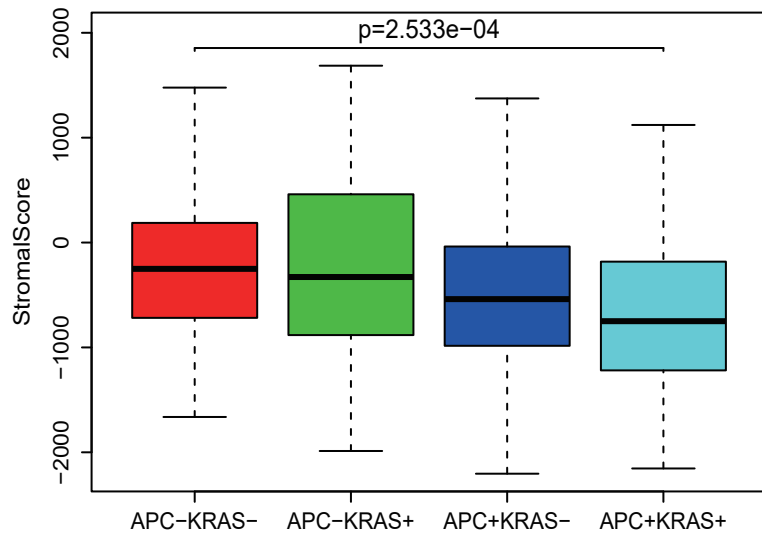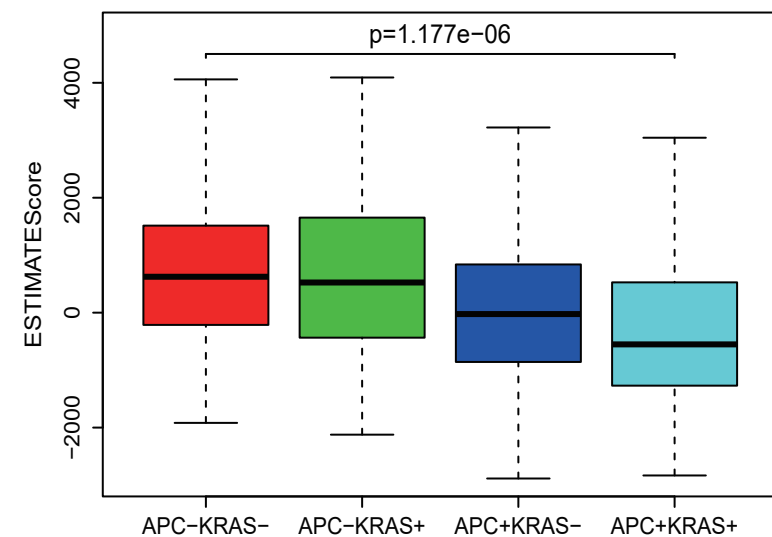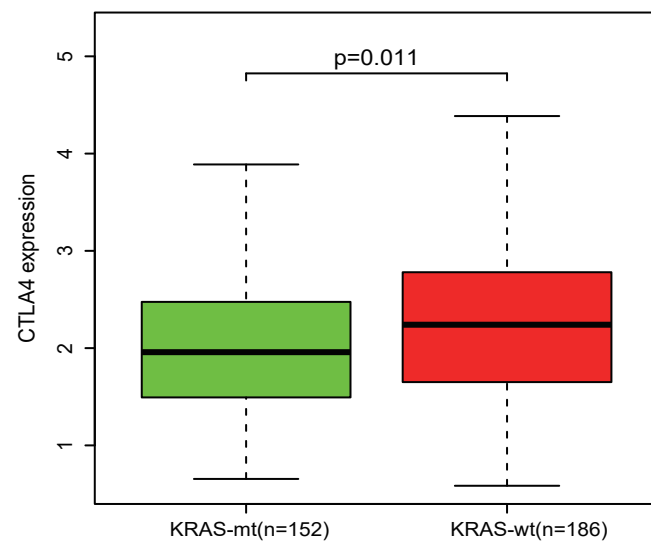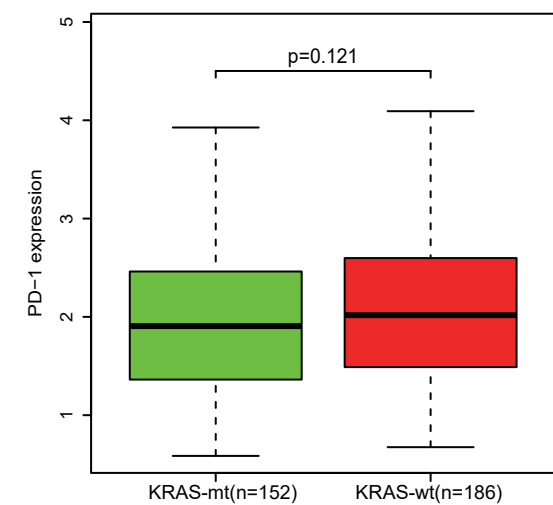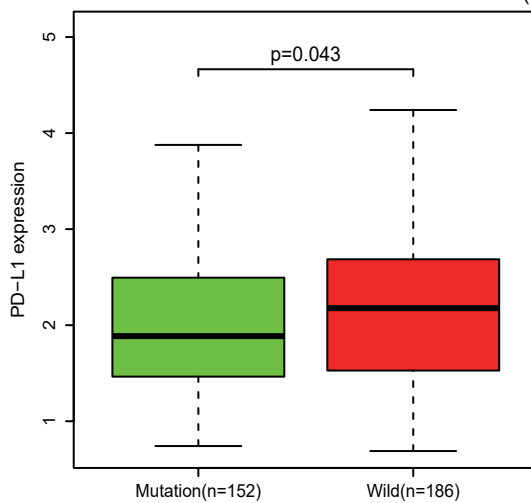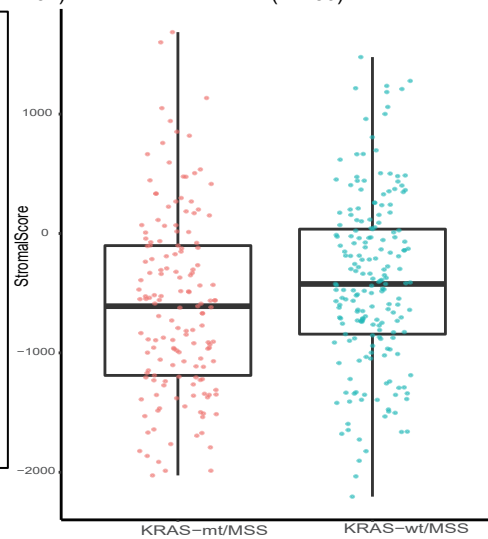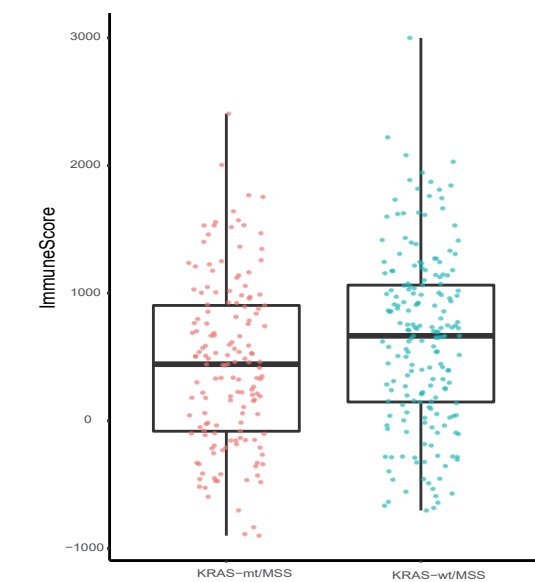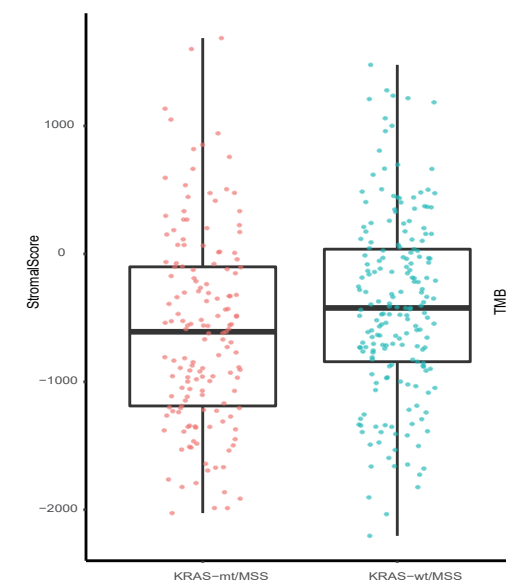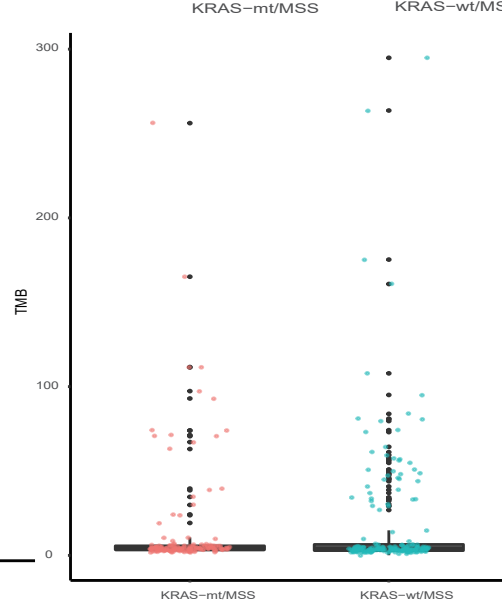

Supplement: Supplementary Materials — Supplement Table S1: all different expression genes (DEGs) listed between APC-wt and APC-mt MSS/pMMR colon cancer from the overall gene expression profile of all 338 colon cancer cases obtained from TCGA database. Supplementary Table S2: APC-mt vs. APC-wt MSS/pMMR colon cancer, 379 genes were downregulated, and 117 genes were upregulated. Supplementary Table S3: APC-wt MSS/pMMR (77 samples) COAD samples in the cohort retrieved from TCGA performed gene set enrichment analysis enriched (GSEA) in 115 KEGG pathways. Supplementary Table S4: APC-wt MSS/pMMR COAD samples in the cohort retrieved from TCGA performed gene set enrichment analysis downregulated (GSEA) in 62 KEGG pathways. Supplementary Table S5: 65 overlapping genes among the DEGs (496 genes related to APC status) and immunophenotypes (1297 genes shown in Supplementary Table S6). Supplementary Table S6: 1297 immune-related genes. Supplementary Table S7: Lasso and Cox regression analyze APC-related DEGs constructing immune scoring model. Supplement Figure 1: the expression of CTLA4, PD-L1, and immune score and stromal score in the KRAS-mt/MSS group was downregulated compared with KRAS-wt/MSS. Supplement Figure 2: the expression of not only PD-1, PD-L1, and CTLA4 but also immune score, ESTIMATE score, and stromal score did not differ between TP53-wt/MSS and TP53-mt/MSS groups. Supplement Figure 3: in the combination of different genotypes of KRAS, TP53 and APC, the TMB and immune score are significantly higher in wild type than KRAS/TP53/APC mutant type colon cancer. Supplement Figure 4: neither mutations in KRAS nor TP53 could affect the proportion of infiltrating immune cell types in MSS/pMMR colon cancer. [file 3592990.f1.zip › supplement figure S1.pdf]

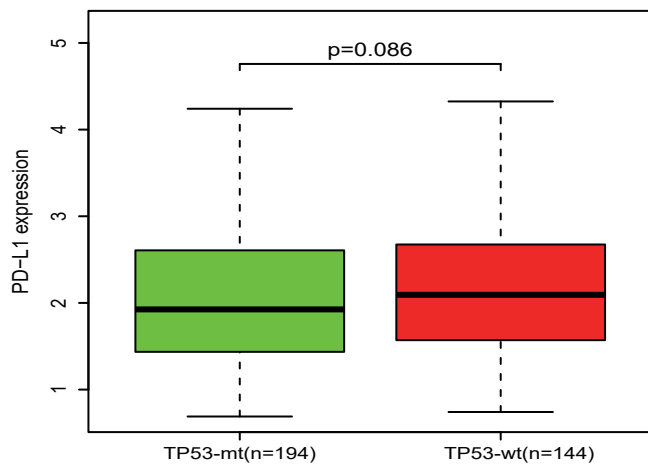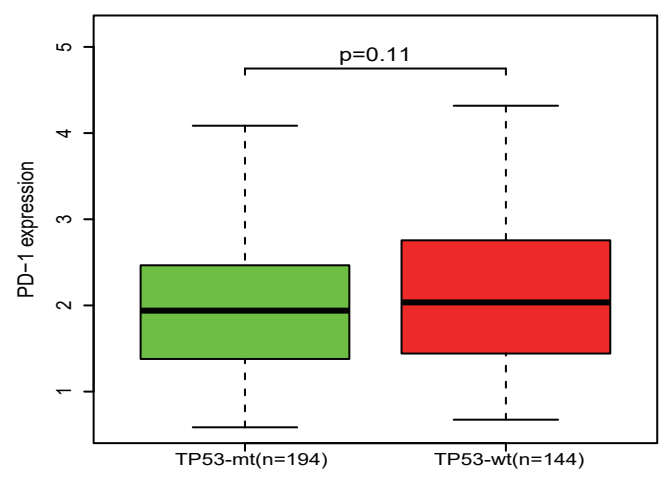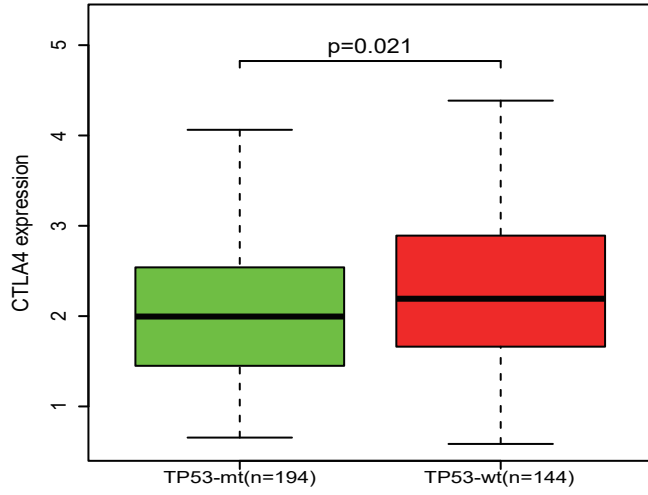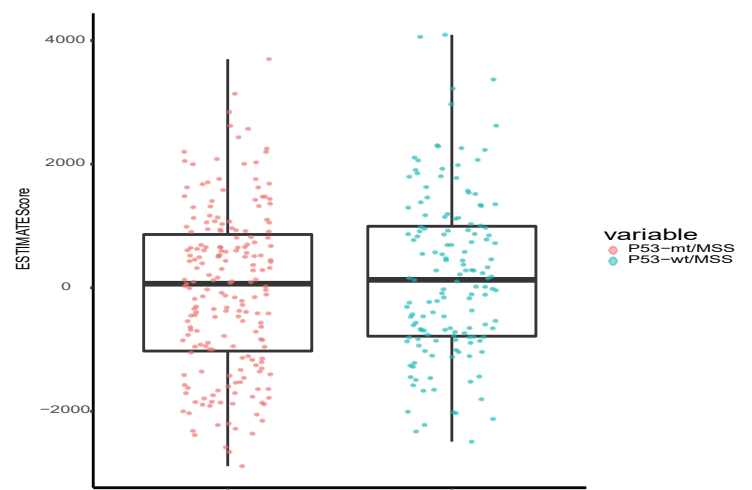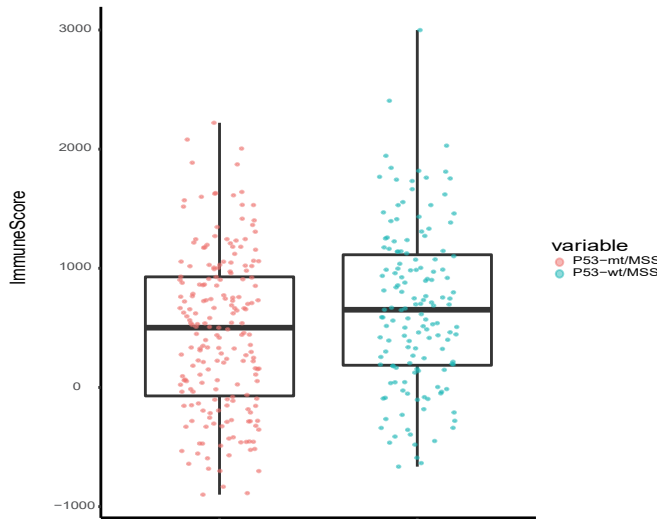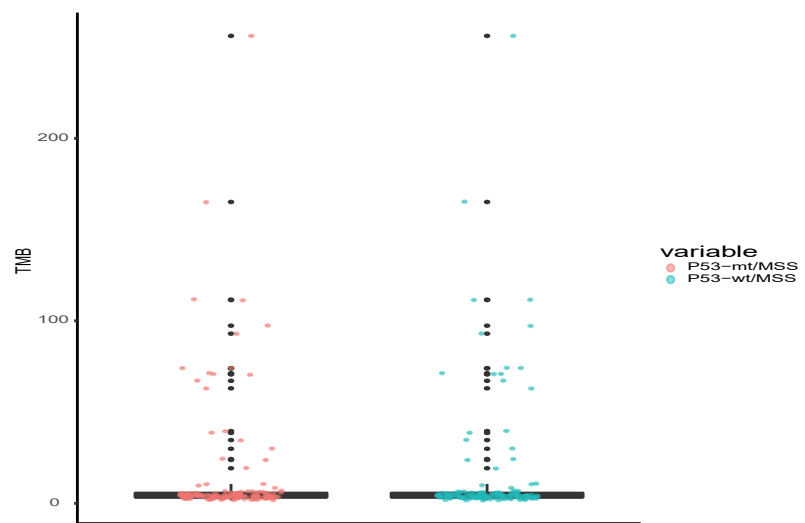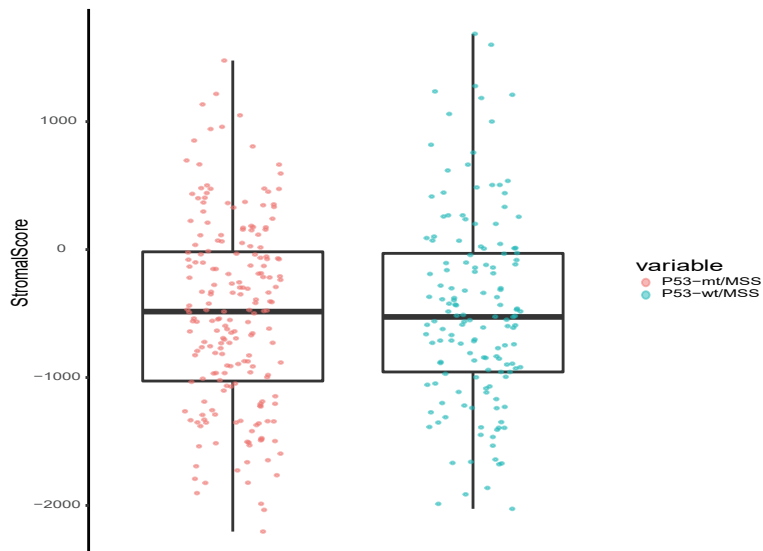

Supplement: Supplementary Materials — Supplement Table S1: all different expression genes (DEGs) listed between APC-wt and APC-mt MSS/pMMR colon cancer from the overall gene expression profile of all 338 colon cancer cases obtained from TCGA database. Supplementary Table S2: APC-mt vs. APC-wt MSS/pMMR colon cancer, 379 genes were downregulated, and 117 genes were upregulated. Supplementary Table S3: APC-wt MSS/pMMR (77 samples) COAD samples in the cohort retrieved from TCGA performed gene set enrichment analysis enriched (GSEA) in 115 KEGG pathways. Supplementary Table S4: APC-wt MSS/pMMR COAD samples in the cohort retrieved from TCGA performed gene set enrichment analysis downregulated (GSEA) in 62 KEGG pathways. Supplementary Table S5: 65 overlapping genes among the DEGs (496 genes related to APC status) and immunophenotypes (1297 genes shown in Supplementary Table S6). Supplementary Table S6: 1297 immune-related genes. Supplementary Table S7: Lasso and Cox regression analyze APC-related DEGs constructing immune scoring model. Supplement Figure 1: the expression of CTLA4, PD-L1, and immune score and stromal score in the KRAS-mt/MSS group was downregulated compared with KRAS-wt/MSS. Supplement Figure 2: the expression of not only PD-1, PD-L1, and CTLA4 but also immune score, ESTIMATE score, and stromal score did not differ between TP53-wt/MSS and TP53-mt/MSS groups. Supplement Figure 3: in the combination of different genotypes of KRAS, TP53 and APC, the TMB and immune score are significantly higher in wild type than KRAS/TP53/APC mutant type colon cancer. Supplement Figure 4: neither mutations in KRAS nor TP53 could affect the proportion of infiltrating immune cell types in MSS/pMMR colon cancer. [file 3592990.f1.zip › supplement figure S2.pdf]

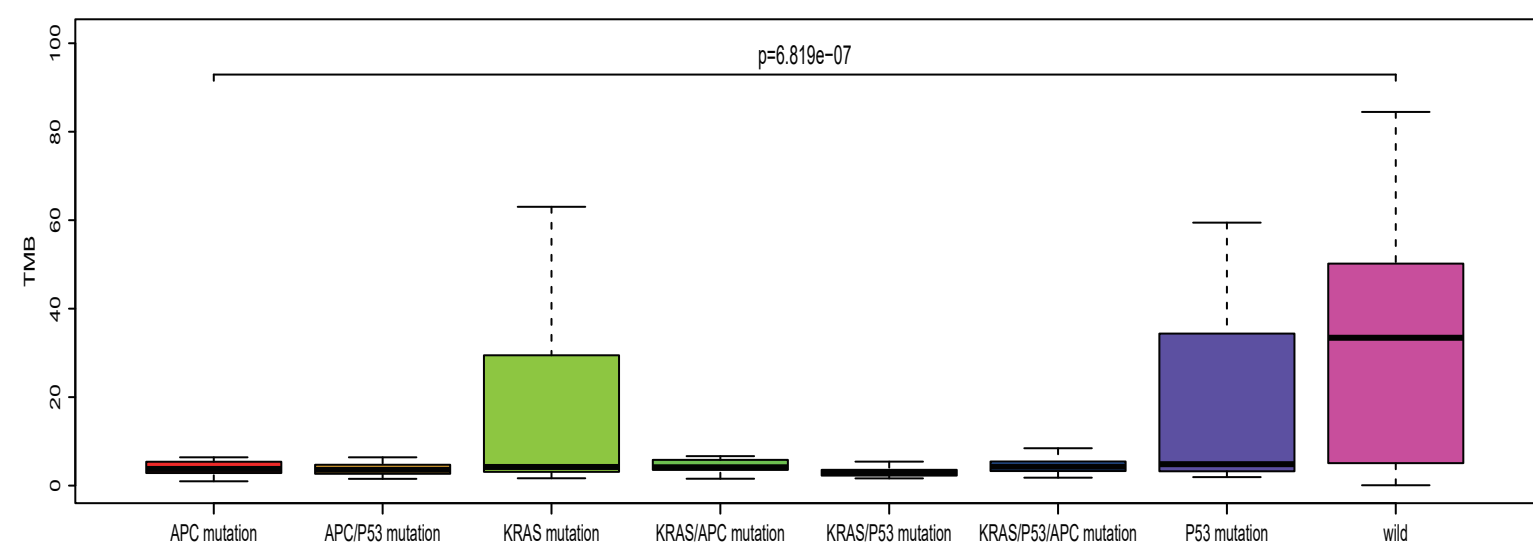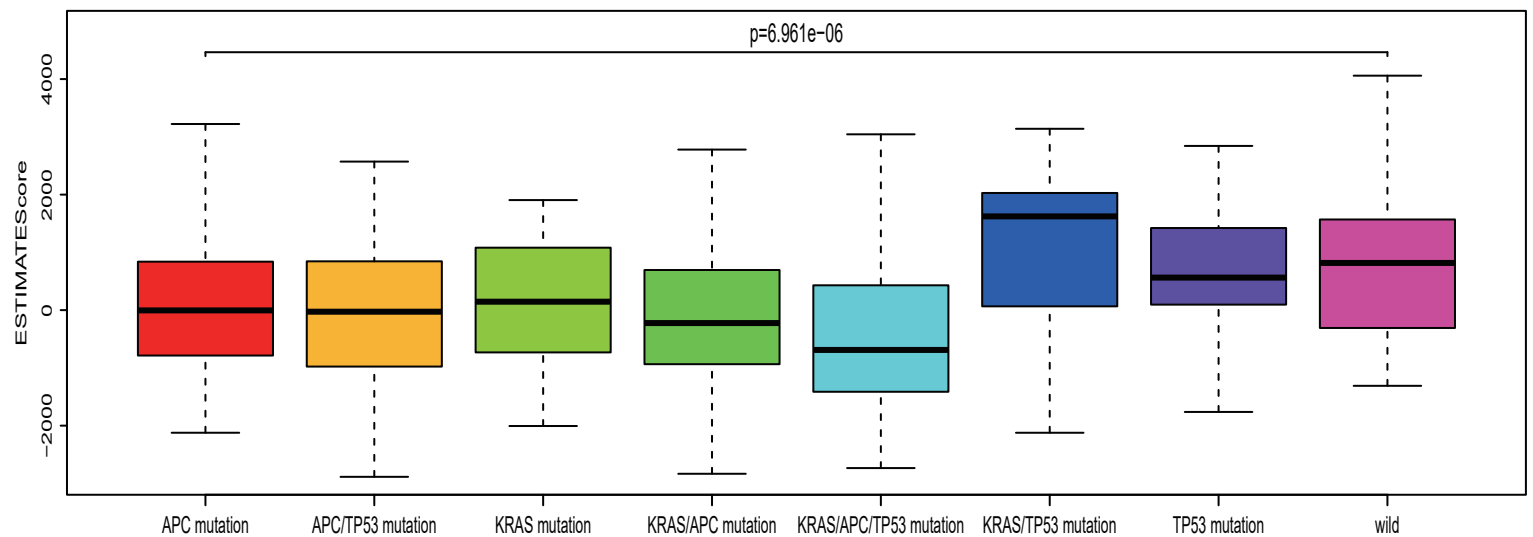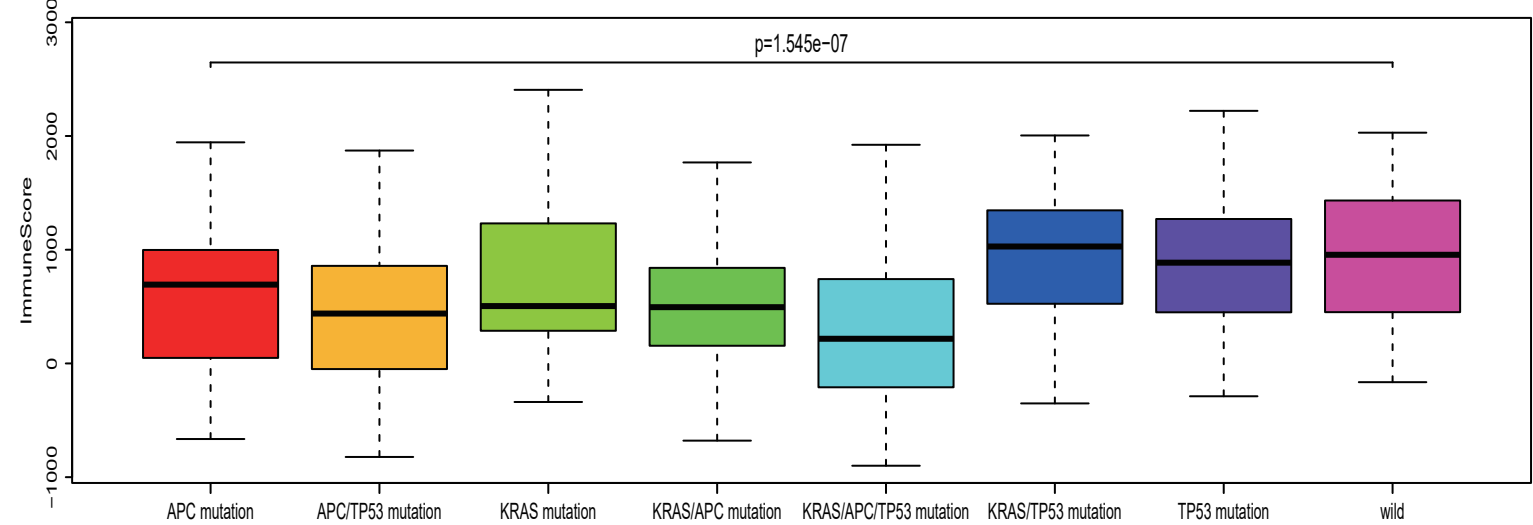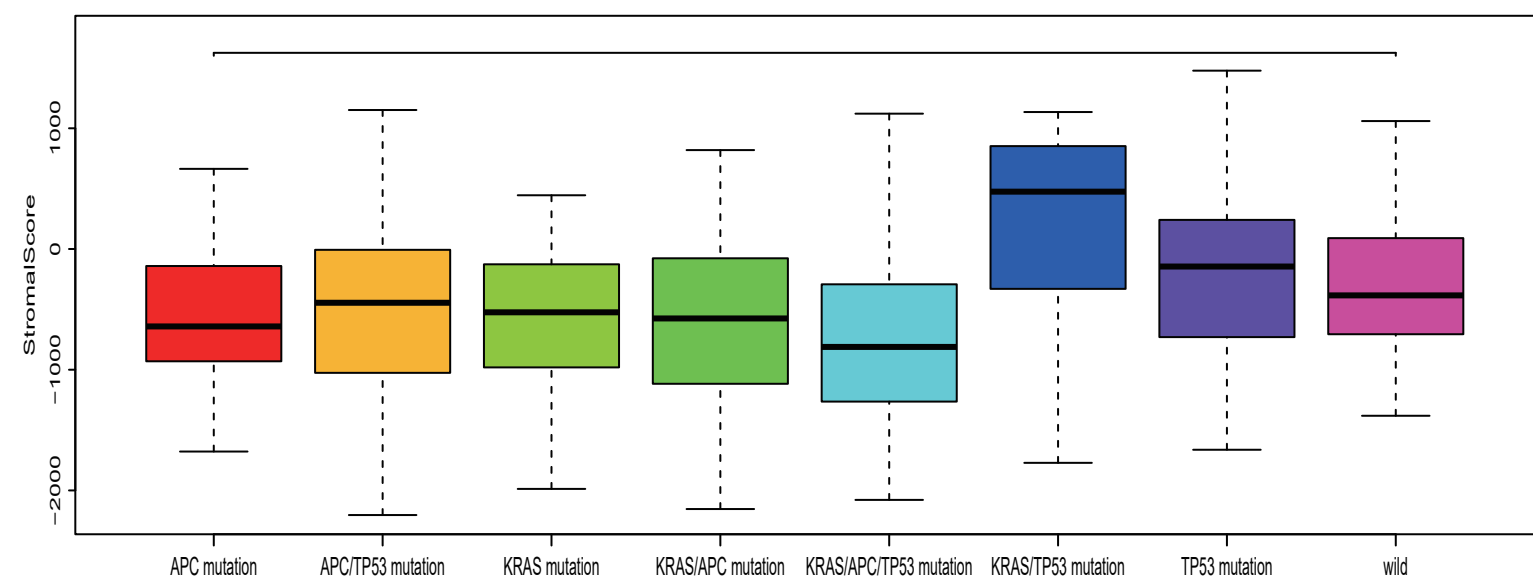

Supplement: Supplementary Materials — Supplement Table S1: all different expression genes (DEGs) listed between APC-wt and APC-mt MSS/pMMR colon cancer from the overall gene expression profile of all 338 colon cancer cases obtained from TCGA database. Supplementary Table S2: APC-mt vs. APC-wt MSS/pMMR colon cancer, 379 genes were downregulated, and 117 genes were upregulated. Supplementary Table S3: APC-wt MSS/pMMR (77 samples) COAD samples in the cohort retrieved from TCGA performed gene set enrichment analysis enriched (GSEA) in 115 KEGG pathways. Supplementary Table S4: APC-wt MSS/pMMR COAD samples in the cohort retrieved from TCGA performed gene set enrichment analysis downregulated (GSEA) in 62 KEGG pathways. Supplementary Table S5: 65 overlapping genes among the DEGs (496 genes related to APC status) and immunophenotypes (1297 genes shown in Supplementary Table S6). Supplementary Table S6: 1297 immune-related genes. Supplementary Table S7: Lasso and Cox regression analyze APC-related DEGs constructing immune scoring model. Supplement Figure 1: the expression of CTLA4, PD-L1, and immune score and stromal score in the KRAS-mt/MSS group was downregulated compared with KRAS-wt/MSS. Supplement Figure 2: the expression of not only PD-1, PD-L1, and CTLA4 but also immune score, ESTIMATE score, and stromal score did not differ between TP53-wt/MSS and TP53-mt/MSS groups. Supplement Figure 3: in the combination of different genotypes of KRAS, TP53 and APC, the TMB and immune score are significantly higher in wild type than KRAS/TP53/APC mutant type colon cancer. Supplement Figure 4: neither mutations in KRAS nor TP53 could affect the proportion of infiltrating immune cell types in MSS/pMMR colon cancer. [file 3592990.f1.zip › supplement figure S3.pdf]

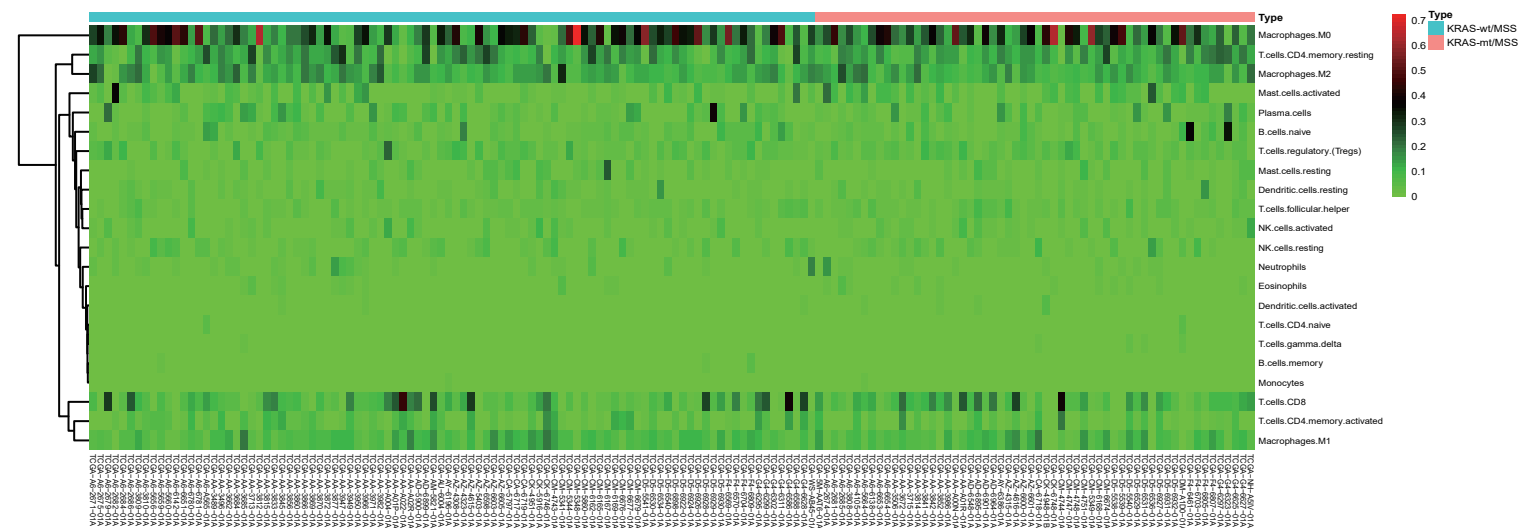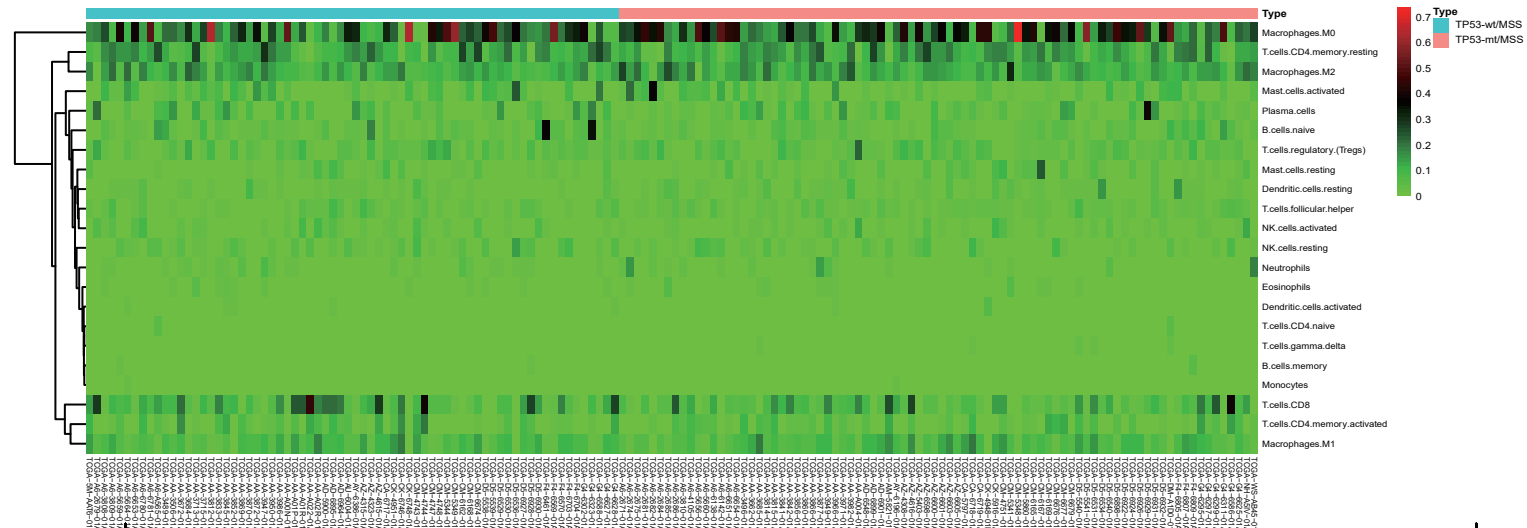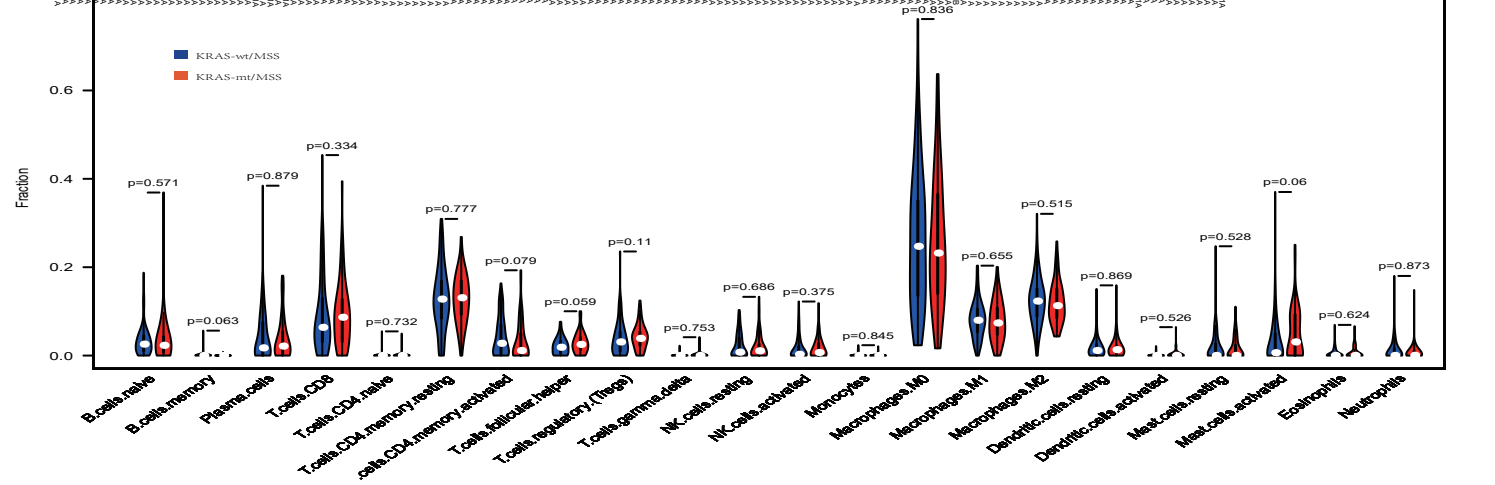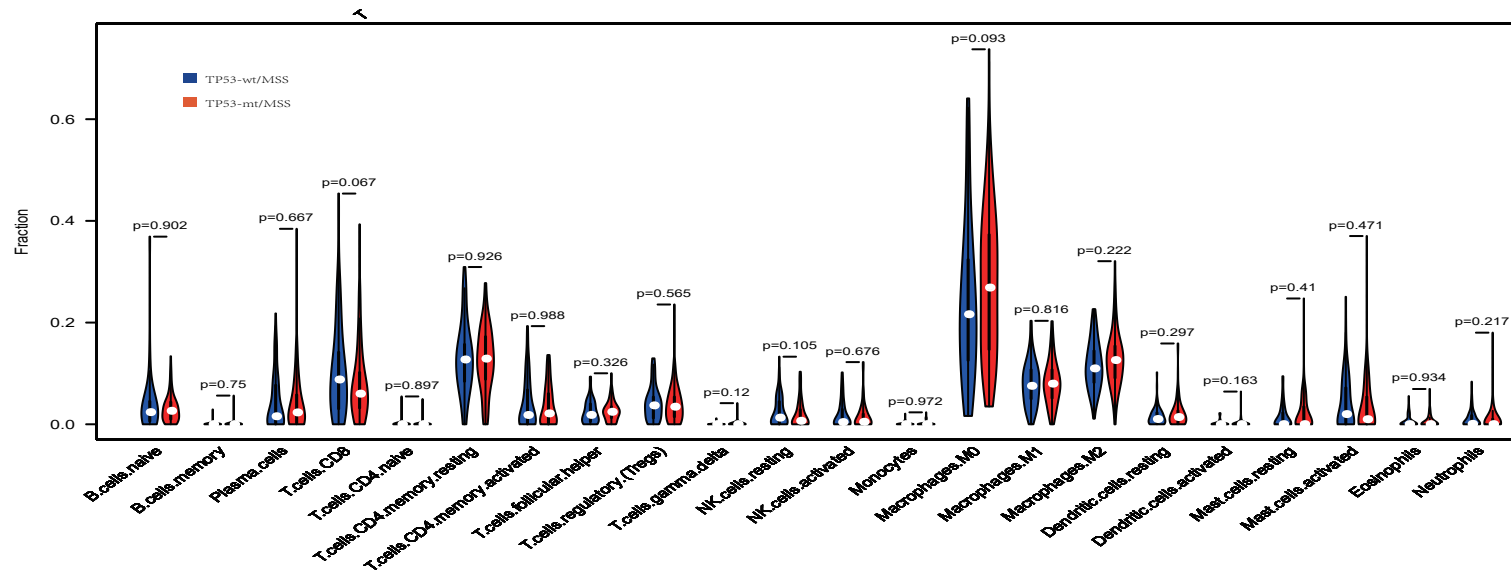

Supplement: Supplementary Materials — Supplement Table S1: all different expression genes (DEGs) listed between APC-wt and APC-mt MSS/pMMR colon cancer from the overall gene expression profile of all 338 colon cancer cases obtained from TCGA database. Supplementary Table S2: APC-mt vs. APC-wt MSS/pMMR colon cancer, 379 genes were downregulated, and 117 genes were upregulated. Supplementary Table S3: APC-wt MSS/pMMR (77 samples) COAD samples in the cohort retrieved from TCGA performed gene set enrichment analysis enriched (GSEA) in 115 KEGG pathways. Supplementary Table S4: APC-wt MSS/pMMR COAD samples in the cohort retrieved from TCGA performed gene set enrichment analysis downregulated (GSEA) in 62 KEGG pathways. Supplementary Table S5: 65 overlapping genes among the DEGs (496 genes related to APC status) and immunophenotypes (1297 genes shown in Supplementary Table S6). Supplementary Table S6: 1297 immune-related genes. Supplementary Table S7: Lasso and Cox regression analyze APC-related DEGs constructing immune scoring model. Supplement Figure 1: the expression of CTLA4, PD-L1, and immune score and stromal score in the KRAS-mt/MSS group was downregulated compared with KRAS-wt/MSS. Supplement Figure 2: the expression of not only PD-1, PD-L1, and CTLA4 but also immune score, ESTIMATE score, and stromal score did not differ between TP53-wt/MSS and TP53-mt/MSS groups. Supplement Figure 3: in the combination of different genotypes of KRAS, TP53 and APC, the TMB and immune score are significantly higher in wild type than KRAS/TP53/APC mutant type colon cancer. Supplement Figure 4: neither mutations in KRAS nor TP53 could affect the proportion of infiltrating immune cell types in MSS/pMMR colon cancer. [file 3592990.f1.zip › supplement figure S4.pdf]
